# Improved AAV9-based gene therapy design for *SURF1*-related Leigh syndrome with minimal toxicity

**DOI:** 10.1016/j.omtm.2025.101554

**Published:** 2025-08-11

**Authors:** Qinglan Ling, Matthew Rioux, Harrison Higgs, Yuhui Hu, Scarlett E. Dwyer, Steven J. Gray

**Affiliations:** 1 Department of Genetic & Cellular Medicine, Horae Gene Therapy Center, UMass Chan Medical School, Worcester, MA 01605, USA; 2 Department of Microbiology, UMass Chan Medical School, Worcester, MA 01605, USA; 3 Department of Pediatrics, UT Southwestern Medical Center, Dallas, TX 75390, USA; 4 Department of Neurology, UT Southwestern Medical Center, Dallas, TX 75390, USA; 5 Department of Molecular Biology, UT Southwestern Medical Center, Dallas, TX 75390, USA; 6 McDermott Center for Human Growth and Development, UT Southwestern Medical Center, Dallas, TX 75390, USA

**Keywords:** gene therapy, gene replacement therapy, adeno-associated virus, AAV, Leigh syndrome, SURF1, mitochondrial diseases, overexpression toxicity

## Abstract

Surfeit locus protein 1 (SURF1)-related Leigh syndrome is an early-onset neurodegenerative disorder characterized by a reduction in complex IV activity that disrupts mitochondrial function. Currently, there are no disease-modifying treatments available. Previously, we reported that a gene replacement therapy for *SURF1*-related Leigh syndrome was developed, which showed improved complex IV activity and restored exercise-induced lactate acidosis, as well as a high safety profile in wild-type (WT) mice. However, further investigations of this original SURF1 vector design uncovered cytotoxicity in multiple tissues of WT rats despite having minimal immune responses. We hypothesized that this cytotoxicity was elicited by SURF1 protein overexpression driven by a strong ubiquitous promoter, CBh. Here, we report the development of an improved gene therapy for SURF1 Leigh syndrome by utilizing a different promoter and polyadenylation sequence. Our data showed that, with lower SURF1 protein expression, the new design conferred a similar level of efficacy, but with minimal cytotoxicity in mice or rats. We propose this new vector design as a promising therapeutic candidate for *SURF1*-related Leigh syndrome, warranting further translational studies.

## Graphical abstract

**Figure:**
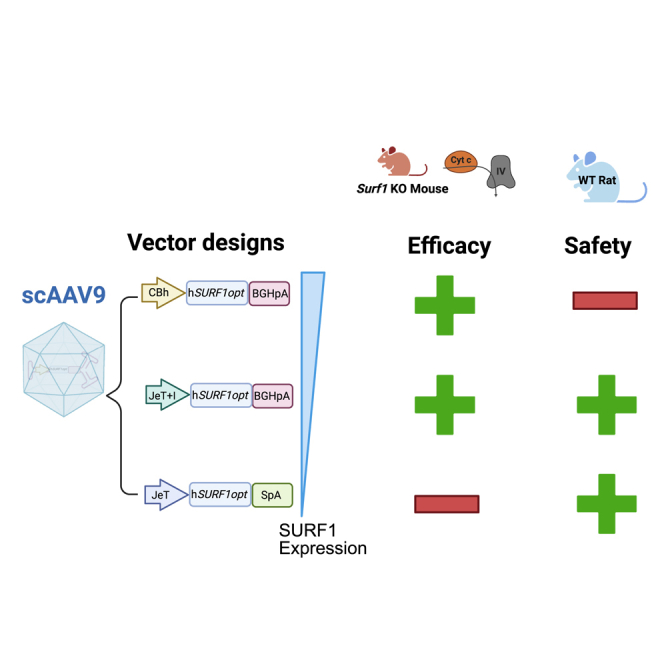

## Teaser

Ling and colleagues identified transgene overexpression-induced cytotoxicity in their previously reported AAV9- based gene replacement therapy for SURF1-related Leigh syndrome. By optimizing the vector design, they now developed an improved version that showed efficacy equivalent to their original design, but without associated cytotoxicity in wild-type rats.

## Introduction

Leigh syndrome (OMIM: #256000), or subacute necrotizing encephalomyopathy, is an autosomal recessive early-onset mitochondrial disorder with no approved treatment other than palliative care. It is estimated that 1 in every 40,000 people worldwide are diagnosed with Leigh syndrome.^1^ The disease is characterized by asymmetric necrotic lesions in various brain regions, including the basal ganglia, diencephalon, brain stem, and spinal cord. Patients also show peripheral neuropathy, myopathy, and respiratory failure, which eventually leads to death.^1^^,^^2^ One of the most common causes of Leigh syndrome are genetic mutations in the surfeit locus protein 1 (*SURF1*) gene,^3^^,^^4^ and these mutations are typically autosomal recessive.^4^ Like 99% of mitochondrial proteins, *SURF1* is encoded in the nuclear genome and localizes in the inner mitochondrial membrane.^5^ The function of SURF1 is proposed to be involved in complex IV/cytochrome *c* oxidase (COX) assembly. Thus, the loss of SURF1 function results in impaired complex IV assembly and reduced activity, leading to dysfunction of the mitochondrial oxidative phosphorylation system.^6^^,^^7^ In most cases, patients with *SURF1*-related Leigh syndrome show 10%–30% remaining COX activity as examined with muscle or skin fibroblasts.^2^^,^^8^^,^^9^^,^^10^ Additionally, 81% of patients show elevated blood lactate levels.^9^

Gene therapy, delivered by adeno-associated virus (AAV) vector, has been developed for several monogenic diseases. Several have been approved by the US Food and Drug Administration, and there are at least 300 ongoing clinical trials.^11^ The monogenic nature of *SURF1*-related Leigh syndrome supports the possibility of a gene replacement therapy. In 2021, our group reported the development of such a therapy delivered by AAV serotype 9 (AAV9) for *SURF1*-related Leigh syndrome, in which a codon-optimized human *SURF1* (h*SURF1*opt) gene was expressed (AAV9/h*SURF1*v1) to restore the function of SURF1.^12^ We showed that our approach was safe and effective in mice, in which we partially restored complex IV activity and corrected exercise-induced lactate acidosis, without signs of toxicity in wild-type (WT) mice.^12^ These data prompted the initiation of a Good Laboratory Practice (GLP) toxicology study in WT rats.

We first present here the GLP toxicology study findings, which generated significant concerns and prompted a revised vector design. We then report an equally effective but safer gene therapy design that represents a promising therapeutic candidate for *SURF1*-related Leigh syndrome.

## Results

### AAV9/h*SURF1*v1-induced cytotoxicity in WT rats

**Figure 1:**
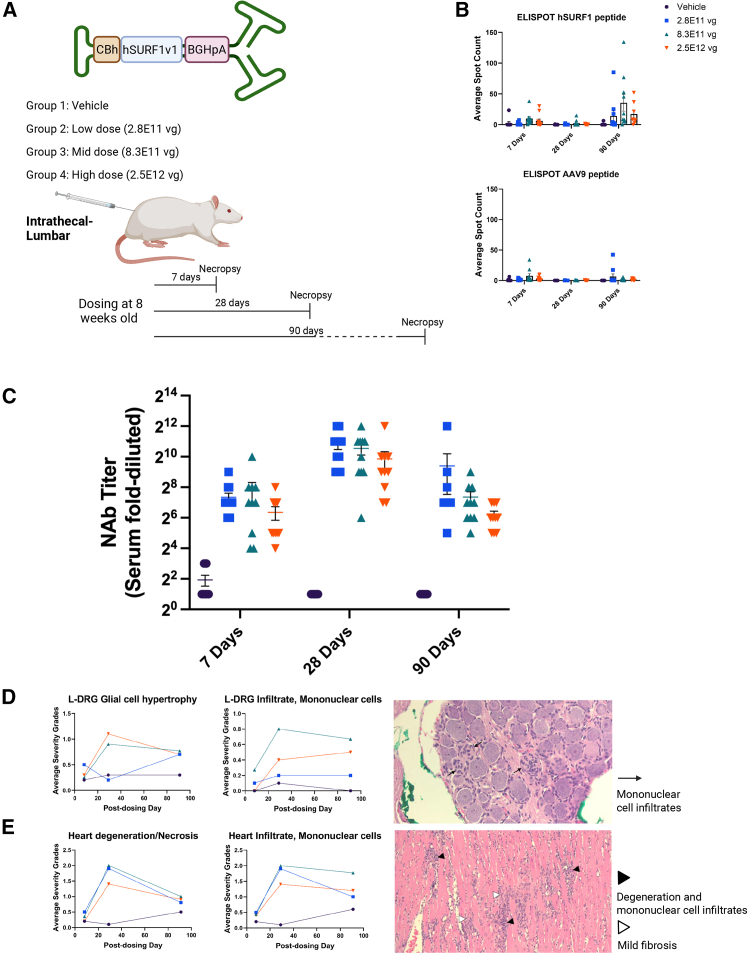
AAV9/h*SURF1*v1 showed cytotoxicity in the DRG and myocardium despite minimum immune responses (A) Study design of GLP toxicology study in WT rats. (B) Average spot count from ELISpot assay detecting innate immune responses to hSURF1 peptide and AAV9 peptide. (C) NAb titer in the serum of the treated rats. (D) Average severity grades of glial cell hypertrophy and mononuclear cell infiltrate in lumbar DRGs (L-DRG). (E) Average severity grades of degeneration/necrosis and mononuclear cell infiltrates in the heart.

We conducted a GLP toxicology study in WT rats, carried out as a contracted study by Charles River Laboratories. In this study, adult WT rats received either vehicle or AAV9/h*SURF1*v1, at doses of 2.8E11 vg, 8.3E11 vg, or 2.49E12 vg per rat through a single lumbar-intrathecal (IT-LP) injection. Separate groups of animals were dosed and sacrificed for tissue collection at 7 days, 28 days, or 90 days post dosing, as shown in Figure 1A. We first examined if there were antigen-related immune responses that may affect transgene expression or induce inflammation. Transduction by AAV9/h*SURF1*v1 presents two foreign antigens to the immune system of WT rats: the AAV9 capsid and WT human SURF1. To screen for cytotoxic T cell responses to these antigens, we used an interferon-gamma (IFN-γ)-based enzyme-linked immunospot (ELISpot) assay for IFN-γ secretion after stimulation of splenocytes collected from the treated rats with overlapping peptide pools spanning the AAV9 VP1 capsid protein or WT hSURF1. As shown in Figure 1B, minimal amounts of IFN-γ secretion were detected in response to the hSURF1 peptide pools at 90 days after treatment, and no IFN-γ secretion was detected in response to AAV9 peptide pools. As expected, all vector treatments elicited the production of neutralizing antibodies (NAbs) against AAV9, as shown in Figure 1C. Histopathological evaluations were carried out in a blinded fashion on various tissues using hematoxylin and eosin (H&E) staining. Signs of cytotoxicities were identified in spinal cord, dorsal root ganglia (DRG), the tibial and sciatic nerves, and heart and liver tissues at all doses tested. Among them, the lumbar DRG and heart showed the most severe changes. Figure 1D summarizes the average severity of glial cell hypertrophy and mononuclear cell infiltrates in the lumbar DRG at three time points and a representative H&E staining image. Similarly, in Figure 1E, we summarize the average severity of necrosis and mononuclear cell infiltrates in the cardiomyocytes at three time points, and a representative image of H&E staining. A detailed pathology report is attached in the supplemental information. These findings were surprising, since we did not identify any of these events in our toxicity study in WT mice. To confirm our previous findings in mice, we conducted another safety study by treating WT mice at 4 weeks old administered with 8E11 vg/mouse of AAV9/h*SURF1*v1 through IT-LP and examined the tissues at 28 days post dosing. Again, no signs of treatment-related cytotoxicity were identified using H&E staining (data not shown). Our findings indicate that the treatment AAV9/h*SURF1*v1 is indeed well tolerated in mice, but cytotoxicity occurred in rats.

### Re-engineer AAV9/h*SURF1* to reduce cytotoxicity

To explore the potential cause of the cytotoxicity shown in the treated rats, we conducted biodistribution analysis for both vector genomic DNA (gDNA) and mRNA of h*SURF1* on the tissues collected from the rats (Figure S1). Compared with 7 days post dosing, at 30 and 90 days post dosing the level of gDNA was significantly diminished (Figure S1). For hSURF1opt mRNA, the highest expression level was observed at 30 days post dosing, which then declined by 90 days (Figure S2). Liver and lumbar DRG showed high gDNA presence and mRNA expression, while the heart (smooth muscle) and bicep (skeletal muscle) showed high mRNA expression despite the reduced presence of vector gDNA. This suggests that the high mRNA expression in the liver and lumbar DRG was driven by high vector particle transduction, while the high mRNA expression in heart and bicep was from high transcription driven by the CBh promoter. The reduced gDNA presence in all tissues across time, coinciding with the rise in transgene expression, suggests a loss of transduced cells that is likely due to hSURF1opt overexpression. Since the toxicity was seen across all three doses, we concluded that it could not be mitigated simply by lowering the vector dose. Rather, it seemed that the CBh promoter-driven expression was producing too much SURF1 protein, particularly in the heart.

**Figure 2:**
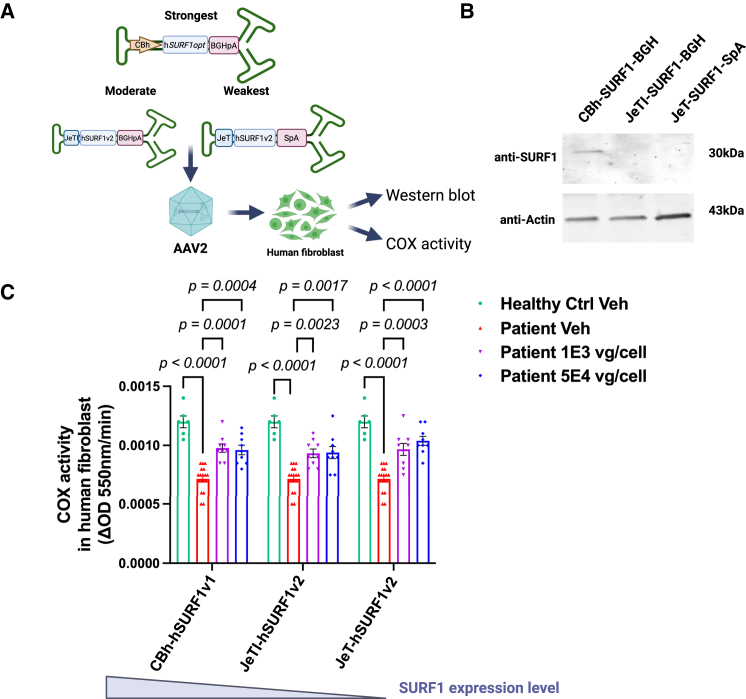
Lower SURF1 transgene expression exerted similar improvement in COX activity in patient fibroblast (A) The designs of newly engineered gene therapy constructs, and the experiment design for (B and C). (B) Western blot results from healthy human fibroblast transduced with three constructs in (A) packaged into AAV2. (C) COX activity results from healthy human fibroblast and patient fibroblast transduced with three constructs in (A) packaged into AAV2. Each data point represents measurement from an individual culture flask, with bars representing the mean ± SEM (standard error of the mean). *p* values were calculated by comparing with the patient vehicle group using Tukey’s multiple comparisons method following ordinary two-way ANOVA.

Therefore, to address the toxicity issue, a reasonable step was to reengineer the gene therapy construct to fine-tune the expression of the h*SURF1* transgene. As shown in Figure 2A, alternative promoters and polyadenylation signals were used to adjust the transgene expression level of two such constructs, one containing stronger elements than the other. The JeT promoter is a synthetic promoter built as a chimeric promoter that merged components of the viral SV40 early promoter, the human beta-actin promoter, and the ubiquitin C promoter.^13^ The JeTI, or UsP, promoter was built by adding an intron sequence between the JeT promoter and the transgene start codon. Previous studies have shown that both JeT and JeTI are weaker promoters than CBh, while JeTI is slightly stronger than JeT.^14^^,^^15^ Additionally, we designed the weakest construct with the JeT promoter and a synthetic poly(A) (SpA)^16^ to drive the lowest expression of h*SURF1*. These two new constructs are referred to as JeTI-h*SURF1*v2 (moderate) and JeT-h*SURF1*v2 (weak), respectively. Additionally, the h*SURF1* transgene was re-codon-optimized to differentiate it from the previous construct as well as to reduce CpG dinucleotides.^17^

We packaged these constructs into an AAV2 capsid and tested these AAV2/h*SURF1* vectors on patient fibroblasts to evaluate their *in vitro* efficacy in restoring the COX activity. First, we examined if the new constructs show lower SURF1 expression compared with CBh-h*SURF1*v1 using a Western blot. As shown in Figure 2B, cells transduced with AAV2/CBh-h*SURF1*v1-BGH showed a clearly visible SURF1 band, while the newer (weaker) constructs did not show any SURF1 protein expression within the sensitivity of this assay. This indicated that the h*SURF1*v2 designs expressed SURF1 lower than CBh-h*SURF1*v1, but the assay limitations did not allow us to quantify a fold change in the expression. Interestingly, COX activity was restored to a similar level with the original and newer designs, despite the differences in multiplicity of infection (MOI) and transgene expression strength of the construct (Figure 2C). This result suggested that a lower amount of SURF1 expression was sufficient to normalize COX activity, and there was no added benefit to overexpressing SURF1. Therefore, to minimize potential cytotoxicity, we initially selected h*SURF1*v2-W as a lead candidate for *in vivo* evaluations.

### AAV9/h*SURF1*v2-W was too weak to show biochemical improvements in *Surf1* knockout mice

**Figure 3:**
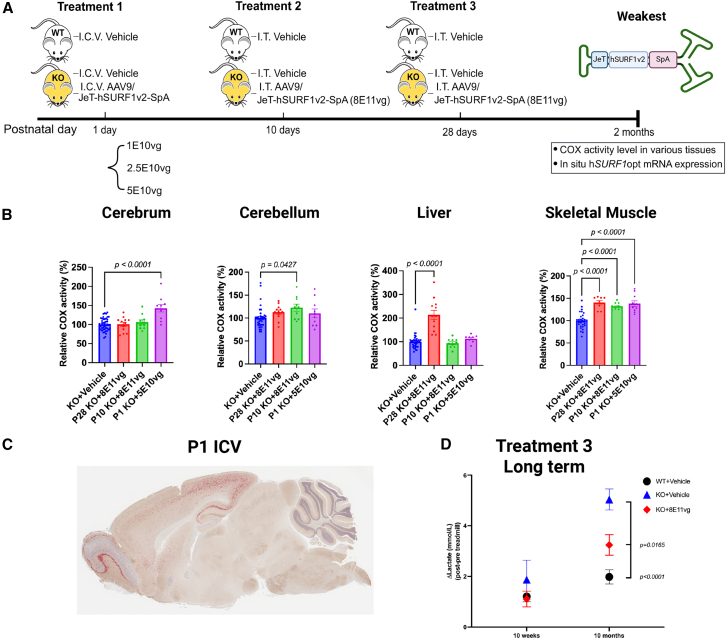
AAV9/JeT-h*SURF1*v2 did not improve COX activity in *Surf1* KO mice dosed at PND10 or PND28 (A) Experiment design with three treatment age, two administration routes, and three doses at PND1. (B) COX activity of KO animals treated with vehicle and AAV9/JeT-hSURF1v2. All data were normalized to the average of KO + vehicle mice. Each data point represents measurement from an individual animal, with bars representing the mean ± SEM. *p* values were calculated by comparing with KO + vehicle mice using Tukey’s multiple comparisons method following ordinary one-way ANOVA. (C) A representative image of brain stained with RNAscope. Red dots represent positive hSURF1 mRNA expression. (D) Δlactate level of mice treated at PND28 and examined at 10 weeks and 10 months of age. Each data point with error bar represents the mean ± SEM of each experimental group. *p* values were calculated by comparing with KO + vehicle mice using a Tukey’s multiple comparison test following two-way ANOVA. *n* = 17–20 per group.

To evaluate the potential of JeT-h*SURF1*v2 as a gene therapy, we intended to use a similar approach as our previous study by packaging the construct into self-complementary AAV9 and evaluating the therapeutic efficacy in the *Surf1* knockout (KO) mice. To achieve a more comprehensive and accurate evaluation, we designed different treatment paradigms as described in Figure 3A. Briefly, IT-LP administration was conducted for both postnatal day (PND)10 and PND28 mice to compare the efficacy, with the hypothesis that earlier treatment may show improved efficacy due to higher numbers of cells transduced.^18^ Additionally, intracerebral ventricular (ICV) injection was conducted with three different doses at PND1, as a proof-of-concept evaluation to examine the degree of the COX activity that can be improved in the brain with the maximum possible number of brain cells transduced. All animals were sacrificed at 2 months of age for biochemical evaluations. The comparisons between the WT + vehicle group and the KO + vehicle group of each tissue are shown in Figure S3A. As shown in Figure 3B, the cerebrum showed significantly increased COX activity in the PND1 treated animals (*p* < 0.0001 compared with the KO + vehicle group, combined from P28-, P10-, and P1-treated animals). Only data from the highest dose are shown in Figure 3, as all three doses showed similar improvements compared with the KO + vehicle group, but only the 5E10 vg/mouse treatment reached statistical significance on its own (*p* = 0.0249) (Figure S3B). In the cerebellum, only PND10-treated animals showed significantly improved COX activity (*p* = 0.0427 compared with the KO + vehicle group), and only PND28 treated animals showed improved COX activity in the liver (*p* < 0.0001 compared with the KO + vehicle group). The skeletal muscle exhibited improved COX activity in all treated animals (*p* < 0.0001 compared with the KO + vehicle group). Figure 3C shows a representative brain slice from a PND1-treated mouse stained for h*SURF1*v2 mRNA using the RNAscope-based *in situ* hybridization, which demonstrates that the expression was concentrated in the forebrain regions. This biodistribution pattern explains the differences in COX activity exhibited in cerebrum versus cerebellum from PND1-treated animals, as shown in Figure 3B.

Additionally, we evaluated the long-term efficacy for animals treated at PND28 by examining the lactate acidosis upon an endurance capacity test. As shown in Figure 3D, the difference between blood lactate level before and after the endurance capacity test (Δlactate) was significantly increased when the animals were tested at 10 months old, and AAV9/JeT-h*SURF1*v2 partially reduced the lactate acidosis (*p* < 0.0001 compared with the KO + vehicle group). It is noteworthy that the original CBh-h*SURF1*v1 vector design completely normalized the lactate acidosis at 10 months of age in this mouse model.^12^

### IT delivery of AAV9/h*SURF1*v2-M improved COX activity deficiency to a similar level exerted by AAV9/h*SURF1*v1

**Figure 4:**
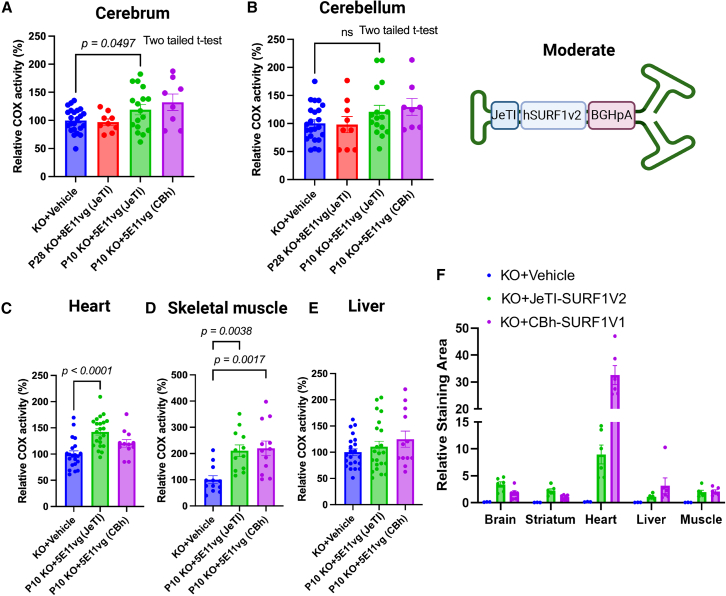
AAV9/JeTI-h*SURF1*v2 improved COX activity in *Surf1* KO mice dosed at PND10 (A–E) Relative COX activity in cerebrum (A), cerebellum (B), liver (C), muscle (D), and heart (E). All data were normalized to the average of the KO + vehicle group. (F) Relative staining area analyzed from RNAscope to examine h*SURF1* mRNA expression. Each data point represents measurement from an individual animal, with bars representing the mean ± SEM. *p* values were calculated by comparing with KO + vehicle mice using Tukey’s multiple comparisons method following ordinary one-way ANOVA for liver, heart, and skeletal muscle. Two-tailed Student’s t test was used for cerebrum and cerebellum.

Since Leigh syndrome is a neurodegenerative disease, it is critical that gene therapy corrects neurological dysfunction. However, AAV9/JeT-h*SURF1*v2 did not show improvement in COX activity in the cerebrum with what we would view as a translatable therapeutic approach, namely, the animals that were treated at PND10 or PND28. These data suggested that the protein expression level in each transduced cell was not sufficient to normalize COX activity levels, and we needed to increase SURF1 protein expression. Therefore, AAV9/JeTI-h*SURF1*v2 was tested through IT-LP administration at PND10 and PND28. To directly compare this design with AAV9/h*SURF1*v1, we also administered AAV9/h*SURF1*v1 side by side with AAV9/JeTI-h*SURF1*v2 at PND10. In all experiment groups, the KO + vehicle group showed between 50% and 40% reduction in COX activity compared with WT mice, as shown in Figure S4. To compare the therapeutic efficacy from both treatment ages and both vectors, we normalized the COX activity of all groups to the average of the KO + vehicle group. As shown in Figure 4A, AAV9/JeTI-h*SURF1*v2 significantly increased cerebrum COX activity compared with the KO + vehicle group (*p* = 0.0497) of the mice treated at PND10 but not at PND28. In the cerebellum, AAV9/JeTI-h*SURF1*v2 increased COX activity by 21% with treatment at PND10 (Figure 4B). AAV9/JeTI-h*SURF1*v2 did not show significant differences compared with AAV9/CBh-h*SURF1*v1 in any of the tissues tested (Figures 4A–4E). In heart tissue, AAV9/JeTI-h*SURF1*v2 showed a trend of higher COX activity than AAV9/CBh-h*SURF1*v1-treated mice. AAV9/JeTI-h*SURF1*v2 induced significantly higher COX activity than vehicle-treated animals (*p* < 0.0001), while the AAV9/h*SURF1*v1 group did not show statistical significance (Figure 4C). Additionally, by using RNAscope, we examined the mRNA expression level of h*SURF1* in the mice treated at PND10. As shown in Figures 4F and S6, AAV9/JeTI-h*SURF1*v2 induced a similar pattern of expression in the brain, liver, and muscle, as compared with AAV9/CBh-h*SURF1*v1. We specifically examined the striatum in the brain (as it is part of the basal ganglia, which is significantly affected in Leigh syndrome), and the expression levels induced by the two vectors were not significantly different. However, AAV9/CBh-h*SURF1*v1 did show significantly greater expression than AAV9/JeTI-h*SURF1*v2 in the heart, which was the most problematic tissue for overexpression-related cytotoxicity for AAV9/CBh-h*SURF1*v1 in rats. We also examined different doses (1.25E11 vg, 2.5E11 vg, and 5E11 vg) of AAV9/JeTI-h*SURF1*v2 to see if a higher dose is required to show therapeutic efficacy. Our data suggested that, for the mice treated at PND10, when lower doses are administered (2.5E11vg and 1E11vg), the improvement in COX activity is diminished (Figure S5). Figure S6 shows representative RNAscope images of target tissue from mice treated with low (1.25E11 vg), mid (2.5E11 vg), or high (5E11 vg) doses of AAV9/SURF1. Red spots show increased *SURF1opt* transgene mRNA expression in a dose-dependent manner across all target tissues. Taken together, a high dose and early treatment of AAV9/JeTI-h*SURF1*v2 through IT-LP effectively improved COX activity in both the CNS and in peripheral tissues by *SURF1* gene replacement.

### Improvements related to endurance capacity and lactate acidosis

**Figure 5:**
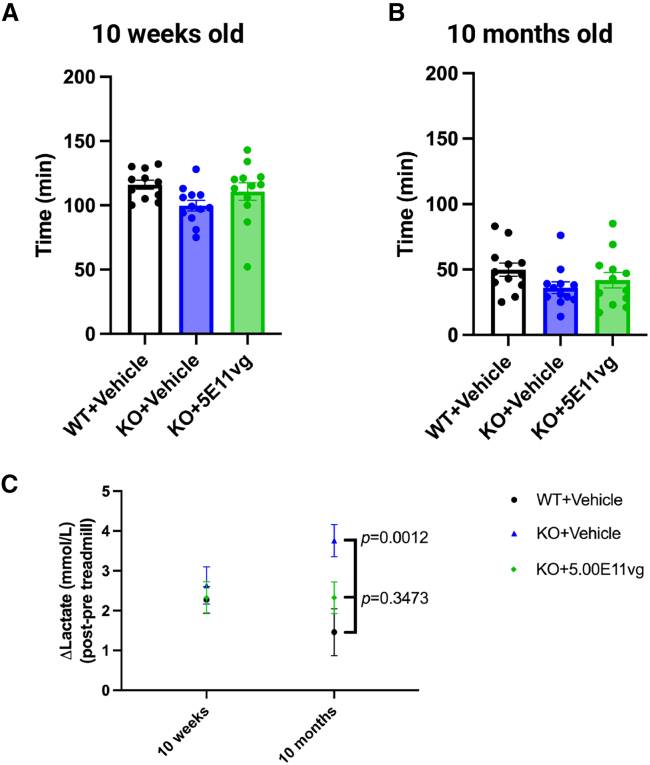
AAV9/JeTI-h*SURF1*v2 partially restored endurance capacity and exercise-induced lactate acidosis in *Surf1* KO mice dosed at PND10 Endurance capacity tested at 10 weeks of age (A) and 10 months of age (B). Each data point represents measurement from an individual animal, with bars representing the mean ± SEM. (C) Δlactate level of mice treated at PND28, and examined at 10 weeks and 10 months of age. Each data point with error bar represents the mean ± SEM of each experimental group. *p* values were calculated by comparing with KO + vehicle mice using a Tukey’s multiple comparison test following two-way ANOVA. *n* = 11–12 per group.

Leigh syndrome patients with *SURF1* deficiency often show elevated blood lactate levels. Previous studies reported that *Surf1* KO mice also exhibited elevated blood lactate levels, especially after exhaustive exercise.^12^ Thus, to evaluate whether gene therapy can correct the blood lactate level, we treated another group of mice with the same high dose (5 × 10^11^ vg/mouse) of AAV9/JeTI-h*SURF1*v2 through IT administration at PND10 and examined blood lactate levels both at rest and after exhaustive exercise. The mice were tested for endurance capacity at two ages, 10 weeks (9 weeks after treatment) and 10 months (10 months after treatment). Blood lactate was examined both before and after running on a treadmill until exhaustion, and the change in blood lactate level was recorded as Δlactate. As shown in Figures 5A and 5B, the KO + vehicle group showed a trend of reduced running time to exhaustion at both ages (comparing WT + vehicle and KO + vehicle groups, mean difference = 16.34 min at 10 weeks old, and mean difference = 13.75 min at 10 months old), and the treatment showed partial improvement (comparing KO + 5E11 vg and KO + vehicle groups, mean difference = 11.00 min at 10 weeks old, and mean difference = 5.75 min at 10 months old). Additionally, as shown in Figure 5C, the Δlactate of *Surf1* KO mice was significantly higher than that of WT animals when tested at 10 months of age (*p* = 0.0012). AAV9/JeTI-h*SURF1*v2 treatment reduced the Δlactate of KO mice closer to the level of WT animals, and the differences between the two groups were not statistically significant (*p* = 0.3473 comparing KO + 5E11 vg and WT groups; *p* = 0.0609 comparing KO + 5E11 vg and KO + vehicle groups). Taken together, our data suggest that IT-administered AAV9/JeTI-h*SURF1*v2 restored endurance capacity and mitigated abnormal lactic acidosis during exhaustive exercise in *Surf1* KO mice.

### IT-LP delivery of AAV9/h*SURF1*v2*-M* is safe in WT rats

**Figure 6:**
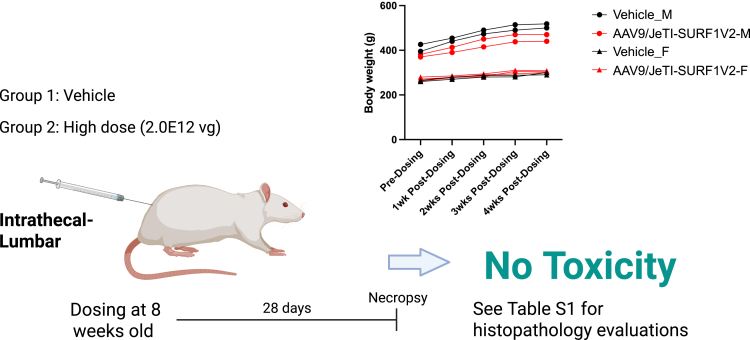
AAV9/JeTI-h*SURF1*v2 did not show toxicity in WT rats Experiment design and the overall body weight changes of four vehicle-treated rats and five AAV9/JeTI-h*SURF1*v2-treated rats.

We evaluated the safety of AAV9/JeTI-h*SURF1*v2 in WT rats by treating 6-week-old rats with 2E12 vg/rat through IT-LP. First, we examined overall growth by tracking body weight changes. As shown in Figure 6, there was no difference between vehicle and AAV9/JeTI-h*SURF1*v2 treated rats. Tissue was collected 4 weeks post dosing, and we performed histopathological evaluations using H&E staining. Treatment with AAV9/JeTI-h*SURF1*v2 did not lead to any adverse histological findings (Table S1). Thus, we conclude that this newer design has acceptable safety in rats, and more specifically that the lower expression mediated by the JeTI-h*SURF1*v2 design overcame the overexpression-related toxicities caused by the original CBh-h*SURF1*v1 design.

## Discussion

In this study, we re-engineered a previously developed gene therapy for *SURF1*-related Leigh syndrome, and demonstrated that the new design is equally effective with minimal toxicity. As we pointed out in our previous publication,^12^ one limitation of developing a gene therapy for *SURF1*-related Leigh syndrome is that the available mouse model does not recapitulate overt phenotypes of the human disease. Thus, the types of outcome readouts for evaluating the efficacy of a gene therapy become limited. The only readouts we have so far are COX activity, endurance capacity, and lactate acidosis in the context of the endurance capacity test at 10 months of age. For COX activity assay, bulk tissues were homogenized for mitochondria isolation. However, since not all cells are transduced by AAV9 to an equal extent, and SURF1 is a non-secreted protein, the results of the assay encompass cells of different *SURF1* transgene expression levels, resulting in different degrees of restoration in COX activity. Consequently, the COX activity values we have presented are an average of all cells (including non-transduced cells), some of which show better restoration than others. Based on the biodistribution data of self-complementary AAV9 from previous publications^14^^,^^19^ and the data we acquired from WT rats, the peripheral organs that are affected in the disease, including heart, liver, and skeletal muscle, have been well transduced. The normalization of endurance capacity and lactic acidosis at 10 months of age further supported the gene therapy efficacy in the skeletal muscle. However, the current animal model makes it difficult to determine whether the number of cells we are targeting with the current therapy is enough to provide any neurological benefits. A recent publication provided promising indications that transfecting less than 30% cells lead to robust phenotypic restoration in two mitochondrial encephalopathy models, suggesting that the increases in COX activity achieved in our studies is sufficient to predict a meaningful therapeutic benefit.^20^

We also analyzed the protein expression profile of the cerebrum of PND10-treated animals, where tissues were collected at PND56. The proteomic data provided further complementary evidence on the restoration of the normal cell state with the gene therapy treatment. COA3 has been suggested as an assembly factor for the COX1 subunit of the COX holoenzyme.^21^ NDUFB9, NDUFA1, and NDUFB8 are subunits of complex I, while UQCR10 is a subunit of complex III. CYB5B is a protein that enables heme binding activity. The expression of each was significantly increased in *Surf1* KO mice, possibly due to a negative feedback mechanism, and these increases were normalized to the level of WT mice with AAV9/JeTI-h*SURF1*v2 treatment (Figure S7). However, *Mtco1*- and *Mtco2*-encoded proteins, two core subunits of COX, showed reduced expression in *Surf1* KO mice, but the gene therapy treatment was only able to exert a slight increase of these proteins (6.0% for Mtco1 and 17.1% for Mtco2; not statistically significant). These data complement and reinforce the COX activity data, indicating that gene therapy treatment was able to partially restore protein expression homeostasis in the cerebrum of *Surf1* KO mice. Raw data used to plot Figure S7 are provided in Supplemental item 3.

Our study presented an example where the efficacy of gene therapy is dictated by both vector transduction and the transgene expression regulated by the transgene cassette. They work hand-in-hand exhibiting a bell-curved relationship, where too little transduction or gene expression leads to suboptimal therapeutic efficacy, while too much transduction and transgene overexpression may lead to cytotoxicity. Mitochondria transfer between neighboring cells occurs as a cellular repair mechanism, instead of a physiological process of constant mitochondria exchange between cells.^22^ Therefore, we expect gene therapy for mitochondrial diseases to be cellular autonomous, meaning only cells that are transduced with the vector are treated. This explains why we are not able to fully restore COX assembly and activity with the current vector when bulk tissue samples are analyzed. Therefore, improvements on increasing gene transfer to the CNS, such as using AAV capsids with enhanced CNS transduction, may greatly enhance therapeutic efficacy.

However, for tissues where vector transduction efficiency is high, the gene expression level can be modulated by the transgene cassette design, and a high expression vector can lead to cytotoxicity. This conclusion was supported by the GLP toxicology study with the original AAV9/CBh-h*SURF1*v1, in which high h*SURF1* mRNA expression led to cell degeneration and mononuclear cell infiltrates in multiple tissues, especially the heart and lumbar DRG. AAV9 delivered through IT-LP has been shown to cause similar DRG-related cytotoxicities in several other studies.^23^^,^^24^^,^^25^ However, the cytotoxicities in the heart were particularly concerning, warranting further investigation. In our studies of *Surf1* KO mice, we observed that COX activity of the heart trended lower in AAV9/CBh-h*SURF*1-v1-treated animals compared with those treated with AAV9/JeTI-h*SURF1*v2. Accordingly, as shown in Figure 4F, mRNA of hSURF1 was approximately 3-fold higher in the heart of AAV9/CBh-h*SURF*1v1-treated animals than AAV9/JeTI-h*SURF1*v2-treated animals. Both studies suggested that the CBh promoter preferentially drives strong expression in cardiomyocytes, and this transgene overexpression presumably creates mitochondrial stress, leading to cytotoxicity. However, this is not the first mitochondrial disease that raised this issue. Studies in gene therapy for Friedreich’s ataxia also showed toxicity in the mouse model. Overexpression of frataxin caused accumulation of iron-sulfate clusters.^26^ There have been several theories on mitochondrial protein overexpression-related stresses, such as mitoUPR^27^ and mitoPOC.^28^ Even though we do not know the exact mechanisms driving SURF1 overexpression toxicity or the toxicity related to other mitochondrial proteins, these observations warrant caution that extra care may be required with the design of mitochondrial disease gene therapies.

## Materials and methods

### Animals

The *Surf1* KO mice were initially generated from Dr. Massimo Zeviani’s Lab, and we acquired them as a gift from Dr. Holly Van Remmen’s Lab at the Oklahoma Medical Research Foundation. We then backcrossed the mice we acquired with WT C57BL/6J mice for 10 generations. Thus, the *Surf1* KO mice in this study are on a C57BL/6J genetic background, which is different from our previous study.^12^ The genotypes were determined by PCR analysis using a mouse ear punch as previously described.^29^ The WT C57BL/6J mice were purchased from the Jackson Laboratories (Bar Harbor, ME, USA). The WT Sprague-Dawley rats were purchased from Charles River Laboratory (Wilmington, MA, USA). The animal studies at University of Texas Southwestern (UTSW) were conducted according to protocols approved by the Institutional Animal Care and Use Committee (IACUC). All mice and rats were weaned between PND21 and PND28. All mice and rats were provided food and water *ad libitum*.

### Plasmids

The complete name for h*SURF1*v1 is self-complementary CBh-codon-optimized human *SURF1*-BGHpA. It is composed of a CBh promoter, a h*SURF1*opt cDNA sequence (ATUM, Newark, CA, USA) and a BGH poly(A) tail, as described.^12^ This construct with a GFP reporter in place of h*SURF1* has been described in multiple publications from our group across different animal models.^30^^,^^31^^,^^32^^,^^33^^,^^34^ The 5′-inverted terminal repeat (ITR) is mutated to form the self-complementary structure as previously described.^35^

For JeT-h*SURF1*v2 and JeTI-hSURF1v2, we replaced the CBh promoter with the JeT or JeTI promoter, respectively. The JeTI promoter is the JeT promoter with an added intron and is also referred to as the UsP promoter.^36^^,^^37^ Additionally, the human *SURF1* cDNA sequence was codon-optimized again (ATUM) to reduce CpG dinucleotides. For JeT-h*SURF1*v2, the poly(A) was replaced with an SpA sequence.

### Virus production

AAV9/JeTI-h*SURF1*v2 and all AAV2 vectors were produced at the University of North Carolina (UNC)-Chapel Hill Vector Core as described.^38^ Purified vectors were dialyzed in PBS (350 mM final NaCl concentration) containing 5% D-sorbitol and stored at −80°C until use. Thawed aliquots were subsequently stored at 4°C. A filter-sterilized solution of PBS (350 mM final NaCl concentration) containing 5% D-sorbitol was used as vehicle and virus dilution buffer. The viral vector was titered by qPCR and confirmed by PAGE and silver stain by the UNC Vector Core.^39^ The vectors in this study were packaged in self-complementary AAV9. This preparation was re-titered by the UTSW Translational Gene Therapy Core (TGTC), to ensure that comparable doses were used across all studies.

AAV9/JeT-h*SURF1*v2 and AAV9/CBh-hSURF1v1 were produced by the UTSW TGTC and formulated in PBS containing 5% d-sorbitol and 0.001% pluronic F-68. It was produced by triple transfection of suspension-cultured HEK293 cells, followed by cell lysis and recovery of recombinant AAV virus from the cells and media. Purification was through filtration, affinity chromatography, and anion exchange chromatography using methods developed at the TGTC. The vector was titered by quantitative PCR directed to the ITR.

### Mouse and rat IT-LP injections

AAV9 vectors were diluted in vehicle solution. The mouse IT-LP injections were conducted through lumbar puncture as previously described, with volumes ranging from 7.3 μL to 16.8 μL.^40^ For ICV injection, the mouse pup was cryo-anesthetized using a barrier to prevent direct contact with the cold source and to keep the pup dry. Lack of response to toe pinch was utilized to ensure the pup reached the correct anesthetic depth. The pup was placed on chilled modeling clay to hold the pup in place and prolong anesthesia during injection. A 33G needle with needle stop attached to a Hamilton syringe was inserted to a depth of 2 mm and used to deliver 1.25 μL of AAV per hemisphere at a rate of 1–2 μL/min into the lateral ventricle. The needle was removed 15 s after discontinuation of plunger movement to prevent backflow. Mice were then placed on top of a warming pad and returned to the home cage with the dam after regaining full activity typical of newborn mice. For IT-LP injections in rats, each rat was induced and maintained under isoflurane gas anesthesia for the duration of the injection procedure. The dorsal lumbar region was shaved, and the skin was prepped using 70% isopropyl alcohol, 2% chlorhexidine solution, and povidone-iodine. A 1-cm incision in the skin was made over the L5–L6 junction. A 1-inch, 27G needle attached to a 100-μL Hamilton syringe was inserted between the vertebrae and into the intrathecal space. The vehicle or the vector solution was delivered at a slow, continuous rate in a volume of 44 μL. The needle was removed 30 s after discontinuation of the plunger movement to prevent backflow. The skin incision was closed with suture. Rats were recovered from anesthesia in a warmed recovery chamber until normal ambulation was regained, then returned to the home cage. Rats were monitored and provided pain medication postoperatively in accordance with institutional and IACUC regulations.

### Safety study in WT CD rats in a GLP study

This animal study was performed by Charles River Laboratories according to GLP, with immunology and biodistribution studies carried by Dr. Gray’s laboratory as GLP exceptions. Male and female Sprague-Dawley rats were randomized into cohorts, with five males and five females per cohort, and dosed as shown in Figure 1A. At the initiation of dosing, the animals assigned to the study were approximately 56–63 days old and weighed between 165 g and 328 g. The AAV9/h*SURF1*v1 vector was injected IT-LP once in each animal by a qualified laboratory technician, in a volume of 20 or 60 μL at a final dose of 2.8E11, 8.3E11, or 2.5E12 vg/rat. All animals were monitored up to 90 days following the injection. Rats were sacrificed on day 7, 28, or 90 after injection, and tissues were collected for biodistribution and histopathology evaluation. For biodistribution, both mRNA and total gDNA were purified from tissue samples collected at necropsy day 28, using a QIAGEN QIAcube HT (Hilden, Germany). mRNA was then reverse-transcribed into cDNA using QIAGEN RT2 HT first strand kit. qPCR was used to determine the quantity of the h*SURF1* transgene per diploid rat genome (DNA analysis) or copies of h*SURF1* per copy rat GAPDH (mRNA analysis). Details of this study are provided in Charles River Laboratories’ final report, provided as supplemental information.

### ELISpot analysis

Splenocytes were isolated at Charles River Laboratory from treated WT rats, and stored in freezing medium (90% fetal bovine serum [FBS], 10% DMSO), in the vapor phase of liquid nitrogen until the ELISpot assay.

For preparation of the peptide pools, peptides were ordered from Mimotopes (Victoria, Australia). The AAV9 capsid pools were both AAV9 capsid comprised of 20-mers with a 10-amino acid offset, and hSURF1 peptide pools were composed of 15-mers with a 5-amino acid offset. The AAV9 capsid library pool contained 146 peptides and the final concentration of each peptide in the pool was 0.63 mg/mL in 1.59% DMSO final. The hSURF1 peptide library pool contained 29 peptides and the final concentration of each peptide in the pool was 1.02 mg/mL in 0.61% DMSO final. Both pools were stored at −80°C.

ELISpot assays were performed using an ImmunoSpot kit (mIFNg-1M/5, Cellular Technology Limited, Shaker Heights, OH, USA). Briefly, splenocytes were thawed, washed, and resuspended in cRMPI-1640 medium for counting. We plated 2E5 splenocytes in 100 μL of cRPMI-1640 medium into each well of an ELISpot plate in quintuplicates whenever possible. We then added 100 μL of cRPMI-1640 medium containing AAV9 capsid or hSURF1 peptide pool to the wells. The controls included cells with no peptide, cells stimulated with a mixture of phorbol 12-myristate 13-acetate and ionomycin (Invitrogen, Waltham, MA, USA; 00-4970-93), medium with interferon-γ, which was supplied with the kit, or medium only. The splenocytes were incubated for 48 h in a humidified 37°C CO_2_ incubator. All other steps were performed according to the manufacturer’s recommendation. Spots were read with the same sensitivity settings for all plates.

### NAb assay

AAV9/CBh-EGFP as a reporter vector was incubated with serial dilutions of serum samples from each rat and then added to Lec2 cells seeded at 6.5E3 cells per well of a 96-well plate. ADK9 antibody (Progen, Wayne, PA, USA) was incubated with serial dilution as a positive control for inhibition. AAV9 was added at an MOI of 3.9E9 vg/well. The infection efficiency was estimated 48 h later via EGFP intensity in infected cells measured using a microplate reader. AAV NAb titers were determined as the dilution of the tested serum that resulting in approximately 50% GFP expression relative to the uninhibited control. Cells not infected with AAV were used to establish the baseline (background) fluorescent signal.

### Cell culture and AAV transduction

Human fibroblasts were cultured in complete culture media consisting of 50% DMEM (+4.5% D-glucose, +L-glutamine, -sodium pyruvate), 50% MEM 1-a (1×), 1% penicillin/streptomycin (10,000 U/mL), 15% FBS, and 1% GlutaMax (1×). Cells were maintained in a humidified 5% CO_2_ incubator at 37°C. At 24 h before AAV transduction, cells were seeded into a T150 flask, and AAV vectors were added into the culture 24 h later based on the MOI described in the Results. Cells were collected 48 h after transduction using 0.05% Trypsin-EDTA, and cell pellets were stored at −80°C for COX activity assay.

### COX activity assay for human fibroblast

The COX Assay was performed using the Complex IV/COX Human Enzyme activity microplate assay kit (Abcam, Waltham, MA), following the manufacturer’s instructions. Briefly, depending on the estimated pellet size, each sample pellet was resuspended in 50–100 mL of Solution 1. Protein concentration of each sample was determined using a BCA assay, and the final protein concentration was adjusted to 5 mg/mL for each sample. A mild detergent was added to each sample to a final dilution of 1:10, and each sample was incubated on ice for 30 min, to break the mitochondrial membrane. The samples were then centrifuged for 20 min at 12,000×*g* at 4°C. The supernatant was collected and diluted with Solution 1, and COX is immunocaptured in a pre-coated 96-well plate. COX activity is determined colorimetrically through the oxidation of reduced cytochrome *c* as an absorbance decrease at 550 nm. The absorbance of each sample was measured once every minute for 120 min and the initial rate of decrease was calculated as COX activity.

### Western blot

SURF1 patient fibroblast was seeded at 3.8E4 cells/well in a six-well plate the day before AAV2 transduction. We added AAV2/hSURF1 into the culture wells at 5E4 vg/cell 24 h later. After transduction for 48 h, cells were collected for western blot. Protein was extracted using RIPA buffer (Thermo Fisher Scientific, Waltham, MA, USA), and the total protein concentration was determined using a BCA assay. Equal amounts of protein were separated by gel electrophoresis using 4–20% mini-PROTEAN TGX precast protein gels (Bio-Rad, Hercules, CA, USA). Proteins were transferred to a PVDF membrane, and upon overnight blocking with 5% blocking buffer (Bio-Rad, Hercules, CA, USA), SURF1 protein was detected using SURF1 primary antibody (GENETEX, Irvine, CA, USA) at 1:500 dilution and goat anti-rabbit Dylight 680 secondary antibody (Thermo Fisher Scientific) at 1:1,000 dilution. Membrane was then incubated with actin-Dylight 680 loading control (Thermo Fisher Scientific) at 1:1,000 overnight at 4°C as an internal reference.

### COX activity assay for mouse tissues

Mice were perfused with 1× PBS 4 weeks post treatment. Tissues were extracted quickly and immediately frozen on dry ice. All tissues were stored at −80°C until use. We used Complex IV/COX Rodent Enzyme activity microplate assay kit (Abcam) to examine the COX activity of our mice model, according to the manufacturer’s instructions. Mitochondria were extracted as previous described.^41^^,^^42^ After mitochondria extraction, COX is immunocaptured within the wells and activity is determined colorimetrically through the oxidation of reduced cytochrome *c* as an absorbance decrease at 550 nm. The absorbance of each sample was measured once every minute for 120 min and the initial rate of decrease was calculated as COX activity. For COX activity of 8-week-old mice, assays were performed in multiple batches, in which at least one WT and one KO + vehicle mouse were included. Each data point was then normalized to the average of all WT COX activity samples measured in that set, as relative COX activity. For COX activity of 18-month-old mice, assays were performed in three batches for each tissue. We noticed that there were significant differences among the raw values of WT COX activity of the three batches. Thus, each data point was normalized to the average of WT COX activity within each batch as relative COX activity.

### Exhaustive exercise on treadmill and blood lactate test

Blood lactate following exhaustive exercise was measured by the UTSW Metabolic Phenotyping Core. All mice were familiarized to the treadmills for 2 days prior to the exercise session. On day 1, they experienced a 5-min rest on the treadmill followed by running for 5 min at the speed of 8 m/min and then for 5-min at the speed of 10 m/min. On day 2, they experienced a 5-min rest on the treadmill followed by running for 5 min at the speed of 10 m/min and then for 5 min at the speed of 12 m/min.

On day 3, mice were placed on the treadmill for 5 min at rest, followed by running at a starting speed of 10 m/min for 40 min, next by running at speeds that were increased at the rate of 1 m/min every 10 min until the speed reached 13 m/min, and finally by running at speeds that were increased at the rate of 1 m/min every 5 min until exhaustion. The exhaustion time was noted as the time at which the mice remained on the electric shock grid for 5 continuous seconds, without attempting to resume running. Blood lactate was taken before putting the mice onto the treadmill and immediately when the mice came off the treadmill. A drop of blood was collected from the tail vein and blood lactate concentration was measured using a Lactate Plus lactate meter (Nova Biomedical, Waltham, MA, USA).

### Proteomics

Proteins from isolated mitochondria were extracted with RIPA buffer, and the protein concentration was determined using BCA assay. We used 2.5 μg/μL protein sample of 50 μg total protein for the analysis. Following disulfide bond reduction and alkylation, samples were digested overnight with trypsin using an S-Trap (Protifi, Fairport, NY, USA). The peptide eluate from the S-Trap was dried and reconstituted in 100 mM TEAB buffer. The TMTpro18plex Isobaric Mass Tagging Kit (Thermo Fisher Scientific) was used to label the samples as per the manufacturer’s instructions. The combined sample then underwent solid-phase extraction cleanup with an Oasis HLB plate (Waters, Milford, MA, USA) and was dried in a SpeedVac. The sample was then reconstituted in a 2% acetonitrile, 0.1% TFA buffer and diluted such that approximately 1 μg of peptides was injected. Peptides were analyzed on a Thermo Orbitrap Eclipse mass spectrometry (MS) system coupled to an Ultimate 3000 RSLC-Nano liquid chromatography system. Samples were injected onto a 75 μm inner diameter, 75-cm long EasySpray column (Thermo Fisher Scientific) and eluted with a gradient from 0% to 28% buffer B over 180 min at a flow rate of 250 nL/min. Buffer A contained 2% (v/v) ACN and 0.1% formic acid in water, and buffer B contained 80% (v/v) ACN, 10% (v/v) trifluoroethanol, and 0.1% formic acid in water at a flow rate of 250 nL/min. Spectra were continuously acquired in a data-dependent manner throughout the gradient, acquiring a full scan in the Orbitrap (at 120,000 resolution with a standard AGC target) followed by tandem MS scans on the most abundant ions in 2.5 s in the ion trap (turbo scan type with an intensity threshold of 5,000, collision-induced dissociation collision energy of 35%, standard AGC target, maximum injection time of 35 ms and isolation width of 0.7 m/z). Charge states from 2 to 6 were included. Dynamic exclusion was enabled with a repeat count of 1, an exclusion duration of 25 s, and an exclusion mass width of ±10 ppm. Real-time search was used for selection of peaks for SPS-MS3 analysis, performed against a list of mouse mitochondrial proteins from UniProt along with additional mouse mitochondrial proteins.^43^ Up to two missed tryptic cleavages were allowed, with carbamidomethylation (+57.0215) of cysteine and TMTpro reagent (+304.2071) of lysine and peptide N-termini used as static modifications and oxidation (+15.9949) of methionine used as a variable modification. MS3 data were collected for up to 10, MS2 peaks that matched to fragments from the real-time peptide search identification, in the Orbitrap at a resolution of 50,000, higher-energy collisional dissociation collision energy of 65% and a scan range of 100–500.

Protein identification and quantification were done using Proteome Discoverer v.3.0 SP1 (Thermo Fisher Scientific). Raw MS data files were analyzed against the human reviewed protein database from UniProt. Both Comet and SequestHT with INFERYS Rescoring were used, with carbamidomethylation (+57.0215) of cysteine and TMTpro reagent (+304.2071) of lysine and peptide N-termini used as static modifications and oxidation (+15.9949) of methionine used as a variable modification. Reporter ion intensities were reported, with further normalization performed by using the total intensity in each channel to correct discrepancies in sample amount in each channel. The false discovery rate cutoff was 1% for all peptides.

### *SURF1* RNAscope

*SURF1* RNA was examined *in situ* for brain, heart, liver, and skeletal muscle from a set of treated animals. The following protocol was carried out by a senior histologist in the Gray lab, blinded to the treatments and genotypes of all animals. Tissues ready for staining were processed and embedded in paraffin and cut into 5-μm sections on slides. RNAscope 2.5 HD Assay kit (Advanced Cell Diagnostics, Newark, CA, USA) was used, with a custom probe designed against the h*SURF1*v1 or h*SURF1*v2 sequences. Slides were deparaffinated by xylene and then xylene was removed with 200-proof ethanol. Then slides were incubated with hydrogen peroxide for 10 min at room temperature and washed with distilled water. Antigen retrieval was performed by boiling the slides in 1× Target Retrieval solution for 10 min, washing with distilled water, dehydrated with 200-proof ethanol, and then air dried. Protease Plus was added to each section, incubated at 40°C for 30 min, and washed with distilled water. Then slides were incubated with h*SURF1*opt RNAscope probe in HybEZ oven at 40°C for 2 h and washed with 1× Wash Buffer. Then slides were incubated with AMP 1–6 for 30 or 15 min following the RNAscope 2.5 HD Detection Kit protocol. Then, slides were incubated with RED solution for 10 min to detect the RNAscope signals. Finally, slides were covered with coverslips and imaged with Aperio imagescope.

### Image analysis

Histology images were analyzed using custom analysis settings in HALO Image Analysis Platform (Halo2.2, Indica Labs, Albuquerque, NM). A region of interest (ROI) was hand drawn on each image to allow for analysis by tissue region. For the brain, we analyzed the entire brain, including cerebrum and cerebellum, and performed a separate analysis for striatum. A threshold for each stain was set using positive and negative control images, and the same analysis settings were applied for every image of the same stain. Percent area strongly staining for each marker of interest was recorded for each tissue/ROI.

### Statistical analysis

Underlying assumptions checking for the continuous variables was performed prior to analysis. Specifically, we used the Shapiro-Wilk test for the normality of the data distribution and Brown-Forsythe test for homogeneity of variance. Univariable comparisons were conducted using Student’s t test or one-way ANOVA for normally distributed data with equal variance, and Wilcoxon or Kruskal-Wallis test, as a non-parametric counterpart, for non-normal data. A statistical significance was assumed at the 0.05 level, and multiple comparisons were adjusted using Tukey correction or Dunn’s multiple comparisons test. Blood lactate data were compared with two-way ANOVA with adjustment of multiple comparisons using Tukey’s multiple comparisons test. GraphPad Prism (GraphPad Software, Inc., San Diego, CA, USA) was used for all statistical analysis and generating the graphs.

## Data availability

The materials described in this publication are available upon request to members of the scientific community for noncommercial purposes.

## Acknowledgments

This study was supported by funding from the Cure MITO Foundation, Taysha Gene Therapies, and the RTW Foundation to S.J.G. Q.L.’s postdoctoral training was supported by 10.13039/100000071NICHD K99/R00 Pathway to Independence Award (K99HD109359). We thank Dr. Massimo Zeviani and Dr. Holly Van Remmen for providing the initial *Surf1* KO mice breeders. We thank the UNC Vector Core and UTSW Translational Gene Therapy Core for producing the vectors used in these studies. We thank UT Southwestern Metabolic Phenotyping Core for performing endurance capacity tests, measuring blood lactate, and conducting blood chemistry tests. We acknowledge Dr. Mary Wright-Carter and her team at UTSW Diagnostic Laboratory for conducting the toxicology evaluation on tissues from the safety study and providing the histopathological safety report. Last, we acknowledge Dr. Andrew Lemoff and his team at the UTSW Proteomics Core for performing proteomics examinations for this study. The figures in this manuscript were created in BioRender.

## Author contributions

Q.L. and S.J.G. contributed to study concept and design. Q.L., M.R., H.H., S.E.D., and Y.H. contributed to data acquisition. Q.L. contributed to data analysis. Q.L. wrote the initial manuscript draft and figures; S.J.G. and M.R. edited the manuscript.

## Declaration of interests

Q.L. and S.J.G. are inventors of the SURF1 vector designs used in this study, and they have received royalty income related to these inventions. S.J.G. has received consulting income from Taysha Gene Therapies.

## Supplemental information

Document S1. Figures S1–S7

Table S1. Non-GLP histopathological evaluation findings

Table S2. Analyzed data of proteomics study in Figure S7

Data S1. GLP toxicology study for AAV9CBh-SURF1v1

Document S2. Article plus supplemental information
