# Supplementary material for: Improved AAV9-based gene therapy design for SURF1-related Leigh syndrome with minimal toxicity
Source: Mol Ther Methods Clin Dev. 2025 Aug 11;33(3):101554. doi: 10.1016/j.omtm.2025.101554 (PMC12398787; doi:10.1016/j.omtm.2025.101554)
Supplement: Document S1. Figures S1–S7 [file mmc1.pdf]

OMTM, Volume *33*

Supplemental information

Improved AAV9-based gene therapy design for *SURF1*-related Leigh syndrome with minimal toxicity

Qinglan Ling, Matthew Rioux, Harrison Higgs, Yuhui Hu, Scarlett E. Dwyer, and Steven J. Gray

Supplemental Information

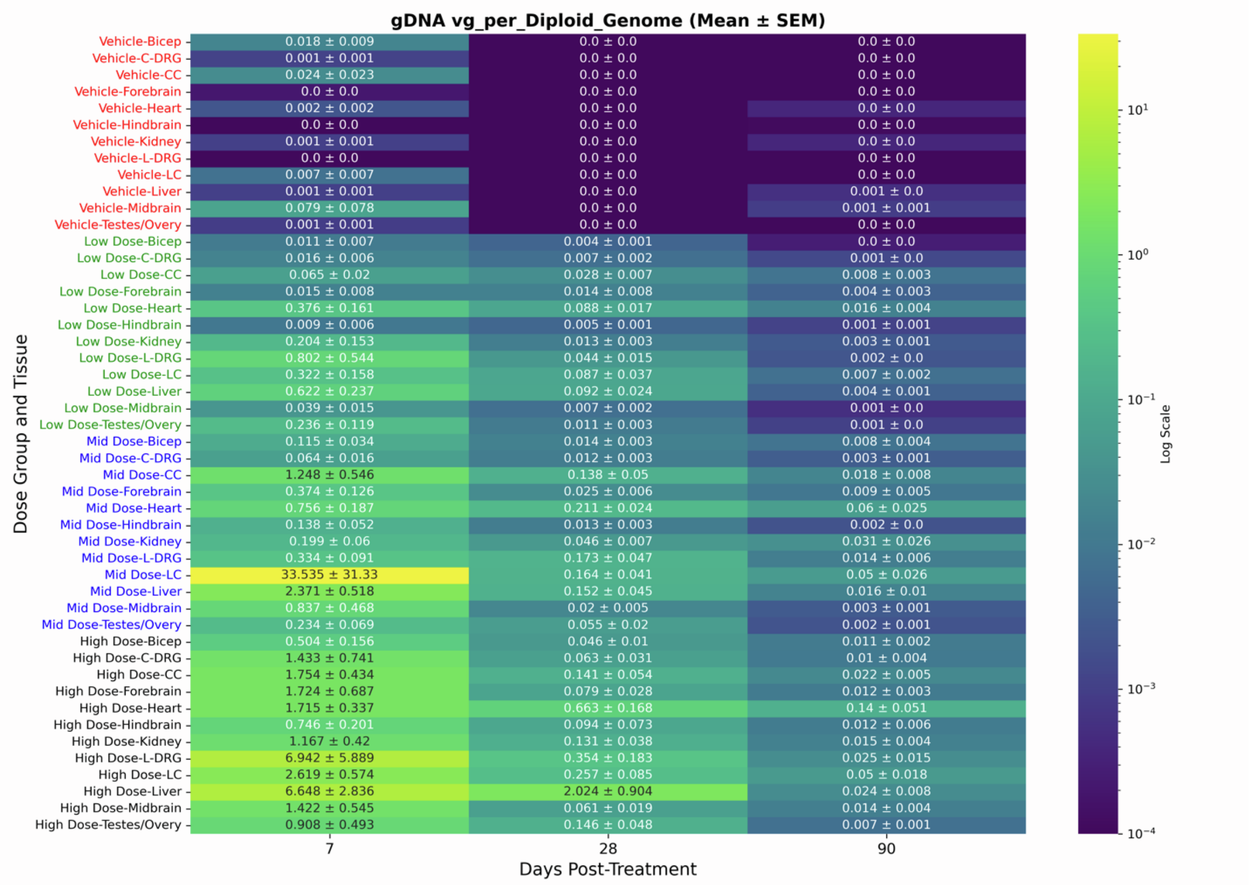

Figure S1. gDNA biodistribution of h*SURF1* normalized to diploid genome copy number. Each data point represents each treatment group, and the number indicates Mean ± SEM (standard error of the mean). Purple shows lower expression and yellow shows higher expression. Color bar is set to log scale.

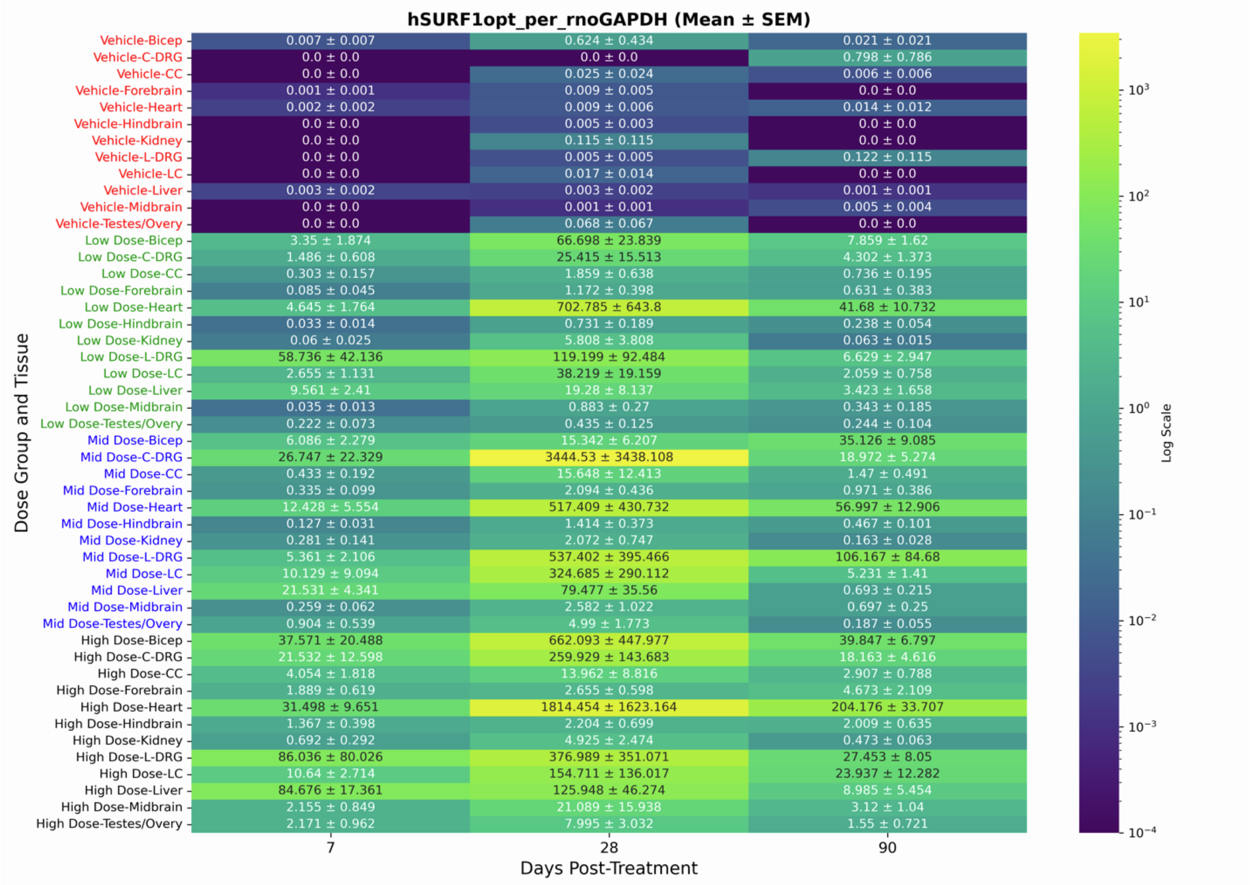

Figure S2. hSURF1opt mRNA expression level normalized to rat endogenous GAPDH copy number. Each data point represents each treatment group, and the number indicates Mean ± SEM (standard error of the mean). Purple shows lower expression and yellow shows higher expression. Color bar is set to log scale.

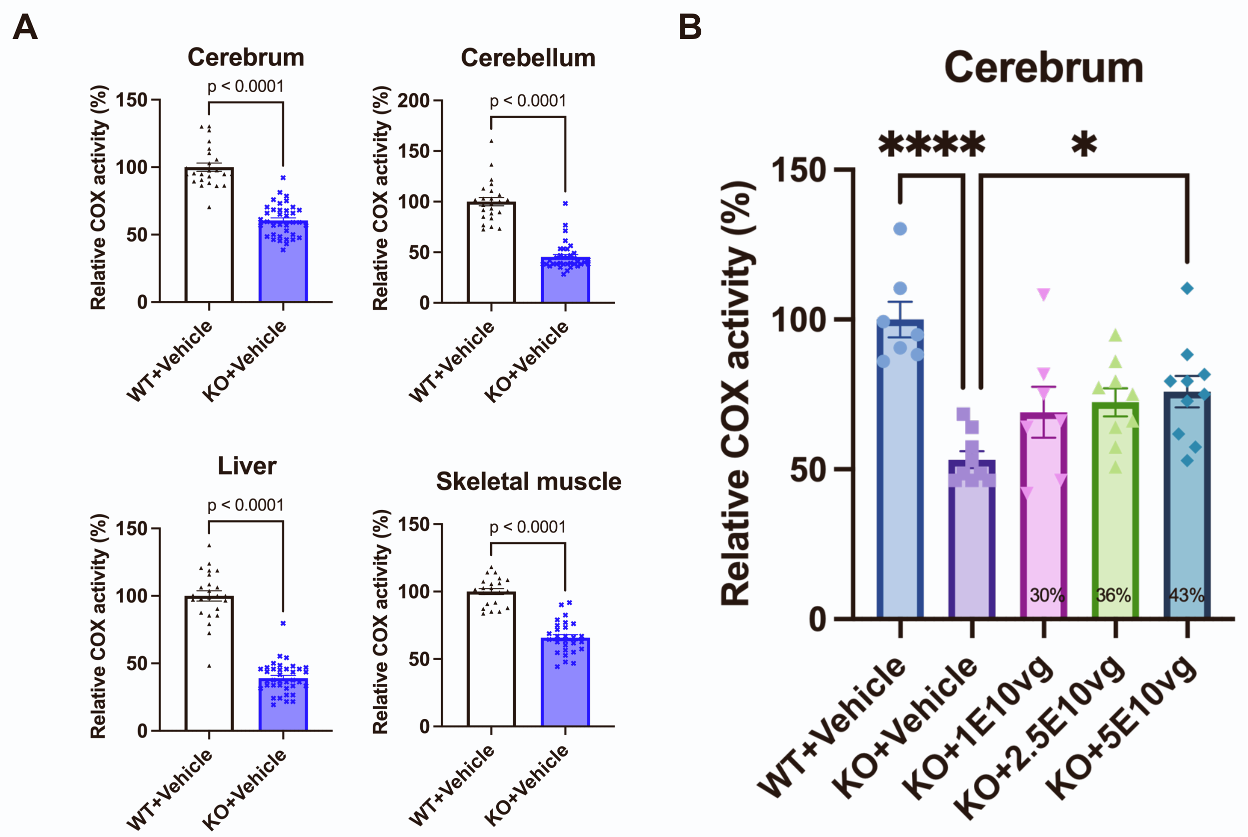

Figure S3. COX activity comparisons in AAV9/JeT-hSURF1v2 efficacy study. (A) COX activity of cerebrum, cerebellum, liver and skeletal muscle was reduced in KO+Vehicle group compared with WT+Vehicle group**.** Student's t-tests were performed. **(B)** AAV9/Jet-hSURF1v2 showed similar improvement in COX activity in cerebrum when injected at different doses through ICV. COX activity of cerebrum from each treatment group. All data were normalized to the average of WT+Vehicle group. Each data point represents measurement from an individual animal, with bars representing the mean ± SEM (standard error of the mean). Tukey's multiple comparison method following an ordinary one-way ANOVA were performed. *p-value <0.05; ****p-value<0.0001. The percentage of improvement compared with the KO+Vehicle group from each treatment is provided at the bottom of the respective column.

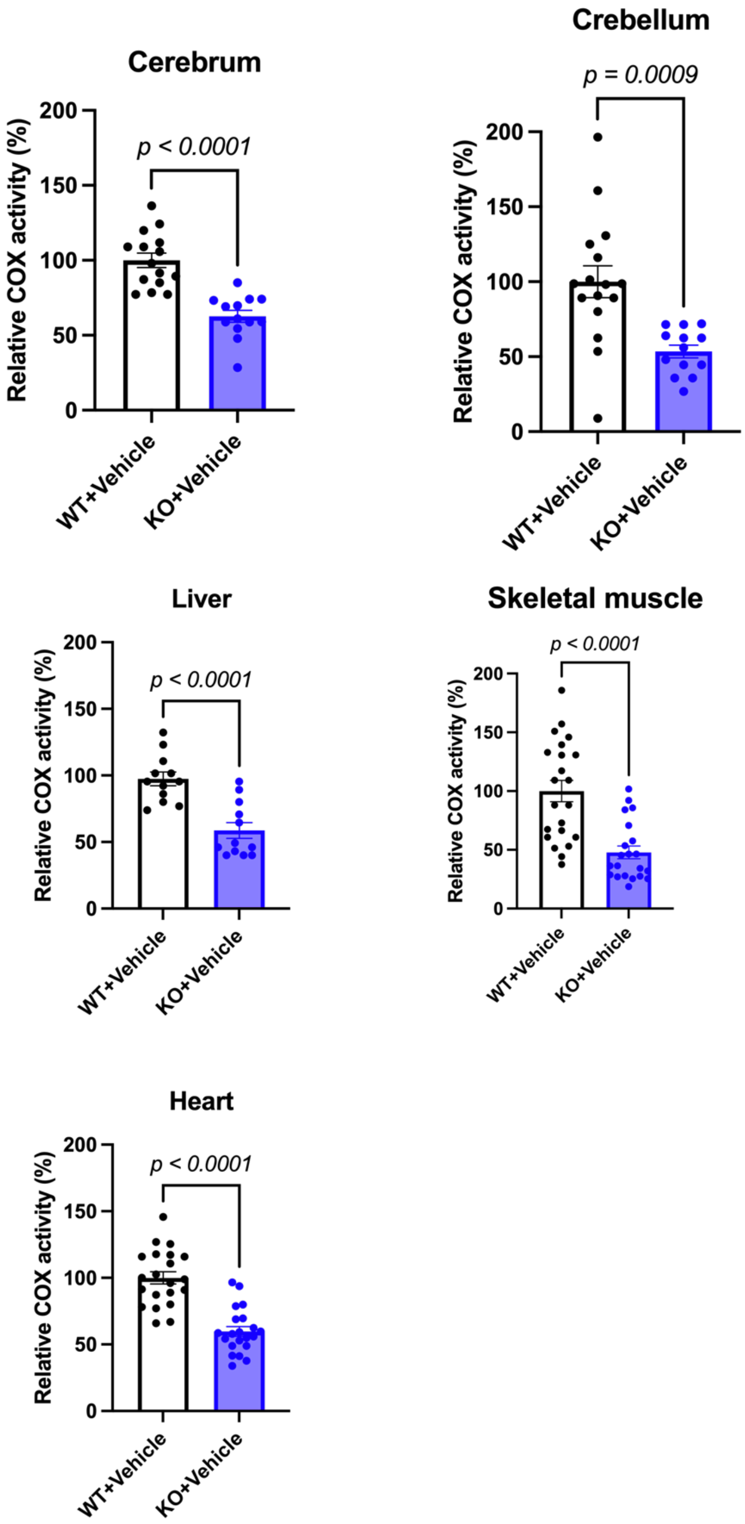

Figure S4. COX activity comparisons in AAV9/JeTI-hSURF1v2 efficacy study. COX activity of cerebrum, cerebellum, liver and skeletal muscle was reduced in KO+Vehicle group compared with WT+Vehicle group. Student's t-tests were performed.

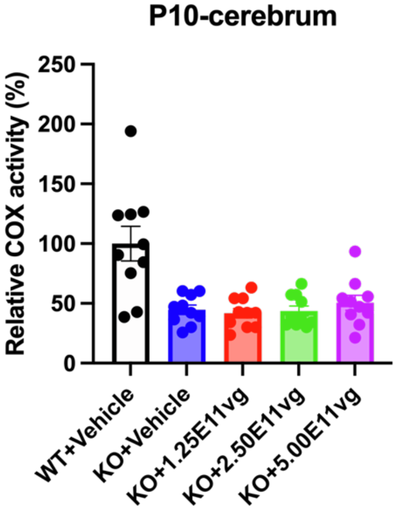
P10-cerebrum
Relative COX activity (%)
250
200
150
100
50
0
WT+Vehicle
KO+Vehicle
KO+1.25E11vg
KO+2.50E11vg
KO+5.00E11vg

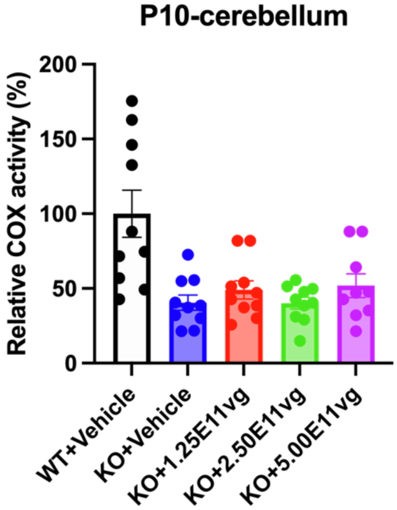
P10-cerebellum
Relative COX activity (%)
200
150
100
50
0
WT+Vehicle
KO+Vehicle
KO+1.25E11vg
KO+2.50E11vg
KO+5.00E11vg

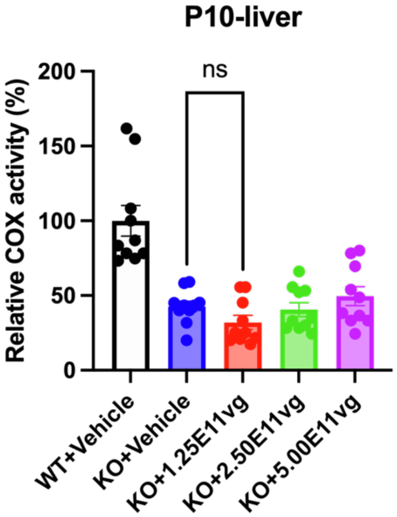
P10-liver
ns
Relative COX activity (%)
200
150
100
50
0
WT+Vehicle
KO+Vehicle
KO+1.25E11vg
KO+2.50E11vg
KO+5.00E11vg

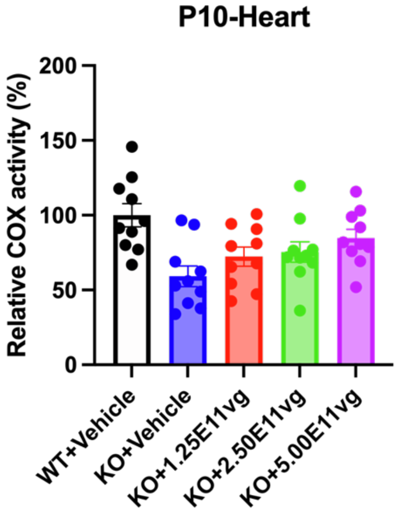
P10-Heart
Relative COX activity (%)
200
150
100
50
0
WT+Vehicle
KO+Vehicle
KO+1.25E11vg
KO+2.50E11vg
KO+5.00E11vg

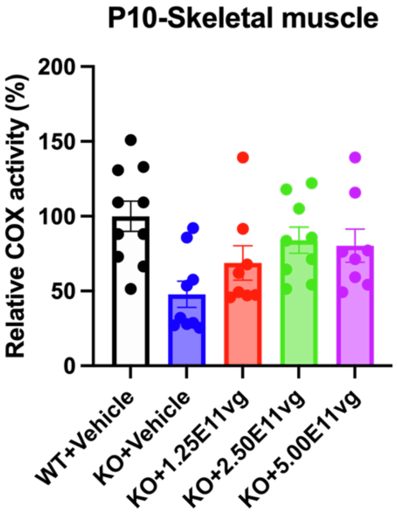
P10-Skeletal muscle
Relative COX activity (%)
200
150
100
50
0
WT+Vehicle
KO+Vehicle
KO+1.25E11vg
KO+2.50E11vg
KO+5.00E11vg

Figure S5. COX activity from *Surf1* KO mice dosed at PND10 with three doses of AAV9/JeTI-h*SURF1*v2. KO mice were dosed either with vehicle (PBS+5% Sorbitol), or with AAV9/SURF1 vector at 3 different doses of low (1.25E11 vg), mid (2.50E11 vg) or high (5.00E11 vg) per animal. All data were normalized to the average of KO+Vehicle mice. Each data point represents measurement from an individual animal, with bars representing the Mean ± SEM (standard error of the mean).

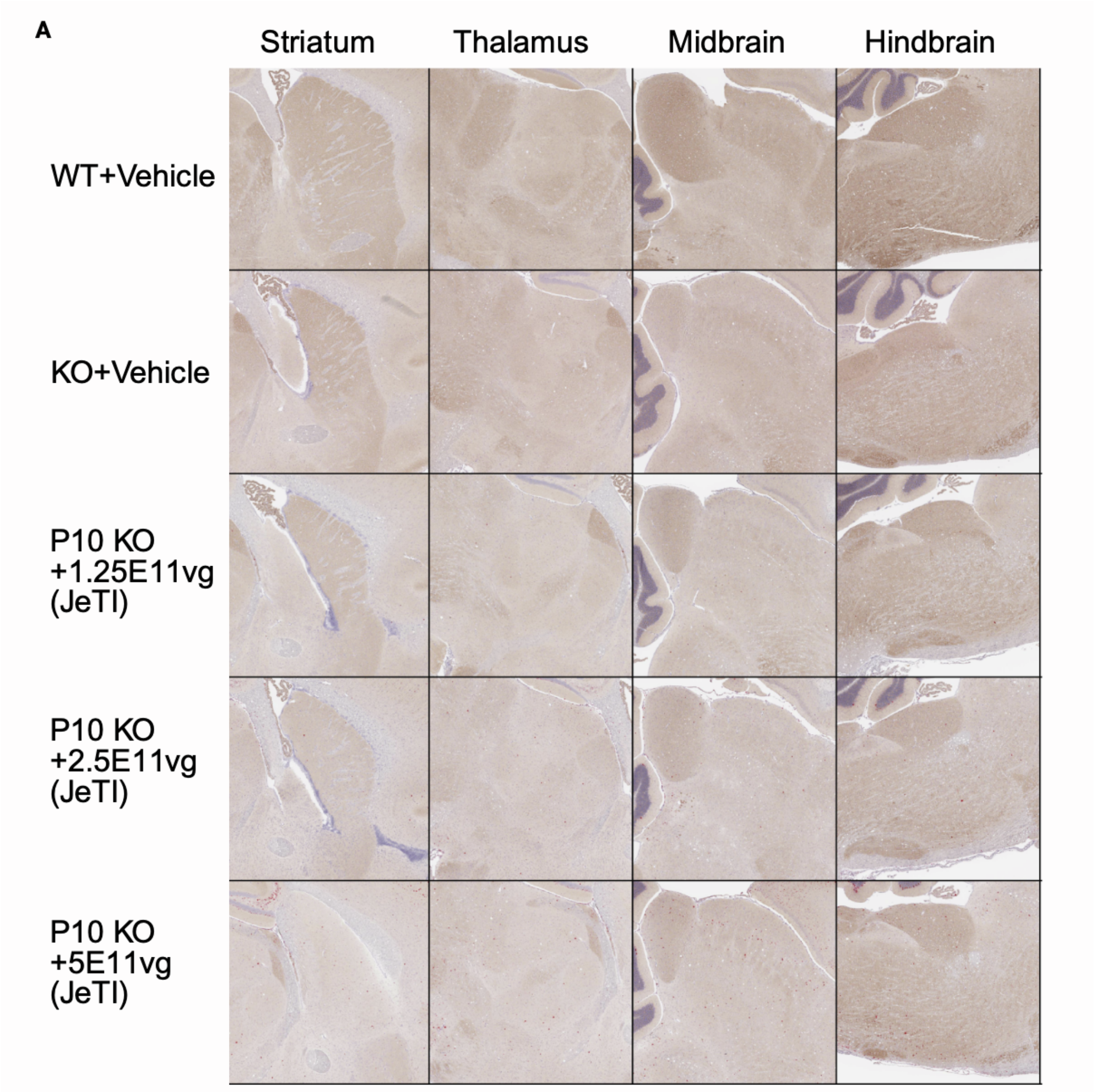
A
Striatum
Thalamus
Midbrain
Hindbrain
WT+Vehicle
KO+Vehicle
P10 KO
+1.25E11vg
(JeTI)
P10 KO
+2.5E11vg
(JeTI)
P10 KO
+5E11vg
(JeTI)

B

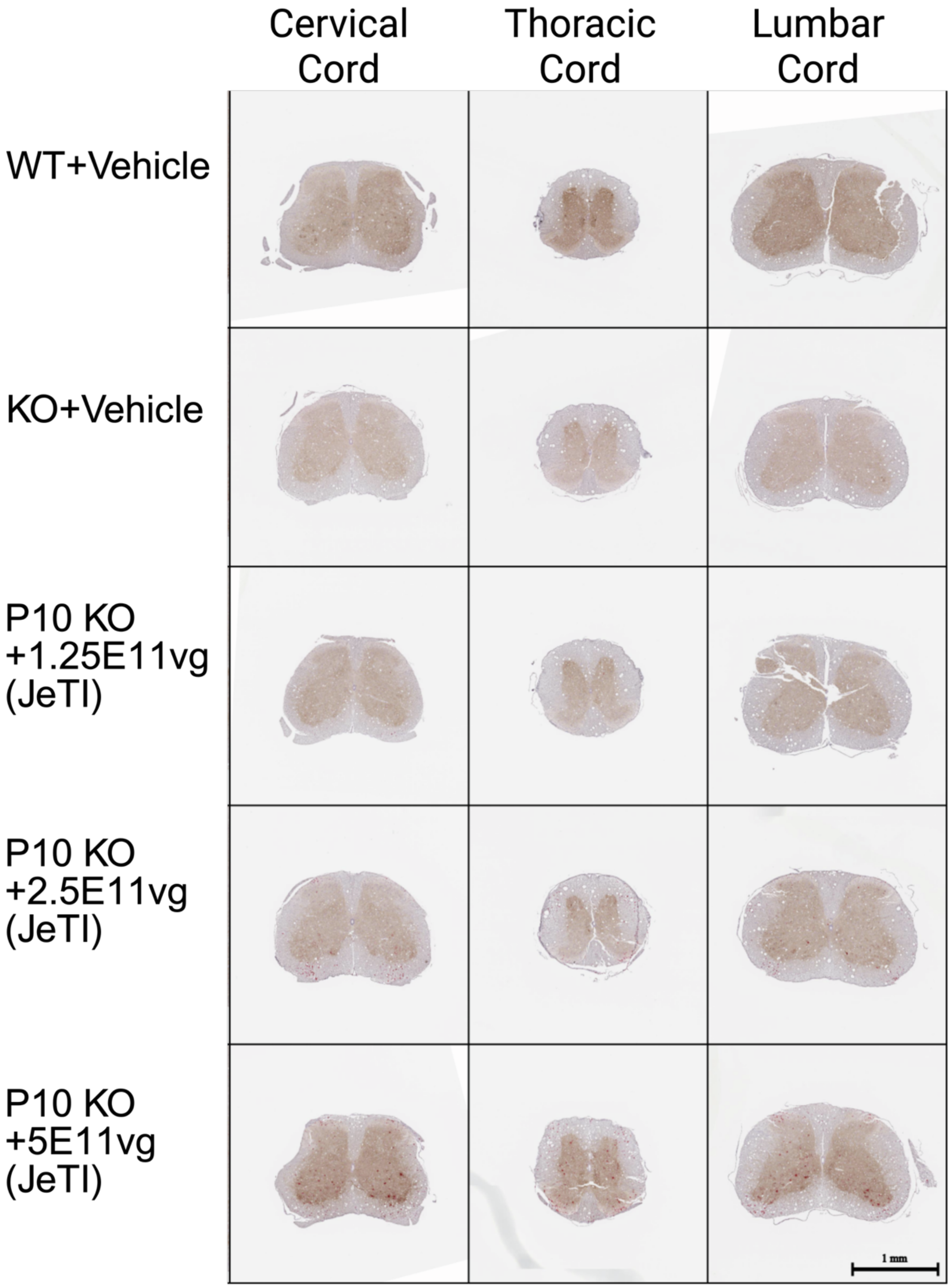
Cervical Cord
Thoracic Cord
Lumbar Cord
WT+Vehicle
KO+Vehicle
P10 KO +1.25E11vg (JeTI)
P10 KO +2.5E11vg (JeTI)
P10 KO +5E11vg (JeTI)
1 mm

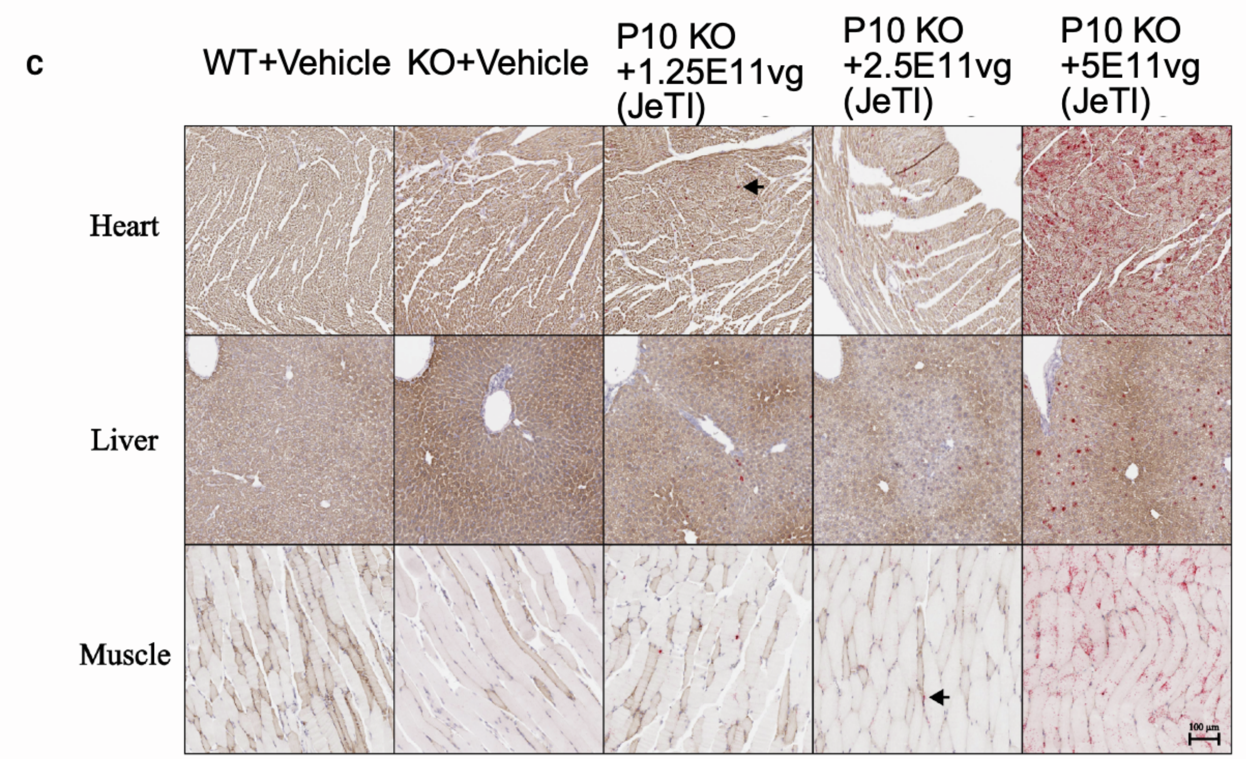

Figure S6. Biodistribution and mRNA expression of dose response group of SURF1 -/- mice treated at P10. KO mice were dosed either with vehicle (PBS+5% Sorbitol), or with AAV9/SURF1 vector at doses low (1.25E11 vg), mid (2.5E11 vg) or high (5E11 vg) per animal. Red spots (example black arrow) show increased *SURF1opt* transgene mRNA expression in the brain (striatum, thalamus, midbrain, hind brain), spinal cord (cervical, thoracic, lumbar), heart, liver, and skeletal muscle (triceps).

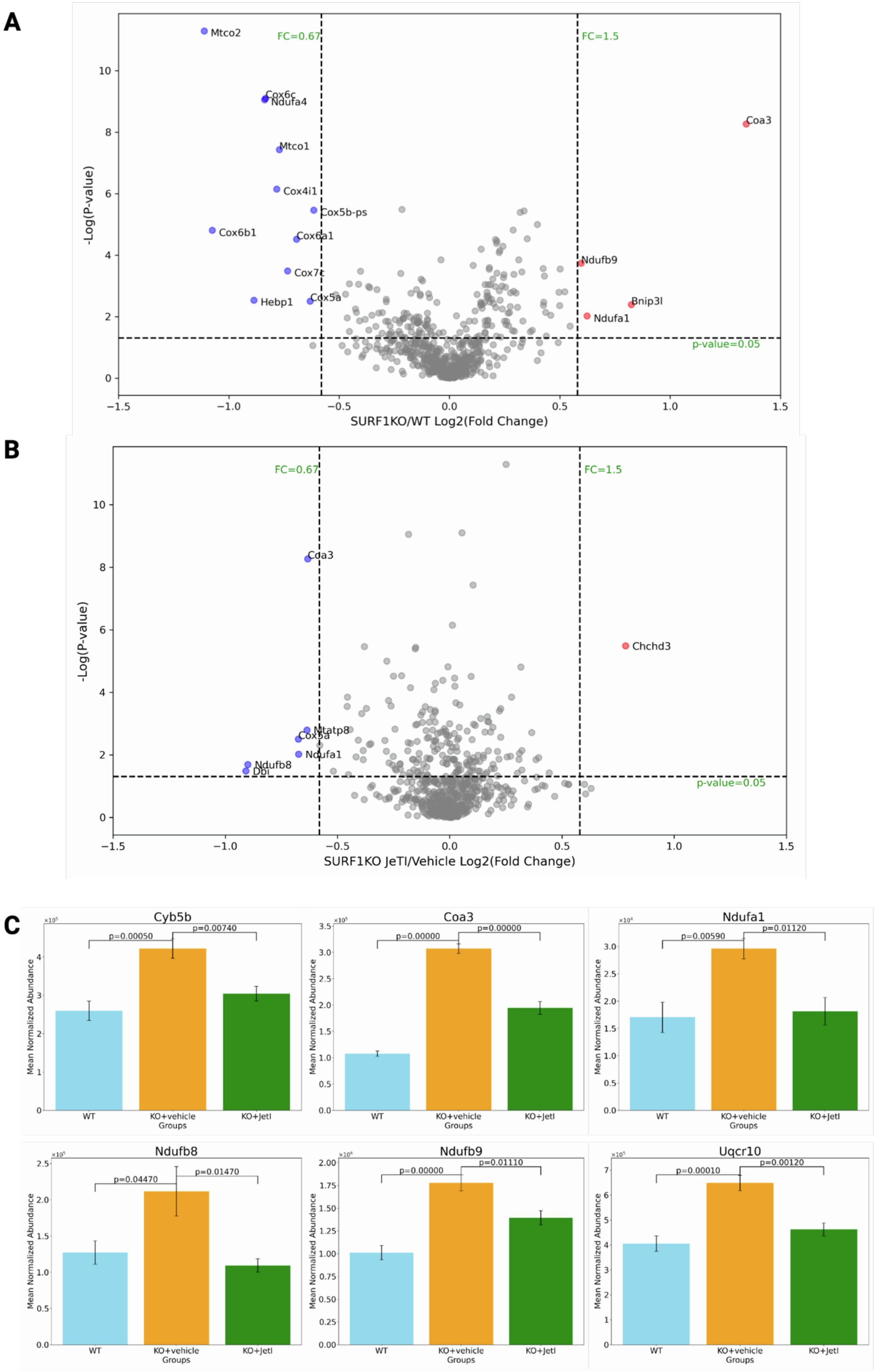
A
FC=0.67
FC=1.5
p-value=0.05
Mtco2
Cox6c
Ndufa4
Mtco1
Cox4i1
Cox5b-ps
Cox6b1
Cox6a1
Cox7c
Hebp1
Cox5a
Coa3
Ndufb9
Bnip3l
Ndufa1
-Log(P-value)
SURF1KO/WT Log2(Fold Change)
B
FC=0.67
FC=1.5
p-value=0.05
Coa3
Chchd3
Mtatp8
Cox5a
Ndufa1
Ndufb8
Dbi
-Log(P-value)
SURF1KO JeTI/Vehicle Log2(Fold Change)
C
Cyb5b
p=0.00050
p=0.00740
Coa3
p=0.00000
p=0.00000
Ndufa1
p=0.00590
p=0.01120
Ndufb8
p=0.04470
p=0.01470
Ndufb9
p=0.00000
p=0.01110
Uqcr10
p=0.00010
p=0.00120
Mean Normalized Abundance
WT
KO+vehicle
KO+JetI
Groups

Figure S7. AAV9/JeTI-h*SURF1*v2 corrected mitochondrial protein expression profile in *Surf1* KO mice dosed at PND10. A) protein expression fold change of *Surf1* KO over WT; FC, fold change. Threshold lines (dotted lines) represent FC of 0.67 and 1.5 fold, and a *p*-value of 0.05 comparing all three groups by one-way ANOVA. **B)** protein expression fold change of *Surf1* KO treated with JeTI-hSURF1v2 over vehicle-treated *Surf1* KO. **C)** Protein expression changes of Cyb5b, Coa3, Ndufa1, Ndufb8, Ndufb9, and Uqcr10. Data represent Mean ± SEM (standard error of the mean). *p*-values were calculated using Tukey's multiple comparisons method following ordinary one-way ANOVA.

Table S1. Non-GLP histopathological evaluation findings

Table S2. Analyzed data of proteomics study in Figure S7

Table S3: GLP toxicology study for AAV9CBh-SURF1v1
