# Supplementary material for: Improved AAV9-based gene therapy design for SURF1-related Leigh syndrome with minimal toxicity
Source: Mol Ther Methods Clin Dev. 2025 Aug 11;33(3):101554. doi: 10.1016/j.omtm.2025.101554 (PMC12398787; doi:10.1016/j.omtm.2025.101554)
Supplement: Data S1. GLP toxicology study for AAV9CBh-SURF1v1 [file mmc4.pdf]

Final Report

Testing Facility Study No. 2954-001

Sponsor Reference No. UTSW.Gray-002

A Single Dose Toxicity Study of AAV9/SURF1 Administered by Intrathecal Injection in Rats

GLP

SPONSOR:
University of Texas Southwestern Medical Center
5323 Harry Hines Boulevard
Dallas, TX 75390
USA

TESTING FACILITY:
Charles River Laboratories, Inc.
54943 North Main Street
Mattawan, MI 49071
USA

TABLE OF CONTENTS

LIST OF FIGURES 4
LIST OF TABLES 5
LIST OF APPENDICES 6
QUALITY ASSURANCE STATEMENT 7
COMPLIANCE STATEMENT AND REPORT APPROVAL 8
1. RESPONSIBLE PERSONNEL 9
2. SUMMARY 10
3. INTRODUCTION 11
4. MATERIALS AND METHODS 12
4.1. Test Materials 12
4.1.1. Test Article and Vehicle Characterization 12
4.1.2. Test Material Identification 12
4.2. Reserve Samples 12
4.3. Test Article and Vehicle Inventory and Disposition 12
4.4. Dose Formulation and Analysis 13
4.4.1. Preparation of Formulations 13
4.4.2. Preparation Details 13
4.4.3. Sample Collection and Analysis 13
4.5. Test System 13
4.5.1. Receipt 13
4.5.2. Justification for Test System and Number of Animals 13
4.5.3. Animal Identification 13
4.5.4. Environmental Acclimation 14
4.5.5. Selection, Assignment, Replacement, and Disposition of Animals 14
4.5.6. Husbandry 14
4.6. Experimental Design 15
4.6.1. Predose Procedures 15
4.6.2. Procedure-Related Medications 15
4.6.3. Dose Administration 15
4.6.4. Postdose Procedures 16
4.6.5. Justification of Route and Dose Levels 16
4.7. In-life Procedures, Observations, and Measurements 16
4.8. Laboratory Evaluations 17
4.8.1. Clinical Pathology 17
4.8.2. Bioanalysis Evaluation 17
4.9. Terminal Procedures 18
4.9.1. Unscheduled Deaths 18
4.9.2. Scheduled Euthanasia 18

4.9.3. Necropsy 18
4.9.4. Organ Weights 18
4.9.5. Tissue Collection and Preservation....18
4.9.6. Tissue Collection for Biodistribution Analysis 19
4.9.7. Tissue Collection for Splenocyte Analysis....19
4.9.8. Histology....19
4.9.9. Microscopic Evaluations....19
5. STATISTICS 19
5.1. Parametric/Non-parametric Comparisons....20
6. COMPUTERIZED SYSTEMS....20
7. RETENTION AND DISPOSITION OF RECORDS, SAMPLES, AND SPECIMENS 21
8. RESULTS 22
8.1. Surgical Procedures 22
8.2. Mortality 22
8.3. Veterinary Treatments 22
8.4. In-life Examinations....22
8.4.1. Detailed Clinical Observations 22
8.4.2. Body Weight and Body Weight Gains....22
8.4.3. Caged Food Consumption 23
8.5. Clinical Pathology....23
1.1. Unscheduled Collections 23
8.6. Hematology....23
8.7. Coagulation....23
8.8. Clinical Chemistry 23
8.9. Microscopic Evaluations....23
8.9.1. Animal Mortality 24
8.9.2. Macroscopic Observations....24
8.9.3. Microscopic Observations 24
9. CONCLUSION....28
10. REFERENCES 29

LIST OF FIGURES

Figure 1 Summary of Mean Body Weigh Values 30
Figure 2 Summary of Mean Body Weight Gain Values....................32

LIST OF TABLES

Table 1 Summary of Clinical Observations 36
Table 2 Summary of Body Weights 44
Table 3 Summary of Body Weight Gains 48
Table 4 Summary of Food Consumption 52

LIST OF APPENDICES

Appendix 1 Deviations, Amended Protocol, and Protocol 58
Appendix 2 Test and Control Material Characterization 131
Appendix 3 Individual Mortality 134
Appendix 4 Individual Clinical Observations 142
Appendix 5 Individual Animal Exam Observation and Treatment Report 164
Appendix 6 Individual Body Weights 180
Appendix 7 Individual Body Weight Gains 197
Appendix 8 Individual Caged Food Consumption Values 214
Appendix 9 Clinical Pathology Report 241
Appendix 10 Pathology Report 392

QUALITY ASSURANCE STATEMENT

Study Number: 2954-001

This Study has been audited by Quality Assurance in accordance with the applicable Good Laboratory Practice regulations. Reports were submitted in accordance with SOPs as follows:

		**Dates Findings Submitted to:**	
**Date(s) of Audit**	**Phase(s) Audited**	**Study Director**	**Testing Facility Management**
26Aug2020	Dose Administration	26Aug2020	26Aug2020
28Jul2021 - 29Jul2021	Report Tables	29Jul2021	29Jul2021
28Jul2021 - 29Jul2021	Data Review - Pathology	29Jul2021	29Jul2021
02Aug2021 - 03Aug2021	Report - Materials and Methods	03Aug2021	03Aug2021
02Aug2021 - 03Aug2021	Data Review - Technical Operations	03Aug2021	03Aug2021
10Aug2021 - 11Aug2021	Phase Report - Clinical Pathology	11Aug2021	11Aug2021
19Aug2021	Report Tables	19Aug2021	19Aug2021
10Aug2023 - 15Aug2023	Final Report	15Aug2023	15Aug2023

In addition to the above-mentioned audits, process-based and/or routine facility inspections were also conducted during the course of this study. Inspection findings, if any, specific to this study were reported by Quality Assurance to the Study Director and Testing Facility Management and listed as a Phase Audit on this Quality Assurance Statement.

The Quality Assurance Statements for any work conducted at Test Sites were reviewed and included in the appropriate section of this report, as applicable.

The Final Report has been reviewed to assure that it accurately describes the materials and methods, and that the reported results accurately reflect the raw data.

COMPLIANCE STATEMENT AND REPORT APPROVAL

The study was conducted in accordance with the U.S. Department of Health and Human Services, Food and Drug Administration, United States Code of Federal Regulations (CFR), Title 21, Part 58: Good Laboratory Practice for Nonclinical Laboratory Studies and as accepted by Regulatory Authorities throughout the European Union (OECD Principles of Good Laboratory Practice), Japan (MHLW), and other countries that are signatories to the OECD Mutual Acceptance of Data Agreement.

Exceptions from the above regulations are listed below.

- Characterization of the test and control articles were performed by the Sponsor or Sponsor contracted site according to established standard operation procedures (SOPs), controls, and approved test methodologies to ensure integrity and validity of the results generated. These analyses were not conducted in compliance with Good Laboratory Practices (GLP) or Good Manufacturing Practices (GMP) regulations.

This study was conducted in accordance with the procedures described herein. All deviations authorized/acknowledged by the Study Director are documented in the Study Records. The report represents an accurate and complete record of the results obtained.

All electronic signatures appear at the end of the document upon finalization.

1. RESPONSIBLE PERSONNEL

Role/Phase	Quality Assurance Unit	Name	Contact Information
Site Head / General Manager	Charles River	Robert Stachlewitz, PhD, DABT	Address as cited for Testing Facility
Senior Director, Safety Evaluation	Charles River	Christopher Papagiannis, BS	Address as cited for Testing Facility
Study Director	Charles River	Sarah Davis, BS, LATG	Address as cited for Testing Facility
**Individual Scientist (IS) at Testing Facility**			
Study Director from 14 Aug 2020 to 28 Apr 2021	Charles River	Barak Gunter, PhD	Address as cited for Testing Facility
Clinical Pathology	Charles River	Charles E Wiedmeyer, DVM, PhD, DACVP	Address as cited for Testing Facility
**Principal Investigator (PI)**			
Anatomic Pathology	Test Site	Jessica Hoane, DVM, MTOX, DACVP, DABT	StageBio<br>8415 Progress Drive, Suite Q<br>Frederick, MD 21701

2. SUMMARY

The objective of this study was to characterize the toxicity, biodistribution, and gene expression of the test article, AAV9/SURF1, for the treatment of SURF1 Leigh Syndrome. The test article was administered via intrathecal injection once on Day 1 to CD® [Crl:CD®(SD)] rats. The study design was as follows:

Text Table 1
Experimental Design

Group No.	Treatment	Dose Level (vg)	Dose Volume[a] (µL)	Dose Concentration (vg/µL)	No. of Animals					
					Day 8 Necropsy[c]		Day 29 (±1) Necropsy[d]		Day 91 (±4) Necropsy	
					M	F	M	F	M	F
1	Vehicle	0	60[a]<br>74[b]	0	5	5	5	5	5	5
2	Low Dose	0.28E12	20[a]<br>25[b]	1.38E10[a]<br>1.12E10[b]	5	5	5	5	5	5
3	Mid Dose	0.83E12	20[a]<br>25[b]	4.15E10[a]<br>3.36E10[b]	6	5	5	5	5	5
4	High Dose	2.49E12	60[a]<br>74[b]	4.15E10[a]<br>3.36E10[b]	5	5	5	5	5	5

[a] Lot 1
[b] Lot 2
[c] Three of the animals from Group 4 received the same total vg dose, but in a 74 µL volume due to lower test article dose concentration in Lot 2 of 3.36E10 vg/µL.
[d] All animals received the same total vg dose assigned to each respective group, but in a 24% higher volume due to test article dose concentration in Lot 2.
M – Male, F – Female, No. – Number

The following parameters and endpoints were evaluated in this study: mortality, clinical signs, body weights, body weight gains, food consumption, clinical pathology parameters (hematology, coagulation, and clinical chemistry), biodistribution (serum, cerebral spinal fluid (CSF), and tissue), organ weights, and macroscopic and microscopic examinations.

Administration of AAV9/SURF1 was not associated with any bodyweight or food consumption changes that were considered adverse. All animals survived to their scheduled terminal necropsies with 1 exceptions, Animal No. 3008 (0.83E12, male), was euthanized in extremis on Day 16 following no improvement of hind limb impairment.

AAV9/SURF1-related clinical pathology changes included minimally higher WBC attributed by higher lymphocytes on Day 29 in females at 2.49E12 (Group 4). This finding lacked a microscopic correlate.

A single bolus of AAV9/SURF1 at doses of 0.28E12 vg, 0.83E12 vg, or 2.49E12 vg was administered intrathecally into the lumbar cistern in male and female Sprague Dawley rats with scheduled terminations at Days 8, 29, and 91. Early death occurred in a single male dosed with AAV9/SURF1 at 0.83E12 vg; the cause of moribundity was due to procedure/test article-related gray matter degeneration/necrosis predominantly centered on the thoracic spinal cord.
There were no macroscopic observations at Days 8, 29, or 91 that were considered to be associated with the administration of AAV9/SURF1.

At the scheduled terminations, single bolus administration of AAV9/SURF1 resulted in microscopic changes in the white matter of the spinal cord at Days 29 and 91 (increased incidence and/or severity of nerve fiber degeneration); the dorsal root ganglia (neuron degeneration/necrosis, mononuclear cell infiltrates, and/or hypertrophy/hyperplasia in [satellite] glial cells) at Days 8, 29, and 91; the dorsal spinal nerve roots (nerve fiber degeneration) at Days 29 and 91; the spinal nerve roots adjacent to the spinal cord at Days 29 and 91 (nerve fiber degeneration); the tibial and sciatic nerves at Days 29 and 91 (increased incidence and/or severity of nerve fiber degeneration and/or Schwann cell hypertrophy/hyperplasia); the heart at Days 29 and 91 (myocardium degeneration/necrosis, mononuclear cell infiltrates, and/or fibrosis); and the liver at Days 8, 29, and 91 (increased incidence and severity of mixed cell infiltrates, single cell necrosis of hepatocytes, Kupffer cell hypertrophy/hyperplasia, and/or increased mitotic figures).
Microscopic findings of the gray matter of the spinal cord (degeneration/necrosis, increases in glial cells, and/or mononuclear cell infiltrates) in single animals at Day 8 and Day 29 were considered related to the inadvertent administration of the test material likely within the central canal. In addition, single animals at Day 29 and Day 91 had locally extensive spinal cord changes consistent with inadvertent needle stick into the spinal cord. The degeneration/necrosis of the gray matter of the spinal cord (in combination with the likely inadvertent administration of the AAV9/SURF1 test article into the central canal of the spinal cord), the neuronal degeneration/necrosis within the lumbar DRG, and the heart myocardial changes (degeneration/necrosis, mononuclear cell infiltration, and/or fibrosis) at moderate and greater severity were considered adverse. The test article-related microscopic changes did not, in general, exhibit a dose-dependent trend of incidence and/or severity; however, there was timepoint-dependent variability in occurrence/severity of some microscopic findings. Any variation in microscopic findings due to the test article lot administered, Lot 1 or Lot 2, could not be definitively determined.

3. INTRODUCTION

The objective of this study was to characterize the toxicity, biodistribution, and gene expression of AAV9/SURF1 for the treatment of SURF1 Leigh Syndrome.

The design of this study was based on the following guidelines.

- Food and Drug Administration (FDA) Guidance for Industry. *Preclinical Assessment of Investigational Cellular and Gene Therapy Products.*
- FDA Draft Guidance for Industry. *Human Gene Therapy for Rare Diseases.*
- International Council of Harmonisation (ICH) Harmonised Tripartite Guideline S6 (R1). *Preclinical Safety Evaluation of Biotechnology-Derived Pharmaceuticals.*
- Final Rules of the Animal Welfare Act regulations (Code of Federal Regulations [CFR], Title 9).
- *Public Health Service Policy on Humane Care and Use of Laboratory Animals* (Office of Laboratory Animal Welfare, Current edition).
- *Guide for the Care and Use of Laboratory Animals* (National Research Council, Current edition).

The deviations, last protocol amendment, and protocol are presented in Appendix 1.

Study Initiation Date:	14 Aug 2020
Initiation of Dosing:	26 Aug 2020
Completion of In-life:	26 May 2021
Experimental Starting Date:	18 Aug 2020
Experimental Completion Date:	02 Nov 2022

4. MATERIALS AND METHODS

4.1. Test Materials

4.1.1. Test Article and Vehicle Characterization

The Sponsor has provided to the Testing Facility documentation of the identity, strength, purity, composition, and stability for the test article. A Certificate of Analysis has been provided to the Testing Facility and is presented in Appendix 2.

Information for the vehicle was limited to that provided by the respective manufacturers.

4.1.2. Test Material Identification

Text Table 2
Test Material Identification

	**Test Material**	**Test Material**
**Identification:**	Surf1 Tox	Surf1
**Batch/Lot No.:**	TOX-07-20-196	TX-07-20-001
**Expiration/Retest Date:**	Not Available	
**Storage Conditions:**	Refrigerated at 2°C to 8°C, protected from light	Frozen at -60°C to -90°C, protected from light
**Provided by:**	Sponsor	

Text Table 3
Vehicle Identification

	**Vehicle**	**Vehicle**
**Identification:**	Formulation Buffer	Vehicle
**Batch/Lot No.:**	NA	TX-10-21-002
**Expiration/Retest Date:**	Not available	
**Storage Conditions:**	Refrigerated at 2°C to 8°C, protected from light	Frozen at -60°C to -90°C, protected from light
**Provided by:**	Sponsor	

4.2. Reserve Samples

For each batch (lot) of test article and vehicle, a reserve sample was collected and maintained under the appropriate storage conditions by the Testing Facility.

4.3. Test Article and Vehicle Inventory and Disposition

The test materials were received by the Testing Facility for distribution as needed. Records of the receipt, distribution, storage, and disposition of test materials (including empty containers of Sponsor-provided materials) were maintained.

4.4. Dose Formulation and Analysis

4.4.1. Preparation of Formulations

Dose formulations were divided into aliquots where required to allow to be dispensed on each dosing occasion.

Text Table 4
Formulation Frequency of Preparation

Dose Formulation	Frequency of Preparation	Storage Conditions, Set to Maintain
Vehicle	Used as received	Refrigerated at 2°C to 8°C
Dose formulations	Prior to dosing[a]	Refrigerated at 2°C to 8°C

[a] 60 days of established stability at refrigerated (2 to 8°C) storage conditions was established.

Any residual volumes from each dosing occasion were retained and stored refrigerated at 2°C to 8°C. These retained volumes were used on subsequent dosing days.

4.4.2. Preparation Details

Dosing formulations were prepared at appropriate concentrations to meet dose level requirements.

4.4.3. Sample Collection and Analysis

The test and control articles were used as received from the Sponsor; therefore, samples for dose formulation analysis were not collected by the Testing Facility.

4.5. Test System

4.5.1. Receipt

On 18 Aug 2020, 24 Sept 2020, 08 Oct 2020, and 19 Apr 2021, CD® [Crl:CD®(SD)] rats were received from Charles River Laboratories, Raleigh, North Carolina. The animals were approximately 7 to 8 weeks old and weighed between 143 g and 255 g at initiation of dosing.

4.5.2. Justification for Test System and Number of Animals

The current state of scientific knowledge and the applicable guidelines cited previously did not provide acceptable alternatives, in vitro or otherwise, to the use of live animals to accomplish the purpose of this study. “The development of knowledge necessary for the improvement of the health and well-being of humans as well as other animals requires in vivo experimentation with a wide variety of animal species” (Federal Register). “Whole animals are essential in research and testing because they best reflect the dynamic interactions between the various cells, tissues, and organs comprising the human body” (NIH Guide).

The rat is the usual rodent model used for evaluating the toxicity of various classes of chemicals and for which there is a large historical database (CDER).

The total number of animals used in this study was considered to be the minimum required to properly characterize the effects of the test article and was designed such that it did not require an unnecessary number of animals to accomplish its objectives.

4.5.3. Animal Identification

Each animal was identified using a subcutaneously implanted electronic identification chip.

4.5.4. Environmental Acclimation

During the at least 8-day acclimation period, the animals were observed daily with respect to general health and any signs of disease.

4.5.5. Selection, Assignment, Replacement, and Disposition of Animals

Animals were randomly assigned to groups upon receipt. Males and females were randomized separately.

The disposition of all animals was documented in the study records.

4.5.6. Husbandry

4.5.6.1. Housing

The animals were pair or group-housed in solid bottom cages with nonaromatic bedding. The housing was equipped with an automatic watering valve as specified in the *USDA Animal Welfare Act* (9 CFR, Parts 1, 2 and 3) and as described in the *Guide for the Care and Use of Laboratory Animals* (National Research Council, Current edition).

Each cage was clearly labeled with study, group, animal number, and sex.

4.5.6.2. Animal Enrichment

Psychological/environmental enrichment was provided according to Testing Facility SOP.

4.5.6.3. Environmental Conditions

Target temperatures of 68°F to 79°F with a target relative humidity of 30% to 70% were maintained. A 12-hour light/12-hour dark cycle was maintained, except when interrupted for designated procedures.

4.5.6.4. Food

Lab Diet® (Certified Rodent Diet #5002, PMI Nutrition International, Inc.) was provided ad libitum except during designated procedures (for a deviation see Appendix 1).

Supplemental diet was provided to the animals as warranted by clinical signs or other changes.

Results of analysis for nutritional components and environmental contaminants are provided by the supplier and are on file at the Testing Facility.

There are no known contaminants in the food that would interfere with this study.

4.5.6.5. Water

Tap water was available ad libitum to each animal via an automatic watering system.

There are no known contaminants in the water that would interfere with this study. The drinking water used was monitored for specified contaminants at periodic intervals according to Testing Facility SOP.

4.5.6.6. Veterinary Care

Veterinary care was available throughout the course of the study, and animals were examined by the veterinary staff as warranted by clinical signs or other changes. All veterinary examinations and recommended therapeutic treatments were documented in the study records and reviewed by

the Study Director. The medical treatments and observations recorded after initiation of dosing are presented in Appendix 5.

4.6. Experimental Design

Text Table 5
Experimental Design

Group No.	Treatment	Dose Level (vg)	Dose Volume (µL)	Dose Concentration (vg/µL)	No. of Animals					
					Day 8 Necropsy[c]		Day 29 (±1) Necropsy[d]		Day 91 (±4) Necropsy	
					M	F	M	F	M	F
1	Vehicle	0	60[a] 74[b]	0	1011-1015	1511-1515	1021 - 1025	1521 - 1525	1006 - 1010	1506-1510
2	Low Dose	0.28E12	20[a] 25[b]	1.38E10[a] 1.12E10[b]	2011-2015	2511-2515	2021 - 2025	2521 - 2525	2006 - 2010	2506-2510
3	Mid Dose	0.83E12	20[a] 25[b]	4.15E10[a] 3.36E10[b]	3011-3015, 4013	3511-3515	3021 - 3025	3521 - 3525	3006 - 3010	3506-3510
4	High Dose	2.49E12	60[a] 74[b]	4.15E10[a] 3.36E10[b]	4011, 4012, 4113, 4021, 4022	4511-4515	4023 - 4027	4521 - 4525	4006 - 4010	4506-4510

[a] Lot 1
[b] Lot 2

4.6.1. Predose Procedures

Preoperative procedures were performed in accordance with Testing Facility SOP. Animals were not fasted overnight prior to surgery.

4.6.2. Procedure-Related Medications

Procedure-related medications were used during the course of the study.

4.6.3. Dose Administration

The vehicle and test article were administered via intrathecal injection once on Day 1. The animals were maintained for a Day 8, 29 ±1, or 91±4 necropsy as outlined in Text Table 5.

Animals were anesthetized, placed in ventral recumbency, and prepped using the aseptic technique. The animal was shaved, eye lube was applied, and cleaned with chlorhexidine scrub and solution.

The dose administration was performed using a gas tight Hamilton syringe with a 27-gauge custom LASI needle (with cannula). Prior to dosing, the needle was primed by having the syringe (with Hamilton needle attached) filled with vehicle. The Hamilton needle was removed, the LASI needle was attached and the vehicle was flushed through the dosing system. 20 µL of vehicle followed by a 5 µL air bubble was drawn into the system and then the appropriate volume of test article. Under fluoroscopic guidance (if needed), a needle was inserted into the lumbar cistern. The location of the needle may have varied based on discretion of the surgeon and the final needle location was recorded (for a deviation see Appendix 1). Once the needle was appropriately placed, the animal was dosed with test article or vehicle, then flushed with vehicle

over 30 ±5 seconds (Groups 1 and 4) and 10 ±5 seconds (Groups 2 and 3). Upon completion of the dose, the needle was removed, and the animal was allowed to recover.

Animal No. 4013 was dosed with Group 3 test material due to insufficient amount of test material on Day 1 and was replaced by a spare animal which became Animal No. 4113.

4.6.4. Postdose Procedures

Postoperative procedures were performed in accordance with Testing Facility SOP.

4.6.5. Justification of Route and Dose Levels

Intrathecal injection is the intended route of administration of this test article in humans.

The dose levels were selected based on information provided by the Sponsor that the target dose conferred efficacy to SURF1 knock-out mice, was found to be well-tolerated in mice up to 10 months post-injection, modeled a relevant dose to human patients, and in an attempt to produce graded responses to the test article. The high-dose level (effectively above a maximum feasible human dose) may produce some toxic effects, but not excessive lethality that would prevent meaningful evaluation. The mid-dose level (target dose) and low-dose level were not expected to produce observable indications of toxicity.

4.7. In-life Procedures, Observations, and Measurements

Text Table 6
General In-life Assessments – Main Study Animals

Parameter	Population(s)	Frequency (minimum required)	Comments
Mortality/Cageside Observations	All Main Study Animals	At least twice daily[a,b] (morning and afternoon) beginning upon arrival through termination/release.	Animals were observed within their cage unless necessary for identification or confirmation of possible findings
Detailed Clinical Observations	All Main Study Animals	Day -1, and weekly throughout the study (for a deviation see Appendix 1).[c]	Animals were removed from the cage. On occasion, clinical observations were recorded at unscheduled intervals.<br><br>Observations included, but were not limited to, evaluation of the skin, fur, eyes, ears, nose, oral cavity, thorax, abdomen, external genitalia, limbs and feet, respiratory and circulatory effects, autonomic effects such as salivation, nervous system effects including tremors, convulsions, reactivity to handling, and unusual behavior.
Individual Body Weights	All Main Study Animals	At receipt, Day -1, and once weekly during the study (for a deviation see Appendix 1).	Fasted weight on the day of necropsy.<br><br>The body weights recorded prior to Day -1 are not reported but are maintained in the study file.

			Body weight gains were calculated for animals between each weighing interval.
Food Consumption	All Main Study Animals	Weekly; from at least Week 1 and throughout the study (for a deviation see Appendix 1).[d]	Quantitatively measured

[a] Included alternate animals until released from study.
[b] Except on days of receipt and necropsy where frequency was at least once daily.
[c] For observations that could not be attributed to an individual animal due to social housing (e.g., watery feces), the observation was noted to each animal in the socialized group.
[d] For observations of reduced appetite that could not be attributed to an individual animal due to social housing, the observations were noted for each animal in the socialized group.

4.8. Laboratory Evaluations

4.8.1. Clinical Pathology

Clinical pathology evaluations were conducted on all animals at necropsy. The materials and methods are described in Appendix 9.

Bone marrow smears were collected and preserved.

4.8.2. Bioanalysis Evaluation

4.8.2.1. Bioanalytical Sample Collection

Blood samples (approximately 1.0 mL) were collected from all animals via cardiac puncture after carbon dioxide inhalation. The animals were fasted prior to blood collection (for a deviation see Appendix 1).

4.8.2.2. Bioanalytical Sample Processing

Blood samples were collected in serum separator tubes and centrifuged at controlled room temperature for 10 minutes. The resulting serum was divided into 2 approximately equal aliquots. All aliquots were stored frozen at -60°C to -90°C.

4.8.2.3. Bioanalytical Sample Analysis

Samples were shipped on dry ice to the Sponsor for analysis of AAV9 concentrations. All analytical work was conducted by the Sponsor using an analytical method developed and qualified by that laboratory.

4.8.2.4. CSF Sample Collection

CSF samples (maximum obtainable volume) were collected from all animals via the cisterna magna. The animals were fasted prior to blood collection (for a deviation see Appendix 1).

4.8.2.5. CSF Sample Processing

Blood samples were collected in tubes and placed on wet ice. The CSF was divided into 2 approximately equal aliquots. All aliquots were stored frozen at -60°C to -90°C.

4.8.2.6. CSF Sample Analysis

Samples were shipped on dry ice to the Sponsor for possible future analysis.

4.9. Terminal Procedures

Terminal procedures are summarized in Text Table 7. For deviations, see Appendix 1.

Text Table 7
Terminal Procedures

Group No.	Scheduled Euthanasia Day	Necropsy Procedures			Histology Processing	Microscopic Evaluation	Biodistribution Collection and Analysis
		Necropsy	Tissue Collection	Organ Weights			
**Unscheduled euthanasia animals**							
Unscheduled euthanasia	NA	X	Full List[a]	NA	Full List[a]	Full List[a]	NA
**Main Study Animals**							
1 through 4	8, 29(±1), or 91(±4)	X	Full List[a]	Full List[a]	Full List[a]	Full List[a]	Full List[a]

Terminal Procedure Tables Footnotes:
X = Procedure conducted; NA = Not applicable.
"Histology Processing"= embedded in paraffin, sectioned, mounted on glass slides, and stained with hematoxylin and eosin.
[a] Protocol designated tissues were collected, weighed, processed, and evaluated as applicable to each procedure.

4.9.1. Unscheduled Deaths

For humane reasons, a main study animal was euthanized as per Testing Facility SOPs. This animal underwent necropsy, and specified tissues were retained. If necessary, animals were refrigerated before necropsy to minimize autolysis.

4.9.2. Scheduled Euthanasia

Main study animals surviving until scheduled euthanasia were euthanized by carbon dioxide inhalation followed by a Testing Facility SOP approved method to ensure death. When possible, the animals were euthanized rotating across dose groups such that similar numbers of animals from each group, including controls were necropsied throughout the day.

4.9.3. Necropsy

Main study animals were subjected to a complete necropsy examination, which included evaluation of the carcass and musculoskeletal system; all external surfaces and orifices; cranial cavity and external surfaces of the brain; and thoracic, abdominal, and pelvic cavities with their associated organs and tissues. The animals were examined carefully for external abnormalities including palpable masses.

Necropsy procedures were performed by qualified personnel with appropriate training and experience in animal anatomy and gross pathology. A veterinary pathologist, or other suitably qualified person, was available.

4.9.4. Organ Weights

Body weights and protocol-designated organ weights were recorded for all surviving animals at the scheduled necropsies and appropriate organ weight ratios were calculated (relative to body and brain weights). Paired organs were weighed together.

4.9.5. Tissue Collection and Preservation

Representative samples of the protocol designated tissues were collected from all animals and preserved in 10% neutral buffered formalin, unless otherwise indicated.

Following the collection of samples for biodistribution and histology, a sample of remaining protocol specified tissues were collected for archival by the Sponsor. The femur was wrapped in phosphate buffered saline (PBS) soaked gauze prior to being flash frozen. Following collection, all samples were flash frozen in liquid nitrogen and stored frozen at -60°C to -90°C.

4.9.6. Tissue Collection for Biodistribution Analysis

Care was taken to ensure that cross contamination did not occur. Gloves were changed between collection and dissection of each tissue for analysis. In addition, non-disposable instruments were wiped down with a 10% bleach solution, rinsed with water, followed by a wipe down of 100% ethanol between each of the specified organs.

Brain and spinal cord were collection first, followed by the liver, kidney, lung, heart, spleen, then all remaining tissues. Two samples of each tissue were obtained for biodistribution analysis. The two samples were weighed, placed into two separate tubes, flash frozen in liquid nitrogen, and stored frozen at -60°C to -90°C. Tissues were shipped on dry ice to the Sponsor for analysis of vector DNA biodistribution and gene expression. All analytical work will be conducted by the Sponsor, using an analytical method developed and qualified by that laboratory.

4.9.7. Tissue Collection for Splenocyte Analysis

Following collection of two spleen samples for biodistribution and a section for histology, the remaining spleen was placed into a vial containing RPMI media and stored refrigerated at 2°C to 8°C on wet ice before processing. Samples were processed according to Testing Facility SOP, except all washes were performed with RPMI media. All samples were shipped on dry ice to the Sponsor for analysis of T-cell responses against AAV9 and SURF1. All analytical work will be conducted by the Sponsor, using an analytical method developed and qualified by that laboratory.

4.9.8. Histology

Protocol designated tissues were embedded in paraffin, sectioned, mounted on glass slides, and stained with hematoxylin and eosin.

4.9.9. Microscopic Evaluations

Fixed hematoxylin and eosin-stained paraffin sections from protocol-designated sections of tissues were processed to slide and all required slides were shipped under ambient condition to TPS (a StageBio Company), Frederick, Maryland, for microscopic evaluation. Special stains were used by the pathologist as needed to aid in the diagnosis of specific lesions. Documentation of use of special stains is maintained in the study file. Photomicrographs of representative lesions seen during the microscopic examination, including those considered to be treatment- related, were taken. Representative photomicrographs are not reported and are maintained in the study file.

5. STATISTICS

All results presented in the tables of the report were calculated using non-rounded values as per the raw data rounding procedure and may not be exactly reproduced from the individual data presented. Text Table 8 defines the set of comparisons used in the statistical analyses described in this section.

Text Table 8
Statistical Comparisons

Control Group	Treatment Group
1	2, 3, 4

The raw data were tabulated within each time interval, and the mean and standard deviation were calculated for each endpoint by sex and group. For each endpoint, treatment groups were compared to the control group using the analysis outlined in Text Table 9.

Text Table 9
Statistical Analysis

Endpoints	Type of Analysis
Body Weights<br>Body Weight Gains<br>Food Consumption<br>Hematology<br>Coagulation<br>Clinical Chemistry<br>Organ Weights (Absolute Weights and Relative to Body and Brain Weights)	Group Pair-wise Comparisons (General ANOVA)

5.1. Parametric/Non-parametric Comparisons

Included below are the details of the statistical routines that were applied to the data, dependent on the data specific assumptions outlined as part of the routine. The actual analysis performed for each endpoint and collection interval is included in the summary tables. The experimental unit for the analysis of food consumption was cage, while for all other endpoints the experimental unit was the individual animal. Food consumption was calculated as described in the Testing Facility SOP.

If the control group had a sample size less than 3, no inferential statistics were calculated. If a particular endpoint and/or parameter within a given collection interval had the same value across all experimental units, no inferential statistics were calculated.

Otherwise, for endpoints and/or parameters where all groups with sample sizes of 3 or greater were included, Levene's test was be used to assess the homogeneity of group variances (Milliken and Johnson, 1992; Royston, 1992).

The groups were compared using an overall one-way ANOVA F-test if Levene's test was not significant or the Kruskal-Wallis test if it was significant. If the overall F-test or Kruskal-Wallis test was found to be significant, then pairwise comparisons were conducted using Dunnett's or Dunn's test, respectively.

Results of all pair-wise comparisons are reported at the 0.05 and 0.01 significance levels. All endpoints were analyzed using two-tailed tests.

6. COMPUTERIZED SYSTEMS

Critical computerized systems used in the study are listed below or presented in the appropriate Phase Report. All computerized systems used in the conduct of this study have been validated; when a particular system has not satisfied all requirements, appropriate administrative and procedural controls were implemented to assure the quality and integrity of data.

Text Table 10
Critical Computerized Systems

System Name	Version No.	Description of Data Collected and/or Analyzed
DocuSign™	Part 11	Collection of 21 CFR Part 11 compliant signature
M-Files®	21.1	Reporting and collection of 21 CFR Part 11 compliant signature
Logbook	5.3	Electronic notebook and data collection system for veterinary communications, observations, and treatments.
ExyLIMS	3.0	A comprehensive laboratory information management system used to manage data, including but not limited to: instrumentation, test articles, standards, and samples.
Deviation Information Library	2.1	Deviations
Provantis®	10.2	In-life; clinical pathology; postmortem; Test Material receipt, accountability and/or formulation activities. Dispense: Test Material receipt, accountability and/or formulation activities.
Einfotree	7.6 or higher	Excel module for data collection with 21 CFR Part 11 compliance requirements, security, audit trail and electronic signatures.
In-house reporting software (using SAS)	(SAS 9.4)	In-life; clinical pathology; postmortem
Siemens Environmental Monitoring	3.11	Environmental monitoring, alarming, and reporting applications.
Niagara Framework® Software System	2.3	

7. RETENTION AND DISPOSITION OF RECORDS, SAMPLES, AND SPECIMENS

All study-specific raw data, documentation, protocol, samples, specimens, and final reports from this study were archived at the Testing Facility in Mattawan, MI by no later than the date of final report issue unless otherwise specified in the protocol. At least 1 year after issue of the draft report, the Sponsor will be contacted.

Electronic data generated by the Testing Facility were archived as noted above, except that the data collected using Provantis and Dispense, reporting files stored on M-Files®, and deviations were archived at the Charles River Laboratories facility location in Wilmington, MA.

All records, retained samples and specimens, and reports generated from phases or segments performed by subcontractors for biodistribution and pathology were returned to the Testing Facility in Mattawan, MI for archiving.

Disposition of residual/retained analytical samples was as described in the table below.

Text Table 11
Disposition of Residual/Retained Samples

Sample Type	Disposition
Residual dosing formulations	Return to Sponsor

8. RESULTS

8.1. Surgical Procedures

The surgical procedure was well tolerated by all animals.

8.2. Mortality

(Appendix 3)

A single animal, Group 3 male Animal 3008 (dosed at 0.83E12 vg), from the Day 91 cohort, was euthanized in extremis on Study Day 16. This animal was noted to have splayed left and right hindlimbs and an abnormal gait on Study Day 14. The cause of moribundity for this animal was determined to be severe degeneration/necrosis (resulting in loss) of the gray matter of the thoracic spinal cord with marked nerve fiber degeneration of the associated white matter.

8.3. Veterinary Treatments

(Appendix 5)

On occasion, veterinary observations were conducted during the course of the study for health monitoring purposes. In general, veterinary observations were similar to, and supported by, the detailed clinical observations.

8.4. In-life Examinations

8.4.1. Detailed Clinical Observations

(Table 1 and Appendix 4)

There were no significant AAV9/SURF1-related adverse clinical observations with the exception of hindlimb findings observed in a single male and female in Group 3 (0.83E12 vg). Other Clinical findings that were observed were considered to be incidental and not test article-related. These observations were generally sporadic, low in frequency, lacked dose-dependency, were considered injury or blood collection-related, occurred at similar frequency to controls, and/or were considered incidental as common findings in rats of this strain/age.

During 3, three cages of female animals (1 cage each of 0 vg, 0.28E12 vg, and 2.49E12 vg) were observed with loss of skin elasticity and/or hunched posture. Following examination, it was determined that the sippers in the cages were flowing too slowly which was immediately corrected. The observations were consistent with dehydration and were consistent with bodyweight losses and food consumption decreases during this period.

On study day 14, Animal No. 3008 (0.83E12 vg, male) was observed with splayed hindlimbs and abnormal gait. The animal was submitted for veterinary consultation and monitored daily. On Day 16, the animal was observed additionally with a severe decreased righting reflex and rigid body appearance. As the condition was not improving the animal was euthanized in extremis on Day 16. On study day 14, Animal No. 3525 (0.83E12 vg, female) was observed with limited usage of both hindlimbs and yellow fur staining which persisted until the animal was submitted for scheduled necropsy on Day 29.

8.4.2. Body Weight and Body Weight Gains

(Body weight - Figure 1, Table 2, and Appendix 6)

(Body weight gains - Figure 2, Table 3, and Appendix 7)

There was no AAV9/SURF1-related effect on body weight or body weight change. Occasional differences from controls were noted but were of low magnitude and considered to reflect normal biological variation or were associated with removal of animals following the Day 29 necropsy.

8.4.3. Caged Food Consumption

(Figure 3, Table 4, and Appendix 8)

There was no AAV9/SURF1-related effect on food consumption. Food consumption was similar among treatment groups and controls. Occasional differences from controls were noted but were of low magnitude and considered to reflect normal biological variation.

8.5. Clinical Pathology

(Appendix 9)

For the purpose of this report, treated animals' values were compared control values.

1.1. Unscheduled Collections

Due to poor clinical condition, Animal No. 3008 (0.83E12 vg, Male) had a collection for clinical pathology and subsequently euthanized on Day 16. There was a mildly lower WBC attributed by lower neutrophils, lymphocytes and monocytes compared to control and cohort animals. There was mildly higher total red cell mass (RBC, Hb, HCT) with moderately lower decreased reticulocytes. These changes suggest a diminished hematopoiesis with evidence of subclinical dehydration. Changes in clinical chemistry included minimally higher alanine aminotransferase (ALT), urea nitrogen, total protein, albumin and globulin. All indicate the presence of subclinical dehydration. All changes are considered not directly related to test material administration.

8.6. Hematology

On Day 29, females at 2.49E12 vg (Group 4) had minimally higher WBC attributed by higher lymphocytes.

All other fluctuations among individual and mean values were considered sporadic, consistent with biologic variation and/or negligible in magnitude, and not related to test material administration.

8.7. Coagulation

There were no AAV9/SURF1-related effects among coagulation parameters in either sex at any dose level. All fluctuations among individual and mean values, regardless of statistical significance, were considered sporadic, consistent with biologic variation and/or negligible in magnitude, and not related to test material administration.

8.8. Clinical Chemistry

There were no AAV9/SURF1-related effects among chemistry parameters in either sex at any dose level. All fluctuations among individual and mean values were considered sporadic, consistent with biologic variation and/or negligible in magnitude, and not related to test material administration.

8.9. Microscopic Evaluations

(Appendix 10)

8.9.1. Animal Mortality

References to figures in the following sections relate to images in Data Section VII.

A single animal, Group 3 male Animal 3008 (dosed at 0.83E12 vg), from the Day 91 cohort, was euthanized in extremis on Study Day 16. This animal was noted to have splayed left and right hindlimbs and an abnormal gait on Study Day 14. The cause of moribundity for this animal was determined to be severe degeneration/necrosis (resulting in loss) of the gray matter of the thoracic spinal cord with marked nerve fiber degeneration of the associated white matter, as shown in Figures 1 (transverse section) and 2 (longitudinal section). These thoracic spinal cord changes were bilateral predominantly within the lateral and ventral horns, with sparing of the dorsal horns, and diffusely affected the entirety of the longitudinal spinal cord section. The gray matter degeneration/necrosis was associated with marked increases in glial cells and mild perivascular mononuclear cell infiltrates. Similar, but less severe findings of gray matter degeneration/necrosis (resulting in vacuolar change and/or loss), white matter nerve fiber degeneration, increases in glial cells and perivascular mononuclear cell infiltrates were present in the lumbar segment of the spinal cord as shown in Figure 3. The cervical spinal cord had only minimal increases in glial cells and minimal perivascular mononuclear cell infiltrates. Additional microscopic changes associated with/secondary to the findings in the spinal cord were nerve fiber degeneration and increases in Schwann cells of the ventral nerve root of the thoracic and lumbar dorsal root ganglion as shown in Figure 4, as well as mild nerve fiber degeneration of the sciatic and tibial nerves. The spinal cord changes were similar to those seen in a Day 8 and Day 29 terminal sacrifice animal, as discussed in Section 8.8.3 below.

8.9.2. Macroscopic Observations

There were no macroscopic observations in terminal sacrifice animals at Days 8, 29 or 91 that were considered to be related to the intrathecal administration of AAV9/SURF1.

8.9.3. Microscopic Observations

References to figures in the following sections relate to images in Data Section VII.

8.9.3.1. Terminal Sacrifice Animals

Test Article Related Findings Day 8

Day 8 female Animal 3512 (0.83E12 vg) had mild degeneration/necrosis of the gray matter of the thoracic spinal cord with minimal nerve fiber degeneration of the associated white matter, as shown in Figures 5 (transverse section) and 6 (longitudinal section). The gray matter degeneration/necrosis in this animal was characterized by cellular swelling and hypereosinophilia. These thoracic spinal cord changes were predominantly just adjacent to the central canal. The gray matter degeneration/necrosis was associated with mild increases in glial cells and minimal perivascular mononuclear cell infiltrates.

There was a single observation of minimal focal mononuclear cell infiltrates in the lumber DRG in a Day 8 male (Animal 2014) dosed at 0.28E12 vg.

Day 8 males and females at all doses of AAV9/SURF1 had increased incidence of minimal mixed cell infiltrates within the liver. In addition, small numbers of males (Animals 3013, 4011, and 4012) dosed at 0.83E12 vg and 2.49E12 vg had minimal to mild single cell necrosis, and a single male (Animal 4011) dosed at 2.49E12 vg had increased mitotic figures. Small numbers of

males (Animals 2011, 3013, and 4011) at all doses AAV9/SURF1 and a single female (Animal 4513) dosed at 2.49E12 vg had minimal hypertrophy/hyperplasia of Kupffer cells.

Nerve fiber degeneration of minimal severity within the sciatic and tibial nerves was considered to be of equivocal incidence/severity in Day 8 animals with most observations of minimal severity affecting three or less nerve fibers, and mild severity present only in a single control male (Animal 1011).

Test Article-Related Findings Day 29

Day 29 female Animal 3525 (0.83E12 vg) had severe degeneration/necrosis (resulting in loss) of the gray matter of the thoracic spinal cord, predominantly of the lateral and ventral horns, with marked nerve fiber degeneration of the associated white matter, as shown in Figures 7 (transverse section) and 8 (longitudinal section). These thoracic spinal cord changes were bilateral predominantly within the lateral and ventral horns and diffusely affected the entirety of the longitudinal spinal cord section. The gray matter degeneration/necrosis was associated with mild increases in glial cells and minimal perivascular mononuclear cell infiltrates. Similar, but less severe findings of gray matter degeneration/necrosis (resulting in vacuolar change and/or loss), white matter nerve fiber degeneration, increases in glial cells and perivascular mononuclear cell infiltrates were present in the lumbar segment of the spinal cord as shown in Figures 9 (transverse section) and 10 (longitudinal section). The cervical spinal cord had only minimal increases in glial cells and minimal perivascular mononuclear cell infiltrates. Moderate nerve fiber degeneration of the ventral lumbar spinal root, mild nerve fiber degeneration of the inferior cerebellar peduncle of the medulla oblongata, and minimal nerve fiber degeneration within the pons was considered associated with/secondary to the findings in the spinal cord.

Additionally, Day 29 males and females at all doses of AAV9/SURF1 had increased incidence and/or severity of nerve fiber degeneration of the white matter of the thoracic and/or lumbar spinal cords. The degeneration was often present in the dorsal white matter, when the location could be identified on the transverse sections. Nerve fiber degeneration of the spinal cord white matter was diagnosed when there was fragmentation, swelling or hypereosinophilia of axons, dilation of myelin sheaths, and/or presence of digestion chambers (phagocytic cells within dilated spaces).

There were infrequent observations within the lumbar dorsal root ganglion (DRG) of males in Day 29 animals administered AAV9/SURF1. Minimal neuronal degeneration/necrosis was present in a single 0.28E12 vg male (Animal 2025) and a single 2.49E12 vg male (Animal 4024) and involved only one or two neuronal cell bodies. Neuronal degeneration/necrosis was characterized by cytoplasmic hypereosinophilia. Minimal mononuclear cell infiltrates were present in the lumbar DRG (Figure 11) in at least one male at all doses of AAV9/SURF1 and in at least one female dosed at least 0.83E12 vg of AAV9/SURF1. A single 0.83E12 vg female, Animal 3523, had minimal multifocal increases in glial cells forming small discrete nodules (Figure 12). In addition, there was also increased incidence and/or severity of nerve fiber degeneration of the lumbar dorsal spinal nerve root in females dosed at least 0.83E12 vg and males dosed at 2.49E12 vg of AAV9/SURF1.

A single Day 29 male (Animal 3023) dosed at 0.83E12 vg had mild nerve fiber degeneration of spinal nerves of the lumbar spinal cord.

Day 29 males and females at all doses of AAV9/SURF1 had increased incidence of minimal to moderate nerve fiber degeneration of the sciatic nerve and/or tibial nerve.

Day 29 males and females at all doses of AAV9/SURF1 had increased incidence and/or severity of myocardial degeneration/necrosis and mononuclear cell infiltrates of the heart, with no dose-dependent trend. There was also increased incidence of fibrosis of the heart at Day 29 at all doses of AAV9/SURF1, except for males dosed at 2.49E12 vg. Representative images of these heart findings are depicted at low (Figure 13) and higher magnification (Figure 14).

Day 29 males and females at all doses of AAV9/SURF1 had increased incidence and/or severity of mixed cell infiltrates within the liver. In addition, males at 2.49E12 vg and a single female (Animal 3523) dosed at 0.83E12 vg had minimal single cell necrosis. A representative image of these liver findings is depicted in Figure 15.

Test Article-Related Findings Day 91

Similar to what was observed at Day 29, there were infrequent observations within the lumbar DRG in Day 91 animals administered AAV9/SURF1. Mononuclear cell infiltrates were present at minimal severity in the lumbar DRG in at least one male at all doses of AAV9/SURF1 and in females dosed at least 2.49E12 vg, and a single male (Animal 2007) dosed at 0.28E12 vg had mild mononuclear cell infiltrates of the lumbar DRG. In addition, there was increased incidence and/or severity of nerve fiber degeneration of the lumbar dorsal spinal nerve root in males at 0.28E12 vg and males and females dosed at 2.49E12 vg.

Mild nerve fiber degeneration of the spinal nerves of either the thoracic or lumbar spinal cords was present in a single Day 91 male dosed at 0.28E12 vg (Animal 2007) and 0.83E12 vg (Animal 3010) and a single female dosed at 0.83E12 vg (Animal 3508) and 2.49E12 vg (Animal 4507) of AAV9/SURF1. A single female (Animal 3509) dosed at 0.83E12 vg had marked nerve fiber degeneration of the spinal nerves of the lumbar spinal cord.

Similar to what was observed at Day 29, Day 91 males and females at all doses AAV9/SURF1 had increased incidence of minimal to mild nerve fiber degeneration of the sciatic nerve and/or tibial nerve.

Similar to what was observed at Day 29, Day 91 males and females at all doses of AAV9/SURF1 had increased incidence and/or severity of myocardial degeneration/necrosis and mononuclear cell infiltrates of the heart, with no clear dose-dependent trend. There was also increased incidence of fibrosis of the heart at Day 91 in males dosed at 0.28E12 vg and 2.49E12 vg and females dosed at 0.83E12 vg.

There was a very slight increase in incidence and/or severity of mixed cell infiltrates of the liver in Day 91 males at all doses of AAV9/SURF1 and females dosed at 2.49E12 vg.

Interpretation of the association of nerve fiber degeneration of the white matter of the cervical, thoracic and/or lumbar spinal cord in Day 91 animals was complicated by the inability to identify the white matter tract affected on the longitudinal spinal cord sections. There was an increase in incidence and severity of white matter degeneration of the thoracic spinal cord in AAV9/SURF1 treated females dosed at least 0.83E12 vg . The incidence of minimal white matter degeneration of the cervical and lumbar spinal cord and of the thoracic spinal cord in other AAV9/SURF1 treatment groups was considered equivocal.

Procedure-Related Findings

Within a single Group 2 Day 91 female, Animal 2506, there was an increase in glial cells that was locally extensive and oriented perpendicular to the long axis of the lumbar spinal cord, as shown in Figure 16. In addition to increased glial cells, there were a small number of macrophages with intracytoplasmic accumulation of golden-brown pigment consistent with hemosiderin. These findings were considered consistent with a needle tract and were considered related to the intrathecal administration procedure.

Minimal to mild mononuclear cell infiltrates were present within multiple locations of the central nervous system in small numbers of animals administered AAV9/SURF1 at Day 8, Day 29, and/or Day 91 including the meninges of the brain, the meninges of varying spinal cord segments, and within the pineal gland. This finding was not present in concurrent study control animals at any time point and was thought most likely to represent a local reaction to the presence of a foreign protein within the cerebrospinal fluid.

Non-Test Article-Related Findings

Minimal focal mononuclear cell infiltrates were present in the epineurium of the spinal nerve root just adjacent to the DRG. These infiltrates were most often seen in lumbar DRG but were also infrequently present in cervical DRG and thoracic DRG. These infiltrates were sometimes bilateral, sometimes unilateral, and as they were seen in controls at Day 29 and Day 91, the minimal mononuclear cell infiltrates of the epineurium were considered to be spontaneous and unrelated to the administration of AAV9/SURF1. Variation in incidence was thought to be due to the inconsistency of the presence of the epineurium at the spinal nerve root/DRG junction in the sections evaluated.

Nerve fiber degeneration of the ventral spinal nerve root was present at minimal severity in a control Day 8 male (Animal 1013) in the thoracic or lumbar spinal nerve roots and also in a control Day 29 female (Animal 1525) in the thoracic spinal nerve root. As this finding was present in controls and typically affected three or less nerve fibers, minimal ventral spinal nerve root nerve fiber degeneration was considered spontaneous and unrelated to administration of AAV9/SURF1.

Nerve fiber degeneration of the spinal nerves was most often seen in lumbar spinal cord sections but rarely also in thoracic spinal cord sections. At minimal severity, this finding was considered to be spontaneous as it was seen in a Day 8 and Day 91 control male in either the thoracic or lumbar spinal cords. Variation in incidence was thought to be due, at least in part, to the inconsistency of the presence of spinal nerves within the spinal cord sections.

Minimal nerve fiber degeneration of the white matter, specifically pyramidal tracts, of the medulla oblongata was present in a small number of animals in multiple treatment groups, including controls, and thus was considered spontaneous and unrelated to administration of AAV9/SURF1.

Additional microscopic findings observed in Day 8, Day 29, or Day 91 animals, not described above, were considered incidental, consistent with those seen in laboratory rats of this strain and age, and/or exhibited no treatment-related trend of incidence, and therefore were considered unrelated to administration of AAV9/SURF1.

9. CONCLUSION

The objective of this study was to characterize the toxicity, biodistribution, and gene expression of AAV9/SURF1 for the treatment of SURF1 Leigh Syndrome.

Administration of AAV9/SURF1 was not associated with any bodyweight or food consumption changes that were considered adverse. All animals survived to their scheduled terminal necropsies with 1 exceptions, Animal No. 3008 (0.83E12, male), was euthanized in extremis on Day 16 following no improvement of hind limb impairment.

AAV9/SURF1-related clinical pathology changes included minimally higher WBC attributed by higher lymphocytes on Day 29 in females at 2.49E12 (Group 4). This finding lacked a microscopic correlate.

A single bolus of AAV9/SURF1 at doses of 0.28E12 vg, 0.83E12 vg, or 2.49E12 vg was administered intrathecally into the lumbar cistern in male and female Sprague Dawley rats with scheduled terminations at Days 8, 29, and 91. Early death occurred in a single male dosed with AAV9/SURF1 at 0.83E12 vg; the cause of moribundity was due to procedure/test article-related gray matter degeneration/necrosis predominantly centered on the thoracic spinal cord.
There were no macroscopic observations at Days 8, 29, or 91 that were considered to be associated with the administration of AAV9/SURF1.
At the scheduled terminations, single bolus administration of AAV9/SURF1 resulted in microscopic changes in the white matter of the spinal cord at Days 29 and 91 (increased incidence and/or severity of nerve fiber degeneration); the dorsal root ganglia (neuron degeneration/necrosis, mononuclear cell infiltrates, and/or hypertrophy/hyperplasia in [satellite] glial cells) at Days 8, 29, and 91; the dorsal spinal nerve roots (nerve fiber degeneration) at Days 29 and 91; the spinal nerve roots adjacent to the spinal cord at Days 29 and 91 (nerve fiber degeneration); the tibial and sciatic nerves at Days 29 and 91 (increased incidence and/or severity of nerve fiber degeneration and/or Schwann cell hypertrophy/hyperplasia); the heart at Days 29 and 91 (myocardium degeneration/necrosis, mononuclear cell infiltrates, and/or fibrosis); and the liver at Days 8, 29, and 91 (increased incidence and severity of mixed cell infiltrates, single cell necrosis of hepatocytes, Kupffer cell hypertrophy/hyperplasia, and/or increased mitotic figures).
Microscopic findings of the gray matter of the spinal cord (degeneration/necrosis, increases in glial cells, and/or mononuclear cell infiltrates) in single animals at Day 8 and Day 29 were considered related to the inadvertent administration of the test material likely within the central canal. In addition, single animals at Day 29 and Day 91 had locally extensive spinal cord changes consistent with inadvertent needle stick into the spinal cord. The degeneration/necrosis of the gray matter of the spinal cord (in combination with the likely inadvertent administration of the AAV9/SURF1 test article into the central canal of the spinal cord), the neuronal degeneration/necrosis within the lumbar DRG, and the heart myocardial changes (degeneration/necrosis, mononuclear cell infiltration, and/or fibrosis) at moderate and greater severity were considered adverse. The test article-related microscopic changes did not, in general, exhibit a dose-dependent trend of incidence and/or severity; however, there was timepoint-dependent variability in occurrence/severity of some microscopic findings. Any variation in microscopic findings due to the test article lot administered, Lot 1 or Lot 2, could not be definitively determined.

10. REFERENCES

Guidance for industry, investigators, and reviewers: exploratory IND Studies. *U.S. F.D.A. Center for Drug Evaluation and Research (CDER)*. 2006 Jan.

Milliken GA, Johnson DE. *Analysis of Messy Data: Volume I: Designed Experiments*. London: Chapman and Hall; 1992.

National Research Council. *Guide for the Care and Use of Laboratory Animals*. 8th ed. Washington, DC: National Academies Press; Current edition.

Position statement on the use of animals in research. *NIH Guide* 1993 Feb 26;22(8).

Principles for the utilization and care of vertebrate animals used in testing, research, and training. *Federal Register*. 1985 May 20;50(97).

Royston, JP. Approximating the Shapiro-Wilk W Test for Nonnormality. *Stat Comput*. 1992;2:117-119.

Figure 1

Summary of Group Mean Body Weights

2954-001

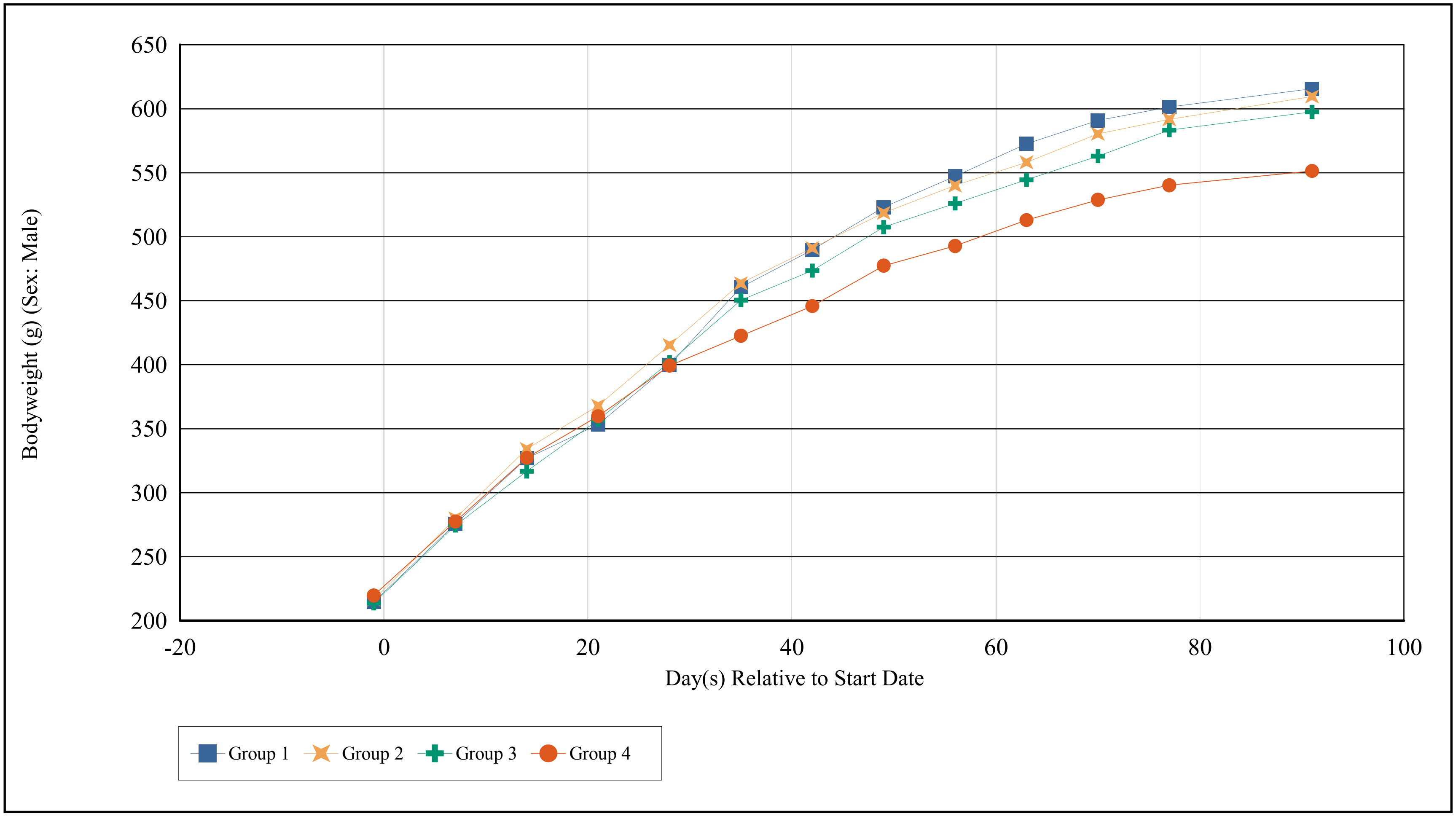

Figure 1

Summary of Group Mean Body Weights

2954-001

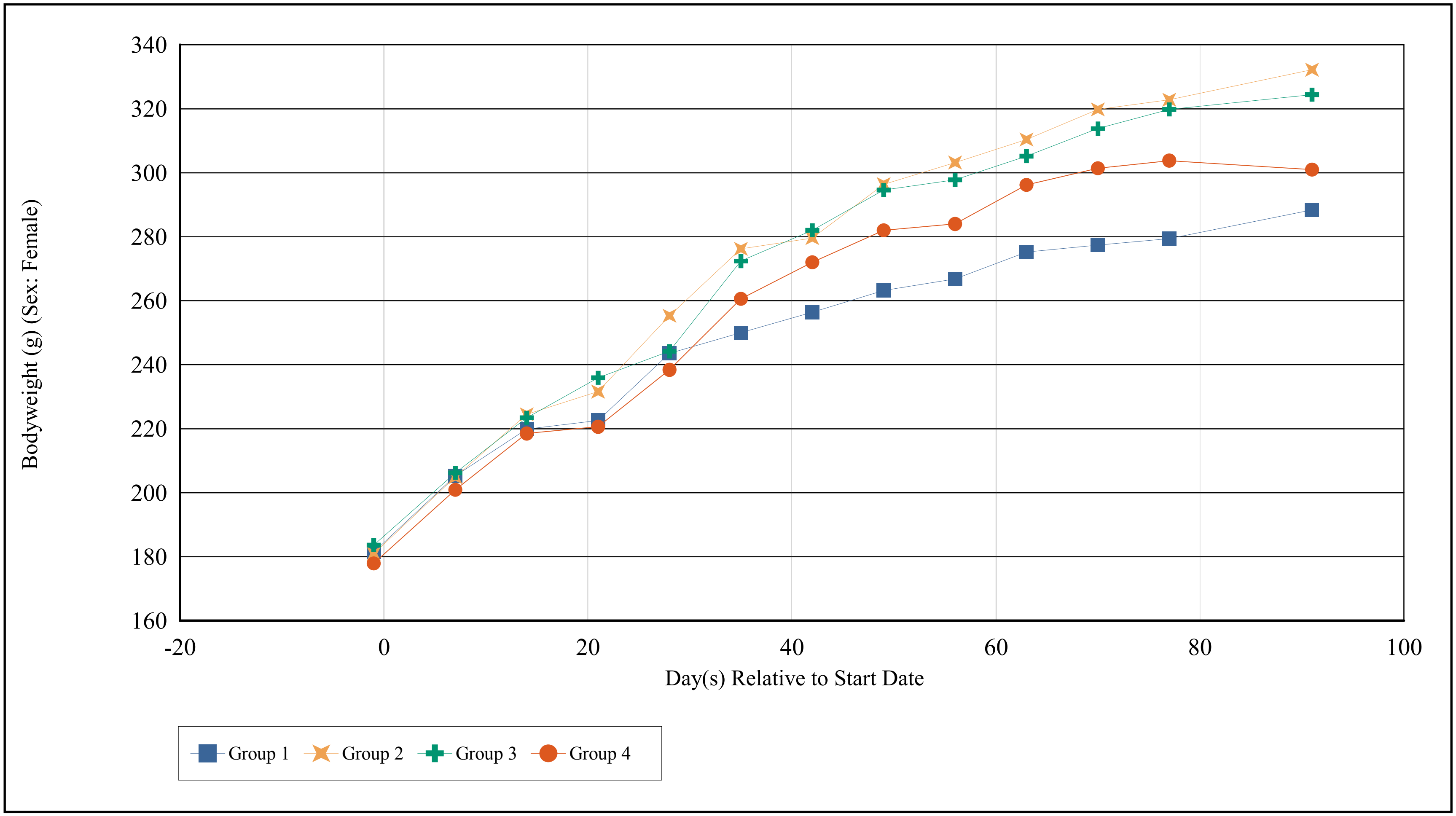

Figure 2

Summary of Group Mean Body Weight Gains (g)

2954-001

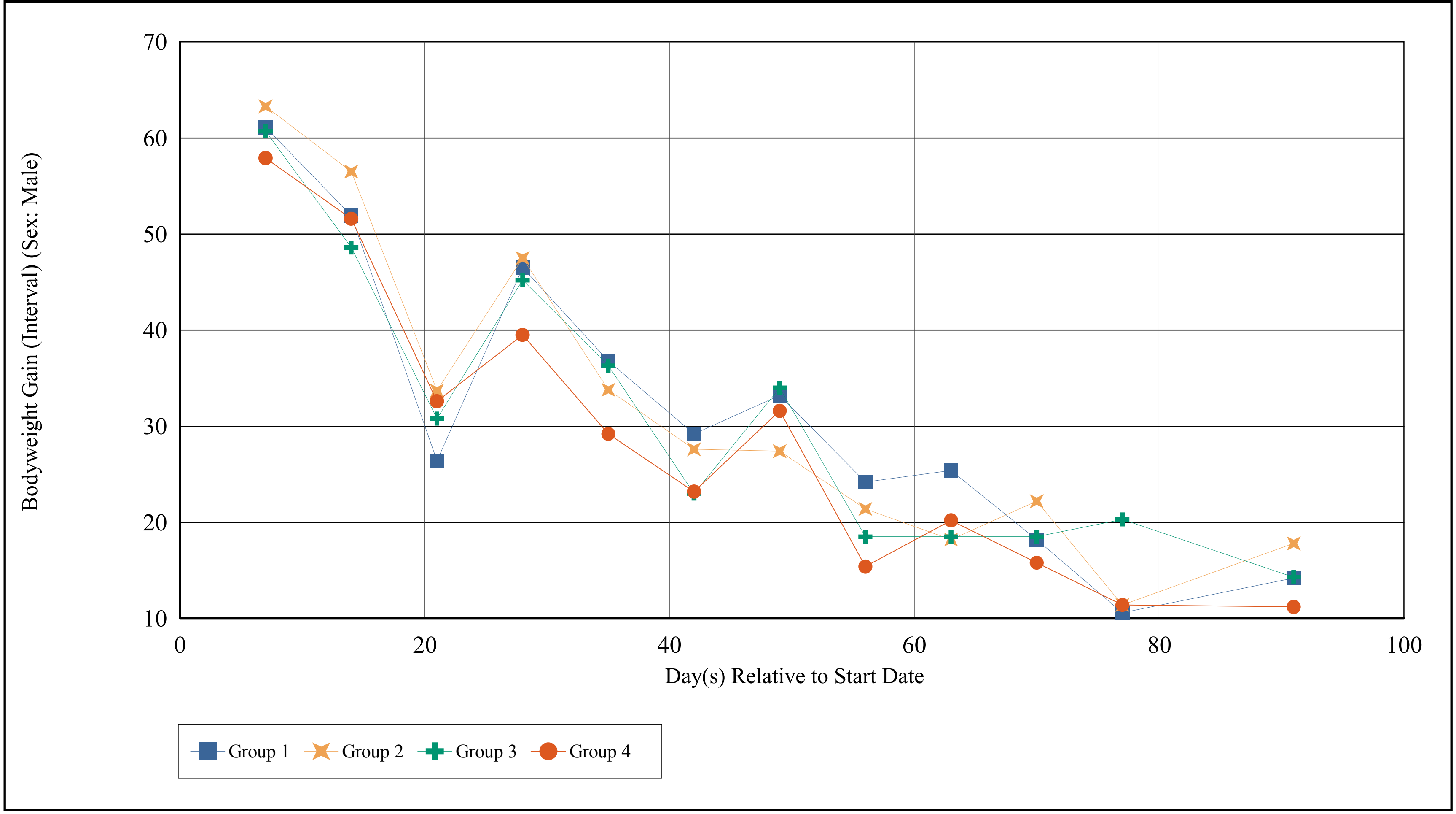

Figure 2

Summary of Group Mean Body Weight Gains (g)

2954-001

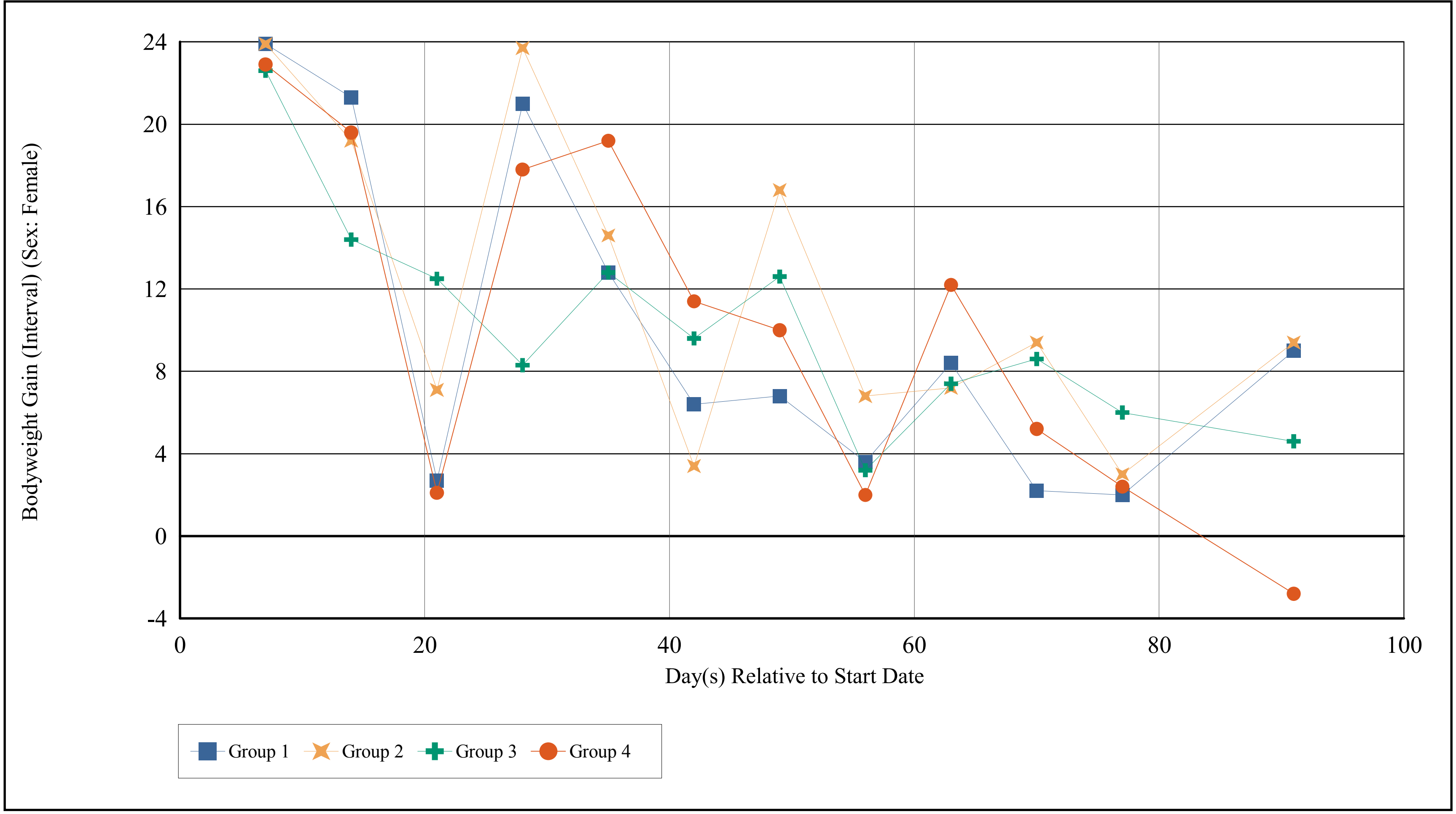

Figure 3

Summary of Group Mean Food Consumption
2954-001

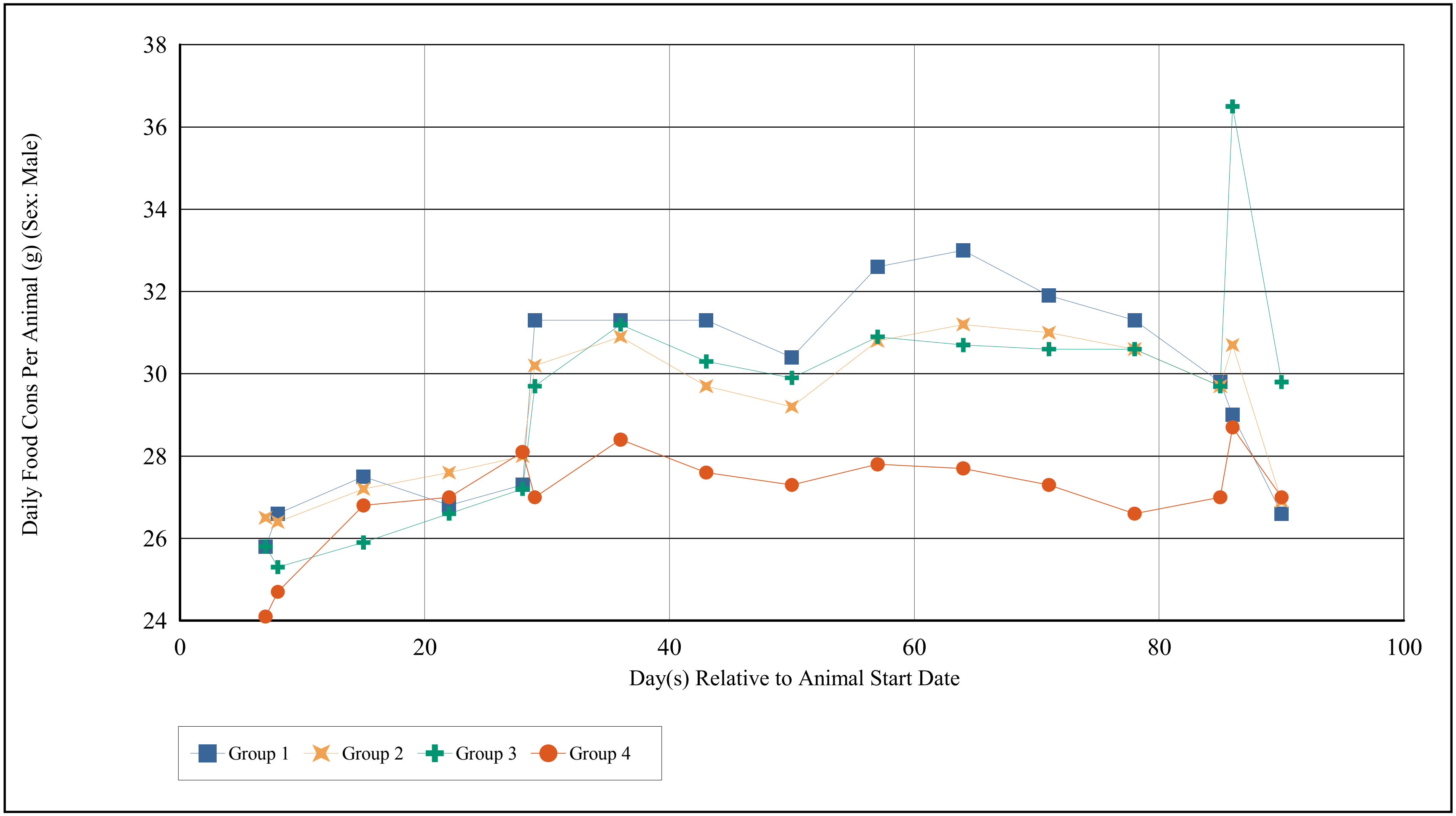

Figure 3

Summary of Group Mean Food Consumption
2954-001

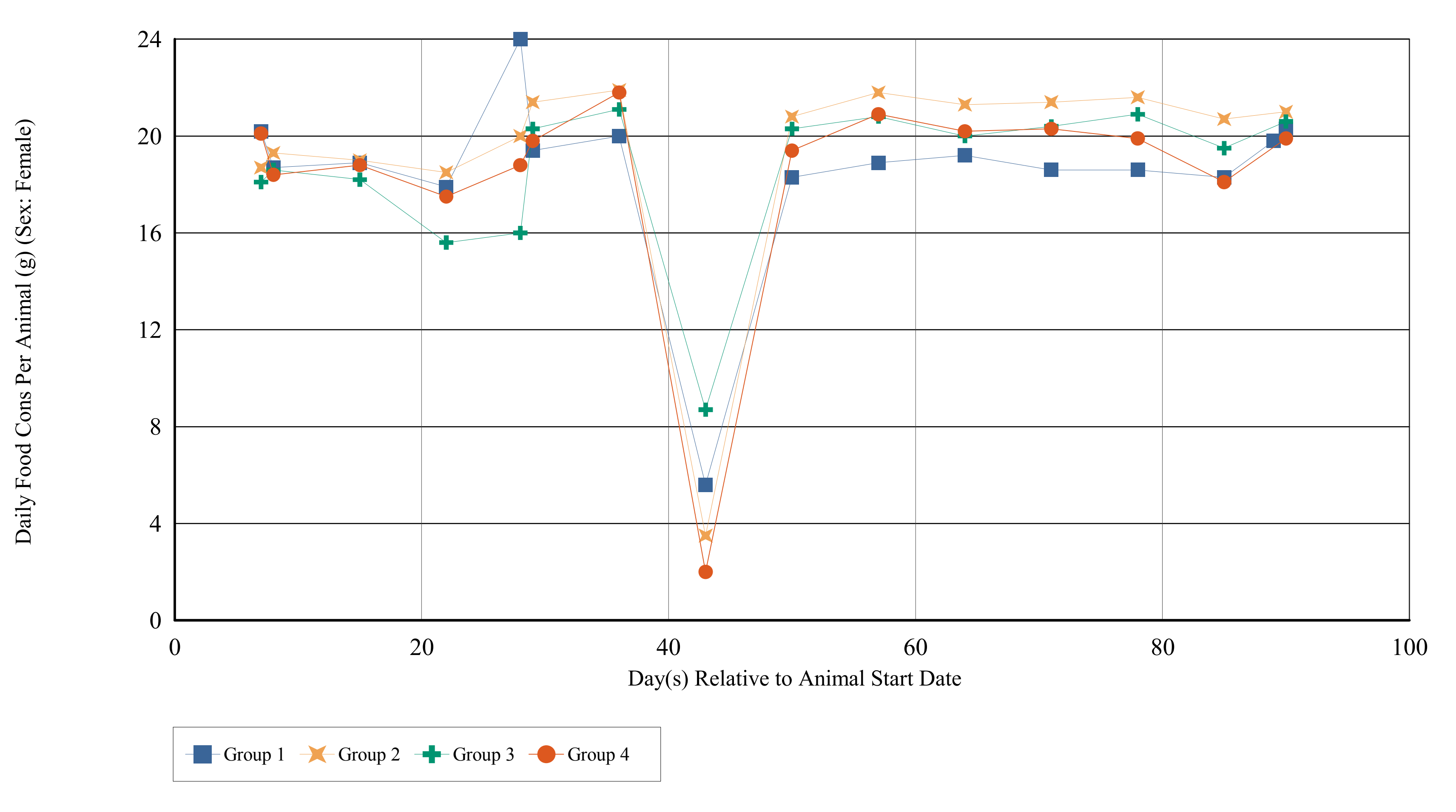

Summary of Clinical Observations

2954-001

Observation Type: All Types	Male			
From Day -1 (Start Date) to 91 (Start Date)	Group 1	Group 2	Group 3	Group 4
**Tail, Bent (PT)**				
Number of Animals Affected	0	0	0	1
Number of Times Recorded	0	0	0	2
% of Affected Animals	0	0	0	7
First to Last seen	-	-	-	-1 - 7
**Fur, Staining, Dorsal Cervical**				
Number of Animals Affected	0	0	2	2
Number of Times Recorded	0	0	7	9
% of Affected Animals	0	0	13	13
First to Last seen	-	-	70 - 91	28 - 84
**Fur, Thin Cover, Dorsal Cervical**				
Number of Animals Affected	1	0	0	0
Number of Times Recorded	1	0	0	0
% of Affected Animals	7	0	0	0
First to Last seen	91 - 91	-	-	-
**Fur, Thin Cover, Face**				
Number of Animals Affected	0	6	4	0
Number of Times Recorded	0	24	7	0
% of Affected Animals	0	40	25	0
First to Last seen	-	14 - 84	21 - 91	-
**Fur, Thin Cover, Forelimb, Left**				
Number of Animals Affected	0	1	0	0
Number of Times Recorded	0	2	0	0
% of Affected Animals	0	7	0	0
First to Last seen	-	77 - 84	-	-
**Fur, Thin Cover, Forelimb, Right**				
Number of Animals Affected	0	1	0	0
Number of Times Recorded	0	1	0	0
% of Affected Animals	0	7	0	0

Summary of Clinical Observations

2954-001

Observation Type: All Types	Male			
From Day -1 (Start Date) to 91 (Start Date)	Group 1	Group 2	Group 3	Group 4
**Fur, Thin Cover, Forelimb, Right (Continued...)**				
First to Last seen	-	84 - 84	-	-
**Skin, Scab, Dorsal Thoracic**				
Number of Animals Affected	1	0	0	0
Number of Times Recorded	1	0	0	0
% of Affected Animals	7	0	0	0
First to Last seen	14 - 14	-	-	-
**Skin, Scab, Face**				
Number of Animals Affected	0	2	2	0
Number of Times Recorded	0	9	2	0
% of Affected Animals	0	13	13	0
First to Last seen	-	28 - 70	28 - 91	-
**Skin, Scab, Muzzle**				
Number of Animals Affected	0	1	0	0
Number of Times Recorded	0	1	0	0
% of Affected Animals	0	7	0	0
First to Last seen	-	84 - 84	-	-
**Splayed Limb, Hindlimb, Left**				
Number of Animals Affected	0	0	1	0
Number of Times Recorded	0	0	1	0
% of Affected Animals	0	0	6	0
First to Last seen	-	-	14 - 14	-
**Splayed Limb, Hindlimb, Right**				
Number of Animals Affected	0	0	1	0
Number of Times Recorded	0	0	1	0
% of Affected Animals	0	0	6	0
First to Last seen	-	-	14 - 14	-
**Eyeball, Abnormal Color, Right**				
Number of Animals Affected	0	1	0	0

Summary of Clinical Observations

2954-001

Observation Type: All Types	Male			
From Day -1 (Start Date) to 91 (Start Date)	Group 1	Group 2	Group 3	Group 4
**Eyeball, Abnormal Color, Right (Continued...)**				
Number of Times Recorded	0	3	0	0
% of Affected Animals	0	7	0	0
First to Last seen	-	70 - 84	-	-
**Discharge, Color, Penis**				
Number of Animals Affected	0	0	1	0
Number of Times Recorded	0	0	1	0
% of Affected Animals	0	0	6	0
First to Last seen	-	-	14 - 14	-
**Low Carriage**				
Number of Animals Affected	0	0	0	1
Number of Times Recorded	0	0	0	1
% of Affected Animals	0	0	0	7
First to Last seen	-	-	-	28 - 28
**Abnormal Gait**				
Number of Animals Affected	0	0	1	0
Number of Times Recorded	0	0	1	0
% of Affected Animals	0	0	6	0
First to Last seen	-	-	14 - 14	-
**Skin, Laceration, Tail**				
Number of Animals Affected	0	0	0	1
Number of Times Recorded	0	0	0	1
% of Affected Animals	0	0	0	7
First to Last seen	-	-	-	1 - 1

Summary of Clinical Observations

2954-001

Observation Type: All Types	Female			
From Day -1 (Start Date) to 91 (Start Date)	Group 1	Group 2	Group 3	Group 4
**Limited Usage, Hindlimb, Left**				
Number of Animals Affected	0	0	1	0
Number of Times Recorded	0	0	3	0
% of Affected Animals	0	0	7	0
First to Last seen	-	-	14 - 28	-
**Limited Usage, Hindlimb, Right**				
Number of Animals Affected	0	0	1	0
Number of Times Recorded	0	0	3	0
% of Affected Animals	0	0	7	0
First to Last seen	-	-	14 - 28	-
**Dehydrated Suspected**				
Number of Animals Affected	3	3	0	2
Number of Times Recorded	3	3	0	2
% of Affected Animals	20	20	0	13
First to Last seen	20 - 21	20 - 21	-	21 - 21
**Breathing, Labored**				
Number of Animals Affected	0	0	1	0
Number of Times Recorded	0	0	1	0
% of Affected Animals	0	0	7	0
First to Last seen	-	-	1 - 1	-
**Hunched Posture**				
Number of Animals Affected	3	0	1	0
Number of Times Recorded	3	0	1	0
% of Affected Animals	20	0	7	0
First to Last seen	20 - 21	-	1 - 1	-
**Fur, Staining, Abdominal**				
Number of Animals Affected	0	0	1	0
Number of Times Recorded	0	0	2	0
% of Affected Animals	0	0	7	0

Summary of Clinical Observations

2954-001

Observation Type: All Types<br>From Day -1 (Start Date) to 91 (Start Date)	Female<br>Group 1	 Group 2	 Group 3	 Group 4
**Fur, Staining, Abdominal (Continued...)**				
First to Last seen	-	-	21 - 28	-
**Fur, Staining, Cranium**				
Number of Animals Affected	1	0	1	1
Number of Times Recorded	1	0	2	2
% of Affected Animals	7	0	7	7
First to Last seen	84 - 84	-	84 - 91	84 - 91
**Fur, Staining, Dorsal Cervical**				
Number of Animals Affected	0	0	2	2
Number of Times Recorded	0	0	4	2
% of Affected Animals	0	0	13	13
First to Last seen	-	-	84 - 91	91 - 91
**Fur, Staining, Face**				
Number of Animals Affected	0	0	2	2
Number of Times Recorded	0	0	9	7
% of Affected Animals	0	0	13	13
First to Last seen	-	-	49 - 91	63 - 91
**Fur, Staining, Urogenital**				
Number of Animals Affected	0	0	1	0
Number of Times Recorded	0	0	2	0
% of Affected Animals	0	0	7	0
First to Last seen	-	-	21 - 28	-
**Fur, Thin Cover, Dorsal Aspect Generalized**				
Number of Animals Affected	0	0	1	0
Number of Times Recorded	0	0	2	0
% of Affected Animals	0	0	7	0
First to Last seen	-	-	84 - 91	-
**Fur, Thin Cover, Dorsal Cervical**				
Number of Animals Affected	0	0	1	0

Summary of Clinical Observations

2954-001

Observation Type: All Types	Female			
From Day -1 (Start Date) to 91 (Start Date)	Group 1	Group 2	Group 3	Group 4
**Fur, Thin Cover, Dorsal Cervical (Continued...)**				
Number of Times Recorded	0	0	1	0
% of Affected Animals	0	0	7	0
First to Last seen	-	-	91 - 91	-
**Fur, Thin Cover, Face**				
Number of Animals Affected	0	0	0	1
Number of Times Recorded	0	0	0	1
% of Affected Animals	0	0	0	7
First to Last seen	-	-	-	84 - 84
**Fur, Thin Cover, Sacral**				
Number of Animals Affected	0	0	1	0
Number of Times Recorded	0	0	3	0
% of Affected Animals	0	0	7	0
First to Last seen	-	-	63 - 77	-
**Skin, Scab, Dorsal Thoracic**				
Number of Animals Affected	0	0	0	1
Number of Times Recorded	0	0	0	2
% of Affected Animals	0	0	0	7
First to Last seen	-	-	-	7 - 14
**Skin, Scab, Face**				
Number of Animals Affected	0	0	0	1
Number of Times Recorded	0	0	0	1
% of Affected Animals	0	0	0	7
First to Last seen	-	-	-	84 - 84
**Nail Missing, Forepaw, Left**				
Number of Animals Affected	1	0	0	0
Number of Times Recorded	1	0	0	0
% of Affected Animals	7	0	0	0
First to Last seen	84 - 84	-	-	-

Summary of Clinical Observations

2954-001

Observation Type: All Types	Female			
From Day -1 (Start Date) to 91 (Start Date)	Group 1	Group 2	Group 3	Group 4
**Swollen, Abdominal**				
Number of Animals Affected	0	0	1	0
Number of Times Recorded	0	0	1	0
% of Affected Animals	0	0	7	0
First to Last seen	-	-	28 - 28	-
**Swollen, Axillary, Left**				
Number of Animals Affected	0	0	1	0
Number of Times Recorded	0	0	3	0
% of Affected Animals	0	0	7	0
First to Last seen	-	-	7 - 21	-
**Swollen, Axillary, Right**				
Number of Animals Affected	0	0	1	0
Number of Times Recorded	0	0	3	0
% of Affected Animals	0	0	7	0
First to Last seen	-	-	7 - 21	-
**Swollen, Inguinal, Left**				
Number of Animals Affected	0	0	1	0
Number of Times Recorded	0	0	1	0
% of Affected Animals	0	0	7	0
First to Last seen	-	-	21 - 21	-
**Swollen, Inguinal, Right**				
Number of Animals Affected	0	0	1	0
Number of Times Recorded	0	0	1	0
% of Affected Animals	0	0	7	0
First to Last seen	-	-	21 - 21	-
**Swollen, Urogenital**				
Number of Animals Affected	0	0	1	0
Number of Times Recorded	0	0	1	0
% of Affected Animals	0	0	7	0

Summary of Clinical Observations

2954-001

Observation Type: All Types	Female			
From Day -1 (Start Date) to 91 (Start Date)	Group 1	Group 2	Group 3	Group 4
**Swollen, Urogenital (Continued...)**				
First to Last seen	-	-	28 - 28	-
**Swollen, Ventral Cervical**				
Number of Animals Affected	0	0	1	0
Number of Times Recorded	0	0	1	0
% of Affected Animals	0	0	7	0
First to Last seen	-	-	21 - 21	-
**Activity Decreased**				
Number of Animals Affected	0	0	1	0
Number of Times Recorded	0	0	1	0
% of Affected Animals	0	0	7	0
First to Last seen	-	-	1 - 1	-

Table 2

Summary of Body Weights

2954-001
Bodyweight (g)

Sex: Male		Day(s) Relative to Start Date						
		-1 [G]	7 [G]	14 [G1]	21 [G1]	28 [G1]	35 [G1]	42 [G1]
Group 1	Mean	214.5	275.6	326.9	353.3	399.8	460.6	489.8
	SD	12.0	11.6	14.4	34.7	29.5	13.9	14.2
	N	15	15	10	10	10	5	5
Group 2	Mean	216.5	279.9	334.2	367.9	415.4	463.6	491.2
	SD	16.2	16.6	20.5	40.7	31.2	32.1	33.1
	N	15	15	10	10	10	5	5
Group 3	Mean	213.6	274.3	316.7	356.8	402.0	450.5	473.5
	SD	24.6	28.6	35.8	30.6	31.1	20.4	26.3
	N	16	16	10	9	9	4	4
Group 4	Mean	219.7	277.5	327.2	359.8	399.3	422.6	445.8*
	SD	22.1	20.4	23.5	28.8	26.2	25.7	26.4
	N	15	15	10	10	10	5	5

[G] - Kruskal-Wallis & Dunn
[G1] - Anova & Dunnett: * = $p \leq 0.05$

Table 2

Summary of Body Weights

2954-001

Bodyweight (g)

Sex: Male		Day(s) Relative to Start Date					
		49	56	63	70	77	91
Group 1	Mean	523.0	547.2	572.6	590.8	601.4	615.6
	SD	24.0	27.8	37.2	37.8	40.3	44.0
	N	5	5	5	5	5	5
Group 2	Mean	518.6	540.0	558.2	580.4	591.8	609.6
	SD	37.6	38.2	39.5	42.8	36.8	38.7
	N	5	5	5	5	5	5
Group 3	Mean	507.5	526.0	544.5	563.0	583.3	597.5
	SD	24.9	29.4	29.9	32.3	36.0	39.8
	N	4	4	4	4	4	4
Group 4	Mean	477.4	492.8	513.0	528.8	540.2	551.4
	SD	32.4	30.0	32.9	33.9	34.9	34.4
	N	5	5	5	5	5	5

Anova & Dunnett

Table 2

Summary of Body Weights

2954-001
Bodyweight (g)

Sex: Female		Day(s) Relative to Start Date						
		-1 [G]	7 [G]	14 [G1]	21 [G1]	28 [G1]	35 [G1]	42 [G1]
Group 1	Mean	181.4	205.3	219.9	222.6	243.6	250.0	256.4
	SD	21.2	23.8	21.5	27.8	23.9	30.3	31.6
	N	15	15	10	10	10	5	5
Group 2	Mean	180.9	204.9	224.5	231.6	255.3	276.2	279.6
	SD	13.5	13.7	13.0	27.8	15.2	24.1	23.6
	N	15	15	10	10	10	5	5
Group 3	Mean	183.6	206.2	223.4	235.9	244.2	272.4	282.0
	SD	9.3	11.6	16.3	19.0	20.7	17.7	19.5
	N	15	15	10	10	10	5	5
Group 4	Mean	177.9	200.9	218.5	220.6	238.4	260.6	272.0
	SD	14.5	12.9	15.0	21.7	15.0	18.8	21.7
	N	15	15	10	10	10	5	5

[G] - Kruskal-Wallis & Dunn
[G1] - Anova & Dunnett

Table 2

Summary of Body Weights

2954-001

Bodyweight (g)

Sex: Female		Day(s) Relative to Start Date					
		49	56	63	70	77	91
Group 1	Mean	263.2	266.8	275.2	277.4	279.4	288.4
	SD	33.4	32.8	32.9	34.5	36.6	32.9
	N	5	5	5	5	5	5
Group 2	Mean	296.4	303.2	310.4	319.8	322.8	332.2
	SD	20.7	20.4	19.4	23.6	16.8	20.8
	N	5	5	5	5	5	5
Group 3	Mean	294.6	297.8	305.2	313.8	319.8	324.4
	SD	23.0	26.5	26.7	27.0	28.2	36.7
	N	5	5	5	5	5	5
Group 4	Mean	282.0	284.0	296.2	301.4	303.8	301.0
	SD	21.9	20.4	20.2	24.4	23.5	18.5
	N	5	5	5	5	5	5

Anova & Dunnett

Table 3

Summary of Body Weight Gains (g)

2954-001
Bodyweight Gain (Interval)

Sex: Male		Day(s) Relative to Start Date						
		-1 → 7 [G]	7 → 14 [G]	14 → 21 [G1]	21 → 28 [G]	28 → 35 [G]	35 → 42 [G]	42 → 49 [G]
Group 1	Mean	61.1	51.9	26.4	46.5	36.8	29.2	33.2
	SD	9.3	11.0	25.0	17.1	5.0	2.5	10.1
	N	15	10	10	10	5	5	5
Group 2	Mean	63.3	56.5	33.7	47.5	33.8	27.6	27.4
	SD	6.5	11.6	24.8	16.8	8.3	6.3	7.7
	N	15	10	10	10	5	5	5
Group 3	Mean	60.7	48.6	30.8	45.2	36.3	23.0	34.0
	SD	6.4	17.2	15.4	11.1	6.6	7.3	5.7
	N	16	10	9	9	4	4	4
Group 4	Mean	57.9	51.6	32.6	39.5	29.2	23.2	31.6
	SD	8.2	9.5	7.6	9.7	5.8	5.2	6.3
	N	15	10	10	10	5	5	5

[G] - Anova & Dunnett
[G1] - Kruskal-Wallis & Dunn

Table 3

Summary of Body Weight Gains (g)

2954-001

Bodyweight Gain (Interval)

Sex: Male		Day(s) Relative to Start Date				
		49 → 56	56 → 63	63 → 70	70 → 77	77 → 91
Group 1	Mean	24.2	25.4	18.2	10.6	14.2
	SD	9.2	12.2	5.6	11.1	4.7
	N	5	5	5	5	5
Group 2	Mean	21.4	18.2	22.2	11.4	17.8
	SD	6.9	5.8	5.8	8.4	5.0
	N	5	5	5	5	5
Group 3	Mean	18.5	18.5	18.5	20.3	14.3
	SD	6.5	4.0	5.3	5.3	4.6
	N	4	4	4	4	4
Group 4	Mean	15.4	20.2	15.8	11.4	11.2
	SD	6.8	5.0	5.4	6.5	3.1
	N	5	5	5	5	5

Anova & Dunnett

Table 3

Summary of Body Weight Gains (g)

2954-001
Bodyweight Gain (Interval)

Sex: Female		Day(s) Relative to Start Date						
		-1 → 7 [G]	7 → 14 [G]	14 → 21 [G1]	21 → 28 [G]	28 → 35 [G]	35 → 42 [G]	42 → 49 [G]
Group 1	Mean	23.9	21.3	2.7	21.0	12.8	6.4	6.8
	SD	6.2	4.8	22.0	18.2	6.4	3.5	5.6
	N	15	10	10	10	5	5	5
Group 2	Mean	23.9	19.2	7.1	23.7	14.6	3.4	16.8*
	SD	7.2	9.3	24.8	21.9	9.1	5.4	5.2
	N	15	10	10	10	5	5	5
Group 3	Mean	22.6	14.4	12.5	8.3	12.8	9.6	12.6
	SD	7.5	10.4	6.0	9.1	7.2	5.8	4.9
	N	15	10	10	10	5	5	5
Group 4	Mean	22.9	19.6	2.1	17.8	19.2	11.4	10.0
	SD	9.3	5.6	22.7	18.7	3.0	3.2	2.5
	N	15	10	10	10	5	5	5

[G] - Anova & Dunnett: * = $p \leq 0.05$
[G1] - Kruskal-Wallis & Dunn

Table 3

Summary of Body Weight Gains (g)

2954-001
Bodyweight Gain (Interval)

Sex: Female		Day(s) Relative to Start Date				
		49 → 56	56 → 63	63 → 70	70 → 77	77 → 91
Group 1	Mean	3.6	8.4	2.2	2.0	9.0
	SD	6.5	3.2	6.1	11.6	6.4
	N	5	5	5	5	5
Group 2	Mean	6.8	7.2	9.4	3.0	9.4
	SD	7.0	6.1	7.0	8.0	6.8
	N	5	5	5	5	5
Group 3	Mean	3.2	7.4	8.6	6.0	4.6
	SD	5.4	4.5	4.3	4.9	9.7
	N	5	5	5	5	5
Group 4	Mean	2.0	12.2	5.2	2.4	-2.8
	SD	6.1	2.6	6.6	3.4	7.4
	N	5	5	5	5	5

Anova & Dunnett

Table 4

Summary of Food Consumption

2954-001

Daily Food Cons Per Animal (g)

Sex: Male		Day(s) Relative to Animal Start Date						
		1 → 7 [I]	1 → 8 [G]	8 → 15 [G]	15 → 22 [G]	27 → 28 [I]	22 → 28 [I]	22 → 29 [I]
Group 1	Mean	25.8n	26.6	27.5	26.8	19.0n	27.3n	31.3n
	SD	1.7	1.5	1.7	2.7	-	0.0	1.4
	N	2	4	4	4	1	2	2
Group 2	Mean	26.5n	26.4	27.2	27.6	28.0n	27.8n	30.2n
	SD	0.3	2.1	1.8	3.9	-	0.4	1.2
	N	2	4	4	4	1	2	2
Group 3	Mean	25.8n	25.3	25.9	26.6	25.0n	27.2n	29.7n
	SD	0.4	1.3	2.3	2.0	-	2.2	0.3
	N	2	4	4	4	1	2	2
Group 4	Mean	24.1n	24.7	26.8	27.0	-	28.1n	27.0n
	SD	1.1	1.8	0.9	0.7	-	0.0	0.2
	N	2	4	4	4	-	2	2

[I] - n - Inappropriate for statistics
[G] - Anova & Dunnett

Table 4

Summary of Food Consumption

2954-001

Daily Food Cons Per Animal (g)

Sex: Male		Day(s) Relative to Animal Start Date						
		29 → 36	36 → 43	43 → 50	50 → 57	57 → 64	64 → 71	71 → 78
Group 1	Mean	31.3n	31.3n	30.4n	32.6n	33.0n	31.9n	31.3n
	SD	0.5	0.2	0.2	0.5	1.2	0.2	1.8
	N	2	2	2	2	2	2	2
Group 2	Mean	30.9n	29.7n	29.2n	30.8n	31.2n	31.0n	30.6n
	SD	1.9	0.9	1.6	1.5	1.5	1.7	0.1
	N	2	2	2	2	2	2	2
Group 3	Mean	31.2n	30.3n	29.9n	30.9n	30.7n	30.6n	30.6n
	SD	1.8	2.2	1.9	2.0	3.1	2.2	3.5
	N	2	2	2	2	2	2	2
Group 4	Mean	28.4n	27.6n	27.3n	27.8n	27.7n	27.3n	26.6n
	SD	1.2	0.1	0.1	0.4	0.9	0.4	0.3
	N	2	2	2	2	2	2	2

n - Inappropriate for statistics

Table 4

Summary of Food Consumption

2954-001

Daily Food Cons Per Animal (g)

Sex: Male		Day(s) Relative to Animal Start Date 78 → 85	85 → 86	85 → 90
Group 1	Mean	29.8n	29.0n	26.6n
	SD	1.2	-	-
	N	2	1	1
Group 2	Mean	29.7n	30.7n	26.9n
	SD	0.6	-	-
	N	2	1	1
Group 3	Mean	29.7n	36.5n	29.8n
	SD	3.6	-	-
	N	2	1	1
Group 4	Mean	27.0n	28.7n	27.0n
	SD	0.0	-	-
	N	2	1	1

n - Inappropriate for statistics

Table 4

Summary of Food Consumption

2954-001

Daily Food Cons Per Animal (g)

Sex: Female		Day(s) Relative to Animal Start Date						
		1 → 7 [I]	1 → 8 [G]	8 → 15 [G]	15 → 22 [G]	27 → 28 [I]	22 → 28 [I]	22 → 29 [I]
Group 1	Mean	20.2 n	18.7	18.9	17.9	24.0 n	17.9 n	19.4 n
	SD	1.5	0.6	0.5	3.3	-	2.0	0.9
	N	2	4	4	4	1	2	2
Group 2	Mean	18.7 n	19.3	19.0	18.5	20.0 n	19.4 n	21.4 n
	SD	1.1	2.0	2.6	4.4	-	2.8	0.8
	N	2	4	4	4	1	2	2
Group 3	Mean	18.1 n	18.6	18.2	15.6	16.0 n	12.1 n	20.3 n
	SD	0.5	2.7	1.3	6.9	-	7.1	0.1
	N	2	4	4	4	1	2	2
Group 4	Mean	20.1 n	18.4	18.8	17.5	-	18.8 n	19.8 n
	SD	9.7	1.4	1.7	3.3	-	2.1	0.9
	N	2	4	4	4	-	2	2

[I] - n - Inappropriate for statistics
[G] - Anova & Dunnett

Table 4

Summary of Food Consumption

2954-001

Daily Food Cons Per Animal (g)

Sex: Female		Day(s) Relative to Animal Start Date						
		29 → 36	36 → 43	43 → 50	50 → 57	57 → 64	64 → 71	71 → 78
Group 1	Mean	20.0n	5.6n	18.3n	18.9n	19.2n	18.6n	18.6n
	SD	1.0	0.2	0.8	0.0	0.4	0.7	1.8
	N	2	2	2	2	2	2	2
Group 2	Mean	21.9n	3.5n	20.8n	21.8n	21.3n	21.4n	21.6n
	SD	1.6	1.2	1.1	0.6	0.7	1.3	1.5
	N	2	2	2	2	2	2	2
Group 3	Mean	21.1n	8.7n	20.3n	20.8n	20.0n	20.4n	20.9n
	SD	0.3	4.5	0.2	0.5	0.3	0.4	0.4
	N	2	2	2	2	2	2	2
Group 4	Mean	21.8n	2.0n	19.4n	20.9n	20.2n	20.3n	19.9n
	SD	0.9	-	1.0	2.0	1.5	2.4	2.0
	N	2	1	2	2	2	2	2

n - Inappropriate for statistics

Table 4

Summary of Food Consumption

2954-001

Daily Food Cons Per Animal (g)

Sex: Female		Day(s) Relative to Animal Start Date 78 → 85	85 → 89	85 → 90
Group 1	Mean	18.3n	19.8n	20.3n
	SD	0.4	-	-
	N	2	1	1
Group 2	Mean	20.7n	-	21.0n
	SD	0.7	-	1.9
	N	2	-	2
Group 3	Mean	19.5n	-	20.6n
	SD	0.5	-	0.4
	N	2	-	2
Group 4	Mean	18.1n	-	19.9n
	SD	0.3	-	0.9
	N	2	-	2

n - Inappropriate for statistics

Appendix 1

DEVIATIONS

All deviations that occurred during the study have been authorized/acknowledged by the Study Director, assessed for impact, and documented in the study records. All Protocol deviations are listed below.

None of the deviations were considered to have impacted the overall integrity of the study or the interpretation of the study results and conclusions.

Formulations and Dosing

- During dosing for 1 Group 3 male (Animal No. 4013), the needle location dose site was not recorded. Subsequent study data are available to demonstrate that the study objectives were satisfactorily achieved as a result of performance of this function. Therefore, this minor documentation error does not negatively impact the Study Director's ability to interpret study data.

Husbandry

- On Day -8, two Group 4 males (Animal No. 4014 and 4015) were unnecessarily fasted overnight. Inappropriate fasting did not negatively impact the overall health status of the animals over the course of the study.

In-Life Observations, Measurements, and Evaluations

- On several occasions for several animals, the clinical observation prior to necropsy was not performed. The missing data points had no impact on the study as enough animals/observations were available to accomplish the objectives of the study.
- Prior to necropsy body weights were not obtained for 3 Group 1 females (Animal No. 1506 to 1508) and 3 Group 2 females (Animal No. 2506 to 2508). The missing data points had no impact on the study as enough animals/observations were available to accomplish the objectives of the study.
- Prior to necropsy, three Group 1 females (Animal No. 1506 to 1508) and 3 Group 2 females (Animal No. 2506 to 2508) were not fasted overnight. The food was removed on Day 90 prior to being delivered to the Necropsy Department. As these are isolated occurrences, this will not affect the quality or integrity of the study.
- On Day 43, the food consumption value for 2 Group 4 females (Animal No. 4509 and 4510) was excluded. The nature of these data is such that they are considered to be either unreasonable in terms of what could be possible or atypical of what might be reasonably expected. Inclusion of such data may lead to erroneous interpretation and as such, these values will be excluded from the mean values. As these are isolated occurrences, this will not affect the quality or integrity of the study.

Laboratory Evaluations

Appendix 1

- At the clinical chemistry blood collection for 1 Group 1 female (Animal No. 1522) only 0.8 mL of blood was collected. The sample still affords analysis despite being slightly low.
- The clinical chemistry blood collection for 1 Group 4 female (Animal No. 4511) could not be obtained. The missing data points had no impact on the study as enough animals/observations were available to accomplish the objectives of the study.

Postmortem and Pathology

- During tissue trimming for 1 Group 1 female (Animal No. 1510) the lumbar spinal cord was not obtained. There will still be a sufficient number of samples from the other animals in this group and as such, the lack of this sample will not impact the ability to interpret the data.
- During tissue trimming for 1 Group 1 male (Animal No. 1008), one iliac lymph node and the left lateral lobe of the liver could not be obtained. There will still be a sufficient number of samples from the other animals in this group and as such, the lack of this sample will not impact the ability to interpret the data.
- During necropsy for 1 Group 4 male (Animal No. 4024), the organ weight was not obtained for the thyroid. The thyroid tissue was placed into 10% neutral buffered formalin (NBF) prior to the organ weight collection. There was a large enough group size to accomplish study objectives, therefore, there was no impact to the study. The missing data points had no impact on the study as enough animals/observations were available to accomplish the objectives of the study.
- During necropsy for 1 Group 3 female (Animal No. 3523), the bone marrow smear was unable to be collected. The bone marrow smear was placed into 10% NBF prior to the organ weight collection. There was a large enough group size to accomplish study objectives, therefore, there was no impact to the study. The missing data points had no impact on the study as enough animals/observations were available to accomplish the objectives of the study.
- During splenocyte isolation in B5, the addition of 10 to 15 mL of RPMI 1640 was not documented after the second lysis incubation. Impact Statement. Subsequent study data are available to demonstrate that the study objectives were satisfactorily achieved as a result of performance of this function. Therefore, this error does not negatively impact the Study Director's ability to interpret study data.
- During splenocyte processing, the samples were resuspended in 5 mL of DPBS prior to counting on Vi-cell for 5 Group 1 males (Animal No. 1011 to 1015), 1 Group 1 female (Animal No. 1511), 5 Group 2 males (Animal No. 2011 to 2015), 1 Group 2 female (Animal No. 2511), 5 Group 3 males (Animal No. 3011 to 3015), 1 Group 3 female (Animal No. 3511), 3 Group 4 males (Animal No. 4011 to 4013), and 1 Group 4 female (Animal No. 4511). The incorrect media

Appendix 1

used did not have an impact on the quality of the samples. Therefore, appropriate evaluations of the data can still be conducted with no impact on study interpretation.

Appendix 1

PROTOCOL AMENDMENT NO. 12

Testing Facility Study No. 2954-001

Sponsor Reference No. UTSW.Gray-002

A Single Dose Toxicity Study of AAV9/SURF1 Administered by Intrathecal Injection in Rats

Status: GLP

SPONSOR:
University of Texas Southwestern Medical Center
5323 Harry Hines Blvd
Dallas, TX 75390
USA

TESTING FACILITY:
Charles River Laboratories, Inc.
54943 North Main Street
Mattawan, MI 49071
USA

Appendix 1

SUMMARY OF CHANGES AND JUSTIFICATIONS

Protocol Effective Date: 14-Aug-2020

Note: When applicable, additions are indicated in **bold underlined** text and deletions are indicated in ~~**bold strikethrough**~~ text in the affected sections of the document.

**Item or Section(s)**	**Justification**
**Amendment 1**	**Effective Date of Change: 17 Sep 2020**
Section 5.2, Section 9	Dose concentration and dose levels updated based on final titer of test article provided in the CoA by the Sponsor. Test article storage conditions updated.
Section 5.3	Vehicle storage conditions updated.
Section 7	Additional animals ordered due to test article availability leading to later dosing dates required and thus animals well out of weight/age range appropriate for interpretation of inlife and postlife data.
Section 14.6	Left hemisphere specified for brain histopathology
Section 14.7	Due to tissue size and sample priority of biodistribution samples, remaining frozen tissue samples for archival will only be taken if available.
Section 15.4	Clarification of splenocyte collection and processing procedure.
Attachment A	Tissue weights removed for non-standard tissues. Left/right designations or specific locations added as needed for select tissues.
**Amendment 2**	**Effective Date of Change: 12 Oct 2020**
Section 4, Attachment B	Anatomic pathologist identified, slide shipment contact and details added.
Section 15.4	Splenocytes will be slow frozen to ensure sample quality and cell viability.
Attachment A	Parathyroid gland will be collected with the thyroid for the remaining frozen tissue to archive.
**Amendment 3**	**Effective Date of Change: 18 Nov 2020**
Section 7	Animals were ordered for the Day 29 cohort and were on site in advance of dosing. However, the sponsor provided details that the test material would not be available for the dosing dates, and no likely availability in the near future. Thus, the animals were removed from study and added to the colony as they would be out of the specified age range for dosing. Additional animals will be ordered at ages specified in the protocol when test material is available.

Appendix 1

**Item or Section(s)**	**Justification**
Section 9, Section 14	A 4 Day window is needed to accommodate necropsy scheduling for the Day 91 necropsies. Expanding the window will allow more adequate allocation of staff and resources to ensure quality tissue collection.
Attachment A	Clarified gland samples that are collected together.
Attachment B	Contact information updated for shipment of bioanalytical and splenocyte samples.
**Amendment 4**	**Effective Date of Change: 21 Jan 2021**
Section 4	PI for Anatomic Pathology updated.
**Amendment 5**	**Effective Date of Change: 12 Feb 2021**
Section 4	PI for Anatomic Pathology updated.
**Amendment 6**	**Effective Date of Change: 15 Apr 2021**
Section 5	Test article concentrations updated after ddpcr analysis completed. Additional lot information included.
Section 4, Attachment B	Addresses updated for pathology test site
Section 7	Additional males are required to complete study enrollment and dosing as well as provide extras in the event of an animal not being suitable for dosing. During the previous dosing interval, animals were onsite however test article was not available. Thus the animals aged out of the dosing window.<br>The number of male animals on study was updated to reflect the additional group 3 animal that was dosed. See justification below.
Section 9	A 6th animal was inadvertently dosed as a group 3 animal and thus the experimental design table has been updated accordingly. Furthermore, following ddpcr analysis, the dose levels, volume, and dose concentration are updated accordingly.
**Amendment 7**	**Effective Date of Change: 29 Apr 2021**
Section 3	Alternate Sponsor representative updated.
Section 4	Study Director and Alternate updated due to a change in personnel.
Section 9	Dose volume for vehicle corrected to match the footnote.
**Amendment 8**	**Effective Date of Change: 20 Jul 2021**
Section 16	Section updated to reflect statistics performed.
Section 25	Reference removed due to the change in statistics performed.
**Amendment 9**	**Effective Date of Change: 09 Aug 2021**
Section 4	Inclusion of a peer review of the histopathology work as requested by the Sponsor.

Appendix 1

Item or Section(s)	Justification
Section 15.2	Removal of standard wording that is not applicable since the pathologist is outsourced.
Attachment B	Addition of shipment details for a peer review of the histopathology work as requested by the Sponsor.
**Amendment 10**	**Effective Date of Change: 25 Aug 2021**
3. Sponsor	Sponsor consultant added at the request of the Sponsor.
**Amendment 11**	**Effective Date of Change: 12 Oct 2022**
4. RESPONSIBLE PERSONNEL	Updated to align with current protocol template requirements.
15.2. Microscopic Evaluation	Clarification that images will be included in the pathology report.
Attachment B	Shipping details added.
**Amendment 12**	**Effective Date of Change: 13 Mar 2023**
4. RESPONSIBLE PERSONNEL	Based on the pathology findings, sample analysis and phase reports will not be included per Sponsor.
13.2. Serum Sample Shipping and Analysis	
13.5. CSF Sample Shipping and Analysis	
15.3. Biodistribution/Gene Expression Analysis	
15.4. Splenocyte Collection and Analysis	

Appendix 1

TABLE OF CONTENTS

1. OBJECTIVE(S) 7
2. PROPOSED STUDY SCHEDULE 7
3. SPONSOR 7
4. RESPONSIBLE PERSONNEL 8
5. TEST MATERIALS 10
6. DOSE FORMULATION AND ANALYSIS 11
7. TEST SYSTEM 12
8. HUSBANDRY 13
9. EXPERIMENTAL DESIGN 15
10. SURGICAL ANIMAL PREPARATION 15
11. IN-LIFE PROCEDURES, OBSERVATIONS, AND MEASUREMENTS 16
12. CLINICAL PATHOLOGY 17
13. BIOANALYTICAL EVALUATION 18
14. TERMINAL PROCEDURES 20
15. HISTOLOGY AND MICROSCOPIC EVALUATION 23
16. STATISTICAL ANALYSIS 24
17. COMPUTERIZED SYSTEMS 26
18. REGULATORY COMPLIANCE 26
19. QUALITY ASSURANCE 27
20. AMENDMENTS AND DEVIATIONS 27
21. RETENTION AND DISPOSITION OF RECORDS, SAMPLES, AND SPECIMENS 27
22. REPORTING 28
23. JUSTIFICATIONS AND GUIDELINES 28
24. ANIMAL WELFARE 29
25. REFERENCES 30
ATTACHMENT A 31
ATTACHMENT B 34
SPONSOR APPROVAL 36

Appendix 1

TESTING FACILITY APPROVAL...37

Appendix 1

1. OBJECTIVE(S)

The objective of this study is to characterize the toxicity, biodistribution, and gene expression of AAV9/SURF1 for the treatment of SURF1 Leigh Syndrome.

2. PROPOSED STUDY SCHEDULE

Proposed study dates are listed below. Actual dates will be included in the Final Report.

Experimental Starting Date:	18 Aug 2020<br>(First date of study-specific data collection)
Experimental Completion Date:	To be included in the Final Report<br>(Last date on which data are collected)
Draft Report:	Approximately 10 weeks<br>(Following last day of necropsy)
Final Report:	The date on which the Study Director signs the final report.

3. SPONSOR

Role	Name/Contact Information
Sponsor Representative	Steven Gray, PhD<br>University of Texas Southwestern Medical Center<br>5323 Harry Hines Blvd<br>University of Texas Southwestern Medical Center<br>Dallas, TX 75390<br>Tel: 214-648-0670<br>E-mail: steven.gray@UTsouthwestern.edu
Alternate Sponsor Representative	Roxana Ploski<br>University of Texas Southwestern Medical Center<br>5323 Harry Hines Blvd<br>University of Texas Southwestern Medical Center<br>Dallas, TX 75390<br>E-mail: roxana.ploski@utsouthwestern.edu.
Sponsor Scientific Consultant	Michael W. Lawlor M.D., Ph.D.<br>Professor<br>Associate Director, Neuroscience Research Center<br>Division of Pediatric Pathology

Appendix 1

	Department of Pathology and Laboratory Medicine<br>Medical College of Wisconsin<br>9200 W. Wisconsin Ave.<br>Milwaukee, WI 53226<br>Tel: 414-955-2959<br>E-mail: mlawlor@mcw.edu

4. RESPONSIBLE PERSONNEL

Role/Phase	Quality Assurance Unit	Name/Contact Information
Study Director	Charles River	Sarah M. Davis, BS, LATG<br>Tel: 269-668-3336 ext. 1250<br>E-mail: sarah.davis2@crl.com
Alternate Contact	Charles River	Andrew C. Petticoffer, BA<br>Tel: 269-668-3336 ext. 1470<br>E-mail: andrew.petticoffer@crl.com
Testing Facility Management	Charles River	Scott T. Wilson, BS, LAT<br>Tel: 269-668-3336 ext. 1610<br>E-mail: scott.wilson@crl.com
Testing Facility QAU	Charles River	Janis Kissel, BS, RQAP-GLP<br>Tel: 269-668-3336, ext. 3170<br>E-mail: janis.kissel@crl.com
Peer Review Pathologist	Not Applicable	Jacqueline Brassard DVM, PhD, DACVP<br>Brassard Toxicologic Pathology Consultancy Corp.<br>163 Mountain View Dr.<br>Tustin, California 92780<br>Tel: 314-397-3208<br>E-mail: dr.jbrassard@gmail.com
**Individual Scientist (IS)**		
Clinical Pathologist	Charles River	Charles Wiedmeyer, DVM, PhD, DACVP<br>Tel:269-668-3336 ext. 1299

Appendix 1

Role/Phase	Quality Assurance Unit	Name/Contact Information
		E-mail: charles.wiedmeyer@crl.com<br>Address as cited for Testing Facility
**Principal Investigator (PI)**		
~~**Serum, CSF, and Tissue Biodistribution Analysis**~~	~~**Test Site**~~	~~**Steven Gray, PhD**~~<br>~~**University of Texas Southwestern Medical Center**~~<br>~~**5323 Harry Hines Blvd**~~<br>~~**University of Texas Southwestern Medical Center**~~<br>~~**Dallas, TX 75390**~~<br>~~**Tel: 214-648-0670**~~<br>~~**E-mail: steven.gray@UTsouthwestern.edu**~~
Anatomic Pathologist	Test Site	Jessica Hoane, DVM, MTOX, DACVP, DABT<br>Senior Pathologist<br>StageBio<br>8415 Progress Drive, Suite Q<br>Frederick, MD 21701<br>Tel: 513-204-4400<br>E-mail: jhoane@stagebio.com

Each IS and PI is required to report all deviations or other circumstances that could affect the quality or integrity of the study to the Study Director in a timely manner for authorization/acknowledgement. Each IS and PI will provide a report addressing their assigned phase of the study, which will be included as an appendix to the Final Report.

The PI Phase Report will include the following:

- A Statement of Compliance (if the applicable phase is GLP)
- A QA Statement (for Sponsor designated PI or for Testing Facility designated PI if audited by a QAU other than that of the Testing Facility and if the applicable phase is GLP)
- The archive site for all records, samples, specimens and reports generated from the phase or segment (alternatively, details regarding the retention of the materials may be provided to the Study Director for inclusion in the Final Report)

- A listing of critical computerized systems used in the conduct and/or interpretation of the assigned study phase

5. TEST MATERIALS

5.1. Test Article Characterization

The Sponsor will provide to the Testing Facility documentation of the identity, strength, purity, composition, and stability for the test article. A Certificate of Analysis or equivalent documentation will be provided for inclusion in the Final Report.

The Sponsor has appropriate documentation on file concerning the method of synthesis, fabrication or derivation of the test article, and this information is available to the appropriate regulatory agencies should it be requested.

5.2. Test Article Identification

**Identification:**	AAV9/SURF1
**Alternate Identification:**	scAAV9/CBh-hsaSURF1opt-BGHpA
**Batch/Lot No.:**	Lot 1: TX-07-20-196<br>Lot 2: TX-07-20-001
**Expiration/Retest Date:**	To be included in the Final Report
**Physical Description:**	To be included in the Final Report
**Purity:**	To be included in the Final Report
**Correction Factor:**	NA
**Concentration:**	Lot 1: TX-07-20-196<br>4.15 E13 vg/mL<br>Lot 2: TX-07-20-001<br>3.36 E13 E13 vg/mL
**Storage Conditions:**	Frozen (-60 to -90°C) or Refrigerated (2 to 8°C)
**Provided by:**	Sponsor
**Test Article Contact:**	Samer Dahshi<br>Tel: 214-648-7164<br>E-mail: samer.dahshi@UTSouthwestern.edu

NA = Not applicable.

5.3. Vehicle Information

**Identification:**	PBS containing 5% D-sorbitol and 0.001% pluronic F-68
**Alternate Identification:**	Diluent
**Storage Conditions:**	Frozen (-60 to -90°C) or Refrigerated (2 to 8°C)
**Characterization:**	Documentation of the strength, purity, composition, stability, and other pertinent information on each batch of vehicle, will be limited to that information listed on the label of this commercially available material, unless otherwise noted.

5.4. Reserve Samples

A reserve sample from each lot of test and vehicle articles used in this study will be collected and stored by the Sponsor in a secure area with the appropriate environmental controls

5.5. Test Article Inventory and Disposition

Records of the receipt, distribution, storage, and disposition of test materials (including empty containers of Sponsor-provided materials) will be maintained until study finalization.

The Sponsor will be contacted for proper disposition of materials (retain/ship/discard) after completion of the in-life phase of the study, and following confirmation that these materials are not assigned to other studies.

5.6. Safety

A Safety Data Sheet (SDS), or equivalent documentation, will be provided by the Sponsor (if available). It is the responsibility of the Sponsor to notify the Testing Facility of any special handling requirements of the test article. Otherwise routine safety precautions will be followed. Appropriate gloves, safety glasses and arm covers will be worn by individuals working with neat test material(s) or formulations.

6. DOSE FORMULATION AND ANALYSIS

6.1. Preparation of Formulations

Dose formulations will be divided into aliquots where required to allow to be dispensed on each dosing occasion.

Preparation Details

Dose Formulation	Frequency of Preparation	Storage Conditions
Vehicle	Used as received	Same as Test Article
Dose Formulations	Prior to dosing[a]	Refrigerated (2 to 8°C)

[a] 60 days of established stability at refrigerated (2 to 8°C) storage conditions has been established.

Any residual volumes from each dosing occasion will be retained and stored refrigerated (2 to 8°C) unless otherwise requested by the Study Director. These retained volumes can be used on subsequent dosing days.

6.2. Preparation Details

The stock solution will be diluted as needed to prepare all dose concentrations using the Sponsor-supplied instructions as a guide.

6.3. Sample Collection and Analysis

The test and control articles will be used as received from the Sponsor; therefore, samples for dose formulation analysis will not be collected by the Testing Facility.

Appendix 1

7. TEST SYSTEM

Species:	Rat
Strain:	CD® [Crl:CD®(SD)]
Condition:	Purpose-bred, naïve
Source:	Charles River Laboratories, Inc., Raleigh, NC or Kingston, NY<br>The source used will be documented in the raw data.
Number Ordered:	
Male:	113
Female:	110
Number on Study:	
Male:	61
Female:	60
Expected Age at Arrival:	Ordered to be 6 weeks of age at arrival
Expected Weight at Arrival:	Commensurate with age; males will generally weigh 215 to 260g and females will generally weigh 140 to 185g, as measured within 3 days of arrival. The actual range will be documented in the data.

The actual age and weight of animals received will be listed in the Final Report.

7.1. Animal Identification

Method:	Each animal will be assigned an animal number to be used in Provantis™ and will be implanted with a microchip bearing a unique identification number. The individual animal number, implant number, and the Testing Facility study number will comprise a unique identification for each animal. The animal cage will be identified by the study number, animal number, group number, and sex.

7.2. Environmental Acclimation

Duration:	At least 1 week
Details:	During this acclimation period, all animals will be observed daily for any clinical signs of disease, and all animals will be given a detailed clinical examination within 3 days of the first dose administration.

7.3. Selection, Assignment, Replacement and Disposition of Animals

Assignment and Randomization:	Animals will be randomly assigned to groups upon receipt. Males and females will be randomized separately.
	All animals with any evidence of disease or physical abnormalities will not be selected for study.
Replacement:	Before the initiation of dosing, any assigned animals considered unsuitable for use in the study will be replaced by alternate animals obtained from the same shipment and maintained under the same environmental conditions.
	After initiation of dosing, study animals may be replaced during the replacement period with alternate animals in the event of accidental injury, non-test article-related health issues, or similar circumstances.
	Alternate animals may be used as replacements per Testing Facility SOP.
Disposition:	Extra animals obtained for this study, but not placed on study, will be either transferred to a Testing Facility stock or training colony, or euthanized and discarded.
	The disposition of all animals will be documented in the study records.

8. HUSBANDRY

8.1. Housing

Housing:	Pair-housed, when possible (animals may be housed 2 to 3/cage during acclimation and in-life depending on study design).
	Housing set-up is as specified in the USDA Animal Welfare Act (9 CFR, Parts 1, 2 and 3) and as described in the *Guide for the Care and Use of Laboratory Animals*. Animals will be separated during designated procedures/activities or will be separated as required for monitoring and/or health purposes, as deemed appropriate by Study Director and/or Clinical Veterinarian.
Caging:	Solid bottom cages with nonaromatic bedding. The bedding will be from an approved supplier and documented in the study data.

8.2. Animal Enrichment

Supplemental Enrichment:	Animal enrichment will be provided according to Testing Facility SOP.

Appendix 1

8.3. Environmental Conditions

Temperature and Humidity:	Temperature and humidity will be maintained according to Testing Facility SOP.
Lighting:	Fluorescent lighting will be provided via an automatic timer for approximately 12 hours per day. On occasion, the dark cycle may be interrupted intermittently due to study-related activities.

8.4. Food

Diet:	The basal diet will be block Lab Diet® Certified Rodent Diet #5002, PMI Nutrition International, Inc.
Frequency:	Ad libitum, except during designated procedures
Analysis:	Results of analysis for nutritional components and environmental contaminants are provided by the supplier and are on file at the Testing Facility.<br>There are no known contaminants in the food that would interfere with this study.

8.5. Water

Type:	Tap water
Frequency:	Supplied ad libitum to all animals via an automatic water system unless otherwise indicated.
Analysis:	There are no known contaminants in the water that would interfere with this study. The drinking water used will be monitored for specified contaminants at periodic intervals according to Testing Facility SOP.

8.6. Veterinary Care

Veterinary care will be available throughout the course of the study and animals will be examined by the veterinary staff as warranted by clinical signs or other changes. In the event that animals show signs of illness or distress, the responsible veterinarian may make initial recommendations about treatment of the animal(s) and/or alteration of study procedures, which must be approved by the Study Director (or scientific designee). Treatment of the animal(s) for minor injuries or ailments may be approved without prior consultation with the Sponsor representative when such treatment does not impact fulfillment of the study objectives. If the condition of the animal(s) warrants significant therapeutic intervention or alterations in study procedures, the Sponsor representative will be contacted, when possible, to discuss appropriate action. If the condition of the animal(s) is such that emergency measures must be taken, the Study Director (or scientific designee) and/or attending veterinarian will attempt to consult with the Sponsor representative prior to responding to the medical crisis, but the Study Director (or

scientific designee) and/or veterinarian has authority to act immediately at his/her discretion to alleviate suffering. The Sponsor representative will be fully informed of any such events.

9. EXPERIMENTAL DESIGN

Group	Treatment	Dose Level (vg)	Dose Volume (µL)	Dose Concentration (vg/µL)	No. of Animals							
					Day 1 Dose		Day 8 Necropsy *		Day 29 (± 1) Necropsy **		Day 91 (± 4) Necropsy	
					M	F	M	F	M	F	M	F
1	Vehicle	0	60<br>74[2]	0	15	15	5	5	5	5	5	5
2	Low Dose	0.28E12	20[1]<br>25[2]	1.38E10[1]<br>1.12E10[2]	15	15	5	5	5	5	5	5
3	Mid Dose	0.83E12	20[1]<br>25[2]	4.15E10[1]<br>3.36 E10[2]	16	15	6	5	5	5	5	5
4	High Dose	2.49E12	60[1]<br>74[2]	4.15E10[1]<br>3.36 E10[2]	15	15	5	5	5	5	5	5

No. = Number
[1] Lot 1
[2] Lot 2

* Three of the animals from group 4 in the Day 8 time point received the same total vg dose, but in a 74 uL volume due to lower test article dose concentration in lot 2 of 3.36E10 vg/uL.

**All animals at the day 29 time point received the same total vg dose assigned to each respective group, but in a 24% higher volume due to lower test article dose concentration in Lot 2. Group 1 volume = 74 uL, group 2 volume = 25 uL, group 3 volume = 25 uL, and group 4 volume = 74 uL.

9.1. Administration of Test Article(s)

Route: Intrathecal injection

Frequency and Duration: Once on Day 1

Dose Level: Up to 2.49E12 vg

10. SURGICAL ANIMAL PREPARATION

10.1. Pre-operative Procedures

Details: Anesthesia will be induced and maintained as indicated in Testing Facility SOP.

Appendix 1

10.2. Surgical Procedure

The procedures outlined below are intended to serve as guidance for successful dose administration. Minor alterations in the surgical procedures may occur as necessary at the discretion of the surgeon or Study Director and will be documented in the study data and will not be considered a protocol deviation.

Animals will be anesthetized, placed in a ventral recumbency, and prepped using aseptic technique. The dose administration will be performed using a gas tight Hamilton syringe with a disposable needle. Under fluoroscopic guidance, if needed, a needle will be inserted into the lumbar cistern. The location of the needle may vary based on the discretion of the surgeon and final needle location will be recorded. Once the needle is appropriately placed, the animals will be dosed with test article or vehicle and flushed with vehicle over 30±5 seconds for groups 1 and 4, and 10±5 seconds for groups 2 and 3. Upon completion of the dose, the needle will be removed and the animal will then be allowed to recover.

10.3. Postoperative Procedures

Details: Postoperative procedures will be conducted in accordance with Testing Facility SOP.

11. IN-LIFE PROCEDURES, OBSERVATIONS, AND MEASUREMENTS

Standard In-life Assessments

Parameter	Population(s)	Frequency (minimum required)	Comments
Mortality/Cageside Observations	All surviving animals	At least twice daily[a,b] (morning and afternoon) beginning upon arrival through termination/release.	Animals will be observed within their cage unless necessary for identification or confirmation of possible findings.<br><br>Animals will be observed for morbidity, mortality, injury, and availability of food and water. Any animals in poor health will be identified for further monitoring and possible euthanasia.

Parameter	Population(s)	Frequency (minimum required)	Comments
Detailed Clinical Observations	All Main Study animals	Weekly; from at least Week -1 and throughout the study.[c]	Animals are removed from the cage.<br><br>Observations will include, but will not be limited to, evaluation of the skin, fur, eyes, ears, nose, oral cavity, thorax, abdomen, external genitalia, limbs and feet, respiratory and circulatory effects, autonomic effects such as salivation, nervous system effects including tremors, convulsions, reactivity to handling, unusual behavior, and palpation of tissue masses.
Individual Body Weights	All Main Study animals	Within 3 days of arrival, Day -1, and once weekly during the terminal and recovery periods.	Fasted weight on the day of necropsy. Not collected from animals found dead.
Food Consumption	All Main Study animals	Weekly.[d]	Quantitatively measured

[a] Procedures on alternate animals will be conducted per Testing Facility SOP.
[b] Except on days of receipt and necropsy where frequency will be at least once daily.
[c] For observations that cannot be attributed to an individual animal due to social housing (e.g., watery feces), the observation will be noted to each animal in the socialized group.
[d] For observations of reduced appetite that cannot be attributed to an individual animal due to social housing, the observations will be noted for each animal in the socialized group.

12. CLINICAL PATHOLOGY

12.1. Sample Collection

Clinical Pathology Sample Collection

Group Nos.	Time Point(s)	Hematology	Coagulation	Clinical Chemistry
All Surviving Animals	At necropsy	X	X	X
Unscheduled Euthanasia (when possible)	See the Unscheduled Euthanasia section of this protocol.			

**Volume (mL)[a]:**	NA	1 mL	1.2 mL	1.3 mL
**Fasting Required:**	Free access to drinking water but will be fasted overnight (at least 8 hours) prior to blood collection.			
**Anticoagulant:**	NA	K_2EDTA	Sodium Citrate	Serum Gel Separator
**Special Requirements:**	NA	NA	NA	NA
**Processing:**	NA	None	Plasma	Serum

X = Sample to be collected; NA = Not applicable; hr = hour; pre = predose; post= postdose.
[a] Additional blood samples may be obtained (e.g. due to sample quality) if permissible sampling frequency and blood volume are not exceeded.

Appendix 1

Blood Sample Collection Method: Cardiac puncture or vena cava after carbon dioxide inhalation

The following clinical pathology tests will be conducted on available samples, as survival allows, and the data will be interpreted by a Clinical Pathologist.

12.2. Hematology

Hematology Parameters

Leukocyte count (total and absolute differential)	Mean corpuscular hemoglobin, mean corpuscular volume, mean corpuscular
Erythrocyte count	hemoglobin concentration (calculated)
Hemoglobin	RDW
Hematocrit	Platelet count
Absolute reticulocytes	Blood smear (preserve and stain)[a]

[a] Blood smear review may be performed on select animals per Testing Facility SOP.

12.3. Coagulation

Coagulation Parameters

Prothrombin time	Fibrinogen
Activated partial thromboplastin time	

12.4. Clinical Chemistry

Clinical Chemistry Parameters

Alkaline phosphatase	Globulin and A/G (albumin/globulin) ratio (calculated)
Total bilirubin (with direct bilirubin if total bilirubin exceeds 1 mg/dL)	Glucose
Aspartate aminotransferase	Total cholesterol
Alanine aminotransferase	Triglycerides
Urea nitrogen	Electrolytes (sodium, potassium, chloride)
Creatinine	Calcium
Total protein	Phosphorus
Albumin	Sample quality

13. BIOANALYTICAL EVALUATION

13.1. Serum Sample Collection

Animals/Interval: All animals prior to their scheduled necropsy

Method/Comments: Cardiac puncture or vena cava after carbon dioxide inhalation

Volume (mL): 1 mL

Anticoagulant: None, Serum Gel Separator

Appendix 1

Whole Blood Storage:	Ambient
Container Label Requirements:	Study number, animal number, matrix of sample, interval and timepoint, analysis type, and aliquot number.
Processing:	Serum<br>The final processed sample(s) will be divided into 2 approximately equal aliquots
Final Storage Temperature:	Frozen (-60 to -90°C)

13.2. Serum Sample Shipping and Analysis

Analysis Performed By:	~~**The serum will be analyzed for AAV9 concentrations. All analytical work will be conducted by the Sponsor, using an analytical method developed and qualified by that laboratory.**~~<br>**The serum will be archived for possible future analysis by the Sponsor. If any future analysis is performed by the Sponsor, this will be not be included in the final report.**
Regulatory Requirements:	~~**The work performed in conjunction with this study will not be conducted in compliance with GLPs and will not be subject to review by the Quality Assurance Unit (QAU) of that laboratory. A Final Report will be prepared and submitted to Testing Facility for inclusion as an appendix in the main study Final Report.**~~<br>**If any possible future analysis is performed in conjunction with this study, it will not be conducted in compliance with GLPs, will not be subject to review by the Quality Assurance Unit (QAU) of that laboratory, and will not be included in the Final Report.**
Sample Shipping Contact:	All samples ~~to be analyzed~~ will be shipped to the designated Test Site, see Attachment B for shipping details.

13.3. Cerebral Spinal Fluid (CSF)

13.4. CSF Sample Collection

Animals and Intervals:	All animals prior to their scheduled necropsy
Collection Site:	Cisterna magna
Volume/Sample:	Maximum obtainable clean sample
Anticoagulant:	None

Sample Storage:	Stored on ice block or wet ice following collection until processing and final storage.
Container Label Requirements:	Study number, animal number, matrix of sample, interval and timepoint, analysis type, and aliquot number.
Processing:	The CSF sample(s) will be divided into 2 approximately aliquots
Final Storage Temperature:	Frozen (-60 to -90°C)

13.5. CSF Sample Shipping and Analysis

Analysis Performed By:	The CSF will be archived for possible future analysis **by the Sponsor**. If any future analysis is performed by the Sponsor, this will be not be included in the final report.
Regulatory Requirements:	If any possible future analysis is performed in conjunction with this study, it will not be conducted in compliance with GLPs, will not be subject to review by the Quality Assurance Unit (QAU) of that laboratory, and will not be included in the Final Report.
Sample Shipping Contact:	All samples will be shipped to the designated Test Site, see Attachment B for shipping details.

14. TERMINAL PROCEDURES

Terminal procedures are summarized in the following tables:

Terminal Procedures

Group No.	Necropsy Procedures			Histology Processing	Microscopic Evaluation	Biodistribution Collection and Analysis
	Necropsy	Tissue Collection	Organ Weights			
Found dead or unscheduled euthanasia	X	Full List[a]	NA	Full List[a]	Full List[a]	NA

Main Study Animals

Group No.	Scheduled Euthanasia Day	Necropsy Procedures			Histology Processing	Microscopic Evaluation	Biodistribution Collection and Analysis
		Necropsy	Tissue Collection	Organ Weights			
1-4	8, 29(±1), or 91(±4)	X	Full List[a]	Full List[a]	Full List[a]	Full List[a]	Full List[a]

Terminal Procedure Tables Footnotes:

Appendix 1

X = Procedure to be conducted; NA = Not applicable.
"Histology Processing"= embedded in paraffin, sectioned, mounted on glass slides, and stained with hematoxylin and eosin.
[a] See Tissue Weighing, Collection, Processing and Evaluation Table for list of tissues applicable to each procedure.

14.1. Method of Euthanasia

Euthanasia will be by carbon dioxide inhalation followed by a Testing Facility SOP approved method to ensure death, e.g. exsanguination.

14.2. Unscheduled Euthanasia

Moribund animals will be subject to Testing Facility SOP criteria and procedures. If possible, the samples below will be collected from animals euthanized in extremis, following veterinary consultation. A veterinary consultation is not required if the samples are collected following anesthesia or euthanasia. Blood collection methods utilized for animals euthanized in extremis may include suitable methods other than those presented in the respective blood collection section(s) of this protocol. Samples below are listed in order of collection priority.

Sample Type	Groups	Volume	Anticoagulant
Clinical Chemistry	All	1.3 mL	Serum Gel Separator
Hematology		1 mL	K_2EDTA
Coagulation		1.2 mL	Sodium Citrate
Serum		1 mL	Serum Gel Separator
CSF		Maximum Obtainable	None

Necropsy examinations will be performed 7 days a week. Animals that are found dead or euthanized in extremis after regular working hours will be refrigerated overnight and necropsies performed at the start of the next day.

14.3. Scheduled Euthanasia

Main Study animals surviving until scheduled euthanasia will be euthanized by the methods described above. When possible, the animals will be euthanized rotating across dose groups such that similar numbers of animals from each group, including controls, will be necropsied throughout the day.

14.4. Necropsy

Animals as detailed in the Terminal Procedures table will be subjected to a complete necropsy examination, which will include evaluation of the carcass and musculoskeletal system; all external surfaces and orifices; cranial cavity and external surfaces of the brain; and thoracic, abdominal, and pelvic cavities with their associated organs and tissues.

All animals will be examined carefully for external abnormalities including palpable masses.

Images may be generated for illustration of or consultation on gross observations. These images will not be used for data generation or interpretation and will not be archived or included in the Final Report.

14.5. Organ Weights

Body weight and the organ weights identified in the Tissue Weighing, Collection, Processing and Evaluation table will be recorded for all animals at the scheduled necropsies, and appropriate organ weight ratios will be calculated (relative to body and brain weights). Paired organs will be weighed together. Organ weights will be collected per Testing Facility SOP.

14.6. Tissue Collection and Preservation for Histopathology Analysis

Representative samples of tissues will be collected and preserved in 10% neutral buffered formalin, as detailed in the Terminal Procedures and Tissue Weighing, Collection, Processing and Evaluation tables. Additional tissue samples may be collected to elucidate abnormal findings.

For the brain (left hemisphere), standard collection and trimming will be performed according to Testing Facility SOP. The sections will include the following: forebrain, midbrain, hindbrain (including brainstem and cerebellum).

14.7. Tissue Collection for Biodistribution/Gene Expression Analysis

Tissues listed in Attachment A will be evaluated for biodistribution/gene expression by the Sponsor. Tissues will be collected and flash frozen in liquid nitrogen. The tissues collected will be split into 2 tubes (10-50 mg per tube). See Attachment B for shipping details. For any of the tissues listed in Attachment A as “remaining frozen tissues to archive”, following, histopathology and/or biodistribution sample collection, tissues will be collected into a single tube, flash frozen and will be archived by the sponsor.

The femur will be wrapped in PBS soaked gauze following collection and prior to flash freezing in liquid nitrogen.

Samples will not be collected for animals euthanized in extremis, or animals found dead.

These samples will be collected first after euthanasia using strict aseptic techniques and disposable instruments for each tissue or organ.

Brain and spinal cord will be collected first, then the liver followed by major organs (kidney, lung, heart, spleen), then remaining tissues.

Samples may be taken by trimming a representative section of the tissue with a clean scalpel, scissors, or by using a clean biopsy punch.

Care will be taken to ensure that cross-contamination between tissues does not occur. Gloves will be changed between collection and dissection of each tissue for analysis. In addition, non-disposable instruments will be wiped down with a 10% bleach solution, rinsed with water,

followed by a wipe down of 100% ethanol between each of the specified organs. Tissues for analysis will be collected prior to sample collection for any other parameters.

Following sample collection of all tissues designated for biodistribution analysis in Attachment A, remaining tissues will be stored frozen (-60 to -90°C) for possible future analysis.

15. HISTOLOGY AND MICROSCOPIC EVALUATION

15.1. Histology

Histology will be conducted per Testing Facility SOPs.

15.2. Microscopic Evaluation

Tissues as detailed in the Terminal Procedures and Tissue Weighing, Collection, Processing and Evaluation tables will be evaluated histopathologically by a board-certified veterinary pathologist.

Special stains may be used at the discretion of the pathologist to further characterize lesions and changes identified during routine evaluation of individual animals. Any special stains will be documented in the individual animal data. Any additional stains or evaluations, if deemed necessary by the pathologist, may be added by protocol amendment following discussion with the Study Director and in consultation the Sponsor. Efforts will be made to evaluate all protocol-required tissues microscopically; however, it is not always feasible for every protocol-required tissue to be present on every slide. Protocol-required tissues that are not examined will be documented in the histopathology data and the impact of these missing tissues on the study will be documented in the pathology report.

Images may be generated for illustration of or consultation on histological observations. These images will not be used for data generation or interpretation and will be archived and included in the Final Report.

15.3. Biodistribution/Gene Expression Analysis

~~**The tissue samples will be analyzed for vector DNA biodistribution and gene expression according to the Sponsor's requirements. All analytical work will be conducted by the Sponsor, using an analytical method developed and qualified by that laboratory that conforms to FDA guidelines but not in adherence with GLP.**~~

The samples will be archived for possible future analysis by the Sponsor. If any future analysis is performed by the Sponsor, this will be not be included in the final report.

If any possible future analysis is performed in conjunction with this study, it will not be conducted in compliance with GLPs, will not be subject to review by the Quality Assurance Unit (QAU) of that laboratory, and will not be included in the Final Report.

~~**The work performed in conjunction with this study will not be conducted in compliance with GLPs and will not subject to review by the Quality Assurance Unit (QAU) of that**~~

~~**laboratory. A Final Report will be prepared and submitted to Testing Facility for inclusion as an appendix in the main study Final Report.**~~

15.4. Splenocyte Collection and Analysis

Samples from the spleen will be collected from all animals at necropsy.

Remaining spleen after removal of a section for biodistribution and microscopic examination, as applicable, will be processed to splenocytes. Dissected spleens will be placed into prechilled tubes containing RPMI media. Samples will be stored refrigerated at 2-8ºC or on wet ice before processing.

Samples will be processed according to standard Testing Facility method, except that all washes will be performed with RPMI Media and samples will be frozen as follows:

Prepare a sufficient number of 2 mL cryovials to hold the cells at up to 2e7 cells/mL. Open the vials and add the appropriate volume of Hybridoma-Grade DMSO (Freezing media is 90% heat-inactivated FBS and 10 % DMSO). Add sufficient heat-inactivated FBS to the cell pellet to put the cells at up to 2e7/mL when added to the DMSO. Gently resuspend the pellet via pipetting and then add to the tubes containing DMSO. The act of addition and higher density of FBS will mix the FBS and DMSO yielding an evenly distributed freezing media. 1-2 gentle inversions can be used to assure even mixing. If adequate splenocytes are available, collect as many as possible and aliquot equally into as many vials as necessary with up to 2e7 cells/vial for each animal. Splenocytes will be stored in frozen storage (-60 to -90°C for initial freezing storage; transferred to Vapor Phase Liquid Nitrogen within 12 to 72 hours of original placement into frozen storage) until shipped to the Sponsor on dry ice. See Attachment B for shipping details.

~~**The splenocytes will be analyzed for T-cell responses against AAV9 and SURF1. All analytical work will be conducted by the Sponsor, using an analytical method developed and qualified by that laboratory.**~~

The samples will be archived for possible future analysis by the Sponsor. If any future analysis is performed by the Sponsor, this will be not be included in the final report.

If any possible future analysis is performed in conjunction with this study, it will not be conducted in compliance with GLPs, will not be subject to review by the Quality Assurance Unit (QAU) of that laboratory, and will not be included in the Final Report.

~~**The work performed in conjunction with this study will not be conducted in compliance with GLPs and will not subject to review by the Quality Assurance Unit (QAU) of that laboratory. A Final Report will be prepared and submitted to Testing Facility for inclusion as an appendix in the main study Final Report.**~~

16. STATISTICAL ANALYSIS

The following presents a proposed statistical analysis plan. Statistical plans are data dependent, and this analysis plan may require modification if standard data assumptions are not met. Other

conceptually equivalent statistical testing routines may also be employed at the discretion of the statistician. The actual analysis plan will be documented in the Final Report.

The raw data will be tabulated within each time interval, and the appropriate summary statistics will be calculated for each endpoint, sex, and group. For each endpoint, treatment groups will be compared to the control group using the analysis outlined below. Data for some endpoints, as indicated, will be transformed by either a log or rank transformation prior to conducting the specified analysis.

16.1. Statistical Comparisons

Control Group	Comparison Group(s)
1	2, 3, 4

16.2. Parametric/Non-Parametric

Endpoints:

- Body Weight and Body Weight Change
- Food Consumption
- Hematology
- Coagulation
- Clinical Chemistry
- Organ Weights
  - Absolute Weights
  - Relative to Body and Brain Weights

Description: The experimental unit for the analysis of food consumption will be cage, while for all other endpoints the experimental unit will be the individual animal. Food consumption will be calculated as described in Testing Facility SOP.

If the control group has a sample size less than 3, no inferential statistics will be calculated. If a particular endpoint and/or parameter within a given collection interval have the same value across all experimental units, no inferential statistics will be calculated.

Otherwise, for endpoints and/or parameters where all groups with sample sizes of 3 or greater are included. Levene's test will be used to assess homogeneity of group variances.[1,2]

The groups will be compared using an overall one-way ANOVA F-test if Levene's test is not significant or the Kruskal-Wallis test if it is significant. If the overall F-test or Kruskal-Wallis test is found to be significant, then pairwise comparisons will be conducted using Dunnett's or Dunn's test, respectively.

Results of all pair-wise comparisons will be reported at the 0.05 and 0.01 significance levels. All endpoints will be analyzed using two-tailed tests unless indicated otherwise.

17. COMPUTERIZED SYSTEMS

The following computerized systems may be used in the study. The actual computerized systems will be documented in the study data.

Critical Computerized Systems

Computer System Name	Description
DocuSign®	Collection of Part 11 compliant signature(s).
ExyLIMS	A comprehensive laboratory information management system used to manage data, including but not limited to: instrumentation, test articles, standards, and samples.
Logbook	Electronic notebook and data collection system for veterinary communications, observations, and treatments.
Deviation Information Library (DEVIL)	Deviations
Share Document Management System (SDMS)	Reporting
Provantis™	Client-server, Oracle-based system used for electronic documentation and data management from compound receipt through reporting.
SAS®	An integrated system of software products that enables a user to perform data entry, retrieval, data management, reporting, graphics, statistical analysis, and applications development.
Siemens Environmental Monitoring and Niagara Framework® Software System	Environmental monitoring, alarming, and reporting applications.

18. REGULATORY COMPLIANCE

The study will be performed in accordance with the U.S. Department of Health and Human Services, Food and Drug Administration, United States Code of Federal Regulations, Title 21, Part 58: Good Laboratory Practice for Nonclinical Laboratory Studies and as accepted by Regulatory Authorities throughout the European Union (OECD Principles of Good Laboratory Practice), Japan (MHLW), and other countries that are signatories to the OECD Mutual Acceptance of Data Agreement.

Exceptions to GLPs include the following study elements:

- Characterization of the test and control articles will be/were performed by the Sponsor or Sponsor subcontractor according to established SOPs, controls, and approved test methodologies to ensure integrity and validity of the results generated; these analyses will not be/were not conducted in compliance with the GLP or GMP regulations.
- Biodistribution, PBMC, Serum, CSF, and Splenocyte Analysis will not be conducted in compliance with GLP regulations.

19. QUALITY ASSURANCE

19.1. Testing Facility

The Testing Facility Quality Assurance Unit (QAU) will monitor the study to assure the facilities, equipment, personnel, methods, practices, records, and controls are in conformance with Good Laboratory Practice regulations. The QAU will review the protocol, conduct inspections at intervals adequate to assure the integrity of the study, and audit the Final Report to assure that it accurately describes the methods and standard operating procedures and that the reported results accurately reflect the raw data of the study.

19.2. Test Site(s)/Subcontractor(s)

For all study phase(s) inspected by test site/subcontractor QAU(s), copies of each periodic inspection report will be made available to the Study Director, Testing Facility Management, and the Testing Facility QAU.

20. AMENDMENTS AND DEVIATIONS

Changes to the approved protocol shall be made in the form of an amendment, which will be signed and dated by the Study Director. Every reasonable effort will be made to discuss any necessary protocol changes in advance with the Sponsor. The Study Director will notify the Sponsor of deviations that may result in a significant impact on the study as soon as possible.

21. RETENTION AND DISPOSITION OF RECORDS, SAMPLES, AND SPECIMENS

All study-specific raw data, electronic data, documentation, protocol, retained samples and specimens, and Interim (if applicable) and Final Reports will be archived per Testing Facility SOP. All retained materials will be archived at Charles River Laboratories-MWN, unless specified by the Sponsor. At least 1 year after issue of the Draft Report, the Sponsor will be contacted.

Samples for clinical pathology evaluations are discarded per Testing Facility SOP unless otherwise indicated in the table below.

Disposition of residual/retained analytical samples will be as described in the table below. See Attachment B for shipping details.

Disposition of Residual/Retained Samples

Sample Type	Disposition	Schedule
Residual Dosing Formulation	Returned to sponsor	Per disposition indicated in the Preparation and Disposition of Dosing Formulations section.

Records to be maintained will include, but will not be limited to, documentation and data for the following:

- Protocol, protocol amendments, and deviations
- Clinical pathology sample collection and evaluation

- Study schedule
- Study-related correspondence
- Test system receipt, health, and husbandry
- Test and control article receipt, identification, preparation, and analysis
- In-life measurements and observations
- Bioanalytical sample collection and evaluation
- Gross and microscopic observations and related data
- Organ weight measurements
- Statistical analysis results

22. REPORTING

An audited comprehensive Draft Report will be prepared following completion of the study and will be finalized following consultation with the Sponsor. The report will include all information necessary to provide a complete and accurate description of the experimental methods and results and any circumstances that may have affected the quality or integrity of the study.

The Sponsor will receive an electronic version of the Draft and Final Report provided in Adobe Acrobat PDF format (hyperlinked and searchable at Final). The PDF document will be created from native electronic files to the extent possible, including text and tables generated by the Testing Facility. Report components not available in native electronic files and/or original signature pages will be scanned and converted to PDF image files for incorporation.

A signed version of the pathology Individual Scientist report will be issued with or subsequent to the issuance of the audited Draft Report. Until the signed pathology report is issued, the unsigned Pathology Report will be marked 'Not Intended for Regulatory Submission' and will be listed on the Statement of Compliance in the audited Draft Report relating to the unsigned nature of this report. After the Pathology Report is signed, any subsequent revisions will be made in the form of an amended report, which will delineate the changes, as well as the reasons for those changes.

Reports should be finalized within 6 months of issue of the Draft Report. If the Sponsor has not provided comments to the report within 6 months of draft issue, the report will be finalized by the Testing Facility unless other arrangements are made by the Sponsor.

23. JUSTIFICATIONS AND GUIDELINES

23.1. Justification of Test System and Number of Animals

The current state of scientific knowledge and the applicable guidelines cited in this protocol do not provide acceptable alternatives, in vitro or otherwise, to the use of live animals to accomplish the purpose of this study. "The development of knowledge necessary for the improvement of the health and well-being of humans as well as other animals requires in vivo experimentation with a wide variety of animal species."[1] "Whole animals are essential in research and testing because they best reflect the dynamic interactions between the various cells, tissues, and organs comprising the human body."[2]

The rat is the usual rodent model used for evaluating the toxicity of various classes of chemicals and for which there is a large historical database.[3]

The total number of animals to be used in this study is considered to be the minimum required to properly characterize the effects of the test article and has been designed such that it does not require an unnecessary number of animals to accomplish its objectives.

23.2. Justification of Route and Dose Levels

Intrathecal injection is the intended route of administration of this test article in humans.

The dose levels were selected based on information provided by the Sponsor that the target dose conferred efficacy to SURF1 knock-out mice, was found to be well-tolerated in mice up to 10 months post-injection, models a relevant dose to human patients, and in an attempt to produce graded responses to the test article. The high-dose level (effectively above a maximum feasible human dose) may produce some toxic effects, but not excessive lethality that would prevent meaningful evaluation. The mid-dose level (target dose) and low-dose level are not expected to produce observable indications of toxicity.

23.3. Guidelines for Study

The design of this study was based on the study objective(s), the overall product development strategy for the test article, and the following study design guidelines:

- FDA Guidance for Industry. *Preclinical Assessment of Investigational Cellular and Gene Therapy Products.*
- FDA Draft Guidance for Industry. *Human Gene Therapy for Rare Diseases*.
- ICH Harmonised Tripartite Guideline S6 (R1). *Preclinical Safety Evaluation of Biotechnology-Derived Pharmaceuticals.*

24. ANIMAL WELFARE

This study will comply with all applicable sections of the Final Rules of the Animal Welfare Act regulations (Code of Federal Regulations, Title 9), the *Public Health Service Policy on Humane Care and Use of Laboratory Animals* from the Office of Laboratory Animal Welfare, and the *Guide for the Care and Use of Laboratory Animals* from the National Research Council.[4,5] The protocol and any amendments or procedures involving the care or use of animals in this study will be reviewed and approved by the Testing Facility Institutional Animal Care and Use Committee before the initiation of such procedures.

If an animal is determined to be in overt pain/distress, or appears moribund and is beyond the point where recovery appears reasonable, the animal will be euthanized for humane reasons in accordance with the *American Veterinary Medical Association (AVMA) Guidelines on Euthanasia* and with the procedures outlined in the protocol.[6]

By approving this protocol, the Sponsor affirms that there are no acceptable non-animal alternatives for this study, that this study is required by a relevant government regulatory agency(ies) and that it does not unnecessarily duplicate any previous experiments.

24.1. Institutional Animal Care and Use Committee Approval

The protocol and any amendment(s) or procedures involving the care and use of animals in this study will be reviewed and approved by CR-MWN Institutional Animal Care and Use Committee (IACUC) before conduct. During the study, the care and use of animals will be conducted with guidance from the guidelines of the USA National Research Council.

25. REFERENCES

1. *Principles for the Utilization and Care of Vertebrate Animals Used in Testing, Research, and Training*, Federal Register, 1985 May 20; 50(97).
2. *Position Statement on the Use of Animals in Research*, 1993 Feb 26; NIH Guide 22(8).
3. *Guidance for Industry, Investigators, and Reviewers: Exploratory IND Studies.* US FDA Center for Drug Evaluation and Research (CDER), 2006 Jan.
4. Office of Laboratory Animal Welfare. *Public Health Services Policy on Humane Care and Use of Laboratory Animals*. Bethesda, MD: National Institutes of Health. Current edition.
5. National Research Council. *Guide for the Care and Use of Laboratory Animals*. Washington, DC: National Academy Press. Current edition.
6. American Veterinary Medical Association. *AVMA Guidelines on Euthanasia*. Current edition.

Appendix 1

ATTACHMENT A

Tissue Weighing, Collection, Processing and Evaluation Table

Organ	Weigh	Macroscopic Evaluation and Collection	Histology Processing and Microscopic Evaluation	Biodistribution Analysis	Remaining Frozen Tissue to Archive
Animal ID	-	X	-	-	-
Artery, aorta	-	X	-	-	-
Body cavity, nasal	-	X	-	-	-
Bone marrow, sternum	-	X	-	-	-
Bone marrow smear (humerous)	-	X[a]	-	-	-
Bone, femur	-	X (1) (Left)	-	-	X (1) (Right)
Bone, sternum	-	X	-	-	-
Brain	X	X	X (Left)	X (Right) (forebrain, midbrain, and hindbrain)	X (Right) (Forebrain, midbrain, and hindbrain)
Epididymis	X (2)	X (2)[b]	-	-	-
Esophagus	-	X	-	-	-
Eye	-	X (2)[b]	X (1) (Left)	-	X (1) (Right)
Ganglion, dorsal root, cervical	-	X (2)	X (1 pair)	X (1 pair)	X (1 pair)
Ganglion, dorsal root, thoracic	-	X (2)	X (1 pair)	-	X (1 pair)
Ganglion, dorsal root, lumbar	-	X (2)	X (1 pair)	X (1 pair)	X (1 pair)
Gland, adrenal	X (2)	X (2)	-	-	-
Gland, clitoral	-	X (2)	-	-	-
Gland, lacrimal	-	X (2) (extra-orbital)	-	-	-
Gland, Harderian	-	X (2)	-	-	-
Gland, mammary	-	X	-	-	-
Gland, parathyroid	-[d]	X (2)	-	-	X (Right)[e]
Gland, pituitary	X	X	-	-	-
Gland, preputial	-	X (2)	-	-	-
Gland, prostate	X	X	-	-	-
Gland, salivary, submandibular	X (Right)	X	-	-	X (Right)
Gland, salivary, sublingual	-	X (2)	-	-	X (Right)
Gland salivary, parotid	-	X (2)	-	-	-
Gland, seminal vesicle	-	X (2)	-	-	-
Gland, thyroid	X (Right)	X (2)	-	-	X (1) (Right)
Gland, Zymbal's	-	X (2)	-	-	-
Gut-associated lymphoid tissue[c]	-	X	-	-	-
Heart	X	X	X	X (Left Ventricle)	X

Appendix 1

Organ	Weigh	Macroscopic Evaluation and Collection	Histology Processing and Microscopic Evaluation	Biodistribution Analysis	Remaining Frozen Tissue to Archive
Joint, femorotibial	-	X (1)	-	-	-
Kidney	X (2)	X (2)	X (Left)	X (Right)	X (Right)
Large intestine, cecum	-	X	-		
Large intestine, colon	-	X	-	-	X
Large intestine, rectum	-	X	-	-	-
Larynx	-	X	-	-	-
Liver	X	X	X	X (Left lateral lobe)	X
Lung	X	X	X	-	X
Lymph node(s) draining administration site(s): Iliac	-	X (2)	X (1) (Left)	-	X (1) (Right)
Lymph node, mandibular	-	X (2)	X (1) (Left)	-	X (1) (Right)
Lymph node, mesenteric	-	X	X	-	X
Muscle, skeletal (bicep femoris and gastrocnemius)	-	X (2)	X (1) (Left)	X (1) (Right)	X (1) (Right)
Nerve, optic	-	X (2)[b]	X (Left)	-	X (Right)
Nerve, sciatic	-	X (2)	X (1) (Left)	X (1) (Right)	X (1) (Right)
Nerve, tibial	-	X (2)	X (1) (Left)	-	X (1) (Right)
Ovary	X (2)	X (2)	X (Left)	X (Right)	X (Right)
Oviduct	-	X (2)	-	-	X (Right)
Pancreas	-	X	X	-	X
Skin	-	X	-	-	X
Small intestine, duodenum	-	X	-	-	X
Small intestine, ileum	-	X	-	-	X
Small intestine, jejunum	-	X	-	-	X
Spinal cord, cervical	-	X	X	X	X
Spinal cord, thoracic	-	X	X	-	X
Spinal cord, lumbar	-	X	X	X	X
Spleen	X	X	X	X	-
Stomach	-	X	-	-	X
Testis	X (2)	X (2)[b]	X (Left)	X (Right)	X (Right)
Thymus	X	X	X	X	X
Tongue	-	X	-	-	-
Trachea	-	X	-	-	-
Ureter	-	X (2)	-	-	-
Urinary bladder	-	X	-	-	X
Uterus/Cervix	X	X	-	-	X
Vagina	-	X	-	-	-

Appendix 1

Organ	Weigh	Macroscopic Evaluation and Collection	Histology Processing and Microscopic Evaluation	Biodistribution Analysis	Remaining Frozen Tissue to Archive

X = Procedure to be conducted. - = Not applicable. (1) = one side. (2) = both sides.
Macroscopic abnormalities in the organs listed and in other organs will be sampled at necropsy, processed for histology and examined microscopically.
*If tissue quantity is limited due to size, sample priority will be as follows: histology > Biodistribution > Remaining Frozen Tissue
[a] Two bone marrow smears will be collected from the humerus at scheduled and unscheduled necropsies (for possible examination). Smears will not be collected from animals that are found dead or from animals that were euthanized moribund and then stored in the refrigerator prior to necropsy. Bone marrow smears are allowed to air dry and are not fixed in formalin.
[b] Eyes and optic nerves are preserved in Davidson's fixative. Testes and epididymides are preserved in modified Davidson's fixative.
[c] From small intestine: Peyer's patch or solitary lymphoid follicle.
[d] Weigh with gland, thyroid.
[e] Collected with gland, thyroid.

Appendix 1

ATTACHMENT B

Shipment of Samples

Samples will be shipped on Monday through Wednesday for next day delivery for domestic shipments and expedited delivery for International shipments using the Conditions for Shipment(s) listed below.

<table>
<tr><th>Sample Matrix</th><th>Sample Type</th><th>Aliquot of Sample</th><th>Proposed Shipment</th><th>Conditions for Shipment</th><th>Recipient/Address</th></tr>
<tr><td>NA</td><td>Remaining Unused Bulk Test Article</td><td>NA</td><td>Following Completion of in-life procedures</td><td rowspan="3">on dry ice</td><td>Attn: Steven Gray, PhD
University of Texas Southwestern Medical Center
6000 Harry Hines Blvd
University of Texas Southwestern Medical Center
Dallas, TX 75390
Tel: 214-648-0670
E-mail: steven.gray@UTsouthwestern.edu</td></tr>
<tr><td>Blood, CSF, tissue samples</td><td>Bioanalytical Samples</td><td>All</td><td>Following collection of terminal samples</td><td rowspan="2">Attn: Juan A. Rodriguez
University of Texas Southwestern
5901 Forest Park Rd, NA2.508
Receiving – North Campus Dock
Dallas, TX 75390
Tel: 281-380-5165
E-mail: Juan.Rodriguez@UTSouthwestern.edu</td></tr>
<tr><td>NA</td><td>Splenocyte Samples</td><td>All</td><td>Following collection of terminal samples</td></tr>
<tr><td>Tissue</td><td>Remaining frozen tissue to archive</td><td>All</td><td>Following issuance of current amendment</td><td>On dry ice</td><td>Attn : Eric Shih
UT Southwestern Med. Ctr.
5901 Forest Park Rd., NA2.508
Dallas, Tx., 75390
Tel : 214-648-0671
E-mail: Eric.Shih@UTSouthwestern.edu</td></tr>
<tr><td>Slides</td><td>Histopathology slides</td><td>NA</td><td>Following slide creation</td><td>ambient</td><td>Attn: Jennifer Creager
TPS [a StageBio Company]
8415 Progress Drive, Suite Q
Frederick, MD 21701
Tel: 301-378-0505
E-mail: jcreager@stagebio.com</td></tr>
</table>

Appendix 1

Sample Matrix	Sample Type	Aliquot of Sample	Proposed Shipment	Conditions for Shipment	Recipient/Address
Slides	Histopathology slides	NA	Following review by anatomic pathologist	ambient	Jacqueline Brassard DVM, PhD, DACVP<br>Brassard Toxicologic Pathology Consultancy Corp.<br>163 Mountain View Dr.<br>Tustin, California 92780<br>Mobile phone: 314-397-3208<br>dr.jbrassard@gmail.com
Not applicable	Return of Pathology slides after Peer Review	Not applicable	Return after completion of peer review	Ambient	Charles River Laboratories, Inc.<br>ATTN: Path Services, Room LS-30<br>54943 North Main Street<br>Mattawan, MI<br>Tel: 269-668-3336 ext. 1136<br>E-mail: kellie.howe@crl.com

Appendix 1

SPONSOR APPROVAL

The protocol amendment was approved by the Sponsor by e-mail on the date designated below. The correspondence giving approval will be archived, as appropriate with other Sponsor communications.

13 Mar 2023
Date of Sponsor Approval

Appendix 1

TESTING FACILITY APPROVAL

All electronic signatures appear at the end of the document upon finalization.

Appendix 1

SIGNATURE(S) FOR DOCUMENT: 2954-001 - Protocol Amendment-12

**Study Director Approval:**	I approve this document.
Name:	**Davis, Sarah (Mattawan)**
Davis, Sarah (Mattawan)	13-Mar-2023 16:48:11 (UTC+00:00)
Electronically Signed in M-Files	Timestamp

Appendix 1

FINAL PROTOCOL

Testing Facility Study No. 2954-001

Sponsor Reference No. UTSW.Gray-002

A Single Dose Toxicity Study of AAV9/SURF1 Administered by Intrathecal Injection in Rats

Status: GLP

SPONSOR:
University of Texas Southwestern Medical Center
5323 Harry Hines Blvd
Dallas, TX 75390
USA

TESTING FACILITY:
Charles River Laboratories, Inc.
54943 North Main Street
Mattawan, MI 49071
USA

TABLE OF CONTENTS

1. OBJECTIVE(S) 4
2. PROPOSED STUDY SCHEDULE 4
3. SPONSOR 4
4. RESPONSIBLE PERSONNEL 5
5. TEST MATERIALS 6
6. DOSE FORMULATION AND ANALYSIS 7
7. TEST SYSTEM 8
8. HUSBANDRY 9
9. EXPERIMENTAL DESIGN 11
10. SURGICAL ANIMAL PREPARATION 11
11. IN-LIFE PROCEDURES, OBSERVATIONS, AND MEASUREMENTS 12
12. CLINICAL PATHOLOGY 13
13. BIOANALYTICAL EVALUATION 14
14. TERMINAL PROCEDURES 15
15. HISTOLOGY AND MICROSCOPIC EVALUATION 18
16. STATISTICAL ANALYSIS 19
17. COMPUTERIZED SYSTEMS 21
18. REGULATORY COMPLIANCE 22
19. QUALITY ASSURANCE 22
20. AMENDMENTS AND DEVIATIONS 23
21. RETENTION AND DISPOSITION OF RECORDS, SAMPLES, AND SPECIMENS 23
22. REPORTING 23
23. JUSTIFICATIONS AND GUIDELINES 24
24. ANIMAL WELFARE 25
25. REFERENCES 25
TESTING FACILITY APPROVAL 27
SPONSOR APPROVAL 28
ATTACHMENT A 29

Appendix 1

ATTACHMENT B 32

Appendix 1

1. OBJECTIVE(S)

The objective of this study is to characterize the toxicity, biodistribution, and gene expression of AAV9/SURF1 for the treatment of SURF1 Leigh Syndrome.

2. PROPOSED STUDY SCHEDULE

Proposed study dates are listed below. Actual dates will be included in the Final Report.

Experimental Starting Date:	18 Aug 2020<br>(First date of study-specific data collection)
Experimental Completion Date:	To be included in the Final Report<br>(Last date on which data are collected)
Draft Report:	Approximately 10 weeks<br>(Following last day of necropsy)
Final Report:	The date on which the Study Director signs the final report.

3. SPONSOR

Role	Name/Contact Information
Sponsor Representative	Steven Gray, PhD<br>University of Texas Southwestern Medical Center<br>5323 Harry Hines Blvd<br>University of Texas Southwestern Medical Center<br>Dallas, TX 75390<br>Tel: 214-648-0670<br>E-mail: steven.gray@UTsouthwestern.edu
Alternate Sponsor Representative	Samuel Hughes<br>University of Texas Southwestern Medical Center<br>5323 Harry Hines Blvd<br>University of Texas Southwestern Medical Center<br>Dallas, TX 75390<br>Tel: 214-648-2926<br>E-mail: Samuel.hughes@UTSouthwestern.edu

Appendix 1

4. RESPONSIBLE PERSONNEL

Role/Phase	Quality Assurance Unit	Name/Contact Information
Study Director	Charles River	Barak W. Gunter, PhD<br>Tel: 269-668-3336 ext. 1099<br>E-mail: barak.gunter@crl.com
Alternate Contact	Charles River	Sarah M. Davis, BS, LATG<br>Tel: 269-668-3336 ext. 1250<br>E-mail: sarah.davis2@crl.com
Testing Facility Management	Charles River	Scott T. Wilson, BS, LAT<br>Tel: 269-668-3336 ext. 1610<br>E-mail: scott.wilson@crl.com
Testing Facility QAU	Charles River	Janis Kissel, BS, RQAP-GLP<br>Tel: 269-668-3336, ext. 3170<br>E-mail: janis.kissel@crl.com
**Individual Scientist (IS)**		
Anatomic Pathologist	Charles River	Will be included in the Final Report
Clinical Pathologist	Charles River	Will be included in the Final Report
**Principal Investigator (PI)**		
Serum, CSF, and Tissue Biodistribution Analysis	Test Site	Steven Gray, PhD<br>University of Texas Southwestern Medical Center<br>5323 Harry Hines Blvd<br>University of Texas Southwestern Medical Center<br>Dallas, TX 75390<br>Tel: 214-648-0670<br>E-mail: steven.gray@UTsouthwestern.edu

Each IS and PI is required to report all deviations or other circumstances that could affect the quality or integrity of the study to the Study Director in a timely manner for authorization/acknowledgement. Each IS and PI will provide a report addressing their assigned phase of the study, which will be included as an appendix to the Final Report.

The PI Phase Report will include the following:

- A Statement of Compliance (if the applicable phase is GLP)

Appendix 1

- A QA Statement (for Sponsor designated PI or for Testing Facility designated PI if audited by a QAU other than that of the Testing Facility and if the applicable phase is GLP)
- The archive site for all records, samples, specimens and reports generated from the phase or segment (alternatively, details regarding the retention of the materials may be provided to the Study Director for inclusion in the Final Report)
- A listing of critical computerized systems used in the conduct and/or interpretation of the assigned study phase

5. TEST MATERIALS

5.1. Test Article Characterization

The Sponsor will provide to the Testing Facility documentation of the identity, strength, purity, composition, and stability for the test article. A Certificate of Analysis or equivalent documentation will be provided for inclusion in the Final Report.

The Sponsor has appropriate documentation on file concerning the method of synthesis, fabrication or derivation of the test article, and this information is available to the appropriate regulatory agencies should it be requested.

5.2. Test Article Identification

**Identification:**	AAV9/SURF1
**Alternate Identification:**	scAAV9/CBh-hsaSURF1opt-BGHpA
**Batch/Lot No.:**	To be included in the Final Report
**Expiration/Retest Date:**	To be included in the Final Report
**Physical Description:**	To be included in the Final Report
**Purity:**	To be included in the Final Report
**Correction Factor:**	NA
**Concentration:**	1E14 vg/mL X
**Storage Conditions:**	Frozen (-60 to -90°C)
**Provided by:**	Sponsor
**Test Article Contact:**	Samer Dahshi<br>Tel: 214-648-7164<br>E-mail: samer.dahshi@UTSouthwestern.edu

NA = Not applicable.

5.3. Vehicle Information

**Identification:**	PBS containing 5% D-sorbitol and 0.001% pluronic F-68
**Alternate Identification:**	Diluent
**Storage Conditions:**	Frozen (-60 to -90°C)

**Characterization:**	Documentation of the strength, purity, composition, stability, and other pertinent information on each batch of vehicle, will be limited to that information listed on the label of this commercially available material, unless otherwise noted.

5.4. Reserve Samples

A reserve sample from each lot of test and vehicle articles used in this study will be collected and stored at the Testing Facility in a secure area with the appropriate environmental controls

5.5. Test Article Inventory and Disposition

Records of the receipt, distribution, storage, and disposition of test materials (including empty containers of Sponsor-provided materials) will be maintained until study finalization.

The Sponsor will be contacted for proper disposition of materials (retain/ship/discard) after completion of the in-life phase of the study, and following confirmation that these materials are not assigned to other studies.

5.6. Safety

A Safety Data Sheet (SDS), or equivalent documentation, will be provided by the Sponsor (if available). It is the responsibility of the Sponsor to notify the Testing Facility of any special handling requirements of the test article. Otherwise routine safety precautions will be followed. Appropriate gloves, safety glasses and arm covers will be worn by individuals working with neat test material(s) or formulations.

6. DOSE FORMULATION AND ANALYSIS

6.1. Preparation of Formulations

Dose formulations will be divided into aliquots where required to allow to be dispensed on each dosing occasion.

Preparation Details

Dose Formulation	Frequency of Preparation	Storage Conditions
Vehicle	Used as received	Same as Test Article
Dose Formulations	Prior to dosing[a]	Refrigerated (2 to 8°C)

[a] 60 days of established stability at refrigerated (2 to 8°C) storage conditions has been established.

Any residual volumes from each dosing occasion will be retained and stored refrigerated (2 to 8°C) unless otherwise requested by the Study Director. These retained volumes can be used on subsequent dosing days.

6.2. Preparation Details

The stock solution will be diluted as needed to prepare all dose concentrations using the Sponsor-supplied instructions as a guide.

Appendix 1

6.3. Sample Collection and Analysis

The test and control articles will be used as received from the Sponsor; therefore, samples for dose formulation analysis will not be collected by the Testing Facility.

7. TEST SYSTEM

Species:	Rat
Strain:	CD® [Crl:CD®(SD)]
Condition:	Purpose-bred, naïve
Source:	Charles River Laboratories, Inc., Raleigh, NC or Kingston, NY<br>The source used will be documented in the raw data.
Number Ordered:	
Male:	66
Female:	66
Number on Study:	
Male:	60
Female:	60
Expected Age at Arrival:	Ordered to be 6 weeks of age at arrival
Expected Weight at Arrival:	Commensurate with age; males will generally weigh 215 to 260g and females will generally weigh 140 to 185g, as measured within 3 days of arrival. The actual range will be documented in the data.

The actual age and weight of animals received will be listed in the Final Report.

7.1. Animal Identification

Method:	Each animal will be assigned an animal number to be used in Provantis™ and will be implanted with a microchip bearing a unique identification number. The individual animal number, implant number, and the Testing Facility study number will comprise a unique identification for each animal. The animal cage will be identified by the study number, animal number, group number, and sex.

7.2. Environmental Acclimation

Duration:	At least 1 week

Appendix 1

Details:	During this acclimation period, all animals will be observed daily for any clinical signs of disease, and all animals will be given a detailed clinical examination within 3 days of the first dose administration.

7.3. Selection, Assignment, Replacement and Disposition of Animals

Assignment and Randomization:	Animals will be randomly assigned to groups upon receipt. Males and females will be randomized separately.<br>All animals with any evidence of disease or physical abnormalities will not be selected for study.
Replacement:	Before the initiation of dosing, any assigned animals considered unsuitable for use in the study will be replaced by alternate animals obtained from the same shipment and maintained under the same environmental conditions.<br>After initiation of dosing, study animals may be replaced during the replacement period with alternate animals in the event of accidental injury, non-test article-related health issues, or similar circumstances.<br>Alternate animals may be used as replacements per Testing Facility SOP.
Disposition:	Extra animals obtained for this study, but not placed on study, will be either transferred to a Testing Facility stock or training colony, or euthanized and discarded.<br>The disposition of all animals will be documented in the study records.

8. HUSBANDRY

8.1. Housing

Housing:	Pair-housed, when possible (animals may be housed 2 to 3/cage during acclimation and in-life depending on study design).<br>Housing set-up is as specified in the USDA Animal Welfare Act (9 CFR, Parts 1, 2 and 3) and as described in the *Guide for the Care and Use of Laboratory Animals*. Animals will be separated during designated procedures/activities or will be separated as required for monitoring and/or health purposes, as deemed appropriate by Study Director and/or Clinical Veterinarian.
Caging:	Solid bottom cages with nonaromatic bedding. The bedding will be from an approved supplier and documented in the study data.

Appendix 1

8.2. Animal Enrichment

Supplemental Enrichment:	Animal enrichment will be provided according to Testing Facility SOP.

8.3. Environmental Conditions

Temperature and Humidity:	Temperature and humidity will be maintained according to Testing Facility SOP.
Lighting:	Fluorescent lighting will be provided via an automatic timer for approximately 12 hours per day. On occasion, the dark cycle may be interrupted intermittently due to study-related activities.

8.4. Food

Diet:	The basal diet will be block Lab Diet® Certified Rodent Diet #5002, PMI Nutrition International, Inc.
Frequency:	Ad libitum, except during designated procedures
Analysis:	Results of analysis for nutritional components and environmental contaminants are provided by the supplier and are on file at the Testing Facility.<br>There are no known contaminants in the food that would interfere with this study.

8.5. Water

Type:	Tap water
Frequency:	Supplied ad libitum to all animals via an automatic water system unless otherwise indicated.
Analysis:	There are no known contaminants in the water that would interfere with this study. The drinking water used will be monitored for specified contaminants at periodic intervals according to Testing Facility SOP.

8.6. Veterinary Care

Veterinary care will be available throughout the course of the study and animals will be examined by the veterinary staff as warranted by clinical signs or other changes. In the event that animals show signs of illness or distress, the responsible veterinarian may make initial recommendations about treatment of the animal(s) and/or alteration of study procedures, which must be approved by the Study Director (or scientific designee). Treatment of the animal(s) for minor injuries or ailments may be approved without prior consultation with the Sponsor representative when such treatment does not impact fulfillment of the study objectives. If the condition of the animal(s) warrants significant therapeutic intervention or alterations in study

procedures, the Sponsor representative will be contacted, when possible, to discuss appropriate action. If the condition of the animal(s) is such that emergency measures must be taken, the Study Director (or scientific designee) and/or attending veterinarian will attempt to consult with the Sponsor representative prior to responding to the medical crisis, but the Study Director (or scientific designee) and/or veterinarian has authority to act immediately at his/her discretion to alleviate suffering. The Sponsor representative will be fully informed of any such events.

9. EXPERIMENTAL DESIGN

Group	Treatment	Dose Level (vg)	Dose Volume (µL)	Dose Concentration (vg/µL)	No. of Animals Day 1 Dose M	F	Day 8 Necropsy M	F	Day 29 (± 1) Necropsy M	F	Day 91 (± 3) Necropsy M	F
1	Vehicle	0	60	0	15	15	5	5	5	5	5	5
2	Low Dose	$0.67x10^{12}$	20	$0.33x10^{11}$	15	15	5	5	5	5	5	5
3	Mid Dose	$2x10^{12}$	20	$1x10^{11}$	15	15	5	5	5	5	5	5
4	High Dose	$6x10^{12}$	60	$1x10^{11}$	15	15	5	5	5	5	5	5

No. = Number

9.1. Administration of Test Article(s)

Route: Intrathecal injection

Frequency and Duration: Once on Day 1

Dose Level: 0, $0.67x10^{12}$, $2x10^{12}$, or $6x10^{12}$ vg

10. SURGICAL ANIMAL PREPARATION

10.1. Pre-operative Procedures

Details: Anesthesia will be induced and maintained as indicated in Testing Facility SOP.

10.2. Surgical Procedure

The procedures outlined below are intended to serve as guidance for successful dose administration. Minor alterations in the surgical procedures may occur as necessary at the discretion of the surgeon or Study Director and will be documented in the study data and will not be considered a protocol deviation.

Animals will be anesthetized, placed in a ventral recumbency, and prepped using aseptic technique. The dose administration will be performed using a gas tight Hamilton syringe with a disposable needle. Under fluoroscopic guidance, if needed, a needle will be inserted into the lumbar cistern. The location of the needle may vary based on the discretion of the surgeon and final needle location will be recorded. Once the needle is appropriately placed, the animals will be dosed with test article or vehicle and flushed with vehicle over 30±5 seconds for groups 1 and

4, and 10±5 seconds for groups 2 and 3. Upon completion of the dose, the needle will be removed and the animal will then be allowed to recover.

10.3. Postoperative Procedures

Details: Postoperative procedures will be conducted in accordance with Testing Facility SOP.

11. IN-LIFE PROCEDURES, OBSERVATIONS, AND MEASUREMENTS

Standard In-life Assessments

Parameter	Population(s)	Frequency (minimum required)	Comments
Mortality/Cageside Observations	All surviving animals	At least twice daily[a,b] (morning and afternoon) beginning upon arrival through termination/release.	Animals will be observed within their cage unless necessary for identification or confirmation of possible findings.<br><br>Animals will be observed for morbidity, mortality, injury, and availability of food and water. Any animals in poor health will be identified for further monitoring and possible euthanasia.
Detailed Clinical Observations	All Main Study animals	Weekly; from at least Week -1 and throughout the study.[c]	Animals are removed from the cage.<br><br>Observations will include, but will not be limited to, evaluation of the skin, fur, eyes, ears, nose, oral cavity, thorax, abdomen, external genitalia, limbs and feet, respiratory and circulatory effects, autonomic effects such as salivation, nervous system effects including tremors, convulsions, reactivity to handling, unusual behavior, and palpation of tissue masses.
Individual Body Weights	All Main Study animals	Within 3 days of arrival, Day -1, and once weekly during the terminal and recovery periods.	Fasted weight on the day of necropsy. Not collected from animals found dead.
Food Consumption	All Main Study animals	Weekly.[d]	Quantitatively measured

Appendix 1

Parameter	Population(s)	Frequency (minimum required)	Comments

[a] Procedures on alternate animals will be conducted per Testing Facility SOP.
[b] Except on days of receipt and necropsy where frequency will be at least once daily.
[c] For observations that cannot be attributed to an individual animal due to social housing (e.g., watery feces), the observation will be noted to each animal in the socialized group.
[d] For observations of reduced appetite that cannot be attributed to an individual animal due to social housing, the observations will be noted for each animal in the socialized group.

12. CLINICAL PATHOLOGY

12.1. Sample Collection

Clinical Pathology Sample Collection

Group Nos.	Time Point(s)	Hematology	Coagulation	Clinical Chemistry
All Surviving Animals	At necropsy	X	X	X
Unscheduled Euthanasia (when possible)	See the Unscheduled Euthanasia section of this protocol.			
**Volume (mL)[a]:**	NA	1 mL	1.2 mL	1.3 mL
**Fasting Required:**	Free access to drinking water but will be fasted overnight (at least 8 hours) prior to blood collection.			
**Anticoagulant:**	NA	K_2EDTA	Sodium Citrate	Serum Gel Separator
**Special Requirements:**	NA	NA	NA	NA
**Processing:**	NA	None	Plasma	Serum

X = Sample to be collected; NA = Not applicable; hr = hour; pre = predose; post= postdose.
[a] Additional blood samples may be obtained (e.g. due to sample quality) if permissible sampling frequency and blood volume are not exceeded.

Blood Sample Collection Method: Cardiac puncture or vena cava after carbon dioxide inhalation

The following clinical pathology tests will be conducted on available samples, as survival allows, and the data will be interpreted by a Clinical Pathologist.

12.2. Hematology

Hematology Parameters

Leukocyte count (total and absolute differential)<br>Erythrocyte count<br>Hemoglobin<br>Hematocrit<br>Absolute reticulocytes	Mean corpuscular hemoglobin, mean corpuscular volume, mean corpuscular hemoglobin concentration (calculated)<br>RDW<br>Platelet count<br>Blood smear (preserve and stain)[a]

[a] Blood smear review may be performed on select animals per Testing Facility SOP.

12.3. Coagulation

Coagulation Parameters

Prothrombin time<br>Activated partial thromboplastin time	Fibrinogen

12.4. Clinical Chemistry

Clinical Chemistry Parameters

Alkaline phosphatase	Globulin and A/G (albumin/globulin) ratio (calculated)
Total bilirubin (with direct bilirubin if total bilirubin exceeds 1 mg/dL)	Glucose
	Total cholesterol
Aspartate aminotransferase	Triglycerides
Alanine aminotransferase	Electrolytes (sodium, potassium, chloride)
Urea nitrogen	Calcium
Creatinine	Phosphorus
Total protein	Sample quality
Albumin	

13. BIOANALYTICAL EVALUATION

13.1. Serum Sample Collection

Animals/Interval:	All animals prior to their scheduled necropsy
Method/Comments:	Cardiac puncture or vena cava after carbon dioxide inhalation
Volume (mL):	1 mL
Anticoagulant:	None, Serum Gel Separator
Whole Blood Storage:	Ambient
Container Label Requirements:	Study number, animal number, matrix of sample, interval and timepoint, analysis type, and aliquot number.
Processing:	Serum<br>The final processed sample(s) will be divided into 2 approximately equal aliquots
Final Storage Temperature:	Frozen (-60 to -90°C)

13.2. Serum Sample Shipping and Analysis

Analysis Performed By:	The serum will be analyzed for AAV9 concentrations. All analytical work will be conducted by the Sponsor, using an analytical method developed and qualified by that laboratory.

Regulatory Requirements:	The work performed in conjunction with this study will not be conducted in compliance with GLPs and will not be subject to review by the Quality Assurance Unit (QAU) of that laboratory. A Final Report will be prepared and submitted to Testing Facility for inclusion as an appendix in the main study Final Report.
Sample Shipping Contact:	All samples to be analyzed will be shipped to the designated Test Site, see Attachment B for shipping details.

13.3. Cerebral Spinal Fluid (CSF)

13.4. CSF Sample Collection

Animals and Intervals:	All animals prior to their scheduled necropsy
Collection Site:	Cisterna magna
Volume/Sample:	Maximum obtainable clean sample
Anticoagulant:	None
Sample Storage:	Stored on ice block or wet ice following collection until processing and final storage.
Container Label Requirements:	Study number, animal number, matrix of sample, interval and timepoint, analysis type, and aliquot number.
Processing:	The CSF sample(s) will be divided into 2 approximately aliquots
Final Storage Temperature:	Frozen (-60 to -90°C)

13.5. CSF Sample Shipping and Analysis

Analysis Performed By:	The CSF will be archived for possible future analysis. If any future analysis is performed by the Sponsor, this will be not be included in the final report.
Regulatory Requirements:	If any possible future analysis is performed in conjunction with this study, it will not be conducted in compliance with GLPs, will not be subject to review by the Quality Assurance Unit (QAU) of that laboratory, and will not be included in the Final Report.
Sample Shipping Contact:	All samples will be shipped to the designated Test Site, see Attachment B for shipping details.

14. TERMINAL PROCEDURES

Terminal procedures are summarized in the following tables:

Appendix 1

Terminal Procedures

Group No.	Necropsy Procedures			Histology Processing	Microscopic Evaluation
	Necropsy	Tissue Collection	Organ Weights		
Found dead or unscheduled euthanasia	X	Full List[a]	NA	Full List[a]	Full List[a]

Main Study Animals

Group No.	Scheduled Euthanasia Day	Necropsy Procedures			Histology Processing	Microscopic Evaluation
		Necropsy	Tissue Collection	Organ Weights		
1-4	8, 29(±1), or 91(±3)	X	Full List[a]	Full List[a]	Full List[a]	Full List[a]

Terminal Procedure Tables Footnotes:

X = Procedure to be conducted; NA = Not applicable.

"Histology Processing"= embedded in paraffin, sectioned, mounted on glass slides, and stained with hematoxylin and eosin.

[a] See Tissue Weighing, Collection, Processing and Evaluation Table for list of tissues applicable to each procedure.

14.1. Method of Euthanasia

Euthanasia will be by carbon dioxide inhalation followed by a Testing Facility SOP approved method to ensure death, e.g. exsanguination.

14.2. Unscheduled Euthanasia

Moribund animals will be subject to Testing Facility SOP criteria and procedures. If possible, the samples below will be collected from animals euthanized in extremis, following veterinary consultation. A veterinary consultation is not required if the samples are collected following anesthesia or euthanasia. Blood collection methods utilized for animals euthanized in extremis may include suitable methods other than those presented in the respective blood collection section(s) of this protocol. Samples below are listed in order of collection priority.

Sample Type	Groups	Volume	Anticoagulant
Clinical Chemistry	All	1.3 mL	Serum Gel Separator
Hematology		1 mL	K_2EDTA
Coagulation		1.2 mL	Sodium Citrate
Serum		1 mL	Serum Gel Separator
CSF		Maximum Obtainable	None

Necropsy examinations will be performed 7 days a week. Animals that are found dead or euthanized in extremis after regular working hours will be refrigerated overnight and necropsies performed at the start of the next day.

14.3. Scheduled Euthanasia

Main Study animals surviving until scheduled euthanasia will be euthanized by the methods described above. When possible, the animals will be euthanized rotating across dose groups such that similar numbers of animals from each group, including controls, will be necropsied throughout the day.

14.4. Necropsy

Animals as detailed in the Terminal Procedures table will be subjected to a complete necropsy examination, which will include evaluation of the carcass and musculoskeletal system; all external surfaces and orifices; cranial cavity and external surfaces of the brain; and thoracic, abdominal, and pelvic cavities with their associated organs and tissues.

All animals will be examined carefully for external abnormalities including palpable masses.

Images may be generated for illustration of or consultation on gross observations. These images will not be used for data generation or interpretation and will not be archived or included in the Final Report.

14.5. Organ Weights

Body weight and the organ weights identified in the Tissue Weighing, Collection, Processing and Evaluation table will be recorded for all animals at the scheduled necropsies, and appropriate organ weight ratios will be calculated (relative to body and brain weights). Paired organs will be weighed together. Organ weights will be collected per Testing Facility SOP.

14.6. Tissue Collection and Preservation for Histopathology Analysis

Representative samples of tissues will be collected and preserved in 10% neutral buffered formalin, as detailed in the Terminal Procedures and Tissue Weighing, Collection, Processing and Evaluation tables. Additional tissue samples may be collected to elucidate abnormal findings.

For the brain, standard collection and trimming will be performed according to Testing Facility SOP. The sections will include the following: forebrain, midbrain, hindbrain (including brainstem and cerebellum).

14.7. Tissue Collection for Biodistribution/Gene Expression Analysis

Tissues listed in Attachment A will be evaluated for biodistribution/gene expression by the Sponsor. Tissues will be collected and flash frozen in liquid nitrogen. Each tissue will be split into 2 tubes when possible, with approximately 50 mg per tube. See Attachment B for shipping details. Remaining samples will be archived by the sponsor.

Samples will not be collected for animals euthanized in extremis, or animals found dead.

These samples will be collected first after euthanasia using strict aseptic techniques and disposable instruments for each tissue or organ.

Brain and spinal cord will be collected first, then the liver followed by major organs (kidney, lung, heart, spleen), then remaining tissues.

Effort will be made to collect samples of each tissue that weigh between 100-200mg, where available, with exception of the spleen (50-100mg). Samples may be taken by trimming a representative section of the tissue with a clean scalpel, scissors, or by using a clean biopsy punch.

Care will be taken to ensure that cross-contamination between tissues does not occur. Gloves will be changed between collection and dissection of each tissue for analysis. In addition, non-disposable instruments will be wiped down with a 10% bleach solution, rinsed with water, followed by a wipe down of 100% ethanol between each of the specified organs. Tissues for analysis will be collected prior to sample collection for any other parameters.

Following sample collection of all tissues designated for biodistribution analysis in Attachment A, remaining tissues will be stored frozen (-60 to -90°C) for possible future analysis.

15. HISTOLOGY AND MICROSCOPIC EVALUATION

15.1. Histology

Histology will be conducted per Testing Facility SOPs.

15.2. Microscopic Evaluation

Tissues as detailed in the Terminal Procedures and Tissue Weighing, Collection, Processing and Evaluation tables will be evaluated histopathologically by a board-certified veterinary pathologist.

Special stains may be used at the discretion of the pathologist to further characterize lesions and changes identified during routine evaluation of individual animals. Any special stains will be documented in the individual animal data. Any additional stains or evaluations, if deemed necessary by the pathologist, may be added by protocol amendment following discussion with the Study Director and in consultation the Sponsor. Efforts will be made to evaluate all protocol-required tissues microscopically; however, it is not always feasible for every protocol-required tissue to be present on every slide. Protocol-required tissues that are not examined will be documented in the histopathology data and the impact of these missing tissues on the study will be documented in the pathology report.

Images may be generated for illustration of or consultation on histological observations. These images will not be used for data generation or interpretation and will not be archived or included in the Final Report.

In the unlikely event that a Testing Facility pathologist cannot be scheduled for the pathology evaluation due to logistics/timing, the pathology evaluation may be outsourced (by protocol amendment) to an external ACVP-certified veterinary pathologist.

Appendix 1

15.3. Biodistribution/Gene Expression Analysis

The tissue samples will be analyzed for vector DNA biodistribution and gene expression according to the Sponsor's requirements. All analytical work will be conducted by the Sponsor, using an analytical method developed and qualified by that laboratory that conforms to FDA guidelines but not in adherence with GLP.

The work performed in conjunction with this study will not be conducted in compliance with GLPs and will not subject to review by the Quality Assurance Unit (QAU) of that laboratory. A Final Report will be prepared and submitted to Testing Facility for inclusion as an appendix in the main study Final Report.

15.4. Splenocyte Collection and Analysis

Samples from the spleen will be collected from all animals at necropsy.

Remaining spleen after removal of a section for biodistribution and microscopic examination, as applicable, will be processed to splenocytes. Dissected spleens will be placed into prechilled tubes containing RPMI media. Samples will be stored refrigerated at 2-8ºC or on wet ice before processing.

Samples will be processed according to standard Testing Facility method, except that all washes will be performed with RPMI Media and samples will be frozen as follows:

Prepare a sufficient number of 2 mL cryovials to hold the cells at 2e7 cells/mL. Open the vials and add 100 mcL of Hybridoma-Grade DMSO (Freezing media is 90% heat-inactivated FBS and 10 % DMSO). Add sufficient heat-inactivated FBS to the cell pellet to put the cells at 2e7/mL when added to the DMSO. Gently resuspend the pellet via pipetting and then add to the tubes containing DMSO. The act of addition and higher density of FBS will mix the FBS and DMSO yielding an evenly distributed freezing media. 1-2 gentle inversions can be used to assure even mixing. If adequate splenocytes are available, 3 aliquots of cells at 2e7/mL will be prepared for each animal. Splenocytes will be stored in frozen liquid nitrogen until shipped to the Sponsor on dry ice. See Attachment B for shipping details.

The splenocytes will be analyzed for T-cell responses against AAV9 and SURF1. All analytical work will be conducted by the Sponsor, using an analytical method developed and qualified by that laboratory.

The work performed in conjunction with this study will not be conducted in compliance with GLPs and will not subject to review by the Quality Assurance Unit (QAU) of that laboratory. A Final Report will be prepared and submitted to Testing Facility for inclusion as an appendix in the main study Final Report.

16. STATISTICAL ANALYSIS

The following presents a proposed statistical analysis plan. Statistical plans are data dependent, and this analysis plan may require modification if standard data assumptions are not met. Other

conceptually equivalent statistical testing routines may also be employed at the discretion of the statistician. The actual analysis plan will be documented in the Final Report.

The raw data will be tabulated within each time interval, and the appropriate summary statistics will be calculated for each endpoint, sex, and group. For each endpoint, treatment groups will be compared to the control group using the analysis outlined below. Data for some endpoints, as indicated, will be transformed by either a log or rank transformation prior to conducting the specified analysis.

16.1. Statistical Comparisons

Control Group	Comparison Group(s)
1	2, 3, 4

16.2. Group Pair-wise Comparison (General ANOVA)

Endpoints:

- Body Weight and Body Weight Change
- Food Consumption
- Hematology
- Coagulation
- Clinical Chemistry
- Organ Weights
  - Absolute Weights
  - Relative to Body and Brain Weights

Description:

The experimental unit for the analysis of food consumption will be cage, while for all other endpoints the experimental unit will be the individual animal. Food consumption will be calculated as described in Testing Facility SOP.

If the control group has a sample size less than 3, no inferential statistics will be calculated. If a particular endpoint and/or parameter within a given collection interval have the same value across all experimental units, no inferential statistics will be calculated.

Otherwise, for endpoints and/or parameters where all groups with sample sizes of 3 or greater are included, the system will test the normality of the residuals and homogeneity of variances to see whether the data is approximately normal or whether a log transformation or rank transformation should be used. Levene's test will be used to assess homogeneity of group variances and Shapiro-Wilk's test will be used to test the normality of the residuals.[1,2]

On the raw data, if Levene's test is not significant ($p \geq 0.01$) and Shapiro-Wilk's test is not significant ($p \geq 0.01$), then a normal distribution will be

used. If either the Levene's test is significant ($p<0.01$) or Shapiro-Wilk's test is significant ($p<0.01$), normality and homogeneity of variances will be tested with a log transformation used on the data.

On the log transformed data, if Levene's test is not significant ($p\geq0.01$) and Shapiro-Wilk's test is not significant ($p\geq0.01$), then a log normal distribution will be used. If either the Levene's test is significant ($p<0.01$) or Shapiro-Wilk's test is significant ($p<0.01$), then a rank transformation will be used on the data.

Raw or Log Transformed Data:

A one-way analysis of variance will be used to test each endpoint for the effects of treatment.[3]

If the treatment effect is significant ($p<0.05$), linear contrasts will be constructed for a Dunnett's pair-wise comparison of treatment groups as described above.

Rank Transformed Data:

A Kruskal-Wallis test will be used to test each endpoint for the effects of treatment.

If the treatment effect is significant ($p<0.05$), a non-parametric Dunn's pair-wise comparison test of each treatment group with the control group.

Results of all pair-wise comparisons will be reported at the 0.05 and 0.01 significance levels. All endpoints will be analyzed using two-tailed tests unless indicated otherwise.

17. COMPUTERIZED SYSTEMS

The following computerized systems may be used in the study. The actual computerized systems will be documented in the study data.

Critical Computerized Systems

Computer System Name	Description
DocuSign®	Collection of Part 11 compliant signature(s).
ExyLIMS	A comprehensive laboratory information management system used to manage data, including but not limited to: instrumentation, test articles, standards, and samples.
Logbook	Electronic notebook and data collection system for veterinary communications, observations, and treatments.
Deviation Information Library (DEVIL)	Deviations
Share Document Management System (SDMS)	Reporting

Computer System Name	Description
Provantis™	Client-server, Oracle-based system used for electronic documentation and data management from compound receipt through reporting.
SAS®	An integrated system of software products that enables a user to perform data entry, retrieval, data management, reporting, graphics, statistical analysis, and applications development.
Siemens Environmental Monitoring and Niagara Framework® Software System	Environmental monitoring, alarming, and reporting applications.

18. REGULATORY COMPLIANCE

The study will be performed in accordance with the U.S. Department of Health and Human Services, Food and Drug Administration, United States Code of Federal Regulations, Title 21, Part 58: Good Laboratory Practice for Nonclinical Laboratory Studies and as accepted by Regulatory Authorities throughout the European Union (OECD Principles of Good Laboratory Practice), Japan (MHLW), and other countries that are signatories to the OECD Mutual Acceptance of Data Agreement.

Exceptions to GLPs include the following study elements:

- Characterization of the test and control articles will be/were performed by the Sponsor or Sponsor subcontractor according to established SOPs, controls, and approved test methodologies to ensure integrity and validity of the results generated; these analyses will not be/were not conducted in compliance with the GLP or GMP regulations.
- Biodistribution, PBMC, Serum, CSF, and Splenocyte Analysis will not be conducted in compliance with GLP regulations.

19. QUALITY ASSURANCE

19.1. Testing Facility

The Testing Facility Quality Assurance Unit (QAU) will monitor the study to assure the facilities, equipment, personnel, methods, practices, records, and controls are in conformance with Good Laboratory Practice regulations. The QAU will review the protocol, conduct inspections at intervals adequate to assure the integrity of the study, and audit the Final Report to assure that it accurately describes the methods and standard operating procedures and that the reported results accurately reflect the raw data of the study.

19.2. Test Site(s)/Subcontractor(s)

For all study phase(s) inspected by test site/subcontractor QAU(s), copies of each periodic inspection report will be made available to the Study Director, Testing Facility Management, and the Testing Facility QAU.

20. AMENDMENTS AND DEVIATIONS

Changes to the approved protocol shall be made in the form of an amendment, which will be signed and dated by the Study Director. Every reasonable effort will be made to discuss any necessary protocol changes in advance with the Sponsor. The Study Director will notify the Sponsor of deviations that may result in a significant impact on the study as soon as possible.

21. RETENTION AND DISPOSITION OF RECORDS, SAMPLES, AND SPECIMENS

All study-specific raw data, electronic data, documentation, protocol, retained samples and specimens, and Interim (if applicable) and Final Reports will be archived per Testing Facility SOP. All retained materials will be archived at Charles River Laboratories-MWN, unless specified by the Sponsor. At least 1 year after issue of the Draft Report, the Sponsor will be contacted.

Samples for clinical pathology evaluations are discarded per Testing Facility SOP unless otherwise indicated in the table below.

Disposition of residual/retained analytical samples will be as described in the table below. See Attachment B for shipping details.

Disposition of Residual/Retained Samples

Sample Type	Disposition	Schedule
Residual Dosing Formulation	Returned to sponsor	Per disposition indicated in the Preparation and Disposition of Dosing Formulations section.

Records to be maintained will include, but will not be limited to, documentation and data for the following:

- Protocol, protocol amendments, and deviations
- Study schedule
- Study-related correspondence
- Test system receipt, health, and husbandry
- Test and control article receipt, identification, preparation, and analysis
- In-life measurements and observations
- Clinical pathology sample collection and evaluation
- Bioanalytical sample collection and evaluation
- Gross and microscopic observations and related data
- Organ weight measurements
- Statistical analysis results

22. REPORTING

An audited comprehensive Draft Report will be prepared following completion of the study and will be finalized following consultation with the Sponsor. The report will include all information necessary to provide a complete and accurate description of the experimental methods and results and any circumstances that may have affected the quality or integrity of the study.

The Sponsor will receive an electronic version of the Draft and Final Report provided in Adobe Acrobat PDF format (hyperlinked and searchable at Final). The PDF document will be created

from native electronic files to the extent possible, including text and tables generated by the Testing Facility. Report components not available in native electronic files and/or original signature pages will be scanned and converted to PDF image files for incorporation.

A signed version of the pathology Individual Scientist report will be issued with or subsequent to the issuance of the audited Draft Report. Until the signed pathology report is issued, the unsigned Pathology Report will be marked ‘Not Intended for Regulatory Submission’ and will be listed on the Statement of Compliance in the audited Draft Report relating to the unsigned nature of this report. After the Pathology Report is signed, any subsequent revisions will be made in the form of an amended report, which will delineate the changes, as well as the reasons for those changes.

Reports should be finalized within 6 months of issue of the Draft Report. If the Sponsor has not provided comments to the report within 6 months of draft issue, the report will be finalized by the Testing Facility unless other arrangements are made by the Sponsor.

23. JUSTIFICATIONS AND GUIDELINES

23.1. Justification of Test System and Number of Animals

The current state of scientific knowledge and the applicable guidelines cited in this protocol do not provide acceptable alternatives, in vitro or otherwise, to the use of live animals to accomplish the purpose of this study. “The development of knowledge necessary for the improvement of the health and well-being of humans as well as other animals requires in vivo experimentation with a wide variety of animal species.”[4] “Whole animals are essential in research and testing because they best reflect the dynamic interactions between the various cells, tissues, and organs comprising the human body.”[5]

The rat is the usual rodent model used for evaluating the toxicity of various classes of chemicals and for which there is a large historical database.[6]

The total number of animals to be used in this study is considered to be the minimum required to properly characterize the effects of the test article and has been designed such that it does not require an unnecessary number of animals to accomplish its objectives.

23.2. Justification of Route and Dose Levels

Intrathecal injection is the intended route of administration of this test article in humans.

The dose levels were selected based on information provided by the Sponsor that the target dose conferred efficacy to SURF1 knock-out mice, was found to be well-tolerated in mice up to 10 months post-injection, models a relevant dose to human patients, and in an attempt to produce graded responses to the test article. The high-dose level (effectively above a maximum feasible human dose) may produce some toxic effects, but not excessive lethality that would prevent meaningful evaluation. The mid-dose level (target dose) and low-dose level are not expected to produce observable indications of toxicity.

23.3. Guidelines for Study

The design of this study was based on the study objective(s), the overall product development strategy for the test article, and the following study design guidelines:

- FDA Guidance for Industry. *Preclinical Assessment of Investigational Cellular and Gene Therapy Products.*
- FDA Draft Guidance for Industry. *Human Gene Therapy for Rare Diseases*.
- ICH Harmonised Tripartite Guideline S6 (R1). *Preclinical Safety Evaluation of Biotechnology-Derived Pharmaceuticals.*

24. ANIMAL WELFARE

This study will comply with all applicable sections of the Final Rules of the Animal Welfare Act regulations (Code of Federal Regulations, Title 9), the *Public Health Service Policy on Humane Care and Use of Laboratory Animals* from the Office of Laboratory Animal Welfare, and the *Guide for the Care and Use of Laboratory Animals* from the National Research Council.[7,8] The protocol and any amendments or procedures involving the care or use of animals in this study will be reviewed and approved by the Testing Facility Institutional Animal Care and Use Committee before the initiation of such procedures.

If an animal is determined to be in overt pain/distress, or appears moribund and is beyond the point where recovery appears reasonable, the animal will be euthanized for humane reasons in accordance with the *American Veterinary Medical Association (AVMA) Guidelines on Euthanasia* and with the procedures outlined in the protocol.[9]

By approving this protocol, the Sponsor affirms that there are no acceptable non-animal alternatives for this study, that this study is required by a relevant government regulatory agency(ies) and that it does not unnecessarily duplicate any previous experiments.

24.1. Institutional Animal Care and Use Committee Approval

The protocol and any amendment(s) or procedures involving the care and use of animals in this study will be reviewed and approved by CR-MWN Institutional Animal Care and Use Committee (IACUC) before conduct. During the study, the care and use of animals will be conducted with guidance from the guidelines of the USA National Research Council.

25. REFERENCES

1. Milliken GA, Johnson DE. *Analysis of messy data*. London: Chapman and Hall; 1992.
2. Royston JP. *Approximating the Shapiro-Wilk W Test for Nonnormality*. Statistics and Computing 2. London: Chapman and Hall; 1992:117–119.
3. Zar JH. *Biostatistical Analysis*. 4th ed. New Jersey: Prentice Hall; 1999.

Appendix 1

4. *Principles for the Utilization and Care of Vertebrate Animals Used in Testing, Research, and Training*, Federal Register, 1985 May 20; 50(97).
5. *Position Statement on the Use of Animals in Research*, 1993 Feb 26; NIH Guide 22(8).
6. *Guidance for Industry, Investigators, and Reviewers: Exploratory IND Studies.* US FDA Center for Drug Evaluation and Research (CDER), 2006 Jan.
7. Office of Laboratory Animal Welfare. *Public Health Services Policy on Humane Care and Use of Laboratory Animals*. Bethesda, MD: National Institutes of Health. Current edition.
8. National Research Council. *Guide for the Care and Use of Laboratory Animals*. Washington, DC: National Academy Press. Current edition.
9. American Veterinary Medical Association. *AVMA Guidelines on Euthanasia*. Current edition.

Appendix 1

TESTING FACILITY APPROVAL

The signature below indicates that Testing Facility Management approves the Study Director identified in this protocol and management's responsibility to the study as defined by the relevant GLP regulations.

DocuSigned by:

Signer Name: Chris Papagiannis
Signing Reason: I approve this document
Signing Time: 14-Aug-2020 | 12:02:42 EDT
F12230D192174A8080CB62F088113272

Testing Facility Management/Date

The signature below indicates that the Study Director approves the study protocol.

DocuSigned by:

Signer Name: Barak Gunter
Signing Reason: I approve this document
Signing Time: 14-Aug-2020 | 12:30:54 EDT
328F1F1C1E5B44A8AF90ED0F055A9167

Study Director/Date

Appendix 1

SPONSOR APPROVAL

The protocol was approved by the Sponsor by e-mail on the date designated below. The correspondence giving approval will be archived, as appropriate with other Sponsor communications.

14 Aug 2020
Date of Sponsor Approval

Appendix 1

ATTACHMENT A

Tissue Weighing, Collection, Processing and Evaluation Table

Organ	Weigh	Macroscopic Evaluation and Collection	Histology Processing and Microscopic Evaluation	Biodistribution Analysis	Remaining Frozen Tissue to Archive
Animal ID	-	X	-	-	-
Artery, aorta	-	X	-	-	-
Body cavity, nasal	-	X	-	-	-
Bone marrow, sternum	-	X	-	-	-
Bone marrow smear	-	X[a]	-	-	-
Bone, femur	-	X (1)	-	-	X(1)
Bone, sternum	-	X	-	-	-
Brain	X	X	X (Left)	X (Right) (forebrain, midbrain, and hindbrain)	X (Right) (Forebrain, midbrain, and hindbrain)
Epididymis	X (2)	X (2)[b]	-	-	-
Esophagus	-	X	-	-	-
Eye	-	X (2)[b]	X (1) (Left)	-	X (1) (Right)
Ganglion, dorsal root, cervical	-	X (2)	X (2)	X	X
Ganglion, dorsal root, thoracic	-	X (2)	X (2)	-	X
Ganglion, dorsal root, lumbar	-	X (2)	X (2)	X	X
Gland, adrenal	X (2)	X (2)	-	-	-
Gland, clitoral	-	X (2)	-	-	-
Gland, lacrimal	-	X (2) (extra-orbital)	-	-	-
Gland, Harderian	-	X (2)	-	-	-
Gland, mammary	-	X	-	-	-
Gland, parathyroid	-[d]	X (2)	-	-	-
Gland, pituitary	X	X	-	-	-
Gland, preputial	-	X (2)	-	-	-
Gland, prostate	X	X	-	-	-
Gland, salivary, submandibular	X	X (2)	-	-	X (2)
Gland, salivary, sublingual	-	X (2)	-	-	-
Gland salivary, parotid	-	X (2)	-	-	-
Gland, seminal vesicle	X	X (2)	-	-	-
Gland, thyroid	X (2)	X (2)	-	-	X (2)
Gland, Zymbal's	-	X (2)	-	-	-
Gut-associated lymphoid tissue[c]	-	X	-	-	-
Heart	X	X	X	X	X
Joint, femorotibial	-	X (1)	-	-	-
Kidney	X (2)	X (2)	X (Left)	X (Right)	X (Right)

Appendix 1

Organ	Weigh	Macroscopic Evaluation and Collection	Histology Processing and Microscopic Evaluation	Biodistribution Analysis	Remaining Frozen Tissue to Archive
Large intestine, cecum	-	X	-		
Large intestine, colon	-	X	-	-	X
Large intestine, rectum	-	X	-	-	-
Larynx	-	X	-	-	-
Liver	X	X	X	X	X
Lung	X	X	X	-	X
Lymph node(s) draining administration site(s): Iliac	-	X (2)	X (1)	-	X (1)
Lymph node, mandibular	X	X (2)	X (1)	-	X (1)
Lymph node, mesenteric	X	X	X	-	X
Muscle, skeletal (bicep femoris and gastrocnemius)	-	X (2)	X (1)	X (1)	X (1)
Nerve, optic	-	X (2)[b]	X (Left)	-	X (Right)
Nerve, sciatic	-	X (2)	X (1)	X (1)	X (1)
Nerve, tibial	-	X (2)	X (1)	-	X (1)
Ovary	X (2)	X (2)	X (Left)	X (Right)	X (Right)
Oviduct	X (2)	X (2)	-	-	X (2)
Pancreas	-	X	X	-	X
Skin	-	X	-	-	X
Small intestine, duodenum	-	X	-	-	X
Small intestine, ileum	-	X	-	-	X
Small intestine, jejunum	-	X	-	-	X
Spinal cord, cervical	-	X	X	X	X
Spinal cord, thoracic	-	X	X	-	X
Spinal cord. lumbar	-	X	X	X	X
Spleen	X	X	X	X	X
Stomach	-	X	-	-	X
Testis	X (2)	X (2)[b]	X (Left)	X (Right)	X (Right)
Thymus	X	X	X	X	X
Tongue	-	X	-	-	-
Trachea	-	X	-	-	-
Ureter	-	X (2)	-	-	-
Urinary bladder	-	X	-	-	X
Uterus/Cervix	X	X	-	-	X
Vagina	-	X	-	-	-

Appendix 1

Organ	Weigh	Macroscopic Evaluation and Collection	Histology Processing and Microscopic Evaluation	Biodistribution Analysis	Remaining Frozen Tissue to Archive

X = Procedure to be conducted. - = Not applicable. (1) = one side. (2) = both sides.
Macroscopic abnormalities in the organs listed and in other organs will be sampled at necropsy, processed for histology and examined microscopically.

[a] Two bone marrow smears will be collected from the femur at scheduled and unscheduled necropsies (for possible examination). Smears will not be collected from animals that are found dead or from animals that were euthanized moribund and then stored in the refrigerator prior to necropsy. Bone marrow smears are allowed to air dry and are not fixed in formalin.

[b] Eyes and optic nerves are preserved in Davidson’s fixative. Testes and epididymides are preserved in modified Davidson’s fixative.

[c] From small intestine: Peyer’s patch or solitary lymphoid follicle.

[d] Weigh with gland, thyroid.

Appendix 1

ATTACHMENT B

Shipment of Samples

Samples will be shipped on Monday through Wednesday for next day delivery for domestic shipments and expedited delivery for International shipments using the Conditions for Shipment(s) listed below.

Sample Matrix	Sample Type	Aliquot of Sample	Proposed Shipment	Conditions for Shipment	Recipient/Address
NA	Remaining Unused Bulk Test Article	NA	Following Completion of in-life procedures	on dry ice	Attn: Steven Gray, PhD<br>University of Texas Southwestern Medical Center<br>6000 Harry Hines Blvd<br>University of Texas Southwestern Medical Center<br>Dallas, TX 75390<br>Tel: 214-648-0670<br>E-mail:<br>steven.gray@UTsouthwestern.edu
Blood, CSF, tissue samples	Bioanalytical Samples	All	Following collection of terminal samples		
NA	Splenocyte Samples	All	Following collection of terminal samples		

Appendix 2

UT Southwestern Form/Checklist

Certificate of Testing

**Batch ID:**	TX-07-20-001
**Product Name:**	AAV9/Surf 1
**Manufacturing Date:**	14DEC2020
**Expiration/Retest Date:**	N/A
**Storage Conditions:**	≤-60˚C, Upon thawing, 4˚C

Testing Site	Test	Result
UTSW-TGTC	pH	~7
UTSW-TGTC	Endotoxin	0.118 EU/mL
UTSW-TGTC	Titer (qPCR linearized plasmid)	7.45E13 vg/mL
UTSW-TGTC	Titer (qPCR ITR)	Not available
UTSW-TGTC	Titer (ddPCR)	3.36E13 vg/mL
UTSW-TGTC	Capsid Titer (ELISA)	7.83E14 vp/mL
UTSW-TGTC	% Empty (AUC)	64%
UTSW-TGTC	Purity (SDS PAGE w/Silver Stain)	VP1/2/3 Bands Observed. No other major bands.
UTSW-TGTC	Potency (Copies Transgene/Copies GAPDH)	Not tested
UTSW-TGTC	Residual HCP	Not detected
UTSW-TGTC	Residual HCD	1.20E4 ng/mL
UTSW-TGTC	Residual Plasmid (KAN Copies/mL)	1.94E13 KAN Copies/mL)
ARL	Sterility	No growth detected.
CRL	Mycoplasma	None detected.

Approved By E. Laverley 04 AUG 2021
QA Manager, UTSW, TGTC

Appendix 2

UT Southwestern Form/Checklist

Certificate of Testing

**Batch ID:**	TOX-07-20-196
**Product Name:**	AAV9/SURF1
**Manufacturing Date:**	21AUG2020
**Expiration/Retest Date:**	N/A
**Storage Conditions:**	≤-60˚C, Upon thawing, 4˚C

Testing Site	Test	Result
UTSW-TGTC	pH	~7
UTSW-TGTC	Endotoxin (EU/mL)	1.02 EU/mL
UTSW-TGTC	Titer (qPCR linearized plasmid)	5.07E13 vg/mL
UTSW-TGTC	Titer (qPCR ITR)	3.18E13 vg/mL
UTSW-TGTC	Titer (ddPCR)	4.15E13 vg/mL
UTSW-TGTC	Capsid Titer (ELISA)	4.67E14 vp/mL
UTSW-TGTC	% Empty (AUC)	46%
UTSW-TGTC	Purity (SDS PAGE w/Silver Stain)	VP1/2/3 Bands Observed. No other major bands.
UTSW-TGTC	Potency	5.64E4(Copies Transgene/Copies GAPDH)
UTSW-TGTC	Residual HCP	Not detected
UTSW-TGTC	Residual HCD	3.10E4 ng/mL
UTSW-TGTC	Residual Plasmid	1.58E13 KAN Copies/mL
ARL	Sterility	No growth detected.
CRL	Mycoplasma	None detected

Approved By E.Laverley 04 AUG 2021

QA Manager, UTSW, TGTC

On occasion, clinical findings may have been observed more than once during the interval and were documented accordingly in the raw data. The individual clinical observations table of this appendix reports the findings observed, not the number of times observed within an interval.

Abbreviations

SIRT – Signs of ill health related to treatment

Appendix 3

Individual Mortality

2954-001

Sex: Male Day(s): 1 to 91 Relative to Start Date

Group 1	Day of Death	Removal Date	Path Removal Reason
1006	87	20-Nov-2020	TERM
1007	87	20-Nov-2020	TERM
1008	87	20-Nov-2020	TERM
1009	91	24-Nov-2020	TERM
1010	91	24-Nov-2020	TERM
1011	8	08-Oct-2020	TERM
1012	8	08-Oct-2020	TERM
1013	8	08-Oct-2020	TERM
1014	8	08-Oct-2020	TERM
1015	8	08-Oct-2020	TERM
1021	29	24-May-2021	TERM
1022	29	24-May-2021	TERM
1023	29	25-May-2021	TERM
1024	29	25-May-2021	TERM
1025	29	26-May-2021	TERM

TE/TERM = Terminal Euthanasia FD = Found Dead UE/UNSC = Unscheduled Euthanasia
IE/INTM = Interim Euthanasia REC = Recovery Euthanasia

Appendix 3

Individual Mortality

2954-001

Sex: Male Day(s): 1 to 91 Relative to Start Date

Group 2	Day of Death	Removal Date	Path Removal Reason
2006	87	20-Nov-2020	TERM
2007	87	20-Nov-2020	TERM
2008	87	20-Nov-2020	TERM
2009	91	24-Nov-2020	TERM
2010	91	24-Nov-2020	TERM
2011	8	08-Oct-2020	TERM
2012	8	08-Oct-2020	TERM
2013	8	08-Oct-2020	TERM
2014	8	08-Oct-2020	TERM
2015	8	08-Oct-2020	TERM
2021	29	24-May-2021	TERM
2022	29	24-May-2021	TERM
2023	29	25-May-2021	TERM
2024	29	25-May-2021	TERM
2025	29	26-May-2021	TERM

TE/TERM = Terminal Euthanasia FD = Found Dead UE/UNSC = Unscheduled Euthanasia
IE/INTM = Interim Euthanasia REC = Recovery Euthanasia

Appendix 3

Individual Mortality

2954-001

Sex: Male Day(s): 1 to 91 Relative to Start Date

Group 3	Day of Death	Removal Date	Path Removal Reason
3006	87	20-Nov-2020	TERM
3007	87	20-Nov-2020	TERM
3008	16	10-Sep-2020	UNSC
3009	91	24-Nov-2020	TERM
3010	91	24-Nov-2020	TERM
3011	8	08-Oct-2020	TERM
3012	8	08-Oct-2020	TERM
3013	8	08-Oct-2020	TERM
3014	8	08-Oct-2020	TERM
3015	8	08-Oct-2020	TERM
4013	8	08-Oct-2020	TERM
3021	29	24-May-2021	TERM
3022	29	24-May-2021	TERM
3023	29	25-May-2021	TERM
3024	29	25-May-2021	TERM
3025	29	26-May-2021	TERM

TE/TERM = Terminal Euthanasia FD = Found Dead UE/UNSC = Unscheduled Euthanasia
IE/INTM = Interim Euthanasia REC = Recovery Euthanasia

Appendix 3

Individual Mortality

2954-001

Sex: Male Day(s): 1 to 91 Relative to Start Date

Group 4	Day of Death	Removal Date	Path Removal Reason
4006	87	20-Nov-2020	TERM
4007	87	20-Nov-2020	TERM
4008	87	20-Nov-2020	TERM
4009	91	24-Nov-2020	TERM
4010	91	24-Nov-2020	TERM
4011	8	08-Oct-2020	TERM
4012	8	08-Oct-2020	TERM
4113	8	05-May-2021	TERM
4021	8	05-May-2021	TERM
4022	8	05-May-2021	TERM
4023	29	24-May-2021	TERM
4024	29	24-May-2021	TERM
4025	29	26-May-2021	TERM
4026	29	26-May-2021	TERM
4027	29	26-May-2021	TERM

TE/TERM = Terminal Euthanasia FD = Found Dead UE/UNSC = Unscheduled Euthanasia
IE/INTM = Interim Euthanasia REC = Recovery Euthanasia

Appendix 3

Individual Mortality

2954-001

Sex: Female Day(s): 1 to 91 Relative to Start Date

Group 1	Day of Death	Removal Date	Path Removal Reason
1506	90	24-Nov-2020	TERM
1507	90	24-Nov-2020	TERM
1508	90	24-Nov-2020	TERM
1509	91	25-Nov-2020	TERM
1510	91	25-Nov-2020	TERM
1511	8	12-Oct-2020	TERM
1512	8	12-Oct-2020	TERM
1513	8	12-Oct-2020	TERM
1514	8	12-Oct-2020	TERM
1515	8	12-Oct-2020	TERM
1521	29	24-May-2021	TERM
1522	29	24-May-2021	TERM
1523	29	25-May-2021	TERM
1524	29	25-May-2021	TERM
1525	29	26-May-2021	TERM

TE/TERM = Terminal Euthanasia FD = Found Dead UE/UNSC = Unscheduled Euthanasia
IE/INTM = Interim Euthanasia REC = Recovery Euthanasia

Appendix 3

Individual Mortality

2954-001

Sex: Female Day(s): 1 to 91 Relative to Start Date

Group 2	Day of Death	Removal Date	Path Removal Reason
2506	90	24-Nov-2020	TERM
2507	90	24-Nov-2020	TERM
2508	90	24-Nov-2020	TERM
2509	91	25-Nov-2020	TERM
2510	91	25-Nov-2020	TERM
2511	8	12-Oct-2020	TERM
2512	8	12-Oct-2020	TERM
2513	8	12-Oct-2020	TERM
2514	8	12-Oct-2020	TERM
2515	8	12-Oct-2020	TERM
2521	29	24-May-2021	TERM
2522	29	24-May-2021	TERM
2523	29	25-May-2021	TERM
2524	29	25-May-2021	TERM
2525	29	26-May-2021	TERM

TE/TERM = Terminal Euthanasia FD = Found Dead UE/UNSC = Unscheduled Euthanasia
IE/INTM = Interim Euthanasia REC = Recovery Euthanasia

Appendix 3

Individual Mortality

2954-001

Sex: Female Day(s): 1 to 91 Relative to Start Date

Group 3	Day of Death	Removal Date	Path Removal Reason
3506	91	25-Nov-2020	TERM
3507	91	25-Nov-2020	TERM
3508	91	25-Nov-2020	TERM
3509	91	25-Nov-2020	TERM
3510	91	25-Nov-2020	TERM
3511	8	12-Oct-2020	TERM
3512	8	12-Oct-2020	TERM
3513	8	12-Oct-2020	TERM
3514	8	12-Oct-2020	TERM
3515	8	12-Oct-2020	TERM
3521	29	24-May-2021	TERM
3522	29	24-May-2021	TERM
3523	29	25-May-2021	TERM
3524	29	25-May-2021	TERM
3525	29	26-May-2021	TERM

TE/TERM = Terminal Euthanasia FD = Found Dead UE/UNSC = Unscheduled Euthanasia
IE/INTM = Interim Euthanasia REC = Recovery Euthanasia

Appendix 3

Individual Mortality

2954-001

Sex: Female Day(s): 1 to 91 Relative to Start Date

Group 4	Day of Death	Removal Date	Path Removal Reason
4506	91	25-Nov-2020	TERM
4507	91	25-Nov-2020	TERM
4508	91	25-Nov-2020	TERM
4509	91	25-Nov-2020	TERM
4510	91	25-Nov-2020	TERM
4511	8	12-Oct-2020	TERM
4512	8	12-Oct-2020	TERM
4513	8	12-Oct-2020	TERM
4514	8	12-Oct-2020	TERM
4515	8	12-Oct-2020	TERM
4521	29	25-May-2021	TERM
4522	29	25-May-2021	TERM
4523	29	26-May-2021	TERM
4524	29	26-May-2021	TERM
4525	29	26-May-2021	TERM

TE/TERM = Terminal Euthanasia FD = Found Dead UE/UNSC = Unscheduled Euthanasia
IE/INTM = Interim Euthanasia REC = Recovery Euthanasia

Individual Clinical Observations

2954-001

Group 1<br>Sex: Male	Observation Type: Toxicology Observations	Day(s) Relative to Start Date						
		-1	7	14	21	28	35	42
1009	Skin, Scab, Dorsal Thoracic	.	.	X	.	.	.	.
1010	Fur, Thin Cover, Dorsal Cervical	.	.	.	.	.	.	.

X=Present

Individual Clinical Observations

2954-001

Group 1<br>Sex: Male	Observation Type: Toxicology Observations	Day(s) Relative to Start Date						
		49	56	63	70	77	84	91
1009	Skin, Scab, Dorsal Thoracic	.	.	.	.	.	.	.
1010	Fur, Thin Cover, Dorsal Cervical	.	.	.	.	.	.	X

X=Present

Individual Clinical Observations

2954-001

Group 2<br>Sex: Male	Observation Type: Toxicology Observations	Day(s) Relative to Start Date						
		-1	7	14	21	28	35	42
2006	Fur, Thin Cover, Face	.	.	.	.	.	X	X
	Skin, Scab, Face	.	.	.	.	X	X	X
2007	Fur, Thin Cover, Face	.	.	.	.	.	X	X
	Skin, Scab, Face	.	.	.	.	.	X	X
	Skin, Scab, Muzzle	.	.	.	.	.	.	.
	Eyeball, Abnormal Color, Right, Pale	.	.	.	.	.	.	.
2008	Fur, Thin Cover, Forelimb, Left	.	.	.	.	.	.	.
	Fur, Thin Cover, Forelimb, Right	.	.	.	.	.	.	.
2010	Fur, Thin Cover, Face	.	.	.	.	X	X	X
2021	Fur, Thin Cover, Face	.	.	.	.	X	.	.
2023	Fur, Thin Cover, Face	.	.	.	.	X	.	.
2025	Fur, Thin Cover, Face	.	.	X	X	X	.	.

X=Present

Individual Clinical Observations

2954-001

Group 2 Sex: Male	Observation Type: Toxicology Observations	Day(s) Relative to Start Date						
		49	56	63	70	77	84	91
2006	Fur, Thin Cover, Face	X	X	X	X	X	X	.
	Skin, Scab, Face	X	X	X	.	.	.	.
2007	Fur, Thin Cover, Face	X	X	X	X	X	X	.
	Skin, Scab, Face	.	.	.	X	.	.	.
	Skin, Scab, Muzzle	.	.	.	.	.	X	.
	Eyeball, Abnormal Color, Right, Pale	.	.	.	X	X	X	.
2008	Fur, Thin Cover, Forelimb, Left	.	.	.	.	X	X	.
	Fur, Thin Cover, Forelimb, Right	.	.	.	.	.	X	.
2010	Fur, Thin Cover, Face	.	.	.	.	.	.	.
2021	Fur, Thin Cover, Face	.	.	.	.	.	.	.
2023	Fur, Thin Cover, Face	.	.	.	.	.	.	.
2025	Fur, Thin Cover, Face	.	.	.	.	.	.	.

X=Present

Individual Clinical Observations

2954-001

Group 3<br>Sex: Male	Observation Type: Toxicology Observations	Day(s) Relative to Start Date						
		-1	7	14	21	28	35	42
3006	Fur, Staining, Dorsal Cervical, Red	.	.	.	.	.	.	.
	Fur, Thin Cover, Face	.	.	.	.	.	.	.
3008	Splayed Limb, Hindlimb, Left	.	.	X	.	.	.	.
	Splayed Limb, Hindlimb, Right	.	.	X	.	.	.	.
	Abnormal Gait	.	.	X	.	.	.	.
3009	Fur, Staining, Dorsal Cervical, Red	.	.	.	.	.	.	.
3010	Fur, Thin Cover, Face	.	.	.	.	.	.	.
	Skin, Scab, Face	.	.	.	.	.	.	.
3022	Discharge, Color, Penis, Clear	.	.	X	.	.	.	.
3023	Fur, Thin Cover, Face	.	.	.	.	X	.	.
	Skin, Scab, Face	.	.	.	.	X	.	.
3025	Fur, Thin Cover, Face	.	.	.	X	X	.	.

X=Present

Individual Clinical Observations

2954-001

Group 3<br>Sex: Male	Observation Type: Toxicology Observations	Day(s) Relative to Start Date						
		49	56	63	70	77	84	91
3006	Fur, Staining, Dorsal Cervical, Red	.	.	.	X	X	X	.
	Fur, Thin Cover, Face	.	.	.	X	X	X	.
3008	Splayed Limb, Hindlimb, Left	.	.	.	.	.	.	.
	Splayed Limb, Hindlimb, Right	.	.	.	.	.	.	.
	Abnormal Gait	.	.	.	.	.	.	.
3009	Fur, Staining, Dorsal Cervical, Red	.	.	.	X	X	X	X
3010	Fur, Thin Cover, Face	.	.	.	.	.	.	X
	Skin, Scab, Face	.	.	.	.	.	.	X
3022	Discharge, Color, Penis, Clear	.	.	.	.	.	.	.
3023	Fur, Thin Cover, Face	.	.	.	.	.	.	.
	Skin, Scab, Face	.	.	.	.	.	.	.
3025	Fur, Thin Cover, Face	.	.	.	.	.	.	.

X=Present

Individual Clinical Observations

2954-001

Group 4<br>Sex: Male	Observation Type: Toxicology Observations	Day(s) Relative to Start Date						
		-1	7	14	21	28	35	42
4006	Fur, Staining, Dorsal Cervical, Red	.	.	.	.	.	.	.
4008	Fur, Staining, Dorsal Cervical, Red	.	.	.	.	X	X	X
4012	Tail, Bent (PT)	X	X	.	.	.	.	.
4023	Low Carriage	.	.	.	.	X	.	.

X=Present

Individual Clinical Observations

2954-001

Group 4 Sex: Male	Observation Type: Toxicology Observations	Day(s) Relative to Start Date						
		49	56	63	70	77	84	91
4006	Fur, Staining, Dorsal Cervical, Red	.	.	.	X	X	X	.
4008	Fur, Staining, Dorsal Cervical, Red	.	.	.	X	X	X	.
4012	Tail, Bent (PT)	.	.	.	.	.	.	.
4023	Low Carriage	.	.	.	.	.	.	.

X=Present

Individual Clinical Observations

2954-001

Group 4<br>Sex: Male	Observation Type: SIRT/Cage Side Observations	Day(s) Relative to Start Date						
		1						
4113	Skin, Laceration, Tail, Moderate	X						

X=Present

Individual Clinical Observations

2954-001

Key Page

Group Information

Short Name	Long Name	Type	Report Headings
1	Group 1	Control	Group 1
2	Group 2	Dose	Group 2
3	Group 3	Dose	Group 3
4	Group 4	Dose	Group 4

Individual Clinical Observations

2954-001

Group 1<br>Sex: Female	Observation Type: Toxicology Observations	Day(s) Relative to Start Date						
		7	14	21	28	49	56	63
1507	Fur, Staining, Cranium, Red	.	.	.	.	.	.	.
	Nail Missing, Forepaw, Left	.	.	.	.	.	.	.
1523	Dehydrated Suspected, Severity Not Recorded	.	.	X	.	.	.	.
	Hunched Posture	.	.	X	.	.	.	.
1524	Dehydrated Suspected, Severity Not Recorded	.	.	X	.	.	.	.
	Hunched Posture	.	.	X	.	.	.	.

X=Present

Individual Clinical Observations

2954-001

Group 1<br>Sex: Female	Observation Type: Toxicology Observations	Day(s) Relative to Start Date						
		70	77	84	91			
1507	Fur, Staining, Cranium, Red	.	.	X	.			
	Nail Missing, Forepaw, Left	.	.	X	.			
1523	Dehydrated Suspected, Severity Not Recorded	.	.	.	.			
	Hunched Posture	.	.	.	.			
1524	Dehydrated Suspected, Severity Not Recorded	.	.	.	.			
	Hunched Posture	.	.	.	.			

X=Present

Individual Clinical Observations

2954-001

Group 2<br>Sex: Female	Observation Type: Toxicology Observations	Day(s) Relative to Start Date						
		7	14	21	28	49	56	63
2523	Dehydrated Suspected, Severity Not Recorded	.	.	X	.	.	.	.
2524	Dehydrated Suspected, Severity Not Recorded	.	.	X	.	.	.	.

X=Present

Individual Clinical Observations

2954-001

Group 2<br>Sex: Female	Observation Type: Toxicology Observations	Day(s) Relative to Start Date						
		70	77	84	91			
2523	Dehydrated Suspected, Severity Not Recorded	.	.	.	.			
2524	Dehydrated Suspected, Severity Not Recorded	.	.	.	.			

Individual Clinical Observations

2954-001

Group 3<br>Sex: Female	Observation Type: Toxicology Observations	Day(s) Relative to Start Date						
		7	14	21	28	49	56	63
3506	Fur, Thin Cover, Dorsal Aspect Generalized	.	.	.	.	.	.	.
3507	Fur, Staining, Cranium, Red	.	.	.	.	.	.	.
	Fur, Staining, Dorsal Cervical, Red	.	.	.	.	.	.	.
3508	Fur, Staining, Face, Red	.	.	.	.	.	.	.
	Fur, Thin Cover, Dorsal Cervical	.	.	.	.	.	.	.
3509	Fur, Thin Cover, Sacral	.	.	.	.	.	.	X
3510	Fur, Staining, Dorsal Cervical, Red	.	.	.	.	.	.	.
	Fur, Staining, Face, Red	.	.	.	.	X	X	X
3523	Swollen, Abdominal, Severity Not Recorded, Firm	.	.	.	X	.	.	.
	Swollen, Axillary, Left, Slight, Firm	X	X	X	.	.	.	.
	Swollen, Axillary, Right, Slight, Firm	X	X	X	.	.	.	.
	Swollen, Inguinal, Left, Slight, Firm	.	.	X	.	.	.	.
	Swollen, Inguinal, Right, Slight, Firm	.	.	X	.	.	.	.
	Swollen, Urogenital, Severity Not Recorded, Firm	.	.	.	X	.	.	.
	Swollen, Ventral Cervical, Slight, Firm	.	.	X	.	.	.	.
3525	Limited Usage, Hindlimb, Left, Moderate	.	X	X	X	.	.	.
	Limited Usage, Hindlimb, Right, Slight	.	X	X	X	.	.	.
	Fur, Staining, Abdominal, Yellow	.	.	X	X	.	.	.
	Fur, Staining, Urogenital, Yellow	.	.	X	X	.	.	.

X=Present

Individual Clinical Observations

2954-001

Group 3<br>Sex: Female	Observation Type: Toxicology Observations	Day(s) Relative to Start Date						
		70	77	84	91			
3506	Fur, Thin Cover, Dorsal Aspect Generalized	.	.	X	X			
3507	Fur, Staining, Cranium, Red	.	.	X	X			
	Fur, Staining, Dorsal Cervical, Red	.	.	X	X			
3508	Fur, Staining, Face, Red	.	.	X	X			
	Fur, Thin Cover, Dorsal Cervical	.	.	.	X			
3509	Fur, Thin Cover, Sacral	X	X	.	.			
3510	Fur, Staining, Dorsal Cervical, Red	.	.	X	X			
	Fur, Staining, Face, Red	X	X	X	X			
3523	Swollen, Abdominal, Severity Not Recorded, Firm	.	.	.	.			
	Swollen, Axillary, Left, Slight, Firm	.	.	.	.			
	Swollen, Axillary, Right, Slight, Firm	.	.	.	.			
	Swollen, Inguinal, Left, Slight, Firm	.	.	.	.			
	Swollen, Inguinal, Right, Slight, Firm	.	.	.	.			
	Swollen, Urogenital, Severity Not Recorded, Firm	.	.	.	.			
	Swollen, Ventral Cervical, Slight, Firm	.	.	.	.			
3525	Limited Usage, Hindlimb, Left, Moderate	.	.	.	.			
	Limited Usage, Hindlimb, Right, Slight	.	.	.	.			
	Fur, Staining, Abdominal, Yellow	.	.	.	.			
	Fur, Staining, Urogenital, Yellow	.	.	.	.			

X=Present

Individual Clinical Observations

2954-001

Group 4<br>Sex: Female	Observation Type: Toxicology Observations	Day(s) Relative to Start Date						
		7	14	21	28	49	56	63
4506	Fur, Staining, Cranium, Red	.	.	.	.	.	.	.
	Fur, Staining, Face, Red	.	.	.	.	.	.	.
4507	Fur, Staining, Dorsal Cervical, Red	.	.	.	.	.	.	.
4509	Fur, Staining, Dorsal Cervical, Red	.	.	.	.	.	.	.
	Fur, Staining, Face, Red	.	.	.	.	.	.	X
	Fur, Thin Cover, Face	.	.	.	.	.	.	.
	Skin, Scab, Face	.	.	.	.	.	.	.
4521	Dehydrated Suspected, Severity Not Recorded	.	.	X	.	.	.	.
4522	Dehydrated Suspected, Severity Not Recorded	.	.	X	.	.	.	.
	Skin, Scab, Dorsal Thoracic	X	X	.	.	.	.	.

X=Present

Individual Clinical Observations

2954-001

Group 4 Sex: Female	Observation Type: Toxicology Observations	Day(s) Relative to Start Date						
		70	77	84	91			
4506	Fur, Staining, Cranium, Red	.	.	X	X			
	Fur, Staining, Face, Red	.	.	X	X			
4507	Fur, Staining, Dorsal Cervical, Red	.	.	.	X			
4509	Fur, Staining, Dorsal Cervical, Red	.	.	.	X			
	Fur, Staining, Face, Red	X	X	X	X			
	Fur, Thin Cover, Face	.	.	X	.			
	Skin, Scab, Face	.	.	X	.			
4521	Dehydrated Suspected, Severity Not Recorded	.	.	.	.			
4522	Dehydrated Suspected, Severity Not Recorded	.	.	.	.			
	Skin, Scab, Dorsal Thoracic	.	.	.	.			

X=Present

Individual Clinical Observations

2954-001

Group 1 Sex: Female	Observation Type: SIRT/Cage Side Observations	Day(s) Relative to Start Date						
		1	20					
1525	Dehydrated Suspected, Severity Not Recorded	.	X					
	Hunched Posture	.	X					

X=Present

Individual Clinical Observations

2954-001

Group 2<br>Sex: Female	Observation Type: SIRT/Cage Side Observations	Day(s) Relative to Start Date						
		1	20					
2525	Dehydrated Suspected, Severity Not Recorded	.	X					

X=Present

Individual Clinical Observations

2954-001

Group 3<br>Sex: Female	Observation Type: SIRT/Cage Side Observations	Day(s) Relative to Start Date						
		1	20					
3521	Breathing, Labored	X	.					
	Hunched Posture	X	.					
	Activity Decreased	X	.					

X=Present

Individual Clinical Observations

2954-001

Key Page

Group Information

Short Name	Long Name	Type	Report Headings
1	Group 1	Control	Group 1
2	Group 2	Dose	Group 2
3	Group 3	Dose	Group 3
4	Group 4	Dose	Group 4

Animal Exam Observation and Treatment Code Page

Body Condition Scoring System (BCS)		
Score[a]	Descriptor	Characteristics
1	Emaciated	Ribs, lumbar, vertebrae, pelvic bones and all bony prominences easily visible. No discernible body fat. Obvious loss of muscle mass.
2	Thin	Ribs visible with no palpable fat. Tops of lumbar vertebrae visible. Pelvic bones becoming prominent. Muscle mass is visible. Obvious waist and abdominal tuck.
3	Normal	Ribs and spinous processes palpable without excess fat covering, but not clearly visible. Waist observed behind ribs when viewed from above. Abdomen tucked up when viewed from side.
4	Overweight	Ribs and spinous processes palpable with difficulty; heavy fat cover. Noticeable fat deposits over lumbar area and base of tail. Waist absent or barely visible. Abdominal tuck may be present.
5	Obese	Ribs not palpable under very heavy fat cover, or palpable only with significant pressure. Heavy fat deposits over lumbar area and base of tail. Waist absent. No abdominal tuck. Obvious abdominal distention may be present.
[a]Intermediate conditions may be indicated using score intervals of 0.5.		

The following abbreviations may be used throughout the table to document general medical information, as well as wound/integument descriptions and food enrichment.

Abbreviations Used Throughout Medical Record Comments				
**Abbreviation**	**Meaning**		**Abbreviation**	**Meaning**
ABD	Abdominal		HCC	Hematology, coagulation, clinical chemistry
~, approx.	Approximately		HFR	Hindfoot right
AD, EAR	Right ear		HFL	Hindfoot left
AF	Animal fasted		HLL	Hindlimb left
AFN	Appetite and feces normal		HLR	Hindlimb right
ANG	Anogenital		HR	Heart rate in beats per minute
AS, EAL	Left ear		HS	Hair sparse
AU	Both ears		IDC	Interdigital cyst
AXL	Axillary left		IGL	Inguinal left
AXR	Axillary right		IGR	Inguinal right
BAR	Bright, alert, and responsive		IM	Intramuscular
BG	Blood glucose		IP	Intraperitoneal
BID	Twice a day		IV	Intravenous
BHV	Behavior		L	Left
BLD	Basal Laboratory Diet		LRS	Lactated Ringer’s Solution
BPM, bpm	Beats per minute		LUM	Lumbar
BF	Baby food		M	Mild
BT	Body temperature		MM	Mucous membranes
BW	Body weight		MOD	moderate
CHX	Chlorhexidine		MOU	Mouth
Clin Med	Clinical Medicine		M-W-F	Monday, Wednesday, Friday
CMP	Clinical Medicine Pharmacy		NA	Not applicable
CRA	Cranial		NAD	Nothing abnormal detected
CRT	Capillary refill time		NBS	Neurobehavioral Sciences
CRV	Cervical		NC	No change in condition
d/c, D/C	Discontinue(d)		Neo/poly/bac	Neomycin, Polymyxin, Bacitracin
DOR	Dorsal		NR	Not required/Not administered
d/x, D/X	Diagnosis		NSAID	Nonsteroidal anti-inflammatory drug
EE	Environmental enrichment		NSF	No significant findings
EENT	Ears, eyes, nose, and throat		Obs	Observations
ENB	Entire body		OD, EYR	Right eye
ENV	Enviro-dri		OS, EYL	Left eye
EXP	Expiration		OU	Both eyes
FBX	Foraging box		PE	Planned event
FE	Food enrichment		PO	Oral
FFL	Forefoot left		PU/PD	Polyuria/Polydipsia
FFR	Forefoot right		PRN, prn	As needed
FLL	Forelimb left		QD	Once daily
FLR	Forelimb right			

Abbreviations Used Throughout Medical Record Comments, Continued				
**Abbreviation**	**Meaning**		**Abbreviation**	**Meaning**
QID	Four times a day		SRG	Surgery
QAR	Quiet, alert, and responsive		SSD	Silver sulfadiazine
R	Right		TAO	Triple antibiotic ointment
RE	Respiratory effort		TA, TM	Test article, Test material
RF	Recently fed		TID	Three times a day
RR, rpm	Respiratory rate in breaths per minute		THR	Thoracic region
RRC	Room recently cleaned		TPR	Temperature, pulse, and respirations
S/R	Suture removal		TX	Treatment
SC/SQ	Subcutaneous		TXØ	Treatment completed/ discontinued
SD	Study Director		TXΔ	Treatment changed
SEV	Severe		UN	Product unavailable at this time
SH	Socially housed/ pair housed		VD	Ventrodorsal
SHL	Shoulder left		VEN	Ventral
SHO	Shoulder right		Vet	Veterinarian
SID	Once a day		VMS	Veterinary Management System
SN	Serial number		W/	With
			WNL	Within normal limits
SOP	Standard operating procedure		X	Times (i.e., 2X weekly)
**WOUND/ INTEGUMENT DESCRIPTIONS**				
B	Black		PLU	Plantar ulcer
CL	Closed		PU	Purulent
D	Dry		RM	Red material
DP	Deep		S	Scabbed
E	Erythemic		SU	Superficial
MO	Moist		SW	Swelling
OP	Open		U	Ulcerated
P	Purple			

Abbreviations Used Throughout Medical Record Comments, Continued				
ENRICHMENT FOOD ABBREVIATIONS				
A	Apples		HNC	Honey Nut Cheerios™
AD	Apple Dried		HY	Honey
AL	Almonds		KWD	Kiwi Dried
APD	Apricots Dried		LC	Lucky Charms™
B	Bananas		MG	Mango
BD	Bananas Dried		MGD	Mango Dried
BF	Baby Food		M	Marshmallow
BFB	Beef Broth		MF	Marshmallow Fluff
BZ	Brazil Nut		MW	Mini Wheats™
C	Carrots		O	Oranges
CA	Cashews		PA	Papaya
CC	Criticare		PAD	Papaya Dried
CH	Cherries		PB	Peanut Butter
CHD	Cherries Dried		PCN	Popcorn
CHB	Chicken Broth		PI	Pineapple
CK	Chick Peas		PID	Pineapple Dried
CL	Celery		PK	Pumpkin
CND	Cranberries Dried		PKS	Pumpkin Seeds
CO	Coconut		PN	Peanuts
COD	Coconut Dried		POP	Lollipop
CP	Cantaloupe		PT	Protein Mix
CPD	Cantaloupe Dried		R	Raisins
CR	Imitation Crab Meat		RCHE	Royal Canin GI High Energy Diet®
CU	Cucumber		RCLF	Royal Canin GI Low Fat Diet®
D	Dates		RCRS	Royal Canin RS Recovery Diet®
DD	Dates Dried		SB	Strawberries
DG	Diet Gel		SBD	Strawberries Dried
EM	Edamame		SFS	Sunflower Seeds
F	Fig Bars		STK	Sticky Mix
FA	Fava Beans		SO	Soybeans
FD	Figs Dried		SY	Spray Cheese
FNM	Fruit and Nut Medley		TFS	Tropical Fruit Salad
G	Grapes		TH	Timothy Hay
GA	Gatorade®		TP	Tropical Mix
GB	Green Beans		TRL	Tral Mix
GE	Gelatin		UNC	Uncrustables®
GG	Golden Grahams™		WN	Walnuts
GP	Green Pepper		Y	Yam/ Sweet Potato
GR	Granola		YD	Yogurt Drops
GRB	Granola Bars			

2954-001

A Single Dose Toxicity Study of AAV9/SURF1 Administered by Intrathecal Injection in Rats

Group 1	Individual Animal Exam Observation and Treatment Report: Female			
	Study Day	Animal Number	Activity	Entry Summary
	21	1523	Assessment	Animal submitted for suspected dehydration and hunched posture. Animal has NAD and was moved to a functioning sipper prior to examination. Offer FE once. Monitor and re-evaluate in 3 days.
	21	1523	Examination	Obs: Other; Obs: NAD
	21	1523	Examination	Obs: Other; Obs: Sipper Function; Obs: Normal
	21	1523	Treatment	Category: Enrichment; Treatment: Food Enrichment; Route: Offer; Frequency: Once; Treatment Date: 17-MAY-21; Date and Time Performed: 17-MAY-21, 02:05:00 PM; Comments: 1 unit DG offered to cage
	23	1523	Examination	Obs: Other; Obs: NAD
	23	1523	Examination	Obs: Other; Obs: Sipper Function; Obs: Normal
	23	1523	Reassessment	Animal continues to have NAD. No further vet care required at this time.
	21	1524	Assessment	Animal submitted for suspected dehydration and hunched posture. Animal has NAD and was moved to a functioning sipper prior to examination. Offer FE once. Monitor and re-evaluate in 3 days.
	21	1524	Examination	Obs: Other; Obs: NAD
	21	1524	Examination	Obs: Other; Obs: Sipper Function; Obs: Normal
	21	1524	Treatment	Category: Enrichment; Treatment: Food Enrichment; Route: Offer; Frequency: Once; Treatment Date: 17-MAY-21; Date and Time Performed: 17-MAY-21, 02:07:00 PM; Comments: 1 unit DG offered to cage
	23	1524	Examination	Obs: Other; Obs: NAD
	23	1524	Examination	Obs: Other; Obs: Sipper Function; Obs: Normal
	23	1524	Reassessment	Animal continues to have NAD. No further vet care required at this time.
	20	1525	Assessment	Animal submitted for suspected dehydration and hunched posture. Animal has NAD and was moved to a functioning sipper prior to examination. Offer FE once. Monitor and re-evaluate in 3 days.
	20	1525	Examination	Obs: Other; Obs: NAD
	20	1525	Examination	Obs: Other; Obs: Sipper Function; Obs: Normal
	20	1525	Treatment	Category: Enrichment; Treatment: Food Enrichment; Route: Offer; Frequency: Once; Treatment Date: 17-MAY-21; Date and Time Performed: 17-MAY-21, 02:08:00 PM; Comments: 1 unit DG offered to cage

2954-001

A Single Dose Toxicity Study of AAV9/SURF1 Administered by Intrathecal Injection in Rats

	Individual Animal Exam Observation and Treatment Report: Female			
Group 1	Study Day	Animal Number	Activity	Entry Summary
	22	1525	Examination	Obs: Other; Obs: NAD
	22	1525	Examination	Obs: Other; Obs: Sipper Function; Obs: Normal
	22	1525	Reassessment	Animal continues to have NAD. No further vet care required at this time.

2954-001

A Single Dose Toxicity Study of AAV9/SURF1 Administered by Intrathecal Injection in Rats

Group 2	Individual Animal Exam Observation and Treatment Report: Female			
	Study Day	Animal Number	Activity	Entry Summary
	21	2523	Assessment	Animal submitted for suspected dehydration and hunched posture. Animal has NAD and was moved to a functioning sipper prior to examination. Offer FE once. Monitor and re-evaluate in 3 days.
	21	2523	Examination	Obs: Other; Obs: NAD
	21	2523	Examination	Obs: Other; Obs: Sipper Function; Obs: Normal
	21	2523	Treatment	Category: Enrichment; Treatment: Food Enrichment; Route: Offer; Frequency: Once; Treatment Date: 17-MAY-21; Date and Time Performed: 17-MAY-21, 02:13:00 PM; Comments: 1 unit DG offered to cage
	23	2523	Examination	Obs: Other; Obs: NAD
	23	2523	Examination	Obs: Other; Obs: Sipper Function; Obs: Normal
	23	2523	Reassessment	Animal continues to have NAD. No further vet care required at this time.
	21	2524	Assessment	Animal submitted for suspected dehydration and hunched posture. Animal has NAD and was moved to a functioning sipper prior to examination. Offer FE once. Monitor and re-evaluate in 3 days.
	21	2524	Examination	Obs: Other; Obs: NAD
	21	2524	Examination	Obs: Other; Obs: Sipper Function; Obs: Normal
	21	2524	Treatment	Category: Enrichment; Treatment: Food Enrichment; Route: Offer; Frequency: Once; Treatment Date: 17-MAY-21; Date and Time Performed: 17-MAY-21, 02:14:00 PM; Comments: 1 unit DG offered to cage
	23	2524	Examination	Obs: Other; Obs: NAD
	23	2524	Examination	Obs: Other; Obs: Sipper Function; Obs: Normal
	23	2524	Reassessment	Animal continues to have NAD. No further vet care required at this time.

2954-001

A Single Dose Toxicity Study of AAV9/SURF1 Administered by Intrathecal Injection in Rats

Group 3	Individual Animal Exam Observation and Treatment Report: Male			
	Study Day	Animal Number	Activity	Entry Summary
	14	3008	Assessment	Animal noted with lameness in both hind limbs with intermittently severe righting reflex and tremoring. Offer FE. Monitor daily. Re-evaluate within 3 days.
	14	3008	Examination	Obs: Activity/Gait/Locomotion; Obs: Activity Abnormal; Obs: Decreased, mild
	14	3008	Examination	Obs: Activity/Gait/Locomotion; Obs: Righting Reflex; Obs: Decreased, mild
	14	3008	Examination	Obs: Activity/Gait/Locomotion; Obs: Righting Reflex; Obs: Intermittent; Obs: Severe
	14	3008	Examination	Obs: Activity/Gait/Locomotion; Obs: Tremors; Obs: Moderate
	14	3008	Examination	Obs: Attitude; Obs: Bright, Alert, Responsive
	14	3008	Examination	Obs: General Body Condition; Obs: 2.5 - Lean
	14	3008	Examination	Obs: Hydration; Obs: Hydration, Normal
	14	3008	Examination	Obs: Musculoskeletal System; Obs: Lameness; Obs: Hindlimb; Obs: Bilateral
	14	3008	Examination	Obs: Musculoskeletal System; Obs: Muscle Tone; Obs: Hindlimb; Obs: Bilateral; Obs: Increased, mild
	14	3008	Treatment	Category: Enrichment; Treatment: Food Enrichment; Route: Offer PRN; Frequency: SID; Treatment Date: 08-SEP-20; Date and Time Performed: 08-SEP-20, 09:54:00 AM; Comments: 1 jar baby food
	15	3008	Examination	Obs: Activity/Gait/Locomotion; Obs: Activity Abnormal; Obs: Decreased, mild
	15	3008	Examination	Obs: Activity/Gait/Locomotion; Obs: Righting Reflex; Obs: Decreased, moderate
	15	3008	Examination	Obs: Appetite; Obs: Appetite, Normal, Food Enrichment
	15	3008	Examination	Obs: Attitude; Obs: Bright, Alert, Responsive
	15	3008	Examination	Obs: Gastrointestinal System; Obs: Feces, Normal
	15	3008	Examination	Obs: General Body Condition; Obs: 2.5 - Lean
	15	3008	Examination	Obs: Hydration; Obs: Hydration, Normal
	15	3008	Examination	Obs: Musculoskeletal System; Obs: Lameness; Obs: Hindlimb; Obs: Bilateral; Obs: Moderate
	15	3008	Treatment	Category: Assessment; Treatment: Monitor; Frequency: Daily; Treatment Date: 09-SEP-20; Date and Time Performed: 09-SEP-20, 10:32:00 AM
	15	3008	Treatment	Category: Enrichment; Treatment: Food Enrichment; Route: Offer PRN; Frequency: SID; Treatment Date: 09-SEP-20; Date and Time Performed: 09-SEP-20, 10:32:00 AM; Comments: 1 Jar BF
	16	3008	Examination	Obs: Activity/Gait/Locomotion; Obs: Activity Abnormal; Obs: Decreased, mild

2954-001

A Single Dose Toxicity Study of AAV9/SURF1 Administered by Intrathecal Injection in Rats

Group 3	Individual Animal Exam Observation and Treatment Report: Male			
	Study Day	Animal Number	Activity	Entry Summary
	16	3008	Examination	Obs: Activity/Gait/Locomotion; Obs: Paralysis, Rigid
	16	3008	Examination	Obs: Activity/Gait/Locomotion; Obs: Righting Reflex; Obs: Decreased, severe
	16	3008	Examination	Obs: Appetite; Obs: Appetite, Normal, Food Enrichment
	16	3008	Examination	Obs: Attitude; Obs: Bright, Alert, Responsive
	16	3008	Examination	Obs: Gastrointestinal System; Obs: Feces, Normal
	16	3008	Examination	Obs: General Body Condition; Obs: 2.5 - Lean
	16	3008	Examination	Obs: Hydration; Obs: Hydration, Normal
	16	3008	Examination	Obs: Musculoskeletal System; Obs: Muscle Tone; Obs: Hindlimb; Obs: Bilateral; Obs: Moderate
	16	3008	Examination	Obs: Other; Obs: No Change
	16	3008	Reassessment	Animals condition has not improved. Animal is approved for 24hr euthanasia.
	16	3008	Treatment	Category: Assessment; Treatment: Monitor; Frequency: Daily; Treatment Date: 10-SEP-20; Date and Time Performed: 10-SEP-20, 08:34:00 AM
	14	3022	Assessment	Animal submitted for discharge from penis. Upon exam, no discharge was noted and animal has NAD. No further vet care required at this time.
	14	3022	Examination	Obs: Other; Obs: NAD
	14	3022	Reassessment	na

2954-001

A Single Dose Toxicity Study of AAV9/SURF1 Administered by Intrathecal Injection in Rats

Group 3	Individual Animal Exam Observation and Treatment Report: Female			
	Study Day	Animal Number	Activity	Entry Summary
	1	3521	Assessment	Animal submitted for decreased activity and respiratory distress. Upon exam, animal appears to be slow to recover from anesthesia. Offer FE once. Monitor and re-evaluate within 1 day for full recovery.
	1	3521	Examination	Obs: Activity/Gait/Locomotion; Obs: Recently Sedated; Obs: For IT dose
	1	3521	Examination	Obs: Attitude; Obs: Attitude, Other (See comment); Obs: Normal reaction to handling
	1	3521	Examination	Obs: Heart/Lungs; Obs: Heart/Lungs, Normal
	1	3521	Treatment	Category: Enrichment; Treatment: Food Enrichment; Route: Offer; Frequency: Once; Treatment Date: 26-APR-21; Date and Time Performed: 26-APR-21, 02:09:00 PM; Comments: 1 jar baby food
	2	3521	Examination	Obs: Activity/Gait/Locomotion; Obs: Activity, Normal
	2	3521	Examination	Obs: Appetite; Obs: Appetite, Normal, Food Enrichment
	2	3521	Examination	Obs: Attitude; Obs: Bright, Alert, Responsive
	2	3521	Examination	Obs: Gastrointestinal System; Obs: Feces, Normal
	2	3521	Examination	Obs: General Body Condition; Obs: 3 - Optimum
	2	3521	Examination	Obs: Heart/Lungs; Obs: Heart/Lungs, Normal
	2	3521	Reassessment	Animal has improved and appears normal and healthy at this time. No further veterinary care required at this time.
	21	3523	Assessment	Animal noted with several firm swellings in axillary, inguinal, and urogenital regions. Animal appears unaffected and non-painful. Monitor weekly and re-evaluate monthly.
	21	3523	Examination	Obs: Activity/Gait/Locomotion; Obs: Activity/Gait/Locomotion, Normal
	21	3523	Examination	Obs: Attitude; Obs: Bright, Alert, Responsive
	21	3523	Examination	Obs: General Body Condition; Obs: 3 - Optimum
	21	3523	Examination	Obs: Hydration; Obs: Hydration, Normal
	21	3523	Examination	Obs: Integumentary System; Obs: Swelling; Obs: Axillary; Obs: Bilateral; Obs: Medium; Obs: Firm
	21	3523	Examination	Obs: Integumentary System; Obs: Swelling; Obs: Inguinal; Obs: Left; Obs: Small; Obs: Firm; Obs: several
	21	3523	Examination	Obs: Integumentary System; Obs: Swelling; Obs: Inguinal; Obs: Right; Obs: Medium; Obs: Firm

2954-001

A Single Dose Toxicity Study of AAV9/SURF1 Administered by Intrathecal Injection in Rats

Group 3	Individual Animal Exam Observation and Treatment Report: Female			
	Study Day	Animal Number	Activity	Entry Summary
	21	3523	Examination	Obs: Integumentary System; Obs: Swelling; Obs: Urogenital; Obs: Medium; Obs: Firm
	21	3523	Examination	Obs: Pain Assessment; Obs: Non-painful
	28	3523	Examination	Obs: Activity/Gait/Locomotion; Obs: Activity, Normal
	28	3523	Examination	Obs: Attitude; Obs: Bright, Alert, Responsive
	28	3523	Examination	Obs: Integumentary System; Obs: Skin, Nodule; Obs: Axillary; Obs: Bilateral; Obs: Small; Obs: Firm
	28	3523	Examination	Obs: Integumentary System; Obs: Skin, Nodule; Obs: Inguinal; Obs: Bilateral; Obs: Small
	28	3523	Examination	Obs: Integumentary System; Obs: Skin, Nodule; Obs: Neck; Obs: Bilateral; Obs: Small; Obs: Firm
	28	3523	Examination	Obs: Pain Assessment; Obs: Non-painful
	28	3523	Treatment	Category: Assessment; Treatment: Monitor; Frequency: Weekly; Treatment Date: 24-MAY-21; Date and Time Performed: 24-MAY-21, 12:33:00 PM
	35	3523	Reassessment	Animal confirmed deceased in Provantis.
	14	3525	Assessment	Animal reported for abnormal gait and hind limb lameness, confirmed upon exam. Animal appears non painful and otherwise normal. Offer FE. Monitor and re-evaluate in 1 week.
	14	3525	Examination	Obs: Activity/Gait/Locomotion; Obs: Activity, Normal
	14	3525	Examination	Obs: Activity/Gait/Locomotion; Obs: Gait Abnormal; Obs: Hindlimb; Obs: Bilateral
	14	3525	Examination	Obs: Attitude; Obs: Bright, Alert, Responsive
	14	3525	Examination	Obs: Gastrointestinal System; Obs: Feces, Normal
	14	3525	Examination	Obs: General Body Condition; Obs: 3 - Optimum
	14	3525	Examination	Obs: Hydration; Obs: Hydration, Normal
	14	3525	Examination	Obs: Musculoskeletal System; Obs: Lameness; Obs: Hindlimb; Obs: Bilateral; Obs: Severe; Obs: dragging limbs
	14	3525	Examination	Obs: Other; Obs: General Observation; Obs: animal observed rearing up for access to food/water
	14	3525	Examination	Obs: Pain Assessment; Obs: Non-painful
	14	3525	Treatment	Category: Enrichment; Treatment: Food Enrichment; Route: Offer PRN; Frequency: SID; Treatment Date: 11-MAY-21; Date and Time Performed: 11-MAY-21, 12:16:00 PM; Comments: 2 units DG

2954-001

A Single Dose Toxicity Study of AAV9/SURF1 Administered by Intrathecal Injection in Rats

Group 3	Individual Animal Exam Observation and Treatment Report: Female			
	Study Day	Animal Number	Activity	Entry Summary
	15	3525	Treatment	Category: Enrichment; Treatment: Food Enrichment; Route: Offer PRN; Frequency: SID; Treatment Date: 12-MAY-21; Date and Time Performed: 12-MAY-21, 08:19:00 AM; Comments: 2 jars baby food
	16	3525	Treatment	Category: Enrichment; Treatment: Food Enrichment; Route: Offer PRN; Frequency: SID; Treatment Date: 13-MAY-21; Date and Time Performed: 13-MAY-21, 10:07:00 AM; Comments: 2 units DG
	17	3525	Treatment	Category: Enrichment; Treatment: Food Enrichment; Route: Offer PRN; Frequency: SID; Treatment Date: 14-MAY-21; Date and Time Performed: 14-MAY-21, 12:59:00 PM; Comments: 2 units DG
	18	3525	Treatment	Category: Enrichment; Treatment: Food Enrichment; Route: Offer PRN; Frequency: SID; Treatment Date: 15-MAY-21; Date and Time Performed: 15-MAY-21, 07:47:00 AM; Comments: 2 units DG
	19	3525	Treatment	Category: Enrichment; Treatment: Food Enrichment; Route: Offer PRN; Frequency: SID; Treatment Date: 16-MAY-21; Date and Time Performed: 16-MAY-21, 08:53:00 AM; Comments: 2 units DG
	20	3525	Treatment	Category: Enrichment; Treatment: Food Enrichment; Route: Offer PRN; Frequency: SID; Treatment Date: 17-MAY-21; Date and Time Performed: 17-MAY-21, 08:16:00 AM; Comments: 1 unit DG
	21	3525	Examination	Obs: Activity/Gait/Locomotion; Obs: Activity, Normal
	21	3525	Examination	Obs: Activity/Gait/Locomotion; Obs: Carriage Low
	21	3525	Examination	Obs: Activity/Gait/Locomotion; Obs: Gait Abnormal; Obs: Hindlimb; Obs: Bilateral; Obs: Mild; Obs: hindlimbs trail slightly
	21	3525	Examination	Obs: Appetite; Obs: Appetite, Normal, Food Enrichment
	21	3525	Examination	Obs: Attitude; Obs: Bright, Alert, Responsive
	21	3525	Examination	Obs: General Body Condition; Obs: 3 - Optimum
	21	3525	Examination	Obs: Hydration; Obs: Hydration, Normal
	21	3525	Reassessment	Animal appears to be improving. Continue FE. Monitor weekly and re-evaluate in 2 weeks.
	21	3525	Treatment	Category: Enrichment; Treatment: Food Enrichment; Route: Offer; Frequency: SID; Treatment Date: 18-MAY-21; Date and Time Performed: 18-MAY-21, 08:47:00 AM; Comments: 2 unit DG
	22	3525	Treatment	Category: Enrichment; Treatment: Food Enrichment; Route: Offer; Frequency: SID; Treatment Date: 19-MAY-21; Date and Time Performed: 19-MAY-21, 08:11:00 AM; Comments: 2 unit DG
	23	3525	Treatment	Category: Enrichment; Treatment: Food Enrichment; Route: Offer; Frequency: SID; Treatment Date: 20-MAY-21; Date and Time Performed: 20-MAY-21, 07:50:00 AM; Comments: 2 unit DG

2954-001

A Single Dose Toxicity Study of AAV9/SURF1 Administered by Intrathecal Injection in Rats

Group 3	Individual Animal Exam Observation and Treatment Report: Female			
	Study Day	Animal Number	Activity	Entry Summary
	24	3525	Treatment	Category: Enrichment; Treatment: Food Enrichment; Route: Offer; Frequency: SID; Treatment Date: 21-MAY-21; Date and Time Performed: 21-MAY-21, 08:52:00 AM; Comments: 2 unit DG
	25	3525	Treatment	Category: Enrichment; Treatment: Food Enrichment; Route: Offer; Frequency: SID; Treatment Date: 22-MAY-21; Date and Time Performed: 22-MAY-21, 08:18:00 AM; Comments: 2 units DG
	26	3525	Treatment	Category: Enrichment; Treatment: Food Enrichment; Route: Offer; Frequency: SID; Treatment Date: 23-MAY-21; Date and Time Performed: 23-MAY-21, 07:38:00 AM; Comments: 2 unit DG
	27	3525	Treatment	Category: Enrichment; Treatment: Food Enrichment; Route: Offer; Frequency: SID; Treatment Date: 24-MAY-21; Date and Time Performed: 24-MAY-21, 12:33:00 PM; Comments: 2 units DG for cage
	28	3525	Treatment	Category: Enrichment; Treatment: Food Enrichment; Route: Offer; Frequency: SID; Treatment Date: 25-MAY-21; Date and Time Performed: 25-MAY-21, 09:04:00 AM; Comments: Not offered, animal not present in room and fasted.
	29	3525	Reassessment	Animal confirmed deceased in Provantis.

2954-001

A Single Dose Toxicity Study of AAV9/SURF1 Administered by Intrathecal Injection in Rats

Group 4	Individual Animal Exam Observation and Treatment Report: Male			
	Study Day	Animal Number	Activity	Entry Summary
	1	4113	Assessment	Animal noted with small laceration on tail with very mild swelling, but appears unaffected and non-painful. Offer EE. Monitor 2 times weekly and re-evaluate in 1 week.
	1	4113	Examination	Obs: Activity/Gait/Locomotion; Obs: Activity, Normal
	1	4113	Examination	Obs: Attitude; Obs: Bright, Alert, Responsive
	1	4113	Examination	Obs: Gastrointestinal System; Obs: Feces, Normal
	1	4113	Examination	Obs: General Body Condition; Obs: 3 - Optimum
	1	4113	Examination	Obs: Hydration; Obs: Hydration, Normal
	1	4113	Examination	Obs: Integumentary System; Obs: Skin, Laceration; Obs: Tail; Obs: Small; Obs: Dry
	1	4113	Examination	Obs: Integumentary System; Obs: Swelling; Obs: Tail; Obs: Mild; Obs: Slight swelling at site of laceration
	1	4113	Examination	Obs: Pain Assessment; Obs: Non-painful
	1	4113	Treatment	Category: Assessment; Treatment: Monitor; Frequency: 2X Weekly - Wed; Treatment Date: 28-APR-21; Date and Time Performed: 28-APR-21, 12:38:00 PM
	1	4113	Treatment	Category: Enrichment; Treatment: Environmental Enrichment; Route: Offer PRN; Frequency: 2X Weekly - Wed; Treatment Date: 28-APR-21; Date and Time Performed: 28-APR-21, 12:37:00 PM; Comments: 3 DT
	3	4113	Examination	Obs: Activity/Gait/Locomotion; Obs: Activity, Normal
	3	4113	Examination	Obs: Appetite; Obs: Appetite, Normal, Basal Diet
	3	4113	Examination	Obs: Attitude; Obs: Bright, Alert, Responsive
	3	4113	Examination	Obs: Gastrointestinal System; Obs: Feces, Normal
	3	4113	Examination	Obs: Hydration; Obs: Hydration, Normal
	3	4113	Examination	Obs: Integumentary System; Obs: Skin, Laceration; Obs: Tail; Obs: Superficial; Obs: Small; Obs: Dry
	3	4113	Examination	Obs: Integumentary System; Obs: Swelling; Obs: Tail; Obs: Mild; Obs: Around laceration
	3	4113	Examination	Obs: Pain Assessment; Obs: Non-painful
	3	4113	Treatment	Category: Assessment; Treatment: Monitor; Frequency: 2X Weekly - Wed; Treatment Date: 30-APR-21; Date and Time Performed: 30-APR-21, 09:40:00 AM

2954-001

A Single Dose Toxicity Study of AAV9/SURF1 Administered by Intrathecal Injection in Rats

Group 4	Individual Animal Exam Observation and Treatment Report: Male			
	Study Day	Animal Number	Activity	Entry Summary
	3	4113	Treatment	Category: Enrichment; Treatment: Environmental Enrichment; Route: Offer PRN; Frequency: 2X Weekly - Wed; Treatment Date: 30-APR-21; Date and Time Performed: 30-APR-21, 09:40:00 AM; Comments: 2 DT
	8	4113	Reassessment	This animal has been confirmed deceased in Provantis.

2954-001

A Single Dose Toxicity Study of AAV9/SURF1 Administered by Intrathecal Injection in Rats

Group 4	Individual Animal Exam Observation and Treatment Report: Female			
	Study Day	Animal Number	Activity	Entry Summary
	21	4521	Assessment	Animal submitted for suspected dehydration and hunched posture. Animal has NAD and was moved to a functioning sipper prior to examination. Offer FE once. Monitor and re-evaluate in 3 days.
	21	4521	Examination	Obs: Other; Obs: NAD
	21	4521	Examination	Obs: Other; Obs: Sipper Function; Obs: Normal
	21	4521	Treatment	Category: Enrichment; Treatment: Food Enrichment; Route: Offer; Frequency: Once; Treatment Date: 17-MAY-21; Date and Time Performed: 17-MAY-21, 02:21:00 PM; Comments: 1 unit DG to cage
	21	4521	Treatment	Category: Enrichment; Treatment: Food Enrichment; Route: Offer; Frequency: Once; Treatment Date: 19-MAY-21; Date and Time Performed: 17-MAY-21, 08:24:00 AM; Comments: 1 unit DG to cage
	23	4521	Examination	Obs: Other; Obs: NAD
	23	4521	Examination	Obs: Other; Obs: Sipper Function; Obs: Absent; Obs: at original location of poly
	23	4521	Reassessment	Sipper malfunction noted at original location of poly and repaired during exam. Animal continues to have NAD. Offer FE once. No further vet care required at this time.
	21	4522	Assessment	Animal submitted for suspected dehydration and hunched posture. Animal has NAD and was moved to a functioning sipper prior to examination. Offer FE once. Monitor and re-evaluate in 3 days.
	21	4522	Examination	Obs: Other; Obs: NAD
	21	4522	Examination	Obs: Other; Obs: Sipper Function; Obs: Normal
	21	4522	Treatment	Category: Enrichment; Treatment: Food Enrichment; Route: Offer; Frequency: Once; Treatment Date: 17-MAY-21; Date and Time Performed: 17-MAY-21, 02:23:00 PM; Comments: 1 unit DG offered to cage
	21	4522	Treatment	Category: Enrichment; Treatment: Food Enrichment; Route: Offer; Frequency: Once; Treatment Date: 19-MAY-21; Date and Time Performed: 17-MAY-21, 08:25:00 AM; Comments: 1 unit DG offered to cage
	23	4522	Examination	Obs: Other; Obs: NAD
	23	4522	Examination	Obs: Other; Obs: Sipper Function; Obs: Absent; Obs: at original location of poly
	23	4522	Reassessment	Sipper malfunction noted at original location of poly and repaired during exam. Animal continues to have NAD. Offer FE once. No further vet care required at this time.

Appendix 6

Individual Body Weights

2954-001
Sex: Male Bodyweight (g)

Group 1	Day(s) Relative to Start Date						
	-1	7	14	21	28	35	42
1006	209	276	341	392	428	473	500
1007	194	255	320	368	414	447	476
1008	216	287	352	390	430	464	491
1009	193	267	324	346	411	445	475
1010	204	286	342	387	436	474	507
1011	208	267	-	-	-	-	-
1012	210	260	-	-	-	-	-
1013	223	285	-	-	-	-	-
1014	218	278	-	-	-	-	-
1015	235	294	-	-	-	-	-
1021	225	285	321	306	375	-	-
1022	214	269	313	294	365	-	-
1023	217	268	310	330	358	-	-
1024	231	288	333	370	410	-	-
1025	221	269	313	350	371	-	-
Mean	214.5	275.6	326.9	353.3	399.8	460.6	489.8
SD	12.0	11.6	14.4	34.7	29.5	13.9	14.2
N	15	15	10	10	10	5	5

Appendix 6

Individual Body Weights

2954-001
Sex: Male Bodyweight (g)

Group 1	Day(s) Relative to Start Date					
	49	56	63	70	77	91
1006	540	570	616	628	651	666
1007	505	527	543	565	579	593
1008	522	531	553	572	590	603
1009	495	524	541	554	553	561
1010	553	584	610	635	634	655
1011	-	-	-	-	-	-
1012	-	-	-	-	-	-
1013	-	-	-	-	-	-
1014	-	-	-	-	-	-
1015	-	-	-	-	-	-
1021	-	-	-	-	-	-
1022	-	-	-	-	-	-
1023	-	-	-	-	-	-
1024	-	-	-	-	-	-
1025	-	-	-	-	-	-
Mean	523.0	547.2	572.6	590.8	601.4	615.6
SD	24.0	27.8	37.2	37.8	40.3	44.0
N	5	5	5	5	5	5

Appendix 6

Individual Body Weights

2954-001
Sex: Male Bodyweight (g)

Group 2	Day(s) Relative to Start Date						
	-1	7	14	21	28	35	42
2006	200	266	338	389	437	469	506
2007	201	268	333	373	416	449	469
2008	220	284	355	406	458	503	531
2009	193	260	316	353	396	418	447
2010	222	292	356	404	442	479	503
2011	206	270	-	-	-	-	-
2012	221	286	-	-	-	-	-
2013	219	291	-	-	-	-	-
2014	248	311	-	-	-	-	-
2015	201	263	-	-	-	-	-
2021	218	274	315	300	370	-	-
2022	220	276	315	305	387	-	-
2023	205	256	304	345	375	-	-
2024	227	300	355	400	440	-	-
2025	247	301	355	404	433	-	-
Mean	216.5	279.9	334.2	367.9	415.4	463.6	491.2
SD	16.2	16.6	20.5	40.7	31.2	32.1	33.1
N	15	15	10	10	10	5	5

Appendix 6

Individual Body Weights

2954-001
Sex: Male Bodyweight (g)

Group 2	Day(s) Relative to Start Date					
	49	56	63	70	77	91
2006	525	549	570	587	594	620
2007	500	532	540	561	576	589
2008	569	589	611	643	642	660
2009	468	483	504	526	542	557
2010	531	547	566	585	605	622
2011	-	-	-	-	-	-
2012	-	-	-	-	-	-
2013	-	-	-	-	-	-
2014	-	-	-	-	-	-
2015	-	-	-	-	-	-
2021	-	-	-	-	-	-
2022	-	-	-	-	-	-
2023	-	-	-	-	-	-
2024	-	-	-	-	-	-
2025	-	-	-	-	-	-
Mean	518.6	540.0	558.2	580.4	591.8	609.6
SD	37.6	38.2	39.5	42.8	36.8	38.7
N	5	5	5	5	5	5

Appendix 6

Individual Body Weights

2954-001
Sex: Male Bodyweight (g)

Group 3	Day(s) Relative to Start Date						
	-1	7	14	21	28	35	42
3006	194	253	308	350	398	430	453
3007	195	263	328	360	431	477	509
3008	175	225	233	-	-	-	-
3009	199	266	338	375	421	455	478
3010	205	263	319	362	407	440	454
3011	253	320	-	-	-	-	-
3012	252	316	-	-	-	-	-
3013	214	271	-	-	-	-	-
3014	233	302	-	-	-	-	-
3015	186	245	-	-	-	-	-
4013	197	254	-	-	-	-	-
3021	205	259	299	312	361	-	-
3022	211	270	317	315	355	-	-
3023	252	316	360	403	439	-	-
3024	237	306	357	389	428	-	-
3025	210	260	308	345	378	-	-
Mean	213.6	274.3	316.7	356.8	402.0	450.5	473.5
SD	24.6	28.6	35.8	30.6	31.1	20.4	26.3
N	16	16	10	9	9	4	4

Appendix 6

Individual Body Weights

2954-001
Sex: Male Bodyweight (g)

Group 3	Day(s) Relative to Start Date					
	49	56	63	70	77	91
3006	482	499	521	543	559	574
3007	540	563	581	605	627	647
3008	-	-	-	-	-	-
3009	512	536	557	571	598	611
3010	496	506	519	533	549	558
3011	-	-	-	-	-	-
3012	-	-	-	-	-	-
3013	-	-	-	-	-	-
3014	-	-	-	-	-	-
3015	-	-	-	-	-	-
4013	-	-	-	-	-	-
3021	-	-	-	-	-	-
3022	-	-	-	-	-	-
3023	-	-	-	-	-	-
3024	-	-	-	-	-	-
3025	-	-	-	-	-	-
Mean	507.5	526.0	544.5	563.0	583.3	597.5
SD	24.9	29.4	29.9	32.3	36.0	39.8
N	4	4	4	4	4	4

Appendix 6

Individual Body Weights

2954-001
Sex: Male Bodyweight (g)

Group 4	Day(s) Relative to Start Date						
	-1	7	14	21	28	35	42
4006	206	283	347	378	419	457	482
4007	186	247	293	308	367	392	411
4008	209	273	334	367	413	440	458
4009	182	244	307	348	388	412	443
4010	187	246	305	334	380	412	435
4011	229	284	-	-	-	-	-
4012	224	278	-	-	-	-	-
4113	233	287	-	-	-	-	-
4021	244	290	-	-	-	-	-
4022	217	268	-	-	-	-	-
4023	230	299	345	385	418	-	-
4024	218	264	300	328	351	-	-
4025	233	290	335	372	410	-	-
4026	255	308	357	395	431	-	-
4027	242	302	349	383	416	-	-
Mean	219.7	277.5	327.2	359.8	399.3	422.6	445.8
SD	22.1	20.4	23.5	28.8	26.2	25.7	26.4
N	15	15	10	10	10	5	5

Appendix 6

Individual Body Weights

2954-001
Sex: Male Bodyweight (g)

Group 4	Day(s) Relative to Start Date					
	49	56	63	70	77	91
4006	521	533	558	579	595	604
4007	434	451	466	486	498	509
4008	494	502	520	538	538	550
4009	471	497	514	524	539	555
4010	467	481	507	517	531	539
4011	-	-	-	-	-	-
4012	-	-	-	-	-	-
4113	-	-	-	-	-	-
4021	-	-	-	-	-	-
4022	-	-	-	-	-	-
4023	-	-	-	-	-	-
4024	-	-	-	-	-	-
4025	-	-	-	-	-	-
4026	-	-	-	-	-	-
4027	-	-	-	-	-	-
Mean	477.4	492.8	513.0	528.8	540.2	551.4
SD	32.4	30.0	32.9	33.9	34.9	34.4
N	5	5	5	5	5	5

Appendix 6

Individual Body Weights

2954-001
Sex: Female Bodyweight (g)

Group 1	Day(s) Relative to Start Date						
	-1	7	14	21	28	35	42
1506	150	173	195	213	224	226	235
1507	159	188	206	219	239	251	257
1508	173	190	209	215	231	247	248
1509	185	215	238	262	282	300	310
1510	143	167	193	203	210	226	232
1511	170	195	-	-	-	-	-
1512	206	237	-	-	-	-	-
1513	175	198	-	-	-	-	-
1514	206	230	-	-	-	-	-
1515	207	234	-	-	-	-	-
1521	181	193	219	229	239	-	-
1522	189	217	238	261	263	-	-
1523	209	234	244	205	266	-	-
1524	170	181	205	173	218	-	-
1525	198	228	252	246	264	-	-
Mean	181.4	205.3	219.9	222.6	243.6	250.0	256.4
SD	21.2	23.8	21.5	27.8	23.9	30.3	31.6
N	15	15	10	10	10	5	5

Appendix 6

Individual Body Weights

2954-001
Sex: Female Bodyweight (g)

Group 1	Day(s) Relative to Start Date					
	49	56	63	70	77	91
1506	247	244	248	245	262	269
1507	257	266	277	274	272	285
1508	250	262	269	269	255	273
1509	322	322	330	336	344	346
1510	240	240	252	263	264	269
1511	-	-	-	-	-	-
1512	-	-	-	-	-	-
1513	-	-	-	-	-	-
1514	-	-	-	-	-	-
1515	-	-	-	-	-	-
1521	-	-	-	-	-	-
1522	-	-	-	-	-	-
1523	-	-	-	-	-	-
1524	-	-	-	-	-	-
1525	-	-	-	-	-	-
Mean	263.2	266.8	275.2	277.4	279.4	288.4
SD	33.4	32.8	32.9	34.5	36.6	32.9
N	5	5	5	5	5	5

Appendix 6

Individual Body Weights

2954-001
Sex: Female Bodyweight (g)

Group 2	Day(s) Relative to Start Date						
	-1	7	14	21	28	35	42
2506	162	194	232	250	272	293	295
2507	160	185	199	218	229	240	240
2508	178	213	226	256	273	289	290
2509	173	203	235	249	262	263	276
2510	181	219	237	267	272	296	297
2511	177	192	-	-	-	-	-
2512	185	206	-	-	-	-	-
2513	178	197	-	-	-	-	-
2514	173	189	-	-	-	-	-
2515	209	236	-	-	-	-	-
2521	176	199	215	232	236	-	-
2522	185	204	219	237	259	-	-
2523	178	202	211	177	248	-	-
2524	194	212	236	196	251	-	-
2525	205	222	235	234	251	-	-
Mean	180.9	204.9	224.5	231.6	255.3	276.2	279.6
SD	13.5	13.7	13.0	27.8	15.2	24.1	23.6
N	15	15	10	10	10	5	5

Appendix 6

Individual Body Weights

2954-001
Sex: Female Bodyweight (g)

Group 2	Day(s) Relative to Start Date					
	49	56	63	70	77	91
2506	310	322	326	344	341	358
2507	264	274	285	285	300	312
2508	310	305	321	327	322	329
2509	287	293	294	308	314	313
2510	311	322	326	335	337	349
2511	-	-	-	-	-	-
2512	-	-	-	-	-	-
2513	-	-	-	-	-	-
2514	-	-	-	-	-	-
2515	-	-	-	-	-	-
2521	-	-	-	-	-	-
2522	-	-	-	-	-	-
2523	-	-	-	-	-	-
2524	-	-	-	-	-	-
2525	-	-	-	-	-	-
Mean	296.4	303.2	310.4	319.8	322.8	332.2
SD	20.7	20.4	19.4	23.6	16.8	20.8
N	5	5	5	5	5	5

Appendix 6

Individual Body Weights

2954-001
Sex: Female Bodyweight (g)

Group 3	Day(s) Relative to Start Date						
	-1	7	14	21	28	35	42
3506	178	211	244	264	284	301	313
3507	180	205	233	249	262	264	282
3508	189	226	241	262	269	278	284
3509	179	211	225	238	241	258	267
3510	175	205	223	230	242	261	264
3511	188	207	-	-	-	-	-
3512	183	198	-	-	-	-	-
3513	169	184	-	-	-	-	-
3514	184	208	-	-	-	-	-
3515	189	206	-	-	-	-	-
3521	169	188	190	205	221	-	-
3522	195	212	224	233	234	-	-
3523	191	215	229	240	228	-	-
3524	182	194	203	215	226	-	-
3525	203	223	222	223	235	-	-
Mean	183.6	206.2	223.4	235.9	244.2	272.4	282.0
SD	9.3	11.6	16.3	19.0	20.7	17.7	19.5
N	15	15	10	10	10	5	5

Appendix 6

Individual Body Weights

2954-001
Sex: Female Bodyweight (g)

Group 3	Day(s) Relative to Start Date					
	49	56	63	70	77	91
3506	331	342	350	360	366	386
3507	291	289	294	308	316	312
3508	299	301	306	308	321	328
3509	282	281	296	304	304	301
3510	270	276	280	289	292	295
3511	-	-	-	-	-	-
3512	-	-	-	-	-	-
3513	-	-	-	-	-	-
3514	-	-	-	-	-	-
3515	-	-	-	-	-	-
3521	-	-	-	-	-	-
3522	-	-	-	-	-	-
3523	-	-	-	-	-	-
3524	-	-	-	-	-	-
3525	-	-	-	-	-	-
Mean	294.6	297.8	305.2	313.8	319.8	324.4
SD	23.0	26.5	26.7	27.0	28.2	36.7
N	5	5	5	5	5	5

Appendix 6

Individual Body Weights

2954-001
Sex: Female Bodyweight (g)

Group 4	Day(s) Relative to Start Date						
	-1	7	14	21	28	35	42
4506	171	204	221	243	253	276	290
4507	146	175	193	208	217	234	243
4508	156	181	205	206	231	248	255
4509	176	212	236	250	254	276	290
4510	163	198	225	243	252	269	282
4511	185	211	-	-	-	-	-
4512	173	189	-	-	-	-	-
4513	180	201	-	-	-	-	-
4514	189	202	-	-	-	-	-
4515	197	221	-	-	-	-	-
4521	190	214	227	192	237	-	-
4522	180	212	236	192	249	-	-
4523	190	199	221	230	238	-	-
4524	195	205	223	233	242	-	-
4525	178	189	198	209	211	-	-
Mean	177.9	200.9	218.5	220.6	238.4	260.6	272.0
SD	14.5	12.9	15.0	21.7	15.0	18.8	21.7
N	15	15	10	10	10	5	5

Appendix 6

Individual Body Weights

2954-001
Sex: Female Bodyweight (g)

Group 4	Day(s) Relative to Start Date					
	49	56	63	70	77	91
4506	298	294	310	310	317	312
4507	255	254	267	268	273	272
4508	262	272	283	284	284	293
4509	303	301	313	322	322	315
4510	292	299	308	323	323	313
4511	-	-	-	-	-	-
4512	-	-	-	-	-	-
4513	-	-	-	-	-	-
4514	-	-	-	-	-	-
4515	-	-	-	-	-	-
4521	-	-	-	-	-	-
4522	-	-	-	-	-	-
4523	-	-	-	-	-	-
4524	-	-	-	-	-	-
4525	-	-	-	-	-	-
Mean	282.0	284.0	296.2	301.4	303.8	301.0
SD	21.9	20.4	20.2	24.4	23.5	18.5
N	5	5	5	5	5	5

Appendix 6

Individual Body Weights

2954-001

Key Page

Measurement Descriptions

Headings Used	Description
Bodyweight	Bodyweight

Measurement/Statistics

Measurement	Descriptive
Bodyweight	Mean
	Standard Deviation
	Count

Group Information

Short Name	Long Name	Type	Report Headings 1-4
1	Group 1	Control	Group 1
2	Group 2	Dose	Group 2
3	Group 3	Dose	Group 3
4	Group 4	Dose	Group 4

Appendix 7

Individual Body Weight Gains (g)

2954-001
Sex: Male Bodyweight Gain (Interval)

Group 1	Day(s) Relative to Start Date						
	-1 → 7	7 → 14	14 → 21	21 → 28	28 → 35	35 → 42	42 → 49
1006	67	65	51	36	45	27	40
1007	61	65	48	46	33	29	29
1008	71	65	38	40	34	27	31
1009	74	57	22	65	34	30	20
1010	82	56	45	49	38	33	46
1011	59	-	-	-	-	-	-
1012	50	-	-	-	-	-	-
1013	62	-	-	-	-	-	-
1014	60	-	-	-	-	-	-
1015	59	-	-	-	-	-	-
1021	60	36	-15	69	-	-	-
1022	55	44	-19	71	-	-	-
1023	51	42	20	28	-	-	-
1024	57	45	37	40	-	-	-
1025	48	44	37	21	-	-	-
Mean	61.1	51.9	26.4	46.5	36.8	29.2	33.2
SD	9.3	11.0	25.0	17.1	5.0	2.5	10.1
N	15	10	10	10	5	5	5

Appendix 7

Individual Body Weight Gains (g)

2954-001
Sex: Male Bodyweight Gain (Interval)

Group 1	Day(s) Relative to Start Date				
	49 → 56	56 → 63	63 → 70	70 → 77	77 → 91
1006	30	46	12	23	15
1007	22	16	22	14	14
1008	9	22	19	18	13
1009	29	17	13	-1	8
1010	31	26	25	-1	21
1011	-	-	-	-	-
1012	-	-	-	-	-
1013	-	-	-	-	-
1014	-	-	-	-	-
1015	-	-	-	-	-
1021	-	-	-	-	-
1022	-	-	-	-	-
1023	-	-	-	-	-
1024	-	-	-	-	-
1025	-	-	-	-	-
Mean	24.2	25.4	18.2	10.6	14.2
SD	9.2	12.2	5.6	11.1	4.7
N	5	5	5	5	5

Appendix 7

Individual Body Weight Gains (g)

2954-001
Sex: Male Bodyweight Gain (Interval)

Group 2	Day(s) Relative to Start Date						
	-1 → 7	7 → 14	14 → 21	21 → 28	28 → 35	35 → 42	42 → 49
2006	66	72	51	48	32	37	19
2007	67	65	40	43	33	20	31
2008	64	71	51	52	45	28	38
2009	67	56	37	43	22	29	21
2010	70	64	48	38	37	24	28
2011	64	-	-	-	-	-	-
2012	65	-	-	-	-	-	-
2013	72	-	-	-	-	-	-
2014	63	-	-	-	-	-	-
2015	62	-	-	-	-	-	-
2021	56	41	-15	70	-	-	-
2022	56	39	-10	82	-	-	-
2023	51	48	41	30	-	-	-
2024	73	55	45	40	-	-	-
2025	54	54	49	29	-	-	-
Mean	63.3	56.5	33.7	47.5	33.8	27.6	27.4
SD	6.5	11.6	24.8	16.8	8.3	6.3	7.7
N	15	10	10	10	5	5	5

Appendix 7

Individual Body Weight Gains (g)

2954-001
Sex: Male Bodyweight Gain (Interval)

Group 2	Day(s) Relative to Start Date				
	49 → 56	56 → 63	63 → 70	70 → 77	77 → 91
2006	24	21	17	7	26
2007	32	8	21	15	13
2008	20	22	32	-1	18
2009	15	21	22	16	15
2010	16	19	19	20	17
2011	-	-	-	-	-
2012	-	-	-	-	-
2013	-	-	-	-	-
2014	-	-	-	-	-
2015	-	-	-	-	-
2021	-	-	-	-	-
2022	-	-	-	-	-
2023	-	-	-	-	-
2024	-	-	-	-	-
2025	-	-	-	-	-
Mean	21.4	18.2	22.2	11.4	17.8
SD	6.9	5.8	5.8	8.4	5.0
N	5	5	5	5	5

Appendix 7

Individual Body Weight Gains (g)

2954-001
Sex: Male Bodyweight Gain (Interval)

Group 3	Day(s) Relative to Start Date						
	-1 → 7	7 → 14	14 → 21	21 → 28	28 → 35	35 → 42	42 → 49
3006	59	55	42	48	32	23	29
3007	68	65	32	71	46	32	31
3008	50	8	-	-	-	-	-
3009	67	72	37	46	34	23	34
3010	58	56	43	45	33	14	42
3011	67	-	-	-	-	-	-
3012	64	-	-	-	-	-	-
3013	57	-	-	-	-	-	-
3014	69	-	-	-	-	-	-
3015	59	-	-	-	-	-	-
4013	57	-	-	-	-	-	-
3021	54	40	13	49	-	-	-
3022	59	47	-2	40	-	-	-
3023	64	44	43	36	-	-	-
3024	69	51	32	39	-	-	-
3025	50	48	37	33	-	-	-
Mean	60.7	48.6	30.8	45.2	36.3	23.0	34.0
SD	6.4	17.2	15.4	11.1	6.6	7.3	5.7
N	16	10	9	9	4	4	4

Appendix 7

Individual Body Weight Gains (g)

2954-001
Sex: Male Bodyweight Gain (Interval)

Group 3	Day(s) Relative to Start Date				
	49 → 56	56 → 63	63 → 70	70 → 77	77 → 91
3006	17	22	22	16	15
3007	23	18	24	22	20
3008	-	-	-	-	-
3009	24	21	14	27	13
3010	10	13	14	16	9
3011	-	-	-	-	-
3012	-	-	-	-	-
3013	-	-	-	-	-
3014	-	-	-	-	-
3015	-	-	-	-	-
4013	-	-	-	-	-
3021	-	-	-	-	-
3022	-	-	-	-	-
3023	-	-	-	-	-
3024	-	-	-	-	-
3025	-	-	-	-	-
Mean	18.5	18.5	18.5	20.3	14.3
SD	6.5	4.0	5.3	5.3	4.6
N	4	4	4	4	4

Appendix 7

Individual Body Weight Gains (g)

2954-001
Sex: Male Bodyweight Gain (Interval)

Group 4	Day(s) Relative to Start Date						
	-1 → 7	7 → 14	14 → 21	21 → 28	28 → 35	35 → 42	42 → 49
4006	77	64	31	41	38	25	39
4007	61	46	15	59	25	19	23
4008	64	61	33	46	27	18	36
4009	62	63	41	40	24	31	28
4010	59	59	29	46	32	23	32
4011	55	-	-	-	-	-	-
4012	54	-	-	-	-	-	-
4113	54	-	-	-	-	-	-
4021	46	-	-	-	-	-	-
4022	51	-	-	-	-	-	-
4023	69	46	40	33	-	-	-
4024	46	36	28	23	-	-	-
4025	57	45	37	38	-	-	-
4026	53	49	38	36	-	-	-
4027	60	47	34	33	-	-	-
Mean	57.9	51.6	32.6	39.5	29.2	23.2	31.6
SD	8.2	9.5	7.6	9.7	5.8	5.2	6.3
N	15	10	10	10	5	5	5

Appendix 7

Individual Body Weight Gains (g)

2954-001
Sex: Male Bodyweight Gain (Interval)

Group 4	Day(s) Relative to Start Date				
	49 → 56	56 → 63	63 → 70	70 → 77	77 → 91
4006	12	25	21	16	9
4007	17	15	20	12	11
4008	8	18	18	0	12
4009	26	17	10	15	16
4010	14	26	10	14	8
4011	-	-	-	-	-
4012	-	-	-	-	-
4113	-	-	-	-	-
4021	-	-	-	-	-
4022	-	-	-	-	-
4023	-	-	-	-	-
4024	-	-	-	-	-
4025	-	-	-	-	-
4026	-	-	-	-	-
4027	-	-	-	-	-
Mean	15.4	20.2	15.8	11.4	11.2
SD	6.8	5.0	5.4	6.5	3.1
N	5	5	5	5	5

Appendix 7

Individual Body Weight Gains (g)

2954-001
Sex: Female Bodyweight Gain (Interval)

Group 1	Day(s) Relative to Start Date						
	-1 → 7	7 → 14	14 → 21	21 → 28	28 → 35	35 → 42	42 → 49
1506	23	22	18	11	2	9	12
1507	29	18	13	20	12	6	0
1508	17	19	6	16	16	1	2
1509	30	23	24	20	18	10	12
1510	24	26	10	7	16	6	8
1511	25	-	-	-	-	-	-
1512	31	-	-	-	-	-	-
1513	23	-	-	-	-	-	-
1514	24	-	-	-	-	-	-
1515	27	-	-	-	-	-	-
1521	12	26	10	10	-	-	-
1522	28	21	23	2	-	-	-
1523	25	10	-39	61	-	-	-
1524	11	24	-32	45	-	-	-
1525	30	24	-6	18	-	-	-
Mean	23.9	21.3	2.7	21.0	12.8	6.4	6.8
SD	6.2	4.8	22.0	18.2	6.4	3.5	5.6
N	15	10	10	10	5	5	5

Appendix 7

Individual Body Weight Gains (g)

2954-001
Sex: Female Bodyweight Gain (Interval)

Group 1	Day(s) Relative to Start Date				
	49 → 56	56 → 63	63 → 70	70 → 77	77 → 91
1506	-3	4	-3	17	7
1507	9	11	-3	-2	13
1508	12	7	0	-14	18
1509	0	8	6	8	2
1510	0	12	11	1	5
1511	-	-	-	-	-
1512	-	-	-	-	-
1513	-	-	-	-	-
1514	-	-	-	-	-
1515	-	-	-	-	-
1521	-	-	-	-	-
1522	-	-	-	-	-
1523	-	-	-	-	-
1524	-	-	-	-	-
1525	-	-	-	-	-
Mean	3.6	8.4	2.2	2.0	9.0
SD	6.5	3.2	6.1	11.6	6.4
N	5	5	5	5	5

Appendix 7

Individual Body Weight Gains (g)

2954-001
Sex: Female Bodyweight Gain (Interval)

Group 2	Day(s) Relative to Start Date						
	-1 → 7	7 → 14	14 → 21	21 → 28	28 → 35	35 → 42	42 → 49
2506	32	38	18	22	21	2	15
2507	25	14	19	11	11	0	24
2508	35	13	30	17	16	1	20
2509	30	32	14	13	1	13	11
2510	38	18	30	5	24	1	14
2511	15	-	-	-	-	-	-
2512	21	-	-	-	-	-	-
2513	19	-	-	-	-	-	-
2514	16	-	-	-	-	-	-
2515	27	-	-	-	-	-	-
2521	23	16	17	4	-	-	-
2522	19	15	18	22	-	-	-
2523	24	9	-34	71	-	-	-
2524	18	24	-40	55	-	-	-
2525	17	13	-1	17	-	-	-
Mean	23.9	19.2	7.1	23.7	14.6	3.4	16.8
SD	7.2	9.3	24.8	21.9	9.1	5.4	5.2
N	15	10	10	10	5	5	5

Appendix 7

Individual Body Weight Gains (g)

2954-001
Sex: Female Bodyweight Gain (Interval)

Group 2	Day(s) Relative to Start Date				
	49 → 56	56 → 63	63 → 70	70 → 77	77 → 91
2506	12	4	18	-3	17
2507	10	11	0	15	12
2508	-5	16	6	-5	7
2509	6	1	14	6	-1
2510	11	4	9	2	12
2511	-	-	-	-	-
2512	-	-	-	-	-
2513	-	-	-	-	-
2514	-	-	-	-	-
2515	-	-	-	-	-
2521	-	-	-	-	-
2522	-	-	-	-	-
2523	-	-	-	-	-
2524	-	-	-	-	-
2525	-	-	-	-	-
Mean	6.8	7.2	9.4	3.0	9.4
SD	7.0	6.1	7.0	8.0	6.8
N	5	5	5	5	5

Appendix 7

Individual Body Weight Gains (g)

2954-001
Sex: Female Bodyweight Gain (Interval)

Group 3	Day(s) Relative to Start Date						
	-1 → 7	7 → 14	14 → 21	21 → 28	28 → 35	35 → 42	42 → 49
3506	33	33	20	20	17	12	18
3507	25	28	16	13	2	18	9
3508	37	15	21	7	9	6	15
3509	32	14	13	3	17	9	15
3510	30	18	7	12	19	3	6
3511	19	-	-	-	-	-	-
3512	15	-	-	-	-	-	-
3513	15	-	-	-	-	-	-
3514	24	-	-	-	-	-	-
3515	17	-	-	-	-	-	-
3521	19	2	15	16	-	-	-
3522	17	12	9	1	-	-	-
3523	24	14	11	-12	-	-	-
3524	12	9	12	11	-	-	-
3525	20	-1	1	12	-	-	-
Mean	22.6	14.4	12.5	8.3	12.8	9.6	12.6
SD	7.5	10.4	6.0	9.1	7.2	5.8	4.9
N	15	10	10	10	5	5	5

Appendix 7

Individual Body Weight Gains (g)

2954-001
Sex: Female Bodyweight Gain (Interval)

Group 3	Day(s) Relative to Start Date				
	49 → 56	56 → 63	63 → 70	70 → 77	77 → 91
3506	11	8	10	6	20
3507	-2	5	14	8	-4
3508	2	5	2	13	7
3509	-1	15	8	0	-3
3510	6	4	9	3	3
3511	-	-	-	-	-
3512	-	-	-	-	-
3513	-	-	-	-	-
3514	-	-	-	-	-
3515	-	-	-	-	-
3521	-	-	-	-	-
3522	-	-	-	-	-
3523	-	-	-	-	-
3524	-	-	-	-	-
3525	-	-	-	-	-
Mean	3.2	7.4	8.6	6.0	4.6
SD	5.4	4.5	4.3	4.9	9.7
N	5	5	5	5	5

Appendix 7

Individual Body Weight Gains (g)

2954-001
Sex: Female Bodyweight Gain (Interval)

Group 4	Day(s) Relative to Start Date						
	-1 → 7	7 → 14	14 → 21	21 → 28	28 → 35	35 → 42	42 → 49
4506	33	17	22	10	23	14	8
4507	29	18	15	9	17	9	12
4508	25	24	1	25	17	7	7
4509	36	24	14	4	22	14	13
4510	35	27	18	9	17	13	10
4511	26	-	-	-	-	-	-
4512	16	-	-	-	-	-	-
4513	21	-	-	-	-	-	-
4514	13	-	-	-	-	-	-
4515	24	-	-	-	-	-	-
4521	24	13	-35	45	-	-	-
4522	32	24	-44	57	-	-	-
4523	9	22	9	8	-	-	-
4524	10	18	10	9	-	-	-
4525	11	9	11	2	-	-	-
Mean	22.9	19.6	2.1	17.8	19.2	11.4	10.0
SD	9.3	5.6	22.7	18.7	3.0	3.2	2.5
N	15	10	10	10	5	5	5

Appendix 7

Individual Body Weight Gains (g)

2954-001
Sex: Female   Bodyweight Gain (Interval)

Group 4	Day(s) Relative to Start Date				
	49 → 56	56 → 63	63 → 70	70 → 77	77 → 91
4506	-4	16	0	7	-5
4507	-1	13	1	5	-1
4508	10	11	1	0	9
4509	-2	12	9	0	-7
4510	7	9	15	0	-10
4511	-	-	-	-	-
4512	-	-	-	-	-
4513	-	-	-	-	-
4514	-	-	-	-	-
4515	-	-	-	-	-
4521	-	-	-	-	-
4522	-	-	-	-	-
4523	-	-	-	-	-
4524	-	-	-	-	-
4525	-	-	-	-	-
Mean	2.0	12.2	5.2	2.4	-2.8
SD	6.1	2.6	6.6	3.4	7.4
N	5	5	5	5	5

Appendix 7

Individual Body Weight Gains (g)

2954-001

Key Page

Measurement Descriptions

Headings Used	Description
Bodyweight Gain (Interval)	Bodyweight Gain (Interval)

Measurement/Statistics

Measurement	Descriptive
Bodyweight Gain (Interval)	Mean
	Standard Deviation
	Count

Group Information

Short Name	Long Name	Type	Report Headings 1-4
1	Group 1	Control	Group 1
2	Group 2	Dose	Group 2
3	Group 3	Dose	Group 3
4	Group 4	Dose	Group 4

Appendix 8

Individual Food Consumption

2954-001

Sex: Male Daily Food Cons Per Animal (g)

Group 1	No. in Cage	Day(s) Relative to Animal Start Date						
		1 → 7	1 → 8	8 → 15	15 → 22	27 → 28	22 → 28	22 → 29
3	3	-	28	30	31	-	-	30
4	2	-	28	28	27	-	-	32
5	3	25	-	-	-	-	-	-
6	2	27	-	-	-	-	-	-
1021	2	-	25	26	24	-	27	-
1023	3	-	26	26	26	-	27	-
1025	1	-	-	-	-	19	-	-
	Mean	25.8	26.6	27.5	26.8	19.0	27.3	31.3
	SD	1.7	1.5	1.7	2.7	-	0.0	1.4
	N	2	4	4	4	1	2	2

Appendix 8

Individual Food Consumption

2954-001
Sex: Male Daily Food Cons Per Animal (g)

Group 1	No. in Cage	Day(s) Relative to Animal Start Date						
		29 → 36	36 → 43	43 → 50	50 → 57	57 → 64	64 → 71	71 → 78
3	3	32	31	30	32	32	32	33
4	2	31	31	31	33	34	32	30
5	3	-	-	-	-	-	-	-
6	2	-	-	-	-	-	-	-
1021	2	-	-	-	-	-	-	-
1023	3	-	-	-	-	-	-	-
1025	1	-	-	-	-	-	-	-
	Mean	31.3	31.3	30.4	32.6	33.0	31.9	31.3
	SD	0.5	0.2	0.2	0.5	1.2	0.2	1.8
	N	2	2	2	2	2	2	2

Appendix 8

Individual Food Consumption

2954-001
Sex: Male Daily Food Cons Per Animal (g)

Group 1	No. in Cage	Day(s) Relative to Animal Start Date		
		78 → 85	85 → 86	85 → 90
3	3	31	29	-
4	2	29	-	27
5	3	-	-	-
6	2	-	-	-
1021	2	-	-	-
1023	3	-	-	-
1025	1	-	-	-
	Mean	29.8	29.0	26.6
	SD	1.2	-	-
	N	2	1	1

Appendix 8

Individual Food Consumption

2954-001

Sex: Male Daily Food Cons Per Animal (g)

Group 2	No. in Cage	Day(s) Relative to Animal Start Date						
		1 → 7	1 → 8	8 → 15	15 → 22	27 → 28	22 → 28	22 → 29
15	3	-	28	29	31	-	-	31
16	2	-	29	29	29	-	-	29
17	3	27	-	-	-	-	-	-
18	2	26	-	-	-	-	-	-
2021	2	-	24	25	22	-	28	-
2023	3	-	25	27	28	-	28	-
2025	1	-	-	-	-	28	-	-
	Mean	26.5	26.4	27.2	27.6	28.0	27.8	30.2
	SD	0.3	2.1	1.8	3.9	-	0.4	1.2
	N	2	4	4	4	1	2	2

Appendix 8

Individual Food Consumption

2954-001

Sex: Male Daily Food Cons Per Animal (g)

Group 2	No. in Cage	Day(s) Relative to Animal Start Date						
		29 → 36	36 → 43	43 → 50	50 → 57	57 → 64	64 → 71	71 → 78
15	3	32	30	30	32	32	32	31
16	2	30	29	28	30	30	30	31
17	3	-	-	-	-	-	-	-
18	2	-	-	-	-	-	-	-
2021	2	-	-	-	-	-	-	-
2023	3	-	-	-	-	-	-	-
2025	1	-	-	-	-	-	-	-
	Mean	30.9	29.7	29.2	30.8	31.2	31.0	30.6
	SD	1.9	0.9	1.6	1.5	1.5	1.7	0.1
	N	2	2	2	2	2	2	2

Appendix 8

Individual Food Consumption

2954-001

Sex: Male Daily Food Cons Per Animal (g)

Group 2	No. in Cage	Day(s) Relative to Animal Start Date 78 → 85	85 → 86	85 → 90
15	3	30	31	-
16	2	29	-	27
17	3	-	-	-
18	2	-	-	-
2021	2	-	-	-
2023	3	-	-	-
2025	1	-	-	-
	Mean	29.7	30.7	26.9
	SD	0.6	-	-
	N	2	1	1

Appendix 8

Individual Food Consumption

2954-001

Sex: Male Daily Food Cons Per Animal (g)

Group 3	No. in Cage	Day(s) Relative to Animal Start Date 1 → 7	1 → 8	8 → 15	15 → 22	27 → 28	22 → 28	22 → 29
27	3	-	25	23	28	-	-	30 n=2
28	2	-	27	28	28	-	-	30
29	3	26	-	-	-	-	-	-
30	2	26	-	-	-	-	-	-
3021	2	-	24	26	24	-	26	-
3023	3	-	26	27	28	-	29	-
3025	1	-	-	-	-	25	-	-
Mean		25.8	25.3	25.9	26.6	25.0	27.2	29.7
SD		0.4	1.3	2.3	2.0	-	2.2	0.3
N		2	4	4	4	1	2	2

n = Number of Animals in Cage

Appendix 8

Individual Food Consumption

2954-001
Sex: Male Daily Food Cons Per Animal (g)

Group 3	No. in Cage	Day(s) Relative to Animal Start Date						
		29 → 36	36 → 43	43 → 50	50 → 57	57 → 64	64 → 71	71 → 78
27	3	33 n=2	32 n=2	31 n=2	32 n=2	33 n=2	32 n=2	33 n=2
28	2	30	29	29	29	29	29	28
29	3	-	-	-	-	-	-	-
30	2	-	-	-	-	-	-	-
3021	2	-	-	-	-	-	-	-
3023	3	-	-	-	-	-	-	-
3025	1	-	-	-	-	-	-	-
	Mean	31.2	30.3	29.9	30.9	30.7	30.6	30.6
	SD	1.8	2.2	1.9	2.0	3.1	2.2	3.5
	N	2	2	2	2	2	2	2

n = Number of Animals in Cage

Appendix 8

Individual Food Consumption

2954-001
Sex: Male Daily Food Cons Per Animal (g)

Group 3	No. in Cage	Day(s) Relative to Animal Start Date		
		78 → 85	85 → 86	85 → 90
27	3	32 n=2	37 n=2	-
28	2	27	-	30
29	3	-	-	-
30	2	-	-	-
3021	2	-	-	-
3023	3	-	-	-
3025	1	-	-	-
	Mean	29.7	36.5	29.8
	SD	3.6	-	-
	N	2	1	1

n = Number of Animals in Cage

Appendix 8

Individual Food Consumption

2954-001
Sex: Male Daily Food Cons Per Animal (g)

Group 4	No. in Cage	Day(s) Relative to Animal Start Date						
		1 → 7	1 → 8	8 → 15	15 → 22	22 → 28	22 → 29	29 → 36
39	3	-	26	27	28	-	27	28
40	2	-	25	26	26	-	27	29
41	3	25	-	-	-	-	-	-
4023	2	-	26	28	28	28	-	-
4026	3	-	22	26	26	28	-	-
4113	3	23	-	-	-	-	-	-
	Mean	24.1	24.7	26.8	27.0	28.1	27.0	28.4
	SD	1.1	1.8	0.9	0.7	0.0	0.2	1.2
	N	2	4	4	4	2	2	2

Appendix 8

Individual Food Consumption

2954-001
Sex: Male Daily Food Cons Per Animal (g)

Group 4	No. in Cage	Day(s) Relative to Animal Start Date						
		36 → 43	43 → 50	50 → 57	57 → 64	64 → 71	71 → 78	78 → 85
39	3	28	27	28	28	28	26	27
40	2	28	27	28	27	27	27	27
41	3	-	-	-	-	-	-	-
4023	2	-	-	-	-	-	-	-
4026	3	-	-	-	-	-	-	-
4113	3	-	-	-	-	-	-	-
Mean		27.6	27.3	27.8	27.7	27.3	26.6	27.0
SD		0.1	0.1	0.4	0.9	0.4	0.3	0.0
N		2	2	2	2	2	2	2

Appendix 8

Individual Food Consumption

2954-001
Sex: Male Daily Food Cons Per Animal (g)

Group 4	No. in Cage	Day(s) Relative to Animal Start Date	
		85 → 86	85 → 90
39	3	29	-
40	2	-	27
41	3	-	-
4023	2	-	-
4026	3	-	-
4113	3	-	-
	Mean	28.7	27.0
	SD	-	-
	N	1	1

Appendix 8

Individual Food Consumption

2954-001
Sex: Female Daily Food Cons Per Animal (g)

Group 1	No. in Cage	Day(s) Relative to Animal Start Date						
		1 → 7	1 → 8	8 → 15	15 → 22	27 → 28	22 → 28	22 → 29
9	3	-	18	18	18	-	-	19
10	2	-	19	19	20	-	-	20
11	3	19	-	-	-	-	-	-
12	2	21	-	-	-	-	-	-
1521	2	-	19	19	21	-	19	-
1523	3	-	18	19	13	-	17	-
1525	1	-	-	-	-	24	-	-
	Mean	20.2	18.7	18.9	17.9	24.0	17.9	19.4
	SD	1.5	0.6	0.5	3.3	-	2.0	0.9
	N	2	4	4	4	1	2	2

Appendix 8

Individual Food Consumption

2954-001
Sex: Female Daily Food Cons Per Animal (g)

Group 1	No. in Cage	Day(s) Relative to Animal Start Date						
		29 → 36	36 → 43	43 → 50	50 → 57	57 → 64	64 → 71	71 → 78
9	3	19	5	18	19	20	18	17
10	2	21	6	19	19	19	19	20
11	3	-	-	-	-	-	-	-
12	2	-	-	-	-	-	-	-
1521	2	-	-	-	-	-	-	-
1523	3	-	-	-	-	-	-	-
1525	1	-	-	-	-	-	-	-
	Mean	20.0	5.6	18.3	18.9	19.2	18.6	18.6
	SD	1.0	0.2	0.8	0.0	0.4	0.7	1.8
	N	2	2	2	2	2	2	2

Appendix 8

Individual Food Consumption

2954-001
Sex: Female Daily Food Cons Per Animal (g)

Group 1	No. in Cage	Day(s) Relative to Animal Start Date		
		78 → 85	85 → 89	85 → 90
9	3	18	20	-
10	2	19	-	20
11	3	-	-	-
12	2	-	-	-
1521	2	-	-	-
1523	3	-	-	-
1525	1	-	-	-
	Mean	18.3	19.8	20.3
	SD	0.4	-	-
	N	2	1	1

Appendix 8

Individual Food Consumption

2954-001
Sex: Female Daily Food Cons Per Animal (g)

Group 2	No. in Cage	Day(s) Relative to Animal Start Date						
		1 → 7	1 → 8	8 → 15	15 → 22	27 → 28	22 → 28	22 → 29
21	3	-	20	19	19	-	-	21
22	2	-	22	23	24	-	-	22
23	3	18	-	-	-	-	-	-
24	2	20	-	-	-	-	-	-
2521	2	-	17	17	18	-	17	-
2523	3	-	19	17	13	-	21	-
2525	1	-	-	-	-	20	-	-
	Mean	18.7	19.3	19.0	18.5	20.0	19.4	21.4
	SD	1.1	2.0	2.6	4.4	-	2.8	0.8
	N	2	4	4	4	1	2	2

Appendix 8

Individual Food Consumption

2954-001

Sex: Female Daily Food Cons Per Animal (g)

Group 2	No. in Cage	Day(s) Relative to Animal Start Date						
		29 → 36	36 → 43	43 → 50	50 → 57	57 → 64	64 → 71	71 → 78
21	3	21	3	20	21	21	21	21
22	2	23	4	22	22	22	22	23
23	3	-	-	-	-	-	-	-
24	2	-	-	-	-	-	-	-
2521	2	-	-	-	-	-	-	-
2523	3	-	-	-	-	-	-	-
2525	1	-	-	-	-	-	-	-
	Mean	21.9	3.5	20.8	21.8	21.3	21.4	21.6
	SD	1.6	1.2	1.1	0.6	0.7	1.3	1.5
	N	2	2	2	2	2	2	2

Appendix 8

Individual Food Consumption

2954-001
Sex: Female Daily Food Cons Per Animal (g)

Group 2	No. in Cage	Day(s) Relative to Animal Start Date	
		78 → 85	85 → 90
21	3	20	20
22	2	21	22
23	3	-	-
24	2	-	-
2521	2	-	-
2523	3	-	-
2525	1	-	-
	Mean	20.7	21.0
	SD	0.7	1.9
	N	2	2

Appendix 8

Individual Food Consumption

2954-001
Sex: Female Daily Food Cons Per Animal (g)

Group 3	No. in Cage	Day(s) Relative to Animal Start Date						
		1 → 7	1 → 8	8 → 15	15 → 22	27 → 28	22 → 28	22 → 29
33	3	-	21	19	20	-	-	20
34	2	-	21	19	21	-	-	20
35	2	18	-	-	-	-	-	-
36	3	18	-	-	-	-	-	-
3521	2	-	15	16	16	-	17	-
3523	3	-	18	18	6	-	7	-
3525	1	-	-	-	-	16	-	-
	Mean	18.1	18.6	18.2	15.6	16.0	12.1	20.3
	SD	0.5	2.7	1.3	6.9	-	7.1	0.1
	N	2	4	4	4	1	2	2

Appendix 8

Individual Food Consumption

2954-001
Sex: Female Daily Food Cons Per Animal (g)

Group 3	No. in Cage	Day(s) Relative to Animal Start Date						
		29 → 36	36 → 43	43 → 50	50 → 57	57 → 64	64 → 71	71 → 78
33	3	21	12	20	21	20	20	21
34	2	21	6	20	21	20	21	21
35	2	-	-	-	-	-	-	-
36	3	-	-	-	-	-	-	-
3521	2	-	-	-	-	-	-	-
3523	3	-	-	-	-	-	-	-
3525	1	-	-	-	-	-	-	-
	Mean	21.1	8.7	20.3	20.8	20.0	20.4	20.9
	SD	0.3	4.5	0.2	0.5	0.3	0.4	0.4
	N	2	2	2	2	2	2	2

Appendix 8

Individual Food Consumption

2954-001
Sex: Female Daily Food Cons Per Animal (g)

Group 3	No. in Cage	Day(s) Relative to Animal Start Date	
		78 → 85	85 → 90
33	3	20	21
34	2	19	20
35	2	-	-
36	3	-	-
3521	2	-	-
3523	3	-	-
3525	1	-	-
	Mean	19.5	20.6
	SD	0.5	0.4
	N	2	2

Appendix 8

Individual Food Consumption

2954-001
Sex: Female Daily Food Cons Per Animal (g)

Group 4	No. in Cage	Day(s) Relative to Animal Start Date						
		1 → 7	1 → 8	8 → 15	15 → 22	22 → 28	22 → 29	29 → 36
45	3	-	18	18	19	-	19	21
46	2	-	20	21	21	-	20	22
47	2	27	-	-	-	-	-	-
48	3	13	-	-	-	-	-	-
4521	2	-	19	19	14	20	-	-
4523	3	-	17	17	17	17	-	-
Mean		20.1	18.4	18.8	17.5	18.8	19.8	21.8
SD		9.7	1.4	1.7	3.3	2.1	0.9	0.9
N		2	4	4	4	2	2	2

Appendix 8

Individual Food Consumption

2954-001
Sex: Female Daily Food Cons Per Animal (g)

Group 4	No. in Cage	Day(s) Relative to Animal Start Date						
		36 → 43	43 → 50	50 → 57	57 → 64	64 → 71	71 → 78	78 → 85
45	3	2	19	19	19	19	18	18
46	2	-7 E a	20	22	21	22	21	18
47	2	-	-	-	-	-	-	-
48	3	-	-	-	-	-	-	-
4521	2	-	-	-	-	-	-	-
4523	3	-	-	-	-	-	-	-
Mean		2.0	19.4	20.9	20.2	20.3	19.9	18.1
SD		-	1.0	2.0	1.5	2.4	2.0	0.3
N		1	2	2	2	2	2	2

E = Exclude
[a] [FC:Value excluded due to negative food consumption value indicating that the feeder was filled and not weighed at some point.]

Appendix 8

Individual Food Consumption

2954-001
Sex: Female Daily Food Cons Per Animal (g)

Group 4	No. in Cage	Day(s) Relative to Animal Start Date
		85 → 90
45	3	21
46	2	19
47	2	-
48	3	-
4521	2	-
4523	3	-
	Mean	19.9
	SD	0.9
	N	2

Appendix 8

Individual Food Consumption

2954-001

Comments and Markers

Page	Measurement	Group	Sex	Cage	Day	Type	Marker
	Daily Food Cons Per Animal	4	Female	46	36 - 43	Quality Flag	E (Exclude)

Comment: Value excluded due to negative food consumption value indicating that the feeder was filled and not weighed at some point.

Appendix 8

Individual Food Consumption

2954-001

Key Page

Cage Contents

Cage Number	Animal Numbers	Cage Number	Animal Numbers
3	1006, 1007, 1008	4	1009, 1010
5	1011, 1012, 1013	6	1014, 1015
9	1506, 1507, 1508	10	1509, 1510
11	1511, 1512, 1513	12	1514, 1515
15	2006, 2007, 2008	16	2009, 2010
17	2011, 2012, 2013	18	2014, 2015
21	2506, 2507, 2508	22	2509, 2510
23	2511, 2512, 2513	24	2514, 2515
27	3006, 3007, [3008]	28	3009, 3010
29	3011, 3012, 3013	30	3014, 3015
33	3506, 3507, 3508	34	3509, 3510
35	3511, 3512	36	3513, 3514, 3515
39	4006, 4007, 4008	40	4009, 4010
41	4011, 4012, 4013	45	4506, 4507, 4508
46	4509, 4510	47	4511, 4512
48	4513, 4514, 4515	1,021	1021, 1022
1,023	1023, 1024, [1025]	1,025	1025
1,521	1521, 1522	1,523	1523, 1524, [1525]
1,525	1525	2,021	2021, 2022
2,023	2023, 2024, [2025]	2,025	2025
2,521	2521, 2522	2,523	2523, 2524, [2525]
2,525	2525	3,021	3021, 3022
3,023	3023, 3024, [3025]	3,025	3025
3,521	3521, 3522	3,523	3523, 3524, [3525]
3,525	3525	4,023	4023, 4024
4,026	4025, 4026, 4027	4,113	4113, 4021, 4022
4,521	4521, 4522	4,523	4523, 4524, 4525

Appendix 8

Individual Food Consumption

2954-001

Key Page

[] = Animal exited cage during results period;

Quality Flags

Symbol	IES Status	Description
E	Excluded	Exclude

Measurement Descriptions

Headings Used	Description
Daily Food Cons Per Animal	Mean Daily Food Cons. Per Animal

Measurement/Statistics

Measurement	Descriptive
Daily Food Cons Per Animal	Mean
	Standard Deviation
	Count

Group Information

Short Name	Long Name	Type	Report Headings 1-4
1	Group 1	Control	Group 1
2	Group 2	Dose	Group 2
3	Group 3	Dose	Group 3
4	Group 4	Dose	Group 4

Comment Abbreviations

FC = Flag Comment

Appendix 9

Final Report

Study Phase: Clinical Pathology

Testing Facility Study No. 2954-001

Sponsor Reference No. UTSW.Gray-002

GLP

TESTING FACILITY:
Charles River Laboratories, Inc.
54943 North Main Street
Mattawan, MI
49071, United States

Appendix 9

TABLE OF CONTENTS

LIST OF TEXT TABLES....3
LIST OF TABLES....4
LIST OF APPENDICES....5
REPORT APPROVAL....6
1. SUMMARY....7
2. MATERIALS AND METHODS....8
2.1. Group Assignments....8
2.2. Hematology, Coagulation, and Clinical Chemistry....8
2.3. Computerized Systems....9
3. RESULTS AND DISCUSSION....10
3.1. Unscheduled Collections....10
3.2. Hematology....10
3.3. Coagulation....10
3.4. Clinical Chemistry....10

LIST OF TEXT TABLES

Text Table 1 Group Assignments 8
Text Table 2 Clinical Pathology Sample Collection 8
Text Table 3 Data Collection and Analysis Software 9

Appendix 9

LIST OF TABLES

Table 1 Summary of Hematology Values 11
Table 2 Summary of Coagulation Values 37
Table 3 Summary of Clinical Chemistry Values 44

Appendix 9

LIST OF APPENDICES

Appendix 1 Individual Hematology Values....74
Appendix 2 Individual Coagulation Values....109
Appendix 3 Individual Clinical Chemistry Values....118

Appendix 9

REPORT APPROVAL

All electronic signatures appear at the end of the document upon finalization.

Appendix 9

1. SUMMARY

This study was conducted for University of Texas Southwestern Medical Center to characterize the toxicity, biodistribution, and gene expression of the test material, AAV9/SURF1, for the treatment of SURF1 Leigh Syndrome when given to CD® [Crl:CD®(SD)] rats via intrathecal (IT) injection once on Day 1. Clinical pathology evaluations were conducted on main study animals as detailed below (see Text Table 2).

Minimally higher WBC attributed by higher lymphocytes on Day 29 in females at 2.49E12 (Group 4). This finding lacked a microscopic correlate.

2. MATERIALS AND METHODS

2.1. Group Assignments

Animals were assigned to the study as indicated in Text Table 1.

Text Table 1
Group Assignments

Group No.	Dose Level (vg)	No. of Animals					
		Day 8 Necropsy		Day 29 (±1) Necropsy		Day 91 (±4) Necropsy	
		M	F	M	F	M	F
1	0	5	5	5	5	5	5
2	0.28E12	5	5	5	5	5	5
3	0.83E12	6	5	5	5	5	5
4	2.49E12	5	5	5	5	5	5

vg = vector genome.

M - Male

F - Female

2.2. Hematology, Coagulation, and Clinical Chemistry

Samples were collected according to Text Table 2 and analyzed for protocol designated endpoints.

Text Table 2
Clinical Pathology Sample Collection

Group No.[a]	Time Point(s)	Hematology	Coagulation	Clinical Chemistry
All surviving animals	Terminal	X	X	X
Unscheduled Euthanasia	When possible, samples were collected from animals with an unscheduled euthanasia.			
Target Volume (mL)[b]:	NA	1 mL	1.2 mL	1.3 mL
Collection Site:	Cardiac puncture or vena cava after carbon dioxide inhalation			
Fasting Required[c]:	The animals had access to drinking water but were fasted overnight (at least 8 hours) prior to scheduled blood collection.			
Anticoagulant:	NA	K_2EDTA	Sodium Citrate	Serum Gel Separator
Processing:	NA	None	Plasma	Serum

X = Sample was collected; NA = Not applicable.

[a] Designated animals were bled at each time point with the exception of collections impacted by unscheduled deaths.

[b] Additional blood samples were obtained due to sample quality or volume as permissible. Suitable methods were used for unscheduled collections and/or redraws.

[c] Three Group 1 females (Animal No. 1506, 1507, and 1508) and three Group 2 females (Animal No. 2506, 2507, and 2508) were not fasted overnight prior to the Day 90 necropsy blood collection.

2.3. Computerized Systems

Critical computerized systems used in the study phase are listed below (Text Table 3). All computerized systems used in the conduct of this study have been validated; when a particular system has not satisfied all requirements, appropriate administrative and procedural controls were implemented to assure the quality and integrity of data.

Text Table 3
Data Collection and Analysis Software

Hematology	Advia 2120i v6.9
Coagulation	STA Compact Max v103.04 or STA-R Evolution v3.04
Clinical Chemistry	AU5800 v05.03

3. RESULTS AND DISCUSSION

For the purpose of this report, treated animals' values were compared control values.

3.1. Unscheduled Collections

Due to poor clinical condition, Animal No. 3008 (0.83E12 vg, Male) had a collection for clinical pathology and subsequently euthanized on Day 16. There was a mildly lower WBC attributed by lower neutrophils, lymphocytes and monocytes compared to control and cohort animals. There was mildly higher total red cell mass (RBC, Hb, HCT) with moderately lower decreased reticulocytes. These changes suggest a diminished hematopoiesis with evidence of subclinical dehydration. Changes in clinical chemistry included minimally higher alanine aminotransferase (ALT), urea nitrogen, total protein, albumin and globulin. All indicate the presence of subclinical dehydration. All changes are considered not directly related to test material administration.

3.2. Hematology

(Table 1 and Appendix 1)

On Day 29, females at 2.49E12 vg (Group 4) had minimally higher WBC attributed by higher lymphocytes.

All other fluctuations among individual and mean values were considered sporadic, consistent with biologic variation and/or negligible in magnitude, and not related to test material administration.

3.3. Coagulation

(Table 2 and Appendix 2)

There were no AAV9/SURF1-related effects among coagulation parameters in either sex at any dose level. All fluctuations among individual and mean values, regardless of statistical significance, were considered sporadic, consistent with biologic variation and/or negligible in magnitude, and not related to test material administration.

3.4. Clinical Chemistry

(Table 3 and Appendix 3)

There were no AAV9/SURF1-related effects among chemistry parameters in either sex at any dose level. All fluctuations among individual and mean values were considered sporadic, consistent with biologic variation and/or negligible in magnitude, and not related to test material administration.

Appendix 9

Table 1

Abbreviations for Hematology Parameters

WBC	-	White Blood Cell Count
NEUT	-	Neutrophils
LYMPH	-	Lymphocytes
MONO	-	Monocytes
EOS	-	Eosinophils
BASO	-	Basophils
LUC	-	Large Unstained Cells
RBC	-	Red Blood Cell Count
HGB	-	Hemoglobin
HCT	-	Hematocrit
MCV	-	Mean Corpuscular Volume
MCH	-	Mean Corpuscular Hemoglobin
MCHC	-	Mean Corpuscular Hemoglobin Concentration
RDW	-	Red Blood Cell Distribution Width
PLT	-	Platelet Count
RETIC	-	Reticulocytes
UNSC	-	Unscheduled bleed

Appendix 9

Table 1

Summary of Hematology Values

2954-001

Sex: Male Day(s) Relative to Start Date			Group 1	Group 2	Group 3	Group 4
WBC (10^3/uL)	8 [G]	Mean	11.224	12.602	13.398	11.988
		SD	3.591	3.490	4.458	4.731
		N	5	5	6	5
		tCtrl	-	1.12	1.19	1.07
	16 (Unsc) [I]	Mean	-	-	5.180 n	-
		SD	-	-	-	-
		N	-	-	1	-
	29 [G]	Mean	14.858	12.886	17.812	16.582
		SD	4.651	7.500	3.819	2.969
		N	5	5	5	5
		tCtrl	-	0.87	1.20	1.12
	90 [G]	Mean	10.846	15.398	16.655	13.818
		SD	7.046	3.271	2.819	4.425
		N	5	5	4	5
		tCtrl	-	1.42	1.54	1.27
NEUT (10^3/uL)	8 [G]	Mean	1.062	1.294	1.257	1.400
		SD	0.248	0.568	0.463	0.250
		N	5	5	6	5
		tCtrl	-	1.22	1.18	1.32

[G] - Anova & Dunnett
[I] - n - Inappropriate for statistics
Day 90 represents all terminal collections done on day 90 (±4)

Appendix 9

Table 1

Summary of Hematology Values

2954-001

Sex: Male		Day(s) Relative to Start Date	Group 1	Group 2	Group 3	Group 4
NEUT (10^3/uL)	16 (Unsc) [I]	Mean	-	-	0.300 n	-
		SD	-	-	-	-
		N	-	-	1	-
	29 [G]	Mean	1.474	1.280	1.840	1.160
		SD	0.612	0.504	0.530	0.177
		N	5	5	5	5
		tCtrl	-	0.87	1.25	0.79
	90 [G]	Mean	1.142	2.480 **	1.723	1.540
		SD	0.636	0.709	0.497	0.497
		N	5	5	4	5
		tCtrl	-	2.17	1.51	1.35
LYMPH (10^3/uL)	8 [G]	Mean	9.612	10.602	11.512	9.802
		SD	3.271	2.824	3.892	4.214
		N	5	5	6	5
		tCtrl	-	1.10	1.20	1.02
	16 (Unsc) [I]	Mean	-	-	4.620 n	-
		SD	-	-	-	-
		N	-	-	1	-

[G] - Anova & Dunnett: ** = p ≤ 0.01
[I] - n - Inappropriate for statistics
Day 90 represents all terminal collections done on day 90 (±4)

Appendix 9

Table 1

Summary of Hematology Values

2954-001

Sex: Male	Day(s) Relative to Start Date		Group 1	Group 2	Group 3	Group 4
LYMPH (10^3/uL)	29 [G]	Mean	12.876	10.966	15.114	14.544
		SD	4.084	6.681	3.215	2.757
		N	5	5	5	5
		tCtrl	-	0.85	1.17	1.13
	90 [G]	Mean	9.270	12.170	14.215	11.506
		SD	6.125	3.487	2.336	3.666
		N	5	5	4	5
		tCtrl	-	1.31	1.53	1.24
MONO (10^3/uL)	8 [G]	Mean	0.262	0.346	0.282	0.418
		SD	0.066	0.151	0.143	0.306
		N	5	5	6	5
		tCtrl	-	1.32	1.08	1.60
	16 (Unsc) [I]	Mean	-	-	0.060 n	-
		SD	-	-	-	-
		N	-	-	1	-
	29 [G1]	Mean	0.194	0.204	0.356	0.344
		SD	0.045	0.125	0.176	0.153
		N	5	5	5	5
		tCtrl	-	1.05	1.84	1.77

[G] - Anova & Dunnett
[G1] - Kruskal-Wallis & Dunn
[I] - n - Inappropriate for statistics
Day 90 represents all terminal collections done on day 90 (±4)

Appendix 9

Table 1

Summary of Hematology Values

2954-001

Sex: Male	Day(s) Relative to Start Date		Group 1	Group 2	Group 3	Group 4
MONO (10^3/uL)	90 [G]	Mean	0.200	0.332	0.360	0.368
		SD	0.162	0.126	0.143	0.148
		N	5	5	4	5
		tCtrl	-	1.66	1.80	1.84
EOS (10^3/uL)	8 [G]	Mean	0.072	0.060	0.052	0.076
		SD	0.043	0.037	0.023	0.026
		N	5	5	6	5
		tCtrl	-	0.83	0.72	1.06
	16 (Unsc) [I]	Mean	-	-	0.080 n	-
		SD	-	-	-	-
		N	-	-	1	-
	29 [G]	Mean	0.062	0.092	0.086	0.124
		SD	0.019	0.044	0.034	0.073
		N	5	5	5	5
		tCtrl	-	1.48	1.39	2.00
	90 [G]	Mean	0.084	0.128	0.070	0.112
		SD	0.084	0.043	0.029	0.068
		N	5	5	4	5
		tCtrl	-	1.52	0.83	1.33

[G] - Anova & Dunnett
[I] - n - Inappropriate for statistics
Day 90 represents all terminal collections done on day 90 (±4)

Appendix 9

Table 1

Summary of Hematology Values

2954-001

Sex: Male		Day(s) Relative to Start Date	Group 1	Group 2	Group 3	Group 4
BASO (10^3/uL)	8 [G]	Mean	0.068	0.082	0.090	0.130
		SD	0.033	0.019	0.043	0.062
		N	5	5	6	5
		tCtrl	-	1.21	1.32	1.91
	16 (Unsc) [I]	Mean	-	-	0.040 n	-
		SD	-	-	-	-
		N	-	-	1	-
	29 [G]	Mean	0.110	0.102	0.148	0.162
		SD	0.047	0.079	0.045	0.044
		N	5	5	5	5
		tCtrl	-	0.93	1.35	1.47
	90 [G]	Mean	0.084	0.126	0.150	0.126
		SD	0.078	0.052	0.062	0.048
		N	5	5	4	5
		tCtrl	-	1.50	1.79	1.50
LUC (10^3/uL)	8 [G]	Mean	0.148	0.220	0.203	0.158
		SD	0.061	0.129	0.083	0.143
		N	5	5	6	5
		tCtrl	-	1.49	1.37	1.07

[G] - Anova & Dunnett
[I] - n - Inappropriate for statistics
Day 90 represents all terminal collections done on day 90 (±4)

Appendix 9
Table 1

Summary of Hematology Values

2954-001

Sex: Male			Group 1	Group 2	Group 3	Group 4
Day(s) Relative to Start Date						
LUC (10^3/uL)	16 (Unsc) [I]	Mean	-	-	0.080 n	-
		SD	-	-	-	-
		N	-	-	1	-
	29 [G]	Mean	0.140	0.240	0.266	0.250
		SD	0.057	0.198	0.077	0.165
		N	5	5	5	5
		tCtrl	-	1.71	1.90	1.79
	90 [G]	Mean	0.064	0.160	0.143	0.170
		SD	0.042	0.058	0.046	0.092
		N	5	5	4	5
		tCtrl	-	2.50	2.23	2.66
RBC (10^6/uL)	8 [G]	Mean	8.136	7.924	7.895	8.020
		SD	0.656	0.665	0.380	0.356
		N	5	5	6	5
		tCtrl	-	0.97	0.97	0.99
	16 (Unsc) [I]	Mean	-	-	9.040 n	-
		SD	-	-	-	-
		N	-	-	1	-

[G] - Anova & Dunnett
[I] - n - Inappropriate for statistics
Day 90 represents all terminal collections done on day 90 (±4)

Appendix 9

Table 1

Summary of Hematology Values

2954-001

Sex: Male	Day(s) Relative to Start Date		Group 1	Group 2	Group 3	Group 4
RBC (10^6/uL)	29 [G]	Mean	8.738	8.736	8.752	9.394
		SD	0.533	0.509	0.517	0.524
		N	5	5	5	5
		tCtrl	-	1.00	1.00	1.08
	90 [G]	Mean	9.072	9.210	9.295	9.480
		SD	0.378	0.209	0.265	0.476
		N	5	5	4	5
		tCtrl	-	1.02	1.02	1.04
HGB (g/dL)	8 [G]	Mean	16.60	16.00	16.17	16.16
		SD	0.61	1.21	0.46	0.88
		N	5	5	6	5
		tCtrl	-	0.96	0.97	0.97
	16 (Unsc) [I]	Mean	-	-	17.80 n	-
		SD	-	-	-	-
		N	-	-	1	-
	29 [G]	Mean	16.60	16.72	16.42	17.22
		SD	0.70	0.80	0.80	0.84
		N	5	5	5	5
		tCtrl	-	1.01	0.99	1.04

[G] - Anova & Dunnett
[I] - n - Inappropriate for statistics
Day 90 represents all terminal collections done on day 90 (±4)

Appendix 9

Table 1

Summary of Hematology Values

2954-001

Sex: Male	Day(s) Relative to Start Date		Group 1	Group 2	Group 3	Group 4
HGB (g/dL)	90 [G]	Mean	15.74	16.28	16.40	16.98
		SD	0.76	0.33	0.52	0.82
		N	5	5	4	5
		tCtrl	-	1.03	1.04	1.08
HCT (%)	8 [G]	Mean	55.04	53.30	53.68	56.44
		SD	3.26	4.05	1.70	5.09
		N	5	5	6	5
		tCtrl	-	0.97	0.98	1.03
	16 (Unsc) [I]	Mean	-	-	58.50 n	-
		SD	-	-	-	-
		N	-	-	1	-
	29 [G]	Mean	56.36	57.14	55.98	58.62
		SD	3.56	2.99	2.43	3.35
		N	5	5	5	5
		tCtrl	-	1.01	0.99	1.04
	90 [G]	Mean	52.12	53.62	53.88	55.52
		SD	3.68	1.55	2.13	3.12
		N	5	5	4	5
		tCtrl	-	1.03	1.03	1.07

[G] - Anova & Dunnett
[I] - n - Inappropriate for statistics
Day 90 represents all terminal collections done on day 90 (±4)

Appendix 9

Table 1

Summary of Hematology Values

2954-001

Sex: Male	Day(s) Relative to Start Date		Group 1	Group 2	Group 3	Group 4
MCV (fL)	8 [G]	Mean	67.76	67.30	68.05	70.28
		SD	1.46	2.36	3.31	3.53
		N	5	5	6	5
		tCtrl	-	0.99	1.00	1.04
	16 (Unsc) [I]	Mean	-	-	64.70 n	-
		SD	-	-	-	-
		N	-	-	1	-
	29 [G1]	Mean	64.48	65.44	64.00	62.40
		SD	0.30	2.50	1.66	1.73
		N	5	5	5	5
		tCtrl	-	1.01	0.99	0.97
	90 [G]	Mean	57.44	58.24	58.00	58.56
		SD	2.66	2.08	1.56	1.47
		N	5	5	4	5
		tCtrl	-	1.01	1.01	1.02
MCH (pg)	8 [G]	Mean	20.48	20.24	20.52	20.16
		SD	0.93	0.62	0.83	0.42
		N	5	5	6	5
		tCtrl	-	0.99	1.00	0.98

[G] - Anova & Dunnett
[G1] - Kruskal-Wallis & Dunn
[I] - n - Inappropriate for statistics
Day 90 represents all terminal collections done on day 90 (±4)

Appendix 9
Table 1

Summary of Hematology Values

2954-001

Sex: Male<br>Day(s) Relative to Start Date			Group 1	Group 2	Group 3	Group 4
MCH (pg)	16 (Unsc) [I]	Mean	-	-	19.60 n	-
		SD	-	-	-	-
		N	-	-	1	-
	29 [G]	Mean	19.02	19.14	18.78	18.32
		SD	0.62	0.71	0.37	0.59
		N	5	5	5	5
		tCtrl	-	1.01	0.99	0.96
	90 [G]	Mean	17.36	17.66	17.60	17.94
		SD	0.66	0.44	0.42	0.40
		N	5	5	4	5
		tCtrl	-	1.02	1.01	1.03
MCHC (g/dL)	8 [G1]	Mean	30.22	30.06	30.17	28.74
		SD	0.72	0.18	0.39	1.34
		N	5	5	6	5
		tCtrl	-	0.99	1.00	0.95
	16 (Unsc) [I]	Mean	-	-	30.30 n	-
		SD	-	-	-	-
		N	-	-	1	-

[G] - Anova & Dunnett
[I] - n - Inappropriate for statistics
[G1] - Kruskal-Wallis & Dunn
Day 90 represents all terminal collections done on day 90 (±4)

Appendix 9

Table 1

Summary of Hematology Values

2954-001

Sex: Male	Day(s) Relative to Start Date		Group 1	Group 2	Group 3	Group 4
MCHC (g/dL)	29 [G]	Mean	29.46	29.26	29.34	29.38
		SD	1.08	0.36	0.24	0.45
		N	5	5	5	5
		tCtrl	-	0.99	1.00	1.00
	90 [G1]	Mean	30.26	30.38	30.38	30.64
		SD	0.84	0.53	0.39	0.27
		N	5	5	4	5
		tCtrl	-	1.00	1.00	1.01
RDW (%)	8 [G1]	Mean	12.46	13.20	13.22	13.44 *
		SD	0.27	0.60	0.52	0.54
		N	5	5	6	5
		tCtrl	-	1.06	1.06	1.08
	16 (Unsc) [I]	Mean	-	-	12.10 n	-
		SD	-	-	-	-
		N	-	-	1	-
	29 [G1]	Mean	11.60	11.48	11.48	11.60
		SD	0.35	0.38	0.42	0.40
		N	5	5	5	5
		tCtrl	-	0.99	0.99	1.00

[G] - Kruskal-Wallis & Dunn
[G1] - Anova & Dunnett: * = p ≤ 0.05
[I] - n - Inappropriate for statistics
Day 90 represents all terminal collections done on day 90 (±4)

Appendix 9
Table 1

Summary of Hematology Values

2954-001

Sex: Male	Day(s) Relative to Start Date		Group 1	Group 2	Group 3	Group 4
RDW (%)	90 [G]	Mean	13.00	12.62	12.80	12.48
		SD	0.48	0.45	0.78	0.43
		N	5	5	4	5
		tCtrl	-	0.97	0.98	0.96
PLT (10^3/uL)	8 [G]	Mean	916.0	1142.0	1199.5	986.2
		SD	327.9	148.0	268.0	348.4
		N	5	5	6	5
		tCtrl	-	1.25	1.31	1.08
	16 (Unsc) [I]	Mean	-	-	921.0 n	-
		SD	-	-	-	-
		N	-	-	1	-
	29 [G]	Mean	949.2	872.8	967.0	872.4
		SD	96.6	176.8	82.8	121.2
		N	5	5	5	5
		tCtrl	-	0.92	1.02	0.92
	90 [G]	Mean	934.6	1026.2	1063.0	919.6
		SD	161.0	213.7	157.3	66.7
		N	5	5	4	5
		tCtrl	-	1.10	1.14	0.98

[G] - Anova & Dunnett
[I] - n - Inappropriate for statistics
Day 90 represents all terminal collections done on day 90 (±4)

Appendix 9
Table 1

Summary of Hematology Values

2954-001

Sex: Male		Day(s) Relative to Start Date	Group 1	Group 2	Group 3	Group 4
RETIC (10^9/L)	8 [G]	Mean	425.16	496.42	474.92	475.40
		SD	46.26	48.20	48.64	46.33
		N	5	5	6	5
		tCtrl	-	1.17	1.12	1.12
	16 (Unsc) [I]	Mean	-	-	100.90 n	-
		SD	-	-	-	-
		N	-	-	1	-
	29 [G]	Mean	221.62	250.78	258.68	243.80
		SD	23.11	29.45	34.48	33.22
		N	5	5	5	5
		tCtrl	-	1.13	1.17	1.10
	90 [G]	Mean	189.04	172.96	201.05	176.24
		SD	35.18	17.58	18.10	13.27
		N	5	5	4	5
		tCtrl	-	0.91	1.06	0.93

[G] - Anova & Dunnett
[I] - n - Inappropriate for statistics
Day 90 represents all terminal collections done on day 90 (±4)

Appendix 9

Table 1

Summary of Hematology Values

2954-001

Sex: Female		Day(s) Relative to Start Date	Group 1	Group 2	Group 3	Group 4
WBC (10^3/uL)	8 [G]	Mean	10.540	7.020	10.276	6.782
		SD	2.032	1.770	1.864	3.722
		N	5	4	5	5
		tCtrl	-	0.67	0.97	0.64
	29 [G]	Mean	6.098	11.552 *	10.195	16.142 **
		SD	2.335	1.919	3.428	3.119
		N	5	5	4	5
		tCtrl	-	1.89	1.67	2.65
	90 [G]	Mean	6.728	10.184	10.824	10.910
		SD	1.917	3.441	4.416	2.097
		N	5	5	5	4
		tCtrl	-	1.51	1.61	1.62
NEUT (10^3/uL)	8 [G]	Mean	0.602	0.565	0.532	0.410
		SD	0.238	0.275	0.177	0.239
		N	5	4	5	5
		tCtrl	-	0.94	0.88	0.68

[G] - Anova & Dunnett: * = $p \leq 0.05$; ** = $p \leq 0.01$
Day 90 represents all terminal collections done on day 90 (±4)

Appendix 9
Table 1

Summary of Hematology Values

2954-001

Sex: Female<br>Day(s) Relative to Start Date			Group 1	Group 2	Group 3	Group 4
NEUT (10^3/uL)	29 [G]	Mean	0.666	0.904	0.935	1.086
		SD	0.239	0.434	0.280	0.370
		N	5	5	4	5
		tCtrl	-	1.36	1.40	1.63
	90 [G]	Mean	0.856	1.068	1.222	1.348
		SD	0.446	0.485	0.817	0.549
		N	5	5	5	4
		tCtrl	-	1.25	1.43	1.57
LYMPH (10^3/uL)	8 [G]	Mean	9.476	6.088	9.160	5.958
		SD	1.811	1.431	1.736	3.280
		N	5	4	5	5
		tCtrl	-	0.64	0.97	0.63
	29 [G]	Mean	5.048	10.168 *	8.855	14.288 **
		SD	1.966	2.071	3.397	3.254
		N	5	5	4	5
		tCtrl	-	2.01	1.75	2.83

[G] - Anova & Dunnett: * = $p \leq 0.05$; ** = $p \leq 0.01$
Day 90 represents all terminal collections done on day 90 (±4)

Appendix 9

Table 1

Summary of Hematology Values

2954-001

Sex: Female	Day(s) Relative to Start Date		Group 1	Group 2	Group 3	Group 4
LYMPH (10^3/uL)	90 [G]	Mean	5.504	8.572	9.120	9.015
		SD	1.747	3.438	3.411	1.512
		N	5	5	5	4
		tCtrl	-	1.56	1.66	1.64
MONO (10^3/uL)	8 [G]	Mean	0.214	0.148	0.278	0.192
		SD	0.032	0.064	0.102	0.095
		N	5	4	5	5
		tCtrl	-	0.69	1.30	0.90
	29 [G]	Mean	0.120	0.146	0.125	0.208
		SD	0.081	0.046	0.025	0.033
		N	5	5	4	5
		tCtrl	-	1.22	1.04	1.73
	90 [G]	Mean	0.136	0.204	0.224	0.293
		SD	0.063	0.092	0.101	0.029
		N	5	5	5	4
		tCtrl	-	1.50	1.65	2.15

[G] - Anova & Dunnett
Day 90 represents all terminal collections done on day 90 (±4)

Appendix 9

Table 1

Summary of Hematology Values

2954-001

Sex: Female		Day(s) Relative to Start Date	Group 1	Group 2	Group 3	Group 4
EOS (10^3/uL)	8 [G]	Mean	0.042	0.063	0.096	0.026
		SD	0.004	0.022	0.045	0.015
		N	5	4	5	5
		tCtrl	-	1.49	2.29	0.62
	29 [G1]	Mean	0.082	0.058	0.068	0.094
		SD	0.045	0.036	0.038	0.059
		N	5	5	4	5
		tCtrl	-	0.71	0.82	1.15
	90 [G1]	Mean	0.090	0.064	0.064	0.043
		SD	0.057	0.042	0.038	0.013
		N	5	5	5	4
		tCtrl	-	0.71	0.71	0.47
BASO (10^3/uL)	8 [G]	Mean	0.098	0.058	0.098	0.114
		SD	0.030	0.030	0.016	0.179
		N	5	4	5	5
		tCtrl	-	0.59	1.00	1.16

[G] - Kruskal-Wallis & Dunn
[G1] - Anova & Dunnett
Day 90 represents all terminal collections done on day 90 (±4)

Appendix 9
Table 1

Summary of Hematology Values

2954-001

Sex: Female		Day(s) Relative to Start Date	Group 1	Group 2	Group 3	Group 4
BASO (10^3/uL)	29 [G]	Mean	0.042	0.100	0.090	0.144 **
		SD	0.030	0.045	0.055	0.044
		N	5	5	4	5
		tCtrl	-	2.38	2.14	3.43
	90 [G]	Mean	0.060	0.112	0.084	0.090
		SD	0.031	0.045	0.038	0.018
		N	5	5	5	4
		tCtrl	-	1.87	1.40	1.50
LUC (10^3/uL)	8 [G]	Mean	0.114	0.110	0.118	0.082
		SD	0.039	0.073	0.015	0.066
		N	5	4	5	5
		tCtrl	-	0.96	1.04	0.72
	29 [G]	Mean	0.142	0.176	0.120	0.322
		SD	0.146	0.080	0.054	0.209
		N	5	5	4	5
		tCtrl	-	1.24	0.85	2.27

[G] - Anova & Dunnett: ** = $p \leq 0.01$
Day 90 represents all terminal collections done on day 90 (±4)

Appendix 9
Table 1

Summary of Hematology Values

2954-001

Sex: Female<br>Day(s) Relative to Start Date			Group 1	Group 2	Group 3	Group 4
LUC (10^3/uL)	90 [G]	Mean	0.086	0.164	0.112	0.118
		SD	0.073	0.069	0.079	0.077
		N	5	5	5	4
		tCtrl	-	1.91	1.30	1.37
RBC (10^6/uL)	8 [G]	Mean	8.194	8.065	8.472	8.114
		SD	0.556	0.277	0.587	0.319
		N	5	4	5	5
		tCtrl	-	0.98	1.03	0.99
	29 [G]	Mean	8.906	8.496	9.073	8.760
		SD	0.789	0.336	0.824	0.482
		N	5	5	4	5
		tCtrl	-	0.95	1.02	0.98
	90 [G]	Mean	8.416	8.556	8.846	8.695
		SD	0.730	0.389	0.400	0.563
		N	5	5	5	4
		tCtrl	-	1.02	1.05	1.03

[G] - Anova & Dunnett
Day 90 represents all terminal collections done on day 90 (±4)

Appendix 9

Table 1

Summary of Hematology Values

2954-001

Sex: Female Day(s) Relative to Start Date			Group 1	Group 2	Group 3	Group 4
HGB (g/dL)	8 [G]	Mean	16.34	15.55	16.42	15.94
		SD	0.76	0.33	1.01	0.86
		N	5	4	5	5
		tCtrl	-	0.95	1.00	0.98
	29 [G]	Mean	16.44	15.82	16.43	16.48
		SD	1.44	0.58	1.11	0.62
		N	5	5	4	5
		tCtrl	-	0.96	1.00	1.00
	90 [G]	Mean	15.44	15.80	16.20	15.73
		SD	1.69	0.20	0.77	0.90
		N	5	5	5	4
		tCtrl	-	1.02	1.05	1.02
HCT (%)	8 [G]	Mean	53.40	51.00	54.28	51.64
		SD	2.95	0.94	2.81	3.48
		N	5	4	5	5
		tCtrl	-	0.96	1.02	0.97

[G] - Anova & Dunnett
Day 90 represents all terminal collections done on day 90 (±4)

Appendix 9
Table 1

Summary of Hematology Values

2954-001

Sex: Female / Day(s) Relative to Start Date			Group 1	Group 2	Group 3	Group 4
HCT (%)	29 [G]	Mean	55.30	53.04	55.73	55.08
		SD	5.35	2.12	3.60	1.44
		N	5	5	4	5
		tCtrl	-	0.96	1.01	1.00
	90 [G]	Mean	50.66	51.10	51.82	50.35
		SD	5.62	1.18	1.71	3.27
		N	5	5	5	4
		tCtrl	-	1.01	1.02	0.99
MCV (fL)	8 [G]	Mean	65.22	63.23	64.16	63.58
		SD	1.76	1.14	2.39	1.91
		N	5	4	5	5
		tCtrl	-	0.97	0.98	0.97
	29 [G1]	Mean	62.08	62.42	61.65	62.96
		SD	0.57	1.97	3.75	2.56
		N	5	5	4	5
		tCtrl	-	1.01	0.99	1.01

[G] - Anova & Dunnett
[G1] - Kruskal-Wallis & Dunn
Day 90 represents all terminal collections done on day 90 (±4)

Appendix 9

Table 1

Summary of Hematology Values

2954-001

Sex: Female<br>Day(s) Relative to Start Date			Group 1	Group 2	Group 3	Group 4
MCV (fL)	90 [G]	Mean	60.10	59.80	58.60	57.95
		SD	2.27	1.79	1.16	0.87
		N	5	5	5	4
		tCtrl	-	1.00	0.98	0.96
MCH (pg)	8 [G1]	Mean	19.98	19.25	19.40	19.62
		SD	0.80	0.31	0.44	0.40
		N	5	4	5	5
		tCtrl	-	0.96	0.97	0.98
	29 [G]	Mean	18.46	18.60	18.15	18.84
		SD	0.24	0.79	1.18	0.61
		N	5	5	4	5
		tCtrl	-	1.01	0.98	1.02
	90 [G1]	Mean	18.34	18.50	18.32	18.10
		SD	0.58	0.67	0.51	0.32
		N	5	5	5	4
		tCtrl	-	1.01	1.00	0.99

[G] - Kruskal-Wallis & Dunn
[G1] - Anova & Dunnett
Day 90 represents all terminal collections done on day 90 (±4)

Appendix 9
Table 1

Summary of Hematology Values

2954-001

Sex: Female<br>Day(s) Relative to Start Date			Group 1	Group 2	Group 3	Group 4
MCHC (g/dL)	8 [G]	Mean	30.60	30.45	30.24	30.88
		SD	0.60	0.37	0.52	0.75
		N	5	4	5	5
		tCtrl	-	1.00	0.99	1.01
	29 [G]	Mean	29.76	29.82	29.48	29.92
		SD	0.45	0.67	0.56	0.55
		N	5	5	4	5
		tCtrl	-	1.00	0.99	1.01
	90 [G]	Mean	30.50	30.96	31.26	31.25
		SD	0.78	0.38	0.55	0.37
		N	5	5	5	4
		tCtrl	-	1.02	1.02	1.02
RDW (%)	8 [G]	Mean	11.32	11.20	11.00	11.06
		SD	0.44	0.24	0.21	0.31
		N	5	4	5	5
		tCtrl	-	0.99	0.97	0.98

[G] - Anova & Dunnett
Day 90 represents all terminal collections done on day 90 (±4)

Appendix 9

Table 1

Summary of Hematology Values

2954-001

Sex: Female<br>Day(s) Relative to Start Date			Group 1	Group 2	Group 3	Group 4
RDW (%)	29 [G]	Mean	10.90	11.08	11.13	11.08
		SD	0.38	0.19	0.13	0.26
		N	5	5	4	5
		tCtrl	-	1.02	1.02	1.02
	90 [G]	Mean	11.50	11.88	11.74	11.55
		SD	0.42	0.75	0.56	0.30
		N	5	5	5	4
		tCtrl	-	1.03	1.02	1.00
PLT (10^3/uL)	8 [G]	Mean	1218.0	1091.8	1260.6	1060.4
		SD	71.8	202.2	131.0	168.0
		N	5	4	5	5
		tCtrl	-	0.90	1.03	0.87
	29 [G]	Mean	1041.3	1118.2	977.8	899.4
		SD	99.9	144.0	183.8	88.9
		N	4	5	4	5
		tCtrl	-	1.07	0.94	0.86

[G] - Anova & Dunnett
Day 90 represents all terminal collections done on day 90 (±4)

Appendix 9

Table 1

Summary of Hematology Values

2954-001

Sex: Female			Group 1	Group 2	Group 3	Group 4
Day(s) Relative to Start Date						
PLT (10^3/uL)	90 [G]	Mean	896.2	991.0	1039.6	1052.5
		SD	156.0	173.1	138.9	86.4
		N	5	5	5	4
		tCtrl	-	1.11	1.16	1.17
RETIC (10^9/L)	8 [G]	Mean	264.32	224.93	232.14	201.12
		SD	39.87	14.51	31.55	48.30
		N	5	4	5	5
		tCtrl	-	0.85	0.88	0.76
	29 [G]	Mean	247.32	234.56	211.65	243.98
		SD	50.61	31.41	33.20	46.12
		N	5	5	4	5
		tCtrl	-	0.95	0.86	0.99
	90 [G1]	Mean	182.02	165.78	172.26	139.85
		SD	47.87	28.52	17.05	20.87
		N	5	5	5	4
		tCtrl	-	0.91	0.95	0.77

[G] - Anova & Dunnett
[G1] - Kruskal-Wallis & Dunn
Day 90 represents all terminal collections done on day 90 (±4)

Appendix 9

Table 2

Abbreviations for Coagulation Parameters

APTT	-	Activated Partial Thromboplastin Time
FIB	-	Fibrinogen
PT	-	Prothrombin Time
UNSC	-	Unscheduled bleed

Appendix 9

Table 2

Summary of Coagulation Values

2954-001

Sex: Male	Day(s) Relative to Start Date		Group 1	Group 2	Group 3	Group 4
PT (sec)	8 [G]	Mean	17.50	16.78	17.82	17.00
		SD	0.26	0.80	0.47	0.40
		N	3	5	6	5
		tCtrl	-	0.96	1.02	0.97
	16 (Unsc) [I]	Mean	-	-	16.70 n	-
		SD	-	-	-	-
		N	-	-	1	-
	29 [G1]	Mean	16.18	15.88	16.08	15.02 *
		SD	0.29	0.73	1.25	0.20
		N	5	5	5	5
		tCtrl	-	0.98	0.99	0.93
	90 [G]	Mean	16.58	16.92	16.80	16.76
		SD	0.74	0.22	0.98	0.36
		N	4	5	4	5
		tCtrl	-	1.02	1.01	1.01
APTT (sec)	8 [G]	Mean	10.60	10.56	11.37	12.82
		SD	1.71	1.60	1.34	1.01
		N	3	5	6	5
		tCtrl	-	1.00	1.07	1.21

[G] - Anova & Dunnett
[G1] - Kruskal-Wallis & Dunn: * = $p \leq 0.05$
[I] - n - Inappropriate for statistics
Day 90 represents all terminal collections done on day 90 (±4)

Appendix 9

Table 2

Summary of Coagulation Values

2954-001

Sex: Male<br>Day(s) Relative to Start Date			Group 1	Group 2	Group 3	Group 4
APTT (sec)	16 (Unsc) [I]	Mean	-	-	9.30 n	-
		SD	-	-	-	-
		N	-	-	1	-
	29 [G]	Mean	13.68	12.66	12.40	13.28
		SD	1.35	0.93	1.37	1.10
		N	5	5	5	5
		tCtrl	-	0.93	0.91	0.97
	90 [G]	Mean	13.38	14.50	13.15	13.40
		SD	1.03	1.69	1.63	0.78
		N	4	5	4	5
		tCtrl	-	1.08	0.98	1.00
FIB (mg/dL)	8 [G1]	Mean	321.3	301.8	305.7	281.2
		SD	8.5	17.8	19.6	70.4
		N	3	5	6	5
		tCtrl	-	0.94	0.95	0.88
	16 (Unsc) [I]	Mean	-	-	329.0 n	-
		SD	-	-	-	-
		N	-	-	1	-

[G] - Anova & Dunnett
[I] - n - Inappropriate for statistics
[G1] - Kruskal-Wallis & Dunn
Day 90 represents all terminal collections done on day 90 (±4)

Appendix 9
Table 2

Summary of Coagulation Values

2954-001

Sex: Male / Day(s) Relative to Start Date			Group 1	Group 2	Group 3	Group 4
FIB (mg/dL)	29 [G]	Mean	291.2	297.4	287.4	291.2
		SD	36.9	49.1	24.7	27.5
		N	5	5	5	5
		tCtrl	-	1.02	0.99	1.00
	90 [G1]	Mean	323.5	346.6	344.3	336.8
		SD	35.2	18.0	15.8	62.3
		N	4	5	4	5
		tCtrl	-	1.07	1.06	1.04

[G] - Anova & Dunnett
[G1] - Kruskal-Wallis & Dunn
Day 90 represents all terminal collections done on day 90 (±4)

Appendix 9
Table 2

Summary of Coagulation Values

2954-001

Sex: Female / Day(s) Relative to Start Date			Group 1	Group 2	Group 3	Group 4
PT (sec)	8 [G]	Mean	16.04	15.00	15.70	15.52
		SD	0.67	0.23	0.32	0.91
		N	5	4	5	5
		tCtrl	-	0.94	0.98	0.97
	29 [G]	Mean	15.50	15.80	15.40	15.90
		SD	0.90	0.45	0.82	0.99
		N	5	5	3	4
		tCtrl	-	1.02	0.99	1.03
	90 [G]	Mean	17.10	16.22	16.76	15.95
		SD	0.88	0.67	0.75	0.98
		N	5	5	5	4
		tCtrl	-	0.95	0.98	0.93
APTT (sec)	8 [G]	Mean	13.00	10.08 *	11.90	13.02
		SD	1.19	1.25	2.19	1.24
		N	5	4	5	5
		tCtrl	-	0.78	0.92	1.00

[G] - Anova & Dunnett: * = $p \leq 0.05$
Day 90 represents all terminal collections done on day 90 (±4)

Appendix 9

Table 2

Summary of Coagulation Values

2954-001

Sex: Female<br>Day(s) Relative to Start Date			Group 1	Group 2	Group 3	Group 4
APTT (sec)	29 [G]	Mean	12.24	12.56	11.80	12.68
		SD	1.95	1.61	3.08	0.65
		N	5	5	3	4
		tCtrl	-	1.03	0.96	1.04
	90 [G1]	Mean	12.74	10.50	12.20	11.63
		SD	0.59	2.14	1.11	1.75
		N	5	5	5	4
		tCtrl	-	0.82	0.96	0.91
FIB (mg/dL)	8 [G]	Mean	296.4	285.8	282.6	274.8
		SD	16.4	12.3	17.6	11.6
		N	5	4	5	5
		tCtrl	-	0.96	0.95	0.93
	29 [G]	Mean	221.0	224.4	216.0	234.5
		SD	16.7	16.6	29.9	11.4
		N	5	5	3	4
		tCtrl	-	1.02	0.98	1.06

[G] - Anova & Dunnett
[G1] - Kruskal-Wallis & Dunn
Day 90 represents all terminal collections done on day 90 (±4)

Appendix 9

Table 2

Summary of Coagulation Values

2954-001

Sex: Female<br>Day(s) Relative to Start Date			Group 1	Group 2	Group 3	Group 4
FIB (mg/dL)	90 [G]	Mean	245.4	240.2	242.2	245.8
		SD	40.0	41.2	27.0	31.6
		N	5	5	5	4
		tCtrl	-	0.98	0.99	1.00

[G] - Anova & Dunnett
Day 90 represents all terminal collections done on day 90 (±4)

Appendix 9
Table 3

Abbreviations for Clinical Chemistry Parameters

AST	-	Aspartate Aminotransferase
ALT	-	Alanine Aminotransferase
ALP	-	Alkaline Phosphatase
GGT	-	Gamma Glutamyl Transferase
TBIL	-	Total Bilirubin
UREAN	-	Urea Nitrogen
CREAT	-	Creatinine
GLUC	-	Glucose
CHOL	-	Cholesterol
TRIG	-	Triglycerides
TPROT	-	Total Protein
ALB	-	Albumin
GLOB	-	Globulin
A/G	-	Albumin/Globulin Ratio
CA	-	Calcium
PHOS	-	Phosphorus
NA	-	Sodium
K	-	Potassium
CL	-	Chloride
UNSC	-	Unscheduled bleed

Appendix 9

Table 3

Summary of Clinical Chemistry Values

2954-001

Sex: Male<br>Day(s) Relative to Start Date			Group 1	Group 2	Group 3	Group 4
AST (U/L)	8 [G]	Mean	122.0	89.0	78.8	141.4
		SD	56.5	16.2	5.9	40.4
		N	5	5	6	5
		tCtrl	-	0.73	0.65	1.16
	16 (Unsc) [I]	Mean	-	-	99.0 n	-
		SD	-	-	-	-
		N	-	-	1	-
	29 [G1]	Mean	87.2	140.8	100.8	103.6
		SD	9.9	38.4	30.4	31.9
		N	5	5	5	5
		tCtrl	-	1.61	1.16	1.19
	90 [G]	Mean	100.4	80.8	97.0	124.0
		SD	11.8	15.8	10.1	50.0
		N	5	5	4	5
		tCtrl	-	0.80	0.97	1.24
ALT (U/L)	8 [G1]	Mean	40.8	29.4	31.7	50.2
		SD	11.2	4.8	4.7	18.1
		N	5	5	6	5
		tCtrl	-	0.72	0.78	1.23

[G] - Kruskal-Wallis & Dunn
[G1] - Anova & Dunnett
[I] - n - Inappropriate for statistics
Day 90 represents all terminal collections done on day 90 (±4)

Appendix 9

Table 3

Summary of Clinical Chemistry Values

2954-001

Sex: Male<br>Day(s) Relative to Start Date			Group 1	Group 2	Group 3	Group 4
ALT (U/L)	16 (Unsc) [I]	Mean	-	-	69.0 n	-
		SD	-	-	-	-
		N	-	-	1	-
	29 [G]	Mean	48.2	61.8	43.0	39.2
		SD	12.9	17.5	8.8	3.6
		N	5	5	5	5
		tCtrl	-	1.28	0.89	0.81
	90 [G1]	Mean	43.0	34.4	40.3	48.8
		SD	6.5	5.0	12.0	14.9
		N	5	5	4	5
		tCtrl	-	0.80	0.94	1.13
ALP (U/L)	8 [G]	Mean	279.6	273.8	247.7	326.0
		SD	35.2	60.2	47.7	112.6
		N	5	5	6	5
		tCtrl	-	0.98	0.89	1.17
	16 (Unsc) [I]	Mean	-	-	208.0 n	-
		SD	-	-	-	-
		N	-	-	1	-

[G] - Kruskal-Wallis & Dunn
[G1] - Anova & Dunnett
[I] - n - Inappropriate for statistics
Day 90 represents all terminal collections done on day 90 (±4)

Appendix 9
Table 3

Summary of Clinical Chemistry Values

2954-001

Sex: Male	Day(s) Relative to Start Date		Group 1	Group 2	Group 3	Group 4
ALP (U/L)	29 [G]	Mean	180.0	211.2	211.8	165.6
		SD	45.3	31.4	32.0	36.5
		N	5	5	5	5
		tCtrl	-	1.17	1.18	0.92
	90 [G]	Mean	103.6	107.4	89.3	104.4
		SD	27.2	18.4	23.3	19.3
		N	5	5	4	5
		tCtrl	-	1.04	0.86	1.01
TBIL (mg/dL)	8 [G]	Mean	0.112	0.106	0.105	0.132
		SD	0.004	0.013	0.014	0.024
		N	5	5	6	5
		tCtrl	-	0.95	0.94	1.18
	16 (Unsc) [I]	Mean	-	-	0.130 n	-
		SD	-	-	-	-
		N	-	-	1	-
	29 [G]	Mean	0.120	0.134	0.130	0.148
		SD	0.016	0.015	0.016	0.018
		N	5	5	5	5
		tCtrl	-	1.12	1.08	1.23

[G] - Anova & Dunnett
[I] - n - Inappropriate for statistics
Day 90 represents all terminal collections done on day 90 (±4)

Appendix 9

Table 3

Summary of Clinical Chemistry Values

2954-001

Sex: Male		Day(s) Relative to Start Date	Group 1	Group 2	Group 3	Group 4
TBIL (mg/dL)	90 [G]	Mean	0.154	0.152	0.158	0.136
		SD	0.013	0.022	0.044	0.035
		N	5	5	4	5
		tCtrl	-	0.99	1.02	0.88
UREAN (mg/dL)	8 [G1]	Mean	13.8	13.8	13.2	16.6
		SD	2.4	2.2	2.9	2.9
		N	5	5	6	5
		tCtrl	-	1.00	0.95	1.20
	16 (Unsc) [I]	Mean	-	-	18.0 n	-
		SD	-	-	-	-
		N	-	-	1	-
	29 [G]	Mean	14.0	15.6	17.0	16.0
		SD	1.4	1.1	4.2	1.4
		N	5	5	5	5
		tCtrl	-	1.11	1.21	1.14
	90 [G1]	Mean	16.0	16.6	17.5	16.8
		SD	2.1	1.9	3.0	0.4
		N	5	5	4	5
		tCtrl	-	1.04	1.09	1.05

[G] - Kruskal-Wallis & Dunn
[G1] - Anova & Dunnett
[I] - n - Inappropriate for statistics
Day 90 represents all terminal collections done on day 90 (±4)

Appendix 9

Table 3

Summary of Clinical Chemistry Values

2954-001

Sex: Male<br>Day(s) Relative to Start Date			Group 1	Group 2	Group 3	Group 4
CREAT (mg/dL)	8 [G]	Mean	0.36	0.30	0.33	0.36
		SD	0.05	0.00	0.05	0.05
		N	5	5	6	5
		tCtrl	-	0.83	0.93	1.00
	16 (Unsc) [I]	Mean	-	-	0.40 n	-
		SD	-	-	-	-
		N	-	-	1	-
	29 [G1]	Mean	0.40	0.38	0.36	0.36
		SD	0.07	0.04	0.09	0.05
		N	5	5	5	5
		tCtrl	-	0.95	0.90	0.90
	90 [G]	Mean	0.50	0.50	0.48	0.48
		SD	0.00	0.10	0.10	0.04
		N	5	5	4	5
		tCtrl	-	1.00	0.95	0.96
GLUC (mg/dL)	8 [G1]	Mean	127.4	121.8	150.8	142.6
		SD	63.4	53.2	93.6	46.3
		N	5	5	6	5
		tCtrl	-	0.96	1.18	1.12

[G] - Kruskal-Wallis & Dunn
[G1] - Anova & Dunnett
[I] - n - Inappropriate for statistics
Day 90 represents all terminal collections done on day 90 (±4)

Appendix 9

Table 3

Summary of Clinical Chemistry Values

2954-001

Sex: Male		Day(s) Relative to Start Date	Group 1	Group 2	Group 3	Group 4
GLUC (mg/dL)	16 (Unsc) [I]	Mean	-	-	492.0 n	-
		SD	-	-	-	-
		N	-	-	1	-
	29 [G]	Mean	291.6	301.2	304.0	244.8
		SD	164.8	74.2	114.3	95.1
		N	5	5	5	5
		tCtrl	-	1.03	1.04	0.84
	90 [G]	Mean	255.4	296.0	319.3	280.0
		SD	120.4	130.0	74.8	130.7
		N	5	5	4	5
		tCtrl	-	1.16	1.25	1.10
CHOL (mg/dL)	8 [G]	Mean	69.6	73.6	73.8	69.0
		SD	6.4	9.4	10.9	16.1
		N	5	5	6	5
		tCtrl	-	1.06	1.06	0.99
	16 (Unsc) [I]	Mean	-	-	78.0 n	-
		SD	-	-	-	-
		N	-	-	1	-

[G] - Anova & Dunnett
[I] - n - Inappropriate for statistics
Day 90 represents all terminal collections done on day 90 (±4)

Appendix 9

Table 3

Summary of Clinical Chemistry Values

2954-001

Sex: Male<br>Day(s) Relative to Start Date			Group 1	Group 2	Group 3	Group 4
CHOL (mg/dL)	29 [G]	Mean	55.2	78.2	66.2	63.4
		SD	7.1	18.3	16.3	15.9
		N	5	5	5	5
		tCtrl	-	1.42	1.20	1.15
	90 [G]	Mean	72.0	76.2	79.8	68.6
		SD	14.4	12.2	20.7	20.1
		N	5	5	4	5
		tCtrl	-	1.06	1.11	0.95
TRIG (mg/dL)	8 [G]	Mean	84.2	74.2	79.2	55.4
		SD	26.5	13.5	16.5	14.3
		N	5	5	6	5
		tCtrl	-	0.88	0.94	0.66
	16 (Unsc) [I]	Mean	-	-	123.0 n	-
		SD	-	-	-	-
		N	-	-	1	-
	29 [G]	Mean	53.2	65.6	62.4	63.2
		SD	17.4	21.3	21.7	11.2
		N	5	5	5	5
		tCtrl	-	1.23	1.17	1.19

[G] - Anova & Dunnett
[I] - n - Inappropriate for statistics
Day 90 represents all terminal collections done on day 90 (±4)

Appendix 9
Table 3

Summary of Clinical Chemistry Values

2954-001

Sex: Male	Day(s) Relative to Start Date		Group 1	Group 2	Group 3	Group 4
TRIG (mg/dL)	90 [G]	Mean	69.8	86.2	72.0	75.4
		SD	16.4	39.4	16.0	15.0
		N	5	5	4	5
		tCtrl	-	1.23	1.03	1.08
TPROT (g/dL)	8 [G]	Mean	6.10	5.96	5.98	6.22
		SD	0.27	0.31	0.35	0.20
		N	5	5	6	5
		tCtrl	-	0.98	0.98	1.02
	16 (Unsc) [I]	Mean	-	-	7.30 n	-
		SD	-	-	-	-
		N	-	-	1	-
	29 [G]	Mean	6.78	6.74	6.70	6.98
		SD	0.65	0.27	0.32	0.41
		N	5	5	5	5
		tCtrl	-	0.99	0.99	1.03
	90 [G]	Mean	6.68	6.98	7.25	6.68
		SD	0.28	0.29	0.24	0.46
		N	5	5	4	5
		tCtrl	-	1.04	1.09	1.00

[G] - Anova & Dunnett
[I] - n - Inappropriate for statistics
Day 90 represents all terminal collections done on day 90 (±4)

Appendix 9
Table 3

Summary of Clinical Chemistry Values

2954-001

Sex: Male / Day(s) Relative to Start Date			Group 1	Group 2	Group 3	Group 4
ALB (g/dL)	8 [G]	Mean	3.50	3.40	3.43	3.48
		SD	0.14	0.14	0.15	0.11
		N	5	5	6	5
		tCtrl	-	0.97	0.98	0.99
	16 (Unsc) [I]	Mean	-	-	3.80 n	-
		SD	-	-	-	-
		N	-	-	1	-
	29 [G]	Mean	3.46	3.40	3.50	3.52
		SD	0.22	0.25	0.20	0.19
		N	5	5	5	5
		tCtrl	-	0.98	1.01	1.02
	90 [G]	Mean	3.54	3.58	3.68	3.50
		SD	0.11	0.08	0.05	0.10
		N	5	5	4	5
		tCtrl	-	1.01	1.04	0.99
GLOB (g/dL)	8 [G]	Mean	2.60	2.56	2.55	2.74
		SD	0.16	0.18	0.21	0.11
		N	5	5	6	5
		tCtrl	-	0.98	0.98	1.05

[G] - Anova & Dunnett
[I] - n - Inappropriate for statistics
Day 90 represents all terminal collections done on day 90 (±4)

Appendix 9

Table 3

Summary of Clinical Chemistry Values

2954-001

Sex: Male<br>Day(s) Relative to Start Date			Group 1	Group 2	Group 3	Group 4
GLOB (g/dL)	16 (Unsc) [I]	Mean	-	-	3.50 n	-
		SD	-	-	-	-
		N	-	-	1	-
	29 [G]	Mean	3.32	3.34	3.20	3.46
		SD	0.44	0.21	0.12	0.24
		N	5	5	5	5
		tCtrl	-	1.01	0.96	1.04
	90 [G]	Mean	3.14	3.40	3.58	3.18
		SD	0.24	0.26	0.22	0.41
		N	5	5	4	5
		tCtrl	-	1.08	1.14	1.01
A/G (ratio)	8 [G]	Mean	1.34	1.32	1.37	1.28
		SD	0.05	0.04	0.05	0.04
		N	5	5	6	5
		tCtrl	-	0.99	1.02	0.96
	16 (Unsc) [I]	Mean	-	-	1.10 n	-
		SD	-	-	-	-
		N	-	-	1	-

[G] - Anova & Dunnett
[I] - n - Inappropriate for statistics
Day 90 represents all terminal collections done on day 90 (±4)

Appendix 9
Table 3

Summary of Clinical Chemistry Values

2954-001

Sex: Male<br>Day(s) Relative to Start Date			Group 1	Group 2	Group 3	Group 4
A/G (ratio)	29 [G]	Mean	1.04	1.02	1.10	1.02
		SD	0.05	0.08	0.00	0.04
		N	5	5	5	5
		tCtrl	-	0.98	1.06	0.98
	90 [G1]	Mean	1.12	1.04	1.03	1.14
		SD	0.11	0.09	0.10	0.15
		N	5	5	4	5
		tCtrl	-	0.93	0.92	1.02
CA (mg/dL)	8 [G1]	Mean	13.34	13.00	12.90	12.60
		SD	0.81	0.90	0.56	0.65
		N	5	5	6	5
		tCtrl	-	0.97	0.97	0.94
	16 (Unsc) [I]	Mean	-	-	13.80 n	-
		SD	-	-	-	-
		N	-	-	1	-
	29 [G1]	Mean	12.82	12.98	12.84	13.56
		SD	1.23	0.38	0.60	1.14
		N	5	5	5	5
		tCtrl	-	1.01	1.00	1.06

[G] - Kruskal-Wallis & Dunn
[G1] - Anova & Dunnett
[I] - n - Inappropriate for statistics
Day 90 represents all terminal collections done on day 90 (±4)

Appendix 9

Table 3

Summary of Clinical Chemistry Values

2954-001

Sex: Male<br>Day(s) Relative to Start Date			Group 1	Group 2	Group 3	Group 4
CA (mg/dL)	90 [G]	Mean	12.32	12.74	12.80	12.64
		SD	1.00	0.49	0.42	0.63
		N	5	5	4	5
		tCtrl	-	1.03	1.04	1.03
PHOS (mg/dL)	8 [G1]	Mean	16.70	15.26	15.53	15.10
		SD	1.20	1.05	1.86	1.88
		N	5	5	6	5
		tCtrl	-	0.91	0.93	0.90
	16 (Unsc) [I]	Mean	-	-	12.90 n	-
		SD	-	-	-	-
		N	-	-	1	-
	29 [G]	Mean	13.90	12.88	13.38	14.76
		SD	1.20	1.02	1.12	0.79
		N	5	5	5	5
		tCtrl	-	0.93	0.96	1.06
	90 [G]	Mean	11.72	11.26	12.30	12.06
		SD	1.76	1.17	0.61	2.78
		N	5	5	4	5
		tCtrl	-	0.96	1.05	1.03

[G] - Anova & Dunnett
[G1] - Kruskal-Wallis & Dunn
[I] - n - Inappropriate for statistics
Day 90 represents all terminal collections done on day 90 (±4)

Appendix 9
Table 3

Summary of Clinical Chemistry Values

2954-001

Sex: Male	Day(s) Relative to Start Date		Group 1	Group 2	Group 3	Group 4
NA (mEq/L)	8 [G]	Mean	145.4	147.0	145.8	144.0
		SD	2.4	1.0	1.2	2.9
		N	5	5	6	5
		tCtrl	-	1.01	1.00	0.99
	16 (Unsc) [I]	Mean	-	-	142.0 n	-
		SD	-	-	-	-
		N	-	-	1	-
	29 [G1]	Mean	147.8	147.0	144.6	145.6
		SD	2.3	2.4	2.1	2.7
		N	5	5	5	5
		tCtrl	-	0.99	0.98	0.99
	90 [G1]	Mean	145.6	146.2	144.8	144.4
		SD	2.7	2.6	1.7	3.4
		N	5	5	4	5
		tCtrl	-	1.00	0.99	0.99
K (mEq/L)	8 [G1]	Mean	10.90	9.44	10.00	10.32
		SD	1.70	1.45	1.29	2.98
		N	5	5	6	5
		tCtrl	-	0.87	0.92	0.95

[G] - Kruskal-Wallis & Dunn
[G1] - Anova & Dunnett
[I] - n - Inappropriate for statistics
Day 90 represents all terminal collections done on day 90 (±4)

Appendix 9

Table 3

Summary of Clinical Chemistry Values

2954-001

Sex: Male	Day(s) Relative to Start Date		Group 1	Group 2	Group 3	Group 4
K (mEq/L)	16 (Unsc) [I]	Mean	-	-	9.60 n	-
		SD	-	-	-	-
		N	-	-	1	-
	29 [G]	Mean	9.96	8.64	9.98	10.50
		SD	1.03	0.95	1.77	2.00
		N	5	5	5	5
		tCtrl	-	0.87	1.00	1.05
	90 [G]	Mean	9.90	9.28	10.48	10.44
		SD	2.60	1.29	1.12	3.78
		N	5	5	4	5
		tCtrl	-	0.94	1.06	1.05
CL (mEq/L)	8 [G]	Mean	100.4	103.8	101.2	101.8
		SD	1.7	2.7	2.8	1.3
		N	5	5	6	5
		tCtrl	-	1.03	1.01	1.01
	16 (Unsc) [I]	Mean	-	-	101.0 n	-
		SD	-	-	-	-
		N	-	-	1	-

[G] - Anova & Dunnett
[I] - n - Inappropriate for statistics
Day 90 represents all terminal collections done on day 90 (±4)

Appendix 9

Table 3

Summary of Clinical Chemistry Values

2954-001

Sex: Male<br>Day(s) Relative to Start Date			Group 1	Group 2	Group 3	Group 4
CL (mEq/L)	29 [G]	Mean	101.2	100.4	99.8	99.6
		SD	2.2	3.4	2.0	1.9
		N	5	5	5	5
		tCtrl	-	0.99	0.99	0.98
	90 [G1]	Mean	100.8	100.6	100.0	100.8
		SD	2.2	1.9	0.0	0.4
		N	5	5	4	5
		tCtrl	-	1.00	0.99	1.00

[G] - Anova & Dunnett
[G1] - Kruskal-Wallis & Dunn
Day 90 represents all terminal collections done on day 90 (±4)

Appendix 9

Table 3

Summary of Clinical Chemistry Values

2954-001

Sex: Female<br>Day(s) Relative to Start Date			Group 1	Group 2	Group 3	Group 4
AST (U/L)	8 [G]	Mean	107.8	91.8	97.2	103.3
		SD	71.5	22.3	39.9	9.8
		N	5	5	5	4
		tCtrl	-	0.85	0.90	0.96
	29 [G1]	Mean	657.2	118.4	497.4	104.0
		SD	842.2	32.3	549.9	28.5
		N	5	5	5	5
		tCtrl	-	0.18	0.76	0.16
	90 [G1]	Mean	104.6	121.0	115.0	135.8
		SD	5.5	32.7	45.4	57.4
		N	5	5	5	5
		tCtrl	-	1.16	1.10	1.30
ALT (U/L)	8 [G]	Mean	49.0	37.4	49.0	32.3
		SD	35.7	7.6	20.8	5.4
		N	5	5	5	4
		tCtrl	-	0.76	1.00	0.66

[G] - Anova & Dunnett
[G1] - Kruskal-Wallis & Dunn
Day 90 represents all terminal collections done on day 90 (±4)

Appendix 9

Table 3

Summary of Clinical Chemistry Values

2954-001

Sex: Female<br>Day(s) Relative to Start Date			Group 1	Group 2	Group 3	Group 4
ALT (U/L)	29 [G]	Mean	326.2	51.4	207.6	43.4
		SD	415.9	23.2	254.3	14.5
		N	5	5	5	5
		tCtrl	-	0.16	0.64	0.13
	90 [G1]	Mean	58.6	54.0	57.0	57.0
		SD	14.3	10.7	31.3	33.9
		N	5	5	5	5
		tCtrl	-	0.92	0.97	0.97
ALP (U/L)	8 [G1]	Mean	183.2	169.4	168.0	173.3
		SD	52.9	46.1	78.4	31.5
		N	5	5	5	4
		tCtrl	-	0.92	0.92	0.95
	29 [G]	Mean	121.2	88.4	102.0	117.6
		SD	20.6	13.9	33.3	16.7
		N	5	5	5	5
		tCtrl	-	0.73	0.84	0.97

[G] - Kruskal-Wallis & Dunn
[G1] - Anova & Dunnett
Day 90 represents all terminal collections done on day 90 (±4)

Appendix 9

Table 3

Summary of Clinical Chemistry Values

2954-001

Sex: Female Day(s) Relative to Start Date			Group 1	Group 2	Group 3	Group 4
ALP (U/L)	90 [G]	Mean	104.8	87.2	51.2	46.4
		SD	75.5	25.9	5.6	1.5
		N	5	5	5	5
		tCtrl	-	0.83	0.49	0.44
TBIL (mg/dL)	8 [G1]	Mean	0.104	0.100	0.102	0.105
		SD	0.019	0.020	0.016	0.017
		N	5	5	5	4
		tCtrl	-	0.96	0.98	1.01
	29 [G]	Mean	0.148	0.142	0.156	0.144
		SD	0.022	0.013	0.046	0.017
		N	5	5	5	5
		tCtrl	-	0.96	1.05	0.97
	90 [G1]	Mean	0.146	0.146	0.168	0.162
		SD	0.017	0.015	0.022	0.023
		N	5	5	5	5
		tCtrl	-	1.00	1.15	1.11

[G] - Kruskal-Wallis & Dunn
[G1] - Anova & Dunnett
Day 90 represents all terminal collections done on day 90 (±4)

Appendix 9

Table 3

Summary of Clinical Chemistry Values

2954-001

Sex: Female<br>Day(s) Relative to Start Date			Group 1	Group 2	Group 3	Group 4
UREAN (mg/dL)	8 [G]	Mean	15.8	19.2	17.4	15.0
		SD	3.5	2.5	2.9	1.8
		N	5	5	5	4
		tCtrl	-	1.22	1.10	0.95
	29 [G]	Mean	17.6	15.6	17.0	16.8
		SD	2.3	2.7	2.8	2.4
		N	5	5	5	5
		tCtrl	-	0.89	0.97	0.95
	90 [G]	Mean	19.2	18.0	18.0	17.0
		SD	2.3	1.7	2.0	2.4
		N	5	5	5	5
		tCtrl	-	0.94	0.94	0.89
CREAT (mg/dL)	8 [G]	Mean	0.40	0.44	0.46	0.43
		SD	0.07	0.05	0.05	0.05
		N	5	5	5	4
		tCtrl	-	1.10	1.15	1.06

[G] - Anova & Dunnett
Day 90 represents all terminal collections done on day 90 (±4)

Appendix 9
Table 3

Summary of Clinical Chemistry Values

2954-001

Sex: Female<br>Day(s) Relative to Start Date			Group 1	Group 2	Group 3	Group 4
CREAT (mg/dL)	29 [G]	Mean	0.46	0.40	0.40	0.38
		SD	0.05	0.07	0.07	0.04
		N	5	5	5	5
		tCtrl	-	0.87	0.87	0.83
	90 [G]	Mean	0.62	0.58	0.52	0.54
		SD	0.08	0.08	0.04	0.05
		N	5	5	5	5
		tCtrl	-	0.94	0.84	0.87
GLUC (mg/dL)	8 [G1]	Mean	299.8	331.8	337.0	285.5
		SD	140.1	64.5	95.1	59.5
		N	5	5	5	4
		tCtrl	-	1.11	1.12	0.95
	29 [G]	Mean	206.4	195.2	343.4	265.2
		SD	66.1	89.2	129.8	100.5
		N	5	5	5	5
		tCtrl	-	0.95	1.66	1.28

[G] - Anova & Dunnett
[G1] - Kruskal-Wallis & Dunn
Day 90 represents all terminal collections done on day 90 (±4)

Appendix 9

Table 3

Summary of Clinical Chemistry Values

2954-001

Sex: Female<br>Day(s) Relative to Start Date			Group 1	Group 2	Group 3	Group 4
GLUC (mg/dL)	90 [G]	Mean	269.8	289.2	199.8	170.4
		SD	137.6	128.9	95.7	101.7
		N	5	5	5	5
		tCtrl	-	1.07	0.74	0.63
CHOL (mg/dL)	8 [G]	Mean	70.6	69.4	79.0	71.5
		SD	13.7	19.3	10.1	8.7
		N	5	5	5	4
		tCtrl	-	0.98	1.12	1.01
	29 [G]	Mean	71.2	58.6	73.2	70.2
		SD	11.5	15.8	5.9	17.5
		N	5	5	5	5
		tCtrl	-	0.82	1.03	0.99
	90 [G]	Mean	88.0	86.0	81.6	80.2
		SD	27.1	10.6	7.6	13.2
		N	5	5	5	5
		tCtrl	-	0.98	0.93	0.91

[G] - Anova & Dunnett
Day 90 represents all terminal collections done on day 90 (±4)

Appendix 9

Table 3

Summary of Clinical Chemistry Values

2954-001

Sex: Female	Day(s) Relative to Start Date		Group 1	Group 2	Group 3	Group 4
TRIG (mg/dL)	8 [G]	Mean	47.8	44.6	50.0	43.5
		SD	11.2	10.3	14.5	4.1
		N	5	5	5	4
		tCtrl	-	0.93	1.05	0.91
	29 [G]	Mean	61.6	51.2	63.2	49.8
		SD	7.6	18.5	17.0	10.7
		N	5	5	5	5
		tCtrl	-	0.83	1.03	0.81
	90 [G1]	Mean	125.2	109.8	58.8	51.8 *
		SD	46.4	59.0	15.2	9.0
		N	5	5	5	5
		tCtrl	-	0.88	0.47	0.41
TPROT (g/dL)	8 [G1]	Mean	6.56	6.42	7.18	6.70
		SD	0.39	0.26	0.45	0.66
		N	5	5	5	4
		tCtrl	-	0.98	1.09	1.02

[G] - Anova & Dunnett
[G1] - Kruskal-Wallis & Dunn: * = p ≤ 0.05
Day 90 represents all terminal collections done on day 90 (±4)

Appendix 9

Table 3

Summary of Clinical Chemistry Values

2954-001

Sex: Female<br>Day(s) Relative to Start Date			Group 1	Group 2	Group 3	Group 4
TPROT (g/dL)	29 [G]	Mean	7.34	7.20	7.22	7.64
		SD	0.56	0.60	0.53	0.42
		N	5	5	5	5
		tCtrl	-	0.98	0.98	1.04
	90 [G]	Mean	8.44	8.16	7.78	7.88
		SD	0.40	0.49	0.24	0.54
		N	5	5	5	5
		tCtrl	-	0.97	0.92	0.93
ALB (g/dL)	8 [G1]	Mean	3.90	3.74	4.14	3.90
		SD	0.24	0.13	0.30	0.35
		N	5	5	5	4
		tCtrl	-	0.96	1.06	1.00
	29 [G]	Mean	4.02	3.86	3.84	3.94
		SD	0.28	0.24	0.24	0.26
		N	5	5	5	5
		tCtrl	-	0.96	0.96	0.98

[G] - Anova & Dunnett
[G1] - Kruskal-Wallis & Dunn
Day 90 represents all terminal collections done on day 90 (±4)

Appendix 9
Table 3

Summary of Clinical Chemistry Values

2954-001

Sex: Female — Day(s) Relative to Start Date			Group 1	Group 2	Group 3	Group 4
ALB (g/dL)	90 [G]	Mean	4.52	4.36	4.08	4.24
		SD	0.19	0.34	0.28	0.23
		N	5	5	5	5
		tCtrl	-	0.96	0.90	0.94
GLOB (g/dL)	8 [G]	Mean	2.66	2.68	3.04 *	2.80
		SD	0.17	0.15	0.17	0.34
		N	5	5	5	4
		tCtrl	-	1.01	1.14	1.05
	29 [G]	Mean	3.32	3.34	3.38	3.70
		SD	0.33	0.36	0.34	0.25
		N	5	5	5	5
		tCtrl	-	1.01	1.02	1.11
	90 [G]	Mean	3.92	3.80	3.70	3.64
		SD	0.26	0.16	0.23	0.38
		N	5	5	5	5
		tCtrl	-	0.97	0.94	0.93

[G] - Anova & Dunnett: * = $p \leq 0.05$
Day 90 represents all terminal collections done on day 90 (±4)

Appendix 9
Table 3

Summary of Clinical Chemistry Values

2954-001

Sex: Female<br>Day(s) Relative to Start Date			Group 1	Group 2	Group 3	Group 4
A/G (ratio)	8 [G]	Mean	1.46	1.38	1.36	1.38
		SD	0.09	0.08	0.05	0.10
		N	5	5	5	4
		tCtrl	-	0.95	0.93	0.94
	29 [G]	Mean	1.22	1.14	1.14	1.08
		SD	0.08	0.05	0.09	0.08
		N	5	5	5	5
		tCtrl	-	0.93	0.93	0.89
	90 [G]	Mean	1.14	1.16	1.12	1.18
		SD	0.09	0.05	0.15	0.11
		N	5	5	5	5
		tCtrl	-	1.02	0.98	1.04
CA (mg/dL)	8 [G]	Mean	13.32	13.36	14.06	13.55
		SD	0.83	0.23	0.47	0.41
		N	5	5	5	4
		tCtrl	-	1.00	1.06	1.02

[G] - Anova & Dunnett
Day 90 represents all terminal collections done on day 90 (±4)

Appendix 9

Table 3

Summary of Clinical Chemistry Values

2954-001

Sex: Female	Day(s) Relative to Start Date		Group 1	Group 2	Group 3	Group 4
CA (mg/dL)	29 [G]	Mean	12.84	12.70	13.80	13.64
		SD	1.10	0.74	2.02	0.61
		N	5	5	5	5
		tCtrl	-	0.99	1.07	1.06
	90 [G]	Mean	13.74	13.40	12.52	12.90
		SD	1.19	0.81	0.57	0.53
		N	5	5	5	5
		tCtrl	-	0.98	0.91	0.94
PHOS (mg/dL)	8 [G]	Mean	14.92	14.42	16.50	15.75
		SD	1.68	0.89	2.56	2.67
		N	5	5	5	4
		tCtrl	-	0.97	1.11	1.06
	29 [G]	Mean	14.06	13.00	13.50	13.00
		SD	2.36	1.12	2.12	0.32
		N	5	5	5	5
		tCtrl	-	0.92	0.96	0.92

[G] - Anova & Dunnett
Day 90 represents all terminal collections done on day 90 (±4)

Appendix 9

Table 3

Summary of Clinical Chemistry Values

2954-001

Sex: Female / Day(s) Relative to Start Date			Group 1	Group 2	Group 3	Group 4
PHOS (mg/dL)	90 [G]	Mean	11.38	11.10	11.14	13.04
		SD	4.66	1.56	0.93	2.04
		N	5	5	5	5
		tCtrl	-	0.98	0.98	1.15
NA (mEq/L)	8 [G1]	Mean	141.2	141.8	139.2	142.8
		SD	1.8	2.8	4.8	2.4
		N	5	5	5	4
		tCtrl	-	1.00	0.99	1.01
	29 [G1]	Mean	142.0	144.4	141.8	142.6
		SD	3.6	1.9	1.9	1.1
		N	5	5	5	5
		tCtrl	-	1.02	1.00	1.00
	90 [G1]	Mean	144.2	144.4	142.8	141.0
		SD	2.7	3.0	1.1	0.7
		N	5	5	5	5
		tCtrl	-	1.00	0.99	0.98

[G] - Kruskal-Wallis & Dunn
[G1] - Anova & Dunnett
Day 90 represents all terminal collections done on day 90 (±4)

Appendix 9
Table 3

Summary of Clinical Chemistry Values

2954-001

Sex: Female Day(s) Relative to Start Date			Group 1	Group 2	Group 3	Group 4
K (mEq/L)	8 [G]	Mean	10.86	10.64	12.88	10.55
		SD	1.27	1.00	4.11	0.77
		N	5	5	5	4
		tCtrl	-	0.98	1.19	0.97
	29 [G]	Mean	11.40	11.20	10.84	10.04
		SD	3.57	3.40	1.05	0.46
		N	5	5	5	5
		tCtrl	-	0.98	0.95	0.88
	90 [G]	Mean	9.62	9.32	10.34	12.52
		SD	3.58	2.49	1.00	1.46
		N	5	5	5	5
		tCtrl	-	0.97	1.07	1.30
CL (mEq/L)	8 [G]	Mean	101.2	102.6	100.4	100.0
		SD	2.4	1.3	1.5	1.2
		N	5	5	5	4
		tCtrl	-	1.01	0.99	0.99

[G] - Anova & Dunnett
Day 90 represents all terminal collections done on day 90 (±4)

Appendix 9

Table 3

Summary of Clinical Chemistry Values

2954-001

Sex: Female<br>Day(s) Relative to Start Date			Group 1	Group 2	Group 3	Group 4
CL (mEq/L)	29 [G]	Mean	100.6	101.8	101.0	100.2
		SD	1.8	2.7	1.6	0.8
		N	5	5	5	5
		tCtrl	-	1.01	1.00	1.00
	90 [G]	Mean	100.4	100.2	101.6	100.8
		SD	1.7	1.8	1.9	1.3
		N	5	5	5	5
		tCtrl	-	1.00	1.01	1.00

[G] - Anova & Dunnett
Day 90 represents all terminal collections done on day 90 (±4)

Appendix 9

Appendix 1

Codes for Individual Hematology Values

WBC	-	White Blood Cell Count
NEUT	-	Neutrophils
LYMPH	-	Lymphocytes
MONO	-	Monocytes
EOS	-	Eosinophils
BASO	-	Basophils
LUC	-	Large Unstained Cells
RBC	-	Red Blood Cell Count
HGB	-	Hemoglobin
HCT	-	Hematocrit
MCV	-	Mean Corpuscular Volume
MCH	-	Mean Corpuscular Hemoglobin
MCHC	-	Mean Corpuscular Hemoglobin Concentration
RDW	-	Red Blood Cell Distribution Width
PLT	-	Platelet Count
RETIC	-	Reticulocytes
WBC MORPH	-	White Blood Cell Morphology
RBCNUCLE	-	Nucleated Red Blood Cells/100 Leukocytes
POLY	-	Polychromasia
UNSC	-	Unscheduled bleed
CLOT	-	Sample Clotted
NAF	-	No Abnormal Findings
ADQ	-	Adequate

Appendix 9

Appendix 1

Manual Evaluation – Blood Cell Morphology:

Platelets:

Species	DEC	ADQ	INC
Dog	0 - 7	6 - 40	≥ 41
Monkey	0 - 13	11 - 40	≥ 41
Rat	0 - 33	26 - 100	≥ 100
Mouse	0 - 33	26 - 120	≥ 121
Rabbit	0 - 7	6 - 67	≥ 68
Pig	0 - 13	11 - 53	≥ 54
Guinea Pig	0 - 7	6 - 40	≥ 41
Cat	0 - 7	6 - 40	≥ 41
Cattle	0 - 7	6 - 53	≥ 54
Sheep	0 - 15	14 - 53	≥ 54

Platelet Morphology:

Observation	1+ (Minimal)	2+ (Mild)	3+ (Moderate)	4+ (Marked)
Platelet Clumps/slide	1-7 small clumps	8-15 small clumps or 1-3 large clumps	>15 small clumps or >3 large clumps	>10 large clumps
Large Platelets	1%-5%	6%-10%	11%-30%	>30%

WBC Morphology:

Observation	1+ (Minimal)	2+ (Mild)	3+ (Moderate)	4+ (Marked)
Immature neutrophils	1%-5%	6%-10%	11%- 30%	>30%
Immature unclassified cells	1%-5%	6% -10%	11%- 30%	>30%
Cytoplasmic vacuolation of lymphocytes	1%-5%	6%-10%	11%- 30%	>30%
Other	1%-5%	6% -10%	11%- 30%	>30%
Neutrophils band form	1%-5%	6% -10%	11% - 30%	>30%
Cytoplasmic vacuolation of neutrophils	1%-5%	6% -10%	11% - 30%	>30%
Döhle Bodies	1%-5%	6% -10%	11% - 30%	>30%
Cytoplasmic basophilia	1%-5%	6% -10%	11% - 30%	>30%

Appendix 9

Appendix 1

RBC Morphology:

Observation	1+ (Minimal)	2+ (Mild)	3+ (Moderate)	4+ (Marked)
Hypochromasia	3% - 5%	6% - 15%	16% - 30%	>30%
Spherocytes	1% - 10%	11% - 20%	21% - 50%	>50%
Keratocytes	1% - 5%	6% - 10%	11% - 20%	>20%
Heinz Bodies	1-3 cells	4-8 cells	9-20 cells	>20 cells
RBC Agglutination	2-4 clumps	5-8 clumps	9-12 clumps	>12 clumps
Eccentrocytes	1% - 5%	6% - 10%	11% - 20%	>20%
Other	1% - 5%	6% - 10%	11% - 20%	>20%
Anisocytosis	3% - 5%	6% - 15%	16% - 30%	>30%
Microcytes	3% - 5%	6% - 15%	16% - 30%	>30%
Macrocytes	3% - 5%	6% - 15%	16% - 30%	>30%
Polychromasia	3% - 5%	6% - 10%	11% - 20%	>20%
Poikilocytosis	1% - 5%	6% - 10%	11% - 20%	>20%
Echinocytes	3% - 5%	6% - 15%	16% - 30%	>30%
Acanthocytes	1% - 3%	4% - 8 %	9% - 20%	>20%
Schistocytes	1% - 5%	6% - 10%	11% - 20%	>20%
Ovalocytes	1% - 5%	6% - 10%	11% - 20%	>20%
Dacrocytes	1% - 5%	6% - 10%	11% - 20%	>20%
Codocytes	1% - 5%	6% - 10%	11% - 20%	>20%
Stomatocytes	1% - 5%	6% - 10%	11% - 20%	>20%
Howell-Jolly Bodies	1-3 cells	4-8 cells	9-20 cells	>20 cells
Basophilic Stippling	1-3 cells	4-8 cells	9-20 cells	>20 cells
Rouleaux	2-4 stacks	5-8 stacks	9-12 stacks	>12 stacks
RBC Clumping	2-4 clumps	5-8 clumps	9-12 clumps	>12 clumps
Plasmodium Species	Present in at least 3 fields	Present in at least 4-6 fields	Present in at least 7-9 fields	Present in >10 fields

Appendix 9

Appendix 1

Individual Hematology Values

2954-001

Sex: Male

Group 1	Day(s) Relative to Start Date	WBC (10^3/uL)	NEUT (10^3/uL)	LYMPH (10^3/uL)	MONO (10^3/uL)	EOS (10^3/uL)	BASO (10^3/uL)
		Reporting Hematology					
1006	87	18.22	2.09	15.19	0.45	0.22	0.17
1007	87	16.69	1.42	14.67	0.25	0.10	0.15
1008	87	12.25	1.04	10.78	0.18	0.07	0.09
1009	91	4.30	0.55	3.65	0.05	0.02	0.00
1010	91	2.77	0.61	2.06	0.07	0.01	0.01
1011	8	17.45	1.34	15.35	0.36	0.11	0.12
1012	8	8.47	0.68	7.39	0.21	0.02	0.04
1013	8	10.17	1.04	8.48	0.22	0.12	0.07
1014	8	10.79	1.21	9.06	0.30	0.04	0.07
1015	8	9.24	1.04	7.78	0.22	0.07	0.04
1021	29	17.59	2.37	14.66	0.21	0.08	0.15
1022	29	9.81	0.97	8.52	0.14	0.03	0.05
1023	29	15.40	1.26	13.45	0.26	0.07	0.13
1024	29	20.83	1.82	18.47	0.17	0.07	0.15
1025	29	10.66	0.95	9.28	0.19	0.06	0.07

Appendix 9

Appendix 1

Individual Hematology Values

2954-001

Sex: Male

Group 1	Day(s) Relative to Start Date	LUC (10^3/uL)	RBC (10^6/uL)	HGB (g/dL)	HCT (%)	MCV (fL)	MCH (pg)
		Reporting Hematology					
1006	87	0.10	9.24	16.7	56.4	61.1	18.1
1007	87	0.10	9.53	15.6	53.3	56.0	16.4
1008	87	0.08	9.00	16.0	53.2	59.2	17.8
1009	91	0.03	9.09	15.8	51.3	56.4	17.4
1010	91	0.01	8.50	14.6	46.4	54.5	17.1
1011	8	0.17	7.56	15.8	51.8	68.5	20.8
1012	8	0.13	9.07	17.3	59.4	65.5	19.1
1013	8	0.24	7.66	16.3	52.7	68.8	21.3
1014	8	0.12	8.57	17.1	57.5	67.1	20.0
1015	8	0.08	7.82	16.5	53.8	68.9	21.2
1021	29	0.11	8.37	16.7	53.6	64.1	19.9
1022	29	0.09	9.14	17.0	59.1	64.6	18.6
1023	29	0.23	9.12	16.7	58.6	64.3	18.3
1024	29	0.16	7.98	15.4	51.5	64.5	19.3
1025	29	0.11	9.08	17.2	59.0	64.9	19.0

Appendix 9

Appendix 1

Individual Hematology Values

2954-001

Sex: Male

Group 1		Reporting Hematology					
	Day(s) Relative to Start Date	MCHC (g/dL)	RDW (%)	PLT (10^3/uL)	RETIC (10^9/L)	POLY	RBCNUCLE (#/100 WBC)
1006	87	29.7	13.1	1080	182.4	-	-
1007	87	29.3	13.0	1107	240.4	-	-
1008	87	30.1	13.5	736	205.8	-	-
1009	91	30.8	12.2	819	165.3	-	-
1010	91	31.4	13.2	931	151.3	-	-
1011	8	30.4	12.6	465	375.9	2+	-
1012	8	29.2	12.1	1161	473.0	-	-
1013	8	31.0	12.3	998	398.1	-	-
1014	8	29.8	12.8	1254	476.2	-	-
1015	8	30.7	12.5	702	402.6	-	-
1021	29	31.1	11.8	879	204.3	-	-
1022	29	28.7	11.5	904	225.1	-	-
1023	29	28.4	11.2	899	192.0	-	-
1024	29	29.9	11.4	948	242.2	-	-
1025	29	29.2	12.1	1116	244.5	-	-

Appendix 9

Appendix 1

Individual Hematology Values

2954-001

Sex: Male

Group 1		Reporting Hematology	
	Day(s) Relative to Start Date	PLATELET CLUMPS	WBC MORPH
1006	87	-	-
1007	87	-	-
1008	87	-	-
1009	91	-	-
1010	91	-	-
1011	8	1+	NAF
1012	8	-	-
1013	8	-	-
1014	8	-	-
1015	8	-	-
1021	29	-	-
1022	29	-	-
1023	29	-	-
1024	29	-	-
1025	29	-	-

Appendix 9

Appendix 1

Individual Hematology Values

2954-001

Sex: Male

Group 2	Day(s) Relative to Start Date	Reporting Hematology WBC (10^3/uL)	NEUT (10^3/uL)	LYMPH (10^3/uL)	MONO (10^3/uL)	EOS (10^3/uL)	BASO (10^3/uL)
2006	87	20.71	2.19	17.51	0.45	0.16	0.19
2007	87	14.53	2.83	11.09	0.22	0.18	0.09
2008	87	14.50	1.60	12.12	0.32	0.11	0.11
2009	91	11.79	3.48	7.82	0.20	0.12	0.07
2010	91	15.46	2.30	12.31	0.47	0.07	0.17
2011	8	14.27	2.09	11.29	0.40	0.06	0.09
2012	8	14.14	1.30	12.09	0.37	0.12	0.07
2013	8	11.64	0.77	10.39	0.26	0.03	0.08
2014	8	15.96	1.57	13.31	0.55	0.06	0.11
2015	8	7.00	0.74	5.93	0.15	0.03	0.06
2021	29	9.51	1.15	7.94	0.19	0.06	0.07
2022	29	23.37	1.75	20.61	0.40	0.15	0.22
2023	29	4.28	0.66	3.47	0.05	0.04	0.02
2024	29	17.41	1.84	14.55	0.20	0.12	0.14
2025	29	9.86	1.00	8.26	0.18	0.09	0.06

Appendix 9

Appendix 1

Individual Hematology Values

2954-001

Sex: Male

Group 2		Reporting Hematology					
	Day(s) Relative to Start Date	LUC (10^3/uL)	RBC (10^6/uL)	HGB (g/dL)	HCT (%)	MCV (fL)	MCH (pg)
2006	87	0.20	9.15	16.5	54.9	60.0	18.0
2007	87	0.12	9.00	16.1	52.2	58.0	17.9
2008	87	0.24	9.09	16.4	55.2	60.7	18.0
2009	91	0.10	9.27	15.8	51.8	55.9	17.0
2010	91	0.14	9.54	16.6	54.0	56.6	17.4
2011	8	0.34	7.08	14.4	47.8	67.4	20.4
2012	8	0.19	7.48	15.1	50.4	67.4	20.2
2013	8	0.11	7.99	16.4	55.1	68.9	20.6
2014	8	0.37	8.30	17.3	57.6	69.4	20.8
2015	8	0.09	8.77	16.8	55.6	63.4	19.2
2021	29	0.11	8.70	17.2	58.5	67.2	19.8
2022	29	0.22	9.50	17.3	59.6	62.8	18.2
2023	29	0.05	8.50	15.9	53.3	62.6	18.6
2024	29	0.56	8.12	15.8	54.6	67.2	19.4
2025	29	0.26	8.86	17.4	59.7	67.4	19.7

Appendix 9

Appendix 1

Individual Hematology Values

2954-001

Sex: Male

Group 2		Reporting Hematology					
	Day(s) Relative to Start Date	MCHC (g/dL)	RDW (%)	PLT (10^3/uL)	RETIC (10^9/L)	POLY	RBCNUCLE (#/100 WBC)
2006	87	30.0	13.0	1074	192.8	-	-
2007	87	31.0	12.3	875	158.7	-	-
2008	87	29.7	12.0	1378	177.8	-	-
2009	91	30.5	13.0	864	151.0	-	-
2010	91	30.7	12.8	940	184.5	-	-
2011	8	30.2	13.8	905	485.2	-	-
2012	8	29.9	13.0	1103	451.2	-	-
2013	8	29.9	13.7	1287	553.2	-	-
2014	8	30.0	12.3	1195	452.1	-	-
2015	8	30.3	13.2	1220	540.4	-	-
2021	29	29.4	11.2	635	255.5	-	-
2022	29	29.0	11.3	1032	294.7	-	-
2023	29	29.8	11.6	746	215.5	-	-
2024	29	28.9	12.1	1030	234.5	-	-
2025	29	29.2	11.2	921	253.7	-	-

Appendix 9
Appendix 1

Individual Hematology Values

2954-001
Sex: Male

Group 2	Day(s) Relative to Start Date	Reporting Hematology PLATELET CLUMPS	Reporting Hematology WBC MORPH
2006	87	-	-
2007	87	-	-
2008	87	-	-
2009	91	-	-
2010	91	-	-
2011	8	-	-
2012	8	-	-
2013	8	-	-
2014	8	-	-
2015	8	-	-
2021	29	-	-
2022	29	-	-
2023	29	-	-
2024	29	-	-
2025	29	-	-

Appendix 9

Appendix 1

Individual Hematology Values

2954-001

Sex: Male

Group 3	Day(s) Relative to Start Date	WBC (10^3/uL)	NEUT (10^3/uL)	LYMPH (10^3/uL)	MONO (10^3/uL)	EOS (10^3/uL)	BASO (10^3/uL)
		Reporting Hematology					
3006	87	14.71	1.64	12.60	0.22	0.04	0.10
3007	87	17.32	2.19	14.50	0.26	0.11	0.13
3008	16 (Unsc)	5.18	0.30	4.62	0.06	0.08	0.04
	87	NT	NT	NT	NT	NT	NT
3009	91	20.36	2.00	17.41	0.44	0.06	0.24
3010	91	14.23	1.06	12.35	0.52	0.07	0.13
3011	8	10.88	1.24	9.06	0.26	0.06	0.06
3012	8	18.60	2.06	15.76	0.26	0.03	0.15
3013	8	15.08	1.27	12.98	0.47	0.06	0.13
3014	8	18.05	1.35	15.88	0.43	0.09	0.10
3015	8	9.55	0.72	8.50	0.16	0.03	0.05
4013	8	8.23	0.90	6.89	0.11	0.04	0.05
3021	29	16.00	1.89	13.51	0.22	0.05	0.10
3022	29	22.91	2.68	19.11	0.50	0.12	0.20
3023	29	12.60	1.38	10.59	0.25	0.10	0.11
3024	29	18.61	1.39	16.00	0.59	0.11	0.14
3025	29	18.94	1.86	16.36	0.22	0.05	0.19

Appendix 9

Appendix 1

Individual Hematology Values

2954-001

Sex: Male

Group 3		Reporting Hematology					
	Day(s) Relative to Start Date	LUC (10^3/uL)	RBC (10^6/uL)	HGB (g/dL)	HCT (%)	MCV (fL)	MCH (pg)
3006	87	0.12	9.45	16.6	54.0	57.2	17.5
3007	87	0.13	9.45	16.2	53.7	56.9	17.1
3008	16 (Unsc)	0.08	9.04	17.8	58.5	64.7	19.6
	87	NT	NT	NT	NT	NT	NT
3009	91	0.21	9.38	17.0	56.5	60.3	18.1
3010	91	0.11	8.90	15.8	51.3	57.6	17.7
3011	8	0.19	7.51	15.7	51.6	68.6	20.9
3012	8	0.34	7.77	16.8	55.9	71.9	21.6
3013	8	0.17	7.93	15.9	52.1	65.7	20.1
3014	8	0.19	7.54	16.0	54.3	72.0	21.2
3015	8	0.09	8.52	16.7	55.1	64.6	19.6
4013	8	0.24	8.10	15.9	53.1	65.5	19.7
3021	29	0.23	8.81	16.4	55.7	63.2	18.6
3022	29	0.28	8.59	16.1	55.0	64.0	18.8
3023	29	0.17	8.78	16.9	57.9	65.9	19.3
3024	29	0.38	8.07	15.3	52.6	65.2	18.9
3025	29	0.27	9.51	17.4	58.7	61.7	18.3

Appendix 9

Appendix 1

Individual Hematology Values

2954-001

Sex: Male

Group 3		Reporting Hematology					
	Day(s) Relative to Start Date	MCHC (g/dL)	RDW (%)	PLT (10^3/uL)	RETIC (10^9/L)	POLY	RBCNUCLE (#/100 WBC)
3006	87	30.6	11.9	1165	224.5	-	-
3007	87	30.1	12.8	1156	204.1	-	-
3008	16 (Unsc)	30.3	12.1	921	100.9	-	-
	87	NT	NT	NT	NT	-	-
3009	91	30.0	12.7	831	193.8	-	-
3010	91	30.8	13.8	1100	181.8	-	-
3011	8	30.4	12.5	1113	465.6	-	-
3012	8	30.1	13.6	1155	503.7	-	-
3013	8	30.6	12.8	978	385.6	-	-
3014	8	29.5	13.6	1691	469.6	-	-
3015	8	30.4	13.8	1287	519.0	-	-
4013	8	30.0	13.0	973	506.0	-	-
3021	29	29.5	12.1	857	278.3	-	-
3022	29	29.4	11.6	907	248.1	-	-
3023	29	29.2	11.2	1051	276.8	-	-
3024	29	29.0	11.5	988	202.9	-	-
3025	29	29.6	11.0	1032	287.3	-	-

Appendix 9

Appendix 1

Individual Hematology Values

2954-001

Sex: Male

Group 3		Reporting Hematology	
	Day(s) Relative to Start Date	PLATELET CLUMPS	WBC MORPH
3006	87	-	-
3007	87	-	-
3008	16 (Unsc)	-	-
	87	-	-
3009	91	-	-
3010	91	-	-
3011	8	-	-
3012	8	-	-
3013	8	-	-
3014	8	-	-
3015	8	-	-
4013	8	-	-
3021	29	-	-
3022	29	-	-
3023	29	-	-
3024	29	-	-
3025	29	-	-

Appendix 9

Appendix 1

Individual Hematology Values

2954-001

Sex: Male

Group 4		Reporting Hematology					
	Day(s) Relative to Start Date	WBC (10^3/uL)	NEUT (10^3/uL)	LYMPH (10^3/uL)	MONO (10^3/uL)	EOS (10^3/uL)	BASO (10^3/uL)
4006	87	13.80	1.26	11.81	0.41	0.11	0.12
4007	87	16.48	1.93	13.48	0.49	0.20	0.15
4008	87	17.73	1.98	14.85	0.33	0.15	0.13
4009	91	14.69	1.72	12.07	0.48	0.08	0.18
4010	91	6.39	0.81	5.32	0.13	0.02	0.05
4011	8	16.87	1.68	13.51	0.94	0.09	0.23
4012	8	16.83	1.35	14.75	0.40	0.05	0.11
4113	8	11.42	1.61	9.20	0.31	0.08	0.13
4021	8	7.51	1.30	5.83	0.14	0.11	0.06
4022	8	7.31	1.06	5.72	0.30	0.05	0.12
4023	29	11.58	0.94	10.11	0.21	0.08	0.09
4024	29	19.36	1.26	17.41	0.23	0.10	0.21
4025	29	16.59	1.13	14.27	0.53	0.25	0.18
4026	29	17.52	1.40	14.85	0.49	0.07	0.17
4027	29	17.86	1.07	16.08	0.26	0.12	0.16

Appendix 9

Appendix 1

Individual Hematology Values

2954-001

Sex: Male

Group 4		Reporting Hematology					
	Day(s) Relative to Start Date	LUC (10^3/uL)	RBC (10^6/uL)	HGB (g/dL)	HCT (%)	MCV (fL)	MCH (pg)
4006	87	0.10	9.24	16.2	52.4	56.7	17.6
4007	87	0.24	10.29	18.3	60.4	58.7	17.8
4008	87	0.28	9.48	16.7	54.7	57.7	17.6
4009	91	0.17	9.32	17.2	56.5	60.6	18.5
4010	91	0.06	9.07	16.5	53.6	59.1	18.2
4011	8	0.40	7.79	16.1	53.6	68.8	20.7
4012	8	0.17	7.72	15.2	50.1	64.9	19.7
4113	8	0.10	7.86	15.6	55.9	71.2	19.8
4021	8	0.05	8.14	16.4	59.3	72.9	20.2
4022	8	0.07	8.59	17.5	63.3	73.6	20.4
4023	29	0.15	8.51	16.0	53.8	63.2	18.8
4024	29	0.16	9.46	17.8	59.7	63.1	18.8
4025	29	0.23	9.49	17.6	60.0	63.2	18.5
4026	29	0.54	9.61	16.7	57.0	59.3	17.4
4027	29	0.17	9.90	18.0	62.6	63.2	18.1

Appendix 9

Appendix 1

Individual Hematology Values

2954-001

Sex: Male

Group 4		Reporting Hematology					
	Day(s) Relative to Start Date	MCHC (g/dL)	RDW (%)	PLT (10^3/uL)	RETIC (10^9/L)	POLY	RBCNUCLE (#/100 WBC)
4006	87	31.0	12.5	960	184.9	-	-
4007	87	30.3	11.8	872	186.5	-	-
4008	87	30.6	12.4	868	160.6	-	-
4009	91	30.5	12.9	880	186.3	-	-
4010	91	30.8	12.8	1018	162.9	-	-
4011	8	30.0	13.0	1552	478.4	-	-
4012	8	30.4	12.8	738	423.0	-	-
4113	8	27.9	14.1	796	549.7	-	-
4021	8	27.7	13.5	750	464.0	-	-
4022	8	27.7	13.8	1095	461.9	-	-
4023	29	29.8	12.1	666	232.3	-	-
4024	29	29.8	11.4	886	216.6	-	-
4025	29	29.3	11.5	976	246.5	-	-
4026	29	29.3	11.9	892	299.8	-	-
4027	29	28.7	11.1	942	223.8	-	-

Appendix 9

Appendix 1

Individual Hematology Values

2954-001

Sex: Male

Group 4	Day(s) Relative to Start Date	Reporting Hematology PLATELET CLUMPS	Reporting Hematology WBC MORPH
4006	87	-	-
4007	87	-	-
4008	87	-	-
4009	91	-	-
4010	91	-	-
4011	8	-	-
4012	8	-	-
4113	8	-	-
4021	8	-	-
4022	8	-	-
4023	29	-	-
4024	29	-	-
4025	29	-	-
4026	29	-	-
4027	29	-	-

Appendix 9

Appendix 1

Individual Hematology Values

2954-001

Sex: Female

Group 1		Reporting Hematology					
	Day(s) Relative to Start Date	WBC (10^3/uL)	NEUT (10^3/uL)	LYMPH (10^3/uL)	MONO (10^3/uL)	EOS (10^3/uL)	BASO (10^3/uL)
1506	90	7.26	0.91	6.07	0.10	0.06	0.06
1507	90	9.58	0.93	8.08	0.21	0.08	0.09
1508	90	6.20	0.33	5.53	0.12	0.07	0.04
1509	91	6.28	1.52	4.26	0.19	0.19	0.09
1510	91	4.32	0.59	3.58	0.06	0.05	0.02
1511	8	13.96	0.88	12.49	0.26	0.04	0.14
1512	8	9.51	0.66	8.41	0.21	0.04	0.12
1513	8	8.77	0.30	8.07	0.18	0.04	0.08
1514	8	10.70	0.42	9.83	0.19	0.04	0.08
1515	8	9.76	0.75	8.58	0.23	0.05	0.07
1521	29	7.40	0.55	6.46	0.09	0.14	0.07
1522	29	6.53	0.87	5.25	0.11	0.09	0.05
1523	29	3.35	0.30	2.95	0.05	0.02	0.01
1524	29	4.17	0.79	3.18	0.09	0.06	0.01
1525	29	9.04	0.82	7.40	0.26	0.10	0.07

Appendix 9

Appendix 1

Individual Hematology Values

2954-001

Sex: Female

Group 1		Reporting Hematology					
	Day(s) Relative to Start Date	LUC (10^3/uL)	RBC (10^6/uL)	HGB (g/dL)	HCT (%)	MCV (fL)	MCH (pg)
1506	90	0.07	7.81	14.2	45.3	58.0	18.2
1507	90	0.20	7.94	14.8	48.7	61.3	18.6
1508	90	0.11	8.13	14.6	49.8	61.2	18.0
1509	91	0.03	9.61	18.4	60.2	62.6	19.2
1510	91	0.02	8.59	15.2	49.3	57.4	17.7
1511	8	0.16	8.90	17.5	58.1	65.3	19.6
1512	8	0.07	8.23	16.7	53.5	65.0	20.3
1513	8	0.10	8.40	16.0	53.4	63.5	19.1
1514	8	0.15	8.07	15.9	51.8	64.2	19.7
1515	8	0.09	7.37	15.6	50.2	68.1	21.2
1521	29	0.09	9.60	17.6	60.0	62.5	18.3
1522	29	0.15	9.31	17.5	58.1	62.4	18.8
1523	29	0.04	7.64	14.1	46.6	61.1	18.4
1524	29	0.04	8.65	16.1	53.9	62.3	18.6
1525	29	0.39	9.33	16.9	57.9	62.1	18.2

Appendix 9
Appendix 1

Individual Hematology Values

2954-001
Sex: Female

Group 1	Day(s) Relative to Start Date	MCHC (g/dL)	RDW (%)	PLT (10^3/uL)	RETIC (10^9/L)	POLY	RBCNUCLE (#/100 WBC)
		Reporting Hematology					
1506	90	31.4	12.1	1014	236.8	-	-
1507	90	30.3	11.6	903	170.1	-	-
1508	90	29.3	11.1	1016	123.9	-	-
1509	91	30.7	11.1	634	225.2	-	-
1510	91	30.8	11.6	914	154.1	-	-
1511	8	30.0	11.9	1253	326.6	-	-
1512	8	31.3	11.6	1123	228.6	-	-
1513	8	30.0	11.3	1315	272.7	-	-
1514	8	30.6	10.8	1211	263.0	-	-
1515	8	31.1	11.0	1188	230.7	-	-
1521	29	29.3	10.6	1184	196.3	-	-
1522	29	30.2	10.5	991	198.9	-	-
1523	29	30.2	11.3	1032	316.9	-	-
1524	29	29.8	11.3	ADQ	264.7	2+	-
1525	29	29.3	10.8	958	259.8	-	-

Appendix 9

Appendix 1

Individual Hematology Values

2954-001

Sex: Female

Group 1	Day(s) Relative to Start Date	Reporting Hematology PLATELET CLUMPS	Reporting Hematology WBC MORPH
1506	90	-	-
1507	90	-	-
1508	90	-	-
1509	91	-	-
1510	91	-	-
1511	8	-	-
1512	8	-	-
1513	8	-	-
1514	8	-	-
1515	8	-	-
1521	29	-	-
1522	29	-	-
1523	29	-	-
1524	29	3+	NAF
1525	29	-	-

Appendix 9

Appendix 1

Individual Hematology Values

2954-001

Sex: Female

Group 2	Day(s) Relative to Start Date	Reporting Hematology WBC (10^3/uL)	NEUT (10^3/uL)	LYMPH (10^3/uL)	MONO (10^3/uL)	EOS (10^3/uL)	BASO (10^3/uL)
2506	90	15.17	0.68	13.86	0.24	0.06	0.15
2507	90	11.45	1.33	9.65	0.15	0.04	0.15
2508	90	9.30	0.74	7.84	0.32	0.07	0.11
2509	91	9.19	1.80	6.69	0.23	0.13	0.11
2510	91	5.81	0.79	4.82	0.08	0.02	0.04
2511	8	8.92	0.94	7.57	0.22	0.07	0.05
2512	8	6.91	0.59	5.97	0.11	0.09	0.05
2513	8	7.57	0.42	6.63	0.18	0.04	0.10
2514	8	CLOT	CLOT	CLOT	CLOT	CLOT	CLOT
2515	8	4.68	0.31	4.18	0.08	0.05	0.03
2521	29	14.29	0.47	13.11	0.19	0.05	0.17
2522	29	10.27	1.63	8.35	0.09	0.04	0.06
2523	29	10.42	0.79	9.19	0.15	0.03	0.08
2524	29	9.93	0.88	8.64	0.11	0.05	0.07
2525	29	12.85	0.75	11.55	0.19	0.12	0.12

Appendix 9

Appendix 1

Individual Hematology Values

2954-001

Sex: Female

Group 2	Day(s) Relative to Start Date	LUC (10^3/uL)	RBC (10^6/uL)	HGB (g/dL)	HCT (%)	MCV (fL)	MCH (pg)
		Reporting Hematology					
2506	90	0.19	8.28	15.5	49.3	59.6	18.7
2507	90	0.13	8.29	15.7	51.1	61.6	18.9
2508	90	0.22	8.25	15.9	50.8	61.6	19.3
2509	91	0.22	8.93	15.9	52.3	58.6	17.8
2510	91	0.06	9.03	16.0	52.0	57.6	17.8
2511	8	0.08	7.68	15.1	49.8	64.9	19.6
2512	8	0.11	8.13	15.8	51.0	62.7	19.4
2513	8	0.21	8.34	15.8	52.1	62.4	18.9
2514	8	CLOT	CLOT	CLOT	CLOT	CLOT	CLOT
2515	8	0.04	8.11	15.5	51.1	62.9	19.1
2521	29	0.31	8.51	15.2	52.3	61.4	17.8
2522	29	0.10	8.36	16.4	55.2	65.9	19.7
2523	29	0.17	8.29	15.3	51.2	61.8	18.4
2524	29	0.17	8.25	15.8	51.1	61.9	19.1
2525	29	0.13	9.07	16.4	55.4	61.1	18.0

Appendix 9

Appendix 1

Individual Hematology Values

2954-001

Sex: Female

Group 2	Day(s) Relative to Start Date	MCHC (g/dL)	RDW (%)	PLT (10^3/uL)	RETIC (10^9/L)	POLY	RBCNUCLE (#/100 WBC)
		Reporting Hematology					
2506	90	31.4	12.4	1055	151.6	-	-
2507	90	30.7	12.7	1086	184.5	-	-
2508	90	31.3	12.1	1190	194.7	-	-
2509	91	30.5	11.2	851	174.6	-	-
2510	91	30.9	11.0	773	123.5	-	-
2511	8	30.2	11.5	894	210.5	-	-
2512	8	31.0	11.3	989	243.9	-	-
2513	8	30.3	11.0	1124	217.5	-	-
2514	8	CLOT	CLOT	CLOT	CLOT	-	-
2515	8	30.3	11.0	1360	227.8	-	-
2521	29	29.1	10.8	996	203.6	-	-
2522	29	29.8	11.1	1219	205.7	-	-
2523	29	29.8	11.2	1313	279.5	-	-
2524	29	30.9	11.0	982	238.5	-	-
2525	29	29.5	11.3	1081	245.5	-	-

Appendix 9

Appendix 1

Individual Hematology Values

2954-001

Sex: Female

Group 2	Day(s) Relative to Start Date	Reporting Hematology PLATELET CLUMPS	Reporting Hematology WBC MORPH
2506	90	-	-
2507	90	-	-
2508	90	-	-
2509	91	-	-
2510	91	-	-
2511	8	-	-
2512	8	-	-
2513	8	-	-
2514	8	-	-
2515	8	-	-
2521	29	-	-
2522	29	-	-
2523	29	-	-
2524	29	-	-
2525	29	-	-

Appendix 9
Appendix 1

Individual Hematology Values

2954-001
Sex: Female

Group 3	Day(s) Relative to Start Date	Reporting Hematology WBC (10^3/uL)	NEUT (10^3/uL)	LYMPH (10^3/uL)	MONO (10^3/uL)	EOS (10^3/uL)	BASO (10^3/uL)
3506	91	10.06	0.91	8.83	0.14	0.05	0.05
3507	91	6.29	0.74	5.31	0.11	0.03	0.05
3508	91	11.12	1.13	9.42	0.27	0.06	0.10
3509	91	18.03	2.65	14.53	0.36	0.13	0.14
3510	91	8.62	0.68	7.51	0.24	0.05	0.08
3511	8	11.30	0.66	10.17	0.17	0.06	0.11
3512	8	11.72	0.76	10.30	0.32	0.15	0.08
3513	8	8.41	0.49	7.25	0.43	0.06	0.08
3514	8	11.86	0.43	10.78	0.26	0.14	0.11
3515	8	8.09	0.32	7.30	0.21	0.07	0.11
3521	29	CLOT	CLOT	CLOT	CLOT	CLOT	CLOT
3522	29	13.61	0.97	12.10	0.16	0.12	0.15
3523	29	5.65	1.22	4.18	0.12	0.04	0.02
3524	29	11.83	1.00	10.35	0.12	0.07	0.11
3525	29	9.69	0.55	8.79	0.10	0.04	0.08

Appendix 9

Appendix 1

Individual Hematology Values

2954-001

Sex: Female

Group 3		Reporting Hematology					
	Day(s) Relative to Start Date	LUC (10^3/uL)	RBC (10^6/uL)	HGB (g/dL)	HCT (%)	MCV (fL)	MCH (pg)
3506	91	0.09	8.48	15.7	50.4	59.5	18.5
3507	91	0.05	9.07	16.5	53.0	58.4	18.2
3508	91	0.13	8.35	15.3	49.7	59.5	18.3
3509	91	0.24	9.22	16.2	52.3	56.7	17.6
3510	91	0.05	9.11	17.3	53.7	58.9	19.0
3511	8	0.12	7.59	14.7	49.5	65.2	19.4
3512	8	0.12	8.16	16.4	55.1	67.5	20.1
3513	8	0.11	8.98	17.2	56.7	63.1	19.2
3514	8	0.14	8.89	16.8	54.3	61.1	18.9
3515	8	0.10	8.74	17.0	55.8	63.9	19.4
3521	29	CLOT	CLOT	CLOT	CLOT	CLOT	CLOT
3522	29	0.11	9.28	16.2	53.9	58.1	17.4
3523	29	0.06	7.86	15.4	52.2	66.5	19.6
3524	29	0.19	9.48	16.1	56.3	59.4	17.0
3525	29	0.12	9.67	18.0	60.5	62.6	18.6

Appendix 9

Appendix 1

Individual Hematology Values

2954-001

Sex: Female

Group 3		Reporting Hematology					
	Day(s) Relative to Start Date	MCHC (g/dL)	RDW (%)	PLT (10^3/uL)	RETIC (10^9/L)	POLY	RBCNUCLE (#/100 WBC)
3506	91	31.2	11.4	969	156.3	-	-
3507	91	31.2	11.7	976	195.6	-	-
3508	91	30.8	11.6	950	160.3	-	-
3509	91	30.9	12.7	1284	184.8	-	-
3510	91	32.2	11.3	1019	164.3	-	-
3511	8	29.7	11.3	1137	206.0	-	-
3512	8	29.8	11.0	1193	241.0	-	-
3513	8	30.3	10.8	1194	195.7	-	-
3514	8	31.0	10.8	1315	274.0	-	-
3515	8	30.4	11.1	1464	244.0	-	-
3521	29	CLOT	CLOT	CLOT	CLOT	-	-
3522	29	30.0	11.0	1174	180.2	-	-
3523	29	29.5	11.3	750	193.3	-	-
3524	29	28.7	11.1	1066	217.3	-	-
3525	29	29.7	11.1	921	255.8	-	-

Appendix 9
Appendix 1

Individual Hematology Values

2954-001
Sex: Female

Group 3		Reporting Hematology	
	Day(s) Relative to Start Date	PLATELET CLUMPS	WBC MORPH
3506	91	-	-
3507	91	-	-
3508	91	-	-
3509	91	-	-
3510	91	-	-
3511	8	-	-
3512	8	-	-
3513	8	-	-
3514	8	-	-
3515	8	-	-
3521	29	-	-
3522	29	-	-
3523	29	-	-
3524	29	-	-
3525	29	-	-

Appendix 9

Appendix 1

Individual Hematology Values

2954-001

Sex: Female

Group 4		Reporting Hematology					
	Day(s) Relative to Start Date	WBC (10^3/uL)	NEUT (10^3/uL)	LYMPH (10^3/uL)	MONO (10^3/uL)	EOS (10^3/uL)	BASO (10^3/uL)
4506	91	11.65	1.76	9.37	0.30	0.04	0.08
4507	91	9.91	0.83	8.59	0.27	0.03	0.10
4508	91	13.45	1.88	10.86	0.33	0.04	0.11
4509	91	CLOT	CLOT	CLOT	CLOT	CLOT	CLOT
4510	91	8.63	0.92	7.24	0.27	0.06	0.07
4511	8	12.81	0.57	11.37	0.22	0.03	0.43
4512	8	5.31	0.40	4.69	0.14	0.02	0.02
4513	8	4.62	0.18	4.30	0.06	0.01	0.03
4514	8	3.42	0.18	2.94	0.23	0.02	0.01
4515	8	7.75	0.72	6.49	0.31	0.05	0.08
4521	29	14.84	0.97	13.37	0.17	0.05	0.11
4522	29	13.10	0.65	11.86	0.21	0.05	0.09
4523	29	14.10	1.59	11.19	0.26	0.18	0.20
4524	29	20.64	0.90	19.13	0.19	0.06	0.16
4525	29	18.03	1.32	15.89	0.21	0.13	0.16

Appendix 9

Appendix 1

Individual Hematology Values

2954-001

Sex: Female

Group 4		Reporting Hematology					
	Day(s) Relative to Start Date	LUC (10^3/uL)	RBC (10^6/uL)	HGB (g/dL)	HCT (%)	MCV (fL)	MCH (pg)
4506	91	0.10	8.15	15.0	47.0	57.7	18.4
4507	91	0.08	9.03	16.5	53.3	59.1	18.3
4508	91	0.23	9.31	16.5	53.0	57.0	17.7
4509	91	CLOT	CLOT	CLOT	CLOT	CLOT	CLOT
4510	91	0.06	8.29	14.9	48.1	58.0	18.0
4511	8	0.19	8.27	16.5	52.8	63.8	19.9
4512	8	0.04	8.60	17.0	57.1	66.4	19.7
4513	8	0.04	7.96	15.5	49.1	61.7	19.5
4514	8	0.04	7.91	15.9	50.7	64.1	20.0
4515	8	0.10	7.83	14.8	48.5	61.9	19.0
4521	29	0.17	8.59	15.8	53.0	61.7	18.4
4522	29	0.24	8.39	16.0	55.2	65.8	19.1
4523	29	0.68	8.49	16.4	54.5	64.2	19.3
4524	29	0.19	9.59	17.3	56.8	59.2	18.0
4525	29	0.33	8.74	16.9	55.9	63.9	19.4

Appendix 9

Appendix 1

Individual Hematology Values

2954-001

Sex: Female

Group 4		Reporting Hematology					
	Day(s) Relative to Start Date	MCHC (g/dL)	RDW (%)	PLT (10^3/uL)	RETIC (10^9/L)	POLY	RBCNUCLE (#/100 WBC)
4506	91	31.8	11.9	1011	165.8	-	-
4507	91	31.0	11.3	1080	114.9	-	-
4508	91	31.1	11.3	1159	137.1	-	-
4509	91	CLOT	CLOT	CLOT	CLOT	-	-
4510	91	31.1	11.7	960	141.6	-	-
4511	8	31.2	11.0	906	234.0	1+	0
4512	8	29.7	11.3	1143	248.4	-	-
4513	8	31.6	10.6	1060	153.2	-	-
4514	8	31.3	11.0	897	145.0	-	-
4515	8	30.6	11.4	1296	225.0	-	-
4521	29	29.9	11.4	1005	227.3	-	-
4522	29	29.0	11.0	941	271.5	-	-
4523	29	30.0	11.1	938	170.0	-	-
4524	29	30.4	11.2	808	277.4	-	-
4525	29	30.3	10.7	805	273.7	-	-

Appendix 9

Appendix 1

Individual Hematology Values

2954-001

Sex: Female

Group 4		Reporting Hematology	
	Day(s) Relative to Start Date	PLATELET CLUMPS	WBC MORPH
4506	91	-	-
4507	91	-	-
4508	91	-	-
4509	91	-	-
4510	91	-	-
4511	8	2+	NAF
4512	8	-	-
4513	8	-	-
4514	8	-	-
4515	8	-	-
4521	29	-	-
4522	29	-	-
4523	29	-	-
4524	29	-	-
4525	29	-	-

Appendix 9

Appendix 2

Codes for Individual Coagulation Values

APTT	-	Activated Partial Thromboplastin Time
FIB	-	Fibrinogen
PT	-	Prothrombin Time
SAMQ	-	Sample Quality Coagulation
UNSC	-	Unscheduled bleed
CLOT	-	Sample Clotted

Sample Quality

N	-	Normal
H	-	Hemolyzed
+	-	Slight (pale/light red)
++	-	Moderate (red)
+++	-	Severe (dark red)

Appendix 9

Appendix 2

Individual Coagulation Values

2954-001

Sex: Male

Group 1		Reporting Coagulation			
	Day(s) Relative to Start Date	PT (sec)	APTT (sec)	FIB (mg/dL)	SAMQ Coagulation
1006	87	17.6	12.0	365	N
1007	87	16.6	13.4	312	H+
1008	87	16.2	13.6	282	H++
1009	91	UTD	UTD	UTD	UTD
1010	91	15.9	14.5	335	H+
1011	8	CLOT	CLOT	CLOT	CLOT
1012	8	17.8	11.1	313	N
1013	8	17.3	8.7	321	N
1014	8	17.4	12.0	330	N
1015	8	CLOT	CLOT	CLOT	CLOT
1021	29	15.9	15.4	261	H+
1022	29	16.2	14.7	308	N
1023	29	16.6	12.3	338	N
1024	29	15.9	13.5	302	N
1025	29	16.3	12.5	247	N

Appendix 9

Appendix 2

Individual Coagulation Values

2954-001

Sex: Male

Group 2	Day(s) Relative to Start Date	Reporting Coagulation PT (sec)	APTT (sec)	FIB (mg/dL)	SAMQ Coagulation
2006	87	17.1	13.1	320	N
2007	87	16.8	13.1	337	N
2008	87	17.1	14.2	354	N
2009	91	17.0	14.9	365	N
2010	91	16.6	17.2	357	H+++
2011	8	17.7	10.6	321	N
2012	8	15.7	9.9	292	H+
2013	8	16.4	8.9	311	N
2014	8	17.4	10.2	309	H+
2015	8	16.7	13.2	276	N
2021	29	15.2	12.6	286	N
2022	29	16.2	11.8	253	N
2023	29	17.0	13.6	312	N
2024	29	15.6	13.6	375	N
2025	29	15.4	11.7	261	H+

Appendix 9

Appendix 2

Individual Coagulation Values

2954-001

Sex: Male

Group 3	Day(s) Relative to Start Date	Reporting Coagulation PT (sec)	APTT (sec)	FIB (mg/dL)	SAMQ Coagulation
3006	87	17.8	13.3	324	N
3007	87	16.7	11.4	349	N
3008	16 (Unsc)	16.7	9.3	329	H+
	87	NT	NT	NT	NT
3009	91	17.2	15.3	362	N
3010	91	15.5	12.6	342	N
3011	8	17.1	12.7	282	N
3012	8	17.5	10.7	332	N
3013	8	17.9	10.1	287	N
3014	8	18.3	13.4	307	N
3015	8	18.3	10.8	323	N
4013	8	17.8	10.5	303	N
3021	29	15.7	11.4	278	N
3022	29	16.7	13.3	250	N
3023	29	14.7	11.0	310	H+
3024	29	17.9	14.3	308	N
3025	29	15.4	12.0	291	H+

Appendix 9

Appendix 2

Individual Coagulation Values

2954-001

Sex: Male

Group 4	Day(s) Relative to Start Date	PT (sec)	APTT (sec)	FIB (mg/dL)	SAMQ Coagulation
4006	87	16.8	14.5	301	N
4007	87	16.4	12.6	290	H+
4008	87	17.2	13.9	285	H+
4009	91	17.0	13.0	417	H+
4010	91	16.4	13.0	391	N
4011	8	16.9	11.6	261	N
4012	8	16.7	12.3	305	N
4113	8	16.9	12.5	338	N
4021	8	16.8	14.1	168	H++
4022	8	17.7	13.6	334	H+
4023	29	15.0	11.4	293	H+
4024	29	15.2	13.2	249	N
4025	29	15.2	14.0	284	N
4026	29	15.0	13.9	320	N
4027	29	14.7	13.9	310	N

Appendix 9

Appendix 2

Individual Coagulation Values

2954-001

Sex: Female

Group 1	Day(s) Relative to Start Date	PT (sec)	APTT (sec)	FIB (mg/dL)	SAMQ Coagulation
		Reporting Coagulation			
1506	90	16.6	12.6	257	H+
1507	90	17.4	12.1	299	N
1508	90	18.5	13.3	256	N
1509	91	16.7	12.3	222	H++
1510	91	16.3	13.4	193	H++
1511	8	15.9	14.8	270	H+
1512	8	15.0	13.1	300	N
1513	8	16.8	13.0	315	N
1514	8	16.4	12.6	296	N
1515	8	16.1	11.5	301	N
1521	29	15.0	13.2	210	H++
1522	29	14.3	10.6	250	H++
1523	29	15.5	15.2	216	H+++
1524	29	16.1	10.8	219	H+
1525	29	16.6	11.4	210	N

Appendix 9

Appendix 2

Individual Coagulation Values

2954-001

Sex: Female

Group 2	Day(s) Relative to Start Date	Reporting Coagulation PT (sec)	APTT (sec)	FIB (mg/dL)	SAMQ Coagulation
2506	90	17.1	13.4	251	H++
2507	90	15.6	12.1	239	H+++
2508	90	15.6	8.4	301	H++
2509	91	16.1	9.4	221	H+
2510	91	16.7	9.2	189	H+
2511	8	14.8	10.8	282	H++
2512	8	14.8	10.7	284	H+
2513	8	15.2	10.6	274	N
2514	8	CLOT	CLOT	CLOT	CLOT
2515	8	15.2	8.2	303	N
2521	29	15.1	10.9	202	H+
2522	29	16.0	14.7	213	N
2523	29	15.7	13.8	242	H++
2524	29	16.3	11.9	229	H+
2525	29	15.9	11.5	236	H+

Appendix 9

Appendix 2

Individual Coagulation Values

2954-001

Sex: Female

Group 3	Day(s) Relative to Start Date	Reporting Coagulation PT (sec)	APTT (sec)	FIB (mg/dL)	SAMQ Coagulation
3506	91	17.5	12.1	248	N
3507	91	17.5	14.0	213	N
3508	91	16.7	12.2	231	H+
3509	91	16.3	11.7	285	H+
3510	91	15.8	11.0	234	H+
3511	8	15.7	10.0	279	N
3512	8	15.5	14.9	284	N
3513	8	15.3	13.3	294	N
3514	8	16.1	11.5	301	N
3515	8	15.9	9.8	255	N
3521	29	CLOT	CLOT	CLOT	CLOT
3522	29	CLOT	CLOT	CLOT	CLOT
3523	29	15.6	8.5	194	N
3524	29	16.1	12.3	250	H++
3525	29	14.5	14.6	204	N

Appendix 9
Appendix 2

Individual Coagulation Values

2954-001
Sex: Female

Group 4	Day(s) Relative to Start Date	PT (sec)	APTT (sec)	FIB (mg/dL)	SAMQ Coagulation
4506	91	16.1	11.5	280	H+
4507	91	15.9	12.7	222	N
4508	91	14.7	9.2	216	H++
4509	91	CLOT	CLOT	CLOT	CLOT
4510	91	17.1	13.1	265	N
4511	8	14.6	11.7	273	H+
4512	8	14.6	12.1	261	N
4513	8	16.2	14.8	291	N
4514	8	15.6	13.6	268	N
4515	8	16.6	12.9	281	N
4521	29	17.3	12.6	238	N
4522	29	15.9	13.6	227	H+
4523	29	CLOT	CLOT	CLOT	CLOT
4524	29	15.1	12.4	249	H+
4525	29	15.3	12.1	224	H++

Appendix 9

Appendix 3

Codes for Individual Clinical Chemistry Values

AST	-	Aspartate Aminotransferase
ALT	-	Alanine Aminotransferase
ALP	-	Alkaline Phosphatase
TBIL	-	Total Bilirubin
UREAN	-	Urea Nitrogen
CREAT	-	Creatinine
GLUC	-	Glucose
CHOL	-	Cholesterol
TRIG	-	Triglycerides
TPROT	-	Total Protein
ALB	-	Albumin
GLOB	-	Globulin
A/G	-	Albumin/Globulin Ratio
CA	-	Calcium
PHOS	-	Phosphorus
NA	-	Sodium
K	-	Potassium
CL	-	Chloride
UNSC	-	Unscheduled bleed
NT	-	Not Taken

Hemolysis Indice

N	-	Normal
+	-	Slight (pale/light red)
++	-	Moderate (red)
+++	-	Severe (dark red)

Lipemia Indice

N - Normal

Icterus Indice

N - Normal

Appendix 9

Appendix 3

Individual Clinical Chemistry Values

2954-001

Sex: Male

Group 1	Day(s) Relative to Start Date	AST (U/L)	ALT (U/L)	ALP (U/L)	TBIL (mg/dL)	UREAN (mg/dL)	CREAT (mg/dL)
		Reporting Biochemistry					
1006	87	112	44	149	0.16	19	0.5
1007	87	89	32	85	0.16	14	0.5
1008	87	113	49	88	0.14	16	0.5
1009	91	89	46	109	0.17	17	0.5
1010	91	99	44	87	0.14	14	0.5
1011	8	193	53	335	0.11	17	0.4
1012	8	91	42	291	0.12	11	0.4
1013	8	87	37	270	0.11	12	0.3
1014	8	67	24	255	0.11	14	0.4
1015	8	172	48	247	0.11	15	0.3
1021	29	89	37	183	0.13	13	0.3
1022	29	80	43	138	0.12	13	0.5
1023	29	100	68	247	0.11	13	0.4
1024	29	75	54	194	0.10	16	0.4
1025	29	92	39	138	0.14	15	0.4

Appendix 9

Appendix 3

Individual Clinical Chemistry Values

2954-001

Sex: Male

Group 1		Reporting Biochemistry					
	Day(s) Relative to Start Date	GLUC (mg/dL)	CHOL (mg/dL)	TRIG (mg/dL)	TPROT (g/dL)	ALB (g/dL)	GLOB (g/dL)
1006	87	307	66	59	6.7	3.5	3.2
1007	87	427	80	70	7.0	3.7	3.3
1008	87	259	57	98	6.4	3.6	2.8
1009	91	151	93	63	6.9	3.5	3.4
1010	91	133	64	59	6.4	3.4	3.0
1011	8	234	70	73	6.1	3.6	2.5
1012	8	87	60	67	6.4	3.6	2.8
1013	8	110	70	84	5.7	3.3	2.4
1014	8	131	70	67	6.3	3.6	2.7
1015	8	75	78	130	6.0	3.4	2.6
1021	29	140	50	42	6.0	3.2	2.8
1022	29	414	62	70	7.8	3.8	4.0
1023	29	454	63	73	6.8	3.4	3.4
1024	29	358	47	47	6.6	3.5	3.1
1025	29	92	54	34	6.7	3.4	3.3

Appendix 9

Appendix 3

Individual Clinical Chemistry Values

2954-001

Sex: Male

Group 1	Day(s) Relative to Start Date	A/G (ratio)	CA (mg/dL)	PHOS (mg/dL)	NA (mEq/L)	K (mEq/L)	CL (mEq/L)
		Reporting Biochemistry					
1006	87	1.1	13.1	14.1	144	12.6	100
1007	87	1.1	13.5	13.0	142	12.4	98
1008	87	1.3	12.3	11.1	146	9.9	100
1009	91	1.0	11.6	10.0	147	7.4	103
1010	91	1.1	11.1	10.4	149	7.2	103
1011	8	1.4	14.0	16.6	143	11.8	101
1012	8	1.3	13.9	18.3	144	13.0	100
1013	8	1.4	12.4	15.1	148	8.6	103
1014	8	1.3	13.9	17.3	144	11.2	99
1015	8	1.3	12.5	16.2	148	9.9	99
1021	29	1.1	11.3	13.4	150	9.7	103
1022	29	1.0	13.9	15.1	148	10.4	100
1023	29	1.0	14.2	14.8	145	9.7	100
1024	29	1.1	12.7	12.1	150	8.6	99
1025	29	1.0	12.0	14.1	146	11.4	104

Appendix 9

Appendix 3

Individual Clinical Chemistry Values

2954-001

Sex: Male

Group 1	Day(s) Relative to Start Date	Hemolysis Indice	Icterus Indice	Lipemia Indice
1006	87	++	N	N
1007	87	+	N	N
1008	87	+	N	N
1009	91	+	N	N
1010	91	N	N	N
1011	8	N	N	N
1012	8	N	N	N
1013	8	N	N	N
1014	8	N	N	N
1015	8	N	N	N
1021	29	+	N	N
1022	29	N	N	N
1023	29	N	N	N
1024	29	N	N	N
1025	29	N	N	N

Appendix 9

Appendix 3

Individual Clinical Chemistry Values

2954-001

Sex: Male

Group 2	Day(s) Relative to Start Date	Reporting Biochemistry AST (U/L)	ALT (U/L)	ALP (U/L)	TBIL (mg/dL)	UREAN (mg/dL)	CREAT (mg/dL)
2006	87	67	33	124	0.12	19	0.6
2007	87	79	40	80	0.17	16	0.4
2008	87	68	28	117	0.17	18	0.6
2009	91	84	32	97	0.14	14	0.5
2010	91	106	39	119	0.16	16	0.4
2011	8	102	37	357	0.12	15	0.3
2012	8	87	29	271	0.12	12	0.3
2013	8	80	27	273	0.09	13	0.3
2014	8	68	24	187	0.10	17	0.3
2015	8	108	30	281	0.10	12	0.3
2021	29	165	42	241	0.12	15	0.4
2022	29	190	85	190	0.15	17	0.4
2023	29	124	73	199	0.12	14	0.4
2024	29	90	60	248	0.15	16	0.3
2025	29	135	49	178	0.13	16	0.4

Appendix 9

Appendix 3

Individual Clinical Chemistry Values

2954-001

Sex: Male

Group 2		Reporting Biochemistry					
	Day(s) Relative to Start Date	GLUC (mg/dL)	CHOL (mg/dL)	TRIG (mg/dL)	TPROT (g/dL)	ALB (g/dL)	GLOB (g/dL)
2006	87	467	65	154	6.9	3.6	3.3
2007	87	203	74	51	6.6	3.5	3.1
2008	87	356	74	78	7.0	3.7	3.3
2009	91	136	71	77	7.0	3.5	3.5
2010	91	318	97	71	7.4	3.6	3.8
2011	8	82	64	53	5.8	3.3	2.5
2012	8	83	73	78	5.5	3.2	2.3
2013	8	145	85	88	6.1	3.5	2.6
2014	8	205	81	70	6.3	3.5	2.8
2015	8	94	65	82	6.1	3.5	2.6
2021	29	229	65	53	6.9	3.5	3.4
2022	29	334	67	50	7.0	3.7	3.3
2023	29	218	67	67	6.4	3.4	3.0
2024	29	388	108	56	6.5	3.0	3.5
2025	29	337	84	102	6.9	3.4	3.5

Appendix 9

Appendix 3

Individual Clinical Chemistry Values

2954-001

Sex: Male

Group 2	Day(s) Relative to Start Date	A/G (ratio)	CA (mg/dL)	PHOS (mg/dL)	NA (mEq/L)	K (mEq/L)	CL (mEq/L)
		Reporting Biochemistry					
2006	87	1.1	13.2	9.2	144	8.3	100
2007	87	1.1	12.2	11.9	148	7.7	100
2008	87	1.1	13.3	11.5	143	10.8	99
2009	91	1.0	12.4	11.7	149	9.4	104
2010	91	0.9	12.6	12.0	147	10.2	100
2011	8	1.3	12.1	15.4	148	9.4	107
2012	8	1.4	12.0	14.4	146	8.7	105
2013	8	1.3	13.6	15.7	147	9.8	105
2014	8	1.3	14.0	14.1	146	7.7	101
2015	8	1.3	13.3	16.7	148	11.6	101
2021	29	1.0	12.8	13.1	148	8.4	104
2022	29	1.1	13.3	14.4	144	10.2	96
2023	29	1.1	12.4	11.6	150	7.6	103
2024	29	0.9	13.3	12.5	145	8.5	98
2025	29	1.0	13.1	12.8	148	8.5	101

Appendix 9

Appendix 3

Individual Clinical Chemistry Values

2954-001

Sex: Male

Group 2	Day(s) Relative to Start Date	Hemolysis Indice	Icterus Indice	Lipemia Indice
2006	87	N	N	N
2007	87	+	N	N
2008	87	N	N	N
2009	91	N	N	N
2010	91	+	N	N
2011	8	N	N	N
2012	8	N	N	N
2013	8	N	N	N
2014	8	N	N	N
2015	8	N	N	N
2021	29	N	N	N
2022	29	N	N	N
2023	29	N	N	N
2024	29	+	N	N
2025	29	N	N	N

Appendix 9

Appendix 3

Individual Clinical Chemistry Values

2954-001

Sex: Male

Group 3	Day(s) Relative to Start Date	AST (U/L)	ALT (U/L)	ALP (U/L)	TBIL (mg/dL)	UREAN (mg/dL)	CREAT (mg/dL)
		Reporting Biochemistry					
3006	87	101	56	96	0.13	22	0.4
3007	87	104	37	119	0.11	16	0.6
3008	16 (Unsc)	99	69	208	0.13	18	0.4
	87	NT	NT	NT	NT	NT	NT
3009	91	101	41	75	0.19	16	0.5
3010	91	82	27	67	0.20	16	0.4
3011	8	71	27	202	0.09	15	0.4
3012	8	77	39	187	0.10	17	0.4
3013	8	83	36	284	0.10	9	0.3
3014	8	77	29	268	0.10	11	0.3
3015	8	77	29	309	0.13	13	0.3
4013	8	88	30	236	0.11	14	0.3
3021	29	101	55	239	0.12	21	0.5
3022	29	67	32	159	0.11	22	0.3
3023	29	138	41	205	0.14	13	0.4
3024	29	75	39	229	0.13	14	0.3
3025	29	123	48	227	0.15	15	0.3

Appendix 9

Appendix 3

Individual Clinical Chemistry Values

2954-001

Sex: Male

Group 3	Day(s) Relative to Start Date	GLUC (mg/dL)	CHOL (mg/dL)	TRIG (mg/dL)	TPROT (g/dL)	ALB (g/dL)	GLOB (g/dL)
		Reporting Biochemistry					
3006	87	318	68	56	7.1	3.6	3.5
3007	87	402	65	94	7.1	3.7	3.4
3008	16 (Unsc)	492	78	123	7.3	3.8	3.5
	87	NT	NT	NT	NT	NT	NT
3009	91	336	76	67	7.6	3.7	3.9
3010	91	221	110	71	7.2	3.7	3.5
3011	8	160	71	74	5.7	3.3	2.4
3012	8	331	82	96	6.6	3.7	2.9
3013	8	69	75	93	5.8	3.4	2.4
3014	8	116	61	51	5.9	3.4	2.5
3015	8	133	90	86	6.2	3.5	2.7
4013	8	96	64	75	5.7	3.3	2.4
3021	29	367	73	62	7.0	3.7	3.3
3022	29	342	40	46	6.2	3.2	3.0
3023	29	376	61	67	6.9	3.6	3.3
3024	29	333	79	96	6.6	3.4	3.2
3025	29	102	78	41	6.8	3.6	3.2

Appendix 9

Appendix 3

Individual Clinical Chemistry Values

2954-001

Sex: Male

Group 3		Reporting Biochemistry					
	Day(s) Relative to Start Date	A/G (ratio)	CA (mg/dL)	PHOS (mg/dL)	NA (mEq/L)	K (mEq/L)	CL (mEq/L)
3006	87	1.0	12.7	12.4	144	9.8	100
3007	87	1.1	13.4	11.7	143	12.0	100
3008	16 (Unsc)	1.1	13.8	12.9	142	9.6	101
	87	NT	NT	NT	NT	NT	NT
3009	91	0.9	12.7	13.1	145	10.6	100
3010	91	1.1	12.4	12.0	147	9.5	100
3011	8	1.4	13.0	16.8	144	11.6	100
3012	8	1.3	13.8	13.6	146	9.3	99
3013	8	1.4	12.6	13.7	145	9.6	101
3014	8	1.4	12.6	17.5	147	11.6	105
3015	8	1.3	13.2	17.3	146	9.4	98
4013	8	1.4	12.2	14.3	147	8.5	104
3021	29	1.1	13.1	14.3	146	10.6	103
3022	29	1.1	12.3	13.0	145	9.1	98
3023	29	1.1	13.4	13.2	146	8.4	98
3024	29	1.1	13.3	11.8	145	9.0	100
3025	29	1.1	12.1	14.6	141	12.8	100

Appendix 9

Appendix 3

Individual Clinical Chemistry Values

2954-001

Sex: Male

Group 3	Day(s) Relative to Start Date	Hemolysis Indice	Icterus Indice	Lipemia Indice
3006	87	N	N	N
3007	87	N	N	N
3008	16 (Unsc)	+	N	N
	87	NT	NT	NT
3009	91	++	N	N
3010	91	++	N	N
3011	8	N	N	N
3012	8	N	N	N
3013	8	N	N	N
3014	8	N	N	N
3015	8	N	N	N
4013	8	N	N	N
3021	29	N	N	N
3022	29	N	N	N
3023	29	+	N	N
3024	29	N	N	N
3025	29	N	N	N

Appendix 9

Appendix 3

Individual Clinical Chemistry Values

2954-001

Sex: Male

Group 4	Day(s) Relative to Start Date	AST (U/L)	ALT (U/L)	ALP (U/L)	TBIL (mg/dL)	UREAN (mg/dL)	CREAT (mg/dL)
		Reporting Biochemistry					
4006	87	76	30	78	0.10	17	0.4
4007	87	110	41	97	0.10	17	0.5
4008	87	92	53	131	0.14	17	0.5
4009	91	203	70	106	0.17	17	0.5
4010	91	139	50	110	0.17	16	0.5
4011	8	170	76	320	0.13	15	0.4
4012	8	123	27	201	0.10	14	0.3
4113	8	84	41	446	0.15	15	0.3
4021	8	187	53	230	0.12	21	0.4
4022	8	143	54	433	0.16	18	0.4
4023	29	159	35	145	0.17	16	0.3
4024	29	88	36	201	0.15	14	0.4
4025	29	101	40	112	0.12	16	0.3
4026	29	80	43	181	0.15	18	0.4
4027	29	90	42	189	0.15	16	0.4

Appendix 9

Appendix 3

Individual Clinical Chemistry Values

2954-001

Sex: Male

Group 4	Day(s) Relative to Start Date	GLUC (mg/dL)	CHOL (mg/dL)	TRIG (mg/dL)	TPROT (g/dL)	ALB (g/dL)	GLOB (g/dL)
		Reporting Biochemistry					
4006	87	249	41	81	6.3	3.5	2.8
4007	87	477	64	96	6.1	3.4	2.7
4008	87	325	79	75	6.9	3.6	3.3
4009	91	128	64	70	6.9	3.4	3.5
4010	91	221	95	55	7.2	3.6	3.6
4011	8	162	59	64	6.2	3.5	2.7
4012	8	215	90	76	5.9	3.3	2.6
4113	8	118	81	42	6.2	3.5	2.7
4021	8	116	51	50	6.4	3.5	2.9
4022	8	102	64	45	6.4	3.6	2.8
4023	29	108	54	52	6.7	3.3	3.4
4024	29	231	81	57	6.7	3.5	3.2
4025	29	375	45	66	6.7	3.4	3.3
4026	29	262	58	81	7.2	3.6	3.6
4027	29	248	79	60	7.6	3.8	3.8

Appendix 9

Appendix 3

Individual Clinical Chemistry Values

2954-001

Sex: Male

Group 4	Day(s) Relative to Start Date	A/G (ratio)	CA (mg/dL)	PHOS (mg/dL)	NA (mEq/L)	K (mEq/L)	CL (mEq/L)
		Reporting Biochemistry					
4006	87	1.3	12.1	10.3	148	7.3	101
4007	87	1.3	13.7	10.8	144	9.8	101
4008	87	1.1	12.7	10.4	145	8.9	101
4009	91	1.0	12.4	16.9	139	17.0	100
4010	91	1.0	12.3	11.9	146	9.2	101
4011	8	1.3	13.1	17.0	140	14.8	103
4012	8	1.3	13.1	13.9	146	8.3	101
4113	8	1.3	12.3	13.5	145	8.3	102
4021	8	1.2	11.6	13.8	147	8.2	103
4022	8	1.3	12.9	17.3	142	12.0	100
4023	29	1.0	11.7	13.5	149	7.3	101
4024	29	1.1	13.3	15.3	144	11.8	101
4025	29	1.0	14.6	14.6	146	11.0	101
4026	29	1.0	14.2	15.5	142	12.4	98
4027	29	1.0	14.0	14.9	147	10.0	97

Appendix 9

Appendix 3

Individual Clinical Chemistry Values

2954-001

Sex: Male

Group 4	Day(s) Relative to Start Date	Hemolysis Indice	Icterus Indice	Lipemia Indice
4006	87	N	N	N
4007	87	N	N	N
4008	87	N	N	N
4009	91	N	N	N
4010	91	+	N	N
4011	8	+	N	N
4012	8	N	N	N
4113	8	N	N	N
4021	8	N	N	N
4022	8	N	N	N
4023	29	+	N	N
4024	29	+	N	N
4025	29	N	N	N
4026	29	N	N	N
4027	29	+	N	N

Appendix 9

Appendix 3

Individual Clinical Chemistry Values

2954-001

Sex: Female

Group 1		Reporting Biochemistry					
	Day(s) Relative to Start Date	AST (U/L)	ALT (U/L)	ALP (U/L)	TBIL (mg/dL)	UREAN (mg/dL)	CREAT (mg/dL)
1506	90	107	51	80	0.14	17	0.6
1507	90	105	81	234	0.15	19	0.5
1508	90	96	54	102	0.12	17	0.6
1509	91	111	44	67	0.16	21	0.7
1510	91	104	63	41	0.16	22	0.7
1511	8	235	111	237	0.13	20	0.4
1512	8	84	34	116	0.11	11	0.3
1513	8	78	47	217	0.08	18	0.4
1514	8	78	24	208	0.09	14	0.4
1515	8	64	29	138	0.11	16	0.5
1521	29	895	475	107	0.15	20	0.5
1522	29	2036	994	92	0.15	18	0.5
1523	29	78	36	133	0.12	14	0.4
1524	29	193	85	136	0.14	17	0.5
1525	29	84	41	138	0.18	19	0.4

Appendix 9

Appendix 3

Individual Clinical Chemistry Values

2954-001

Sex: Female

Group 1		Reporting Biochemistry					
	Day(s) Relative to Start Date	GLUC (mg/dL)	CHOL (mg/dL)	TRIG (mg/dL)	TPROT (g/dL)	ALB (g/dL)	GLOB (g/dL)
1506	90	313	80	112	8.5	4.5	4.0
1507	90	342	134	182	9.1	4.8	4.3
1508	90	438	70	161	8.3	4.4	3.9
1509	91	118	89	105	8.1	4.3	3.8
1510	91	138	67	66	8.2	4.6	3.6
1511	8	167	61	62	6.8	4.0	2.8
1512	8	134	69	47	6.8	4.0	2.8
1513	8	401	92	52	6.0	3.6	2.4
1514	8	356	57	31	6.3	3.7	2.6
1515	8	441	74	47	6.9	4.2	2.7
1521	29	322	54	57	8.2	4.4	3.8
1522	29	163	74	62	7.3	3.9	3.4
1523	29	194	82	65	6.8	3.9	2.9
1524	29	188	66	72	7.5	4.2	3.3
1525	29	165	80	52	6.9	3.7	3.2

Appendix 9

Appendix 3

Individual Clinical Chemistry Values

2954-001

Sex: Female

Group 1	Day(s) Relative to Start Date	A/G (ratio)	CA (mg/dL)	PHOS (mg/dL)	NA (mEq/L)	K (mEq/L)	CL (mEq/L)
		Reporting Biochemistry					
1506	90	1.1	12.8	6.6	147	6.6	101
1507	90	1.1	14.7	8.9	144	9.2	99
1508	90	1.1	14.9	14.5	144	8.0	99
1509	91	1.1	14.1	17.9	140	15.8	100
1510	91	1.3	12.2	9.0	146	8.5	103
1511	8	1.4	13.4	17.1	141	12.6	100
1512	8	1.4	12.2	13.1	139	11.6	101
1513	8	1.5	13.7	16.0	144	10.4	103
1514	8	1.4	12.9	13.5	141	10.4	104
1515	8	1.6	14.4	14.9	141	9.3	98
1521	29	1.2	13.7	16.1	137	15.2	98
1522	29	1.1	11.4	15.6	143	10.8	103
1523	29	1.3	11.9	10.1	147	5.8	100
1524	29	1.3	13.5	14.1	141	13.6	101
1525	29	1.2	13.7	14.4	142	11.6	101

Appendix 9

Appendix 3

Individual Clinical Chemistry Values

2954-001

Sex: Female

Group 1	Day(s) Relative to Start Date	Hemolysis Indice	Icterus Indice	Lipemia Indice
1506	90	N	N	N
1507	90	N	N	N
1508	90	N	N	N
1509	91	N	N	N
1510	91	N	N	N
1511	8	N	N	N
1512	8	N	N	N
1513	8	N	N	N
1514	8	N	N	N
1515	8	N	N	N
1521	29	+	N	N
1522	29	++	N	N
1523	29	N	N	N
1524	29	+	N	N
1525	29	N	N	N

Appendix 9

Appendix 3

Individual Clinical Chemistry Values

2954-001

Sex: Female

Group 2	Day(s) Relative to Start Date	AST (U/L)	ALT (U/L)	ALP (U/L)	TBIL (mg/dL)	UREAN (mg/dL)	CREAT (mg/dL)
		Reporting Biochemistry					
2506	90	82	58	119	0.13	17	0.5
2507	90	109	48	85	0.15	17	0.7
2508	90	164	71	101	0.14	18	0.6
2509	91	144	48	49	0.17	17	0.5
2510	91	106	45	82	0.14	21	0.6
2511	8	82	32	180	0.09	21	0.4
2512	8	66	37	104	0.11	16	0.4
2513	8	85	31	154	0.11	21	0.5
2514	8	101	50	178	0.12	21	0.4
2515	8	125	37	231	0.07	17	0.5
2521	29	141	56	96	0.15	14	0.4
2522	29	160	90	80	0.15	17	0.5
2523	29	96	38	100	0.12	16	0.4
2524	29	114	39	98	0.14	19	0.3
2525	29	81	34	68	0.15	12	0.4

Appendix 9

Appendix 3

Individual Clinical Chemistry Values

2954-001

Sex: Female

Group 2	Day(s) Relative to Start Date	Reporting Biochemistry GLUC (mg/dL)	CHOL (mg/dL)	TRIG (mg/dL)	TPROT (g/dL)	ALB (g/dL)	GLOB (g/dL)
2506	90	368	90	183	7.5	3.9	3.6
2507	90	394	83	130	8.6	4.6	4.0
2508	90	387	96	136	8.3	4.5	3.8
2509	91	143	92	37	8.6	4.7	3.9
2510	91	154	69	63	7.8	4.1	3.7
2511	8	426	87	44	6.3	3.6	2.7
2512	8	254	61	62	6.4	3.8	2.6
2513	8	359	91	40	6.8	3.9	2.9
2514	8	313	63	42	6.5	3.8	2.7
2515	8	307	45	35	6.1	3.6	2.5
2521	29	307	85	68	7.7	4.0	3.7
2522	29	136	43	73	7.7	4.1	3.6
2523	29	274	55	47	6.5	3.6	2.9
2524	29	107	52	35	6.6	3.6	3.0
2525	29	152	58	33	7.5	4.0	3.5

Appendix 9

Appendix 3

Individual Clinical Chemistry Values

2954-001

Sex: Female

Group 2	Day(s) Relative to Start Date	A/G (ratio)	CA (mg/dL)	PHOS (mg/dL)	NA (mEq/L)	K (mEq/L)	CL (mEq/L)
		Reporting Biochemistry					
2506	90	1.1	13.3	10.0	145	7.5	100
2507	90	1.2	13.9	12.6	146	9.6	99
2508	90	1.2	14.5	10.1	143	8.9	98
2509	91	1.2	12.6	9.8	148	7.2	102
2510	91	1.1	12.7	13.0	140	13.4	102
2511	8	1.3	13.6	14.7	138	12.2	102
2512	8	1.5	13.4	14.3	141	10.6	102
2513	8	1.3	13.5	13.0	144	9.4	102
2514	8	1.4	13.3	14.7	141	10.6	102
2515	8	1.4	13.0	15.4	145	10.4	105
2521	29	1.1	13.7	13.0	143	11.8	100
2522	29	1.1	13.1	14.9	146	16.8	106
2523	29	1.2	12.2	12.2	143	10.2	99
2524	29	1.2	11.8	12.2	143	8.4	102
2525	29	1.1	12.7	12.7	147	8.8	102

Appendix 9

Appendix 3

Individual Clinical Chemistry Values

2954-001

Sex: Female

Group 2	Day(s) Relative to Start Date	Hemolysis Indice	Icterus Indice	Lipemia Indice
2506	90	N	N	N
2507	90	N	N	N
2508	90	N	N	N
2509	91	N	N	N
2510	91	N	N	N
2511	8	N	N	N
2512	8	N	N	N
2513	8	N	N	N
2514	8	+	N	N
2515	8	N	N	N
2521	29	N	N	N
2522	29	N	N	N
2523	29	N	N	N
2524	29	+	N	N
2525	29	N	N	N

Appendix 9

Appendix 3

Individual Clinical Chemistry Values

2954-001

Sex: Female

Group 3		Reporting Biochemistry					
	Day(s) Relative to Start Date	AST (U/L)	ALT (U/L)	ALP (U/L)	TBIL (mg/dL)	UREAN (mg/dL)	CREAT (mg/dL)
3506	91	99	48	53	0.14	20	0.5
3507	91	76	25	50	0.20	16	0.5
3508	91	123	64	42	0.16	16	0.6
3509	91	190	107	55	0.17	18	0.5
3510	91	87	41	56	0.17	20	0.5
3511	8	168	84	302	0.11	21	0.5
3512	8	76	38	161	0.12	14	0.4
3513	8	85	52	108	0.11	18	0.5
3514	8	73	38	115	0.09	15	0.4
3515	8	84	33	154	0.08	19	0.5
3521	29	1388	622	89	0.21	17	0.4
3522	29	670	284	137	0.20	17	0.4
3523	29	213	34	83	0.14	17	0.5
3524	29	73	31	137	0.11	21	0.4
3525	29	143	67	64	0.12	13	0.3

Appendix 9

Appendix 3

Individual Clinical Chemistry Values

2954-001

Sex: Female

Group 3		Reporting Biochemistry					
	Day(s) Relative to Start Date	GLUC (mg/dL)	CHOL (mg/dL)	TRIG (mg/dL)	TPROT (g/dL)	ALB (g/dL)	GLOB (g/dL)
3506	91	214	81	61	7.6	3.9	3.7
3507	91	121	73	47	7.8	4.2	3.6
3508	91	344	93	84	8.1	4.5	3.6
3509	91	216	84	48	7.9	3.8	4.1
3510	91	104	77	54	7.5	4.0	3.5
3511	8	484	86	49	6.9	4.0	2.9
3512	8	232	88	47	7.0	4.0	3.0
3513	8	297	67	74	7.8	4.5	3.3
3514	8	305	85	35	7.5	4.4	3.1
3515	8	367	69	45	6.7	3.8	2.9
3521	29	217	72	38	6.7	3.5	3.2
3522	29	307	81	69	8.0	4.1	3.9
3523	29	488	65	57	6.9	3.9	3.0
3524	29	473	76	68	7.0	3.7	3.3
3525	29	232	72	84	7.5	4.0	3.5

Appendix 9

Appendix 3

Individual Clinical Chemistry Values

2954-001

Sex: Female

Group 3	Day(s) Relative to Start Date	A/G (ratio)	CA (mg/dL)	PHOS (mg/dL)	NA (mEq/L)	K (mEq/L)	CL (mEq/L)
3506	91	1.1	13.1	12.6	143	11.6	104
3507	91	1.2	11.7	11.1	144	10.4	103
3508	91	1.3	12.9	10.5	141	10.8	101
3509	91	0.9	12.7	11.3	143	8.9	99
3510	91	1.1	12.2	10.2	143	10.0	101
3511	8	1.4	13.6	12.0	140	11.2	100
3512	8	1.3	13.5	18.2	142	9.4	101
3513	8	1.4	14.4	18.0	131	20.0	98
3514	8	1.4	14.4	17.3	140	11.6	101
3515	8	1.3	14.4	17.0	143	12.2	102
3521	29	1.1	11.8	16.3	141	11.8	100
3522	29	1.1	13.3	14.0	139	11.6	101
3523	29	1.3	17.2	10.4	142	9.6	103
3524	29	1.1	13.2	13.0	144	9.8	102
3525	29	1.1	13.5	13.8	143	11.4	99

Appendix 9

Appendix 3

Individual Clinical Chemistry Values

2954-001

Sex: Female

Group 3	Day(s) Relative to Start Date	Hemolysis Indice	Icterus Indice	Lipemia Indice
3506	91	N	N	N
3507	91	N	N	N
3508	91	N	N	N
3509	91	N	N	N
3510	91	N	N	N
3511	8	N	N	N
3512	8	N	N	N
3513	8	N	N	N
3514	8	N	N	N
3515	8	N	N	N
3521	29	+++	N	N
3522	29	++	N	N
3523	29	+	N	N
3524	29	N	N	N
3525	29	N	N	N

Appendix 9

Appendix 3

Individual Clinical Chemistry Values

2954-001

Sex: Female

Group 4		Reporting Biochemistry					
	Day(s) Relative to Start Date	AST (U/L)	ALT (U/L)	ALP (U/L)	TBIL (mg/dL)	UREAN (mg/dL)	CREAT (mg/dL)
4506	91	73	31	46	0.16	16	0.5
4507	91	185	59	46	0.18	16	0.6
4508	91	100	33	45	0.19	19	0.5
4509	91	114	48	46	0.14	20	0.6
4510	91	207	114	49	0.14	14	0.5
4511	8	NT	NT	NT	NT	NT	NT
4512	8	109	33	217	0.12	16	0.5
4513	8	112	38	167	0.11	14	0.4
4514	8	102	33	167	0.11	13	0.4
4515	8	90	25	142	0.08	17	0.4
4521	29	79	48	131	0.13	16	0.3
4522	29	86	34	106	0.15	21	0.4
4523	29	144	67	98	0.14	16	0.4
4524	29	124	33	115	0.17	15	0.4
4525	29	87	35	138	0.13	16	0.4

Appendix 9

Appendix 3

Individual Clinical Chemistry Values

2954-001

Sex: Female

Group 4	Day(s) Relative to Start Date	GLUC (mg/dL)	CHOL (mg/dL)	TRIG (mg/dL)	TPROT (g/dL)	ALB (g/dL)	GLOB (g/dL)
		Reporting Biochemistry					
4506	91	119	75	48	7.9	4.3	3.6
4507	91	146	87	67	8.4	4.6	3.8
4508	91	141	61	43	7.5	4.1	3.4
4509	91	97	82	51	7.2	4.0	3.2
4510	91	349	96	50	8.4	4.2	4.2
4511	8	NT	NT	NT	NT	NT	NT
4512	8	320	73	39	7.4	4.2	3.2
4513	8	227	60	47	6.3	3.6	2.7
4514	8	244	81	41	7.1	4.2	2.9
4515	8	351	72	47	6.0	3.6	2.4
4521	29	229	72	47	7.0	3.6	3.4
4522	29	349	90	67	8.0	4.3	3.7
4523	29	268	59	39	7.7	3.8	3.9
4524	29	116	47	44	7.5	4.0	3.5
4525	29	364	83	52	8.0	4.0	4.0

Appendix 9

Appendix 3

Individual Clinical Chemistry Values

2954-001

Sex: Female

Group 4	Day(s) Relative to Start Date	A/G (ratio)	CA (mg/dL)	PHOS (mg/dL)	NA (mEq/L)	K (mEq/L)	CL (mEq/L)
		Reporting Biochemistry					
4506	91	1.2	13.0	12.5	141	11.8	100
4507	91	1.2	13.1	14.9	140	13.2	100
4508	91	1.2	12.2	12.1	141	13.0	101
4509	91	1.3	12.6	15.3	142	14.2	103
4510	91	1.0	13.6	10.4	141	10.4	100
4511	8	NT	NT	NT	NT	NT	NT
4512	8	1.3	13.9	19.5	141	10.8	99
4513	8	1.3	13.1	14.4	143	11.0	99
4514	8	1.4	13.9	15.7	141	11.0	101
4515	8	1.5	13.3	13.4	146	9.4	101
4521	29	1.1	13.1	12.9	142	9.8	100
4522	29	1.2	14.1	13.2	141	10.6	99
4523	29	1.0	13.8	13.3	143	10.2	100
4524	29	1.1	12.9	13.1	144	10.2	101
4525	29	1.0	14.3	12.5	143	9.4	101

Appendix 9

Appendix 3

Individual Clinical Chemistry Values

2954-001

Sex: Female

Group 4	Day(s) Relative to Start Date	Hemolysis Indice	Icterus Indice	Lipemia Indice
4506	91	N	N	N
4507	91	N	N	N
4508	91	N	N	N
4509	91	N	N	N
4510	91	N	N	N
4511	8	NT	NT	NT
4512	8	N	N	N
4513	8	N	N	N
4514	8	N	N	N
4515	8	N	N	N
4521	29	N	N	N
4522	29	N	N	N
4523	29	N	N	N
4524	29	N	N	N
4525	29	N	N	N

Appendix 9

SIGNATURE(S) FOR DOCUMENT: 2954-001 - 2954-001 Clinical Pathology Final Report

**Individual Scientist:**	I approve this document.
Name:	**Wiedmeyer, Charles**
Wiedmeyer, Charles	17-Aug-2023 14:35:36 (UTC+00:00)
Electronically Signed in M-Files	Timestamp

Appendix 10

Signature Page

SBDOC004226: Final Pathology Report

StageBio Project ID: 02776-0018

Testing Facility: Charles River Laboratories, Inc. / 2954-001

Sponsor: University of Texas Southwestern Medical Center / UTSW.Gray-002

Title: A SINGLE DOSE TOXICITY STUDY OF AAV9/SURF1 ADMINISTERED BY INTRATHECAL INJECTION IN RATS

Reason for Signing	Signature
I certify that all data, interpretations, and conclusions in this report by StageBio are accurate based on the best information available at the time of signature. The work performed at StageBio was in compliance with the provisions stated in the Compliance Statement section of this report.	E17B6A7C-E739-4CA8-8B6F -5014930EAD38<br><br>Jessica Hoane<br>11/2/2022 12:58:24 PM<br>Principal Investigator / Study Pathologist

Appendix 10

FINAL PATHOLOGY REPORT

STUDY PHASE: PATHOLOGY

TESTING FACILITY (TF) STUDY NUMBER: 2954-001

SPONSOR (SPR) STUDY NUMBER: UTSW.GRAY-002

TEST SITE (STAGEBIO) PROJECT ID: 02776-0018

STAGEBIO DOCUMENT ID: SBDOC004226

A SINGLE DOSE TOXICITY STUDY OF AAV9/SURF1 ADMINISTERED BY INTRATHECAL INJECTION IN RATS

TEST SITE:
StageBio
8415 Progress Drive, Suite Q
Frederick, MD 21701

TESTING FACILITY:
Charles River Laboratories, Inc
54943 North Main Street
Mattawan, MI 49071

SPONSOR:
University of Texas Southwestern Medical Center
5323 Harry Hines Blvd
Dallas, TX 75390

02 NOVEMBER 2022

TABLE OF CONTENTS

1. SUMMARY ... 4
2. COMPLIANCE STATEMENT ... 6
3. RESPONSIBLE PERSONNEL ... 7
4. INTRODUCTION ... 8
5. MATERIALS AND METHODS ... 8
TABLE 1. STUDY DESIGN ... 9
5.1. NECROPSY/TISSUE HARVEST ... 9
5.2. SLIDE RECEIPT AT STAGEBIO ... 10
5.3. REPORT ORGANIZATION ... 10
6. RESULTS ... 11
6.1. GENERAL ... 11
6.2. ANIMAL MORTALITY ... 11
6.3. MACROSCOPIC OBSERVATIONS ... 12
6.4. MICROSCOPIC OBSERVATIONS ... 12
6.4.1. TERMINAL SACRIFICE ANIMALS ... 12
6.4.1.1. TEST ARTICLE-RELATED FINDINGS DAY 8 (TABLE 2 AND DATA SECTION I) ... 12
TABLE 2. SUMMARY OF MICROSCOPIC FINDINGS – TERMINAL EUTHANASIA (DAY 8)* ... 13
6.4.1.2. TEST ARTICLE-RELATED FINDINGS DAY 29 (TABLE 3 AND DATA SECTION I) ... 15
TABLE 3. SUMMARY OF MICROSCOPIC FINDINGS – TERMINAL EUTHANASIA (DAY 29)* ... 16
6.4.1.3. TEST ARTICLE-RELATED FINDINGS DAY 91 (TABLE 4 AND DATA SECTION I) ... 22
TABLE 4. SUMMARY OF MICROSCOPIC FINDINGS – TERMINAL EUTHANASIA (DAY 91)* ... 22
6.4.1.4. PROCEDURE-RELATED FINDINGS ... 27
6.4.1.5. NON-TEST ARTICLE-RELATED FINDINGS ... 28
7. DISCUSSION ... 29
8. CONCLUSIONS ... 31
9. REFERENCES ... 32
DATA SECTION I: INDIVIDUAL ANIMAL REPORTS ... I-0
DATA SECTION II: MICROSCOPIC DATA REPORT ... II-0
DATA SECTION III: SUMMARY INCIDENCE AND AVERAGE SEVERITY REPORT ... III-0
DATA SECTION IV: GROSS LESION AND MICROSCOPIC CORRELATE REPORT ... IV-0
DATA SECTION V: COMMENTS REPORT ... V-0

Appendix 10

DATA SECTION VI: FIGURES VI-0
DATA SECTION VII: DIAGNOSIS EXPLANATION REPORT VII-0
APPENDIX 1: QUALITY ASSURANCE STATEMENT 1-0
APPENDIX 2: SUMMARY OF GLP, PROTOCOL, AND SOP DEVIATIONS 2-0
APPENDIX 3: ABBREVIATIONS AND TERMS POSSIBLY USED IN THE DATA SECTIONS 3-0
APPENDIX 4: GLOSSARY OF ANATOMIC TERMS 4-0

1. SUMMARY

Introduction:
This report represents the morphologic observations and an interpretation of those observations from the study titled, *A Single Dose Toxicity Study of AAV9/SURF1 Administered by Intrathecal Injection in Rats*.

The objective of this study (as specifically stated in the study protocol) was to characterize the toxicity, biodistribution and gene expression of AAV9/SURF1 for the treatment of SURF1 Leigh Syndrome.

Methods:
On Study Day 1, 121 rats CD®[Crl:CD®(SD)] (61 male and 60 female) were administered the test article, AAV9/SURF1, or control article/vehicle (PBS with 5% D-sorbitol and 0.001% pluronic F-68) via the lumbar cistern. Group 1 animals (n=15/sex/group) were administered vehicle at 0 vg; Group 2 animals (n=15/sex/group) were administered test article at 0.28E12 vg; Group 3 animals (n=16 males/group and n=15 females/group) were administered test article at 0.83E12 vg; Group 4 animals (n=15/sex/group) were administered test article at 2.49E12 vg. On Study Days 8, 29 (±1), and 91 (±4) (n=41, 40, and 39, respectively), animals were euthanized via carbon dioxide inhalation. Animal 3008 (assigned to the Day 91 cohort) was sacrificed early on Study Day 16.

StageBio received Hematoxylin and Eosin-stained glass slides from Charles River Laboratories for microscopic evaluation.

Jessica Hoane, DVM, MTOX, DACVP, DABT, Senior Pathologist at StageBio located in Frederick, MD 21701, conducted all pathology evaluations detailed in this report. Jacqueline Brassard DVM, PhD, DACVP Pathologist at Brassard Toxicologic Pathology Consultancy Corp. located in Tustin, CA 92780 performed a pathology peer review.

Conclusions:
A single bolus of AAV9/SURF1 at doses of 0.28E12 vg, 0.83E12 vg, or 2.49E12 vg was administered intrathecally into the lumbar cistern in male and female Sprague Dawley rats with scheduled terminations at Days 8, 29, and 91. Early death occurred in a single male dosed with AAV9/SURF1 at 0.83E12 vg; the cause of moribundity was due to procedure/test article-related gray matter degeneration/necrosis predominantly centered on the thoracic spinal cord. There were no macroscopic observations at Days 8, 29, or 91 that were considered to be associated

with the administration of AAV9/SURF1. At the scheduled terminations, single bolus administration of AAV9/SURF1 resulted in microscopic changes in the white matter of the spinal cord at Days 29 and 91 (increased incidence and/or severity of nerve fiber degeneration); the dorsal root ganglia (neuron degeneration/necrosis, mononuclear cell infiltrates, and/or hypertrophy/hyperplasia in [satellite] glial cells) at Days 8, 29, and 91; the dorsal spinal nerve roots (nerve fiber degeneration) at Days 29 and 91; the spinal nerve roots adjacent to the spinal cord at Days 29 and 91 (nerve fiber degeneration); the tibial and sciatic nerves at Days 29 and 91 (increased incidence and/or severity of nerve fiber degeneration and/or Schwann cell hypertrophy/hyperplasia); the heart at Days 29 and 91 (myocardium degeneration/necrosis, mononuclear cell infiltrates, and/or fibrosis); and the liver at Days 8, 29, and 91 (increased incidence and severity of mixed cell infiltrates, single cell necrosis of hepatocytes, Kupffer cell hypertrophy/hyperplasia, and/or increased mitotic figures). Microscopic findings of the gray matter of the spinal cord (degeneration/necrosis, increases in glial cells, and/or mononuclear cell infiltrates) in single animals at Day 8 and Day 29 were considered related to the inadvertent administration of the test material likely within the central canal. In addition, single animals at Day 29 and Day 91 had locally extensive spinal cord changes consistent with inadvertent needle stick into the spinal cord. The degeneration/necrosis of the gray matter of the spinal cord (in combination with the likely inadvertent administration of the AAV9/SURF1 test article into the central canal of the spinal cord), the neuronal degeneration/necrosis within the lumbar DRG, and the heart myocardial changes (degeneration/necrosis, mononuclear cell infiltration, and/or fibrosis) at moderate and greater severity were considered adverse. The test article-related microscopic changes did not, in general, exhibit a dose-dependent trend of incidence and/or severity; however, there was timepoint-dependent variability in occurrence/severity of some microscopic findings. Any variation in microscopic findings due to the test article lot administered, Lot 1 or Lot 2, could not be definitively determined.

2. COMPLIANCE STATEMENT

The portion of this study performed by StageBio was performed in accordance with U.S. Department of Health and Human Services, Food and Drug Administration, United States Code of Federal Regulations, Title 21, Part 58: Good Laboratory Practice for Nonclinical Laboratory Studies and as accepted by Regulatory Authorities throughout the European Union (OECD Principles of Good Laboratory Practice), Japan (MHLW), and other countries that are signatories to the OECD Mutual Acceptance of Data Agreement, as well as the study protocol and amendments, and applicable StageBio Standard Operating Procedures (SOPs).

The Quality Assurance Statement pertinent to this study is contained in Appendix 1. Quality Assurance Audit findings were reported to the study director and study director's management.

GLP, protocol, and SOP deviations are summarized in Appendix 2. Deviations were documented separately and provided to the study director for review.

Raw Data

The raw data produced by StageBio for this study included the following:

- the signed copy of the final pathology report, including all appendices and data sections
- any pertinent documentation needed to substantiate the interpretations in the final pathology report
- the StageBio study correspondence file, which contains electronic and/or hard copies of all pertinent study materials (memos, emails, verbal communication records, draft reports submitted to the study director/sponsor, etc.) that might be necessary to reconstruct the study

Images in this report were not used to make original observations. The images were illustrations of selected portions of the glass slides and used to illustrate methodology. All original observations were made during the examination of glass slides.

Prior to or within a reasonable time upon completion of the final pathology report, all specimens and raw data, including original documentation, will be returned to Charles River Laboratories-MWN.

3. RESPONSIBLE PERSONNEL

Study Pathologist: Jessica Hoane, DVM, MTOX, DACVP, DABT
Senior Pathologist, StageBio
8415 Progress Drive, Suite Q
Frederick, MD 21701
513-204-4400 (Phone)
jhoane@stagebio.com

4. INTRODUCTION

This report represents the morphologic observations and an interpretation of those observations from the study titled, *A Single Dose Toxicity Study of AAV9/SURF1 Administered by Intrathecal Injection in Rats*.

The objective of this study (as specifically stated in the study protocol) was to characterize the toxicity, biodistribution and gene expression of AAV9/SURF1 for the treatment of SURF1 Leigh Syndrome.

This study was sponsored by University of Texas Southwestern Medical Center, Dallas, TX. The sponsor study reference number was UTSW.Gray-002.

The testing facility for this study was Charles River Laboratories, Inc. Mattawan, MI. The testing facility study reference number was 2954-001.

Jessica Hoane, DVM, MTOX, DACVP, DABT, Senior Pathologist at StageBio located in Frederick, MD 21701, conducted all pathology evaluations detailed in this report. The StageBio Project ID was 02776-0018. Jacqueline Brassard DVM, PhD, DACVP Pathologist at Brassard Toxicologic Pathology Consultancy Corp. located in Tustin, CA 92780 performed a pathology peer review.

5. MATERIALS AND METHODS

(In this pathology report, the study design and any methods performed at the testing facility were recorded from the study protocol/amendments.)

The test article for this study was AAV9/SURF1 and the control article/vehicle was PBS containing 5% D-sorbitol and 0.001% pluronic F-68. On Study Day 1, 121 rats CD®[Crl:CD®(SD)] (61 male and 60 female) were dosed using a gas tight Hamilton syringe with a disposable needle inserted into the lumbar cistern. Animals in Group 1 were administered the vehicle (0 vg, n=15/sex/group) and flushed with the vehicle over 30±5 seconds. Animals in Group 2 were administered the test article (0.28E12 vg, n=15/sex/group) and flushed with vehicle over 10±5 seconds. Animals in Group 3 were administered the test article (0.83E12 vg, n=16 males/group and n=15 females/group) and flushed with vehicle over 10±5 seconds. Animals in Group 4 were administered the test article (2.49E12 vg, n=15/sex/group) and flushed with the vehicle over 30±5 seconds. On Study Days 8, 29 (±1), and 91 (±4) (n=41, 40, and 39, respectively), animals

were euthanized via carbon dioxide inhalation. Animal 3008 (assigned to the Day 91 cohort) was sacrificed early on Study Day 16.

The table below reflects the study design as it pertained to the pathology aspect of this study.

Table 1. Study Design

Group	Treatment	Dose Level (vg)	Dose Volume (µL)	Dose Concentration (vg/µL)	Number of Animals							
					Day 1 Dose		Day 8 Necropsy*		Day 29 (±1) Necropsy**		Day 91 (±4) Necropsy	
					M	F	M	F	M	F	M	F
1	Vehicle	0	60[1]<br>74[2]	0	15	15	5	5	5	5	5	5
2	Low Dose	0.28E12	20[1]<br>25[2]	1.38E10[1]<br>1.12E10[2]	15	15	5	5	5	5	5	5
3	Mid Dose	0.83E12	20[1]<br>25[2]	4.15E10[1]<br>3.36E10[2]	16	15	6	5	5	5	5	5
4	High Dose	2.49E12	60[1]<br>74[2]	4.15E10[1]<br>3.36E10[2]	15	15	5	5	5	5	5	5

No. = Number
[1] Lot 1
[2] Lot 2
* Three of the animals from Group 4 in the Day 8 timepoint received the same total vg dose, but in a 74 µL volume due to lower test article dose concentration in Lot 2 of 3.36E10 vg/µL.
** All animals at the Day 29 timepoint received the same total vg dose assigned to each respective group, but in a 24% higher volume due to lower test article dose concentration in Lot 2. Group 1 volume = 74 µL, Group 2 volume = 25 µL, Group 3 volume = 25 µL, and Group 4 volume = 74 µL.

5.1. NECROPSY/TISSUE HARVEST

Charles River Laboratories (CRL) personnel performed all necropsies. At necropsy, tissues were collected and preserved in 10% neutral buffered formalin (NBF). Tissues collected were: brain (left hemisphere) including forebrain, midbrain, hindbrain (including brainstem and cerebellum), eye (left), dorsal root ganglion (cervical, thoracic, and lumbar), heart, kidney (left), liver, lung, Iliac lymph node (left), mandibular lymph node (left), mesenteric lymph node, skeletal muscle (bicep femoris and gastrocnemius [left]), optic nerve (left), sciatic nerve (left), tibial nerve (left), ovary/testis (left), pancreas, spinal cord (cervical, thoracic, and lumbar), spleen, and thymus.

5.2. SLIDE RECEIPT AT STAGEBIO

StageBio received Hematoxylin and Eosin-stained glass slides from CRL for microscopic evaluation.

Jessica Hoane, DVM, MTOX, DACVP, DABT, Senior Pathologist at StageBio located in Frederick, MD 21701, conducted all pathology evaluations detailed in this report. Jacqueline Brassard DVM, PhD, DACVP Pathologist at Brassard Toxicologic Pathology Consultancy Corp. located in Tustin, CA 92780 performed a pathology peer review.

5.3. REPORT ORGANIZATION

Data sections in this pathology report are organized as follows. See the corresponding section introduction pages at the end of this report for expanded explanations of the contents of each data section.

- Data Section I contains the Individual Animal Reports. This data section contains animal identifying information, microscopic findings and any related comments, and gross findings with microscopic correlates (as applicable).
- Data Section II contains the Microscopic Data Report. This data section lists all individual microscopic findings, organized by dose group, in a cross-tab format.
- Data Section III contains the Summary Incidence and Average Severity Report, which lists the incidence of microscopic changes and the group average severity for those changes.
- Data Section IV contains the Gross Lesion and Microscopic Correlate Report.
- Data Section V contains the Comments Report. This report lists all comments pertinent to the study, individual animals, and/or individual findings.
    - Study (General) comments and animal comments not specifically related to a microscopic finding are only in this report (they are not included in Data Section I).
- Data Section VI contains Figures referenced in this report.
- Data Section VII contains the Diagnosis Explanation Report.
    - This report provides a description of each diagnosis used, as well as information regarding how the diagnosis was graded.

Appendices in this pathology report are organized as follows:

- Appendix 1 contains the Quality Assurance Statement for the phases of this study performed at StageBio.

- Appendix 2 contains the Summary of GLP, Protocol, and SOP Deviations that occurred during the phases of this study performed by StageBio
- Appendix 3 contains the Abbreviations and Terms Possibly Used in the Data Sections. This appendix describes the general parameters for the semi-quantitative grading schemes used for any diagnoses not specifically explained in the Diagnosis Explanation Report (Data Section VII). The various grade scores are also defined. The grading scores are relative to each other, i.e. a Grade 2 is more severe than a Grade 1, a Grade 3 is more severe than a Grade 2, etc. The percentages attached to the various grades refer to the tissue that might be expected to be involved, taking into account the relative degree of involvement. For example, a notable focal response may be graded similarly to a more diffuse but less intense response.
- Appendix 4 contains the Glossary of Anatomic Terms. This glossary may include terms not used in this specific study and serves as a general reference to define certain anatomic sites and specific diagnoses.

The computer systems used in the generation of this report include:

- ButtTox for pathology data entry
- BioDox for digital signature collection

6. <u>RESULTS</u>

6.1. GENERAL

The quality of the slides was excellent for microscopic evaluation.

6.2. ANIMAL MORTALITY

References to figures in the following sections relate to images in Data Section VI.

A single animal, Group 3 male Animal 3008 (dosed at 0.83E12 vg), from the Day 91 cohort, was euthanized in extremis on Study Day 16. This animal was noted to have splayed left and right hindlimbs and an abnormal gait on Study Day 14. The cause of moribundity for this animal was determined to be severe degeneration/necrosis (resulting in loss) of the gray matter of the thoracic spinal cord with marked nerve fiber degeneration of the associated white matter, as shown in Figures 1 (transverse section) and 2 (longitudinal section). These thoracic spinal cord changes were bilateral predominantly within the lateral and ventral horns, with sparing of the dorsal horns, and diffusely affected the entirety of the longitudinal spinal cord section. The gray matter degeneration/necrosis was associated with marked increases in glial cells and mild

perivascular mononuclear cell infiltrates. Similar, but less severe findings of gray matter degeneration/necrosis (resulting in vacuolar change and/or loss), white matter nerve fiber degeneration, and increases in glial cells were present in the lumbar segment of the spinal cord as shown in Figure 3. The cervical spinal cord had only minimal increases in glial cells and minimal perivascular mononuclear cell infiltrates. Additional microscopic changes associated with/secondary to the findings in the spinal cord were nerve fiber degeneration and Schwann cell hypertrophy/hyperplasia within the thoracic and lumbar ventral spinal nerve root as shown in Figure 4, as well as mild nerve fiber degeneration and/or minimal Schwann cell hypertrophy/hyperplasia of the sciatic and tibial nerves. The findings in the spinal cord of this animal were considered adverse and related to the presence of the test article, but were also likely dependent upon the inadvertent administration of the test article into the central canal of the spinal cord. These spinal cord findings were similar to those seen in a Day 8 and Day 29 terminal sacrifice animal, as discussed in Section 6.4.1.1 and 6.4.1.2, respectively, below.

6.3. MACROSCOPIC OBSERVATIONS

There were no macroscopic observations in terminal sacrifice animals at Days 8, 29, or 91 that were considered to be related to the intrathecal administration of AAV9/SURF1.

6.4. MICROSCOPIC OBSERVATIONS

References to figures in the following sections relate to images in Data Section VI.

6.4.1. TERMINAL SACRIFICE ANIMALS

6.4.1.1. Test Article-Related Findings Day 8 (Table 2 and Data Section I)

Day 8 female Animal 3512 (0.83E12 vg) had mild degeneration/necrosis of the gray matter of the thoracic spinal cord with minimal nerve fiber degeneration of the associated white matter, as shown in Figures 5 (transverse section) and 6 (longitudinal section). The gray matter degeneration/necrosis in this animal was characterized by cellular swelling and hypereosinophilia. These thoracic spinal cord changes were predominantly just adjacent to the central canal. The gray matter degeneration/necrosis was associated with mild increases in glial cells and minimal perivascular mononuclear cell infiltrates. Similar to the spinal cord findings in early death Animal 3008 (0.83E12 vg), the findings in the spinal cord of this animal were considered adverse but likely dependent upon the inadvertent administration of the test article into the central canal of the spinal cord.

Appendix 10

Additional microscopic changes of note in Day 8 animals were present at various levels (cervical, thoracic, and/or lumbar) of dorsal root ganglia (DRG), heart and liver, as summarized in Table 2 below. At Day 8, the liver was the only tissue that exhibited a definitive test article-related trend in incidence; however, the findings with the DRG and heart are worth noting due to the observations within these tissues at the latter timepoints.

Table 2. Summary of Microscopic Findings – Terminal Euthanasia (Day 8)*

	Males				Females			
**Group**	**1**	**2**	**3**	**4**	**1**	**2**	**3**	**4**
**Dose (vg)**	**0**	**0.28E12**	**0.83E12**	**2.49E12**	**0**	**0.28E12**	**0.83E12**	**2.49E12**
**No. Animals Examined**	**5**	**5**	**6**	**5**	**5**	**5**	**5**	**5**
**Cervical dorsal root ganglia**								
**No. Tissues Examined**	**5**	**5**	**6**	**5**	**5**	**5**	**5**	**5**
Mononuclear cell infiltrates	(0)[a]	(0)	(1)	(0)	(0)	(0)	(0)	(0)
Minimal	--	--	3012	--	--	--	--	--
(Satellite) Glial cell Hypertrophy/hyperplasia	(1)	(3)	(0)	(0)	(1)	(0)	(0)	(2)
Minimal	1014	2011, 2012, 2013	--	--	1515	--	--	4511, 4515
**Lumbar dorsal root ganglia**								
**No. Tissues Examined**	**5**	**5**	**6**	**5**	**5**	**5**	**5**	**5**
Mononuclear cell infiltrates	(0)	(1)	(2)	(0)	(0)	(0)	(1)	(0)
Minimal	--	2014	3011, 4013	--	--	--	3513	--
(Satellite) Glial cell Hypertrophy/hyperplasia	(1)	(4)	(3)	(0)	(1)	(1)	(0)	(3)
Minimal	1014	2011, 2012, 2013, 2015	3011, 3013, 3014	--	1512	2511	--	4511, 4514, 4515
**Thoracic dorsal root ganglia**								
**No. Tissues Examined**	**5**	**5**	**6**	**5**	**5**	**5**	**5**	**5**
(Satellite) Glial cell Hypertrophy/hyperplasia	(1)	(1)	(0)	(0)	(0)	(0)	(0)	(1)
Minimal	1014	2011	--	--	--	--	--	4515

Appendix 10

Table 2 continued

	Males				Females			
**Group**	**1**	**2**	**3**	**4**	**1**	**2**	**3**	**4**
**Dose (vg)**	**0**	**0.28E12**	**0.83E12**	**2.49E12**	**0**	**0.28E12**	**0.83E12**	**2.49E12**
**No. Animals Examined**	**5**	**5**	**6**	**5**	**5**	**5**	**5**	**5**
**Heart**								
**No. Tissues Examined**	**5**	**5**	**6**	**5**	**5**	**5**	**5**	**5**
Myocardium Degeneration/necrosis	(0)	(5)	(3)	(0)	(2)	(0)	(1)	(2)
Minimal	--	2011, 2012, 2013, 2014, 2015	3012, 3014, 3015	--	1511, 1512	--	3511	4512, 4515
Mononuclear cell infiltrates	(0)	(5)	(4)	(1)	(2)	(0)	(1)	(3)
Minimal	--	2011, 2012, 2013, 2014, 2015	3012, 3013, 3014, 3015	4113	1511, 1512	--	3511	4512, 4513 4515
**Liver**								
**No. Tissues Examined**	**5**	**5**	**6**	**5**	**5**	**5**	**5**	**5**
Mixed Cell Infiltrates	(2)	(5)	(6)	(5)	(2)	(4)	(5)	(5)
Minimal	1012, 1014	2011, 2012, 2013, 2014, 2015	3011, 3012, 3013, 3014, 3015, 4013	4011, 4012, 4113, 4021, 4022	1511, 1513	2511, 2513, 2514, 2515	3511, 3512, 3513, 3514, 3515	4511, 4512, 4513, 4514, 4515
Hepatocyte Single cell necrosis	(0)	(0)	(1)	(2)	(0)	(0)	(0)	(0)
Minimal	--	--	3013	4012	--	--	--	--
Mild	--	--	--	4011				
Increased mitotic figures	(0)	(0)	(0)	(1)	(0)	(0)	(0)	(0)
Minimal	--	--	--	4011	--	--	--	--
Kupffer Cell Hypertrophy/hyperplasia	(0)	(1)	(1)	(1)	(0)	(0)	(0)	(1)
Minimal	--	2011	3013	4011	--	--	--	4513

*Severity grades for which there were no findings were omitted from the table.
[a] Numbers in parentheses represent the number of animals with the finding.

Minimal changes in the cervical, thoracic, and/or lumbar dorsal root ganglia (DRG) of mononuclear cell infiltrate and/or (satellite) glial cell hypertrophy/hyperplasia were observed in a small number of animals at Day 8. There was no clear dose-dependent trend in incidence for either DRG observation. It is uncertain if this variation in incidence represents normal biologic variation or was related to the administration of AAV9/SURF1 at this time point.

Myocardial degeneration/necrosis with or without mononuclear cell infiltrates at minimal severity were observed in animals at all doses at Day 8 with no treatment or dose dependent trend.

Microscopic findings of the liver at Day 8 were of typically of minimal severity and included increased incidence of mixed cell infiltrates, single cell necrosis of hepatocytes, increased mitotic figures of hepatocytes and Kupffer cell hypertrophy/hyperplasia. Day 8 males and females at all doses of AAV9/SURF1 had increased incidence of minimal mixed cell infiltrates within the liver when compared to controls. In addition, small numbers of males dosed at 0.83E12 vg and 2.49E12 vg had minimal to mild single cell necrosis, and a single male dosed at 2.49E12 vg had increased hepatocellular mitotic figures. Small numbers of males at all doses of AAV9/SURF1 and a single female dosed at 2.49E12 vg had minimal hypertrophy/hyperplasia of Kupffer cells. There were no liver enzyme alterations in the serum chemistry results for Day 8 animals that correlated with the microscopic liver changes.

6.4.1.2. Test Article-Related Findings Day 29 (Table 3 and Data Section I)

Day 29 female Animal 3525 (0.83E12 vg) had severe degeneration/necrosis (resulting in loss) of the gray matter of the thoracic spinal cord, predominantly of the lateral and ventral horns, with moderate nerve fiber degeneration of the associated white matter, as shown in Figures 7 (transverse section) and 8 (longitudinal section). These thoracic spinal cord changes were bilateral predominantly within the lateral and ventral horns and diffusely affected the entirety of the longitudinal spinal cord section. The gray matter degeneration/necrosis was associated with mild increases in glial cells and minimal perivascular mononuclear cell infiltrates. Similar, but less severe findings of gray matter degeneration/necrosis (resulting in vacuolar change and/or loss), white matter nerve fiber degeneration, increases in glial cells and perivascular mononuclear cell infiltrates were present in the lumbar segment of the spinal cord as shown in Figures 9 (transverse section) and 10 (longitudinal section). The cervical spinal cord had only minimal increases in glial cells and minimal white matter nerve fiber degeneration. Moderate nerve fiber degeneration of the ventral lumbar spinal root, mild nerve fiber degeneration of the inferior cerebellar peduncle of the medulla oblongata, and minimal nerve fiber degeneration

within the pons was considered associated with/secondary to the findings in the spinal cord. Similar to the spinal cord findings in early death Animal 3008 (0.83E12 vg), the findings in the spinal cord of this animal were considered adverse but likely dependent upon the inadvertent administration of the test article into the central canal of the spinal cord.

Additional microscopic changes considered associated with the administration of AAV9/SURF1 at Day 29 were present in various levels (cervical, thoracic, and/or lumbar) of spinal cord white matter, dorsal root ganglia (DRG) and spinal nerve root (dorsal, ventral and/or not otherwise specified [NOS]), sciatic and tibial nerves, heart, and liver, as summarized in Table 3 below.

Table 3. Summary of Microscopic Findings – Terminal Euthanasia (Day 29)*

	Males				Females			
**Group**	**1**	**2**	**3**	**4**	**1**	**2**	**3**	**4**
**Dose (vg)**	**0**	**0.28E12**	**0.83E12**	**2.49E12**	**0**	**0.28E12**	**0.83E12**	**2.49E12**
**No. Animals Examined**	**5**	**5**	**5**	**5**	**5**	**5**	**5**	**5**
**Cervical spinal cord**								
**No. Tissues Examined**	**5**	**5**	**5**	**5**	**5**	**5**	**5**	**5**
White Matter Nerve fiber degeneration	(1)[a]	(1)	(0)	(0)	(0)	(0)	(2)	(1)
Minimal	1024	2025	--	--	--	--	3521, 3525	4524
**Lumbar spinal cord**								
**No. Tissues Examined**	**5**	**5**	**5**	**5**	**5**	**5**	**5**	**5**
White Matter Nerve fiber degeneration	(1)	(0)	(3)	(2)	(0)	(3)	(3)	(3)
Minimal	1025	--	3021, 3023	4023, 4024	--	2521, 2522, 2523	3521, 3522	4521, 4523, 4524
Mild	--	--	3022	--	--	--	--	--
Moderate	--	--	--	--	--	--	3525	--
**Thoracic spinal cord**								
**No. Tissues Examined**	**5**	**5**	**5**	**5**	**5**	**5**	**5**	**5**
White Matter Nerve fiber degeneration	(0)	(1)	(3)	(2)	(0)	(2)	(5)	(3)
Minimal	--	2023	3021, 3022	4024, 4026	--	2521, 2525	3521, 3522, 3523, 3524	4522, 4523, 4524
Mild	--	--	3023	--	--	--	--	--
Moderate	--	--	--	--	--	--	3525	

Appendix 10

Table 3 continued

	Males				Females			
**Group**	**1**	**2**	**3**	**4**	**1**	**2**	**3**	**4**
**Dose (vg)**	**0**	**0.28E12**	**0.83E12**	**2.49E12**	**0**	**0.28E12**	**0.83E12**	**2.49E12**
**No. Animals Examined**	**5**	**5**	**5**	**5**	**5**	**5**	**5**	**5**
**Cervical dorsal root ganglia**								
**No. Tissues Examined**	**5**	**5**	**5**	**5**	**5**	**5**	**5**	**5**
Mononuclear cell infiltrates	(0)	(0)	(1)	(0)	(0)	(0)	(0)	(0)
Minimal	--	--	3021	--	--	--	--	--
(Satellite) Glial cell Hypertrophy/hyperplasia	(3)	(0)	(4)	(3)	(1)	(0)	(2)	(5)
Minimal	1021, 1024, 1025	--	3021, 3022, 3023, 3024	4024, 4025, 4027	1525	--	3521, 3525	4521, 4522, 4523, 4524, 4525
**Lumbar dorsal root ganglia**								
**No. Tissues Examined**	**5**	**5**	**5**	**5**	**5**	**5**	**5**	**5**
Neuron Degeneration/necrosis	(0)	(1)	(0)	(1)	(0)	(0)	(0)	(0)
Minimal	--	2025	--	4024	--	--	--	--
Mononuclear cell infiltrates	(0)	(2)	(4)	(2)	(1)	(0)	(4)	(1)
Minimal	--	2022, 2025	3021, 3022, 3024, 3025	4023	1522	--	3522, 3523, 3524, 3525	4524
Mild	--	--	--	4024	--	--	--	--
(Satellite) Glial cell Hypertrophy/hyperplasia	(3)	(2)	(5)	(5)	(0)	(0)	(4)	(5)
Minimal	1021, 1024, 1025	2021, 2023	3021, 3022, 3023, 3024, 3025	4023, 4025, 4026, 4027	--	--	3521, 3522, 3523, 3524	4521, 4522, 4523, 4524, 4525
Mild	--	--	--	4024	--	--	--	--
**Thoracic dorsal root ganglia**								
**No. Tissues Examined**	**5**	**5**	**4**	**5**	**5**	**5**	**5**	**5**
(Satellite) Glial cell Hypertrophy/hyperplasia	(0)	(0)	(1)	(3)	(1)	(0)	(1)	(1)
Minimal	--	--	3024	4023, 4024, 4025	1524	--	3523	4521

Appendix 10

Table 3 continued

	Males				Females			
**Group**	**1**	**2**	**3**	**4**	**1**	**2**	**3**	**4**
**Dose (vg)**	**0**	**0.28E12**	**0.83E12**	**2.49E12**	**0**	**0.28E12**	**0.83E12**	**2.49E12**
**No. Animals Examined**	**5**	**5**	**5**	**5**	**5**	**5**	**5**	**5**
**Lumbar spinal nerve root**								
**No. Tissues Examined**	**5**	**5**	**5**	**5**	**5**	**5**	**5**	**5**
Dorsal nerve root Nerve fiber degeneration	(1)	(2)	(2)	(3)	(0)	(0)	(3)	(2)
Minimal	1023	2021, 2023	3022, 3023	4023, 4026	--	--	3524, 3525	--
Mild	--	--	--	4024	--	--	--	4522, 4524
Moderate	--	--	--	--	--	--	3523	--
Ventral nerve root Nerve fiber degeneration	(0)	(0)	(2)	(0)	(0)	(2)	(2)	(0)
Minimal	--	--	3023, 3024	--	--	2523, 2524	3521	--
Moderate	--	--	--	--	--	--	3525	--
Spinal nerve root, NOS[b] Nerve fiber degeneration	(0)	(1)	(1)	(2)	(0)	(0)	(0)	(0)
Minimal	--	2025	--	4025, 4026	--	--	--	--
Mild	--	--	3023	--	--	--	--	--
**Thoracic spinal nerve root**								
**No. Tissues Examined**	**5**	**5**	**4**	**5**	**5**	**5**	**5**	**5**
Dorsal nerve root Nerve fiber degeneration	(0)	(0)	(0)	(1)	(0)	(0)	(0)	(0)
Minimal	--	--	--	4024	--	--	--	--
Ventral nerve root Nerve fiber degeneration	(0)	(0)	(0)	(0)	(1)	(0)	(0)	(0)
Minimal	--	--	--	--	1525	--	--	--
**Sciatic Nerve**								
**No. Tissues Examined**	**5**	**5**	**5**	**5**	**5**	**5**	**5**	**5**
Nerve fiber degeneration	(0)	(4)	(4)	(2)	(0)	(2)	(5)	(4)
Minimal	--	2021, 2022, 2023, 2025	3023, 3024	--	--	2525	3523, 3524, 3525	4522
Mild	--	--	3022	4024	--	2523	3522	4521, 4523
Moderate	--	--	3021	4023	--	--	3521	4524
Schwann cell Hypertrophy/hyperplasia	(0)	(0)	(0)	(1)	(0)	(0)	(0)	(0)
Mild	--	--	--	4023	--	--	--	--

Appendix 10

Table 3 continued

	Males				Females			
**Group**	**1**	**2**	**3**	**4**	**1**	**2**	**3**	**4**
**Dose (vg)**	**0**	**0.28E12**	**0.83E12**	**2.49E12**	**0**	**0.28E12**	**0.83E12**	**2.49E12**
**No. Animals Examined**	**5**	**5**	**5**	**5**	**5**	**5**	**5**	**5**
**Tibial Nerve**								
**No. Tissues Examined**	**5**	**5**	**5**	**5**	**5**	**5**	**5**	**5**
Nerve fiber degeneration	(0)	(2)	(2)	(2)	(0)	(1)	(4)	(4)
Minimal	--	2023	--	--	--	--	3523, 3524	4522
Mild	--	2025	--	4024	--	2523	3522	4521, 4523
Moderate	--	--	3021, 3022	4023	--	--	3521	4524
Schwann cell Hypertrophy/hyperplasia	(0)	(0)	(2)	(2)	(0)	(0)	(0)	(0)
Minimal	--	--	3021, 3022	4024	--	--	--	--
Mild	--	--	--	4023	--	--	--	--
**Heart**								
**No. Tissues Examined**	**5**	**5**	**5**	**5**	**5**	**5**	**5**	**5**
Myocardium Degeneration/necrosis	(1)	(5)	(5)	(5)	(0)	(5)	(4)	(4)
Minimal	1023	2021, 2022, 2025	3022	4023, 4024, 4025, 4027	--	2522, 2523	3523	4522, 4524
Mild	--	2023	3024, 3025	4026	--	2521, 2524	3522, 3525	4521
Moderate	--	--	3021	--	--	--	3521	--
Marked	--	2024	3023	--	--	2525	--	4523
Fibrosis	(0)	(2)	(2)	(0)	(0)	(2)	(1)	(1)
Minimal	--	2023	3023, 3024	--	--	2521	3522	4523
Mild	--	2024	--	--	--	--	--	--
Moderate	--	--	--	--	--	2525	--	--
Mononuclear cell infiltrates	(1)	(5)	(5)	(5)	(0)	(5)	(4)	(4)
Minimal	1023	2021, 2022, 2025	3022	4023, 4024, 4025, 4027	--	2522, 2523	3523	4522, 4524
Mild	--	2023	3024, 3025	4026	--	2521, 2524	3522, 3525	4521
Moderate	--	--	3021	--	--	--	3521	--
Marked	--	2024	3023	--	--	2525	--	4523

Appendix 10

Table 3 continued

	Males				Females			
**Group**	**1**	**2**	**3**	**4**	**1**	**2**	**3**	**4**
**Dose (vg)**	**0**	**0.28E12**	**0.83E12**	**2.49E12**	**0**	**0.28E12**	**0.83E12**	**2.49E12**
**No. Animals Examined**	**5**	**5**	**5**	**5**	**5**	**5**	**5**	**5**
**Liver**								
**No. Tissues Examined**	**5**	**5**	**5**	**5**	**5**	**5**	**5**	**5**
Mixed Cell Infiltrates	(4)	(5)	(5)	(5)	(3)	(4)	(5)	(5)
Minimal	1021, 1022, 1023, 1024	2021, 2022, 2023, 2024, 2025	3021, 3023, 3024, 3025	4024, 4026	1521, 1524, 1525	2521, 2524, 2525	3521, 3523, 3524, 3525	4521, 4522, 4523, 4524
Mild	--	--	3022	4023, 4025, 4027	--	2522	3522	4525
Hepatocyte Single cell necrosis	(0)	(0)	(0)	(3)	(0)	(0)	(1)	(0)
Minimal	--	--	--	4023, 4027	--	--	3523	--
Mild	--	--	--	4025	--	--	--	--
Kupffer Cell Hypertrophy/hyperplasia	(0)	(0)	(0)	(2)	(0)	(0)	(1)	(0)
Minimal	--	--	--	4027	--	--	3523	--
Mild	--	--	--	4025	--	--	--	--

*Severity grades for which there were no findings were omitted from the table.
[a] Numbers in parentheses represent the number of animals with the finding.
[b] Spinal nerve root, NOS (not otherwise specified) were sections of spinal nerve root collected and processed with routine sections of spinal cord and could not be definitively assigned to either ventral or dorsal nerve root origin.

Day 29 males and females at all doses of AAV9/SURF1 had increased incidence and/or severity of nerve fiber degeneration of the white matter of the thoracic and/or lumbar spinal cords. The degeneration was often present in the dorsal white matter when the location could be identified on the transverse sections. Nerve fiber degeneration of the spinal cord white matter was diagnosed when there was fragmentation, swelling or hypereosinophilia of axons, dilation of myelin sheaths, and/or presence of digestion chambers (phagocytic cells within dilated spaces).

There were observations of mononuclear cell infiltrates (Figure 11) and/or satellite glial cell (SGC) hypertrophy/hyperplasia (Figure 12) within the cervical, thoracic, and/or lumbar DRG of Day 29 males and females in both controls and animals administered AAV9/SURF1 with a trend of increased incidence and/or severity in AAV9/SURF1 treated animals. The lumbar DRG

exhibited the most microscopic changes, followed by the cervical DRG. SGC hypertrophy/hyperplasia was present in the cervical, thoracic, and/or lumbar DRG at all doses with increased incidence in animals dosed at least 0.83E12 vg and increased severity at 2.49E12 vg. One female dosed at 0.83E12 vg (Animal 3523), had minimal multifocal hypertrophy/hyperplasia in SGCs forming small discrete nodules (nageotte nodules; Figure 12).

Minimal neuronal degeneration/necrosis was present in the lumbar DRG in a single 0.28E12 vg male and a single 2.49E12 vg male and involved one to two neuronal cell bodies. Neuronal degeneration/necrosis was characterized by cytoplasmic hypereosinophilia.

There was increased incidence and/or severity of nerve fiber degeneration of the lumbar dorsal spinal nerve root in females dosed at least 0.83E12 vg and males dosed at least 0.28E12 vg of AAV9/SURF1. There was a low incidence of minimal or moderate nerve fiber degeneration of the lumbar ventral spinal nerve root, occurring in females dosed at 0.28E12 vg and males and females dosed at 0.83E12 vg. Nerve fiber degeneration of spinal nerves with unspecified/undeterminable orientation (relative to ventral or dorsal) was present in a small number of males at all doses AAV9/SURF1.

Day 29 males and females at all doses of AAV9/SURF1 had increased incidence of nerve fiber degeneration of the sciatic nerve and/or tibial nerve.

Day 29 males and females at all doses of AAV9/SURF1 had increased incidence and/or severity of myocardial degeneration/necrosis with mononuclear cell infiltrates of the heart as compared to Day 8, without a dose-dependent trend. There was also increased incidence of fibrosis of the heart at Day 29 at all doses of AAV9/SURF1, except for males dosed at 2.49E12 vg. Representative images of these heart findings are depicted at low (Figure 13) and higher magnification (Figure 14).

Day 29 males and females at all doses of AAV9/SURF1 had increased incidence and/or severity of mixed cell infiltrates within the liver. Minimal to mild Kupffer cell hypertrophy/hyperplasia was observed in a small number of animals, a single female dosed at 0.83E12 vg and two males dosed at 2.49E12 vg. In addition, single cell necrosis of the liver was observed at minimal severity in a female dosed at 0.83E12 vg and at minimal to mild severity in males dosed at 2.49E12 vg. There were no liver enzyme alterations in the serum chemistry results for Day 29 animals that correlated with the microscopic liver changes. A representative image of some of the Day 29 liver findings is depicted in Figure 15.

Statistically significantly increased numbers of lymphocytes (lymphocytosis) occurred in females dosed at 0.28E12 vg and 2.49E12 vg at Day 29 (see CRL's Draft Clinical Pathology Report [provided by the Study Director on 02MAR2022]). As the lymphocytosis was transient, had no clear dose-response, and does not have a clear correlation with the microscopic observations (e.g. mononuclear cell infiltrates) at this time point, no concrete conclusion can be made about the correlation of the lymphocytosis and the administration of AAV9/SURF1.

6.4.1.3. Test Article-Related Findings Day 91 (Table 4 and Data Section I)

Microscopic changes considered associated with the administration of AAV9/SURF1 at Day 91 were present in various levels (cervical, thoracic, and/or lumbar) of spinal cord, dorsal root ganglia (DRG) and spinal nerve root (dorsal, ventral and/or NOS), sciatic and tibial nerves, heart, and liver, as summarized in Table 4 below.

Table 4. Summary of Microscopic Findings – Terminal Euthanasia (Day 91)*

	Males				Females			
**Group**	**1**	**2**	**3**	**4**	**1**	**2**	**3**	**4**
**Dose (vg)**	**0**	**0.28E12**	**0.83E12**	**2.49E12**	**0**	**0.28E12**	**0.83E12**	**2.49E12**
**No. Animals Examined**	**5**	**5**	**4**	**5**	**5**	**5**	**5**	**5**
**Cervical spinal cord**								
**No. Tissues Examined**	**5**	**5**	**4**	**5**	**5**	**5**	**5**	**5**
White Matter Nerve fiber degeneration	(0)[a]	(2)	(2)	(2)	(0)	(0)	(0)	(2)
Minimal	--	2006, 2007	3007, 3009	4006, 4007	--	--	--	4506, 4510
**Lumbar spinal cord**								
**No. Tissues Examined**	**5**	**5**	**4**	**5**	**4**	**5**	**5**	**5**
White Matter Nerve fiber degeneration	(1)	(1)	(2)	(0)	(0)	(0)	(0)	(1)
Minimal	1006	2006	3006, 3009	--	--	--	--	4506
**Thoracic spinal cord**								
**No. Tissues Examined**	**5**	**5**	**4**	**5**	**5**	**5**	**5**	**5**
White Matter Nerve fiber degeneration	(2)	(2)	(3)	(2)	(0)	(2)	(3)	(5)
Minimal	1007, 1009	2009, 2010	3006, 3007, 3009	4008, 4009	--	2506, 2510	3506, 3510	4506, 4507, 4508, 4509, 4510
Mild	--	--	--	--	--	--	3509	--

Appendix 10

Table 4 continued

	Males				Females			
**Group**	**1**	**2**	**3**	**4**	**1**	**2**	**3**	**4**
**Dose (vg)**	**0**	**0.28E12**	**0.83E12**	**2.49E12**	**0**	**0.28E12**	**0.83E12**	**2.49E12**
**No. Animals Examined**	**5**	**5**	**4**	**5**	**5**	**5**	**5**	**5**
**Cervical dorsal root ganglia**								
**No. Tissues Examined**	**5**	**5**	**4**	**5**	**5**	**5**	**5**	**5**
Mononuclear cell infiltrates	(0)	(1)	(0)	(0)	(0)	(1)	(0)	(1)
Minimal	--	2009	--	--	--	2506	--	4509
(Satellite) Glial cell Hypertrophy/hyperplasia	(0)	(2)	(3)	(2)	(0)	(1)	(3)	(3)
Minimal	--	2008, 2009	3007, 3009, 3010	4006, 4008	--	2510	3508, 3509, 3510	4506, 4509, 4510
**Lumbar dorsal root ganglia**								
**No. Tissues Examined**	**5**	**5**	**4**	**5**	**5**	**5**	**5**	**5**
Mononuclear cell infiltrates	(0)	(1)	(3)	(2)	(0)	(0)	(3)	(3)
Minimal	--	--	3006, 3007, 3010	4007, 4010	--	--	3506, 3507, 3509	4507, 4509, 4510
Mild	--	2007	--	--	--	--	--	--
(Satellite) Glial cell Hypertrophy/hyperplasia	(3)	(3)	(3)	(3)	(0)	(4)	(4)	(4)
Minimal	1006, 1007, 1008	2007, 2009, 2010	3007, 3009, 3010	4006, 4007, 4010	--	2506, 2507, 2508, 2510	3506, 3507, 3508, 3509	4506, 4507, 4508, 4509
**Thoracic dorsal root ganglia**								
**No. Tissues Examined**	**5**	**5**	**4**	**5**	**5**	**5**	**5**	**5**
Mononuclear cell infiltrates	(1)	(0)	(2)	(0)	(0)	(1)	(1)	(1)
Minimal	1007	--	3006, 3009	--	--	2510	3506	4508
(Satellite) Glial cell Hypertrophy/hyperplasia	(0)	(1)	(3)	(2)	(0)	(0)	(3)	(2)
Minimal	--	2009	3007, 3009, 3010	4007, 4010	--	--	3508, 3509, 3510	4507, 4509

Appendix 10

Table 4 continued

	Males				Females			
**Group**	**1**	**2**	**3**	**4**	**1**	**2**	**3**	**4**
**Dose (vg)**	**0**	**0.28E12**	**0.83E12**	**2.49E12**	**0**	**0.28E12**	**0.83E12**	**2.49E12**
**No. Animals Examined**	**5**	**5**	**4**	**5**	**5**	**5**	**5**	**5**
**Lumbar spinal nerve root**								
**No. Tissues Examined**	**5**	**5**	**4**	**5**	**5**	**5**	**5**	**5**
Dorsal nerve root Nerve fiber degeneration	(0)	(2)	(0)	(2)	(0)	(0)	(0)	(2)
Minimal	--	2008	--	4006	--	--	--	4507, 4509
Mild	--	2007	--	4007	--	--	--	--
Ventral nerve root Nerve fiber degeneration	(0)	(0)	(1)	(1)	(0)	(1)	(2)	(0)
Minimal	--	--	3010	4010	--	2510	--	--
Mild	--	--	--	--	--	--	3506, 3508	--
Spinal nerve root, NOS[b] Nerve fiber degeneration,	(1)	(0)	(1)	(1)	(0)	(0)	(4)	(2)
Minimal	1007	--	--	4010	--	--	3506, 3507	4510
Mild	--	--	3010	--	--	--	3508	4507
Marked	--	--	--	--	--	--	3509	--
**Thoracic spinal nerve root**								
**No. Tissues Examined**	**5**	**5**	**4**	**5**	**5**	**5**	**5**	**5**
Spinal nerve root, NOS[b] Nerve fiber degeneration	(0)	(1)	(0)	(1)	(0)	(0)	(0)	(0)
Minimal	--	--	--	4010	--	--	--	--
Mild	--	2007	--	--	--	--	--	--
**Sciatic Nerve**								
**No. Tissues Examined**	**5**	**5**	**4**	**5**	**5**	**5**	**5**	**5**
Nerve fiber degeneration	(1)	(3)	(4)	(3)	(0)	(2)	(3)	(4)
Minimal	1008	2007, 2008, 2009	3007, 3009, 3010	4006, 4010	--	2507, 2508	3506, 3508	4509, 4510
Mild	--	--	3006	4007	--	--	3509	4506, 4507
Schwann cell Hypertrophy/hyperplasia	(0)	(0)	(0)	(0)	(0)	(0)	(1)	
Minimal	--	--	--	--	--	--	3509	--

Appendix 10

Table 4 continued

	Males				Females			
**Group**	**1**	**2**	**3**	**4**	**1**	**2**	**3**	**4**
**Dose (vg)**	**0**	**0.28E12**	**0.83E12**	**2.49E12**	**0**	**0.28E12**	**0.83E12**	**2.49E12**
**No. Animals Examined**	**5**	**5**	**4**	**5**	**5**	**5**	**5**	**5**
**Tibial Nerve**								
**No. Tissues Examined**	**5**	**5**	**4**	**5**	**5**	**5**	**5**	**5**
Nerve fiber degeneration	(0)	(1)	(2)	(3)	(0)	(1)	(3)	(4)
Minimal	--	2007	3009	4006, 4007, 4010	--	2507	3506, 3508	4509, 4510
Mild	--	--	3006	--	--	--	3509	4506, 4507
Schwann cell Hypertrophy/hyperplasia	(0)	(0)	(0)	(0)	(0)	(0)	(1)	(0)
Mild	--	--	--	--	--	--	3509	--
**Heart**								
**No. Tissues Examined**	**5**	**5**	**4**	**5**	**5**	**5**	**5**	**5**
Myocardium Degeneration/necrosis	(4)	(5)	(4)	(4)	(1)	(3)	(4)	(5)
Minimal	1007, 1008, 1009, 1010	2006, 2007, 2008, 2009, 2010	3007, 3009, 3010	4006, 4007, 4008, 4010	1510	2506, 2508, 2510	3506, 3507, 3508, 3509	4506, 4507, 4508, 4509, 4510
Mild	--	--	3006	--	--	--	--	--
Fibrosis	(1)	(4)	(1)	(5)	(0)	(0)	(4)	(2)
Minimal	1007	2006, 2007, 2009, 2010	--	4009, 4010	--	--	3506, 3509	4508, 4510
Mild	--	--	--	4006, 4007, 4008	--	--	3507, 3508	--
Moderate	--	--	3006	--	--	--	--	--
Mononuclear cell infiltrates	(4)	(5)	(4)	(4)	(2)	(3)	(5)	(5)
Minimal	1007, 1008, 1009, 1010	2006, 2007, 2008, 2009	3007, 3009, 3010	4010	1509, 1510	2508, 2510	3510	4506, 4507, 4508, 4509, 4510
Mild	--	2010	--	4006, 4007, 4008	--	2506	3506, 3507, 3508, 3509	--
Marked	--	--	3006	--	--	--	--	--

Table 4 continued

	Males				Females			
**Group**	**1**	**2**	**3**	**4**	**1**	**2**	**3**	**4**
**Dose (vg)**	**0**	**0.28E12**	**0.83E12**	**2.49E12**	**0**	**0.28E12**	**0.83E12**	**2.49E12**
**No. Animals Examined**	**5**	**5**	**4**	**5**	**5**	**5**	**5**	**5**
**Liver**								
**No. Tissues Examined**	**5**	**5**	**4**	**5**	**5**	**5**	**5**	**5**
Mixed Cell Infiltrates	(3)	(5)	(3)	(4)	(2)	(1)	(1)	(4)
Minimal	1007, 1009, 1010	2006, 2007, 2008, 2009	3006, 3007, 3009	4006, 4007, 4008, 4009	1506, 1510	2510	3510	4506, 4508, 4509, 4510
Mild	--	2010	--	--	--	--	--	--

*Severity grades for which there were no findings were omitted from the table.
[a] Numbers in parentheses represent the number of animals with the finding.
[b] Spinal nerve root, NOS (not otherwise specified) were sections of spinal nerve root collected and processed with routine sections of spinal cord and could not be definitively assigned to either ventral or dorsal nerve root origin.

Day 91 males and females at all doses of AAV9/SURF1 had increased incidence and/or severity of nerve fiber degeneration of the white matter of the cervical, thoracic and/or lumbar spinal cord. The degeneration was most often present in the dorsal white matter, when the location could be identified on the transverse sections, but was also observed in the ventral, lateral or ventrolateral white matter.

There were observations of mononuclear cell infiltration and/or SGC hypertrophy/hyperplasia within the cervical, thoracic, and/or lumbar DRG of Day 91 males and females in both controls and animals administered AAV9/SURF1. There was a trend of increased incidence and/or severity of mononuclear cell infiltration in the lumbar DRG of males and females dosed at least 0.83E12 vg and of SGC hypertrophy/hyperplasia in cervical and thoracic DRG at all doses of AAV9/SURF1 when compared to Day 91 controls. There was increased incidence and/or severity of nerve fiber degeneration of the lumbar dorsal spinal nerve root in males dosed at 0.28E12 vg and males and females dosed at 2.49E12 vg. There was low incidence of minimal or mild nerve fiber degeneration of the lumbar ventral spinal nerve root, occurring in males dosed at least 0.83E12 vg and females dosed at 0.28E12 and 0.83E12 vg. Nerve fiber degeneration of spinal nerves of the lumbar and/or thoracic regions with unspecified/undeterminable orientation (relative to ventral or dorsal) was present in a small number of males at all doses of AAV9/SURF1 and females dosed at least 0.83E12 vg. In comparison, to Day 29 animals, there was no definitive trend present across all treatment groups in increase or decrease incidence

and/or severity of lumbar or cervical DRG changes seen at Day 91. There was, however, a small increased incidence of mononuclear cell infiltrates and SGC hypertrophy/hyperplasia in most AAV9/SURF1 treatment groups in the Day 91 thoracic DRG, when compared to Day 29.

Similar to what was observed at Day 29, Day 91 males and females at all doses AAV9/SURF1 had increased incidence of minimal to mild nerve fiber degeneration of the sciatic nerve and/or tibial nerve. In addition, there was minimal to mild Schwann cell hypertrophy/hyperplasia of the sciatic nerve and/or tibial nerve in a single female at 0.83E12 vg.

Day 91 males and females at all doses of AAV9/SURF1 had increased incidence and/or severity of myocardial degeneration/necrosis and mononuclear cell infiltrates of the heart, with no clear dose-dependent trend. Myocardial degeneration/necrosis and mononuclear cell infiltrates were present, in general, at lower average severity in Day 91 AAV9/SURF1 treatment groups than seen at Day 29. The increased incidence and/or severity of fibrosis of the heart at Day 91 in males dosed at least 0.28E12 vg and females dosed at least 0.83E12 vg, lacked a clear dose dependent trend. Heart fibrosis was present, in general, at increased incidence in Day 91 AAV9/SURF1 treatment groups than seen at Day 29.

At Day 91, the findings in the liver had largely resolved. Mixed cell infiltrates were present in all treatment groups, including male and female controls, and were of equivocal incidence in males across treatment groups, but exhibited an increase incidence in females at 2.49E12 vg compared to that in controls. The mixed cell infiltrates in the liver were minimal in all animals with the exception of Animal 2010, which had mild infiltrates.

6.4.1.4. Procedure-Related Findings

Day 29 male Animal 3023 (0.83E12 vg) had minimal locally extensive inflammation of the thoracic spinal cord at the junction of the white and gray matter associated with mild nerve fiber degeneration of the adjacent lateral white matter tract. The linearity of the lesion was consistent with accidental needle puncture of the spinal cord.

Day 91 female Animal 2506 (0.28E12 vg) had locally extensive increased glial cells that was oriented perpendicular to the long axis of the lumbar spinal cord, as shown in Figure 16. In addition to increased glial cells, there were a small number of macrophages with intracytoplasmic accumulation of golden-brown pigment consistent with hemosiderin. These findings were considered consistent with a needle tract and were considered related to the intrathecal administration procedure.

Minimal to mild mononuclear cell infiltrates were present within multiple locations of the central nervous system in small numbers of animals administered AAV9/SURF1 at Day 8, Day 29, and/or Day 91 including the meninges of the brain, the meninges of varying spinal cord segments, and within the pineal gland. This finding was not present in concurrent study control animals at any timepoint and was thought most likely to represent a local reaction to the presence of the test article within the cerebrospinal fluid.

6.4.1.5. Non-Test Article-Related Findings

Minimal focal mononuclear cell infiltrates were present in the epineurium of the spinal nerve root just adjacent to the DRG. These infiltrates were most often seen in lumbar DRG but were also infrequently present in cervical DRG and thoracic DRG. These infiltrates were sometimes bilateral, sometimes unilateral, and as they were seen in controls at Day 29 and Day 91, the minimal mononuclear cell infiltrates of the epineurium were considered to be spontaneous and unrelated to the administration of AAV9/SURF1. Variation in incidence was thought to be due to the inconsistency of the presence of the epineurium at the spinal nerve root/DRG junction in the sections evaluated.

Minimal nerve fiber degeneration of the white matter, specifically pyramidal tracts, of the medulla oblongata was present in a small number of animals in multiple treatment groups, including controls, and thus was considered spontaneous and unrelated to administration of AAV9/SURF1.

Nerve fiber degeneration of minimal severity within the sciatic and tibial nerves was considered to be of equivocal incidence/severity in Day 8 animals with most observations of minimal severity affecting three or less nerve fibers, and mild severity present only in a single control male (Animal 1011). A minimal degree of nerve fiber degeneration is common in rats as a spontaneous background change (Pardo et al., 2020).

Additional microscopic findings observed in Day 8, Day 29, or Day 91 animals, not described above, were considered incidental, consistent with those seen in laboratory rats of this strain and age, and/or exhibited no treatment-related trend of incidence, and therefore were considered unrelated to administration of AAV9/SURF1.

7. DISCUSSION

The spinal cord findings present in Day 8 Group 3 (0.83E12 vg; female) Animal 3512, Day 29 Group 3 (0.83E12 vg; female) Animal 3525, and early death Day 16 Group 3 (0.83E12 vg; male) Animal 3008, were similar in that they were focused primarily on the thoracic spinal cord lateral and ventral gray matter and were bilateral around the central canal. In the Day 8 Animal 3512, the gray matter degeneration/necrosis was mild in severity and was very focused around the central canal. In the Day 16 early death Animal 3008 and the Day 29 Animal 3525, the gray matter degeneration/necrosis were severe, affected the lateral and ventral horns bilaterally symmetric around the central canal, and extended through the sections of thoracic spinal cord available for evaluation and into the adjacent lumbar spinal cord, with minimal cellular changes including increases in glial cells and/or mononuclear cell infiltrates in the cervical spinal cord. As the changes were similar, exhibited bilateral symmetry targeting the gray matter predominantly of the thoracic spinal cord, and exhibited progression over time, these changes were considered related to the inadvertent administration of AAV9/SURF1 likely into the central canal of the spinal cord.

Nerve fiber degeneration of the spinal cord white matter was present at increased incidence and/or severity in AAV9/SURF1 treated animals at all doses at Day 29 and Day 91. Central axonopathy associated with AAV administration has been reported in primates (Hordeaux et al., 2020).

At Day 29, there was rare incidence of neuronal degeneration/necrosis in the lumbar DRG of two males (Animals 2025 and 4024) dosed at 0.28E12 vg and 2.49E12 vg, respectively, and in one female (Animal 3523) dosed at 0.83E12 vg, multifocal increases in glial cells resulting in small nodule formation, reminiscent of what has been described for nodules of Nageotte. The cellular infiltrates/increases in this animal were considered to be responses to neuronal cell death. Although these changes were largely minimal in severity, they were considered adverse due to the loss of ganglion neurons. Mononuclear cell infiltrates and/or SGC hypertrophy/hyperplasia of the cervical, thoracic and/or lumbar ganglia were typically minimal in severity. Differentiation of mononuclear cell infiltration and SGC hypertrophy/hyperplasia was sometimes problematic due to occasional collection artifact. As these findings were present in males and females in all treatment groups, including controls, there was some background level of these changes, and there was no clear treatment-related association of the changes in the DRG with administration of AAV9/SURF1 at Day 8. At Day 29 and Day 91, there were trends of increased incidence of mononuclear cell infiltrates and/or SGC hyperplasia of one or more of the DRG levels examined in AAV9/SURF1 treatment groups. Mononuclear cell

infiltrates and neuronal degeneration within DRG associated with AAV administration has been reported in primates (Hordeaux et al., 2020). The changes in the lumbar DRG were often, but not always, associated with nerve fiber degeneration of the dorsal spinal nerve root. It may be beneficial in future studies to collect and evaluate DRGs from the lumbosacral/sacral region of the spinal cord.

Minimal nerve fiber degeneration of the spinal nerves was considered spontaneous and unrelated to administration of AAV9/SURF1; however, mild nerve fiber degeneration was considered of greater severity than expected as a spontaneous finding, and thus was considered related to AAV9/SURF1 administration.

Nerve fiber degeneration of the sciatic and tibial nerves exhibited an increase in incidence and/or severity at all doses of AAV9/SURF1 at both Days 29 and 91, and was, in general, present at lower average severity at Day 91 than at Day 29. In addition, Schwann cell hypertrophy/hyperplasia within the sciatic nerve and/or tibial nerve was present in a small number of males at Day 29 and a single female at Day 91. Peripheral axonopathy associated with AAV administration has been reported in primates (Hordeaux et al., 2020).

Rodent progressive cardiomyopathy is a common spontaneous findings of Sprague Dawley rats (Chanut et al., 2013), and was seen in control animals in this study (diagnosed as its separate components of degeneration/necrosis, cellular infiltrates, and fibrosis). However, these changes in AAV9/SURF1 animals were considered to be test article related as they exhibited increased incidence and severity over controls and the changes were more likely to be multifocal and distributed over a larger extent of the heart when compared to controls. Myocardial changes within the heart at Day 29 and Day 91 timepoints in AAV9/SURF1 treated animals were considered related to the administration of AAV9/SURF1, and these findings exhibited no dose dependent trend. The increased incidence of myocardial changes in some AAV9/SURF1 treated animals at the Day 8 timepoint cannot be definitively attributed solely to spontaneous background changes and may be related to AAV9/SURF1 administration. The changes in the heart were multifocal in nature and could affect any portion of the heart myocardium, including atria. The degeneration/necrosis of the myocardium was always associated with mononuclear cell infiltration, and fibrosis resulting from the damage to the heart was at higher incidence in Day 91 animals. The heart changes at moderate or greater severity would be considered adverse because the extent of change at moderate or greater severity would be expected to result in diminished function of the heart and diminished capacity to respond to increased demand on the heart.

Minimal mixed cell infiltration of the liver is a common spontaneous finding of Sprague Dawley rats and was seen in controls in this study. However, this finding was present at increased incidence and/or severity in AAV9/SURF1 treated animals at all timepoints and was considered due to increased hepatocyte cell death. Single cell necrosis of hepatocytes was evident in a small number of AAV9/SURF1 treated animals at both Day 8 and Day 29, but not at Day 91, and this change was not associated with statistically significant increases of mean group liver enzyme values (AST, ALT or ALKP). The severity of the mixed cell infiltrates was decreased in Day 91 animals compared to Day 8 and Day 29. Kupffer cell hypertrophy/hyperplasia was present only in a small number of animals at Day 8 and Day 29. There is no clear association the Day 91 liver findings with the statistically significant decreases in liver weights, both absolute and relative to brain and body weight, in Day 91 females dosed at least 0.83E12 vg or of absolute liver weight in Day 91 males dosed at 2.49E12 vg.

Two lots, Lot 1 and Lot 2, of the AAV9/SURF1 test article were used to dose animals in the study. All Day 91 animals and most Day 8 animals, with the exception of three of the ten Group 4 animals, received Lot 1 AAV9/SURF1. The three Group 4 Day 8 animals and all Day 29 animals received Lot 2 AAV9/SURF1. As the test article Lots were, for the most part, given to animals on studies of different duration, and there was some variation in microscopic findings based upon study duration, it is difficult to fully assess any variation in test article effects associated with the two test article lots. However, the gray matter spinal cord changes occurred in an animal from each the Day 8, Day 29, and Day 91 cohorts in the same dose group (0.83E12 vg).

8. CONCLUSIONS

A single bolus of AAV9/SURF1 at doses of 0.28E12 vg, 0.83E12 vg, or 2.49E12 vg was administered intrathecally into the lumbar cistern in male and female Sprague Dawley rats with scheduled terminations at Days 8, 29, and 91. Early death occurred in a single male dosed with AAV9/SURF1 at 0.83E12 vg; the cause of moribundity was due to procedure/test article-related gray matter degeneration/necrosis predominantly centered on the thoracic spinal cord. There were no macroscopic observations at Days 8, 29, or 91 that were considered to be associated with the administration of AAV9/SURF1. At the scheduled terminations, single bolus administration of AAV9/SURF1 resulted in microscopic changes in the white matter of the spinal cord at Days 29 and 91 (increased incidence and/or severity of nerve fiber degeneration); the dorsal root ganglia (neuron degeneration/necrosis, mononuclear cell infiltrates, and/or hypertrophy/hyperplasia in [satellite] glial cells) at Days 8, 29, and 91; the dorsal spinal nerve roots (nerve fiber degeneration) at Days 29 and 91; the spinal nerve roots adjacent to the spinal

cord at Days 29 and 91 (nerve fiber degeneration); the tibial and sciatic nerves at Days 29 and 91 (increased incidence and/or severity of nerve fiber degeneration and/or Schwann cell hypertrophy/hyperplasia); the heart at Days 29 and 91 (myocardium degeneration/necrosis, mononuclear cell infiltrates, and/or fibrosis); and the liver at Days 8, 29, and 91 (increased incidence and severity of mixed cell infiltrates, single cell necrosis of hepatocytes, Kupffer cell hypertrophy/hyperplasia, and/or increased mitotic figures). Microscopic findings of the gray matter of the spinal cord (degeneration/necrosis, increases in glial cells, and/or mononuclear cell infiltrates) in single animals at Day 8 and Day 29 were considered related to the inadvertent administration of the test material likely within the central canal. In addition, single animals at Day 29 and Day 91 had locally extensive spinal cord changes consistent with inadvertent needle stick into the spinal cord. The degeneration/necrosis of the gray matter of the spinal cord (in combination with the likely inadvertent administration of the AAV9/SURF1 test article into the central canal of the spinal cord), the neuronal degeneration/necrosis within the lumbar DRG, and the heart myocardial changes (degeneration/necrosis, mononuclear cell infiltration, and/or fibrosis) at moderate and greater severity were considered adverse. The test article-related microscopic changes did not, in general, exhibit a dose-dependent trend of incidence and/or severity; however, there was timepoint-dependent variability in occurrence/severity of some microscopic findings. Any variation in microscopic findings due to the test article lot administered, Lot 1 or Lot 2, could not be definitively determined.

9. REFERENCES

Chanut, F., Kimbrough, C., Hailey, R., Berridge, B., Hughes-Earle, A., et al. (2013). Spontaneous Cardiomyopathy in Young Sprague-Dawley Rats: Evaluation of Biological and Environmental Variability. *Toxicologic Pathology*, 41: 1126-1136.

Hordeaux, J., Buza, EL., Dyer, C., Goode, T., Mitchell, TW., et al. (2020). Adeno-Associated Virus-Induced Dorsal Root Ganglion Pathology. Human Gene Therapy. 31, 808-818.

Pardo, ID., Weber, K., Cramer, S., Krinke, GJ., Butt, MT., et al. (2020). Atlas of Normal Microanatomy, Procedural and Processing Artifacts, Common Background Findings, and Neurotoxic Lesions in the Peripheral Nervous System of Laboratory Animals. *Toxicologic Pathology*, 48(1): 105-131.

Appendix 10

DATA SECTION I: INDIVIDUAL ANIMAL REPORTS

This data section contains the following information:

- Animal ID, sex, sacrifice, treatment group, fate, time on test, cause of death (if other than a scheduled sacrifice)
- Gross Examination (Gross Lesions)
  - Each gross lesion description contains the following:
    - Gross lesion description (tissue and text) provided by the testing facility or recorded at the time of tissue trimming (on a StageBio histology form).
    - TGL# (Tissue Gross Lesion Number)
      - The TGL# is used to correlate microscopic changes.
      - TGL# is further defined in Appendix 3.
    - Microscopic correlate (or an indication that no correlating change was present). Note that the correlating microscopic change does not contain any comments specific to that microscopic diagnosis. That information is present under the specific tissue/diagnosis in the Microscopic Examination portion of the report for that animal.
- Microscopic Examination.
  - Disposition: Normal
    - Lists those tissues interpreted to be within normal limits, including tissues for which microscopic changes determined to be artifacts of preparation were recorded (providing there were no abnormal findings).
    - Bilateral tissues may have the disposition Normal Unilateral; Not Examined Unilateral, which is the equivalent of normal.
  - Disposition: Abnormal/Finding
    - Lists those tissues with findings interpreted to be abnormal.
    - All microscopic diagnoses include at least the tissue, a diagnosis, and a grade. A specific site and various modifiers may also be listed.
    - Includes comments specific to that diagnosis
  - Tissues under other dispositions (Examined; Unable to Obtain Specimen; Not Present at Trim; Autolyzed; Recut, Retrim, or Reembed Pending) may also be listed. See Appendix 3 for a definition of these dispositions.

Appendix 10

Individual Animal Report

Study ID: 02776-0018: CRL 2954-001 / U of Tex Southwestern Med UTSW.Gray-002
Study Title: A SINGLE DOSE TOXICITY STUDY OF AAV9/SURF1 ADMINISTERED BY INTRATHECAL INJECTION IN RATS

Animal: 1011 (Male)

Sacrifice: DAY 08
Group: 1MD08 / VEHICLE (0 VG)
Fate: SCHEDULED SACRIFICE / Time on Test: 08 / Cause(s) of Death:

Gross or Microscopic Finding
Disposition
Tissue: Site / Finding, Severity

MICROSCOPIC EXAMINATION

Disposition: NORMAL

BRAIN, AMYGDALOID BODY
BRAIN, BASAL NUCLEI/STRIATUM
BRAIN, CEREBELLUM
BRAIN, CEREBRAL CORTEX
BRAIN, HIPPOCAMPUS
BRAIN, HYPOTHALAMUS
BRAIN, MEDULLA OBLONGATA
BRAIN, MENINGES
BRAIN, MIDBRAIN
BRAIN, OLFACTORY BULB
BRAIN, PONS
BRAIN, THALAMUS
BRAIN, VENTRICULAR SYSTEM
BRAIN, WHITE MATTER
EYE
GANGLION, DORSAL ROOT, CERVICAL
GANGLION, DORSAL ROOT, LUMBAR
GANGLION, DORSAL ROOT, THORACIC
HEART
LUNG
LYMPH NODE, ILIAC
LYMPH NODE, MANDIBULAR
LYMPH NODE, MESENTERIC
MUSCLE, BICEPS FEMORIS
MUSCLE, GASTROCNEMIUS
NERVE ROOT, SPINAL, CERVICAL
NERVE ROOT, SPINAL, LUMBAR
NERVE ROOT, SPINAL, THORACIC
NERVE, OPTIC
NERVE, TIBIAL
PANCREAS
SPINAL CORD, CERVICAL
SPINAL CORD, THORACIC
SPLEEN

Appendix 10

Individual Animal Report

Study ID: 02776-0018: CRL 2954-001 / U of Tex Southwestern Med UTSW.Gray-002
Study Title: A SINGLE DOSE TOXICITY STUDY OF AAV9/SURF1 ADMINISTERED BY INTRATHECAL INJECTION IN RATS

Animal: 1011 (Male)

Sacrifice: DAY 08
Group: 1MD08 / VEHICLE (0 VG)
Fate: SCHEDULED SACRIFICE / Time on Test: 08 / Cause(s) of Death:

Gross or Microscopic Finding
Disposition
Tissue: Site / Finding, Severity

TESTIS
THYMUS

Disposition: **ABNORMAL/FINDING**

KIDNEY
KIDNEY: CHRONIC PROGRESSIVE NEPHROPATHY, MINIMAL
LIVER
LIVER: EXTRAMEDULLARY HEMATOPOIESIS, MINIMAL
NERVE, SCIATIC
NERVE, SCIATIC: DEGENERATION, NERVE FIBER, MILD
SPINAL CORD, LUMBAR
SPINAL CORD, LUMBAR, NERVE ROOT, SPINAL: DEGENERATION, NERVE FIBER, MINIMAL

Appendix 10

Individual Animal Report

Study ID: 02776-0018: CRL 2954-001 / U of Tex Southwestern Med UTSW.Gray-002
Study Title: A SINGLE DOSE TOXICITY STUDY OF AAV9/SURF1 ADMINISTERED BY INTRATHECAL INJECTION IN RATS

Animal: 1012 (Male)

Sacrifice: DAY 08
Group: 1MD08 / VEHICLE (0 VG)
Fate: SCHEDULED SACRIFICE / Time on Test: 08 / Cause(s) of Death:

Gross or Microscopic Finding
Disposition
Tissue: Site / Finding, Severity

MICROSCOPIC EXAMINATION

Disposition: NORMAL

BRAIN, AMYGDALOID BODY
BRAIN, BASAL NUCLEI/STRIATUM
BRAIN, CEREBELLUM
BRAIN, CEREBRAL CORTEX
BRAIN, HIPPOCAMPUS
BRAIN, HYPOTHALAMUS
BRAIN, MEDULLA OBLONGATA
BRAIN, MENINGES
BRAIN, MIDBRAIN
BRAIN, OLFACTORY BULB
BRAIN, PONS
BRAIN, THALAMUS
BRAIN, VENTRICULAR SYSTEM
BRAIN, WHITE MATTER
EYE
GANGLION, DORSAL ROOT, CERVICAL
GANGLION, DORSAL ROOT, LUMBAR
GANGLION, DORSAL ROOT, THORACIC
HEART
KIDNEY
LUNG
LYMPH NODE, ILIAC
LYMPH NODE, MANDIBULAR
LYMPH NODE, MESENTERIC
MUSCLE, BICEPS FEMORIS
MUSCLE, GASTROCNEMIUS
NERVE ROOT, SPINAL, CERVICAL
NERVE ROOT, SPINAL, LUMBAR
NERVE ROOT, SPINAL, THORACIC
NERVE, OPTIC
NERVE, SCIATIC
NERVE, TIBIAL
PANCREAS
SPINAL CORD, CERVICAL

Appendix 10

Individual Animal Report

Study ID: 02776-0018: CRL 2954-001 / U of Tex Southwestern Med UTSW.Gray-002
Study Title: A SINGLE DOSE TOXICITY STUDY OF AAV9/SURF1 ADMINISTERED BY INTRATHECAL INJECTION IN RATS

Animal: 1012 (Male)

Sacrifice: DAY 08
Group: 1MD08 / VEHICLE (0 VG)
Fate: SCHEDULED SACRIFICE / Time on Test: 08 / Cause(s) of Death:

Gross or Microscopic Finding
Disposition
Tissue: Site / Finding, Severity

SPINAL CORD, LUMBAR
SPINAL CORD, THORACIC
SPLEEN
TESTIS
THYMUS

Disposition: ABNORMAL/FINDING

LIVER
LIVER: INFILTRATE, MIXED, MINIMAL

Appendix 10

Individual Animal Report

Study ID: 02776-0018: CRL 2954-001 / U of Tex Southwestern Med UTSW.Gray-002
Study Title: A SINGLE DOSE TOXICITY STUDY OF AAV9/SURF1 ADMINISTERED BY INTRATHECAL INJECTION IN RATS

Animal: 1013 (Male)

Sacrifice: DAY 08
Group: 1MD08 / VEHICLE (0 VG)
Fate: SCHEDULED SACRIFICE / Time on Test: 08 / Cause(s) of Death:

Gross or Microscopic Finding
Disposition
Tissue: Site / Finding, Severity

MICROSCOPIC EXAMINATION

Disposition: NORMAL

BRAIN, AMYGDALOID BODY
BRAIN, BASAL NUCLEI/STRIATUM
BRAIN, CEREBELLUM
BRAIN, CEREBRAL CORTEX
BRAIN, HIPPOCAMPUS
BRAIN, HYPOTHALAMUS
BRAIN, MEDULLA OBLONGATA
BRAIN, MENINGES
BRAIN, MIDBRAIN
BRAIN, OLFACTORY BULB
BRAIN, PONS
BRAIN, THALAMUS
BRAIN, VENTRICULAR SYSTEM
BRAIN, WHITE MATTER
EYE
GANGLION, DORSAL ROOT, CERVICAL
GANGLION, DORSAL ROOT, LUMBAR
GANGLION, DORSAL ROOT, THORACIC
HEART
LUNG
LYMPH NODE, ILIAC
LYMPH NODE, MANDIBULAR
LYMPH NODE, MESENTERIC
MUSCLE, BICEPS FEMORIS
MUSCLE, GASTROCNEMIUS
NERVE ROOT, SPINAL, CERVICAL
NERVE, OPTIC
NERVE, TIBIAL
PANCREAS
SPINAL CORD, CERVICAL
SPINAL CORD, LUMBAR
SPINAL CORD, THORACIC
SPLEEN
TESTIS

Appendix 10

Individual Animal Report

Study ID: 02776-0018: CRL 2954-001 / U of Tex Southwestern Med UTSW.Gray-002
Study Title: A SINGLE DOSE TOXICITY STUDY OF AAV9/SURF1 ADMINISTERED BY INTRATHECAL INJECTION IN RATS

Animal: 1013 (Male)

Sacrifice: DAY 08
Group: 1MD08 / VEHICLE (0 VG)
Fate: SCHEDULED SACRIFICE / Time on Test: 08 / Cause(s) of Death:

Gross or Microscopic Finding
Disposition
Tissue: Site / Finding, Severity

THYMUS

Disposition: ABNORMAL/FINDING

KIDNEY
KIDNEY, TUBULAR: BASOPHILIA, MINIMAL
KIDNEY, TUBULAR: CYST, MINIMAL
LIVER
LIVER: EXTRAMEDULLARY HEMATOPOIESIS, MINIMAL
NERVE ROOT, SPINAL, LUMBAR
NERVE ROOT, SPINAL, LUMBAR, NERVE ROOT, VENTRAL: DEGENERATION, NERVE FIBER, MINIMAL
NERVE ROOT, SPINAL, THORACIC
NERVE ROOT, SPINAL, THORACIC, NERVE ROOT, VENTRAL: DEGENERATION, NERVE FIBER, MINIMAL
NERVE, SCIATIC
NERVE, SCIATIC: DEGENERATION, NERVE FIBER, MINIMAL

Appendix 10

Individual Animal Report

Study ID: 02776-0018: CRL 2954-001 / U of Tex Southwestern Med UTSW.Gray-002
Study Title: A SINGLE DOSE TOXICITY STUDY OF AAV9/SURF1 ADMINISTERED BY INTRATHECAL INJECTION IN RATS

Animal: 1014 (Male)

Sacrifice: DAY 08
Group: 1MD08 / VEHICLE (0 VG)
Fate: SCHEDULED SACRIFICE / Time on Test: 08 / Cause(s) of Death:

Gross or Microscopic Finding
Disposition
Tissue: Site / Finding, Severity

MICROSCOPIC EXAMINATION

Disposition: NORMAL

BRAIN, AMYGDALOID BODY
BRAIN, BASAL NUCLEI/STRIATUM
BRAIN, CEREBELLUM
BRAIN, CEREBRAL CORTEX
BRAIN, HIPPOCAMPUS
BRAIN, HYPOTHALAMUS
BRAIN, MEDULLA OBLONGATA
BRAIN, MENINGES
BRAIN, MIDBRAIN
BRAIN, OLFACTORY BULB
BRAIN, PONS
BRAIN, THALAMUS
BRAIN, VENTRICULAR SYSTEM
BRAIN, WHITE MATTER
EYE
HEART
LUNG
LYMPH NODE, ILIAC
LYMPH NODE, MANDIBULAR
LYMPH NODE, MESENTERIC
MUSCLE, BICEPS FEMORIS
MUSCLE, GASTROCNEMIUS
NERVE ROOT, SPINAL, CERVICAL
NERVE ROOT, SPINAL, LUMBAR
NERVE ROOT, SPINAL, THORACIC
NERVE, OPTIC
NERVE, SCIATIC
NERVE, TIBIAL
PANCREAS
SPINAL CORD, CERVICAL
SPINAL CORD, LUMBAR
SPINAL CORD, THORACIC
SPLEEN
TESTIS

Appendix 10

Individual Animal Report

Study ID: 02776-0018: CRL 2954-001 / U of Tex Southwestern Med UTSW.Gray-002
Study Title: A SINGLE DOSE TOXICITY STUDY OF AAV9/SURF1 ADMINISTERED BY INTRATHECAL INJECTION IN RATS

Animal: 1014 (Male)

Sacrifice: DAY 08
Group: 1MD08 / VEHICLE (0 VG)
Fate: SCHEDULED SACRIFICE / Time on Test: 08 / Cause(s) of Death:

Gross or Microscopic Finding
Disposition
Tissue: Site / Finding, Severity

THYMUS

Disposition: **ABNORMAL/FINDING**

GANGLION, DORSAL ROOT, CERVICAL
GANGLION, DORSAL ROOT, CERVICAL, GLIAL CELL: HYPERTROPHY/HYPERPLASIA, MINIMAL
GANGLION, DORSAL ROOT, LUMBAR
GANGLION, DORSAL ROOT, LUMBAR, GLIAL CELL: HYPERTROPHY/HYPERPLASIA, MINIMAL
GANGLION, DORSAL ROOT, THORACIC
GANGLION, DORSAL ROOT, THORACIC, GLIAL CELL: HYPERTROPHY/HYPERPLASIA, MINIMAL
KIDNEY
KIDNEY: CHRONIC PROGRESSIVE NEPHROPATHY, MINIMAL
LIVER
LIVER: EXTRAMEDULLARY HEMATOPOIESIS, MINIMAL
LIVER: INFILTRATE, MIXED, MINIMAL

Appendix 10

Individual Animal Report

Study ID: 02776-0018: CRL 2954-001 / U of Tex Southwestern Med UTSW.Gray-002
Study Title: A SINGLE DOSE TOXICITY STUDY OF AAV9/SURF1 ADMINISTERED BY INTRATHECAL INJECTION IN RATS

Animal: 1015 (Male)

Sacrifice: DAY 08
Group: 1MD08 / VEHICLE (0 VG)
Fate: SCHEDULED SACRIFICE / Time on Test: 08 / Cause(s) of Death:

Gross or Microscopic Finding
Disposition
Tissue: Site / Finding, Severity

MICROSCOPIC EXAMINATION

Disposition: NORMAL

BRAIN, AMYGDALOID BODY
BRAIN, BASAL NUCLEI/STRIATUM
BRAIN, CEREBELLUM
BRAIN, CEREBRAL CORTEX
BRAIN, HIPPOCAMPUS
BRAIN, HYPOTHALAMUS
BRAIN, MEDULLA OBLONGATA
BRAIN, MENINGES
BRAIN, MIDBRAIN
BRAIN, OLFACTORY BULB
BRAIN, PONS
BRAIN, THALAMUS
BRAIN, VENTRICULAR SYSTEM
BRAIN, WHITE MATTER
EYE
GANGLION, DORSAL ROOT, CERVICAL
GANGLION, DORSAL ROOT, LUMBAR
GANGLION, DORSAL ROOT, THORACIC
HEART
LIVER
LUNG
LYMPH NODE, ILIAC
LYMPH NODE, MANDIBULAR
LYMPH NODE, MESENTERIC
MUSCLE, BICEPS FEMORIS
MUSCLE, GASTROCNEMIUS
NERVE ROOT, SPINAL, CERVICAL
NERVE ROOT, SPINAL, LUMBAR
NERVE ROOT, SPINAL, THORACIC
NERVE, OPTIC
NERVE, TIBIAL
PANCREAS
SPINAL CORD, CERVICAL
SPINAL CORD, LUMBAR

Appendix 10

Individual Animal Report

Study ID: 02776-0018: CRL 2954-001 / U of Tex Southwestern Med UTSW.Gray-002
Study Title: A SINGLE DOSE TOXICITY STUDY OF AAV9/SURF1 ADMINISTERED BY INTRATHECAL INJECTION IN RATS

Animal: 1015 (Male)

Sacrifice: DAY 08
Group: 1MD08 / VEHICLE (0 VG)
Fate: SCHEDULED SACRIFICE / Time on Test: 08 / Cause(s) of Death:

Gross or Microscopic Finding
Disposition
Tissue: Site / Finding, Severity

SPINAL CORD, THORACIC
SPLEEN
TESTIS
THYMUS

Disposition: ABNORMAL/FINDING

KIDNEY
KIDNEY: CHRONIC PROGRESSIVE NEPHROPATHY, MINIMAL
NERVE, SCIATIC
NERVE, SCIATIC: DEGENERATION, NERVE FIBER, MINIMAL

Appendix 10

Individual Animal Report

Study ID: 02776-0018: CRL 2954-001 / U of Tex Southwestern Med UTSW.Gray-002
Study Title: A SINGLE DOSE TOXICITY STUDY OF AAV9/SURF1 ADMINISTERED BY INTRATHECAL INJECTION IN RATS

Animal: 2011 (Male)

Sacrifice: DAY 08
Group: 2MD08 / AAV9/SURF1 LOW DOSE (0.28E12 VG)
Fate: SCHEDULED SACRIFICE / Time on Test: 08 / Cause(s) of Death:

Gross or Microscopic Finding
Disposition
Tissue: Site / Finding, Severity

MICROSCOPIC EXAMINATION

Disposition: NORMAL

BRAIN, AMYGDALOID BODY
BRAIN, BASAL NUCLEI/STRIATUM
BRAIN, CEREBELLUM
BRAIN, CEREBRAL CORTEX
BRAIN, HIPPOCAMPUS
BRAIN, HYPOTHALAMUS
BRAIN, MEDULLA OBLONGATA
BRAIN, MENINGES
BRAIN, MIDBRAIN
BRAIN, OLFACTORY BULB
BRAIN, PONS
BRAIN, THALAMUS
BRAIN, VENTRICULAR SYSTEM
BRAIN, WHITE MATTER
EYE
KIDNEY
LUNG
LYMPH NODE, ILIAC
LYMPH NODE, MANDIBULAR
LYMPH NODE, MESENTERIC
MUSCLE, BICEPS FEMORIS
MUSCLE, GASTROCNEMIUS
NERVE ROOT, SPINAL, CERVICAL
NERVE ROOT, SPINAL, LUMBAR
NERVE, OPTIC
NERVE, SCIATIC
NERVE, TIBIAL
PANCREAS
SPINAL CORD, CERVICAL
SPINAL CORD, LUMBAR
SPINAL CORD, THORACIC
SPLEEN
TESTIS
THYMUS

Appendix 10

Individual Animal Report

Study ID: 02776-0018: CRL 2954-001 / U of Tex Southwestern Med UTSW.Gray-002
Study Title: A SINGLE DOSE TOXICITY STUDY OF AAV9/SURF1 ADMINISTERED BY INTRATHECAL INJECTION IN RATS

Animal: 2011 (Male)

Sacrifice: DAY 08
Group: 2MD08 / AAV9/SURF1 LOW DOSE (0.28E12 VG)
Fate: SCHEDULED SACRIFICE / Time on Test: 08 / Cause(s) of Death:

Gross or Microscopic Finding
Disposition
Tissue: Site / Finding, Severity

Disposition: **ABNORMAL/FINDING**

GANGLION, DORSAL ROOT, CERVICAL
GANGLION, DORSAL ROOT, CERVICAL, GLIAL CELL: HYPERTROPHY/HYPERPLASIA, MINIMAL
GANGLION, DORSAL ROOT, LUMBAR
GANGLION, DORSAL ROOT, LUMBAR, GLIAL CELL: HYPERTROPHY/HYPERPLASIA, MINIMAL
GANGLION, DORSAL ROOT, THORACIC
GANGLION, DORSAL ROOT, THORACIC, GLIAL CELL: HYPERTROPHY/HYPERPLASIA, MINIMAL
HEART
HEART, MYOCARDIUM: DEGENERATION/NECROSIS, MINIMAL, MULTIFOCAL
HEART, MYOCARDIUM: INFILTRATE, MONONUCLEAR CELLS, MINIMAL, MULTIFOCAL
LIVER
LIVER: EXTRAMEDULLARY HEMATOPOIESIS, MINIMAL
LIVER: HYPERTROPHY/HYPERPLASIA, MINIMAL
COMMENT(S): AFFECTING KUPFFER CELLS
LIVER: INFILTRATE, MIXED, MINIMAL
NERVE ROOT, SPINAL, THORACIC
NERVE ROOT, SPINAL, THORACIC, NERVE ROOT, VENTRAL: DEGENERATION, NERVE FIBER, MINIMAL

Appendix 10

Individual Animal Report

Study ID: 02776-0018: CRL 2954-001 / U of Tex Southwestern Med UTSW.Gray-002
Study Title: A SINGLE DOSE TOXICITY STUDY OF AAV9/SURF1 ADMINISTERED BY INTRATHECAL INJECTION IN RATS

Animal: 2012 (Male)

Sacrifice: DAY 08
Group: 2MD08 / AAV9/SURF1 LOW DOSE (0.28E12 VG)
Fate: SCHEDULED SACRIFICE / Time on Test: 08 / Cause(s) of Death:

Gross or Microscopic Finding
Disposition
Tissue: Site / Finding, Severity

MICROSCOPIC EXAMINATION

Disposition: NORMAL

BRAIN, AMYGDALOID BODY
BRAIN, BASAL NUCLEI/STRIATUM
BRAIN, CEREBELLUM
BRAIN, CEREBRAL CORTEX
BRAIN, HIPPOCAMPUS
BRAIN, HYPOTHALAMUS
BRAIN, MEDULLA OBLONGATA
BRAIN, MENINGES
BRAIN, MIDBRAIN
BRAIN, OLFACTORY BULB
BRAIN, PONS
BRAIN, THALAMUS
BRAIN, VENTRICULAR SYSTEM
BRAIN, WHITE MATTER
EYE
GANGLION, DORSAL ROOT, THORACIC
KIDNEY
LUNG
LYMPH NODE, ILIAC
LYMPH NODE, MANDIBULAR
LYMPH NODE, MESENTERIC
MUSCLE, BICEPS FEMORIS
MUSCLE, GASTROCNEMIUS
NERVE ROOT, SPINAL, CERVICAL
NERVE ROOT, SPINAL, LUMBAR
NERVE ROOT, SPINAL, THORACIC
NERVE, OPTIC
NERVE, TIBIAL
PANCREAS
SPINAL CORD, CERVICAL
SPINAL CORD, LUMBAR
SPINAL CORD, THORACIC
SPLEEN
TESTIS

Individual Animal Report

Study ID: 02776-0018: CRL 2954-001 / U of Tex Southwestern Med UTSW.Gray-002
Study Title: A SINGLE DOSE TOXICITY STUDY OF AAV9/SURF1 ADMINISTERED BY INTRATHECAL INJECTION IN RATS

Animal: 2012 (Male)

Sacrifice: DAY 08
Group: 2MD08 / AAV9/SURF1 LOW DOSE (0.28E12 VG)
Fate: SCHEDULED SACRIFICE / Time on Test: 08 / Cause(s) of Death:

Gross or Microscopic Finding
Disposition
Tissue: Site / Finding, Severity

THYMUS

Disposition: **ABNORMAL/FINDING**

GANGLION, DORSAL ROOT, CERVICAL
GANGLION, DORSAL ROOT, CERVICAL, GLIAL CELL: HYPERTROPHY/HYPERPLASIA, MINIMAL
GANGLION, DORSAL ROOT, LUMBAR
GANGLION, DORSAL ROOT, LUMBAR, GLIAL CELL: HYPERTROPHY/HYPERPLASIA, MINIMAL
HEART
HEART, MYOCARDIUM: DEGENERATION/NECROSIS, MINIMAL, MULTIFOCAL
HEART, MYOCARDIUM: INFILTRATE, MONONUCLEAR CELLS, MINIMAL, MULTIFOCAL
LIVER
LIVER: EXTRAMEDULLARY HEMATOPOIESIS, MINIMAL
LIVER: INFILTRATE, MIXED, MINIMAL
NERVE, SCIATIC
NERVE, SCIATIC: DEGENERATION, NERVE FIBER, MINIMAL

Appendix 10

Individual Animal Report

Study ID: 02776-0018: CRL 2954-001 / U of Tex Southwestern Med UTSW.Gray-002
Study Title: A SINGLE DOSE TOXICITY STUDY OF AAV9/SURF1 ADMINISTERED BY INTRATHECAL INJECTION IN RATS

Animal: 2013 (Male)

Sacrifice: DAY 08
Group: 2MD08 / AAV9/SURF1 LOW DOSE (0.28E12 VG)
Fate: SCHEDULED SACRIFICE / Time on Test: 08 / Cause(s) of Death:

Gross or Microscopic Finding
Disposition
Tissue: Site / Finding, Severity

MICROSCOPIC EXAMINATION

Disposition: NORMAL

BRAIN, AMYGDALOID BODY
BRAIN, BASAL NUCLEI/STRIATUM
BRAIN, CEREBELLUM
BRAIN, CEREBRAL CORTEX
BRAIN, HIPPOCAMPUS
BRAIN, HYPOTHALAMUS
BRAIN, MEDULLA OBLONGATA
BRAIN, MENINGES
BRAIN, MIDBRAIN
BRAIN, OLFACTORY BULB
BRAIN, PONS
BRAIN, THALAMUS
BRAIN, VENTRICULAR SYSTEM
BRAIN, WHITE MATTER
EYE
GANGLION, DORSAL ROOT, THORACIC
KIDNEY
LUNG
LYMPH NODE, ILIAC
LYMPH NODE, MANDIBULAR
LYMPH NODE, MESENTERIC
MUSCLE, BICEPS FEMORIS
MUSCLE, GASTROCNEMIUS
NERVE ROOT, SPINAL, CERVICAL
NERVE ROOT, SPINAL, LUMBAR
NERVE ROOT, SPINAL, THORACIC
NERVE, OPTIC
PANCREAS
SPINAL CORD, CERVICAL
SPINAL CORD, LUMBAR
SPINAL CORD, THORACIC
SPLEEN
TESTIS
THYMUS

Appendix 10

Individual Animal Report

Study ID: 02776-0018: CRL 2954-001 / U of Tex Southwestern Med UTSW.Gray-002
Study Title: A SINGLE DOSE TOXICITY STUDY OF AAV9/SURF1 ADMINISTERED BY INTRATHECAL INJECTION IN RATS

Animal: 2013 (Male)

Sacrifice: DAY 08
Group: 2MD08 / AAV9/SURF1 LOW DOSE (0.28E12 VG)
Fate: SCHEDULED SACRIFICE / Time on Test: 08 / Cause(s) of Death:

Gross or Microscopic Finding
Disposition
Tissue: Site / Finding, Severity

Disposition: **ABNORMAL/FINDING**

GANGLION, DORSAL ROOT, CERVICAL
GANGLION, DORSAL ROOT, CERVICAL, GLIAL CELL: HYPERTROPHY/HYPERPLASIA, MINIMAL
GANGLION, DORSAL ROOT, LUMBAR
GANGLION, DORSAL ROOT, LUMBAR, GLIAL CELL: HYPERTROPHY/HYPERPLASIA, MINIMAL
HEART
HEART, MYOCARDIUM: DEGENERATION/NECROSIS, MINIMAL, FOCAL
HEART, MYOCARDIUM: INFILTRATE, MONONUCLEAR CELLS, MINIMAL, FOCAL
LIVER
LIVER: EXTRAMEDULLARY HEMATOPOIESIS, MINIMAL
LIVER: INFILTRATE, MIXED, MINIMAL
NERVE, SCIATIC
NERVE, SCIATIC: DEGENERATION, NERVE FIBER, MINIMAL
NERVE, TIBIAL
NERVE, TIBIAL: DEGENERATION, NERVE FIBER, MINIMAL

Appendix 10

Individual Animal Report

Study ID: 02776-0018: CRL 2954-001 / U of Tex Southwestern Med UTSW.Gray-002
Study Title: A SINGLE DOSE TOXICITY STUDY OF AAV9/SURF1 ADMINISTERED BY INTRATHECAL INJECTION IN RATS

Animal: 2014 (Male)

Sacrifice: DAY 08
Group: 2MD08 / AAV9/SURF1 LOW DOSE (0.28E12 VG)
Fate: SCHEDULED SACRIFICE / Time on Test: 08 / Cause(s) of Death:

Gross or Microscopic Finding
Disposition
Tissue: Site / Finding, Severity

MICROSCOPIC EXAMINATION

Disposition: NORMAL

BRAIN, AMYGDALOID BODY
BRAIN, BASAL NUCLEI/STRIATUM
BRAIN, CEREBELLUM
BRAIN, CEREBRAL CORTEX
BRAIN, HIPPOCAMPUS
BRAIN, HYPOTHALAMUS
BRAIN, MEDULLA OBLONGATA
BRAIN, MENINGES
BRAIN, MIDBRAIN
BRAIN, OLFACTORY BULB
BRAIN, PONS
BRAIN, THALAMUS
BRAIN, VENTRICULAR SYSTEM
BRAIN, WHITE MATTER
EYE
GANGLION, DORSAL ROOT, CERVICAL
GANGLION, DORSAL ROOT, THORACIC
LUNG
LYMPH NODE, ILIAC
LYMPH NODE, MANDIBULAR
LYMPH NODE, MESENTERIC
MUSCLE, BICEPS FEMORIS
MUSCLE, GASTROCNEMIUS
NERVE ROOT, SPINAL, CERVICAL
NERVE ROOT, SPINAL, THORACIC
NERVE, OPTIC
NERVE, TIBIAL
PANCREAS
SPINAL CORD, CERVICAL
SPINAL CORD, LUMBAR
SPINAL CORD, THORACIC
SPLEEN
TESTIS
THYMUS

Appendix 10

Individual Animal Report

Study ID: 02776-0018: CRL 2954-001 / U of Tex Southwestern Med UTSW.Gray-002
Study Title: A SINGLE DOSE TOXICITY STUDY OF AAV9/SURF1 ADMINISTERED BY INTRATHECAL INJECTION IN RATS

Animal: 2014 (Male)

Sacrifice: DAY 08
Group: 2MD08 / AAV9/SURF1 LOW DOSE (0.28E12 VG)
Fate: SCHEDULED SACRIFICE / Time on Test: 08 / Cause(s) of Death:

Gross or Microscopic Finding
Disposition
Tissue: Site / Finding, Severity

Disposition: ABNORMAL/FINDING

GANGLION, DORSAL ROOT, LUMBAR
GANGLION, DORSAL ROOT, LUMBAR: INFILTRATE, MONONUCLEAR CELLS, MINIMAL, FOCAL
HEART
HEART, MYOCARDIUM: DEGENERATION/NECROSIS, MINIMAL
HEART, MYOCARDIUM: INFILTRATE, MONONUCLEAR CELLS, MINIMAL
KIDNEY
KIDNEY: CHRONIC PROGRESSIVE NEPHROPATHY, MINIMAL
LIVER
LIVER: EXTRAMEDULLARY HEMATOPOIESIS, MINIMAL
LIVER: INFILTRATE, MIXED, MINIMAL
NERVE ROOT, SPINAL, LUMBAR
NERVE ROOT, SPINAL, LUMBAR, EPINEURIUM: INFILTRATE, MONONUCLEAR CELLS, MINIMAL, FOCAL
NERVE, SCIATIC
NERVE, SCIATIC: DEGENERATION, NERVE FIBER, MINIMAL

Appendix 10

Individual Animal Report

Study ID: 02776-0018: CRL 2954-001 / U of Tex Southwestern Med UTSW.Gray-002
Study Title: A SINGLE DOSE TOXICITY STUDY OF AAV9/SURF1 ADMINISTERED BY INTRATHECAL INJECTION IN RATS

Animal: 2015 (Male)

Sacrifice: DAY 08
Group: 2MD08 / AAV9/SURF1 LOW DOSE (0.28E12 VG)
Fate: SCHEDULED SACRIFICE / Time on Test: 08 / Cause(s) of Death:

Gross or Microscopic Finding
Disposition
Tissue: Site / Finding, Severity

MICROSCOPIC EXAMINATION

Disposition: NORMAL

BRAIN, AMYGDALOID BODY
BRAIN, BASAL NUCLEI/STRIATUM
BRAIN, CEREBELLUM
BRAIN, CEREBRAL CORTEX
BRAIN, HIPPOCAMPUS
BRAIN, HYPOTHALAMUS
BRAIN, MEDULLA OBLONGATA
BRAIN, MENINGES
BRAIN, MIDBRAIN
BRAIN, OLFACTORY BULB
BRAIN, PONS
BRAIN, THALAMUS
BRAIN, VENTRICULAR SYSTEM
BRAIN, WHITE MATTER
EYE
GANGLION, DORSAL ROOT, CERVICAL
GANGLION, DORSAL ROOT, THORACIC
LUNG
LYMPH NODE, ILIAC
LYMPH NODE, MANDIBULAR
LYMPH NODE, MESENTERIC
MUSCLE, BICEPS FEMORIS
MUSCLE, GASTROCNEMIUS
NERVE ROOT, SPINAL, CERVICAL
NERVE ROOT, SPINAL, LUMBAR
NERVE ROOT, SPINAL, THORACIC
NERVE, OPTIC
NERVE, SCIATIC
NERVE, TIBIAL
PANCREAS
SPINAL CORD, CERVICAL
SPINAL CORD, THORACIC
SPLEEN
TESTIS

Appendix 10

Individual Animal Report

Study ID: 02776-0018: CRL 2954-001 / U of Tex Southwestern Med UTSW.Gray-002
Study Title: A SINGLE DOSE TOXICITY STUDY OF AAV9/SURF1 ADMINISTERED BY INTRATHECAL INJECTION IN RATS

Animal: 2015 (Male)

Sacrifice: DAY 08
Group: 2MD08 / AAV9/SURF1 LOW DOSE (0.28E12 VG)
Fate: SCHEDULED SACRIFICE / Time on Test: 08 / Cause(s) of Death:

Gross or Microscopic Finding
Disposition
Tissue: Site / Finding, Severity

THYMUS

Disposition: **ABNORMAL/FINDING**

GANGLION, DORSAL ROOT, LUMBAR
GANGLION, DORSAL ROOT, LUMBAR, GLIAL CELL: HYPERTROPHY/HYPERPLASIA, MINIMAL
HEART
HEART, MYOCARDIUM: DEGENERATION/NECROSIS, MINIMAL
HEART, MYOCARDIUM: INFILTRATE, MONONUCLEAR CELLS, MINIMAL
KIDNEY
KIDNEY, TUBULAR: CYST, MINIMAL
LIVER
LIVER: EXTRAMEDULLARY HEMATOPOIESIS, MINIMAL
LIVER: INFILTRATE, MIXED, MINIMAL
SPINAL CORD, LUMBAR
SPINAL CORD, LUMBAR, WHITE MATTER: DEGENERATION, NERVE FIBER, MINIMAL
COMMENT(S): SEEN IN LONG SECTION AND EXACT LOCATION COULD NOT BE DETERMINED

Appendix 10

Individual Animal Report

Study ID: 02776-0018: CRL 2954-001 / U of Tex Southwestern Med UTSW.Gray-002
Study Title: A SINGLE DOSE TOXICITY STUDY OF AAV9/SURF1 ADMINISTERED BY INTRATHECAL INJECTION IN RATS

Animal: 3011 (Male)

Sacrifice: DAY 08
Group: 3MD08 / AAV9/SURF1 MID DOSE (0.83E12 VG)
Fate: SCHEDULED SACRIFICE / Time on Test: 08 / Cause(s) of Death:

Gross or Microscopic Finding
Disposition
Tissue: Site / Finding, Severity

MICROSCOPIC EXAMINATION

Disposition: **NORMAL**

BRAIN, AMYGDALOID BODY
BRAIN, BASAL NUCLEI/STRIATUM
BRAIN, CEREBELLUM
BRAIN, CEREBRAL CORTEX
BRAIN, HIPPOCAMPUS
BRAIN, HYPOTHALAMUS
BRAIN, MEDULLA OBLONGATA
BRAIN, MENINGES
BRAIN, MIDBRAIN
BRAIN, OLFACTORY BULB
BRAIN, PONS
BRAIN, THALAMUS
BRAIN, VENTRICULAR SYSTEM
BRAIN, WHITE MATTER
EYE
GANGLION, DORSAL ROOT, CERVICAL
GANGLION, DORSAL ROOT, THORACIC
HEART
LUNG
LYMPH NODE, ILIAC
LYMPH NODE, MANDIBULAR
LYMPH NODE, MESENTERIC
MUSCLE, BICEPS FEMORIS
MUSCLE, GASTROCNEMIUS
NERVE ROOT, SPINAL, CERVICAL
NERVE ROOT, SPINAL, THORACIC
NERVE, OPTIC
NERVE, TIBIAL
PANCREAS
SPINAL CORD, CERVICAL
SPINAL CORD, LUMBAR
SPINAL CORD, THORACIC
SPLEEN
TESTIS

Appendix 10

Individual Animal Report

Study ID: 02776-0018: CRL 2954-001 / U of Tex Southwestern Med UTSW.Gray-002
Study Title: A SINGLE DOSE TOXICITY STUDY OF AAV9/SURF1 ADMINISTERED BY INTRATHECAL INJECTION IN RATS

Animal: 3011 (Male)

Sacrifice: DAY 08
Group: 3MD08 / AAV9/SURF1 MID DOSE (0.83E12 VG)
Fate: SCHEDULED SACRIFICE / Time on Test: 08 / Cause(s) of Death:

Gross or Microscopic Finding
Disposition
Tissue: Site / Finding, Severity

THYMUS

Disposition: **ABNORMAL/FINDING**

GANGLION, DORSAL ROOT, LUMBAR
GANGLION, DORSAL ROOT, LUMBAR: INFILTRATE, MONONUCLEAR CELLS, MINIMAL, FOCAL
GANGLION, DORSAL ROOT, LUMBAR, GLIAL CELL: HYPERTROPHY/HYPERPLASIA, MINIMAL
KIDNEY
KIDNEY: CHRONIC PROGRESSIVE NEPHROPATHY, MINIMAL
LIVER
LIVER: INFILTRATE, MIXED, MINIMAL
NERVE ROOT, SPINAL, LUMBAR
NERVE ROOT, SPINAL, LUMBAR, EPINEURIUM: INFILTRATE, MONONUCLEAR CELLS, MINIMAL, FOCAL
NERVE, SCIATIC
NERVE, SCIATIC: DEGENERATION, NERVE FIBER, MINIMAL

Appendix 10

Individual Animal Report

Study ID: 02776-0018: CRL 2954-001 / U of Tex Southwestern Med UTSW.Gray-002
Study Title: A SINGLE DOSE TOXICITY STUDY OF AAV9/SURF1 ADMINISTERED BY INTRATHECAL INJECTION IN RATS

Animal: 3012 (Male)

Sacrifice: DAY 08
Group: 3MD08 / AAV9/SURF1 MID DOSE (0.83E12 VG)
Fate: SCHEDULED SACRIFICE / Time on Test: 08 / Cause(s) of Death:

Gross or Microscopic Finding
Disposition
Tissue: Site / Finding, Severity

MICROSCOPIC EXAMINATION

Disposition: **NORMAL**

BRAIN, AMYGDALOID BODY
BRAIN, BASAL NUCLEI/STRIATUM
BRAIN, CEREBELLUM
BRAIN, CEREBRAL CORTEX
BRAIN, HIPPOCAMPUS
BRAIN, HYPOTHALAMUS
BRAIN, MEDULLA OBLONGATA
BRAIN, MENINGES
BRAIN, MIDBRAIN
BRAIN, OLFACTORY BULB
BRAIN, PONS
BRAIN, THALAMUS
BRAIN, VENTRICULAR SYSTEM
BRAIN, WHITE MATTER
EYE
GANGLION, DORSAL ROOT, LUMBAR
GANGLION, DORSAL ROOT, THORACIC
LUNG
LYMPH NODE, ILIAC
LYMPH NODE, MANDIBULAR
LYMPH NODE, MESENTERIC
MUSCLE, BICEPS FEMORIS
MUSCLE, GASTROCNEMIUS
NERVE ROOT, SPINAL, CERVICAL
NERVE ROOT, SPINAL, LUMBAR
NERVE ROOT, SPINAL, THORACIC
NERVE, OPTIC
NERVE, TIBIAL
PANCREAS
SPINAL CORD, CERVICAL
SPINAL CORD, LUMBAR
SPINAL CORD, THORACIC
SPLEEN
TESTIS

Appendix 10

Individual Animal Report

Study ID: 02776-0018: CRL 2954-001 / U of Tex Southwestern Med UTSW.Gray-002
Study Title: A SINGLE DOSE TOXICITY STUDY OF AAV9/SURF1 ADMINISTERED BY INTRATHECAL INJECTION IN RATS

Animal: 3012 (Male)

Sacrifice: DAY 08
Group: 3MD08 / AAV9/SURF1 MID DOSE (0.83E12 VG)
Fate: SCHEDULED SACRIFICE / Time on Test: 08 / Cause(s) of Death:

Gross or Microscopic Finding
Disposition
Tissue: Site / Finding, Severity

THYMUS

Disposition: **ABNORMAL/FINDING**

GANGLION, DORSAL ROOT, CERVICAL
GANGLION, DORSAL ROOT, CERVICAL: INFILTRATE, MONONUCLEAR CELLS, MINIMAL, FOCAL
HEART
HEART, MYOCARDIUM: DEGENERATION/NECROSIS, MINIMAL
HEART, MYOCARDIUM: INFILTRATE, MONONUCLEAR CELLS, MINIMAL
KIDNEY
KIDNEY, TUBULAR: BASOPHILIA, MINIMAL
KIDNEY, TUBULAR: CYST, MINIMAL
LIVER
LIVER: EXTRAMEDULLARY HEMATOPOIESIS, MINIMAL
LIVER: INFILTRATE, MIXED, MINIMAL
NERVE, SCIATIC
NERVE, SCIATIC: DEGENERATION, NERVE FIBER, MINIMAL

Appendix 10

Individual Animal Report

Study ID: 02776-0018: CRL 2954-001 / U of Tex Southwestern Med UTSW.Gray-002
Study Title: A SINGLE DOSE TOXICITY STUDY OF AAV9/SURF1 ADMINISTERED BY INTRATHECAL INJECTION IN RATS

Animal: 3013 (Male)

Sacrifice: DAY 08
Group: 3MD08 / AAV9/SURF1 MID DOSE (0.83E12 VG)
Fate: SCHEDULED SACRIFICE / Time on Test: 08 / Cause(s) of Death:

Gross or Microscopic Finding
Disposition
Tissue: Site / Finding, Severity

MICROSCOPIC EXAMINATION

Disposition: NORMAL

BRAIN, AMYGDALOID BODY
BRAIN, BASAL NUCLEI/STRIATUM
BRAIN, CEREBELLUM
BRAIN, CEREBRAL CORTEX
BRAIN, HIPPOCAMPUS
BRAIN, HYPOTHALAMUS
BRAIN, MEDULLA OBLONGATA
BRAIN, MENINGES
BRAIN, MIDBRAIN
BRAIN, PONS
BRAIN, THALAMUS
BRAIN, VENTRICULAR SYSTEM
BRAIN, WHITE MATTER
EYE
GANGLION, DORSAL ROOT, CERVICAL
GANGLION, DORSAL ROOT, THORACIC
KIDNEY
LUNG
LYMPH NODE, ILIAC
LYMPH NODE, MANDIBULAR
LYMPH NODE, MESENTERIC
MUSCLE, BICEPS FEMORIS
MUSCLE, GASTROCNEMIUS
NERVE ROOT, SPINAL, CERVICAL
NERVE ROOT, SPINAL, THORACIC
NERVE, OPTIC
NERVE, TIBIAL
PANCREAS
SPINAL CORD, CERVICAL
SPINAL CORD, LUMBAR
SPINAL CORD, THORACIC
SPLEEN
TESTIS
THYMUS

Appendix 10

Individual Animal Report

Study ID: 02776-0018: CRL 2954-001 / U of Tex Southwestern Med UTSW.Gray-002
Study Title: A SINGLE DOSE TOXICITY STUDY OF AAV9/SURF1 ADMINISTERED BY INTRATHECAL INJECTION IN RATS

Animal: 3013 (Male)

Sacrifice: DAY 08
Group: 3MD08 / AAV9/SURF1 MID DOSE (0.83E12 VG)
Fate: SCHEDULED SACRIFICE / Time on Test: 08 / Cause(s) of Death:

Gross or Microscopic Finding
Disposition
Tissue: Site / Finding, Severity

Disposition: ABNORMAL/FINDING

GANGLION, DORSAL ROOT, LUMBAR
GANGLION, DORSAL ROOT, LUMBAR, GLIAL CELL: HYPERTROPHY/HYPERPLASIA, MINIMAL
HEART
HEART, MYOCARDIUM: INFILTRATE, MONONUCLEAR CELLS, MINIMAL, FOCAL
LIVER
LIVER: EXTRAMEDULLARY HEMATOPOIESIS, MINIMAL
LIVER: HYPERTROPHY/HYPERPLASIA, MINIMAL
COMMENT(S): AFFECTING KUPFFER CELLS
LIVER: INFILTRATE, MIXED, MINIMAL
LIVER, HEPATOCYTE: NECROSIS, SINGLE CELL, MINIMAL
NERVE ROOT, SPINAL, LUMBAR
NERVE ROOT, SPINAL, LUMBAR, EPINEURIUM: INFILTRATE, MONONUCLEAR CELLS, MINIMAL, FOCAL
NERVE, SCIATIC
NERVE, SCIATIC: DEGENERATION, NERVE FIBER, MINIMAL

Disposition: UNABLE TO OBTAIN SPECIMEN

BRAIN, OLFACTORY BULB

Appendix 10

Individual Animal Report

Study ID: 02776-0018: CRL 2954-001 / U of Tex Southwestern Med UTSW.Gray-002
Study Title: A SINGLE DOSE TOXICITY STUDY OF AAV9/SURF1 ADMINISTERED BY INTRATHECAL INJECTION IN RATS

Animal: 3014 (Male)

Sacrifice: DAY 08
Group: 3MD08 / AAV9/SURF1 MID DOSE (0.83E12 VG)
Fate: SCHEDULED SACRIFICE / Time on Test: 08 / Cause(s) of Death:

Gross or Microscopic Finding
Disposition
Tissue: Site / Finding, Severity

MICROSCOPIC EXAMINATION

Disposition: NORMAL

BRAIN, AMYGDALOID BODY
BRAIN, BASAL NUCLEI/STRIATUM
BRAIN, CEREBELLUM
BRAIN, CEREBRAL CORTEX
BRAIN, HIPPOCAMPUS
BRAIN, HYPOTHALAMUS
BRAIN, MEDULLA OBLONGATA
BRAIN, MENINGES
BRAIN, MIDBRAIN
BRAIN, OLFACTORY BULB
BRAIN, PONS
BRAIN, THALAMUS
BRAIN, VENTRICULAR SYSTEM
BRAIN, WHITE MATTER
EYE
GANGLION, DORSAL ROOT, CERVICAL
GANGLION, DORSAL ROOT, THORACIC
LUNG
LYMPH NODE, ILIAC
LYMPH NODE, MANDIBULAR
LYMPH NODE, MESENTERIC
MUSCLE, BICEPS FEMORIS
MUSCLE, GASTROCNEMIUS
NERVE ROOT, SPINAL, CERVICAL
NERVE ROOT, SPINAL, LUMBAR
NERVE ROOT, SPINAL, THORACIC
NERVE, OPTIC
PANCREAS
SPINAL CORD, CERVICAL
SPINAL CORD, LUMBAR
SPLEEN
TESTIS
THYMUS

Appendix 10

Individual Animal Report

Study ID: 02776-0018: CRL 2954-001 / U of Tex Southwestern Med UTSW.Gray-002
Study Title: A SINGLE DOSE TOXICITY STUDY OF AAV9/SURF1 ADMINISTERED BY INTRATHECAL INJECTION IN RATS

Animal: 3014 (Male)

Sacrifice: DAY 08
Group: 3MD08 / AAV9/SURF1 MID DOSE (0.83E12 VG)
Fate: SCHEDULED SACRIFICE / Time on Test: 08 / Cause(s) of Death:

Gross or Microscopic Finding
Disposition
Tissue: Site / Finding, Severity

Disposition: ABNORMAL/FINDING

GANGLION, DORSAL ROOT, LUMBAR
GANGLION, DORSAL ROOT, LUMBAR, GLIAL CELL: HYPERTROPHY/HYPERPLASIA, MINIMAL
HEART
HEART, MYOCARDIUM: DEGENERATION/NECROSIS, MINIMAL, MULTIFOCAL
HEART, MYOCARDIUM: INFILTRATE, MONONUCLEAR CELLS, MINIMAL, MULTIFOCAL
KIDNEY
KIDNEY, TUBULAR: BASOPHILIA, MINIMAL
LIVER
LIVER: INFILTRATE, MIXED, MINIMAL
NERVE, SCIATIC
NERVE, SCIATIC: DEGENERATION, NERVE FIBER, MINIMAL
SPINAL CORD, THORACIC
SPINAL CORD, THORACIC, WHITE MATTER: DEGENERATION, NERVE FIBER, MINIMAL
COMMENT(S): CHANGE DEFINITIVE TO DORSAL TRACT SEEN IN CROSS SECTION AND CHANGE SEEN IN LONG SECTION WAS OF UNDETERMINED LOCATION (VENTRAL OR DORSAL)

Disposition: UNABLE TO OBTAIN SPECIMEN

NERVE, TIBIAL

Appendix 10

Individual Animal Report

Study ID: 02776-0018: CRL 2954-001 / U of Tex Southwestern Med UTSW.Gray-002
Study Title: A SINGLE DOSE TOXICITY STUDY OF AAV9/SURF1 ADMINISTERED BY INTRATHECAL INJECTION IN RATS

Animal: 3015 (Male)

Sacrifice: DAY 08
Group: 3MD08 / AAV9/SURF1 MID DOSE (0.83E12 VG)
Fate: SCHEDULED SACRIFICE / Time on Test: 08 / Cause(s) of Death:

Gross or Microscopic Finding
Disposition
Tissue: Site / Finding, Severity

MICROSCOPIC EXAMINATION

Disposition: NORMAL

BRAIN, AMYGDALOID BODY
BRAIN, BASAL NUCLEI/STRIATUM
BRAIN, CEREBELLUM
BRAIN, CEREBRAL CORTEX
BRAIN, HIPPOCAMPUS
BRAIN, HYPOTHALAMUS
BRAIN, MEDULLA OBLONGATA
BRAIN, MENINGES
BRAIN, MIDBRAIN
BRAIN, OLFACTORY BULB
BRAIN, PONS
BRAIN, THALAMUS
BRAIN, VENTRICULAR SYSTEM
BRAIN, WHITE MATTER
EYE
GANGLION, DORSAL ROOT, CERVICAL
GANGLION, DORSAL ROOT, LUMBAR
GANGLION, DORSAL ROOT, THORACIC
LYMPH NODE, ILIAC
LYMPH NODE, MANDIBULAR
LYMPH NODE, MESENTERIC
MUSCLE, BICEPS FEMORIS
MUSCLE, GASTROCNEMIUS
NERVE ROOT, SPINAL, CERVICAL
NERVE ROOT, SPINAL, LUMBAR
NERVE ROOT, SPINAL, THORACIC
NERVE, OPTIC
NERVE, SCIATIC
NERVE, TIBIAL
PANCREAS
SPINAL CORD, CERVICAL
SPINAL CORD, LUMBAR
SPINAL CORD, THORACIC
SPLEEN

Appendix 10

Individual Animal Report

Study ID: 02776-0018: CRL 2954-001 / U of Tex Southwestern Med UTSW.Gray-002
Study Title: A SINGLE DOSE TOXICITY STUDY OF AAV9/SURF1 ADMINISTERED BY INTRATHECAL INJECTION IN RATS

Animal: 3015 (Male)

Sacrifice: DAY 08
Group: 3MD08 / AAV9/SURF1 MID DOSE (0.83E12 VG)
Fate: SCHEDULED SACRIFICE / Time on Test: 08 / Cause(s) of Death:

Gross or Microscopic Finding
Disposition
Tissue: Site / Finding, Severity

TESTIS
THYMUS

Disposition: **ABNORMAL/FINDING**

HEART
HEART, MYOCARDIUM: DEGENERATION/NECROSIS, MINIMAL
HEART, MYOCARDIUM: INFILTRATE, MONONUCLEAR CELLS, MINIMAL
KIDNEY
KIDNEY: INFILTRATE, MONONUCLEAR CELLS, MINIMAL
LIVER
LIVER: INFILTRATE, MIXED, MINIMAL
LUNG
LUNG: INFLAMMATION, MINIMAL, SUBACUTE, FOCAL
COMMENT(S): ASSOCIATED WITH BRIGHT EOSINOPHILIC ACELLULAR ELONGATE CRYSTALLINE MATERIAL.

Appendix 10

Individual Animal Report

Study ID: 02776-0018: CRL 2954-001 / U of Tex Southwestern Med UTSW.Gray-002
Study Title: A SINGLE DOSE TOXICITY STUDY OF AAV9/SURF1 ADMINISTERED BY INTRATHECAL INJECTION IN RATS

Animal: 4013 (Male)

Sacrifice: DAY 08
Group: 3MD08 / AAV9/SURF1 MID DOSE (0.83E12 VG)
Fate: SCHEDULED SACRIFICE / Time on Test: 08 / Cause(s) of Death:

Gross or Microscopic Finding
Disposition
Tissue: Site / Finding, Severity

MICROSCOPIC EXAMINATION

Disposition: NORMAL

BRAIN, AMYGDALOID BODY
BRAIN, BASAL NUCLEI/STRIATUM
BRAIN, CEREBELLUM
BRAIN, CEREBRAL CORTEX
BRAIN, HIPPOCAMPUS
BRAIN, HYPOTHALAMUS
BRAIN, MEDULLA OBLONGATA
BRAIN, MENINGES
BRAIN, MIDBRAIN
BRAIN, OLFACTORY BULB
BRAIN, PONS
BRAIN, THALAMUS
BRAIN, VENTRICULAR SYSTEM
BRAIN, WHITE MATTER
EYE
GANGLION, DORSAL ROOT, CERVICAL
GANGLION, DORSAL ROOT, THORACIC
HEART
LUNG
LYMPH NODE, ILIAC
LYMPH NODE, MANDIBULAR
LYMPH NODE, MESENTERIC
MUSCLE, BICEPS FEMORIS
MUSCLE, GASTROCNEMIUS
NERVE ROOT, SPINAL, CERVICAL
NERVE ROOT, SPINAL, THORACIC
NERVE, OPTIC
NERVE, TIBIAL
PANCREAS
SPINAL CORD, CERVICAL
SPINAL CORD, THORACIC
TESTIS
THYMUS

Appendix 10

Individual Animal Report

Study ID: 02776-0018: CRL 2954-001 / U of Tex Southwestern Med UTSW.Gray-002
Study Title: A SINGLE DOSE TOXICITY STUDY OF AAV9/SURF1 ADMINISTERED BY INTRATHECAL INJECTION IN RATS

Animal: 4013 (Male)

Sacrifice: DAY 08
Group: 3MD08 / AAV9/SURF1 MID DOSE (0.83E12 VG)
Fate: SCHEDULED SACRIFICE / Time on Test: 08 / Cause(s) of Death:

Gross or Microscopic Finding
Disposition
Tissue: Site / Finding, Severity

Disposition: **ABNORMAL/FINDING**

GANGLION, DORSAL ROOT, LUMBAR
GANGLION, DORSAL ROOT, LUMBAR: INFILTRATE, MONONUCLEAR CELLS, MINIMAL, FOCAL
KIDNEY
KIDNEY: CHRONIC PROGRESSIVE NEPHROPATHY, MINIMAL
LIVER
LIVER: EXTRAMEDULLARY HEMATOPOIESIS, MINIMAL
LIVER: INFILTRATE, MIXED, MINIMAL
NERVE ROOT, SPINAL, LUMBAR
NERVE ROOT, SPINAL, LUMBAR, EPINEURIUM: INFILTRATE, MONONUCLEAR CELLS, MINIMAL, FOCAL
NERVE, SCIATIC
NERVE, SCIATIC: DEGENERATION, NERVE FIBER, MINIMAL
SPINAL CORD, LUMBAR
SPINAL CORD, LUMBAR, NERVE ROOT, SPINAL: DEGENERATION, NERVE FIBER, MINIMAL
SPLEEN
SPLEEN: EXTRAMEDULLARY HEMATOPOIESIS, MINIMAL

Appendix 10

Individual Animal Report

Study ID: 02776-0018: CRL 2954-001 / U of Tex Southwestern Med UTSW.Gray-002
Study Title: A SINGLE DOSE TOXICITY STUDY OF AAV9/SURF1 ADMINISTERED BY INTRATHECAL INJECTION IN RATS

Animal: 4011 (Male)

Sacrifice: DAY 08
Group: 4MD08 / AAV9/SURF1 HIGH DOSE (2.49E12 VG)
Fate: SCHEDULED SACRIFICE / Time on Test: 08 / Cause(s) of Death:

Gross or Microscopic Finding
Disposition
Tissue: Site / Finding, Severity

MICROSCOPIC EXAMINATION

Disposition: NORMAL

BRAIN, AMYGDALOID BODY
BRAIN, BASAL NUCLEI/STRIATUM
BRAIN, CEREBELLUM
BRAIN, CEREBRAL CORTEX
BRAIN, HIPPOCAMPUS
BRAIN, HYPOTHALAMUS
BRAIN, MEDULLA OBLONGATA
BRAIN, MENINGES
BRAIN, MIDBRAIN
BRAIN, OLFACTORY BULB
BRAIN, PONS
BRAIN, THALAMUS
BRAIN, VENTRICULAR SYSTEM
BRAIN, WHITE MATTER
EYE
GANGLION, DORSAL ROOT, CERVICAL
GANGLION, DORSAL ROOT, LUMBAR
GANGLION, DORSAL ROOT, THORACIC
HEART
LUNG
LYMPH NODE, ILIAC
LYMPH NODE, MANDIBULAR
LYMPH NODE, MESENTERIC
MUSCLE, GASTROCNEMIUS
NERVE ROOT, SPINAL, CERVICAL
NERVE ROOT, SPINAL, LUMBAR
NERVE ROOT, SPINAL, THORACIC
NERVE, OPTIC
NERVE, SCIATIC
NERVE, TIBIAL
SPINAL CORD, CERVICAL
SPINAL CORD, LUMBAR
SPLEEN
TESTIS

Appendix 10

Individual Animal Report

Study ID: 02776-0018: CRL 2954-001 / U of Tex Southwestern Med UTSW.Gray-002
Study Title: A SINGLE DOSE TOXICITY STUDY OF AAV9/SURF1 ADMINISTERED BY INTRATHECAL INJECTION IN RATS

Animal: 4011 (Male)

Sacrifice: DAY 08
Group: 4MD08 / AAV9/SURF1 HIGH DOSE (2.49E12 VG)
Fate: SCHEDULED SACRIFICE / Time on Test: 08 / Cause(s) of Death:

Gross or Microscopic Finding
Disposition
Tissue: Site / Finding, Severity

THYMUS

Disposition: **ABNORMAL/FINDING**

KIDNEY
KIDNEY, TUBULAR: BASOPHILIA, MINIMAL
LIVER
LIVER: EXTRAMEDULLARY HEMATOPOIESIS, MINIMAL
LIVER: HYPERTROPHY/HYPERPLASIA, MINIMAL
COMMENT(S): AFFECTING KUPFFER CELLS
LIVER: INFILTRATE, MIXED, MINIMAL
LIVER: MITOTIC FIGURES, INCREASED, MINIMAL
LIVER, HEPATOCYTE: NECROSIS, SINGLE CELL, MILD
MUSCLE, BICEPS FEMORIS
MUSCLE, BICEPS FEMORIS: DEGENERATION/NECROSIS, MINIMAL, MULTIFOCAL
PANCREAS
PANCREAS: APOPTOSIS/SINGLE CELL NECROSIS, MINIMAL
PANCREAS: MITOTIC FIGURES, INCREASED, MINIMAL
SPINAL CORD, THORACIC
SPINAL CORD, THORACIC, MENINGES: INFILTRATE, MONONUCLEAR CELLS, MINIMAL, MULTIFOCAL

Appendix 10

Individual Animal Report

Study ID: 02776-0018: CRL 2954-001 / U of Tex Southwestern Med UTSW.Gray-002
Study Title: A SINGLE DOSE TOXICITY STUDY OF AAV9/SURF1 ADMINISTERED BY INTRATHECAL INJECTION IN RATS

Animal: 4012 (Male)

Sacrifice: DAY 08
Group: 4MD08 / AAV9/SURF1 HIGH DOSE (2.49E12 VG)
Fate: SCHEDULED SACRIFICE / Time on Test: 08 / Cause(s) of Death:

Gross or Microscopic Finding
Disposition
Tissue: Site / Finding, Severity

MICROSCOPIC EXAMINATION

Disposition: NORMAL

BRAIN, AMYGDALOID BODY
BRAIN, BASAL NUCLEI/STRIATUM
BRAIN, CEREBELLUM
BRAIN, CEREBRAL CORTEX
BRAIN, HIPPOCAMPUS
BRAIN, HYPOTHALAMUS
BRAIN, MEDULLA OBLONGATA
BRAIN, MENINGES
BRAIN, MIDBRAIN
BRAIN, OLFACTORY BULB
BRAIN, PONS
BRAIN, THALAMUS
BRAIN, VENTRICULAR SYSTEM
BRAIN, WHITE MATTER
EYE
GANGLION, DORSAL ROOT, CERVICAL
GANGLION, DORSAL ROOT, LUMBAR
GANGLION, DORSAL ROOT, THORACIC
HEART
LUNG
LYMPH NODE, ILIAC
LYMPH NODE, MANDIBULAR
LYMPH NODE, MESENTERIC
MUSCLE, BICEPS FEMORIS
MUSCLE, GASTROCNEMIUS
NERVE ROOT, SPINAL, LUMBAR
NERVE ROOT, SPINAL, THORACIC
NERVE, OPTIC
NERVE, SCIATIC
NERVE, TIBIAL
PANCREAS
SPINAL CORD, CERVICAL
SPINAL CORD, LUMBAR
SPINAL CORD, THORACIC

Appendix 10

Individual Animal Report

Study ID: 02776-0018: CRL 2954-001 / U of Tex Southwestern Med UTSW.Gray-002
Study Title: A SINGLE DOSE TOXICITY STUDY OF AAV9/SURF1 ADMINISTERED BY INTRATHECAL INJECTION IN RATS

Animal: 4012 (Male)

Sacrifice: DAY 08
Group: 4MD08 / AAV9/SURF1 HIGH DOSE (2.49E12 VG)
Fate: SCHEDULED SACRIFICE / Time on Test: 08 / Cause(s) of Death:

Gross or Microscopic Finding
Disposition
Tissue: Site / Finding, Severity

SPLEEN
TESTIS

Disposition: **ABNORMAL/FINDING**

KIDNEY
KIDNEY: CHRONIC PROGRESSIVE NEPHROPATHY, MINIMAL
LIVER
LIVER: EXTRAMEDULLARY HEMATOPOIESIS, MINIMAL
LIVER: INFILTRATE, MIXED, MINIMAL
LIVER, HEPATOCYTE: NECROSIS, SINGLE CELL, MINIMAL
NERVE ROOT, SPINAL, CERVICAL
NERVE ROOT, SPINAL, CERVICAL, NERVE ROOT, VENTRAL: DEGENERATION, NERVE FIBER, MINIMAL

Disposition: **UNABLE TO OBTAIN SPECIMEN**

THYMUS

Appendix 10

Individual Animal Report

Study ID: 02776-0018: CRL 2954-001 / U of Tex Southwestern Med UTSW.Gray-002
Study Title: A SINGLE DOSE TOXICITY STUDY OF AAV9/SURF1 ADMINISTERED BY INTRATHECAL INJECTION IN RATS

Animal: 4021 (Male)

Sacrifice: DAY 08
Group: 4MD08 / AAV9/SURF1 HIGH DOSE (2.49E12 VG)
Fate: SCHEDULED SACRIFICE / Time on Test: 08 / Cause(s) of Death:

Gross or Microscopic Finding
Disposition
Tissue: Site / Finding, Severity

MICROSCOPIC EXAMINATION

Disposition: **NORMAL**

BRAIN, AMYGDALOID BODY
BRAIN, BASAL NUCLEI/STRIATUM
BRAIN, CEREBELLUM
BRAIN, CEREBRAL CORTEX
BRAIN, HIPPOCAMPUS
BRAIN, HYPOTHALAMUS
BRAIN, MEDULLA OBLONGATA
BRAIN, MENINGES
BRAIN, MIDBRAIN
BRAIN, OLFACTORY BULB
BRAIN, PONS
BRAIN, THALAMUS
BRAIN, VENTRICULAR SYSTEM
BRAIN, WHITE MATTER
EYE
GANGLION, DORSAL ROOT, CERVICAL
GANGLION, DORSAL ROOT, LUMBAR
GANGLION, DORSAL ROOT, THORACIC
HEART
LUNG
LYMPH NODE, ILIAC
LYMPH NODE, MANDIBULAR
LYMPH NODE, MESENTERIC
MUSCLE, BICEPS FEMORIS
MUSCLE, GASTROCNEMIUS
NERVE ROOT, SPINAL, CERVICAL
NERVE ROOT, SPINAL, LUMBAR
NERVE ROOT, SPINAL, THORACIC
NERVE, OPTIC
NERVE, SCIATIC
NERVE, TIBIAL
PANCREAS
SPINAL CORD, CERVICAL
SPINAL CORD, LUMBAR

Appendix 10

Individual Animal Report

Study ID: 02776-0018: CRL 2954-001 / U of Tex Southwestern Med UTSW.Gray-002
Study Title: A SINGLE DOSE TOXICITY STUDY OF AAV9/SURF1 ADMINISTERED BY INTRATHECAL INJECTION IN RATS

Animal: 4021 (Male)

Sacrifice: DAY 08
Group: 4MD08 / AAV9/SURF1 HIGH DOSE (2.49E12 VG)
Fate: SCHEDULED SACRIFICE / Time on Test: 08 / Cause(s) of Death:

Gross or Microscopic Finding
Disposition
Tissue: Site / Finding, Severity

SPINAL CORD, THORACIC
SPLEEN
TESTIS
THYMUS

Disposition: ABNORMAL/FINDING

KIDNEY
KIDNEY, TUBULAR: BASOPHILIA, MINIMAL
LIVER
LIVER: INFILTRATE, MIXED, MINIMAL

Appendix 10

Individual Animal Report

Study ID: 02776-0018: CRL 2954-001 / U of Tex Southwestern Med UTSW.Gray-002
Study Title: A SINGLE DOSE TOXICITY STUDY OF AAV9/SURF1 ADMINISTERED BY INTRATHECAL INJECTION IN RATS

Animal: 4022 (Male)

Sacrifice: DAY 08
Group: 4MD08 / AAV9/SURF1 HIGH DOSE (2.49E12 VG)
Fate: SCHEDULED SACRIFICE / Time on Test: 08 / Cause(s) of Death:

Gross or Microscopic Finding
Disposition
Tissue: Site / Finding, Severity

MICROSCOPIC EXAMINATION

Disposition: NORMAL

BRAIN, AMYGDALOID BODY
BRAIN, BASAL NUCLEI/STRIATUM
BRAIN, CEREBELLUM
BRAIN, CEREBRAL CORTEX
BRAIN, HIPPOCAMPUS
BRAIN, HYPOTHALAMUS
BRAIN, MEDULLA OBLONGATA
BRAIN, MENINGES
BRAIN, MIDBRAIN
BRAIN, OLFACTORY BULB
BRAIN, PONS
BRAIN, THALAMUS
BRAIN, VENTRICULAR SYSTEM
BRAIN, WHITE MATTER
EYE
GANGLION, DORSAL ROOT, CERVICAL
GANGLION, DORSAL ROOT, LUMBAR
GANGLION, DORSAL ROOT, THORACIC
HEART
LUNG
LYMPH NODE, ILIAC
LYMPH NODE, MANDIBULAR
LYMPH NODE, MESENTERIC
MUSCLE, BICEPS FEMORIS
MUSCLE, GASTROCNEMIUS
NERVE ROOT, SPINAL, CERVICAL
NERVE ROOT, SPINAL, LUMBAR
NERVE ROOT, SPINAL, THORACIC
NERVE, OPTIC
NERVE, TIBIAL
PANCREAS
SPINAL CORD, CERVICAL
SPINAL CORD, LUMBAR
SPINAL CORD, THORACIC

Appendix 10

Individual Animal Report

Study ID: 02776-0018: CRL 2954-001 / U of Tex Southwestern Med UTSW.Gray-002
Study Title: A SINGLE DOSE TOXICITY STUDY OF AAV9/SURF1 ADMINISTERED BY INTRATHECAL INJECTION IN RATS

Animal: 4022 (Male)

Sacrifice: DAY 08
Group: 4MD08 / AAV9/SURF1 HIGH DOSE (2.49E12 VG)
Fate: SCHEDULED SACRIFICE / Time on Test: 08 / Cause(s) of Death:

Gross or Microscopic Finding
Disposition
Tissue: Site / Finding, Severity

SPLEEN
TESTIS
THYMUS

Disposition: **ABNORMAL/FINDING**

KIDNEY
KIDNEY: CHRONIC PROGRESSIVE NEPHROPATHY, MINIMAL
LIVER
LIVER: INFILTRATE, MIXED, MINIMAL
NERVE, SCIATIC
NERVE, SCIATIC: DEGENERATION, NERVE FIBER, MINIMAL

Appendix 10

Individual Animal Report

Study ID: 02776-0018: CRL 2954-001 / U of Tex Southwestern Med UTSW.Gray-002
Study Title: A SINGLE DOSE TOXICITY STUDY OF AAV9/SURF1 ADMINISTERED BY INTRATHECAL INJECTION IN RATS

Animal: 4113 (Male)

Sacrifice: DAY 08
Group: 4MD08 / AAV9/SURF1 HIGH DOSE (2.49E12 VG)
Fate: SCHEDULED SACRIFICE / Time on Test: 08 / Cause(s) of Death:

Gross or Microscopic Finding
Disposition
Tissue: Site / Finding, Severity

MICROSCOPIC EXAMINATION

Disposition: NORMAL

BRAIN, AMYGDALOID BODY
BRAIN, BASAL NUCLEI/STRIATUM
BRAIN, CEREBELLUM
BRAIN, CEREBRAL CORTEX
BRAIN, HIPPOCAMPUS
BRAIN, HYPOTHALAMUS
BRAIN, MEDULLA OBLONGATA
BRAIN, MENINGES
BRAIN, MIDBRAIN
BRAIN, OLFACTORY BULB
BRAIN, PONS
BRAIN, THALAMUS
BRAIN, VENTRICULAR SYSTEM
BRAIN, WHITE MATTER
EYE
GANGLION, DORSAL ROOT, CERVICAL
GANGLION, DORSAL ROOT, LUMBAR
GANGLION, DORSAL ROOT, THORACIC
KIDNEY
LUNG
LYMPH NODE, ILIAC
LYMPH NODE, MANDIBULAR
LYMPH NODE, MESENTERIC
MUSCLE, BICEPS FEMORIS
MUSCLE, GASTROCNEMIUS
NERVE ROOT, SPINAL, CERVICAL
NERVE ROOT, SPINAL, LUMBAR
NERVE ROOT, SPINAL, THORACIC
NERVE, OPTIC
NERVE, SCIATIC
NERVE, TIBIAL
PANCREAS
SPINAL CORD, CERVICAL
SPINAL CORD, THORACIC

Appendix 10

Individual Animal Report

Study ID: 02776-0018: CRL 2954-001 / U of Tex Southwestern Med UTSW.Gray-002
Study Title: A SINGLE DOSE TOXICITY STUDY OF AAV9/SURF1 ADMINISTERED BY INTRATHECAL INJECTION IN RATS

Animal: 4113 (Male)

Sacrifice: DAY 08
Group: 4MD08 / AAV9/SURF1 HIGH DOSE (2.49E12 VG)
Fate: SCHEDULED SACRIFICE / Time on Test: 08 / Cause(s) of Death:

Gross or Microscopic Finding
Disposition
Tissue: Site / Finding, Severity

SPLEEN
TESTIS
THYMUS

Disposition: **ABNORMAL/FINDING**

HEART
HEART, MYOCARDIUM: INFILTRATE, MONONUCLEAR CELLS, MINIMAL, MULTIFOCAL
LIVER
LIVER: EXTRAMEDULLARY HEMATOPOIESIS, MINIMAL
LIVER: INFILTRATE, MIXED, MINIMAL
SPINAL CORD, LUMBAR
SPINAL CORD, LUMBAR, NERVE ROOT, SPINAL: DEGENERATION, NERVE FIBER, MINIMAL

Appendix 10

Individual Animal Report

Study ID: 02776-0018: CRL 2954-001 / U of Tex Southwestern Med UTSW.Gray-002
Study Title: A SINGLE DOSE TOXICITY STUDY OF AAV9/SURF1 ADMINISTERED BY INTRATHECAL INJECTION IN RATS

Animal: 1511 (Female)

Sacrifice: DAY 08
Group: 1FD08 / VEHICLE (0 VG)
Fate: SCHEDULED SACRIFICE / Time on Test: 08 / Cause(s) of Death:

Gross or Microscopic Finding
Disposition
Tissue: Site / Finding, Severity

MICROSCOPIC EXAMINATION

Disposition: NORMAL

BRAIN, AMYGDALOID BODY
BRAIN, BASAL NUCLEI/STRIATUM
BRAIN, CEREBELLUM
BRAIN, CEREBRAL CORTEX
BRAIN, HIPPOCAMPUS
BRAIN, HYPOTHALAMUS
BRAIN, MEDULLA OBLONGATA
BRAIN, MENINGES
BRAIN, MIDBRAIN
BRAIN, OLFACTORY BULB
BRAIN, PONS
BRAIN, THALAMUS
BRAIN, VENTRICULAR SYSTEM
BRAIN, WHITE MATTER
GANGLION, DORSAL ROOT, CERVICAL
GANGLION, DORSAL ROOT, LUMBAR
GANGLION, DORSAL ROOT, THORACIC
KIDNEY
LUNG
LYMPH NODE, ILIAC
LYMPH NODE, MANDIBULAR
LYMPH NODE, MESENTERIC
MUSCLE, BICEPS FEMORIS
MUSCLE, GASTROCNEMIUS
NERVE ROOT, SPINAL, CERVICAL
NERVE ROOT, SPINAL, LUMBAR
NERVE ROOT, SPINAL, THORACIC
NERVE, OPTIC
NERVE, SCIATIC
NERVE, TIBIAL
OVARY
PANCREAS
SPINAL CORD, CERVICAL
SPINAL CORD, LUMBAR

Appendix 10

Individual Animal Report

Study ID: 02776-0018: CRL 2954-001 / U of Tex Southwestern Med UTSW.Gray-002
Study Title: A SINGLE DOSE TOXICITY STUDY OF AAV9/SURF1 ADMINISTERED BY INTRATHECAL INJECTION IN RATS

Animal: 1511 (Female)

Sacrifice: DAY 08
Group: 1FD08 / VEHICLE (0 VG)
Fate: SCHEDULED SACRIFICE / Time on Test: 08 / Cause(s) of Death:

Gross or Microscopic Finding
Disposition
Tissue: Site / Finding, Severity

SPINAL CORD, THORACIC
SPLEEN
THYMUS

Disposition: **ABNORMAL/FINDING**

EYE
EYE: RETINAL ROSETTE, MINIMAL
HEART
HEART, MYOCARDIUM: DEGENERATION/NECROSIS, MINIMAL, FOCAL
HEART, MYOCARDIUM: INFILTRATE, MONONUCLEAR CELLS, MINIMAL, FOCAL
LIVER
LIVER: EXTRAMEDULLARY HEMATOPOIESIS, MINIMAL
LIVER: INFILTRATE, MIXED, MINIMAL

Appendix 10

Individual Animal Report

Study ID: 02776-0018: CRL 2954-001 / U of Tex Southwestern Med UTSW.Gray-002
Study Title: A SINGLE DOSE TOXICITY STUDY OF AAV9/SURF1 ADMINISTERED BY INTRATHECAL INJECTION IN RATS

Animal: 1512 (Female)

Sacrifice: DAY 08
Group: 1FD08 / VEHICLE (0 VG)
Fate: SCHEDULED SACRIFICE / Time on Test: 08 / Cause(s) of Death:

Gross or Microscopic Finding
Disposition
Tissue: Site / Finding, Severity

MICROSCOPIC EXAMINATION

Disposition: NORMAL

BRAIN, AMYGDALOID BODY
BRAIN, BASAL NUCLEI/STRIATUM
BRAIN, CEREBELLUM
BRAIN, CEREBRAL CORTEX
BRAIN, HIPPOCAMPUS
BRAIN, HYPOTHALAMUS
BRAIN, MEDULLA OBLONGATA
BRAIN, MENINGES
BRAIN, MIDBRAIN
BRAIN, OLFACTORY BULB
BRAIN, PONS
BRAIN, THALAMUS
BRAIN, VENTRICULAR SYSTEM
BRAIN, WHITE MATTER
EYE
GANGLION, DORSAL ROOT, CERVICAL
GANGLION, DORSAL ROOT, THORACIC
LUNG
LYMPH NODE, ILIAC
LYMPH NODE, MANDIBULAR
LYMPH NODE, MESENTERIC
MUSCLE, BICEPS FEMORIS
MUSCLE, GASTROCNEMIUS
NERVE ROOT, SPINAL, CERVICAL
NERVE ROOT, SPINAL, LUMBAR
NERVE ROOT, SPINAL, THORACIC
NERVE, OPTIC
NERVE, SCIATIC
NERVE, TIBIAL
OVARY
PANCREAS
SPINAL CORD, CERVICAL
SPINAL CORD, LUMBAR
SPINAL CORD, THORACIC

Appendix 10

Individual Animal Report

Study ID: 02776-0018: CRL 2954-001 / U of Tex Southwestern Med UTSW.Gray-002
Study Title: A SINGLE DOSE TOXICITY STUDY OF AAV9/SURF1 ADMINISTERED BY INTRATHECAL INJECTION IN RATS

Animal: 1512 (Female)

Sacrifice: DAY 08
Group: 1FD08 / VEHICLE (0 VG)
Fate: SCHEDULED SACRIFICE / Time on Test: 08 / Cause(s) of Death:

Gross or Microscopic Finding
Disposition
Tissue: Site / Finding, Severity

SPLEEN
THYMUS

Disposition: **ABNORMAL/FINDING**

GANGLION, DORSAL ROOT, LUMBAR
GANGLION, DORSAL ROOT, LUMBAR, GLIAL CELL: HYPERTROPHY/HYPERPLASIA, MINIMAL
HEART
HEART, MYOCARDIUM: DEGENERATION/NECROSIS, MINIMAL, FOCAL
HEART, MYOCARDIUM: INFILTRATE, MONONUCLEAR CELLS, MINIMAL, FOCAL
KIDNEY
KIDNEY: CHRONIC PROGRESSIVE NEPHROPATHY, MINIMAL
LIVER
LIVER: EXTRAMEDULLARY HEMATOPOIESIS, MINIMAL

Appendix 10

Individual Animal Report

Study ID: 02776-0018: CRL 2954-001 / U of Tex Southwestern Med UTSW.Gray-002
Study Title: A SINGLE DOSE TOXICITY STUDY OF AAV9/SURF1 ADMINISTERED BY INTRATHECAL INJECTION IN RATS

Animal: 1513 (Female)

Sacrifice: DAY 08
Group: 1FD08 / VEHICLE (0 VG)
Fate: SCHEDULED SACRIFICE / Time on Test: 08 / Cause(s) of Death:

Gross or Microscopic Finding
Disposition
Tissue: Site / Finding, Severity

MICROSCOPIC EXAMINATION

Disposition: NORMAL

BRAIN, AMYGDALOID BODY
BRAIN, BASAL NUCLEI/STRIATUM
BRAIN, CEREBELLUM
BRAIN, CEREBRAL CORTEX
BRAIN, HIPPOCAMPUS
BRAIN, HYPOTHALAMUS
BRAIN, MEDULLA OBLONGATA
BRAIN, MENINGES
BRAIN, MIDBRAIN
BRAIN, OLFACTORY BULB
BRAIN, PONS
BRAIN, THALAMUS
BRAIN, VENTRICULAR SYSTEM
BRAIN, WHITE MATTER
EYE
GANGLION, DORSAL ROOT, CERVICAL
GANGLION, DORSAL ROOT, LUMBAR
GANGLION, DORSAL ROOT, THORACIC
HEART
LUNG
LYMPH NODE, ILIAC
LYMPH NODE, MANDIBULAR
LYMPH NODE, MESENTERIC
MUSCLE, BICEPS FEMORIS
MUSCLE, GASTROCNEMIUS
NERVE ROOT, SPINAL, CERVICAL
NERVE ROOT, SPINAL, LUMBAR
NERVE ROOT, SPINAL, THORACIC
NERVE, OPTIC
NERVE, SCIATIC
NERVE, TIBIAL
OVARY
PANCREAS
SPINAL CORD, CERVICAL

Appendix 10

Individual Animal Report

Study ID: 02776-0018: CRL 2954-001 / U of Tex Southwestern Med UTSW.Gray-002
Study Title: A SINGLE DOSE TOXICITY STUDY OF AAV9/SURF1 ADMINISTERED BY INTRATHECAL INJECTION IN RATS

Animal: 1513 (Female)

Sacrifice: DAY 08
Group: 1FD08 / VEHICLE (0 VG)
Fate: SCHEDULED SACRIFICE / Time on Test: 08 / Cause(s) of Death:

Gross or Microscopic Finding
Disposition
Tissue: Site / Finding, Severity

SPINAL CORD, LUMBAR
SPINAL CORD, THORACIC
SPLEEN
THYMUS

Disposition: ABNORMAL/FINDING

KIDNEY
KIDNEY, TUBULAR: BASOPHILIA, MINIMAL
LIVER
LIVER: EXTRAMEDULLARY HEMATOPOIESIS, MINIMAL
LIVER: INFILTRATE, MIXED, MINIMAL

Appendix 10

Individual Animal Report

Study ID: 02776-0018: CRL 2954-001 / U of Tex Southwestern Med UTSW.Gray-002
Study Title: A SINGLE DOSE TOXICITY STUDY OF AAV9/SURF1 ADMINISTERED BY INTRATHECAL INJECTION IN RATS

Animal: 1514 (Female)

Sacrifice: DAY 08
Group: 1FD08 / VEHICLE (0 VG)
Fate: SCHEDULED SACRIFICE / Time on Test: 08 / Cause(s) of Death:

Gross or Microscopic Finding
Disposition
Tissue: Site / Finding, Severity

MICROSCOPIC EXAMINATION

Disposition: NORMAL

BRAIN, AMYGDALOID BODY
BRAIN, BASAL NUCLEI/STRIATUM
BRAIN, CEREBELLUM
BRAIN, CEREBRAL CORTEX
BRAIN, HIPPOCAMPUS
BRAIN, HYPOTHALAMUS
BRAIN, MEDULLA OBLONGATA
BRAIN, MENINGES
BRAIN, MIDBRAIN
BRAIN, OLFACTORY BULB
BRAIN, PONS
BRAIN, THALAMUS
BRAIN, VENTRICULAR SYSTEM
BRAIN, WHITE MATTER
EYE
GANGLION, DORSAL ROOT, CERVICAL
GANGLION, DORSAL ROOT, LUMBAR
GANGLION, DORSAL ROOT, THORACIC
HEART
KIDNEY
LIVER
LYMPH NODE, ILIAC
LYMPH NODE, MANDIBULAR
LYMPH NODE, MESENTERIC
MUSCLE, BICEPS FEMORIS
MUSCLE, GASTROCNEMIUS
NERVE ROOT, SPINAL, CERVICAL
NERVE ROOT, SPINAL, LUMBAR
NERVE ROOT, SPINAL, THORACIC
NERVE, OPTIC
NERVE, TIBIAL
OVARY
PANCREAS
SPINAL CORD, CERVICAL

Appendix 10

Individual Animal Report

Study ID: 02776-0018: CRL 2954-001 / U of Tex Southwestern Med UTSW.Gray-002
Study Title: A SINGLE DOSE TOXICITY STUDY OF AAV9/SURF1 ADMINISTERED BY INTRATHECAL INJECTION IN RATS

Animal: 1514 (Female)

Sacrifice: DAY 08
Group: 1FD08 / VEHICLE (0 VG)
Fate: SCHEDULED SACRIFICE / Time on Test: 08 / Cause(s) of Death:

Gross or Microscopic Finding
Disposition
Tissue: Site / Finding, Severity

SPINAL CORD, LUMBAR
SPINAL CORD, THORACIC
SPLEEN
THYMUS

Disposition: ABNORMAL/FINDING

LUNG
LUNG, ALVEOLUS: INFILTRATE, MACROPHAGES, MINIMAL, FOCAL
NERVE, SCIATIC
NERVE, SCIATIC: DEGENERATION, NERVE FIBER, MINIMAL

Appendix 10

Individual Animal Report

Study ID: 02776-0018: CRL 2954-001 / U of Tex Southwestern Med UTSW.Gray-002
Study Title: A SINGLE DOSE TOXICITY STUDY OF AAV9/SURF1 ADMINISTERED BY INTRATHECAL INJECTION IN RATS

Animal: 1515 (Female)

Sacrifice: DAY 08
Group: 1FD08 / VEHICLE (0 VG)
Fate: SCHEDULED SACRIFICE / Time on Test: 08 / Cause(s) of Death:

Gross or Microscopic Finding
Disposition
Tissue: Site / Finding, Severity

MICROSCOPIC EXAMINATION

Disposition: NORMAL

BRAIN, AMYGDALOID BODY
BRAIN, BASAL NUCLEI/STRIATUM
BRAIN, CEREBELLUM
BRAIN, CEREBRAL CORTEX
BRAIN, HIPPOCAMPUS
BRAIN, HYPOTHALAMUS
BRAIN, MEDULLA OBLONGATA
BRAIN, MENINGES
BRAIN, MIDBRAIN
BRAIN, OLFACTORY BULB
BRAIN, PONS
BRAIN, THALAMUS
BRAIN, VENTRICULAR SYSTEM
BRAIN, WHITE MATTER
GANGLION, DORSAL ROOT, LUMBAR
GANGLION, DORSAL ROOT, THORACIC
HEART
KIDNEY
LIVER
LUNG
LYMPH NODE, ILIAC
LYMPH NODE, MANDIBULAR
LYMPH NODE, MESENTERIC
MUSCLE, BICEPS FEMORIS
MUSCLE, GASTROCNEMIUS
NERVE ROOT, SPINAL, CERVICAL
NERVE ROOT, SPINAL, LUMBAR
NERVE ROOT, SPINAL, THORACIC
NERVE, OPTIC
OVARY
PANCREAS
SPINAL CORD, CERVICAL
SPINAL CORD, THORACIC
SPLEEN

Appendix 10

Individual Animal Report

Study ID: 02776-0018: CRL 2954-001 / U of Tex Southwestern Med UTSW.Gray-002
Study Title: A SINGLE DOSE TOXICITY STUDY OF AAV9/SURF1 ADMINISTERED BY INTRATHECAL INJECTION IN RATS

Animal: 1515 (Female)

Sacrifice: DAY 08
Group: 1FD08 / VEHICLE (0 VG)
Fate: SCHEDULED SACRIFICE / Time on Test: 08 / Cause(s) of Death:

Gross or Microscopic Finding
Disposition
Tissue: Site / Finding, Severity

THYMUS

Disposition: ABNORMAL/FINDING

EYE
EYE: RETINAL ROSETTE, MINIMAL
GANGLION, DORSAL ROOT, CERVICAL
GANGLION, DORSAL ROOT, CERVICAL, GLIAL CELL: HYPERTROPHY/HYPERPLASIA, MINIMAL
NERVE, SCIATIC
NERVE, SCIATIC: DEGENERATION, NERVE FIBER, MINIMAL
NERVE, TIBIAL
NERVE, TIBIAL: DEGENERATION, NERVE FIBER, MINIMAL
COMMENT(S): CROSS SECTION NOT PRESENT ON ORIGINAL OR REWORK SLIDE
SPINAL CORD, LUMBAR
SPINAL CORD, LUMBAR, WHITE MATTER: DEGENERATION, NERVE FIBER, MINIMAL, DORSAL

Appendix 10

Individual Animal Report

Study ID: 02776-0018: CRL 2954-001 / U of Tex Southwestern Med UTSW.Gray-002
Study Title: A SINGLE DOSE TOXICITY STUDY OF AAV9/SURF1 ADMINISTERED BY INTRATHECAL INJECTION IN RATS

Animal: 2511 (Female)

Sacrifice: DAY 08
Group: 2FD08 / AAV9/SURF1 LOW DOSE (0.28E12 VG)
Fate: SCHEDULED SACRIFICE / Time on Test: 08 / Cause(s) of Death:

Gross or Microscopic Finding
Disposition
Tissue: Site / Finding, Severity

MICROSCOPIC EXAMINATION

Disposition: NORMAL

BRAIN, AMYGDALOID BODY
BRAIN, BASAL NUCLEI/STRIATUM
BRAIN, CEREBELLUM
BRAIN, CEREBRAL CORTEX
BRAIN, HIPPOCAMPUS
BRAIN, HYPOTHALAMUS
BRAIN, MENINGES
BRAIN, MIDBRAIN
BRAIN, OLFACTORY BULB
BRAIN, PONS
BRAIN, THALAMUS
BRAIN, VENTRICULAR SYSTEM
BRAIN, WHITE MATTER
GANGLION, DORSAL ROOT, CERVICAL
GANGLION, DORSAL ROOT, THORACIC
HEART
LUNG
LYMPH NODE, ILIAC
LYMPH NODE, MANDIBULAR
LYMPH NODE, MESENTERIC
MUSCLE, BICEPS FEMORIS
MUSCLE, GASTROCNEMIUS
NERVE ROOT, SPINAL, CERVICAL
NERVE ROOT, SPINAL, THORACIC
NERVE, OPTIC
NERVE, SCIATIC
NERVE, TIBIAL
OVARY
PANCREAS
SPINAL CORD, CERVICAL
SPINAL CORD, LUMBAR
SPINAL CORD, THORACIC
SPLEEN
THYMUS

Appendix 10

Individual Animal Report

Study ID: 02776-0018: CRL 2954-001 / U of Tex Southwestern Med UTSW.Gray-002
Study Title: A SINGLE DOSE TOXICITY STUDY OF AAV9/SURF1 ADMINISTERED BY INTRATHECAL INJECTION IN RATS

Animal: 2511 (Female)

Sacrifice: DAY 08
Group: 2FD08 / AAV9/SURF1 LOW DOSE (0.28E12 VG)
Fate: SCHEDULED SACRIFICE / Time on Test: 08 / Cause(s) of Death:

Gross or Microscopic Finding
Disposition
Tissue: Site / Finding, Severity

Disposition: **ABNORMAL/FINDING**

BRAIN, MEDULLA OBLONGATA
BRAIN, MEDULLA OBLONGATA, WHITE MATTER: DEGENERATION, NERVE FIBER, MINIMAL
COMMENT(S): PYRAMIDAL TRACT

EYE
EYE: RETINAL ROSETTE, MINIMAL

GANGLION, DORSAL ROOT, LUMBAR
GANGLION, DORSAL ROOT, LUMBAR, GLIAL CELL: HYPERTROPHY/HYPERPLASIA, MINIMAL

KIDNEY
KIDNEY, TUBULAR: CYST, MINIMAL

LIVER
LIVER: INFILTRATE, MIXED, MINIMAL

NERVE ROOT, SPINAL, LUMBAR
NERVE ROOT, SPINAL, LUMBAR, EPINEURIUM: INFILTRATE, MONONUCLEAR CELLS, MINIMAL, FOCAL

Appendix 10

Individual Animal Report

Study ID: 02776-0018: CRL 2954-001 / U of Tex Southwestern Med UTSW.Gray-002
Study Title: A SINGLE DOSE TOXICITY STUDY OF AAV9/SURF1 ADMINISTERED BY INTRATHECAL INJECTION IN RATS

Animal: 2512 (Female)

Sacrifice: DAY 08
Group: 2FD08 / AAV9/SURF1 LOW DOSE (0.28E12 VG)
Fate: SCHEDULED SACRIFICE / Time on Test: 08 / Cause(s) of Death:

Gross or Microscopic Finding
Disposition
Tissue: Site / Finding, Severity

MICROSCOPIC EXAMINATION

Disposition: NORMAL

BRAIN, AMYGDALOID BODY
BRAIN, BASAL NUCLEI/STRIATUM
BRAIN, CEREBELLUM
BRAIN, CEREBRAL CORTEX
BRAIN, HIPPOCAMPUS
BRAIN, HYPOTHALAMUS
BRAIN, MEDULLA OBLONGATA
BRAIN, MENINGES
BRAIN, MIDBRAIN
BRAIN, OLFACTORY BULB
BRAIN, PONS
BRAIN, THALAMUS
BRAIN, VENTRICULAR SYSTEM
BRAIN, WHITE MATTER
EYE
GANGLION, DORSAL ROOT, CERVICAL
GANGLION, DORSAL ROOT, LUMBAR
GANGLION, DORSAL ROOT, THORACIC
HEART
KIDNEY
LIVER
LUNG
LYMPH NODE, ILIAC
LYMPH NODE, MANDIBULAR
LYMPH NODE, MESENTERIC
MUSCLE, BICEPS FEMORIS
MUSCLE, GASTROCNEMIUS
NERVE ROOT, SPINAL, CERVICAL
NERVE ROOT, SPINAL, THORACIC
NERVE, OPTIC
NERVE, SCIATIC
NERVE, TIBIAL
OVARY
PANCREAS

Appendix 10

Individual Animal Report

Study ID: 02776-0018: CRL 2954-001 / U of Tex Southwestern Med UTSW.Gray-002
Study Title: A SINGLE DOSE TOXICITY STUDY OF AAV9/SURF1 ADMINISTERED BY INTRATHECAL INJECTION IN RATS

Animal: 2512 (Female)

Sacrifice: DAY 08
Group: 2FD08 / AAV9/SURF1 LOW DOSE (0.28E12 VG)
Fate: SCHEDULED SACRIFICE / Time on Test: 08 / Cause(s) of Death:

Gross or Microscopic Finding
Disposition
Tissue: Site / Finding, Severity

SPINAL CORD, CERVICAL
SPINAL CORD, LUMBAR
SPINAL CORD, THORACIC
SPLEEN
THYMUS

Disposition: ABNORMAL/FINDING

NERVE ROOT, SPINAL, LUMBAR
NERVE ROOT, SPINAL, LUMBAR, EPINEURIUM: INFILTRATE, MONONUCLEAR CELLS, MINIMAL, FOCAL

Appendix 10

Individual Animal Report

Study ID: 02776-0018: CRL 2954-001 / U of Tex Southwestern Med UTSW.Gray-002
Study Title: A SINGLE DOSE TOXICITY STUDY OF AAV9/SURF1 ADMINISTERED BY INTRATHECAL INJECTION IN RATS

Animal: 2513 (Female)

Sacrifice: DAY 08
Group: 2FD08 / AAV9/SURF1 LOW DOSE (0.28E12 VG)
Fate: SCHEDULED SACRIFICE / Time on Test: 08 / Cause(s) of Death:

Gross or Microscopic Finding
Disposition
Tissue: Site / Finding, Severity

MICROSCOPIC EXAMINATION

Disposition: NORMAL

BRAIN, AMYGDALOID BODY
BRAIN, BASAL NUCLEI/STRIATUM
BRAIN, CEREBELLUM
BRAIN, CEREBRAL CORTEX
BRAIN, HIPPOCAMPUS
BRAIN, HYPOTHALAMUS
BRAIN, MEDULLA OBLONGATA
BRAIN, MENINGES
BRAIN, MIDBRAIN
BRAIN, OLFACTORY BULB
BRAIN, PONS
BRAIN, THALAMUS
BRAIN, VENTRICULAR SYSTEM
BRAIN, WHITE MATTER
EYE
GANGLION, DORSAL ROOT, CERVICAL
GANGLION, DORSAL ROOT, LUMBAR
GANGLION, DORSAL ROOT, THORACIC
HEART
KIDNEY
LUNG
LYMPH NODE, ILIAC
LYMPH NODE, MANDIBULAR
LYMPH NODE, MESENTERIC
MUSCLE, BICEPS FEMORIS
MUSCLE, GASTROCNEMIUS
NERVE ROOT, SPINAL, CERVICAL
NERVE ROOT, SPINAL, THORACIC
NERVE, OPTIC
NERVE, SCIATIC
NERVE, TIBIAL
OVARY
PANCREAS
SPINAL CORD, CERVICAL

Appendix 10

Individual Animal Report

Study ID: 02776-0018: CRL 2954-001 / U of Tex Southwestern Med UTSW.Gray-002
Study Title: A SINGLE DOSE TOXICITY STUDY OF AAV9/SURF1 ADMINISTERED BY INTRATHECAL INJECTION IN RATS

Animal: 2513 (Female)

Sacrifice: DAY 08
Group: 2FD08 / AAV9/SURF1 LOW DOSE (0.28E12 VG)
Fate: SCHEDULED SACRIFICE / Time on Test: 08 / Cause(s) of Death:

Gross or Microscopic Finding
Disposition
Tissue: Site / Finding, Severity

SPINAL CORD, LUMBAR
SPINAL CORD, THORACIC
SPLEEN
THYMUS

Disposition: **ABNORMAL/FINDING**

LIVER
LIVER: INFILTRATE, MIXED, MINIMAL
NERVE ROOT, SPINAL, LUMBAR
NERVE ROOT, SPINAL, LUMBAR, NERVE ROOT, VENTRAL: DEGENERATION, NERVE FIBER, MILD

Appendix 10

Individual Animal Report

Study ID: 02776-0018: CRL 2954-001 / U of Tex Southwestern Med UTSW.Gray-002
Study Title: A SINGLE DOSE TOXICITY STUDY OF AAV9/SURF1 ADMINISTERED BY INTRATHECAL INJECTION IN RATS

Animal: 2514 (Female)

Sacrifice: DAY 08
Group: 2FD08 / AAV9/SURF1 LOW DOSE (0.28E12 VG)
Fate: SCHEDULED SACRIFICE / Time on Test: 08 / Cause(s) of Death:

Gross or Microscopic Finding
Disposition
Tissue: Site / Finding, Severity

MICROSCOPIC EXAMINATION

Disposition: NORMAL

BRAIN, AMYGDALOID BODY
BRAIN, BASAL NUCLEI/STRIATUM
BRAIN, CEREBELLUM
BRAIN, CEREBRAL CORTEX
BRAIN, HIPPOCAMPUS
BRAIN, HYPOTHALAMUS
BRAIN, MEDULLA OBLONGATA
BRAIN, MENINGES
BRAIN, MIDBRAIN
BRAIN, OLFACTORY BULB
BRAIN, PONS
BRAIN, THALAMUS
BRAIN, VENTRICULAR SYSTEM
BRAIN, WHITE MATTER
EYE
GANGLION, DORSAL ROOT, CERVICAL
GANGLION, DORSAL ROOT, LUMBAR
GANGLION, DORSAL ROOT, THORACIC
HEART
KIDNEY
LYMPH NODE, ILIAC
LYMPH NODE, MANDIBULAR
LYMPH NODE, MESENTERIC
MUSCLE, BICEPS FEMORIS
MUSCLE, GASTROCNEMIUS
NERVE ROOT, SPINAL, CERVICAL
NERVE ROOT, SPINAL, THORACIC
NERVE, OPTIC
OVARY
PANCREAS
SPINAL CORD, CERVICAL
SPINAL CORD, LUMBAR
SPINAL CORD, THORACIC
SPLEEN

Appendix 10

Individual Animal Report

Study ID: 02776-0018: CRL 2954-001 / U of Tex Southwestern Med UTSW.Gray-002
Study Title: A SINGLE DOSE TOXICITY STUDY OF AAV9/SURF1 ADMINISTERED BY INTRATHECAL INJECTION IN RATS

Animal: 2514 (Female)

Sacrifice: DAY 08
Group: 2FD08 / AAV9/SURF1 LOW DOSE (0.28E12 VG)
Fate: SCHEDULED SACRIFICE / Time on Test: 08 / Cause(s) of Death:

Gross or Microscopic Finding
Disposition
Tissue: Site / Finding, Severity

THYMUS

Disposition: **ABNORMAL/FINDING**

LIVER
LIVER: INFILTRATE, MIXED, MINIMAL
LUNG
LUNG: INFLAMMATION, MINIMAL, SUBACUTE, FOCAL
NERVE ROOT, SPINAL, LUMBAR
NERVE ROOT, SPINAL, LUMBAR, NERVE ROOT, VENTRAL: DEGENERATION, NERVE FIBER, MILD
NERVE, SCIATIC
NERVE, SCIATIC: DEGENERATION, NERVE FIBER, MINIMAL
NERVE, TIBIAL
NERVE, TIBIAL: DEGENERATION, NERVE FIBER, MINIMAL

Appendix 10

Individual Animal Report

Study ID: 02776-0018: CRL 2954-001 / U of Tex Southwestern Med UTSW.Gray-002
Study Title: A SINGLE DOSE TOXICITY STUDY OF AAV9/SURF1 ADMINISTERED BY INTRATHECAL INJECTION IN RATS

Animal: 2515 (Female)

Sacrifice: DAY 08
Group: 2FD08 / AAV9/SURF1 LOW DOSE (0.28E12 VG)
Fate: SCHEDULED SACRIFICE / Time on Test: 08 / Cause(s) of Death:

Gross or Microscopic Finding
Disposition
Tissue: Site / Finding, Severity

MICROSCOPIC EXAMINATION

Disposition: NORMAL

BRAIN, AMYGDALOID BODY
BRAIN, BASAL NUCLEI/STRIATUM
BRAIN, CEREBELLUM
BRAIN, CEREBRAL CORTEX
BRAIN, HIPPOCAMPUS
BRAIN, HYPOTHALAMUS
BRAIN, MEDULLA OBLONGATA
BRAIN, MENINGES
BRAIN, MIDBRAIN
BRAIN, OLFACTORY BULB
BRAIN, PONS
BRAIN, THALAMUS
BRAIN, VENTRICULAR SYSTEM
BRAIN, WHITE MATTER
EYE
GANGLION, DORSAL ROOT, CERVICAL
GANGLION, DORSAL ROOT, LUMBAR
GANGLION, DORSAL ROOT, THORACIC
HEART
KIDNEY
LYMPH NODE, ILIAC
LYMPH NODE, MANDIBULAR
LYMPH NODE, MESENTERIC
MUSCLE, BICEPS FEMORIS
MUSCLE, GASTROCNEMIUS
NERVE ROOT, SPINAL, CERVICAL
NERVE ROOT, SPINAL, LUMBAR
NERVE ROOT, SPINAL, THORACIC
NERVE, OPTIC
OVARY
PANCREAS
SPINAL CORD, CERVICAL
SPINAL CORD, LUMBAR
SPINAL CORD, THORACIC

Appendix 10

Individual Animal Report

Study ID: 02776-0018: CRL 2954-001 / U of Tex Southwestern Med UTSW.Gray-002
Study Title: A SINGLE DOSE TOXICITY STUDY OF AAV9/SURF1 ADMINISTERED BY INTRATHECAL INJECTION IN RATS

Animal: 2515 (Female)

Sacrifice: DAY 08
Group: 2FD08 / AAV9/SURF1 LOW DOSE (0.28E12 VG)
Fate: SCHEDULED SACRIFICE / Time on Test: 08 / Cause(s) of Death:

Gross or Microscopic Finding
Disposition
Tissue: Site / Finding, Severity

SPLEEN
THYMUS

Disposition: **ABNORMAL/FINDING**

LIVER
LIVER: INFILTRATE, MIXED, MINIMAL
LUNG
LUNG, ALVEOLUS: INFILTRATE, MACROPHAGES, MINIMAL, FOCAL
NERVE, SCIATIC
NERVE, SCIATIC: DEGENERATION, NERVE FIBER, MINIMAL
NERVE, TIBIAL
NERVE, TIBIAL: DEGENERATION, NERVE FIBER, MINIMAL

Appendix 10

Individual Animal Report

Study ID: 02776-0018: CRL 2954-001 / U of Tex Southwestern Med UTSW.Gray-002
Study Title: A SINGLE DOSE TOXICITY STUDY OF AAV9/SURF1 ADMINISTERED BY INTRATHECAL INJECTION IN RATS

Animal: 3511 (Female)

Sacrifice: DAY 08
Group: 3FD08 / AAV9/SURF1 MID DOSE (0.83E12 VG)
Fate: SCHEDULED SACRIFICE / Time on Test: 08 / Cause(s) of Death:

Gross or Microscopic Finding
Disposition
Tissue: Site / Finding, Severity

MICROSCOPIC EXAMINATION

Disposition: NORMAL

BRAIN, AMYGDALOID BODY
BRAIN, BASAL NUCLEI/STRIATUM
BRAIN, CEREBELLUM
BRAIN, CEREBRAL CORTEX
BRAIN, HIPPOCAMPUS
BRAIN, HYPOTHALAMUS
BRAIN, MEDULLA OBLONGATA
BRAIN, MENINGES
BRAIN, MIDBRAIN
BRAIN, OLFACTORY BULB
BRAIN, PONS
BRAIN, THALAMUS
BRAIN, VENTRICULAR SYSTEM
BRAIN, WHITE MATTER
EYE
GANGLION, DORSAL ROOT, CERVICAL
GANGLION, DORSAL ROOT, LUMBAR
GANGLION, DORSAL ROOT, THORACIC
LUNG
LYMPH NODE, ILIAC
LYMPH NODE, MANDIBULAR
LYMPH NODE, MESENTERIC
MUSCLE, BICEPS FEMORIS
MUSCLE, GASTROCNEMIUS
NERVE ROOT, SPINAL, CERVICAL
NERVE ROOT, SPINAL, THORACIC
NERVE, OPTIC
NERVE, SCIATIC
NERVE, TIBIAL
OVARY
PANCREAS
SPINAL CORD, CERVICAL
SPINAL CORD, LUMBAR
SPINAL CORD, THORACIC

Appendix 10

Individual Animal Report

Study ID: 02776-0018: CRL 2954-001 / U of Tex Southwestern Med UTSW.Gray-002
Study Title: A SINGLE DOSE TOXICITY STUDY OF AAV9/SURF1 ADMINISTERED BY INTRATHECAL INJECTION IN RATS

Animal: 3511 (Female)

Sacrifice: DAY 08
Group: 3FD08 / AAV9/SURF1 MID DOSE (0.83E12 VG)
Fate: SCHEDULED SACRIFICE / Time on Test: 08 / Cause(s) of Death:

Gross or Microscopic Finding
Disposition
Tissue: Site / Finding, Severity

SPLEEN
THYMUS

Disposition: **ABNORMAL/FINDING**

HEART
HEART, MYOCARDIUM: DEGENERATION/NECROSIS, MINIMAL, MULTIFOCAL
HEART, MYOCARDIUM: INFILTRATE, MONONUCLEAR CELLS, MINIMAL, MULTIFOCAL
KIDNEY
KIDNEY: CHRONIC PROGRESSIVE NEPHROPATHY, MINIMAL
LIVER
LIVER: INFILTRATE, MIXED, MINIMAL
NERVE ROOT, SPINAL, LUMBAR
NERVE ROOT, SPINAL, LUMBAR, EPINEURIUM: INFILTRATE, MONONUCLEAR CELLS, MINIMAL, FOCAL

Appendix 10

Individual Animal Report

Study ID: 02776-0018: CRL 2954-001 / U of Tex Southwestern Med UTSW.Gray-002
Study Title: A SINGLE DOSE TOXICITY STUDY OF AAV9/SURF1 ADMINISTERED BY INTRATHECAL INJECTION IN RATS

Animal: 3512 (Female)

Sacrifice: DAY 08
Group: 3FD08 / AAV9/SURF1 MID DOSE (0.83E12 VG)
Fate: SCHEDULED SACRIFICE / Time on Test: 08 / Cause(s) of Death:

Gross or Microscopic Finding
Disposition
Tissue: Site / Finding, Severity

MICROSCOPIC EXAMINATION

Disposition: NORMAL

BRAIN, AMYGDALOID BODY
BRAIN, BASAL NUCLEI/STRIATUM
BRAIN, CEREBELLUM
BRAIN, CEREBRAL CORTEX
BRAIN, HIPPOCAMPUS
BRAIN, HYPOTHALAMUS
BRAIN, MEDULLA OBLONGATA
BRAIN, MENINGES
BRAIN, MIDBRAIN
BRAIN, OLFACTORY BULB
BRAIN, PONS
BRAIN, THALAMUS
BRAIN, VENTRICULAR SYSTEM
BRAIN, WHITE MATTER
EYE
GANGLION, DORSAL ROOT, CERVICAL
GANGLION, DORSAL ROOT, LUMBAR
GANGLION, DORSAL ROOT, THORACIC
HEART
KIDNEY
LUNG
LYMPH NODE, ILIAC
LYMPH NODE, MANDIBULAR
LYMPH NODE, MESENTERIC
MUSCLE, BICEPS FEMORIS
MUSCLE, GASTROCNEMIUS
NERVE ROOT, SPINAL, CERVICAL
NERVE ROOT, SPINAL, LUMBAR
NERVE ROOT, SPINAL, THORACIC
NERVE, OPTIC
NERVE, SCIATIC
NERVE, TIBIAL
OVARY
PANCREAS

Appendix 10

Individual Animal Report

Study ID: 02776-0018: CRL 2954-001 / U of Tex Southwestern Med UTSW.Gray-002
Study Title: A SINGLE DOSE TOXICITY STUDY OF AAV9/SURF1 ADMINISTERED BY INTRATHECAL INJECTION IN RATS

Animal: 3512 (Female)

Sacrifice: DAY 08
Group: 3FD08 / AAV9/SURF1 MID DOSE (0.83E12 VG)
Fate: SCHEDULED SACRIFICE / Time on Test: 08 / Cause(s) of Death:

Gross or Microscopic Finding
Disposition
Tissue: Site / Finding, Severity

SPINAL CORD, CERVICAL
SPINAL CORD, LUMBAR
SPLEEN
THYMUS

Disposition: **ABNORMAL/FINDING**

LIVER
LIVER: EXTRAMEDULLARY HEMATOPOIESIS, MINIMAL
LIVER: INFILTRATE, MIXED, MINIMAL
SPINAL CORD, THORACIC
SPINAL CORD, THORACIC, GLIAL CELL: INCREASED CELLULARITY, MILD
COMMENT(S): PREDOMINANTLY AFFECTING GRAY MATTER REGIONS JUST ADJACENT TO THE CENTRAL CANAL
SPINAL CORD, THORACIC, GRAY MATTER: DEGENERATION/NECROSIS, MILD
COMMENT(S): AFFECTED CELLS ARE ADJACENT TO THE CENTRAL CANAL
SPINAL CORD, THORACIC, GRAY MATTER: INFILTRATE, MONONUCLEAR CELLS, MINIMAL, MULTIFOCAL, PERIVASCULAR
COMMENT(S): ADJACENT TO CENTRAL CANAL
SPINAL CORD, THORACIC, WHITE MATTER: DEGENERATION, NERVE FIBER, MINIMAL
COMMENT(S): MINIMAL CHANGES SEEN IN BOTH LATERAL AND VENTRAL WHITE MATTER TRACTS

Appendix 10

Individual Animal Report

Study ID: 02776-0018: CRL 2954-001 / U of Tex Southwestern Med UTSW.Gray-002
Study Title: A SINGLE DOSE TOXICITY STUDY OF AAV9/SURF1 ADMINISTERED BY INTRATHECAL INJECTION IN RATS

Animal: 3513 (Female)

Sacrifice: DAY 08
Group: 3FD08 / AAV9/SURF1 MID DOSE (0.83E12 VG)
Fate: SCHEDULED SACRIFICE / Time on Test: 08 / Cause(s) of Death:

Gross or Microscopic Finding
Disposition
Tissue: Site / Finding, Severity

MICROSCOPIC EXAMINATION

Disposition: NORMAL

BRAIN, AMYGDALOID BODY
BRAIN, BASAL NUCLEI/STRIATUM
BRAIN, CEREBELLUM
BRAIN, CEREBRAL CORTEX
BRAIN, HIPPOCAMPUS
BRAIN, HYPOTHALAMUS
BRAIN, MEDULLA OBLONGATA
BRAIN, MENINGES
BRAIN, MIDBRAIN
BRAIN, OLFACTORY BULB
BRAIN, PONS
BRAIN, THALAMUS
BRAIN, VENTRICULAR SYSTEM
BRAIN, WHITE MATTER
EYE
GANGLION, DORSAL ROOT, CERVICAL
GANGLION, DORSAL ROOT, THORACIC
HEART
KIDNEY
LUNG
LYMPH NODE, ILIAC
LYMPH NODE, MANDIBULAR
LYMPH NODE, MESENTERIC
MUSCLE, BICEPS FEMORIS
MUSCLE, GASTROCNEMIUS
NERVE ROOT, SPINAL, CERVICAL
NERVE ROOT, SPINAL, THORACIC
NERVE, OPTIC
NERVE, SCIATIC
NERVE, TIBIAL
OVARY
PANCREAS
SPINAL CORD, CERVICAL
SPINAL CORD, LUMBAR

Appendix 10

Individual Animal Report

Study ID: 02776-0018: CRL 2954-001 / U of Tex Southwestern Med UTSW.Gray-002
Study Title: A SINGLE DOSE TOXICITY STUDY OF AAV9/SURF1 ADMINISTERED BY INTRATHECAL INJECTION IN RATS

Animal: 3513 (Female)

Sacrifice: DAY 08
Group: 3FD08 / AAV9/SURF1 MID DOSE (0.83E12 VG)
Fate: SCHEDULED SACRIFICE / Time on Test: 08 / Cause(s) of Death:

Gross or Microscopic Finding
Disposition
Tissue: Site / Finding, Severity

SPINAL CORD, THORACIC
SPLEEN
THYMUS

Disposition: **ABNORMAL/FINDING**

GANGLION, DORSAL ROOT, LUMBAR
GANGLION, DORSAL ROOT, LUMBAR: INFILTRATE, MONONUCLEAR CELLS, MINIMAL
LIVER
LIVER: INFILTRATE, MIXED, MINIMAL
NERVE ROOT, SPINAL, LUMBAR
NERVE ROOT, SPINAL, LUMBAR, EPINEURIUM: INFILTRATE, MONONUCLEAR CELLS, MINIMAL, FOCAL

Appendix 10

Individual Animal Report

Study ID: 02776-0018: CRL 2954-001 / U of Tex Southwestern Med UTSW.Gray-002
Study Title: A SINGLE DOSE TOXICITY STUDY OF AAV9/SURF1 ADMINISTERED BY INTRATHECAL INJECTION IN RATS

Animal: 3514 (Female)

Sacrifice: DAY 08
Group: 3FD08 / AAV9/SURF1 MID DOSE (0.83E12 VG)
Fate: SCHEDULED SACRIFICE / Time on Test: 08 / Cause(s) of Death:

Gross or Microscopic Finding
Disposition
Tissue: Site / Finding, Severity

MICROSCOPIC EXAMINATION

Disposition: **NORMAL**

BRAIN, AMYGDALOID BODY
BRAIN, BASAL NUCLEI/STRIATUM
BRAIN, CEREBELLUM
BRAIN, CEREBRAL CORTEX
BRAIN, HIPPOCAMPUS
BRAIN, HYPOTHALAMUS
BRAIN, MEDULLA OBLONGATA
BRAIN, MENINGES
BRAIN, MIDBRAIN
BRAIN, OLFACTORY BULB
BRAIN, PONS
BRAIN, THALAMUS
BRAIN, VENTRICULAR SYSTEM
BRAIN, WHITE MATTER
EYE
GANGLION, DORSAL ROOT, CERVICAL
GANGLION, DORSAL ROOT, LUMBAR
GANGLION, DORSAL ROOT, THORACIC
HEART
LUNG
LYMPH NODE, ILIAC
LYMPH NODE, MANDIBULAR
LYMPH NODE, MESENTERIC
MUSCLE, BICEPS FEMORIS
MUSCLE, GASTROCNEMIUS
NERVE ROOT, SPINAL, CERVICAL
NERVE ROOT, SPINAL, LUMBAR
NERVE ROOT, SPINAL, THORACIC
NERVE, OPTIC
NERVE, SCIATIC
OVARY
PANCREAS
SPINAL CORD, CERVICAL
SPINAL CORD, THORACIC

Appendix 10

Individual Animal Report

Study ID: 02776-0018: CRL 2954-001 / U of Tex Southwestern Med UTSW.Gray-002
Study Title: A SINGLE DOSE TOXICITY STUDY OF AAV9/SURF1 ADMINISTERED BY INTRATHECAL INJECTION IN RATS

Animal: 3514 (Female)

Sacrifice: DAY 08
Group: 3FD08 / AAV9/SURF1 MID DOSE (0.83E12 VG)
Fate: SCHEDULED SACRIFICE / Time on Test: 08 / Cause(s) of Death:

Gross or Microscopic Finding
Disposition
Tissue: Site / Finding, Severity

SPLEEN
THYMUS

Disposition: **ABNORMAL/FINDING**

KIDNEY
KIDNEY, TUBULAR: BASOPHILIA, MINIMAL
LIVER
LIVER: INFILTRATE, MIXED, MINIMAL
NERVE, TIBIAL
NERVE, TIBIAL: DEGENERATION, NERVE FIBER, MINIMAL
SPINAL CORD, LUMBAR
SPINAL CORD, LUMBAR, NERVE ROOT, SPINAL: DEGENERATION, NERVE FIBER, MINIMAL

Appendix 10

Individual Animal Report

Study ID: 02776-0018: CRL 2954-001 / U of Tex Southwestern Med UTSW.Gray-002
Study Title: A SINGLE DOSE TOXICITY STUDY OF AAV9/SURF1 ADMINISTERED BY INTRATHECAL INJECTION IN RATS

Animal: 3515 (Female)

Sacrifice: DAY 08
Group: 3FD08 / AAV9/SURF1 MID DOSE (0.83E12 VG)
Fate: SCHEDULED SACRIFICE / Time on Test: 08 / Cause(s) of Death:

Gross or Microscopic Finding
Disposition
Tissue: Site / Finding, Severity

MICROSCOPIC EXAMINATION

Disposition: **NORMAL**

BRAIN, AMYGDALOID BODY
BRAIN, BASAL NUCLEI/STRIATUM
BRAIN, CEREBELLUM
BRAIN, CEREBRAL CORTEX
BRAIN, HIPPOCAMPUS
BRAIN, HYPOTHALAMUS
BRAIN, MEDULLA OBLONGATA
BRAIN, MENINGES
BRAIN, MIDBRAIN
BRAIN, OLFACTORY BULB
BRAIN, PONS
BRAIN, THALAMUS
BRAIN, VENTRICULAR SYSTEM
BRAIN, WHITE MATTER
EYE
GANGLION, DORSAL ROOT, CERVICAL
GANGLION, DORSAL ROOT, LUMBAR
GANGLION, DORSAL ROOT, THORACIC
HEART
KIDNEY
LUNG
LYMPH NODE, ILIAC
LYMPH NODE, MANDIBULAR
LYMPH NODE, MESENTERIC
MUSCLE, BICEPS FEMORIS
MUSCLE, GASTROCNEMIUS
NERVE ROOT, SPINAL, CERVICAL
NERVE ROOT, SPINAL, THORACIC
NERVE, OPTIC
OVARY
PANCREAS
SPINAL CORD, CERVICAL
SPINAL CORD, LUMBAR
SPLEEN

Appendix 10

Individual Animal Report

Study ID: 02776-0018: CRL 2954-001 / U of Tex Southwestern Med UTSW.Gray-002
Study Title: A SINGLE DOSE TOXICITY STUDY OF AAV9/SURF1 ADMINISTERED BY INTRATHECAL INJECTION IN RATS

Animal: 3515 (Female)

Sacrifice: DAY 08
Group: 3FD08 / AAV9/SURF1 MID DOSE (0.83E12 VG)
Fate: SCHEDULED SACRIFICE / Time on Test: 08 / Cause(s) of Death:

Gross or Microscopic Finding
Disposition
Tissue: Site / Finding, Severity

THYMUS

Disposition: **ABNORMAL/FINDING**

LIVER
LIVER: EXTRAMEDULLARY HEMATOPOIESIS, MINIMAL
LIVER: INFILTRATE, MIXED, MINIMAL
NERVE ROOT, SPINAL, LUMBAR
NERVE ROOT, SPINAL, LUMBAR, EPINEURIUM: INFILTRATE, MONONUCLEAR CELLS, MINIMAL, FOCAL
NERVE, SCIATIC
NERVE, SCIATIC: DEGENERATION, NERVE FIBER, MINIMAL
NERVE, TIBIAL
NERVE, TIBIAL: DEGENERATION, NERVE FIBER, MINIMAL
SPINAL CORD, THORACIC
SPINAL CORD, THORACIC, WHITE MATTER: DEGENERATION, NERVE FIBER, MINIMAL
COMMENT(S): SEEN IN LONG SECTION AND EXACT LOCATION COULD NOT BE DETERMINED

Appendix 10

Individual Animal Report

Study ID: 02776-0018: CRL 2954-001 / U of Tex Southwestern Med UTSW.Gray-002
Study Title: A SINGLE DOSE TOXICITY STUDY OF AAV9/SURF1 ADMINISTERED BY INTRATHECAL INJECTION IN RATS

Animal: 4511 (Female)

Sacrifice: DAY 08
Group: 4FD08 / AAV9/SURF1 HIGH DOSE (2.49E12 VG)
Fate: SCHEDULED SACRIFICE / Time on Test: 08 / Cause(s) of Death:

Gross or Microscopic Finding
Disposition
Tissue: Site / Finding, Severity

MICROSCOPIC EXAMINATION

Disposition: NORMAL

BRAIN, AMYGDALOID BODY
BRAIN, BASAL NUCLEI/STRIATUM
BRAIN, CEREBELLUM
BRAIN, CEREBRAL CORTEX
BRAIN, HIPPOCAMPUS
BRAIN, HYPOTHALAMUS
BRAIN, MEDULLA OBLONGATA
BRAIN, MENINGES
BRAIN, MIDBRAIN
BRAIN, OLFACTORY BULB
BRAIN, PONS
BRAIN, THALAMUS
BRAIN, VENTRICULAR SYSTEM
BRAIN, WHITE MATTER
EYE
GANGLION, DORSAL ROOT, THORACIC
HEART
LUNG
LYMPH NODE, MANDIBULAR
LYMPH NODE, MESENTERIC
MUSCLE, BICEPS FEMORIS
MUSCLE, GASTROCNEMIUS
NERVE ROOT, SPINAL, CERVICAL
NERVE ROOT, SPINAL, THORACIC
NERVE, OPTIC
NERVE, SCIATIC
NERVE, TIBIAL
OVARY
PANCREAS
SPINAL CORD, CERVICAL
SPINAL CORD, LUMBAR
SPINAL CORD, THORACIC
SPLEEN
THYMUS

Appendix 10

Individual Animal Report

Study ID: 02776-0018: CRL 2954-001 / U of Tex Southwestern Med UTSW.Gray-002
Study Title: A SINGLE DOSE TOXICITY STUDY OF AAV9/SURF1 ADMINISTERED BY INTRATHECAL INJECTION IN RATS

Animal: 4511 (Female)

Sacrifice: DAY 08
Group: 4FD08 / AAV9/SURF1 HIGH DOSE (2.49E12 VG)
Fate: SCHEDULED SACRIFICE / Time on Test: 08 / Cause(s) of Death:

Gross or Microscopic Finding
Disposition
Tissue: Site / Finding, Severity

Disposition: ABNORMAL/FINDING

GANGLION, DORSAL ROOT, CERVICAL
GANGLION, DORSAL ROOT, CERVICAL, GLIAL CELL: HYPERTROPHY/HYPERPLASIA, MINIMAL
GANGLION, DORSAL ROOT, LUMBAR
GANGLION, DORSAL ROOT, LUMBAR, GLIAL CELL: HYPERTROPHY/HYPERPLASIA, MINIMAL
KIDNEY
KIDNEY: CHRONIC PROGRESSIVE NEPHROPATHY, MINIMAL
LIVER
LIVER: INFILTRATE, MIXED, MINIMAL
NERVE ROOT, SPINAL, LUMBAR
NERVE ROOT, SPINAL, LUMBAR, EPINEURIUM: INFILTRATE, MONONUCLEAR CELLS, MINIMAL, FOCAL

Disposition: UNABLE TO OBTAIN SPECIMEN

LYMPH NODE, ILIAC

Appendix 10

Individual Animal Report

Study ID: 02776-0018: CRL 2954-001 / U of Tex Southwestern Med UTSW.Gray-002
Study Title: A SINGLE DOSE TOXICITY STUDY OF AAV9/SURF1 ADMINISTERED BY INTRATHECAL INJECTION IN RATS

Animal: 4512 (Female)

Sacrifice: DAY 08
Group: 4FD08 / AAV9/SURF1 HIGH DOSE (2.49E12 VG)
Fate: SCHEDULED SACRIFICE / Time on Test: 08 / Cause(s) of Death:

Gross or Microscopic Finding
Disposition
Tissue: Site / Finding, Severity

MICROSCOPIC EXAMINATION

Disposition: NORMAL

BRAIN, AMYGDALOID BODY
BRAIN, BASAL NUCLEI/STRIATUM
BRAIN, CEREBELLUM
BRAIN, CEREBRAL CORTEX
BRAIN, HIPPOCAMPUS
BRAIN, HYPOTHALAMUS
BRAIN, MEDULLA OBLONGATA
BRAIN, MENINGES
BRAIN, MIDBRAIN
BRAIN, OLFACTORY BULB
BRAIN, PONS
BRAIN, THALAMUS
BRAIN, VENTRICULAR SYSTEM
BRAIN, WHITE MATTER
EYE
GANGLION, DORSAL ROOT, CERVICAL
GANGLION, DORSAL ROOT, LUMBAR
GANGLION, DORSAL ROOT, THORACIC
KIDNEY
LUNG
LYMPH NODE, ILIAC
LYMPH NODE, MANDIBULAR
LYMPH NODE, MESENTERIC
MUSCLE, BICEPS FEMORIS
MUSCLE, GASTROCNEMIUS
NERVE ROOT, SPINAL, CERVICAL
NERVE ROOT, SPINAL, LUMBAR
NERVE ROOT, SPINAL, THORACIC
NERVE, OPTIC
NERVE, SCIATIC
NERVE, TIBIAL
OVARY
PANCREAS
SPINAL CORD, CERVICAL

Appendix 10

Individual Animal Report

Study ID: 02776-0018: CRL 2954-001 / U of Tex Southwestern Med UTSW.Gray-002
Study Title: A SINGLE DOSE TOXICITY STUDY OF AAV9/SURF1 ADMINISTERED BY INTRATHECAL INJECTION IN RATS

Animal: 4512 (Female)

Sacrifice: DAY 08
Group: 4FD08 / AAV9/SURF1 HIGH DOSE (2.49E12 VG)
Fate: SCHEDULED SACRIFICE / Time on Test: 08 / Cause(s) of Death:

Gross or Microscopic Finding
Disposition
Tissue: Site / Finding, Severity

SPINAL CORD, LUMBAR
SPINAL CORD, THORACIC
SPLEEN
THYMUS

Disposition: **ABNORMAL/FINDING**

HEART
HEART, MYOCARDIUM: DEGENERATION/NECROSIS, MINIMAL, FOCAL
HEART, MYOCARDIUM: INFILTRATE, MONONUCLEAR CELLS, MINIMAL, MULTIFOCAL
LIVER
LIVER: INFILTRATE, MIXED, MINIMAL

Appendix 10

Individual Animal Report

Study ID: 02776-0018: CRL 2954-001 / U of Tex Southwestern Med UTSW.Gray-002
Study Title: A SINGLE DOSE TOXICITY STUDY OF AAV9/SURF1 ADMINISTERED BY INTRATHECAL INJECTION IN RATS

Animal: 4513 (Female)

Sacrifice: DAY 08
Group: 4FD08 / AAV9/SURF1 HIGH DOSE (2.49E12 VG)
Fate: SCHEDULED SACRIFICE / Time on Test: 08 / Cause(s) of Death:

Gross or Microscopic Finding
Disposition
Tissue: Site / Finding, Severity

MICROSCOPIC EXAMINATION

Disposition: NORMAL

BRAIN, AMYGDALOID BODY
BRAIN, BASAL NUCLEI/STRIATUM
BRAIN, CEREBELLUM
BRAIN, CEREBRAL CORTEX
BRAIN, HIPPOCAMPUS
BRAIN, HYPOTHALAMUS
BRAIN, MEDULLA OBLONGATA
BRAIN, MENINGES
BRAIN, MIDBRAIN
BRAIN, OLFACTORY BULB
BRAIN, PONS
BRAIN, THALAMUS
BRAIN, VENTRICULAR SYSTEM
BRAIN, WHITE MATTER
EYE
GANGLION, DORSAL ROOT, CERVICAL
GANGLION, DORSAL ROOT, LUMBAR
GANGLION, DORSAL ROOT, THORACIC
KIDNEY
LUNG
LYMPH NODE, ILIAC
LYMPH NODE, MANDIBULAR
LYMPH NODE, MESENTERIC
MUSCLE, BICEPS FEMORIS
MUSCLE, GASTROCNEMIUS
NERVE ROOT, SPINAL, CERVICAL
NERVE ROOT, SPINAL, LUMBAR
NERVE ROOT, SPINAL, THORACIC
NERVE, OPTIC
NERVE, SCIATIC
NERVE, TIBIAL
OVARY
PANCREAS
SPINAL CORD, CERVICAL

Appendix 10

Individual Animal Report

Study ID: 02776-0018: CRL 2954-001 / U of Tex Southwestern Med UTSW.Gray-002
Study Title: A SINGLE DOSE TOXICITY STUDY OF AAV9/SURF1 ADMINISTERED BY INTRATHECAL INJECTION IN RATS

Animal: 4513 (Female)

Sacrifice: DAY 08
Group: 4FD08 / AAV9/SURF1 HIGH DOSE (2.49E12 VG)
Fate: SCHEDULED SACRIFICE / Time on Test: 08 / Cause(s) of Death:

Gross or Microscopic Finding
Disposition
Tissue: Site / Finding, Severity

SPINAL CORD, LUMBAR
SPINAL CORD, THORACIC
SPLEEN
THYMUS

Disposition: **ABNORMAL/FINDING**

HEART
HEART, MYOCARDIUM: INFILTRATE, MONONUCLEAR CELLS, MINIMAL, MULTIFOCAL
LIVER
LIVER: HYPERTROPHY/HYPERPLASIA, MINIMAL
COMMENT(S): AFFECTING KUPFFER CELLS
LIVER: INFILTRATE, MIXED, MINIMAL
LIVER, HEPATOCYTE: VACUOLATION, MINIMAL
COMMENT(S): AFFECTING HEPATOCYTES IN PERIPORTAL REGIONS

Appendix 10

Individual Animal Report

Study ID: 02776-0018: CRL 2954-001 / U of Tex Southwestern Med UTSW.Gray-002
Study Title: A SINGLE DOSE TOXICITY STUDY OF AAV9/SURF1 ADMINISTERED BY INTRATHECAL INJECTION IN RATS

Animal: 4514 (Female)

Sacrifice: DAY 08
Group: 4FD08 / AAV9/SURF1 HIGH DOSE (2.49E12 VG)
Fate: SCHEDULED SACRIFICE / Time on Test: 08 / Cause(s) of Death:

Gross or Microscopic Finding
Disposition
Tissue: Site / Finding, Severity

MICROSCOPIC EXAMINATION

Disposition: NORMAL

BRAIN, AMYGDALOID BODY
BRAIN, BASAL NUCLEI/STRIATUM
BRAIN, CEREBELLUM
BRAIN, CEREBRAL CORTEX
BRAIN, HIPPOCAMPUS
BRAIN, HYPOTHALAMUS
BRAIN, MENINGES
BRAIN, MIDBRAIN
BRAIN, OLFACTORY BULB
BRAIN, PONS
BRAIN, THALAMUS
BRAIN, VENTRICULAR SYSTEM
BRAIN, WHITE MATTER
EYE
GANGLION, DORSAL ROOT, CERVICAL
GANGLION, DORSAL ROOT, THORACIC
HEART
KIDNEY
LUNG
LYMPH NODE, ILIAC
LYMPH NODE, MANDIBULAR
LYMPH NODE, MESENTERIC
MUSCLE, BICEPS FEMORIS
MUSCLE, GASTROCNEMIUS
NERVE ROOT, SPINAL, CERVICAL
NERVE ROOT, SPINAL, THORACIC
NERVE, OPTIC
NERVE, SCIATIC
NERVE, TIBIAL
OVARY
PANCREAS
SPINAL CORD, CERVICAL
SPINAL CORD, LUMBAR
SPINAL CORD, THORACIC

Appendix 10

Individual Animal Report

Study ID: 02776-0018: CRL 2954-001 / U of Tex Southwestern Med UTSW.Gray-002
Study Title: A SINGLE DOSE TOXICITY STUDY OF AAV9/SURF1 ADMINISTERED BY INTRATHECAL INJECTION IN RATS

Animal: 4514 (Female)

Sacrifice: DAY 08
Group: 4FD08 / AAV9/SURF1 HIGH DOSE (2.49E12 VG)
Fate: SCHEDULED SACRIFICE / Time on Test: 08 / Cause(s) of Death:

Gross or Microscopic Finding
Disposition
Tissue: Site / Finding, Severity

SPLEEN
THYMUS

Disposition: ABNORMAL/FINDING

GANGLION, DORSAL ROOT, LUMBAR
GANGLION, DORSAL ROOT, LUMBAR, GLIAL CELL: HYPERTROPHY/HYPERPLASIA, MINIMAL
LIVER
LIVER: INFILTRATE, MIXED, MINIMAL
NERVE ROOT, SPINAL, LUMBAR
NERVE ROOT, SPINAL, LUMBAR, EPINEURIUM: INFILTRATE, MONONUCLEAR CELLS, MINIMAL, FOCAL

Disposition: UNABLE TO OBTAIN SPECIMEN

BRAIN, MEDULLA OBLONGATA

Appendix 10

Individual Animal Report

Study ID: 02776-0018: CRL 2954-001 / U of Tex Southwestern Med UTSW.Gray-002
Study Title: A SINGLE DOSE TOXICITY STUDY OF AAV9/SURF1 ADMINISTERED BY INTRATHECAL INJECTION IN RATS

Animal: 4515 (Female)

Sacrifice: DAY 08
Group: 4FD08 / AAV9/SURF1 HIGH DOSE (2.49E12 VG)
Fate: SCHEDULED SACRIFICE / Time on Test: 08 / Cause(s) of Death:

Gross or Microscopic Finding
Disposition
Tissue: Site / Finding, Severity

MICROSCOPIC EXAMINATION

Disposition: NORMAL

BRAIN, AMYGDALOID BODY
BRAIN, BASAL NUCLEI/STRIATUM
BRAIN, CEREBELLUM
BRAIN, CEREBRAL CORTEX
BRAIN, HIPPOCAMPUS
BRAIN, HYPOTHALAMUS
BRAIN, MEDULLA OBLONGATA
BRAIN, MENINGES
BRAIN, MIDBRAIN
BRAIN, OLFACTORY BULB
BRAIN, PONS
BRAIN, THALAMUS
BRAIN, VENTRICULAR SYSTEM
BRAIN, WHITE MATTER
EYE
KIDNEY
LUNG
LYMPH NODE, ILIAC
LYMPH NODE, MANDIBULAR
LYMPH NODE, MESENTERIC
MUSCLE, BICEPS FEMORIS
MUSCLE, GASTROCNEMIUS
NERVE ROOT, SPINAL, CERVICAL
NERVE ROOT, SPINAL, THORACIC
NERVE, OPTIC
NERVE, SCIATIC
NERVE, TIBIAL
OVARY
PANCREAS
SPINAL CORD, CERVICAL
SPINAL CORD, LUMBAR
SPINAL CORD, THORACIC
SPLEEN
THYMUS

Appendix 10

Individual Animal Report

Study ID: 02776-0018: CRL 2954-001 / U of Tex Southwestern Med UTSW.Gray-002
Study Title: A SINGLE DOSE TOXICITY STUDY OF AAV9/SURF1 ADMINISTERED BY INTRATHECAL INJECTION IN RATS

Animal: 4515 (Female)

Sacrifice: DAY 08
Group: 4FD08 / AAV9/SURF1 HIGH DOSE (2.49E12 VG)
Fate: SCHEDULED SACRIFICE / Time on Test: 08 / Cause(s) of Death:

Gross or Microscopic Finding
Disposition
Tissue: Site / Finding, Severity

Disposition: **ABNORMAL/FINDING**

GANGLION, DORSAL ROOT, CERVICAL
GANGLION, DORSAL ROOT, CERVICAL, GLIAL CELL: HYPERTROPHY/HYPERPLASIA, MINIMAL
GANGLION, DORSAL ROOT, LUMBAR
GANGLION, DORSAL ROOT, LUMBAR, GLIAL CELL: HYPERTROPHY/HYPERPLASIA, MINIMAL
GANGLION, DORSAL ROOT, THORACIC
GANGLION, DORSAL ROOT, THORACIC, GLIAL CELL: HYPERTROPHY/HYPERPLASIA, MINIMAL
HEART
HEART, MYOCARDIUM: DEGENERATION/NECROSIS, MINIMAL, FOCAL
HEART, MYOCARDIUM: INFILTRATE, MONONUCLEAR CELLS, MINIMAL, FOCAL
LIVER
LIVER: INFILTRATE, MIXED, MINIMAL
NERVE ROOT, SPINAL, LUMBAR
NERVE ROOT, SPINAL, LUMBAR, EPINEURIUM: INFILTRATE, MONONUCLEAR CELLS, MINIMAL, FOCAL

Appendix 10

Individual Animal Report

Study ID: 02776-0018: CRL 2954-001 / U of Tex Southwestern Med UTSW.Gray-002
Study Title: A SINGLE DOSE TOXICITY STUDY OF AAV9/SURF1 ADMINISTERED BY INTRATHECAL INJECTION IN RATS

Animal: 1021 (Male)

Sacrifice: DAY 29
Group: 1MD29 / VEHICLE (0 VG)
Fate: SCHEDULED SACRIFICE / Time on Test: 29 / Cause(s) of Death:

Gross or Microscopic Finding
Disposition
Tissue: Site / Finding, Severity

MICROSCOPIC EXAMINATION

Disposition: NORMAL

BRAIN, AMYGDALOID BODY
BRAIN, BASAL NUCLEI/STRIATUM
BRAIN, CEREBELLUM
BRAIN, CEREBRAL CORTEX
BRAIN, HIPPOCAMPUS
BRAIN, HYPOTHALAMUS
BRAIN, MEDULLA OBLONGATA
BRAIN, MENINGES
BRAIN, MIDBRAIN
BRAIN, OLFACTORY BULB
BRAIN, PONS
BRAIN, THALAMUS
BRAIN, VENTRICULAR SYSTEM
BRAIN, WHITE MATTER
EYE
GANGLION, DORSAL ROOT, THORACIC
HEART
LUNG
LYMPH NODE, ILIAC
LYMPH NODE, MANDIBULAR
LYMPH NODE, MESENTERIC
MUSCLE, BICEPS FEMORIS
MUSCLE, GASTROCNEMIUS
NERVE ROOT, SPINAL, CERVICAL
NERVE ROOT, SPINAL, THORACIC
NERVE, OPTIC
NERVE, SCIATIC
NERVE, TIBIAL
PANCREAS
SPINAL CORD, CERVICAL
SPINAL CORD, LUMBAR
SPINAL CORD, THORACIC
SPLEEN
TESTIS

Appendix 10

Individual Animal Report

Study ID: 02776-0018: CRL 2954-001 / U of Tex Southwestern Med UTSW.Gray-002
Study Title: A SINGLE DOSE TOXICITY STUDY OF AAV9/SURF1 ADMINISTERED BY INTRATHECAL INJECTION IN RATS

Animal: 1021 (Male)

Sacrifice: DAY 29
Group: 1MD29 / VEHICLE (0 VG)
Fate: SCHEDULED SACRIFICE / Time on Test: 29 / Cause(s) of Death:

Gross or Microscopic Finding
Disposition
Tissue: Site / Finding, Severity

THYMUS

Disposition: **ABNORMAL/FINDING**

GANGLION, DORSAL ROOT, CERVICAL
GANGLION, DORSAL ROOT, CERVICAL, GLIAL CELL: HYPERTROPHY/HYPERPLASIA, MINIMAL
GANGLION, DORSAL ROOT, LUMBAR
GANGLION, DORSAL ROOT, LUMBAR, GLIAL CELL: HYPERTROPHY/HYPERPLASIA, MINIMAL
KIDNEY
KIDNEY: CHRONIC PROGRESSIVE NEPHROPATHY, MINIMAL
LIVER
LIVER: INFILTRATE, MIXED, MINIMAL
NERVE ROOT, SPINAL, LUMBAR
NERVE ROOT, SPINAL, LUMBAR, EPINEURIUM: INFILTRATE, MONONUCLEAR CELLS, MINIMAL, FOCAL

Appendix 10

Individual Animal Report

Study ID: 02776-0018: CRL 2954-001 / U of Tex Southwestern Med UTSW.Gray-002
Study Title: A SINGLE DOSE TOXICITY STUDY OF AAV9/SURF1 ADMINISTERED BY INTRATHECAL INJECTION IN RATS

Animal: 1022 (Male)

Sacrifice: DAY 29
Group: 1MD29 / VEHICLE (0 VG)
Fate: SCHEDULED SACRIFICE / Time on Test: 29 / Cause(s) of Death:

Gross or Microscopic Finding
Disposition
Tissue: Site / Finding, Severity

MICROSCOPIC EXAMINATION

Disposition: NORMAL

BRAIN, AMYGDALOID BODY
BRAIN, BASAL NUCLEI/STRIATUM
BRAIN, CEREBELLUM
BRAIN, CEREBRAL CORTEX
BRAIN, HIPPOCAMPUS
BRAIN, HYPOTHALAMUS
BRAIN, MEDULLA OBLONGATA
BRAIN, MENINGES
BRAIN, MIDBRAIN
BRAIN, OLFACTORY BULB
BRAIN, PONS
BRAIN, THALAMUS
BRAIN, VENTRICULAR SYSTEM
BRAIN, WHITE MATTER
EYE
GANGLION, DORSAL ROOT, CERVICAL
GANGLION, DORSAL ROOT, LUMBAR
GANGLION, DORSAL ROOT, THORACIC
HEART
LUNG
LYMPH NODE, MANDIBULAR
LYMPH NODE, MESENTERIC
MUSCLE, BICEPS FEMORIS
MUSCLE, GASTROCNEMIUS
NERVE ROOT, SPINAL, CERVICAL
NERVE ROOT, SPINAL, LUMBAR
NERVE ROOT, SPINAL, THORACIC
NERVE, OPTIC
NERVE, SCIATIC
NERVE, TIBIAL
PANCREAS
SPINAL CORD, CERVICAL
SPINAL CORD, LUMBAR
SPINAL CORD, THORACIC

Appendix 10

Individual Animal Report

Study ID: 02776-0018: CRL 2954-001 / U of Tex Southwestern Med UTSW.Gray-002
Study Title: A SINGLE DOSE TOXICITY STUDY OF AAV9/SURF1 ADMINISTERED BY INTRATHECAL INJECTION IN RATS

Animal: 1022 (Male)

Sacrifice: DAY 29
Group: 1MD29 / VEHICLE (0 VG)
Fate: SCHEDULED SACRIFICE / Time on Test: 29 / Cause(s) of Death:

Gross or Microscopic Finding
Disposition
Tissue: Site / Finding, Severity

SPLEEN
TESTIS
THYMUS

Disposition: **ABNORMAL/FINDING**

KIDNEY
KIDNEY: CHRONIC PROGRESSIVE NEPHROPATHY, MINIMAL
LIVER
LIVER: INFILTRATE, MIXED, MINIMAL

Disposition: **UNABLE TO OBTAIN SPECIMEN**

LYMPH NODE, ILIAC

Appendix 10

Individual Animal Report

Study ID: 02776-0018: CRL 2954-001 / U of Tex Southwestern Med UTSW.Gray-002
Study Title: A SINGLE DOSE TOXICITY STUDY OF AAV9/SURF1 ADMINISTERED BY INTRATHECAL INJECTION IN RATS

Animal: 1023 (Male)

Sacrifice: DAY 29
Group: 1MD29 / VEHICLE (0 VG)
Fate: SCHEDULED SACRIFICE / Time on Test: 29 / Cause(s) of Death:

Gross or Microscopic Finding
Disposition
Tissue: Site / Finding, Severity

MICROSCOPIC EXAMINATION

Disposition: NORMAL

BRAIN, AMYGDALOID BODY
BRAIN, BASAL NUCLEI/STRIATUM
BRAIN, CEREBELLUM
BRAIN, CEREBRAL CORTEX
BRAIN, HIPPOCAMPUS
BRAIN, HYPOTHALAMUS
BRAIN, MENINGES
BRAIN, MIDBRAIN
BRAIN, OLFACTORY BULB
BRAIN, PONS
BRAIN, THALAMUS
BRAIN, VENTRICULAR SYSTEM
BRAIN, WHITE MATTER
EYE
GANGLION, DORSAL ROOT, CERVICAL
GANGLION, DORSAL ROOT, LUMBAR
GANGLION, DORSAL ROOT, THORACIC
LUNG
LYMPH NODE, ILIAC
LYMPH NODE, MANDIBULAR
LYMPH NODE, MESENTERIC
MUSCLE, BICEPS FEMORIS
MUSCLE, GASTROCNEMIUS
NERVE ROOT, SPINAL, CERVICAL
NERVE ROOT, SPINAL, THORACIC
NERVE, OPTIC
NERVE, SCIATIC
NERVE, TIBIAL
PANCREAS
SPINAL CORD, CERVICAL
SPINAL CORD, LUMBAR
SPINAL CORD, THORACIC
SPLEEN
TESTIS

Appendix 10

Individual Animal Report

Study ID: 02776-0018: CRL 2954-001 / U of Tex Southwestern Med UTSW.Gray-002
Study Title: A SINGLE DOSE TOXICITY STUDY OF AAV9/SURF1 ADMINISTERED BY INTRATHECAL INJECTION IN RATS

Animal: 1023 (Male)

Sacrifice: DAY 29
Group: 1MD29 / VEHICLE (0 VG)
Fate: SCHEDULED SACRIFICE / Time on Test: 29 / Cause(s) of Death:

Gross or Microscopic Finding
Disposition
Tissue: Site / Finding, Severity

THYMUS

Disposition: **ABNORMAL/FINDING**

BRAIN, MEDULLA OBLONGATA
BRAIN, MEDULLA OBLONGATA, WHITE MATTER: DEGENERATION, NERVE FIBER, MINIMAL
COMMENT(S): PYRAMIDAL TRACT

HEART
HEART, MYOCARDIUM: DEGENERATION/NECROSIS, MINIMAL, MULTIFOCAL
HEART, MYOCARDIUM: INFILTRATE, MONONUCLEAR CELLS, MINIMAL, MULTIFOCAL

KIDNEY
KIDNEY: CHRONIC PROGRESSIVE NEPHROPATHY, MINIMAL

LIVER
LIVER: INFILTRATE, MIXED, MINIMAL

NERVE ROOT, SPINAL, LUMBAR
NERVE ROOT, SPINAL, LUMBAR, NERVE ROOT, DORSAL: DEGENERATION, NERVE FIBER, MINIMAL

Appendix 10

Individual Animal Report

Study ID: 02776-0018: CRL 2954-001 / U of Tex Southwestern Med UTSW.Gray-002
Study Title: A SINGLE DOSE TOXICITY STUDY OF AAV9/SURF1 ADMINISTERED BY INTRATHECAL INJECTION IN RATS

Animal: 1024 (Male)

Sacrifice: DAY 29
Group: 1MD29 / VEHICLE (0 VG)
Fate: SCHEDULED SACRIFICE / Time on Test: 29 / Cause(s) of Death:

Gross or Microscopic Finding
Disposition
Tissue: Site / Finding, Severity

MICROSCOPIC EXAMINATION

Disposition: NORMAL

BRAIN, AMYGDALOID BODY
BRAIN, BASAL NUCLEI/STRIATUM
BRAIN, CEREBELLUM
BRAIN, CEREBRAL CORTEX
BRAIN, HIPPOCAMPUS
BRAIN, HYPOTHALAMUS
BRAIN, MEDULLA OBLONGATA
BRAIN, MENINGES
BRAIN, MIDBRAIN
BRAIN, OLFACTORY BULB
BRAIN, PONS
BRAIN, THALAMUS
BRAIN, VENTRICULAR SYSTEM
BRAIN, WHITE MATTER
EYE
GANGLION, DORSAL ROOT, THORACIC
HEART
KIDNEY
LUNG
LYMPH NODE, ILIAC
LYMPH NODE, MANDIBULAR
LYMPH NODE, MESENTERIC
MUSCLE, BICEPS FEMORIS
MUSCLE, GASTROCNEMIUS
NERVE ROOT, SPINAL, CERVICAL
NERVE ROOT, SPINAL, THORACIC
NERVE, OPTIC
NERVE, SCIATIC
NERVE, TIBIAL
PANCREAS
SPINAL CORD, LUMBAR
SPINAL CORD, THORACIC
SPLEEN
THYMUS

Appendix 10

Individual Animal Report

Study ID: 02776-0018: CRL 2954-001 / U of Tex Southwestern Med UTSW.Gray-002
Study Title: A SINGLE DOSE TOXICITY STUDY OF AAV9/SURF1 ADMINISTERED BY INTRATHECAL INJECTION IN RATS

Animal: 1024 (Male)

Sacrifice: DAY 29
Group: 1MD29 / VEHICLE (0 VG)
Fate: SCHEDULED SACRIFICE / Time on Test: 29 / Cause(s) of Death:

Gross or Microscopic Finding
Disposition
Tissue: Site / Finding, Severity

Disposition: ABNORMAL/FINDING

GANGLION, DORSAL ROOT, CERVICAL
GANGLION, DORSAL ROOT, CERVICAL, GLIAL CELL: HYPERTROPHY/HYPERPLASIA, MINIMAL
GANGLION, DORSAL ROOT, LUMBAR
GANGLION, DORSAL ROOT, LUMBAR, GLIAL CELL: HYPERTROPHY/HYPERPLASIA, MINIMAL
LIVER
LIVER: INFILTRATE, MIXED, MINIMAL
NERVE ROOT, SPINAL, LUMBAR
NERVE ROOT, SPINAL, LUMBAR, EPINEURIUM: INFILTRATE, MONONUCLEAR CELLS, MINIMAL, FOCAL
SPINAL CORD, CERVICAL
SPINAL CORD, CERVICAL, WHITE MATTER: DEGENERATION, NERVE FIBER, MINIMAL, DORSAL
TESTIS
TESTIS, TUBULAR: DEGENERATION, MINIMAL

Appendix 10

Individual Animal Report

Study ID: 02776-0018: CRL 2954-001 / U of Tex Southwestern Med UTSW.Gray-002
Study Title: A SINGLE DOSE TOXICITY STUDY OF AAV9/SURF1 ADMINISTERED BY INTRATHECAL INJECTION IN RATS

Animal: 1025 (Male)

Sacrifice: DAY 29
Group: 1MD29 / VEHICLE (0 VG)
Fate: SCHEDULED SACRIFICE / Time on Test: 29 / Cause(s) of Death:

Gross or Microscopic Finding
Disposition
Tissue: Site / Finding, Severity

MICROSCOPIC EXAMINATION

Disposition: NORMAL

BRAIN, AMYGDALOID BODY
BRAIN, BASAL NUCLEI/STRIATUM
BRAIN, CEREBELLUM
BRAIN, CEREBRAL CORTEX
BRAIN, HIPPOCAMPUS
BRAIN, HYPOTHALAMUS
BRAIN, MEDULLA OBLONGATA
BRAIN, MENINGES
BRAIN, MIDBRAIN
BRAIN, OLFACTORY BULB
BRAIN, PONS
BRAIN, THALAMUS
BRAIN, VENTRICULAR SYSTEM
BRAIN, WHITE MATTER
EYE
GANGLION, DORSAL ROOT, THORACIC
HEART
LIVER
LUNG
LYMPH NODE, ILIAC
LYMPH NODE, MANDIBULAR
LYMPH NODE, MESENTERIC
MUSCLE, BICEPS FEMORIS
MUSCLE, GASTROCNEMIUS
NERVE ROOT, SPINAL, CERVICAL
NERVE ROOT, SPINAL, LUMBAR
NERVE ROOT, SPINAL, THORACIC
NERVE, OPTIC
NERVE, SCIATIC
NERVE, TIBIAL
PANCREAS
SPINAL CORD, CERVICAL
SPINAL CORD, THORACIC
SPLEEN

Appendix 10

Individual Animal Report

Study ID: 02776-0018: CRL 2954-001 / U of Tex Southwestern Med UTSW.Gray-002
Study Title: A SINGLE DOSE TOXICITY STUDY OF AAV9/SURF1 ADMINISTERED BY INTRATHECAL INJECTION IN RATS

Animal: 1025 (Male)

Sacrifice: DAY 29
Group: 1MD29 / VEHICLE (0 VG)
Fate: SCHEDULED SACRIFICE / Time on Test: 29 / Cause(s) of Death:

Gross or Microscopic Finding
Disposition
Tissue: Site / Finding, Severity

TESTIS
THYMUS

Disposition: **ABNORMAL/FINDING**

GANGLION, DORSAL ROOT, CERVICAL
GANGLION, DORSAL ROOT, CERVICAL, GLIAL CELL: HYPERTROPHY/HYPERPLASIA, MINIMAL
GANGLION, DORSAL ROOT, LUMBAR
GANGLION, DORSAL ROOT, LUMBAR, GLIAL CELL: HYPERTROPHY/HYPERPLASIA, MINIMAL, FOCAL
KIDNEY
KIDNEY: CHRONIC PROGRESSIVE NEPHROPATHY, MINIMAL
KIDNEY, TUBULAR: CYST, MINIMAL
SPINAL CORD, LUMBAR
SPINAL CORD, LUMBAR, WHITE MATTER: DEGENERATION, NERVE FIBER, MINIMAL
COMMENT(S): SEEN IN LONG SECTION AND EXACT LOCATION COULD NOT BE DETERMINED

Appendix 10

Individual Animal Report

Study ID: 02776-0018: CRL 2954-001 / U of Tex Southwestern Med UTSW.Gray-002
Study Title: A SINGLE DOSE TOXICITY STUDY OF AAV9/SURF1 ADMINISTERED BY INTRATHECAL INJECTION IN RATS

Animal: 2021 (Male)

Sacrifice: DAY 29
Group: 2MD29 / AAV9/SURF1 LOW DOSE (0.28E12 VG)
Fate: SCHEDULED SACRIFICE / Time on Test: 29 / Cause(s) of Death:

Gross or Microscopic Finding
Disposition
Tissue: Site / Finding, Severity

MICROSCOPIC EXAMINATION

Disposition: NORMAL

BRAIN, AMYGDALOID BODY
BRAIN, BASAL NUCLEI/STRIATUM
BRAIN, CEREBELLUM
BRAIN, CEREBRAL CORTEX
BRAIN, HIPPOCAMPUS
BRAIN, HYPOTHALAMUS
BRAIN, MEDULLA OBLONGATA
BRAIN, MENINGES
BRAIN, MIDBRAIN
BRAIN, OLFACTORY BULB
BRAIN, PONS
BRAIN, THALAMUS
BRAIN, VENTRICULAR SYSTEM
BRAIN, WHITE MATTER
EYE
GANGLION, DORSAL ROOT, CERVICAL
GANGLION, DORSAL ROOT, THORACIC
KIDNEY
LUNG
LYMPH NODE, ILIAC
LYMPH NODE, MANDIBULAR
LYMPH NODE, MESENTERIC
MUSCLE, BICEPS FEMORIS
MUSCLE, GASTROCNEMIUS
NERVE ROOT, SPINAL, CERVICAL
NERVE ROOT, SPINAL, THORACIC
NERVE, OPTIC
NERVE, TIBIAL
PANCREAS
SPINAL CORD, CERVICAL
SPINAL CORD, LUMBAR
SPINAL CORD, THORACIC
SPLEEN
TESTIS

Appendix 10

Individual Animal Report

Study ID: 02776-0018: CRL 2954-001 / U of Tex Southwestern Med UTSW.Gray-002
Study Title: A SINGLE DOSE TOXICITY STUDY OF AAV9/SURF1 ADMINISTERED BY INTRATHECAL INJECTION IN RATS

Animal: 2021 (Male)

Sacrifice: DAY 29
Group: 2MD29 / AAV9/SURF1 LOW DOSE (0.28E12 VG)
Fate: SCHEDULED SACRIFICE / Time on Test: 29 / Cause(s) of Death:

Gross or Microscopic Finding
Disposition
Tissue: Site / Finding, Severity

THYMUS

Disposition: **ABNORMAL/FINDING**

GANGLION, DORSAL ROOT, LUMBAR
GANGLION, DORSAL ROOT, LUMBAR, GLIAL CELL: HYPERTROPHY/HYPERPLASIA, MINIMAL
HEART
HEART, MYOCARDIUM: DEGENERATION/NECROSIS, MINIMAL, MULTIFOCAL
HEART, MYOCARDIUM: INFILTRATE, MONONUCLEAR CELLS, MINIMAL, MULTIFOCAL
LIVER
LIVER: INFILTRATE, MIXED, MINIMAL
NERVE ROOT, SPINAL, LUMBAR
NERVE ROOT, SPINAL, LUMBAR, EPINEURIUM: INFILTRATE, MONONUCLEAR CELLS, MINIMAL, FOCAL
NERVE ROOT, SPINAL, LUMBAR, NERVE ROOT, DORSAL: DEGENERATION, NERVE FIBER, MINIMAL
NERVE, SCIATIC
NERVE, SCIATIC: DEGENERATION, NERVE FIBER, MINIMAL

Appendix 10

Individual Animal Report

Study ID: 02776-0018: CRL 2954-001 / U of Tex Southwestern Med UTSW.Gray-002
Study Title: A SINGLE DOSE TOXICITY STUDY OF AAV9/SURF1 ADMINISTERED BY INTRATHECAL INJECTION IN RATS

Animal: 2022 (Male)

Sacrifice: DAY 29
Group: 2MD29 / AAV9/SURF1 LOW DOSE (0.28E12 VG)
Fate: SCHEDULED SACRIFICE / Time on Test: 29 / Cause(s) of Death:

Gross or Microscopic Finding
Disposition
Tissue: Site / Finding, Severity

MICROSCOPIC EXAMINATION

Disposition: NORMAL

BRAIN, AMYGDALOID BODY
BRAIN, BASAL NUCLEI/STRIATUM
BRAIN, CEREBELLUM
BRAIN, CEREBRAL CORTEX
BRAIN, HIPPOCAMPUS
BRAIN, HYPOTHALAMUS
BRAIN, MEDULLA OBLONGATA
BRAIN, MENINGES
BRAIN, MIDBRAIN
BRAIN, OLFACTORY BULB
BRAIN, PONS
BRAIN, THALAMUS
BRAIN, VENTRICULAR SYSTEM
BRAIN, WHITE MATTER
EYE
GANGLION, DORSAL ROOT, CERVICAL
GANGLION, DORSAL ROOT, THORACIC
LUNG
LYMPH NODE, ILIAC
LYMPH NODE, MANDIBULAR
LYMPH NODE, MESENTERIC
MUSCLE, BICEPS FEMORIS
MUSCLE, GASTROCNEMIUS
NERVE ROOT, SPINAL, CERVICAL
NERVE ROOT, SPINAL, THORACIC
NERVE, OPTIC
NERVE, TIBIAL
PANCREAS
SPINAL CORD, CERVICAL
SPINAL CORD, LUMBAR
SPINAL CORD, THORACIC
SPLEEN
TESTIS
THYMUS

Appendix 10

Individual Animal Report

Study ID: 02776-0018: CRL 2954-001 / U of Tex Southwestern Med UTSW.Gray-002
Study Title: A SINGLE DOSE TOXICITY STUDY OF AAV9/SURF1 ADMINISTERED BY INTRATHECAL INJECTION IN RATS

Animal: 2022 (Male)

Sacrifice: DAY 29
Group: 2MD29 / AAV9/SURF1 LOW DOSE (0.28E12 VG)
Fate: SCHEDULED SACRIFICE / Time on Test: 29 / Cause(s) of Death:

Gross or Microscopic Finding
Disposition
Tissue: Site / Finding, Severity

Disposition: ABNORMAL/FINDING

GANGLION, DORSAL ROOT, LUMBAR
GANGLION, DORSAL ROOT, LUMBAR: INFILTRATE, MONONUCLEAR CELLS, MINIMAL, FOCAL
HEART
HEART, MYOCARDIUM: DEGENERATION/NECROSIS, MINIMAL, MULTIFOCAL
HEART, MYOCARDIUM: INFILTRATE, MONONUCLEAR CELLS, MINIMAL, MULTIFOCAL
KIDNEY
KIDNEY, TUBULAR: BASOPHILIA, MINIMAL
LIVER
LIVER: INFILTRATE, MIXED, MINIMAL
LIVER, PERIVASCULAR: INFILTRATE, MONONUCLEAR CELLS, MINIMAL
NERVE ROOT, SPINAL, LUMBAR
NERVE ROOT, SPINAL, LUMBAR, EPINEURIUM: INFILTRATE, MONONUCLEAR CELLS, MINIMAL, FOCAL
NERVE, SCIATIC
NERVE, SCIATIC: DEGENERATION, NERVE FIBER, MINIMAL

Appendix 10

Individual Animal Report

Study ID: 02776-0018: CRL 2954-001 / U of Tex Southwestern Med UTSW.Gray-002
Study Title: A SINGLE DOSE TOXICITY STUDY OF AAV9/SURF1 ADMINISTERED BY INTRATHECAL INJECTION IN RATS

Animal: 2023 (Male)

Sacrifice: DAY 29
Group: 2MD29 / AAV9/SURF1 LOW DOSE (0.28E12 VG)
Fate: SCHEDULED SACRIFICE / Time on Test: 29 / Cause(s) of Death:

Gross or Microscopic Finding
Disposition
Tissue: Site / Finding, Severity

MICROSCOPIC EXAMINATION

Disposition: NORMAL

BRAIN, AMYGDALOID BODY
BRAIN, BASAL NUCLEI/STRIATUM
BRAIN, CEREBELLUM
BRAIN, CEREBRAL CORTEX
BRAIN, HIPPOCAMPUS
BRAIN, HYPOTHALAMUS
BRAIN, MEDULLA OBLONGATA
BRAIN, OLFACTORY BULB
BRAIN, PONS
BRAIN, THALAMUS
BRAIN, VENTRICULAR SYSTEM
BRAIN, WHITE MATTER
EYE
GANGLION, DORSAL ROOT, CERVICAL
GANGLION, DORSAL ROOT, THORACIC
KIDNEY
LUNG
LYMPH NODE, ILIAC
LYMPH NODE, MANDIBULAR
LYMPH NODE, MESENTERIC
MUSCLE, BICEPS FEMORIS
MUSCLE, GASTROCNEMIUS
NERVE ROOT, SPINAL, CERVICAL
NERVE ROOT, SPINAL, THORACIC
NERVE, OPTIC
PANCREAS
SPINAL CORD, CERVICAL
SPINAL CORD, LUMBAR
SPLEEN
TESTIS
THYMUS

Disposition: ABNORMAL/FINDING

BRAIN, MENINGES

Appendix 10

Individual Animal Report

Study ID: 02776-0018: CRL 2954-001 / U of Tex Southwestern Med UTSW.Gray-002
Study Title: A SINGLE DOSE TOXICITY STUDY OF AAV9/SURF1 ADMINISTERED BY INTRATHECAL INJECTION IN RATS

Animal: 2023 (Male)

Sacrifice: DAY 29
Group: 2MD29 / AAV9/SURF1 LOW DOSE (0.28E12 VG)
Fate: SCHEDULED SACRIFICE / Time on Test: 29 / Cause(s) of Death:

Gross or Microscopic Finding
Disposition
Tissue: Site / Finding, Severity

BRAIN, MENINGES: INFILTRATE, MONONUCLEAR CELLS, MINIMAL, MULTIFOCAL
BRAIN, MIDBRAIN
BRAIN, MIDBRAIN, PINEAL GLAND: INFILTRATE, MONONUCLEAR CELLS, MINIMAL, FOCAL
GANGLION, DORSAL ROOT, LUMBAR
GANGLION, DORSAL ROOT, LUMBAR, GLIAL CELL: HYPERTROPHY/HYPERPLASIA, MINIMAL
HEART
HEART, MYOCARDIUM: DEGENERATION/NECROSIS, MILD
HEART, MYOCARDIUM: FIBROSIS, MINIMAL
HEART, MYOCARDIUM: INFILTRATE, MONONUCLEAR CELLS, MILD
LIVER
LIVER: INFILTRATE, MIXED, MINIMAL
NERVE ROOT, SPINAL, LUMBAR
NERVE ROOT, SPINAL, LUMBAR, EPINEURIUM: INFILTRATE, MONONUCLEAR CELLS, MINIMAL, FOCAL
NERVE ROOT, SPINAL, LUMBAR, NERVE ROOT, DORSAL: DEGENERATION, NERVE FIBER, MINIMAL
NERVE, SCIATIC
NERVE, SCIATIC: DEGENERATION, NERVE FIBER, MINIMAL
NERVE, TIBIAL
NERVE, TIBIAL: DEGENERATION, NERVE FIBER, MINIMAL
SPINAL CORD, THORACIC
SPINAL CORD, THORACIC, WHITE MATTER: DEGENERATION, NERVE FIBER, MINIMAL
COMMENT(S): SEEN IN LONG SECTION AND EXACT LOCATION COULD NOT BE DETERMINED

Appendix 10

Individual Animal Report

Study ID: 02776-0018: CRL 2954-001 / U of Tex Southwestern Med UTSW.Gray-002
Study Title: A SINGLE DOSE TOXICITY STUDY OF AAV9/SURF1 ADMINISTERED BY INTRATHECAL INJECTION IN RATS

Animal: 2024 (Male)

Sacrifice: DAY 29
Group: 2MD29 / AAV9/SURF1 LOW DOSE (0.28E12 VG)
Fate: SCHEDULED SACRIFICE / Time on Test: 29 / Cause(s) of Death:

Gross or Microscopic Finding
Disposition
Tissue: Site / Finding, Severity

MICROSCOPIC EXAMINATION

Disposition: NORMAL

BRAIN, AMYGDALOID BODY
BRAIN, BASAL NUCLEI/STRIATUM
BRAIN, CEREBELLUM
BRAIN, CEREBRAL CORTEX
BRAIN, HIPPOCAMPUS
BRAIN, HYPOTHALAMUS
BRAIN, MEDULLA OBLONGATA
BRAIN, MIDBRAIN
BRAIN, OLFACTORY BULB
BRAIN, PONS
BRAIN, THALAMUS
BRAIN, VENTRICULAR SYSTEM
BRAIN, WHITE MATTER
EYE
GANGLION, DORSAL ROOT, CERVICAL
GANGLION, DORSAL ROOT, LUMBAR
GANGLION, DORSAL ROOT, THORACIC
LUNG
LYMPH NODE, ILIAC
LYMPH NODE, MANDIBULAR
LYMPH NODE, MESENTERIC
MUSCLE, BICEPS FEMORIS
MUSCLE, GASTROCNEMIUS
NERVE ROOT, SPINAL, CERVICAL
NERVE ROOT, SPINAL, LUMBAR
NERVE ROOT, SPINAL, THORACIC
NERVE, OPTIC
NERVE, SCIATIC
NERVE, TIBIAL
SPINAL CORD, LUMBAR
SPINAL CORD, THORACIC
SPLEEN
TESTIS
THYMUS

Appendix 10

Individual Animal Report

Study ID: 02776-0018: CRL 2954-001 / U of Tex Southwestern Med UTSW.Gray-002
Study Title: A SINGLE DOSE TOXICITY STUDY OF AAV9/SURF1 ADMINISTERED BY INTRATHECAL INJECTION IN RATS

Animal: 2024 (Male)

Sacrifice: DAY 29
Group: 2MD29 / AAV9/SURF1 LOW DOSE (0.28E12 VG)
Fate: SCHEDULED SACRIFICE / Time on Test: 29 / Cause(s) of Death:

Gross or Microscopic Finding
Disposition
Tissue: Site / Finding, Severity

Disposition: ABNORMAL/FINDING

BRAIN, MENINGES
BRAIN, MENINGES: INFILTRATE, MONONUCLEAR CELLS, MINIMAL, FOCAL
HEART
HEART, MYOCARDIUM: DEGENERATION/NECROSIS, MARKED, MULTIFOCAL
HEART, MYOCARDIUM: FIBROSIS, MILD, MULTIFOCAL
HEART, MYOCARDIUM: INFILTRATE, MONONUCLEAR CELLS, MARKED, MULTIFOCAL
KIDNEY
KIDNEY: CHRONIC PROGRESSIVE NEPHROPATHY, MINIMAL
LIVER
LIVER: INFILTRATE, MIXED, MINIMAL
LIVER, HEPATOCYTE: VACUOLATION, MILD, MULTIFOCAL
COMMENT(S): SOME HEPATOCYTES ALSO HAVE BRIGHT EOSINOPHILIC INTRACYTOPLASMIC ‘INCLUSIONS’
PANCREAS
PANCREAS: FIBROSIS, MINIMAL, MULTIFOCAL
COMMENT(S): AFFECTING ISLETS OF LANGERHANS; SOME AFFECTED ISLETS ALSO HAVE CHRONIC ACTIVE INFLAMMATION, HEMORRHAGE AND HEMOSIDERIN PIGMENT
SPINAL CORD, CERVICAL
SPINAL CORD, CERVICAL, GLIAL CELL: INCREASED CELLULARITY, MINIMAL, FOCAL
COMMENT(S): IN DORSAL WHITE MATTER AT JUNCTION WITH SPINAL NERVE

Appendix 10

Individual Animal Report

Study ID: 02776-0018: CRL 2954-001 / U of Tex Southwestern Med UTSW.Gray-002
Study Title: A SINGLE DOSE TOXICITY STUDY OF AAV9/SURF1 ADMINISTERED BY INTRATHECAL INJECTION IN RATS

Animal: 2025 (Male)

Sacrifice: DAY 29
Group: 2MD29 / AAV9/SURF1 LOW DOSE (0.28E12 VG)
Fate: SCHEDULED SACRIFICE / Time on Test: 29 / Cause(s) of Death:

Gross or Microscopic Finding
Disposition
Tissue: Site / Finding, Severity

MICROSCOPIC EXAMINATION

Disposition: NORMAL

BRAIN, AMYGDALOID BODY
BRAIN, BASAL NUCLEI/STRIATUM
BRAIN, CEREBELLUM
BRAIN, CEREBRAL CORTEX
BRAIN, HIPPOCAMPUS
BRAIN, HYPOTHALAMUS
BRAIN, MEDULLA OBLONGATA
BRAIN, MENINGES
BRAIN, MIDBRAIN
BRAIN, OLFACTORY BULB
BRAIN, PONS
BRAIN, THALAMUS
BRAIN, VENTRICULAR SYSTEM
BRAIN, WHITE MATTER
EYE
GANGLION, DORSAL ROOT, CERVICAL
GANGLION, DORSAL ROOT, THORACIC
LYMPH NODE, ILIAC
LYMPH NODE, MANDIBULAR
LYMPH NODE, MESENTERIC
MUSCLE, BICEPS FEMORIS
MUSCLE, GASTROCNEMIUS
NERVE ROOT, SPINAL, CERVICAL
NERVE ROOT, SPINAL, THORACIC
NERVE, OPTIC
PANCREAS
SPINAL CORD, THORACIC
SPLEEN
TESTIS
THYMUS

Disposition: ABNORMAL/FINDING

GANGLION, DORSAL ROOT, LUMBAR
GANGLION, DORSAL ROOT, LUMBAR: INFILTRATE, MONONUCLEAR CELLS, MINIMAL, FOCAL

Appendix 10

Individual Animal Report

Study ID: 02776-0018: CRL 2954-001 / U of Tex Southwestern Med UTSW.Gray-002
Study Title: A SINGLE DOSE TOXICITY STUDY OF AAV9/SURF1 ADMINISTERED BY INTRATHECAL INJECTION IN RATS

Animal: 2025 (Male)

Sacrifice: DAY 29
Group: 2MD29 / AAV9/SURF1 LOW DOSE (0.28E12 VG)
Fate: SCHEDULED SACRIFICE / Time on Test: 29 / Cause(s) of Death:

Gross or Microscopic Finding
Disposition
Tissue: Site / Finding, Severity

GANGLION, DORSAL ROOT, LUMBAR, NEURON: DEGENERATION/NECROSIS, MINIMAL, SINGLE
HEART
HEART, MYOCARDIUM: DEGENERATION/NECROSIS, MINIMAL, MULTIFOCAL
HEART, MYOCARDIUM: INFILTRATE, MONONUCLEAR CELLS, MINIMAL, MULTIFOCAL
KIDNEY
KIDNEY: CHRONIC PROGRESSIVE NEPHROPATHY, MINIMAL
LIVER
LIVER: INFILTRATE, MIXED, MINIMAL
LUNG
LUNG, PERIVASCULAR: INFILTRATE, EOSINOPHILS, MINIMAL, FOCAL
NERVE ROOT, SPINAL, LUMBAR
NERVE ROOT, SPINAL, LUMBAR, EPINEURIUM: INFILTRATE, MONONUCLEAR CELLS, MINIMAL, FOCAL
NERVE, SCIATIC
NERVE, SCIATIC: DEGENERATION, NERVE FIBER, MINIMAL
NERVE, TIBIAL
NERVE, TIBIAL: DEGENERATION, NERVE FIBER, MILD
SPINAL CORD, CERVICAL
SPINAL CORD, CERVICAL, WHITE MATTER: DEGENERATION, NERVE FIBER, MINIMAL, DORSAL
SPINAL CORD, LUMBAR
SPINAL CORD, LUMBAR, NERVE ROOT, SPINAL: DEGENERATION, NERVE FIBER, MINIMAL

Appendix 10

Individual Animal Report

Study ID: 02776-0018: CRL 2954-001 / U of Tex Southwestern Med UTSW.Gray-002
Study Title: A SINGLE DOSE TOXICITY STUDY OF AAV9/SURF1 ADMINISTERED BY INTRATHECAL INJECTION IN RATS

Animal: 3021 (Male)

Sacrifice: DAY 29
Group: 3MD29 / AAV9/SURF1 MID DOSE (0.83E12 VG)
Fate: SCHEDULED SACRIFICE / Time on Test: 29 / Cause(s) of Death:

Gross or Microscopic Finding
Disposition
Tissue: Site / Finding, Severity

MICROSCOPIC EXAMINATION

Disposition: NORMAL

BRAIN, AMYGDALOID BODY
BRAIN, BASAL NUCLEI/STRIATUM
BRAIN, CEREBELLUM
BRAIN, CEREBRAL CORTEX
BRAIN, HIPPOCAMPUS
BRAIN, HYPOTHALAMUS
BRAIN, MEDULLA OBLONGATA
BRAIN, MENINGES
BRAIN, MIDBRAIN
BRAIN, OLFACTORY BULB
BRAIN, PONS
BRAIN, THALAMUS
BRAIN, VENTRICULAR SYSTEM
BRAIN, WHITE MATTER
EYE
GANGLION, DORSAL ROOT, THORACIC
KIDNEY
LYMPH NODE, ILIAC
LYMPH NODE, MANDIBULAR
LYMPH NODE, MESENTERIC
MUSCLE, BICEPS FEMORIS
MUSCLE, GASTROCNEMIUS
NERVE ROOT, SPINAL, CERVICAL
NERVE ROOT, SPINAL, THORACIC
NERVE, OPTIC
PANCREAS
SPINAL CORD, CERVICAL
SPLEEN
TESTIS
THYMUS

Disposition: ABNORMAL/FINDING

GANGLION, DORSAL ROOT, CERVICAL
GANGLION, DORSAL ROOT, CERVICAL: INFILTRATE, MONONUCLEAR CELLS, MINIMAL, FOCAL

Appendix 10

Individual Animal Report

Study ID: 02776-0018: CRL 2954-001 / U of Tex Southwestern Med UTSW.Gray-002
Study Title: A SINGLE DOSE TOXICITY STUDY OF AAV9/SURF1 ADMINISTERED BY INTRATHECAL INJECTION IN RATS

Animal: 3021 (Male)

Sacrifice: DAY 29
Group: 3MD29 / AAV9/SURF1 MID DOSE (0.83E12 VG)
Fate: SCHEDULED SACRIFICE / Time on Test: 29 / Cause(s) of Death:

Gross or Microscopic Finding
Disposition
Tissue: Site / Finding, Severity

GANGLION, DORSAL ROOT, CERVICAL, GLIAL CELL: HYPERTROPHY/HYPERPLASIA, MINIMAL
GANGLION, DORSAL ROOT, LUMBAR
GANGLION, DORSAL ROOT, LUMBAR: INFILTRATE, MONONUCLEAR CELLS, MINIMAL, FOCAL
GANGLION, DORSAL ROOT, LUMBAR, GLIAL CELL: HYPERTROPHY/HYPERPLASIA, MINIMAL
HEART
HEART, MYOCARDIUM: DEGENERATION/NECROSIS, MODERATE, MULTIFOCAL
HEART, MYOCARDIUM: INFILTRATE, MONONUCLEAR CELLS, MODERATE, MULTIFOCAL
LIVER
LIVER: INFILTRATE, MIXED, MINIMAL
LUNG
LUNG: INFLAMMATION, MINIMAL, SUBACUTE, FOCAL
NERVE ROOT, SPINAL, LUMBAR
NERVE ROOT, SPINAL, LUMBAR, EPINEURIUM: INFILTRATE, MONONUCLEAR CELLS, MINIMAL, FOCAL
NERVE, SCIATIC
NERVE, SCIATIC: DEGENERATION, NERVE FIBER, MODERATE
NERVE, TIBIAL
NERVE, TIBIAL: DEGENERATION, NERVE FIBER, MODERATE
NERVE, TIBIAL, SCHWANN CELL: HYPERTROPHY/HYPERPLASIA, MINIMAL
SPINAL CORD, LUMBAR
SPINAL CORD, LUMBAR, WHITE MATTER: DEGENERATION, NERVE FIBER, MINIMAL, DORSAL
SPINAL CORD, THORACIC
SPINAL CORD, THORACIC, WHITE MATTER: DEGENERATION, NERVE FIBER, MINIMAL, DORSAL

Appendix 10

Individual Animal Report

Study ID: 02776-0018: CRL 2954-001 / U of Tex Southwestern Med UTSW.Gray-002
Study Title: A SINGLE DOSE TOXICITY STUDY OF AAV9/SURF1 ADMINISTERED BY INTRATHECAL INJECTION IN RATS

Animal: 3022 (Male)

Sacrifice: DAY 29
Group: 3MD29 / AAV9/SURF1 MID DOSE (0.83E12 VG)
Fate: SCHEDULED SACRIFICE / Time on Test: 29 / Cause(s) of Death:

Gross or Microscopic Finding
Disposition
Tissue: Site / Finding, Severity

MICROSCOPIC EXAMINATION

Disposition: NORMAL

BRAIN, AMYGDALOID BODY
BRAIN, BASAL NUCLEI/STRIATUM
BRAIN, CEREBELLUM
BRAIN, CEREBRAL CORTEX
BRAIN, HIPPOCAMPUS
BRAIN, HYPOTHALAMUS
BRAIN, MEDULLA OBLONGATA
BRAIN, MENINGES
BRAIN, MIDBRAIN
BRAIN, OLFACTORY BULB
BRAIN, PONS
BRAIN, THALAMUS
BRAIN, VENTRICULAR SYSTEM
BRAIN, WHITE MATTER
EYE
GANGLION, DORSAL ROOT, THORACIC
LUNG
LYMPH NODE, ILIAC
LYMPH NODE, MANDIBULAR
LYMPH NODE, MESENTERIC
MUSCLE, BICEPS FEMORIS
MUSCLE, GASTROCNEMIUS
NERVE ROOT, SPINAL, CERVICAL
NERVE ROOT, SPINAL, THORACIC
NERVE, OPTIC
PANCREAS
SPINAL CORD, CERVICAL
SPLEEN
TESTIS
THYMUS

Disposition: ABNORMAL/FINDING

GANGLION, DORSAL ROOT, CERVICAL
GANGLION, DORSAL ROOT, CERVICAL, GLIAL CELL: HYPERTROPHY/HYPERPLASIA, MINIMAL

Appendix 10

Individual Animal Report

Study ID: 02776-0018: CRL 2954-001 / U of Tex Southwestern Med UTSW.Gray-002
Study Title: A SINGLE DOSE TOXICITY STUDY OF AAV9/SURF1 ADMINISTERED BY INTRATHECAL INJECTION IN RATS

Animal: 3022 (Male)

Sacrifice: DAY 29
Group: 3MD29 / AAV9/SURF1 MID DOSE (0.83E12 VG)
Fate: SCHEDULED SACRIFICE / Time on Test: 29 / Cause(s) of Death:

Gross or Microscopic Finding
Disposition
Tissue: Site / Finding, Severity

GANGLION, DORSAL ROOT, LUMBAR
GANGLION, DORSAL ROOT, LUMBAR: INFILTRATE, MONONUCLEAR CELLS, MINIMAL, MULTIFOCAL
GANGLION, DORSAL ROOT, LUMBAR, GLIAL CELL: HYPERTROPHY/HYPERPLASIA, MINIMAL
HEART
HEART, MYOCARDIUM: DEGENERATION/NECROSIS, MINIMAL, MULTIFOCAL
HEART, MYOCARDIUM: INFILTRATE, MONONUCLEAR CELLS, MINIMAL, MULTIFOCAL
KIDNEY
KIDNEY: CHRONIC PROGRESSIVE NEPHROPATHY, MINIMAL
LIVER
LIVER: INFILTRATE, MIXED, MILD
NERVE ROOT, SPINAL, LUMBAR
NERVE ROOT, SPINAL, LUMBAR, EPINEURIUM: INFILTRATE, MONONUCLEAR CELLS, MINIMAL, FOCAL
NERVE ROOT, SPINAL, LUMBAR, NERVE ROOT, DORSAL: DEGENERATION, NERVE FIBER, MINIMAL
NERVE, SCIATIC
NERVE, SCIATIC: DEGENERATION, NERVE FIBER, MILD
NERVE, TIBIAL
NERVE, TIBIAL: DEGENERATION, NERVE FIBER, MODERATE
NERVE, TIBIAL, SCHWANN CELL: HYPERTROPHY/HYPERPLASIA, MINIMAL
SPINAL CORD, LUMBAR
SPINAL CORD, LUMBAR, WHITE MATTER: DEGENERATION, NERVE FIBER, MILD, DORSAL
COMMENT(S): MILD DEGENERATION PRESENT WITHIN THE DORSAL WHITE MATTER TRACTS AND MINIMAL CHANGES PRESENT WITHIN THE LATERAL WHITE MATTER TRACTS
SPINAL CORD, THORACIC
SPINAL CORD, THORACIC, WHITE MATTER: DEGENERATION, NERVE FIBER, MINIMAL, DORSAL

Appendix 10

Individual Animal Report

Study ID: 02776-0018: CRL 2954-001 / U of Tex Southwestern Med UTSW.Gray-002
Study Title: A SINGLE DOSE TOXICITY STUDY OF AAV9/SURF1 ADMINISTERED BY INTRATHECAL INJECTION IN RATS

Animal: 3023 (Male)

Sacrifice: DAY 29
Group: 3MD29 / AAV9/SURF1 MID DOSE (0.83E12 VG)
Fate: SCHEDULED SACRIFICE / Time on Test: 29 / Cause(s) of Death:

Gross or Microscopic Finding
Disposition
Tissue: Site / Finding, Severity

MICROSCOPIC EXAMINATION

Disposition: NORMAL

BRAIN, AMYGDALOID BODY
BRAIN, BASAL NUCLEI/STRIATUM
BRAIN, CEREBELLUM
BRAIN, CEREBRAL CORTEX
BRAIN, HIPPOCAMPUS
BRAIN, HYPOTHALAMUS
BRAIN, MEDULLA OBLONGATA
BRAIN, MENINGES
BRAIN, OLFACTORY BULB
BRAIN, PONS
BRAIN, THALAMUS
BRAIN, VENTRICULAR SYSTEM
BRAIN, WHITE MATTER
EYE
KIDNEY
LUNG
LYMPH NODE, ILIAC
LYMPH NODE, MANDIBULAR
LYMPH NODE, MESENTERIC
MUSCLE, BICEPS FEMORIS
MUSCLE, GASTROCNEMIUS
NERVE ROOT, SPINAL, CERVICAL
NERVE, OPTIC
NERVE, TIBIAL
PANCREAS
SPINAL CORD, CERVICAL
SPLEEN
TESTIS
THYMUS

Disposition: ABNORMAL/FINDING

BRAIN, MIDBRAIN
BRAIN, MIDBRAIN, PINEAL GLAND: INFILTRATE, MONONUCLEAR CELLS, MINIMAL, FOCAL
GANGLION, DORSAL ROOT, CERVICAL

Appendix 10

Individual Animal Report

Study ID: 02776-0018: CRL 2954-001 / U of Tex Southwestern Med UTSW.Gray-002
Study Title: A SINGLE DOSE TOXICITY STUDY OF AAV9/SURF1 ADMINISTERED BY INTRATHECAL INJECTION IN RATS

Animal: 3023 (Male)

Sacrifice: DAY 29
Group: 3MD29 / AAV9/SURF1 MID DOSE (0.83E12 VG)
Fate: SCHEDULED SACRIFICE / Time on Test: 29 / Cause(s) of Death:

Gross or Microscopic Finding
Disposition
Tissue: Site / Finding, Severity

GANGLION, DORSAL ROOT, CERVICAL, GLIAL CELL: HYPERTROPHY/HYPERPLASIA, MINIMAL
GANGLION, DORSAL ROOT, LUMBAR
GANGLION, DORSAL ROOT, LUMBAR, GLIAL CELL: HYPERTROPHY/HYPERPLASIA, MINIMAL
HEART
HEART, MYOCARDIUM: DEGENERATION/NECROSIS, MARKED
HEART, MYOCARDIUM: FIBROSIS, MINIMAL
HEART, MYOCARDIUM: INFILTRATE, MONONUCLEAR CELLS, MARKED
LIVER
LIVER: INFILTRATE, MIXED, MINIMAL
NERVE ROOT, SPINAL, LUMBAR
NERVE ROOT, SPINAL, LUMBAR, NERVE ROOT, DORSAL: DEGENERATION, NERVE FIBER, MINIMAL
NERVE ROOT, SPINAL, LUMBAR, NERVE ROOT, VENTRAL: DEGENERATION, NERVE FIBER, MINIMAL
NERVE, SCIATIC
NERVE, SCIATIC: DEGENERATION, NERVE FIBER, MINIMAL
SPINAL CORD, LUMBAR
SPINAL CORD, LUMBAR, NERVE ROOT, SPINAL: DEGENERATION, NERVE FIBER, MILD
SPINAL CORD, LUMBAR, WHITE MATTER: DEGENERATION, NERVE FIBER, MINIMAL, DORSAL
SPINAL CORD, THORACIC
SPINAL CORD, THORACIC, GRAY MATTER: INFLAMMATION, MINIMAL, LOCALLY EXTENSIVE
COMMENT(S): TRACT-LIKE LESION ALONG GRAY MATTER/LATERAL WHITE MATTER TRACT JUNCTION
SPINAL CORD, THORACIC, MENINGES: INFILTRATE, MONONUCLEAR CELLS, MINIMAL, LOCALLY EXTENSIVE
SPINAL CORD, THORACIC, WHITE MATTER: DEGENERATION, NERVE FIBER, MILD, LATERAL

Disposition: UNABLE TO OBTAIN SPECIMEN
GANGLION, DORSAL ROOT, THORACIC
NERVE ROOT, SPINAL, THORACIC

Appendix 10

Individual Animal Report

Study ID: 02776-0018: CRL 2954-001 / U of Tex Southwestern Med UTSW.Gray-002
Study Title: A SINGLE DOSE TOXICITY STUDY OF AAV9/SURF1 ADMINISTERED BY INTRATHECAL INJECTION IN RATS

Animal: 3024 (Male)

Sacrifice: DAY 29
Group: 3MD29 / AAV9/SURF1 MID DOSE (0.83E12 VG)
Fate: SCHEDULED SACRIFICE / Time on Test: 29 / Cause(s) of Death:

Gross or Microscopic Finding
Disposition
Tissue: Site / Finding, Severity

MICROSCOPIC EXAMINATION

Disposition: NORMAL

BRAIN, AMYGDALOID BODY
BRAIN, BASAL NUCLEI/STRIATUM
BRAIN, CEREBELLUM
BRAIN, CEREBRAL CORTEX
BRAIN, HIPPOCAMPUS
BRAIN, HYPOTHALAMUS
BRAIN, MEDULLA OBLONGATA
BRAIN, MENINGES
BRAIN, MIDBRAIN
BRAIN, OLFACTORY BULB
BRAIN, PONS
BRAIN, THALAMUS
BRAIN, VENTRICULAR SYSTEM
BRAIN, WHITE MATTER
EYE
LUNG
LYMPH NODE, ILIAC
LYMPH NODE, MANDIBULAR
LYMPH NODE, MESENTERIC
MUSCLE, BICEPS FEMORIS
MUSCLE, GASTROCNEMIUS
NERVE ROOT, SPINAL, CERVICAL
NERVE ROOT, SPINAL, THORACIC
NERVE, OPTIC
NERVE, TIBIAL
SPINAL CORD, CERVICAL
SPINAL CORD, LUMBAR
SPINAL CORD, THORACIC
SPLEEN
TESTIS
THYMUS

Disposition: ABNORMAL/FINDING

GANGLION, DORSAL ROOT, CERVICAL

Appendix 10

Individual Animal Report

Study ID: 02776-0018: CRL 2954-001 / U of Tex Southwestern Med UTSW.Gray-002
Study Title: A SINGLE DOSE TOXICITY STUDY OF AAV9/SURF1 ADMINISTERED BY INTRATHECAL INJECTION IN RATS

Animal: 3024 (Male)

Sacrifice: DAY 29
Group: 3MD29 / AAV9/SURF1 MID DOSE (0.83E12 VG)
Fate: SCHEDULED SACRIFICE / Time on Test: 29 / Cause(s) of Death:

Gross or Microscopic Finding
Disposition
Tissue: Site / Finding, Severity

GANGLION, DORSAL ROOT, CERVICAL, GLIAL CELL: HYPERTROPHY/HYPERPLASIA, MINIMAL
GANGLION, DORSAL ROOT, LUMBAR
GANGLION, DORSAL ROOT, LUMBAR: INFILTRATE, MONONUCLEAR CELLS, MINIMAL
GANGLION, DORSAL ROOT, LUMBAR, GLIAL CELL: HYPERTROPHY/HYPERPLASIA, MINIMAL
GANGLION, DORSAL ROOT, THORACIC
GANGLION, DORSAL ROOT, THORACIC, GLIAL CELL: HYPERTROPHY/HYPERPLASIA, MINIMAL
HEART
HEART, MYOCARDIUM: DEGENERATION/NECROSIS, MILD
HEART, MYOCARDIUM: FIBROSIS, MINIMAL
HEART, MYOCARDIUM: INFILTRATE, MONONUCLEAR CELLS, MILD
KIDNEY
KIDNEY: CHRONIC PROGRESSIVE NEPHROPATHY, MINIMAL
LIVER
LIVER: INFILTRATE, MIXED, MINIMAL
NERVE ROOT, SPINAL, LUMBAR
NERVE ROOT, SPINAL, LUMBAR, EPINEURIUM: INFILTRATE, MONONUCLEAR CELLS, MINIMAL, FOCAL
NERVE ROOT, SPINAL, LUMBAR, NERVE ROOT, VENTRAL: DEGENERATION, NERVE FIBER, MINIMAL
NERVE, SCIATIC
NERVE, SCIATIC: DEGENERATION, NERVE FIBER, MINIMAL
PANCREAS
PANCREAS: ATROPHY, MINIMAL, FOCAL

Appendix 10

Individual Animal Report

Study ID: 02776-0018: CRL 2954-001 / U of Tex Southwestern Med UTSW.Gray-002
Study Title: A SINGLE DOSE TOXICITY STUDY OF AAV9/SURF1 ADMINISTERED BY INTRATHECAL INJECTION IN RATS

Animal: 3025 (Male)

Sacrifice: DAY 29
Group: 3MD29 / AAV9/SURF1 MID DOSE (0.83E12 VG)
Fate: SCHEDULED SACRIFICE / Time on Test: 29 / Cause(s) of Death:

Gross or Microscopic Finding
Disposition
Tissue: Site / Finding, Severity

MICROSCOPIC EXAMINATION

Disposition: **NORMAL**

BRAIN, AMYGDALOID BODY
BRAIN, BASAL NUCLEI/STRIATUM
BRAIN, CEREBELLUM
BRAIN, CEREBRAL CORTEX
BRAIN, HIPPOCAMPUS
BRAIN, HYPOTHALAMUS
BRAIN, MEDULLA OBLONGATA
BRAIN, MENINGES
BRAIN, MIDBRAIN
BRAIN, OLFACTORY BULB
BRAIN, PONS
BRAIN, THALAMUS
BRAIN, VENTRICULAR SYSTEM
BRAIN, WHITE MATTER
EYE
GANGLION, DORSAL ROOT, CERVICAL
GANGLION, DORSAL ROOT, THORACIC
LYMPH NODE, ILIAC
LYMPH NODE, MANDIBULAR
LYMPH NODE, MESENTERIC
MUSCLE, BICEPS FEMORIS
MUSCLE, GASTROCNEMIUS
NERVE ROOT, SPINAL, CERVICAL
NERVE ROOT, SPINAL, LUMBAR
NERVE ROOT, SPINAL, THORACIC
NERVE, OPTIC
NERVE, SCIATIC
NERVE, TIBIAL
PANCREAS
SPINAL CORD, CERVICAL
SPINAL CORD, LUMBAR
SPINAL CORD, THORACIC
SPLEEN
TESTIS

Appendix 10

Individual Animal Report

Study ID: 02776-0018: CRL 2954-001 / U of Tex Southwestern Med UTSW.Gray-002
Study Title: A SINGLE DOSE TOXICITY STUDY OF AAV9/SURF1 ADMINISTERED BY INTRATHECAL INJECTION IN RATS

Animal: 3025 (Male)

Sacrifice: DAY 29
Group: 3MD29 / AAV9/SURF1 MID DOSE (0.83E12 VG)
Fate: SCHEDULED SACRIFICE / Time on Test: 29 / Cause(s) of Death:

Gross or Microscopic Finding
Disposition
Tissue: Site / Finding, Severity

THYMUS

Disposition: **ABNORMAL/FINDING**

GANGLION, DORSAL ROOT, LUMBAR
GANGLION, DORSAL ROOT, LUMBAR: INFILTRATE, MONONUCLEAR CELLS, MINIMAL, FOCAL
GANGLION, DORSAL ROOT, LUMBAR, GLIAL CELL: HYPERTROPHY/HYPERPLASIA, MINIMAL
HEART
HEART, MYOCARDIUM: DEGENERATION/NECROSIS, MILD, MULTIFOCAL
HEART, MYOCARDIUM: INFILTRATE, MONONUCLEAR CELLS, MILD, MULTIFOCAL
KIDNEY
KIDNEY, TUBULAR: CYST, MINIMAL
LIVER
LIVER: INFILTRATE, MIXED, MINIMAL
LUNG
LUNG, ALVEOLUS: INFILTRATE, MACROPHAGES, MINIMAL, FOCAL

Appendix 10

Individual Animal Report

Study ID: 02776-0018: CRL 2954-001 / U of Tex Southwestern Med UTSW.Gray-002
Study Title: A SINGLE DOSE TOXICITY STUDY OF AAV9/SURF1 ADMINISTERED BY INTRATHECAL INJECTION IN RATS

Animal: 4023 (Male)

Sacrifice: DAY 29
Group: 4MD29 / AAV9/SURF1 HIGH DOSE (2.49E12 VG)
Fate: SCHEDULED SACRIFICE / Time on Test: 29 / Cause(s) of Death:

Gross or Microscopic Finding
Disposition
Tissue: Site / Finding, Severity

MICROSCOPIC EXAMINATION

Disposition: NORMAL

BRAIN, AMYGDALOID BODY
BRAIN, BASAL NUCLEI/STRIATUM
BRAIN, CEREBELLUM
BRAIN, CEREBRAL CORTEX
BRAIN, HIPPOCAMPUS
BRAIN, HYPOTHALAMUS
BRAIN, MEDULLA OBLONGATA
BRAIN, MENINGES
BRAIN, MIDBRAIN
BRAIN, OLFACTORY BULB
BRAIN, PONS
BRAIN, THALAMUS
BRAIN, VENTRICULAR SYSTEM
BRAIN, WHITE MATTER
EYE
GANGLION, DORSAL ROOT, CERVICAL
LUNG
LYMPH NODE, ILIAC
LYMPH NODE, MANDIBULAR
LYMPH NODE, MESENTERIC
MUSCLE, BICEPS FEMORIS
MUSCLE, GASTROCNEMIUS
NERVE ROOT, SPINAL, CERVICAL
NERVE ROOT, SPINAL, THORACIC
NERVE, OPTIC
PANCREAS
SPINAL CORD, CERVICAL
SPINAL CORD, THORACIC
SPLEEN
TESTIS
THYMUS

Disposition: ABNORMAL/FINDING

GANGLION, DORSAL ROOT, LUMBAR

Appendix 10

Individual Animal Report

Study ID: 02776-0018: CRL 2954-001 / U of Tex Southwestern Med UTSW.Gray-002
Study Title: A SINGLE DOSE TOXICITY STUDY OF AAV9/SURF1 ADMINISTERED BY INTRATHECAL INJECTION IN RATS

Animal: 4023 (Male)

Sacrifice: DAY 29
Group: 4MD29 / AAV9/SURF1 HIGH DOSE (2.49E12 VG)
Fate: SCHEDULED SACRIFICE / Time on Test: 29 / Cause(s) of Death:

Gross or Microscopic Finding
Disposition
Tissue: Site / Finding, Severity

GANGLION, DORSAL ROOT, LUMBAR: INFILTRATE, MONONUCLEAR CELLS, MINIMAL, MULTIFOCAL
GANGLION, DORSAL ROOT, LUMBAR, GLIAL CELL: HYPERTROPHY/HYPERPLASIA, MINIMAL
GANGLION, DORSAL ROOT, THORACIC
GANGLION, DORSAL ROOT, THORACIC, GLIAL CELL: HYPERTROPHY/HYPERPLASIA, MINIMAL
HEART
HEART, MYOCARDIUM: DEGENERATION/NECROSIS, MINIMAL, MULTIFOCAL
HEART, MYOCARDIUM: INFILTRATE, MONONUCLEAR CELLS, MINIMAL, MULTIFOCAL
KIDNEY
KIDNEY, TUBULAR: BASOPHILIA, MINIMAL
LIVER
LIVER: INFILTRATE, MIXED, MILD
LIVER, HEPATOCYTE: NECROSIS, SINGLE CELL, MINIMAL
NERVE ROOT, SPINAL, LUMBAR
NERVE ROOT, SPINAL, LUMBAR, NERVE ROOT, DORSAL: DEGENERATION, NERVE FIBER, MINIMAL
NERVE, SCIATIC
NERVE, SCIATIC: DEGENERATION, NERVE FIBER, MODERATE
NERVE, SCIATIC, SCHWANN CELL: HYPERTROPHY/HYPERPLASIA, MILD
NERVE, TIBIAL
NERVE, TIBIAL: DEGENERATION, NERVE FIBER, MODERATE
NERVE, TIBIAL, SCHWANN CELL: HYPERTROPHY/HYPERPLASIA, MILD
SPINAL CORD, LUMBAR
SPINAL CORD, LUMBAR, WHITE MATTER: DEGENERATION, NERVE FIBER, MINIMAL, DORSAL

Appendix 10

Individual Animal Report

Study ID: 02776-0018: CRL 2954-001 / U of Tex Southwestern Med UTSW.Gray-002
Study Title: A SINGLE DOSE TOXICITY STUDY OF AAV9/SURF1 ADMINISTERED BY INTRATHECAL INJECTION IN RATS

Animal: 4024 (Male)

Sacrifice: DAY 29
Group: 4MD29 / AAV9/SURF1 HIGH DOSE (2.49E12 VG)
Fate: SCHEDULED SACRIFICE / Time on Test: 29 / Cause(s) of Death:

Gross or Microscopic Finding
Disposition
Tissue: Site / Finding, Severity

GROSS EXAMINATION

Disposition:

TGL #1: LUNG; GROSS LESION: DILATATION
// MICROSCOPIC CORRELATION: NO MICROSCOPIC CORRELATE

MICROSCOPIC EXAMINATION

Disposition: NORMAL

BRAIN, AMYGDALOID BODY
BRAIN, BASAL NUCLEI/STRIATUM
BRAIN, CEREBELLUM
BRAIN, CEREBRAL CORTEX
BRAIN, HIPPOCAMPUS
BRAIN, HYPOTHALAMUS
BRAIN, MEDULLA OBLONGATA
BRAIN, MENINGES
BRAIN, MIDBRAIN
BRAIN, OLFACTORY BULB
BRAIN, PONS
BRAIN, THALAMUS
BRAIN, VENTRICULAR SYSTEM
BRAIN, WHITE MATTER
EYE
LUNG
LYMPH NODE, ILIAC
LYMPH NODE, MANDIBULAR
LYMPH NODE, MESENTERIC
MUSCLE, BICEPS FEMORIS
MUSCLE, GASTROCNEMIUS
NERVE ROOT, SPINAL, CERVICAL
NERVE, OPTIC
PANCREAS
SPINAL CORD, CERVICAL
SPLEEN
TESTIS
THYMUS

Appendix 10

Individual Animal Report

Study ID: 02776-0018: CRL 2954-001 / U of Tex Southwestern Med UTSW.Gray-002
Study Title: A SINGLE DOSE TOXICITY STUDY OF AAV9/SURF1 ADMINISTERED BY INTRATHECAL INJECTION IN RATS

Animal: 4024 (Male)

Sacrifice: DAY 29
Group: 4MD29 / AAV9/SURF1 HIGH DOSE (2.49E12 VG)
Fate: SCHEDULED SACRIFICE / Time on Test: 29 / Cause(s) of Death:

Gross or Microscopic Finding
Disposition
Tissue: Site / Finding, Severity

Disposition: **ABNORMAL/FINDING**

GANGLION, DORSAL ROOT, CERVICAL
GANGLION, DORSAL ROOT, CERVICAL, GLIAL CELL: HYPERTROPHY/HYPERPLASIA, MINIMAL
GANGLION, DORSAL ROOT, LUMBAR
GANGLION, DORSAL ROOT, LUMBAR: INFILTRATE, MONONUCLEAR CELLS, MILD, MULTIFOCAL
GANGLION, DORSAL ROOT, LUMBAR, GLIAL CELL: HYPERTROPHY/HYPERPLASIA, MILD
GANGLION, DORSAL ROOT, LUMBAR, NEURON: DEGENERATION/NECROSIS, MINIMAL, SINGLE
GANGLION, DORSAL ROOT, THORACIC
GANGLION, DORSAL ROOT, THORACIC, GLIAL CELL: HYPERTROPHY/HYPERPLASIA, MINIMAL
HEART
HEART, MYOCARDIUM: DEGENERATION/NECROSIS, MINIMAL, MULTIFOCAL
HEART, MYOCARDIUM: INFILTRATE, MONONUCLEAR CELLS, MINIMAL, MULTIFOCAL
KIDNEY
KIDNEY: CHRONIC PROGRESSIVE NEPHROPATHY, MINIMAL
LIVER
LIVER: INFILTRATE, MIXED, MINIMAL
NERVE ROOT, SPINAL, LUMBAR
NERVE ROOT, SPINAL, LUMBAR, EPINEURIUM: INFILTRATE, MONONUCLEAR CELLS, MINIMAL, FOCAL
NERVE ROOT, SPINAL, LUMBAR, NERVE ROOT, DORSAL: DEGENERATION, NERVE FIBER, MILD
NERVE ROOT, SPINAL, THORACIC
NERVE ROOT, SPINAL, THORACIC, NERVE ROOT, DORSAL: DEGENERATION, NERVE FIBER, MINIMAL
NERVE, SCIATIC
NERVE, SCIATIC: DEGENERATION, NERVE FIBER, MILD
NERVE, TIBIAL
NERVE, TIBIAL: DEGENERATION, NERVE FIBER, MILD
NERVE, TIBIAL, SCHWANN CELL: HYPERTROPHY/HYPERPLASIA, MINIMAL
SPINAL CORD, LUMBAR
SPINAL CORD, LUMBAR, WHITE MATTER: DEGENERATION, NERVE FIBER, MINIMAL
COMMENT(S): SEEN IN LONG SECTION AND EXACT LOCATION COULD NOT BE DETERMINED
SPINAL CORD, THORACIC
SPINAL CORD, THORACIC, WHITE MATTER: DEGENERATION, NERVE FIBER, MINIMAL, DORSAL

Appendix 10

Individual Animal Report

Study ID: 02776-0018: CRL 2954-001 / U of Tex Southwestern Med UTSW.Gray-002
Study Title: A SINGLE DOSE TOXICITY STUDY OF AAV9/SURF1 ADMINISTERED BY INTRATHECAL INJECTION IN RATS

Animal: 4025 (Male)

Sacrifice: DAY 29
Group: 4MD29 / AAV9/SURF1 HIGH DOSE (2.49E12 VG)
Fate: SCHEDULED SACRIFICE / Time on Test: 29 / Cause(s) of Death:

Gross or Microscopic Finding
Disposition
Tissue: Site / Finding, Severity

MICROSCOPIC EXAMINATION

Disposition: NORMAL

BRAIN, AMYGDALOID BODY
BRAIN, BASAL NUCLEI/STRIATUM
BRAIN, CEREBELLUM
BRAIN, CEREBRAL CORTEX
BRAIN, HIPPOCAMPUS
BRAIN, HYPOTHALAMUS
BRAIN, MEDULLA OBLONGATA
BRAIN, MENINGES
BRAIN, MIDBRAIN
BRAIN, OLFACTORY BULB
BRAIN, PONS
BRAIN, THALAMUS
BRAIN, VENTRICULAR SYSTEM
BRAIN, WHITE MATTER
EYE
KIDNEY
LYMPH NODE, ILIAC
LYMPH NODE, MANDIBULAR
LYMPH NODE, MESENTERIC
MUSCLE, BICEPS FEMORIS
MUSCLE, GASTROCNEMIUS
NERVE ROOT, SPINAL, CERVICAL
NERVE ROOT, SPINAL, LUMBAR
NERVE ROOT, SPINAL, THORACIC
NERVE, OPTIC
NERVE, SCIATIC
NERVE, TIBIAL
SPINAL CORD, CERVICAL
SPINAL CORD, THORACIC
SPLEEN
TESTIS
THYMUS

Appendix 10

Individual Animal Report

Study ID: 02776-0018: CRL 2954-001 / U of Tex Southwestern Med UTSW.Gray-002
Study Title: A SINGLE DOSE TOXICITY STUDY OF AAV9/SURF1 ADMINISTERED BY INTRATHECAL INJECTION IN RATS

Animal: 4025 (Male)

Sacrifice: DAY 29
Group: 4MD29 / AAV9/SURF1 HIGH DOSE (2.49E12 VG)
Fate: SCHEDULED SACRIFICE / Time on Test: 29 / Cause(s) of Death:

Gross or Microscopic Finding
Disposition
Tissue: Site / Finding, Severity

Disposition: **ABNORMAL/FINDING**

GANGLION, DORSAL ROOT, CERVICAL
GANGLION, DORSAL ROOT, CERVICAL, GLIAL CELL: HYPERTROPHY/HYPERPLASIA, MINIMAL
GANGLION, DORSAL ROOT, LUMBAR
GANGLION, DORSAL ROOT, LUMBAR, GLIAL CELL: HYPERTROPHY/HYPERPLASIA, MINIMAL
GANGLION, DORSAL ROOT, THORACIC
GANGLION, DORSAL ROOT, THORACIC, GLIAL CELL: HYPERTROPHY/HYPERPLASIA, MINIMAL
HEART
HEART, MYOCARDIUM: DEGENERATION/NECROSIS, MINIMAL, MULTIFOCAL
HEART, MYOCARDIUM: INFILTRATE, MONONUCLEAR CELLS, MINIMAL, MULTIFOCAL
LIVER
LIVER: HYPERTROPHY/HYPERPLASIA, MILD
COMMENT(S): AFFECTING KUPFFER CELLS
LIVER: INFILTRATE, MIXED, MILD
LIVER, HEPATOCYTE: NECROSIS, SINGLE CELL, MILD
LUNG
LUNG: INFLAMMATION, MINIMAL, SUBACUTE, MULTIFOCAL
PANCREAS
PANCREAS: APOPTOSIS/SINGLE CELL NECROSIS, MINIMAL
SPINAL CORD, LUMBAR
SPINAL CORD, LUMBAR, NERVE ROOT, SPINAL: DEGENERATION, NERVE FIBER, MINIMAL

Appendix 10

Individual Animal Report

Study ID: 02776-0018: CRL 2954-001 / U of Tex Southwestern Med UTSW.Gray-002
Study Title: A SINGLE DOSE TOXICITY STUDY OF AAV9/SURF1 ADMINISTERED BY INTRATHECAL INJECTION IN RATS

Animal: 4026 (Male)

Sacrifice: DAY 29
Group: 4MD29 / AAV9/SURF1 HIGH DOSE (2.49E12 VG)
Fate: SCHEDULED SACRIFICE / Time on Test: 29 / Cause(s) of Death:

Gross or Microscopic Finding
Disposition
Tissue: Site / Finding, Severity

MICROSCOPIC EXAMINATION

Disposition: **NORMAL**

BRAIN, AMYGDALOID BODY
BRAIN, BASAL NUCLEI/STRIATUM
BRAIN, CEREBELLUM
BRAIN, CEREBRAL CORTEX
BRAIN, HIPPOCAMPUS
BRAIN, HYPOTHALAMUS
BRAIN, MEDULLA OBLONGATA
BRAIN, MENINGES
BRAIN, MIDBRAIN
BRAIN, OLFACTORY BULB
BRAIN, PONS
BRAIN, THALAMUS
BRAIN, VENTRICULAR SYSTEM
BRAIN, WHITE MATTER
EYE
GANGLION, DORSAL ROOT, CERVICAL
GANGLION, DORSAL ROOT, THORACIC
LUNG
LYMPH NODE, ILIAC
LYMPH NODE, MANDIBULAR
LYMPH NODE, MESENTERIC
MUSCLE, BICEPS FEMORIS
MUSCLE, GASTROCNEMIUS
NERVE ROOT, SPINAL, CERVICAL
NERVE ROOT, SPINAL, THORACIC
NERVE, OPTIC
NERVE, SCIATIC
NERVE, TIBIAL
PANCREAS
SPINAL CORD, CERVICAL
SPLEEN
TESTIS
THYMUS

Appendix 10

Individual Animal Report

Study ID: 02776-0018: CRL 2954-001 / U of Tex Southwestern Med UTSW.Gray-002
Study Title: A SINGLE DOSE TOXICITY STUDY OF AAV9/SURF1 ADMINISTERED BY INTRATHECAL INJECTION IN RATS

Animal: 4026 (Male)

Sacrifice: DAY 29
Group: 4MD29 / AAV9/SURF1 HIGH DOSE (2.49E12 VG)
Fate: SCHEDULED SACRIFICE / Time on Test: 29 / Cause(s) of Death:

Gross or Microscopic Finding
Disposition
Tissue: Site / Finding, Severity

Disposition: ABNORMAL/FINDING

GANGLION, DORSAL ROOT, LUMBAR
GANGLION, DORSAL ROOT, LUMBAR, GLIAL CELL: HYPERTROPHY/HYPERPLASIA, MINIMAL
HEART
HEART, MYOCARDIUM: DEGENERATION/NECROSIS, MILD, MULTIFOCAL
HEART, MYOCARDIUM: INFILTRATE, MONONUCLEAR CELLS, MILD, MULTIFOCAL
KIDNEY
KIDNEY, TUBULAR: CYST, MINIMAL
LIVER
LIVER: INFILTRATE, MIXED, MINIMAL
NERVE ROOT, SPINAL, LUMBAR
NERVE ROOT, SPINAL, LUMBAR, EPINEURIUM: INFILTRATE, MONONUCLEAR CELLS, MINIMAL, FOCAL
NERVE ROOT, SPINAL, LUMBAR, NERVE ROOT, DORSAL: DEGENERATION, NERVE FIBER, MINIMAL
SPINAL CORD, LUMBAR
SPINAL CORD, LUMBAR, NERVE ROOT, SPINAL: DEGENERATION, NERVE FIBER, MINIMAL
SPINAL CORD, THORACIC
SPINAL CORD, THORACIC, WHITE MATTER: DEGENERATION, NERVE FIBER, MINIMAL
COMMENT(S): SEEN IN LONG SECTION AND EXACT LOCATION COULD NOT BE DETERMINED

Appendix 10

Individual Animal Report

Study ID: 02776-0018: CRL 2954-001 / U of Tex Southwestern Med UTSW.Gray-002
Study Title: A SINGLE DOSE TOXICITY STUDY OF AAV9/SURF1 ADMINISTERED BY INTRATHECAL INJECTION IN RATS

Animal: 4027 (Male)

Sacrifice: DAY 29
Group: 4MD29 / AAV9/SURF1 HIGH DOSE (2.49E12 VG)
Fate: SCHEDULED SACRIFICE / Time on Test: 29 / Cause(s) of Death:

Gross or Microscopic Finding
Disposition
Tissue: Site / Finding, Severity

MICROSCOPIC EXAMINATION

Disposition: NORMAL

BRAIN, AMYGDALOID BODY
BRAIN, BASAL NUCLEI/STRIATUM
BRAIN, CEREBELLUM
BRAIN, CEREBRAL CORTEX
BRAIN, HIPPOCAMPUS
BRAIN, HYPOTHALAMUS
BRAIN, MEDULLA OBLONGATA
BRAIN, MENINGES
BRAIN, MIDBRAIN
BRAIN, OLFACTORY BULB
BRAIN, PONS
BRAIN, THALAMUS
BRAIN, VENTRICULAR SYSTEM
BRAIN, WHITE MATTER
EYE
GANGLION, DORSAL ROOT, THORACIC
LUNG
LYMPH NODE, ILIAC
LYMPH NODE, MANDIBULAR
LYMPH NODE, MESENTERIC
MUSCLE, BICEPS FEMORIS
MUSCLE, GASTROCNEMIUS
NERVE ROOT, SPINAL, CERVICAL
NERVE ROOT, SPINAL, THORACIC
NERVE, OPTIC
NERVE, SCIATIC
NERVE, TIBIAL
PANCREAS
SPINAL CORD, CERVICAL
SPINAL CORD, LUMBAR
SPINAL CORD, THORACIC
SPLEEN
TESTIS
THYMUS

Appendix 10

Individual Animal Report

Study ID: 02776-0018: CRL 2954-001 / U of Tex Southwestern Med UTSW.Gray-002
Study Title: A SINGLE DOSE TOXICITY STUDY OF AAV9/SURF1 ADMINISTERED BY INTRATHECAL INJECTION IN RATS

Animal: 4027 (Male)

Sacrifice: DAY 29
Group: 4MD29 / AAV9/SURF1 HIGH DOSE (2.49E12 VG)
Fate: SCHEDULED SACRIFICE / Time on Test: 29 / Cause(s) of Death:

Gross or Microscopic Finding
Disposition
Tissue: Site / Finding, Severity

Disposition: ABNORMAL/FINDING

GANGLION, DORSAL ROOT, CERVICAL
GANGLION, DORSAL ROOT, CERVICAL, GLIAL CELL: HYPERTROPHY/HYPERPLASIA, MINIMAL
GANGLION, DORSAL ROOT, LUMBAR
GANGLION, DORSAL ROOT, LUMBAR, GLIAL CELL: HYPERTROPHY/HYPERPLASIA, MINIMAL
HEART
HEART, MYOCARDIUM: DEGENERATION/NECROSIS, MINIMAL, MULTIFOCAL
HEART, MYOCARDIUM: INFILTRATE, MONONUCLEAR CELLS, MINIMAL, MULTIFOCAL
KIDNEY
KIDNEY: CHRONIC PROGRESSIVE NEPHROPATHY, MINIMAL
LIVER
LIVER: HYPERTROPHY/HYPERPLASIA, MINIMAL
COMMENT(S): AFFECTING KUPFFER CELLS
LIVER: INFILTRATE, MIXED, MILD
LIVER, HEPATOCYTE: NECROSIS, SINGLE CELL, MINIMAL
NERVE ROOT, SPINAL, LUMBAR
NERVE ROOT, SPINAL, LUMBAR, EPINEURIUM: INFILTRATE, MONONUCLEAR CELLS, MINIMAL, FOCAL

Appendix 10

Individual Animal Report

Study ID: 02776-0018: CRL 2954-001 / U of Tex Southwestern Med UTSW.Gray-002
Study Title: A SINGLE DOSE TOXICITY STUDY OF AAV9/SURF1 ADMINISTERED BY INTRATHECAL INJECTION IN RATS

Animal: 1521 (Female)

Sacrifice: DAY 29
Group: 1FD29 / VEHICLE (0 VG)
Fate: SCHEDULED SACRIFICE / Time on Test: 29 / Cause(s) of Death:

Gross or Microscopic Finding
Disposition
Tissue: Site / Finding, Severity

MICROSCOPIC EXAMINATION

Disposition: NORMAL

BRAIN, AMYGDALOID BODY
BRAIN, BASAL NUCLEI/STRIATUM
BRAIN, CEREBELLUM
BRAIN, CEREBRAL CORTEX
BRAIN, HIPPOCAMPUS
BRAIN, HYPOTHALAMUS
BRAIN, MEDULLA OBLONGATA
BRAIN, MENINGES
BRAIN, MIDBRAIN
BRAIN, OLFACTORY BULB
BRAIN, PONS
BRAIN, THALAMUS
BRAIN, VENTRICULAR SYSTEM
BRAIN, WHITE MATTER
EYE
GANGLION, DORSAL ROOT, CERVICAL
GANGLION, DORSAL ROOT, LUMBAR
GANGLION, DORSAL ROOT, THORACIC
HEART
KIDNEY
LUNG
LYMPH NODE, ILIAC
LYMPH NODE, MANDIBULAR
LYMPH NODE, MESENTERIC
MUSCLE, BICEPS FEMORIS
MUSCLE, GASTROCNEMIUS
NERVE ROOT, SPINAL, LUMBAR
NERVE ROOT, SPINAL, THORACIC
NERVE, OPTIC
NERVE, SCIATIC
NERVE, TIBIAL
OVARY
PANCREAS
SPINAL CORD, CERVICAL

Appendix 10

Individual Animal Report

Study ID: 02776-0018: CRL 2954-001 / U of Tex Southwestern Med UTSW.Gray-002
Study Title: A SINGLE DOSE TOXICITY STUDY OF AAV9/SURF1 ADMINISTERED BY INTRATHECAL INJECTION IN RATS

Animal: 1521 (Female)

Sacrifice: DAY 29
Group: 1FD29 / VEHICLE (0 VG)
Fate: SCHEDULED SACRIFICE / Time on Test: 29 / Cause(s) of Death:

Gross or Microscopic Finding
Disposition
Tissue: Site / Finding, Severity

SPINAL CORD, LUMBAR
SPINAL CORD, THORACIC
SPLEEN
THYMUS

Disposition: **ABNORMAL/FINDING**

LIVER
LIVER: INFILTRATE, MIXED, MINIMAL
LIVER, HEPATOCYTE: VACUOLATION, MINIMAL, FOCAL
NERVE ROOT, SPINAL, CERVICAL
NERVE ROOT, SPINAL, CERVICAL, EPINEURIUM: INFILTRATE, MONONUCLEAR CELLS, MINIMAL, FOCAL

Appendix 10

Individual Animal Report

Study ID: 02776-0018: CRL 2954-001 / U of Tex Southwestern Med UTSW.Gray-002
Study Title: A SINGLE DOSE TOXICITY STUDY OF AAV9/SURF1 ADMINISTERED BY INTRATHECAL INJECTION IN RATS

Animal: 1522 (Female)

Sacrifice: DAY 29
Group: 1FD29 / VEHICLE (0 VG)
Fate: SCHEDULED SACRIFICE / Time on Test: 29 / Cause(s) of Death:

Gross or Microscopic Finding
Disposition
Tissue: Site / Finding, Severity

MICROSCOPIC EXAMINATION

Disposition: NORMAL

BRAIN, AMYGDALOID BODY
BRAIN, BASAL NUCLEI/STRIATUM
BRAIN, CEREBELLUM
BRAIN, CEREBRAL CORTEX
BRAIN, HIPPOCAMPUS
BRAIN, HYPOTHALAMUS
BRAIN, MEDULLA OBLONGATA
BRAIN, MENINGES
BRAIN, MIDBRAIN
BRAIN, OLFACTORY BULB
BRAIN, PONS
BRAIN, THALAMUS
BRAIN, VENTRICULAR SYSTEM
BRAIN, WHITE MATTER
EYE
GANGLION, DORSAL ROOT, CERVICAL
GANGLION, DORSAL ROOT, THORACIC
HEART
LIVER
LUNG
LYMPH NODE, ILIAC
LYMPH NODE, MANDIBULAR
LYMPH NODE, MESENTERIC
MUSCLE, BICEPS FEMORIS
MUSCLE, GASTROCNEMIUS
NERVE ROOT, SPINAL, CERVICAL
NERVE ROOT, SPINAL, THORACIC
NERVE, OPTIC
NERVE, SCIATIC
NERVE, TIBIAL
OVARY
PANCREAS
SPINAL CORD, CERVICAL
SPINAL CORD, LUMBAR

Appendix 10

Individual Animal Report

Study ID: 02776-0018: CRL 2954-001 / U of Tex Southwestern Med UTSW.Gray-002
Study Title: A SINGLE DOSE TOXICITY STUDY OF AAV9/SURF1 ADMINISTERED BY INTRATHECAL INJECTION IN RATS

Animal: 1522 (Female)

Sacrifice: DAY 29
Group: 1FD29 / VEHICLE (0 VG)
Fate: SCHEDULED SACRIFICE / Time on Test: 29 / Cause(s) of Death:

Gross or Microscopic Finding
Disposition
Tissue: Site / Finding, Severity

SPINAL CORD, THORACIC
SPLEEN
THYMUS

Disposition: **ABNORMAL/FINDING**

GANGLION, DORSAL ROOT, LUMBAR
GANGLION, DORSAL ROOT, LUMBAR: INFILTRATE, MONONUCLEAR CELLS, MINIMAL, FOCAL
KIDNEY
KIDNEY, TUBULAR: BASOPHILIA, MINIMAL
NERVE ROOT, SPINAL, LUMBAR
NERVE ROOT, SPINAL, LUMBAR, EPINEURIUM: INFILTRATE, MONONUCLEAR CELLS, MINIMAL, FOCAL

Appendix 10

Individual Animal Report

Study ID: 02776-0018: CRL 2954-001 / U of Tex Southwestern Med UTSW.Gray-002
Study Title: A SINGLE DOSE TOXICITY STUDY OF AAV9/SURF1 ADMINISTERED BY INTRATHECAL INJECTION IN RATS

Animal: 1523 (Female)

Sacrifice: DAY 29
Group: 1FD29 / VEHICLE (0 VG)
Fate: SCHEDULED SACRIFICE / Time on Test: 29 / Cause(s) of Death:

Gross or Microscopic Finding
Disposition
Tissue: Site / Finding, Severity

MICROSCOPIC EXAMINATION

Disposition: NORMAL

BRAIN, AMYGDALOID BODY
BRAIN, BASAL NUCLEI/STRIATUM
BRAIN, CEREBELLUM
BRAIN, CEREBRAL CORTEX
BRAIN, HIPPOCAMPUS
BRAIN, HYPOTHALAMUS
BRAIN, MEDULLA OBLONGATA
BRAIN, MENINGES
BRAIN, MIDBRAIN
BRAIN, OLFACTORY BULB
BRAIN, PONS
BRAIN, THALAMUS
BRAIN, VENTRICULAR SYSTEM
BRAIN, WHITE MATTER
EYE
GANGLION, DORSAL ROOT, CERVICAL
GANGLION, DORSAL ROOT, LUMBAR
GANGLION, DORSAL ROOT, THORACIC
HEART
KIDNEY
LIVER
LYMPH NODE, ILIAC
LYMPH NODE, MANDIBULAR
LYMPH NODE, MESENTERIC
MUSCLE, BICEPS FEMORIS
MUSCLE, GASTROCNEMIUS
NERVE ROOT, SPINAL, CERVICAL
NERVE ROOT, SPINAL, THORACIC
NERVE, OPTIC
NERVE, SCIATIC
NERVE, TIBIAL
OVARY
PANCREAS
SPINAL CORD, CERVICAL

Appendix 10

Individual Animal Report

Study ID: 02776-0018: CRL 2954-001 / U of Tex Southwestern Med UTSW.Gray-002
Study Title: A SINGLE DOSE TOXICITY STUDY OF AAV9/SURF1 ADMINISTERED BY INTRATHECAL INJECTION IN RATS

Animal: 1523 (Female)

Sacrifice: DAY 29
Group: 1FD29 / VEHICLE (0 VG)
Fate: SCHEDULED SACRIFICE / Time on Test: 29 / Cause(s) of Death:

Gross or Microscopic Finding
Disposition
Tissue: Site / Finding, Severity

SPINAL CORD, LUMBAR
SPINAL CORD, THORACIC
SPLEEN
THYMUS

Disposition: **ABNORMAL/FINDING**

LUNG
LUNG: INFLAMMATION, MINIMAL, SUBACUTE, FOCAL
NERVE ROOT, SPINAL, LUMBAR
NERVE ROOT, SPINAL, LUMBAR, EPINEURIUM: INFILTRATE, MONONUCLEAR CELLS, MINIMAL, FOCAL

Appendix 10

Individual Animal Report

Study ID: 02776-0018: CRL 2954-001 / U of Tex Southwestern Med UTSW.Gray-002
Study Title: A SINGLE DOSE TOXICITY STUDY OF AAV9/SURF1 ADMINISTERED BY INTRATHECAL INJECTION IN RATS

Animal: 1524 (Female)

Sacrifice: DAY 29
Group: 1FD29 / VEHICLE (0 VG)
Fate: SCHEDULED SACRIFICE / Time on Test: 29 / Cause(s) of Death:

Gross or Microscopic Finding
Disposition
Tissue: Site / Finding, Severity

MICROSCOPIC EXAMINATION

Disposition: **NORMAL**

BRAIN, AMYGDALOID BODY
BRAIN, BASAL NUCLEI/STRIATUM
BRAIN, CEREBELLUM
BRAIN, CEREBRAL CORTEX
BRAIN, HIPPOCAMPUS
BRAIN, HYPOTHALAMUS
BRAIN, MEDULLA OBLONGATA
BRAIN, MENINGES
BRAIN, MIDBRAIN
BRAIN, OLFACTORY BULB
BRAIN, PONS
BRAIN, THALAMUS
BRAIN, VENTRICULAR SYSTEM
BRAIN, WHITE MATTER
EYE
GANGLION, DORSAL ROOT, CERVICAL
GANGLION, DORSAL ROOT, LUMBAR
HEART
KIDNEY
LYMPH NODE, ILIAC
LYMPH NODE, MANDIBULAR
LYMPH NODE, MESENTERIC
MUSCLE, BICEPS FEMORIS
MUSCLE, GASTROCNEMIUS
NERVE ROOT, SPINAL, CERVICAL
NERVE ROOT, SPINAL, LUMBAR
NERVE ROOT, SPINAL, THORACIC
NERVE, OPTIC
NERVE, SCIATIC
NERVE, TIBIAL
OVARY
PANCREAS
SPINAL CORD, CERVICAL
SPINAL CORD, LUMBAR

Appendix 10

Individual Animal Report

Study ID: 02776-0018: CRL 2954-001 / U of Tex Southwestern Med UTSW.Gray-002
Study Title: A SINGLE DOSE TOXICITY STUDY OF AAV9/SURF1 ADMINISTERED BY INTRATHECAL INJECTION IN RATS

Animal: 1524 (Female)

Sacrifice: DAY 29
Group: 1FD29 / VEHICLE (0 VG)
Fate: SCHEDULED SACRIFICE / Time on Test: 29 / Cause(s) of Death:

Gross or Microscopic Finding
Disposition
Tissue: Site / Finding, Severity

SPINAL CORD, THORACIC
SPLEEN
THYMUS

Disposition: **ABNORMAL/FINDING**

GANGLION, DORSAL ROOT, THORACIC
GANGLION, DORSAL ROOT, THORACIC, GLIAL CELL: HYPERTROPHY/HYPERPLASIA, MINIMAL
LIVER
LIVER: INFILTRATE, MIXED, MINIMAL
LUNG
LUNG, PERIVASCULAR: INFILTRATE, EOSINOPHILS, MINIMAL, FOCAL

Appendix 10

Individual Animal Report

Study ID: 02776-0018: CRL 2954-001 / U of Tex Southwestern Med UTSW.Gray-002
Study Title: A SINGLE DOSE TOXICITY STUDY OF AAV9/SURF1 ADMINISTERED BY INTRATHECAL INJECTION IN RATS

Animal: 1525 (Female)

Sacrifice: DAY 29
Group: 1FD29 / VEHICLE (0 VG)
Fate: SCHEDULED SACRIFICE / Time on Test: 29 / Cause(s) of Death:

Gross or Microscopic Finding
Disposition
Tissue: Site / Finding, Severity

MICROSCOPIC EXAMINATION

Disposition: NORMAL

BRAIN, AMYGDALOID BODY
BRAIN, BASAL NUCLEI/STRIATUM
BRAIN, CEREBELLUM
BRAIN, CEREBRAL CORTEX
BRAIN, HIPPOCAMPUS
BRAIN, HYPOTHALAMUS
BRAIN, MEDULLA OBLONGATA
BRAIN, MENINGES
BRAIN, MIDBRAIN
BRAIN, OLFACTORY BULB
BRAIN, PONS
BRAIN, THALAMUS
BRAIN, VENTRICULAR SYSTEM
BRAIN, WHITE MATTER
EYE
GANGLION, DORSAL ROOT, LUMBAR
GANGLION, DORSAL ROOT, THORACIC
HEART
LUNG
LYMPH NODE, ILIAC
LYMPH NODE, MANDIBULAR
LYMPH NODE, MESENTERIC
MUSCLE, BICEPS FEMORIS
MUSCLE, GASTROCNEMIUS
NERVE ROOT, SPINAL, CERVICAL
NERVE ROOT, SPINAL, LUMBAR
NERVE, OPTIC
NERVE, SCIATIC
NERVE, TIBIAL
OVARY
PANCREAS
SPINAL CORD, CERVICAL
SPINAL CORD, LUMBAR
SPINAL CORD, THORACIC

Appendix 10

Individual Animal Report

Study ID: 02776-0018: CRL 2954-001 / U of Tex Southwestern Med UTSW.Gray-002
Study Title: A SINGLE DOSE TOXICITY STUDY OF AAV9/SURF1 ADMINISTERED BY INTRATHECAL INJECTION IN RATS

Animal: 1525 (Female)

Sacrifice: DAY 29
Group: 1FD29 / VEHICLE (0 VG)
Fate: SCHEDULED SACRIFICE / Time on Test: 29 / Cause(s) of Death:

Gross or Microscopic Finding
Disposition
Tissue: Site / Finding, Severity

SPLEEN
THYMUS

Disposition: **ABNORMAL/FINDING**

GANGLION, DORSAL ROOT, CERVICAL
GANGLION, DORSAL ROOT, CERVICAL, GLIAL CELL: HYPERTROPHY/HYPERPLASIA, MINIMAL
KIDNEY
KIDNEY: CHRONIC PROGRESSIVE NEPHROPATHY, MINIMAL
LIVER
LIVER: INFILTRATE, MIXED, MINIMAL
NERVE ROOT, SPINAL, THORACIC
NERVE ROOT, SPINAL, THORACIC, NERVE ROOT, VENTRAL: DEGENERATION, NERVE FIBER, MINIMAL

Appendix 10

Individual Animal Report

Study ID: 02776-0018: CRL 2954-001 / U of Tex Southwestern Med UTSW.Gray-002
Study Title: A SINGLE DOSE TOXICITY STUDY OF AAV9/SURF1 ADMINISTERED BY INTRATHECAL INJECTION IN RATS

Animal: 2521 (Female)

Sacrifice: DAY 29
Group: 2FD29 / AAV9/SURF1 LOW DOSE (0.28E12 VG)
Fate: SCHEDULED SACRIFICE / Time on Test: 29 / Cause(s) of Death:

Gross or Microscopic Finding
Disposition
Tissue: Site / Finding, Severity

MICROSCOPIC EXAMINATION

Disposition: **NORMAL**

BRAIN, AMYGDALOID BODY
BRAIN, BASAL NUCLEI/STRIATUM
BRAIN, CEREBELLUM
BRAIN, CEREBRAL CORTEX
BRAIN, HIPPOCAMPUS
BRAIN, HYPOTHALAMUS
BRAIN, MEDULLA OBLONGATA
BRAIN, MENINGES
BRAIN, MIDBRAIN
BRAIN, OLFACTORY BULB
BRAIN, PONS
BRAIN, THALAMUS
BRAIN, VENTRICULAR SYSTEM
BRAIN, WHITE MATTER
EYE
GANGLION, DORSAL ROOT, CERVICAL
GANGLION, DORSAL ROOT, LUMBAR
GANGLION, DORSAL ROOT, THORACIC
KIDNEY
LUNG
LYMPH NODE, ILIAC
LYMPH NODE, MANDIBULAR
LYMPH NODE, MESENTERIC
MUSCLE, BICEPS FEMORIS
MUSCLE, GASTROCNEMIUS
NERVE ROOT, SPINAL, CERVICAL
NERVE ROOT, SPINAL, LUMBAR
NERVE ROOT, SPINAL, THORACIC
NERVE, OPTIC
NERVE, SCIATIC
NERVE, TIBIAL
OVARY
PANCREAS
SPINAL CORD, CERVICAL

Appendix 10

Individual Animal Report

Study ID: 02776-0018: CRL 2954-001 / U of Tex Southwestern Med UTSW.Gray-002
Study Title: A SINGLE DOSE TOXICITY STUDY OF AAV9/SURF1 ADMINISTERED BY INTRATHECAL INJECTION IN RATS

Animal: 2521 (Female)

Sacrifice: DAY 29
Group: 2FD29 / AAV9/SURF1 LOW DOSE (0.28E12 VG)
Fate: SCHEDULED SACRIFICE / Time on Test: 29 / Cause(s) of Death:

Gross or Microscopic Finding
Disposition
Tissue: Site / Finding, Severity

SPLEEN
THYMUS

Disposition: **ABNORMAL/FINDING**

HEART
HEART, MYOCARDIUM: DEGENERATION/NECROSIS, MILD
HEART, MYOCARDIUM: FIBROSIS, MINIMAL
HEART, MYOCARDIUM: INFILTRATE, MONONUCLEAR CELLS, MILD

LIVER
LIVER: INFILTRATE, MIXED, MINIMAL

SPINAL CORD, LUMBAR
SPINAL CORD, LUMBAR, WHITE MATTER: DEGENERATION, NERVE FIBER, MINIMAL
COMMENT(S): SEEN IN LONG SECTION AND EXACT LOCATION COULD NOT BE DETERMINED

SPINAL CORD, THORACIC
SPINAL CORD, THORACIC, WHITE MATTER: DEGENERATION, NERVE FIBER, MINIMAL
COMMENT(S): SEEN IN LONG SECTION AND EXACT LOCATION COULD NOT BE DETERMINED

Appendix 10

Individual Animal Report

Study ID: 02776-0018: CRL 2954-001 / U of Tex Southwestern Med UTSW.Gray-002
Study Title: A SINGLE DOSE TOXICITY STUDY OF AAV9/SURF1 ADMINISTERED BY INTRATHECAL INJECTION IN RATS

Animal: 2522 (Female)

Sacrifice: DAY 29
Group: 2FD29 / AAV9/SURF1 LOW DOSE (0.28E12 VG)
Fate: SCHEDULED SACRIFICE / Time on Test: 29 / Cause(s) of Death:

Gross or Microscopic Finding
Disposition
Tissue: Site / Finding, Severity

MICROSCOPIC EXAMINATION

Disposition: **NORMAL**

BRAIN, AMYGDALOID BODY
BRAIN, BASAL NUCLEI/STRIATUM
BRAIN, CEREBELLUM
BRAIN, CEREBRAL CORTEX
BRAIN, HIPPOCAMPUS
BRAIN, HYPOTHALAMUS
BRAIN, MEDULLA OBLONGATA
BRAIN, MENINGES
BRAIN, MIDBRAIN
BRAIN, OLFACTORY BULB
BRAIN, PONS
BRAIN, THALAMUS
BRAIN, VENTRICULAR SYSTEM
BRAIN, WHITE MATTER
EYE
GANGLION, DORSAL ROOT, CERVICAL
GANGLION, DORSAL ROOT, LUMBAR
GANGLION, DORSAL ROOT, THORACIC
KIDNEY
LUNG
LYMPH NODE, ILIAC
LYMPH NODE, MANDIBULAR
LYMPH NODE, MESENTERIC
MUSCLE, BICEPS FEMORIS
MUSCLE, GASTROCNEMIUS
NERVE ROOT, SPINAL, CERVICAL
NERVE ROOT, SPINAL, LUMBAR
NERVE ROOT, SPINAL, THORACIC
NERVE, OPTIC
NERVE, SCIATIC
NERVE, TIBIAL
OVARY
PANCREAS
SPINAL CORD, CERVICAL

Appendix 10

Individual Animal Report

Study ID: 02776-0018: CRL 2954-001 / U of Tex Southwestern Med UTSW.Gray-002
Study Title: A SINGLE DOSE TOXICITY STUDY OF AAV9/SURF1 ADMINISTERED BY INTRATHECAL INJECTION IN RATS

Animal: 2522 (Female)

Sacrifice: DAY 29
Group: 2FD29 / AAV9/SURF1 LOW DOSE (0.28E12 VG)
Fate: SCHEDULED SACRIFICE / Time on Test: 29 / Cause(s) of Death:

Gross or Microscopic Finding
Disposition
Tissue: Site / Finding, Severity

SPINAL CORD, THORACIC
SPLEEN
THYMUS

Disposition: **ABNORMAL/FINDING**

HEART
HEART, MYOCARDIUM: DEGENERATION/NECROSIS, MINIMAL
HEART, MYOCARDIUM: INFILTRATE, MONONUCLEAR CELLS, MINIMAL
LIVER
LIVER: INFILTRATE, MIXED, MILD
SPINAL CORD, LUMBAR
SPINAL CORD, LUMBAR, WHITE MATTER: DEGENERATION, NERVE FIBER, MINIMAL, LATERAL

Appendix 10

Individual Animal Report

Study ID: 02776-0018: CRL 2954-001 / U of Tex Southwestern Med UTSW.Gray-002
Study Title: A SINGLE DOSE TOXICITY STUDY OF AAV9/SURF1 ADMINISTERED BY INTRATHECAL INJECTION IN RATS

Animal: 2523 (Female)

Sacrifice: DAY 29
Group: 2FD29 / AAV9/SURF1 LOW DOSE (0.28E12 VG)
Fate: SCHEDULED SACRIFICE / Time on Test: 29 / Cause(s) of Death:

Gross or Microscopic Finding
Disposition
Tissue: Site / Finding, Severity

MICROSCOPIC EXAMINATION

Disposition: **NORMAL**

BRAIN, AMYGDALOID BODY
BRAIN, BASAL NUCLEI/STRIATUM
BRAIN, CEREBELLUM
BRAIN, CEREBRAL CORTEX
BRAIN, HIPPOCAMPUS
BRAIN, HYPOTHALAMUS
BRAIN, MEDULLA OBLONGATA
BRAIN, MENINGES
BRAIN, MIDBRAIN
BRAIN, OLFACTORY BULB
BRAIN, PONS
BRAIN, THALAMUS
BRAIN, VENTRICULAR SYSTEM
BRAIN, WHITE MATTER
EYE
GANGLION, DORSAL ROOT, CERVICAL
GANGLION, DORSAL ROOT, LUMBAR
GANGLION, DORSAL ROOT, THORACIC
KIDNEY
LIVER
LUNG
LYMPH NODE, ILIAC
LYMPH NODE, MANDIBULAR
LYMPH NODE, MESENTERIC
MUSCLE, BICEPS FEMORIS
MUSCLE, GASTROCNEMIUS
NERVE ROOT, SPINAL, CERVICAL
NERVE ROOT, SPINAL, THORACIC
NERVE, OPTIC
OVARY
PANCREAS
SPINAL CORD, CERVICAL
SPINAL CORD, THORACIC
SPLEEN

Appendix 10

Individual Animal Report

Study ID: 02776-0018: CRL 2954-001 / U of Tex Southwestern Med UTSW.Gray-002
Study Title: A SINGLE DOSE TOXICITY STUDY OF AAV9/SURF1 ADMINISTERED BY INTRATHECAL INJECTION IN RATS

Animal: 2523 (Female)

Sacrifice: DAY 29
Group: 2FD29 / AAV9/SURF1 LOW DOSE (0.28E12 VG)
Fate: SCHEDULED SACRIFICE / Time on Test: 29 / Cause(s) of Death:

Gross or Microscopic Finding
Disposition
Tissue: Site / Finding, Severity

THYMUS

Disposition: **ABNORMAL/FINDING**

HEART
HEART, MYOCARDIUM: DEGENERATION/NECROSIS, MINIMAL
HEART, MYOCARDIUM: INFILTRATE, MONONUCLEAR CELLS, MINIMAL
NERVE ROOT, SPINAL, LUMBAR
NERVE ROOT, SPINAL, LUMBAR, NERVE ROOT, VENTRAL: DEGENERATION, NERVE FIBER, MINIMAL
NERVE, SCIATIC
NERVE, SCIATIC: DEGENERATION, NERVE FIBER, MILD
NERVE, TIBIAL
NERVE, TIBIAL: DEGENERATION, NERVE FIBER, MILD
SPINAL CORD, LUMBAR
SPINAL CORD, LUMBAR, WHITE MATTER: DEGENERATION, NERVE FIBER, MINIMAL, DORSAL

Appendix 10

Individual Animal Report

Study ID: 02776-0018: CRL 2954-001 / U of Tex Southwestern Med UTSW.Gray-002
Study Title: A SINGLE DOSE TOXICITY STUDY OF AAV9/SURF1 ADMINISTERED BY INTRATHECAL INJECTION IN RATS

Animal: 2524 (Female)

Sacrifice: DAY 29
Group: 2FD29 / AAV9/SURF1 LOW DOSE (0.28E12 VG)
Fate: SCHEDULED SACRIFICE / Time on Test: 29 / Cause(s) of Death:

Gross or Microscopic Finding
Disposition
Tissue: Site / Finding, Severity

MICROSCOPIC EXAMINATION

Disposition: NORMAL

BRAIN, AMYGDALOID BODY
BRAIN, BASAL NUCLEI/STRIATUM
BRAIN, CEREBELLUM
BRAIN, CEREBRAL CORTEX
BRAIN, HIPPOCAMPUS
BRAIN, HYPOTHALAMUS
BRAIN, MEDULLA OBLONGATA
BRAIN, MENINGES
BRAIN, MIDBRAIN
BRAIN, OLFACTORY BULB
BRAIN, PONS
BRAIN, THALAMUS
BRAIN, VENTRICULAR SYSTEM
BRAIN, WHITE MATTER
EYE
GANGLION, DORSAL ROOT, CERVICAL
GANGLION, DORSAL ROOT, LUMBAR
GANGLION, DORSAL ROOT, THORACIC
KIDNEY
LUNG
LYMPH NODE, ILIAC
LYMPH NODE, MANDIBULAR
LYMPH NODE, MESENTERIC
MUSCLE, BICEPS FEMORIS
MUSCLE, GASTROCNEMIUS
NERVE ROOT, SPINAL, CERVICAL
NERVE ROOT, SPINAL, THORACIC
NERVE, OPTIC
NERVE, SCIATIC
NERVE, TIBIAL
OVARY
PANCREAS
SPINAL CORD, CERVICAL
SPINAL CORD, LUMBAR

Appendix 10

Individual Animal Report

Study ID: 02776-0018: CRL 2954-001 / U of Tex Southwestern Med UTSW.Gray-002
Study Title: A SINGLE DOSE TOXICITY STUDY OF AAV9/SURF1 ADMINISTERED BY INTRATHECAL INJECTION IN RATS

Animal: 2524 (Female)

Sacrifice: DAY 29
Group: 2FD29 / AAV9/SURF1 LOW DOSE (0.28E12 VG)
Fate: SCHEDULED SACRIFICE / Time on Test: 29 / Cause(s) of Death:

Gross or Microscopic Finding
Disposition
Tissue: Site / Finding, Severity

SPINAL CORD, THORACIC
SPLEEN
THYMUS

Disposition: **ABNORMAL/FINDING**

HEART
HEART, MYOCARDIUM: DEGENERATION/NECROSIS, MILD
HEART, MYOCARDIUM: INFILTRATE, MONONUCLEAR CELLS, MILD
LIVER
LIVER: INFILTRATE, MIXED, MINIMAL
NERVE ROOT, SPINAL, LUMBAR
NERVE ROOT, SPINAL, LUMBAR, NERVE ROOT, VENTRAL: DEGENERATION, NERVE FIBER, MINIMAL

Appendix 10

Individual Animal Report

Study ID: 02776-0018: CRL 2954-001 / U of Tex Southwestern Med UTSW.Gray-002
Study Title: A SINGLE DOSE TOXICITY STUDY OF AAV9/SURF1 ADMINISTERED BY INTRATHECAL INJECTION IN RATS

Animal: 2525 (Female)

Sacrifice: DAY 29
Group: 2FD29 / AAV9/SURF1 LOW DOSE (0.28E12 VG)
Fate: SCHEDULED SACRIFICE / Time on Test: 29 / Cause(s) of Death:

Gross or Microscopic Finding
Disposition
Tissue: Site / Finding, Severity

MICROSCOPIC EXAMINATION

Disposition: NORMAL

BRAIN, AMYGDALOID BODY
BRAIN, BASAL NUCLEI/STRIATUM
BRAIN, CEREBELLUM
BRAIN, CEREBRAL CORTEX
BRAIN, HIPPOCAMPUS
BRAIN, HYPOTHALAMUS
BRAIN, MEDULLA OBLONGATA
BRAIN, MENINGES
BRAIN, MIDBRAIN
BRAIN, OLFACTORY BULB
BRAIN, PONS
BRAIN, THALAMUS
BRAIN, VENTRICULAR SYSTEM
BRAIN, WHITE MATTER
EYE
GANGLION, DORSAL ROOT, CERVICAL
GANGLION, DORSAL ROOT, LUMBAR
GANGLION, DORSAL ROOT, THORACIC
KIDNEY
LYMPH NODE, ILIAC
LYMPH NODE, MANDIBULAR
LYMPH NODE, MESENTERIC
MUSCLE, BICEPS FEMORIS
MUSCLE, GASTROCNEMIUS
NERVE ROOT, SPINAL, CERVICAL
NERVE ROOT, SPINAL, LUMBAR
NERVE ROOT, SPINAL, THORACIC
NERVE, OPTIC
NERVE, TIBIAL
OVARY
PANCREAS
SPINAL CORD, CERVICAL
SPINAL CORD, LUMBAR
SPLEEN

Appendix 10

Individual Animal Report

Study ID: 02776-0018: CRL 2954-001 / U of Tex Southwestern Med UTSW.Gray-002
Study Title: A SINGLE DOSE TOXICITY STUDY OF AAV9/SURF1 ADMINISTERED BY INTRATHECAL INJECTION IN RATS

Animal: 2525 (Female)

Sacrifice: DAY 29
Group: 2FD29 / AAV9/SURF1 LOW DOSE (0.28E12 VG)
Fate: SCHEDULED SACRIFICE / Time on Test: 29 / Cause(s) of Death:

Gross or Microscopic Finding
Disposition
Tissue: Site / Finding, Severity

THYMUS

Disposition: **ABNORMAL/FINDING**

HEART
HEART, MYOCARDIUM: DEGENERATION/NECROSIS, MARKED
HEART, MYOCARDIUM: FIBROSIS, MODERATE
HEART, MYOCARDIUM: INFILTRATE, MONONUCLEAR CELLS, MARKED
LIVER
LIVER: INFILTRATE, MIXED, MINIMAL
LUNG
LUNG: INFLAMMATION, MILD, SUBACUTE, MULTIFOCAL
NERVE, SCIATIC
NERVE, SCIATIC: DEGENERATION, NERVE FIBER, MINIMAL
SPINAL CORD, THORACIC
SPINAL CORD, THORACIC, WHITE MATTER: DEGENERATION, NERVE FIBER, MINIMAL, DORSAL

Appendix 10

Individual Animal Report

Study ID: 02776-0018: CRL 2954-001 / U of Tex Southwestern Med UTSW.Gray-002
Study Title: A SINGLE DOSE TOXICITY STUDY OF AAV9/SURF1 ADMINISTERED BY INTRATHECAL INJECTION IN RATS

Animal: 3521 (Female)

Sacrifice: DAY 29
Group: 3FD29 / AAV9/SURF1 MID DOSE (0.83E12 VG)
Fate: SCHEDULED SACRIFICE / Time on Test: 29 / Cause(s) of Death:

Gross or Microscopic Finding
Disposition
Tissue: Site / Finding, Severity

MICROSCOPIC EXAMINATION

Disposition: NORMAL

BRAIN, AMYGDALOID BODY
BRAIN, BASAL NUCLEI/STRIATUM
BRAIN, CEREBELLUM
BRAIN, CEREBRAL CORTEX
BRAIN, HIPPOCAMPUS
BRAIN, HYPOTHALAMUS
BRAIN, MEDULLA OBLONGATA
BRAIN, MENINGES
BRAIN, MIDBRAIN
BRAIN, OLFACTORY BULB
BRAIN, THALAMUS
BRAIN, VENTRICULAR SYSTEM
BRAIN, WHITE MATTER
EYE
GANGLION, DORSAL ROOT, THORACIC
KIDNEY
LUNG
LYMPH NODE, ILIAC
LYMPH NODE, MANDIBULAR
LYMPH NODE, MESENTERIC
MUSCLE, BICEPS FEMORIS
MUSCLE, GASTROCNEMIUS
NERVE ROOT, SPINAL, CERVICAL
NERVE ROOT, SPINAL, THORACIC
NERVE, OPTIC
OVARY
PANCREAS
SPLEEN
THYMUS

Disposition: ABNORMAL/FINDING

BRAIN, PONS
BRAIN, PONS: DEGENERATION, NERVE FIBER, MINIMAL
GANGLION, DORSAL ROOT, CERVICAL

Appendix 10

Individual Animal Report

Study ID: 02776-0018: CRL 2954-001 / U of Tex Southwestern Med UTSW.Gray-002
Study Title: A SINGLE DOSE TOXICITY STUDY OF AAV9/SURF1 ADMINISTERED BY INTRATHECAL INJECTION IN RATS

Animal: 3521 (Female)

Sacrifice: DAY 29
Group: 3FD29 / AAV9/SURF1 MID DOSE (0.83E12 VG)
Fate: SCHEDULED SACRIFICE / Time on Test: 29 / Cause(s) of Death:

Gross or Microscopic Finding
Disposition
Tissue: Site / Finding, Severity

GANGLION, DORSAL ROOT, CERVICAL, GLIAL CELL: HYPERTROPHY/HYPERPLASIA, MINIMAL
GANGLION, DORSAL ROOT, LUMBAR
GANGLION, DORSAL ROOT, LUMBAR, GLIAL CELL: HYPERTROPHY/HYPERPLASIA, MINIMAL
HEART
HEART, MYOCARDIUM: DEGENERATION/NECROSIS, MODERATE
HEART, MYOCARDIUM: INFILTRATE, MONONUCLEAR CELLS, MODERATE
LIVER
LIVER: INFILTRATE, MIXED, MINIMAL
NERVE ROOT, SPINAL, LUMBAR
NERVE ROOT, SPINAL, LUMBAR, NERVE ROOT, VENTRAL: DEGENERATION, NERVE FIBER, MINIMAL
NERVE, SCIATIC
NERVE, SCIATIC: DEGENERATION, NERVE FIBER, MODERATE
NERVE, TIBIAL
NERVE, TIBIAL: DEGENERATION, NERVE FIBER, MODERATE
SPINAL CORD, CERVICAL
SPINAL CORD, CERVICAL: INFILTRATE, MONONUCLEAR CELLS, MINIMAL, FOCAL
SPINAL CORD, CERVICAL, WHITE MATTER: DEGENERATION, NERVE FIBER, MINIMAL
COMMENT(S): SEEN IN LONG SECTION AND EXACT LOCATION COULD NOT BE DETERMINED
SPINAL CORD, LUMBAR
SPINAL CORD, LUMBAR: INFILTRATE, MONONUCLEAR CELLS, MINIMAL, FOCAL
SPINAL CORD, LUMBAR, WHITE MATTER: DEGENERATION, NERVE FIBER, MINIMAL, DORSAL
SPINAL CORD, THORACIC
SPINAL CORD, THORACIC, WHITE MATTER: DEGENERATION, NERVE FIBER, MINIMAL
COMMENT(S): SEEN IN LONG SECTION AND EXACT LOCATION COULD NOT BE DETERMINED

Appendix 10

Individual Animal Report

Study ID: 02776-0018: CRL 2954-001 / U of Tex Southwestern Med UTSW.Gray-002
Study Title: A SINGLE DOSE TOXICITY STUDY OF AAV9/SURF1 ADMINISTERED BY INTRATHECAL INJECTION IN RATS

Animal: 3522 (Female)

Sacrifice: DAY 29
Group: 3FD29 / AAV9/SURF1 MID DOSE (0.83E12 VG)
Fate: SCHEDULED SACRIFICE / Time on Test: 29 / Cause(s) of Death:

Gross or Microscopic Finding
Disposition
Tissue: Site / Finding, Severity

MICROSCOPIC EXAMINATION

Disposition: NORMAL

BRAIN, AMYGDALOID BODY
BRAIN, BASAL NUCLEI/STRIATUM
BRAIN, CEREBELLUM
BRAIN, CEREBRAL CORTEX
BRAIN, HIPPOCAMPUS
BRAIN, HYPOTHALAMUS
BRAIN, MEDULLA OBLONGATA
BRAIN, MENINGES
BRAIN, MIDBRAIN
BRAIN, OLFACTORY BULB
BRAIN, PONS
BRAIN, THALAMUS
BRAIN, VENTRICULAR SYSTEM
BRAIN, WHITE MATTER
EYE
GANGLION, DORSAL ROOT, CERVICAL
GANGLION, DORSAL ROOT, THORACIC
KIDNEY
LUNG
LYMPH NODE, ILIAC
LYMPH NODE, MANDIBULAR
LYMPH NODE, MESENTERIC
MUSCLE, BICEPS FEMORIS
MUSCLE, GASTROCNEMIUS
NERVE ROOT, SPINAL, CERVICAL
NERVE ROOT, SPINAL, LUMBAR
NERVE ROOT, SPINAL, THORACIC
NERVE, OPTIC
OVARY
PANCREAS
SPINAL CORD, CERVICAL
SPLEEN
THYMUS

Appendix 10

Individual Animal Report

Study ID: 02776-0018: CRL 2954-001 / U of Tex Southwestern Med UTSW.Gray-002
Study Title: A SINGLE DOSE TOXICITY STUDY OF AAV9/SURF1 ADMINISTERED BY INTRATHECAL INJECTION IN RATS

Animal: 3522 (Female)

Sacrifice: DAY 29
Group: 3FD29 / AAV9/SURF1 MID DOSE (0.83E12 VG)
Fate: SCHEDULED SACRIFICE / Time on Test: 29 / Cause(s) of Death:

Gross or Microscopic Finding
Disposition
Tissue: Site / Finding, Severity

Disposition: ABNORMAL/FINDING

GANGLION, DORSAL ROOT, LUMBAR
GANGLION, DORSAL ROOT, LUMBAR: INFILTRATE, MONONUCLEAR CELLS, MINIMAL, FOCAL
GANGLION, DORSAL ROOT, LUMBAR, GLIAL CELL: HYPERTROPHY/HYPERPLASIA, MINIMAL
HEART
HEART, MYOCARDIUM: DEGENERATION/NECROSIS, MILD
HEART, MYOCARDIUM: FIBROSIS, MINIMAL
HEART, MYOCARDIUM: INFILTRATE, MONONUCLEAR CELLS, MILD
LIVER
LIVER: INFILTRATE, MIXED, MILD
NERVE, SCIATIC
NERVE, SCIATIC: DEGENERATION, NERVE FIBER, MILD
NERVE, SCIATIC: INFILTRATE, MIXED, MINIMAL
NERVE, TIBIAL
NERVE, TIBIAL: DEGENERATION, NERVE FIBER, MILD
SPINAL CORD, LUMBAR
SPINAL CORD, LUMBAR, WHITE MATTER: DEGENERATION, NERVE FIBER, MINIMAL, DORSAL
SPINAL CORD, THORACIC
SPINAL CORD, THORACIC, WHITE MATTER: DEGENERATION, NERVE FIBER, MINIMAL, DORSAL

Appendix 10

Individual Animal Report

Study ID: 02776-0018: CRL 2954-001 / U of Tex Southwestern Med UTSW.Gray-002
Study Title: A SINGLE DOSE TOXICITY STUDY OF AAV9/SURF1 ADMINISTERED BY INTRATHECAL INJECTION IN RATS

Animal: 3523 (Female)

Sacrifice: DAY 29
Group: 3FD29 / AAV9/SURF1 MID DOSE (0.83E12 VG)
Fate: SCHEDULED SACRIFICE / Time on Test: 29 / Cause(s) of Death:

Gross or Microscopic Finding
Disposition
Tissue: Site / Finding, Severity

GROSS EXAMINATION

Disposition:

TGL #1: LYMPH NODE, AXILLARY; GROSS LESION: ENLARGEMENT; GENERALIZED, BILATERAL // MICROSCOPIC CORRELATION: NO MICROSCOPIC CORRELATE

TGL #2: LYMPH NODE, INGUINAL; GROSS LESION: ENLARGEMENT, GENERALIZED, BILATERAL // MICROSCOPIC CORRELATION: NO MICROSCOPIC CORRELATE

MICROSCOPIC EXAMINATION

Disposition: NORMAL

BRAIN, AMYGDALOID BODY
BRAIN, BASAL NUCLEI/STRIATUM
BRAIN, CEREBELLUM
BRAIN, CEREBRAL CORTEX
BRAIN, HIPPOCAMPUS
BRAIN, HYPOTHALAMUS
BRAIN, MEDULLA OBLONGATA
BRAIN, MENINGES
BRAIN, MIDBRAIN
BRAIN, OLFACTORY BULB
BRAIN, PONS
BRAIN, THALAMUS
BRAIN, VENTRICULAR SYSTEM
BRAIN, WHITE MATTER
EYE
GANGLION, DORSAL ROOT, CERVICAL
KIDNEY
LUNG
LYMPH NODE, ILIAC
LYMPH NODE, MANDIBULAR
LYMPH NODE, MESENTERIC
MUSCLE, BICEPS FEMORIS
MUSCLE, GASTROCNEMIUS
NERVE ROOT, SPINAL, CERVICAL
NERVE ROOT, SPINAL, THORACIC
NERVE, OPTIC
OVARY
PANCREAS

Appendix 10

Individual Animal Report

Study ID: 02776-0018: CRL 2954-001 / U of Tex Southwestern Med UTSW.Gray-002
Study Title: A SINGLE DOSE TOXICITY STUDY OF AAV9/SURF1 ADMINISTERED BY INTRATHECAL INJECTION IN RATS

Animal: 3523 (Female)

Sacrifice: DAY 29
Group: 3FD29 / AAV9/SURF1 MID DOSE (0.83E12 VG)
Fate: SCHEDULED SACRIFICE / Time on Test: 29 / Cause(s) of Death:

Gross or Microscopic Finding
Disposition
Tissue: Site / Finding, Severity

SPINAL CORD, CERVICAL
SPINAL CORD, LUMBAR
SPLEEN
THYMUS

Disposition: ABNORMAL/FINDING

GANGLION, DORSAL ROOT, LUMBAR
GANGLION, DORSAL ROOT, LUMBAR: INFILTRATE, MONONUCLEAR CELLS, MINIMAL
GANGLION, DORSAL ROOT, LUMBAR, GLIAL CELL: HYPERTROPHY/HYPERPLASIA, MINIMAL, MULTIFOCAL
COMMENT(S): FORMING SMALL DISCRETE CLUSTERS CONSISTENT TO WHAT HAS BEEN DESCRIBED AS NAGEOTTE NODULES
GANGLION, DORSAL ROOT, THORACIC
GANGLION, DORSAL ROOT, THORACIC, GLIAL CELL: HYPERTROPHY/HYPERPLASIA, MINIMAL
HEART
HEART, MYOCARDIUM: DEGENERATION/NECROSIS, MINIMAL
HEART, MYOCARDIUM: INFILTRATE, MONONUCLEAR CELLS, MINIMAL
LIVER
LIVER: HYPERTROPHY/HYPERPLASIA, MINIMAL
COMMENT(S): AFFECTING KUPFFER CELLS
LIVER: INFILTRATE, MIXED, MINIMAL
LIVER, HEPATOCYTE: NECROSIS, SINGLE CELL, MINIMAL
NERVE ROOT, SPINAL, LUMBAR
NERVE ROOT, SPINAL, LUMBAR, NERVE ROOT, DORSAL: DEGENERATION, NERVE FIBER, MODERATE
NERVE, SCIATIC
NERVE, SCIATIC: DEGENERATION, NERVE FIBER, MINIMAL
NERVE, TIBIAL
NERVE, TIBIAL: DEGENERATION, NERVE FIBER, MINIMAL
SPINAL CORD, THORACIC
SPINAL CORD, THORACIC, WHITE MATTER: DEGENERATION, NERVE FIBER, MINIMAL
COMMENT(S): SEEN IN LONG SECTION AND EXACT LOCATION COULD NOT BE DETERMINED

Appendix 10

Individual Animal Report

Study ID: 02776-0018: CRL 2954-001 / U of Tex Southwestern Med UTSW.Gray-002
Study Title: A SINGLE DOSE TOXICITY STUDY OF AAV9/SURF1 ADMINISTERED BY INTRATHECAL INJECTION IN RATS

Animal: 3524 (Female)

Sacrifice: DAY 29
Group: 3FD29 / AAV9/SURF1 MID DOSE (0.83E12 VG)
Fate: SCHEDULED SACRIFICE / Time on Test: 29 / Cause(s) of Death:

Gross or Microscopic Finding
Disposition
Tissue: Site / Finding, Severity

MICROSCOPIC EXAMINATION

Disposition: NORMAL

BRAIN, AMYGDALOID BODY
BRAIN, BASAL NUCLEI/STRIATUM
BRAIN, CEREBELLUM
BRAIN, CEREBRAL CORTEX
BRAIN, HIPPOCAMPUS
BRAIN, HYPOTHALAMUS
BRAIN, MEDULLA OBLONGATA
BRAIN, MENINGES
BRAIN, MIDBRAIN
BRAIN, OLFACTORY BULB
BRAIN, PONS
BRAIN, THALAMUS
BRAIN, VENTRICULAR SYSTEM
BRAIN, WHITE MATTER
EYE
GANGLION, DORSAL ROOT, CERVICAL
GANGLION, DORSAL ROOT, THORACIC
HEART
KIDNEY
LUNG
LYMPH NODE, ILIAC
LYMPH NODE, MANDIBULAR
LYMPH NODE, MESENTERIC
MUSCLE, BICEPS FEMORIS
MUSCLE, GASTROCNEMIUS
NERVE ROOT, SPINAL, CERVICAL
NERVE ROOT, SPINAL, THORACIC
OVARY
PANCREAS
SPINAL CORD, CERVICAL
SPINAL CORD, LUMBAR
SPLEEN
THYMUS

Appendix 10

Individual Animal Report

Study ID: 02776-0018: CRL 2954-001 / U of Tex Southwestern Med UTSW.Gray-002
Study Title: A SINGLE DOSE TOXICITY STUDY OF AAV9/SURF1 ADMINISTERED BY INTRATHECAL INJECTION IN RATS

Animal: 3524 (Female)

Sacrifice: DAY 29
Group: 3FD29 / AAV9/SURF1 MID DOSE (0.83E12 VG)
Fate: SCHEDULED SACRIFICE / Time on Test: 29 / Cause(s) of Death:

Gross or Microscopic Finding
Disposition
Tissue: Site / Finding, Severity

Disposition: ABNORMAL/FINDING

GANGLION, DORSAL ROOT, LUMBAR
GANGLION, DORSAL ROOT, LUMBAR: INFILTRATE, MONONUCLEAR CELLS, MINIMAL, MULTIFOCAL
GANGLION, DORSAL ROOT, LUMBAR, GLIAL CELL: HYPERTROPHY/HYPERPLASIA, MINIMAL
LIVER
LIVER: INFILTRATE, MIXED, MINIMAL
NERVE ROOT, SPINAL, LUMBAR
NERVE ROOT, SPINAL, LUMBAR, NERVE ROOT, DORSAL: DEGENERATION, NERVE FIBER, MINIMAL
NERVE, SCIATIC
NERVE, SCIATIC: DEGENERATION, NERVE FIBER, MINIMAL
NERVE, TIBIAL
NERVE, TIBIAL: DEGENERATION, NERVE FIBER, MINIMAL
SPINAL CORD, THORACIC
SPINAL CORD, THORACIC, WHITE MATTER: DEGENERATION, NERVE FIBER, MINIMAL, VENTROLATERAL

Disposition: UNABLE TO OBTAIN SPECIMEN

NERVE, OPTIC

Individual Animal Report

Study ID: 02776-0018: CRL 2954-001 / U of Tex Southwestern Med UTSW.Gray-002
Study Title: A SINGLE DOSE TOXICITY STUDY OF AAV9/SURF1 ADMINISTERED BY INTRATHECAL INJECTION IN RATS

Animal: 3525 (Female)

Sacrifice: DAY 29
Group: 3FD29 / AAV9/SURF1 MID DOSE (0.83E12 VG)
Fate: SCHEDULED SACRIFICE / Time on Test: 29 / Cause(s) of Death:

Gross or Microscopic Finding
Disposition
Tissue: Site / Finding, Severity

MICROSCOPIC EXAMINATION

Disposition: NORMAL

BRAIN, AMYGDALOID BODY
BRAIN, BASAL NUCLEI/STRIATUM
BRAIN, CEREBRAL CORTEX
BRAIN, HIPPOCAMPUS
BRAIN, HYPOTHALAMUS
BRAIN, MIDBRAIN
BRAIN, OLFACTORY BULB
BRAIN, THALAMUS
BRAIN, VENTRICULAR SYSTEM
BRAIN, WHITE MATTER
EYE
GANGLION, DORSAL ROOT, THORACIC
KIDNEY
LUNG
LYMPH NODE, ILIAC
LYMPH NODE, MANDIBULAR
LYMPH NODE, MESENTERIC
MUSCLE, BICEPS FEMORIS
MUSCLE, GASTROCNEMIUS
NERVE ROOT, SPINAL, CERVICAL
NERVE ROOT, SPINAL, THORACIC
NERVE, OPTIC
NERVE, TIBIAL
OVARY
PANCREAS
SPLEEN

Disposition: ABNORMAL/FINDING

BRAIN, CEREBELLUM
BRAIN, CEREBELLUM, WHITE MATTER: DEGENERATION, NERVE FIBER, MILD
BRAIN, MEDULLA OBLONGATA
BRAIN, MEDULLA OBLONGATA, WHITE MATTER: DEGENERATION, NERVE FIBER, MILD
COMMENT(S): INFERIOR CEREBELLAR PEDUNCLE
BRAIN, MENINGES
BRAIN, MENINGES: INFILTRATE, MONONUCLEAR CELLS, MINIMAL, FOCAL

Appendix 10

Individual Animal Report

Study ID: 02776-0018: CRL 2954-001 / U of Tex Southwestern Med UTSW.Gray-002
Study Title: A SINGLE DOSE TOXICITY STUDY OF AAV9/SURF1 ADMINISTERED BY INTRATHECAL INJECTION IN RATS

Animal: 3525 (Female)

Sacrifice: DAY 29
Group: 3FD29 / AAV9/SURF1 MID DOSE (0.83E12 VG)
Fate: SCHEDULED SACRIFICE / Time on Test: 29 / Cause(s) of Death:

Gross or Microscopic Finding
Disposition
Tissue: Site / Finding, Severity

BRAIN, PONS
BRAIN, PONS: DEGENERATION, NERVE FIBER, MINIMAL
COMMENT(S): A SINGLE DIGESTION CHAMBER IS PRESENT
GANGLION, DORSAL ROOT, CERVICAL
GANGLION, DORSAL ROOT, CERVICAL, GLIAL CELL: HYPERTROPHY/HYPERPLASIA, MINIMAL
GANGLION, DORSAL ROOT, LUMBAR
GANGLION, DORSAL ROOT, LUMBAR: INFILTRATE, MONONUCLEAR CELLS, MINIMAL, MULTIFOCAL
HEART
HEART, MYOCARDIUM: DEGENERATION/NECROSIS, MILD
HEART, MYOCARDIUM: INFILTRATE, MONONUCLEAR CELLS, MILD
LIVER
LIVER: INFILTRATE, MIXED, MINIMAL
NERVE ROOT, SPINAL, LUMBAR
NERVE ROOT, SPINAL, LUMBAR, EPINEURIUM: INFILTRATE, MONONUCLEAR CELLS, MINIMAL, FOCAL
NERVE ROOT, SPINAL, LUMBAR, NERVE ROOT, DORSAL: DEGENERATION, NERVE FIBER, MINIMAL
NERVE ROOT, SPINAL, LUMBAR, NERVE ROOT, VENTRAL: DEGENERATION, NERVE FIBER, MODERATE
NERVE, SCIATIC
NERVE, SCIATIC: DEGENERATION, NERVE FIBER, MINIMAL
SPINAL CORD, CERVICAL
SPINAL CORD, CERVICAL, GLIAL CELL: INCREASED CELLULARITY, MINIMAL, FOCAL
COMMENT(S): WITHIN GRAY MATTER
SPINAL CORD, CERVICAL, WHITE MATTER: DEGENERATION, NERVE FIBER, MINIMAL, LATERAL
SPINAL CORD, LUMBAR
SPINAL CORD, LUMBAR, GLIAL CELL: INCREASED CELLULARITY, MILD
COMMENT(S): PREDOMINANTLY WITHIN GRAY MATTER
SPINAL CORD, LUMBAR, GRAY MATTER: DEGENERATION/NECROSIS, MODERATE
COMMENT(S): AFFECTING CELLS WITHIN LATERAL AND VENTRAL HORNS BILATERALLY
SPINAL CORD, LUMBAR, GRAY MATTER: INFILTRATE, MONONUCLEAR CELLS, MINIMAL, MULTIFOCAL, PERIVASCULAR
SPINAL CORD, LUMBAR, WHITE MATTER: DEGENERATION, NERVE FIBER, MODERATE, VENTRAL
COMMENT(S): MODERATE DEGENERATION WITHIN VENTRAL WHITE MATTER TRACTS AND MILD CHANGES WITHIN LATERAL WHITE MATTER TRACTS
SPINAL CORD, THORACIC
SPINAL CORD, THORACIC, GLIAL CELL: INCREASED CELLULARITY, MILD
COMMENT(S): PREDOMINANTLY WITHIN GRAY MATTER
SPINAL CORD, THORACIC, GRAY MATTER: DEGENERATION/NECROSIS, SEVERE
COMMENT(S): PREDOMINANTLY AFFECTING THE LATERAL AND VENTRAL HORNS BILATERALLY
SPINAL CORD, THORACIC, GRAY MATTER: INFILTRATE, MONONUCLEAR CELLS, MINIMAL, MULTIFOCAL, PERIVASCULAR

Appendix 10

Individual Animal Report

Study ID: 02776-0018: CRL 2954-001 / U of Tex Southwestern Med UTSW.Gray-002
Study Title: A SINGLE DOSE TOXICITY STUDY OF AAV9/SURF1 ADMINISTERED BY INTRATHECAL INJECTION IN RATS

Animal: 3525 (Female)

Sacrifice: DAY 29
Group: 3FD29 / AAV9/SURF1 MID DOSE (0.83E12 VG)
Fate: SCHEDULED SACRIFICE / Time on Test: 29 / Cause(s) of Death:

Gross or Microscopic Finding
Disposition
Tissue: Site / Finding, Severity

SPINAL CORD, THORACIC, WHITE MATTER: DEGENERATION, NERVE FIBER, MODERATE, VENTRAL
COMMENT(S): MODERATE DEGENERATION BOTH WITH VENTRAL AND LATERAL WHITE MATTER TRACTS

Disposition: **UNABLE TO OBTAIN SPECIMEN**
THYMUS

Appendix 10

Individual Animal Report

Study ID: 02776-0018: CRL 2954-001 / U of Tex Southwestern Med UTSW.Gray-002
Study Title: A SINGLE DOSE TOXICITY STUDY OF AAV9/SURF1 ADMINISTERED BY INTRATHECAL INJECTION IN RATS

Animal: 4521 (Female)

Sacrifice: DAY 29
Group: 4FD29 / AAV9/SURF1 HIGH DOSE (2.49E12 VG)
Fate: SCHEDULED SACRIFICE / Time on Test: 29 / Cause(s) of Death:

Gross or Microscopic Finding
Disposition
Tissue: Site / Finding, Severity

MICROSCOPIC EXAMINATION

Disposition: NORMAL

BRAIN, AMYGDALOID BODY
BRAIN, BASAL NUCLEI/STRIATUM
BRAIN, CEREBELLUM
BRAIN, CEREBRAL CORTEX
BRAIN, HIPPOCAMPUS
BRAIN, HYPOTHALAMUS
BRAIN, MEDULLA OBLONGATA
BRAIN, MENINGES
BRAIN, MIDBRAIN
BRAIN, OLFACTORY BULB
BRAIN, PONS
BRAIN, THALAMUS
BRAIN, VENTRICULAR SYSTEM
BRAIN, WHITE MATTER
EYE
LUNG
LYMPH NODE, ILIAC
LYMPH NODE, MANDIBULAR
LYMPH NODE, MESENTERIC
MUSCLE, BICEPS FEMORIS
MUSCLE, GASTROCNEMIUS
NERVE ROOT, SPINAL, CERVICAL
NERVE ROOT, SPINAL, THORACIC
NERVE, OPTIC
OVARY
PANCREAS
SPINAL CORD, CERVICAL
SPINAL CORD, THORACIC
SPLEEN
THYMUS

Disposition: ABNORMAL/FINDING

GANGLION, DORSAL ROOT, CERVICAL
GANGLION, DORSAL ROOT, CERVICAL, GLIAL CELL: HYPERTROPHY/HYPERPLASIA, MINIMAL

Appendix 10

Individual Animal Report

Study ID: 02776-0018: CRL 2954-001 / U of Tex Southwestern Med UTSW.Gray-002
Study Title: A SINGLE DOSE TOXICITY STUDY OF AAV9/SURF1 ADMINISTERED BY INTRATHECAL INJECTION IN RATS

Animal: 4521 (Female)

Sacrifice: DAY 29
Group: 4FD29 / AAV9/SURF1 HIGH DOSE (2.49E12 VG)
Fate: SCHEDULED SACRIFICE / Time on Test: 29 / Cause(s) of Death:

Gross or Microscopic Finding
Disposition
Tissue: Site / Finding, Severity

GANGLION, DORSAL ROOT, LUMBAR
GANGLION, DORSAL ROOT, LUMBAR, GLIAL CELL: HYPERTROPHY/HYPERPLASIA, MINIMAL
GANGLION, DORSAL ROOT, THORACIC
GANGLION, DORSAL ROOT, THORACIC, GLIAL CELL: HYPERTROPHY/HYPERPLASIA, MINIMAL
HEART
HEART, MYOCARDIUM: DEGENERATION/NECROSIS, MILD
HEART, MYOCARDIUM: INFILTRATE, MONONUCLEAR CELLS, MILD
KIDNEY
KIDNEY: CHRONIC PROGRESSIVE NEPHROPATHY, MINIMAL
LIVER
LIVER: INFILTRATE, MIXED, MINIMAL
NERVE ROOT, SPINAL, LUMBAR
NERVE ROOT, SPINAL, LUMBAR, EPINEURIUM: INFILTRATE, MONONUCLEAR CELLS, MINIMAL, FOCAL
NERVE, SCIATIC
NERVE, SCIATIC: DEGENERATION, NERVE FIBER, MILD
NERVE, TIBIAL
NERVE, TIBIAL: DEGENERATION, NERVE FIBER, MILD
SPINAL CORD, LUMBAR
SPINAL CORD, LUMBAR, WHITE MATTER: DEGENERATION, NERVE FIBER, MINIMAL, DORSAL

Appendix 10

Individual Animal Report

Study ID: 02776-0018: CRL 2954-001 / U of Tex Southwestern Med UTSW.Gray-002
Study Title: A SINGLE DOSE TOXICITY STUDY OF AAV9/SURF1 ADMINISTERED BY INTRATHECAL INJECTION IN RATS

Animal: 4522 (Female)

Sacrifice: DAY 29
Group: 4FD29 / AAV9/SURF1 HIGH DOSE (2.49E12 VG)
Fate: SCHEDULED SACRIFICE / Time on Test: 29 / Cause(s) of Death:

Gross or Microscopic Finding
Disposition
Tissue: Site / Finding, Severity

MICROSCOPIC EXAMINATION

Disposition: NORMAL

BRAIN, AMYGDALOID BODY
BRAIN, BASAL NUCLEI/STRIATUM
BRAIN, CEREBELLUM
BRAIN, CEREBRAL CORTEX
BRAIN, HIPPOCAMPUS
BRAIN, HYPOTHALAMUS
BRAIN, MEDULLA OBLONGATA
BRAIN, MENINGES
BRAIN, MIDBRAIN
BRAIN, OLFACTORY BULB
BRAIN, PONS
BRAIN, THALAMUS
BRAIN, VENTRICULAR SYSTEM
BRAIN, WHITE MATTER
EYE
GANGLION, DORSAL ROOT, THORACIC
KIDNEY
LUNG
LYMPH NODE, ILIAC
LYMPH NODE, MANDIBULAR
LYMPH NODE, MESENTERIC
MUSCLE, BICEPS FEMORIS
MUSCLE, GASTROCNEMIUS
NERVE ROOT, SPINAL, CERVICAL
NERVE ROOT, SPINAL, THORACIC
NERVE, OPTIC
OVARY
PANCREAS
SPINAL CORD, CERVICAL
SPINAL CORD, LUMBAR
SPLEEN
THYMUS

Appendix 10

Individual Animal Report

Study ID: 02776-0018: CRL 2954-001 / U of Tex Southwestern Med UTSW.Gray-002
Study Title: A SINGLE DOSE TOXICITY STUDY OF AAV9/SURF1 ADMINISTERED BY INTRATHECAL INJECTION IN RATS

Animal: 4522 (Female)

Sacrifice: DAY 29
Group: 4FD29 / AAV9/SURF1 HIGH DOSE (2.49E12 VG)
Fate: SCHEDULED SACRIFICE / Time on Test: 29 / Cause(s) of Death:

Gross or Microscopic Finding
Disposition
Tissue: Site / Finding, Severity

Disposition: **ABNORMAL/FINDING**

GANGLION, DORSAL ROOT, CERVICAL
GANGLION, DORSAL ROOT, CERVICAL, GLIAL CELL: HYPERTROPHY/HYPERPLASIA, MINIMAL
GANGLION, DORSAL ROOT, LUMBAR
GANGLION, DORSAL ROOT, LUMBAR, GLIAL CELL: HYPERTROPHY/HYPERPLASIA, MINIMAL
HEART
HEART, MYOCARDIUM: DEGENERATION/NECROSIS, MINIMAL, MULTIFOCAL
HEART, MYOCARDIUM: INFILTRATE, MONONUCLEAR CELLS, MINIMAL, MULTIFOCAL
LIVER
LIVER: INFILTRATE, MIXED, MINIMAL
NERVE ROOT, SPINAL, LUMBAR
NERVE ROOT, SPINAL, LUMBAR, NERVE ROOT, DORSAL: DEGENERATION, NERVE FIBER, MILD
NERVE, SCIATIC
NERVE, SCIATIC: DEGENERATION, NERVE FIBER, MINIMAL
NERVE, TIBIAL
NERVE, TIBIAL: DEGENERATION, NERVE FIBER, MINIMAL
SPINAL CORD, THORACIC
SPINAL CORD, THORACIC, WHITE MATTER: DEGENERATION, NERVE FIBER, MINIMAL, DORSAL

Appendix 10

Individual Animal Report

Study ID: 02776-0018: CRL 2954-001 / U of Tex Southwestern Med UTSW.Gray-002
Study Title: A SINGLE DOSE TOXICITY STUDY OF AAV9/SURF1 ADMINISTERED BY INTRATHECAL INJECTION IN RATS

Animal: 4523 (Female)

Sacrifice: DAY 29
Group: 4FD29 / AAV9/SURF1 HIGH DOSE (2.49E12 VG)
Fate: SCHEDULED SACRIFICE / Time on Test: 29 / Cause(s) of Death:

Gross or Microscopic Finding
Disposition
Tissue: Site / Finding, Severity

MICROSCOPIC EXAMINATION

Disposition: NORMAL

BRAIN, AMYGDALOID BODY
BRAIN, BASAL NUCLEI/STRIATUM
BRAIN, CEREBELLUM
BRAIN, CEREBRAL CORTEX
BRAIN, HIPPOCAMPUS
BRAIN, HYPOTHALAMUS
BRAIN, MEDULLA OBLONGATA
BRAIN, MIDBRAIN
BRAIN, OLFACTORY BULB
BRAIN, PONS
BRAIN, THALAMUS
BRAIN, VENTRICULAR SYSTEM
BRAIN, WHITE MATTER
EYE
GANGLION, DORSAL ROOT, THORACIC
KIDNEY
LYMPH NODE, ILIAC
LYMPH NODE, MANDIBULAR
LYMPH NODE, MESENTERIC
MUSCLE, BICEPS FEMORIS
MUSCLE, GASTROCNEMIUS
NERVE ROOT, SPINAL, CERVICAL
NERVE ROOT, SPINAL, THORACIC
NERVE, OPTIC
OVARY
SPINAL CORD, CERVICAL
SPLEEN
THYMUS

Disposition: ABNORMAL/FINDING

BRAIN, MENINGES
BRAIN, MENINGES: INFILTRATE, MONONUCLEAR CELLS, MINIMAL, MULTIFOCAL
GANGLION, DORSAL ROOT, CERVICAL
GANGLION, DORSAL ROOT, CERVICAL, GLIAL CELL: HYPERTROPHY/HYPERPLASIA, MINIMAL

Appendix 10

Individual Animal Report

Study ID: 02776-0018: CRL 2954-001 / U of Tex Southwestern Med UTSW.Gray-002
Study Title: A SINGLE DOSE TOXICITY STUDY OF AAV9/SURF1 ADMINISTERED BY INTRATHECAL INJECTION IN RATS

Animal: 4523 (Female)

Sacrifice: DAY 29
Group: 4FD29 / AAV9/SURF1 HIGH DOSE (2.49E12 VG)
Fate: SCHEDULED SACRIFICE / Time on Test: 29 / Cause(s) of Death:

Gross or Microscopic Finding
Disposition
Tissue: Site / Finding, Severity

GANGLION, DORSAL ROOT, LUMBAR
GANGLION, DORSAL ROOT, LUMBAR, GLIAL CELL: HYPERTROPHY/HYPERPLASIA, MINIMAL
HEART
HEART, MYOCARDIUM: DEGENERATION/NECROSIS, MARKED
HEART, MYOCARDIUM: FIBROSIS, MINIMAL
HEART, MYOCARDIUM: INFILTRATE, MONONUCLEAR CELLS, MARKED
LIVER
LIVER: INFILTRATE, MIXED, MINIMAL
LUNG
LUNG: INFLAMMATION, MINIMAL, SUBACUTE, FOCAL
NERVE ROOT, SPINAL, LUMBAR
NERVE ROOT, SPINAL, LUMBAR, EPINEURIUM: INFILTRATE, MONONUCLEAR CELLS, MINIMAL, FOCAL
NERVE, SCIATIC
NERVE, SCIATIC: DEGENERATION, NERVE FIBER, MILD
NERVE, TIBIAL
NERVE, TIBIAL: DEGENERATION, NERVE FIBER, MILD
PANCREAS
PANCREAS: APOPTOSIS/SINGLE CELL NECROSIS, MINIMAL
SPINAL CORD, LUMBAR
SPINAL CORD, LUMBAR, WHITE MATTER: DEGENERATION, NERVE FIBER, MINIMAL, DORSAL
SPINAL CORD, THORACIC
SPINAL CORD, THORACIC, WHITE MATTER: DEGENERATION, NERVE FIBER, MINIMAL, DORSAL

Appendix 10

Individual Animal Report

Study ID: 02776-0018: CRL 2954-001 / U of Tex Southwestern Med UTSW.Gray-002
Study Title: A SINGLE DOSE TOXICITY STUDY OF AAV9/SURF1 ADMINISTERED BY INTRATHECAL INJECTION IN RATS

Animal: 4524 (Female)

Sacrifice: DAY 29
Group: 4FD29 / AAV9/SURF1 HIGH DOSE (2.49E12 VG)
Fate: SCHEDULED SACRIFICE / Time on Test: 29 / Cause(s) of Death:

Gross or Microscopic Finding
Disposition
Tissue: Site / Finding, Severity

MICROSCOPIC EXAMINATION

Disposition: **NORMAL**

BRAIN, AMYGDALOID BODY
BRAIN, BASAL NUCLEI/STRIATUM
BRAIN, CEREBELLUM
BRAIN, CEREBRAL CORTEX
BRAIN, HIPPOCAMPUS
BRAIN, HYPOTHALAMUS
BRAIN, MEDULLA OBLONGATA
BRAIN, MENINGES
BRAIN, OLFACTORY BULB
BRAIN, PONS
BRAIN, THALAMUS
BRAIN, VENTRICULAR SYSTEM
BRAIN, WHITE MATTER
EYE
GANGLION, DORSAL ROOT, THORACIC
LUNG
LYMPH NODE, ILIAC
LYMPH NODE, MANDIBULAR
LYMPH NODE, MESENTERIC
MUSCLE, BICEPS FEMORIS
MUSCLE, GASTROCNEMIUS
NERVE ROOT, SPINAL, CERVICAL
NERVE ROOT, SPINAL, THORACIC
NERVE, OPTIC
OVARY
PANCREAS
SPLEEN
THYMUS

Disposition: **ABNORMAL/FINDING**

BRAIN, MIDBRAIN
BRAIN, MIDBRAIN, PINEAL GLAND: INFILTRATE, MONONUCLEAR CELLS, MILD
GANGLION, DORSAL ROOT, CERVICAL
GANGLION, DORSAL ROOT, CERVICAL, GLIAL CELL: HYPERTROPHY/HYPERPLASIA, MINIMAL

Appendix 10

Individual Animal Report

Study ID: 02776-0018: CRL 2954-001 / U of Tex Southwestern Med UTSW.Gray-002
Study Title: A SINGLE DOSE TOXICITY STUDY OF AAV9/SURF1 ADMINISTERED BY INTRATHECAL INJECTION IN RATS

Animal: 4524 (Female)

Sacrifice: DAY 29
Group: 4FD29 / AAV9/SURF1 HIGH DOSE (2.49E12 VG)
Fate: SCHEDULED SACRIFICE / Time on Test: 29 / Cause(s) of Death:

Gross or Microscopic Finding
Disposition
Tissue: Site / Finding, Severity

GANGLION, DORSAL ROOT, LUMBAR
GANGLION, DORSAL ROOT, LUMBAR: INFILTRATE, MONONUCLEAR CELLS, MINIMAL, MULTIFOCAL
GANGLION, DORSAL ROOT, LUMBAR, GLIAL CELL: HYPERTROPHY/HYPERPLASIA, MINIMAL
HEART
HEART, MYOCARDIUM: DEGENERATION/NECROSIS, MINIMAL
HEART, MYOCARDIUM: INFILTRATE, MONONUCLEAR CELLS, MINIMAL
KIDNEY
KIDNEY: CHRONIC PROGRESSIVE NEPHROPATHY, MINIMAL
LIVER
LIVER: INFILTRATE, MIXED, MINIMAL
NERVE ROOT, SPINAL, LUMBAR
NERVE ROOT, SPINAL, LUMBAR, NERVE ROOT, DORSAL: DEGENERATION, NERVE FIBER, MILD
COMMENT(S): ONE SIDE MINIMALLY AFFECTED
NERVE, SCIATIC
NERVE, SCIATIC: DEGENERATION, NERVE FIBER, MODERATE
NERVE, TIBIAL
NERVE, TIBIAL: DEGENERATION, NERVE FIBER, MODERATE
SPINAL CORD, CERVICAL
SPINAL CORD, CERVICAL, MENINGES: INFILTRATE, MONONUCLEAR CELLS, MINIMAL, MULTIFOCAL
SPINAL CORD, CERVICAL, WHITE MATTER: DEGENERATION, NERVE FIBER, MINIMAL
COMMENT(S): SEEN IN LONG SECTION AND EXACT LOCATION COULD NOT BE DETERMINED
SPINAL CORD, LUMBAR
SPINAL CORD, LUMBAR, WHITE MATTER: DEGENERATION, NERVE FIBER, MINIMAL, DORSAL
SPINAL CORD, THORACIC
SPINAL CORD, THORACIC, WHITE MATTER: DEGENERATION, NERVE FIBER, MINIMAL, DORSAL

Appendix 10

Individual Animal Report

Study ID: 02776-0018: CRL 2954-001 / U of Tex Southwestern Med UTSW.Gray-002
Study Title: A SINGLE DOSE TOXICITY STUDY OF AAV9/SURF1 ADMINISTERED BY INTRATHECAL INJECTION IN RATS

Animal: 4525 (Female)

Sacrifice: DAY 29
Group: 4FD29 / AAV9/SURF1 HIGH DOSE (2.49E12 VG)
Fate: SCHEDULED SACRIFICE / Time on Test: 29 / Cause(s) of Death:

Gross or Microscopic Finding
Disposition
Tissue: Site / Finding, Severity

MICROSCOPIC EXAMINATION

Disposition: NORMAL

BRAIN, AMYGDALOID BODY
BRAIN, BASAL NUCLEI/STRIATUM
BRAIN, CEREBELLUM
BRAIN, CEREBRAL CORTEX
BRAIN, HIPPOCAMPUS
BRAIN, HYPOTHALAMUS
BRAIN, MEDULLA OBLONGATA
BRAIN, MENINGES
BRAIN, MIDBRAIN
BRAIN, OLFACTORY BULB
BRAIN, PONS
BRAIN, THALAMUS
BRAIN, VENTRICULAR SYSTEM
BRAIN, WHITE MATTER
EYE
GANGLION, DORSAL ROOT, THORACIC
HEART
KIDNEY
LUNG
LYMPH NODE, ILIAC
LYMPH NODE, MANDIBULAR
LYMPH NODE, MESENTERIC
MUSCLE, BICEPS FEMORIS
MUSCLE, GASTROCNEMIUS
NERVE ROOT, SPINAL, CERVICAL
NERVE ROOT, SPINAL, LUMBAR
NERVE ROOT, SPINAL, THORACIC
NERVE, OPTIC
NERVE, SCIATIC
NERVE, TIBIAL
OVARY
PANCREAS
SPINAL CORD, CERVICAL
SPINAL CORD, LUMBAR

Appendix 10

Individual Animal Report

Study ID: 02776-0018: CRL 2954-001 / U of Tex Southwestern Med UTSW.Gray-002
Study Title: A SINGLE DOSE TOXICITY STUDY OF AAV9/SURF1 ADMINISTERED BY INTRATHECAL INJECTION IN RATS

Animal: 4525 (Female)

Sacrifice: DAY 29
Group: 4FD29 / AAV9/SURF1 HIGH DOSE (2.49E12 VG)
Fate: SCHEDULED SACRIFICE / Time on Test: 29 / Cause(s) of Death:

Gross or Microscopic Finding
Disposition
Tissue: Site / Finding, Severity

SPINAL CORD, THORACIC
SPLEEN
THYMUS

Disposition: **ABNORMAL/FINDING**

GANGLION, DORSAL ROOT, CERVICAL
GANGLION, DORSAL ROOT, CERVICAL, GLIAL CELL: HYPERTROPHY/HYPERPLASIA, MINIMAL
GANGLION, DORSAL ROOT, LUMBAR
GANGLION, DORSAL ROOT, LUMBAR, GLIAL CELL: HYPERTROPHY/HYPERPLASIA, MINIMAL
LIVER
LIVER: INFILTRATE, MIXED, MILD

Appendix 10

Individual Animal Report

Study ID: 02776-0018: CRL 2954-001 / U of Tex Southwestern Med UTSW.Gray-002
Study Title: A SINGLE DOSE TOXICITY STUDY OF AAV9/SURF1 ADMINISTERED BY INTRATHECAL INJECTION IN RATS

Animal: 1006 (Male)

Sacrifice: DAY 91
Group: 1MD91 / VEHICLE (0 VG)
Fate: SCHEDULED SACRIFICE / Time on Test: 87 / Cause(s) of Death:

Gross or Microscopic Finding
Disposition
Tissue: Site / Finding, Severity

MICROSCOPIC EXAMINATION

Disposition: NORMAL

BRAIN, AMYGDALOID BODY
BRAIN, BASAL NUCLEI/STRIATUM
BRAIN, CEREBELLUM
BRAIN, CEREBRAL CORTEX
BRAIN, HIPPOCAMPUS
BRAIN, HYPOTHALAMUS
BRAIN, MEDULLA OBLONGATA
BRAIN, MENINGES
BRAIN, MIDBRAIN
BRAIN, OLFACTORY BULB
BRAIN, PONS
BRAIN, THALAMUS
BRAIN, VENTRICULAR SYSTEM
BRAIN, WHITE MATTER
GANGLION, DORSAL ROOT, CERVICAL
GANGLION, DORSAL ROOT, THORACIC
HEART
KIDNEY
LIVER
LUNG
LYMPH NODE, ILIAC
LYMPH NODE, MANDIBULAR
LYMPH NODE, MESENTERIC
MUSCLE, BICEPS FEMORIS
MUSCLE, GASTROCNEMIUS
NERVE ROOT, SPINAL, CERVICAL
NERVE ROOT, SPINAL, LUMBAR
NERVE ROOT, SPINAL, THORACIC
NERVE, SCIATIC
NERVE, TIBIAL
PANCREAS
SPINAL CORD, CERVICAL
SPINAL CORD, THORACIC
SPLEEN

Appendix 10

Individual Animal Report

Study ID: 02776-0018: CRL 2954-001 / U of Tex Southwestern Med UTSW.Gray-002
Study Title: A SINGLE DOSE TOXICITY STUDY OF AAV9/SURF1 ADMINISTERED BY INTRATHECAL INJECTION IN RATS

Animal: 1006 (Male)

Sacrifice: DAY 91
Group: 1MD91 / VEHICLE (0 VG)
Fate: SCHEDULED SACRIFICE / Time on Test: 87 / Cause(s) of Death:

Gross or Microscopic Finding
Disposition
Tissue: Site / Finding, Severity

TESTIS
THYMUS

Disposition: **ABNORMAL/FINDING**

GANGLION, DORSAL ROOT, LUMBAR
GANGLION, DORSAL ROOT, LUMBAR, GLIAL CELL: HYPERTROPHY/HYPERPLASIA, MINIMAL
SPINAL CORD, LUMBAR
SPINAL CORD, LUMBAR, WHITE MATTER: DEGENERATION, NERVE FIBER, MINIMAL, VENTRAL

Disposition: **NOT PRESENT AT TRIM**

EYE
NERVE, OPTIC

Appendix 10

Individual Animal Report

Study ID: 02776-0018: CRL 2954-001 / U of Tex Southwestern Med UTSW.Gray-002
Study Title: A SINGLE DOSE TOXICITY STUDY OF AAV9/SURF1 ADMINISTERED BY INTRATHECAL INJECTION IN RATS

Animal: 1007 (Male)

Sacrifice: DAY 91
Group: 1MD91 / VEHICLE (0 VG)
Fate: SCHEDULED SACRIFICE / Time on Test: 87 / Cause(s) of Death:

Gross or Microscopic Finding
Disposition
Tissue: Site / Finding, Severity

MICROSCOPIC EXAMINATION

Disposition: NORMAL

BRAIN, AMYGDALOID BODY
BRAIN, BASAL NUCLEI/STRIATUM
BRAIN, CEREBELLUM
BRAIN, CEREBRAL CORTEX
BRAIN, HIPPOCAMPUS
BRAIN, HYPOTHALAMUS
BRAIN, MEDULLA OBLONGATA
BRAIN, MENINGES
BRAIN, MIDBRAIN
BRAIN, OLFACTORY BULB
BRAIN, PONS
BRAIN, THALAMUS
BRAIN, VENTRICULAR SYSTEM
BRAIN, WHITE MATTER
GANGLION, DORSAL ROOT, CERVICAL
LUNG
LYMPH NODE, ILIAC
LYMPH NODE, MANDIBULAR
LYMPH NODE, MESENTERIC
MUSCLE, BICEPS FEMORIS
MUSCLE, GASTROCNEMIUS
NERVE ROOT, SPINAL, CERVICAL
NERVE ROOT, SPINAL, LUMBAR
NERVE ROOT, SPINAL, THORACIC
NERVE, OPTIC
NERVE, SCIATIC
NERVE, TIBIAL
PANCREAS
SPINAL CORD, CERVICAL
SPLEEN
TESTIS
THYMUS

Appendix 10

Individual Animal Report

Study ID: 02776-0018: CRL 2954-001 / U of Tex Southwestern Med UTSW.Gray-002
Study Title: A SINGLE DOSE TOXICITY STUDY OF AAV9/SURF1 ADMINISTERED BY INTRATHECAL INJECTION IN RATS

Animal: 1007 (Male)

Sacrifice: DAY 91
Group: 1MD91 / VEHICLE (0 VG)
Fate: SCHEDULED SACRIFICE / Time on Test: 87 / Cause(s) of Death:

Gross or Microscopic Finding
Disposition
Tissue: Site / Finding, Severity

Disposition: ABNORMAL/FINDING

GANGLION, DORSAL ROOT, LUMBAR
GANGLION, DORSAL ROOT, LUMBAR, GLIAL CELL: HYPERTROPHY/HYPERPLASIA, MINIMAL
GANGLION, DORSAL ROOT, THORACIC
GANGLION, DORSAL ROOT, THORACIC: INFILTRATE, MONONUCLEAR CELLS, MINIMAL, FOCAL
HEART
HEART, MYOCARDIUM: DEGENERATION/NECROSIS, MINIMAL, FOCAL
HEART, MYOCARDIUM: FIBROSIS, MINIMAL, FOCAL
HEART, MYOCARDIUM: INFILTRATE, MONONUCLEAR CELLS, MINIMAL, FOCAL
KIDNEY
KIDNEY: CHRONIC PROGRESSIVE NEPHROPATHY, MINIMAL
LIVER
LIVER: INFILTRATE, MIXED, MINIMAL
LIVER: NECROSIS, MINIMAL, FOCAL
LIVER, HEPATOCYTE: VACUOLATION, MINIMAL, LOCALLY EXTENSIVE
COMMENT(S): CONSISTENT WITH TENSION LIPIDOSIS
SPINAL CORD, LUMBAR
SPINAL CORD, LUMBAR, NERVE ROOT, SPINAL: DEGENERATION, NERVE FIBER, MINIMAL
SPINAL CORD, THORACIC
SPINAL CORD, THORACIC, WHITE MATTER: DEGENERATION, NERVE FIBER, MINIMAL
COMMENT(S): SEEN IN LONG SECTION AND EXACT LOCATION COULD NOT BE DETERMINED

Disposition: UNABLE TO OBTAIN SPECIMEN

EYE

Appendix 10

Individual Animal Report

Study ID: 02776-0018: CRL 2954-001 / U of Tex Southwestern Med UTSW.Gray-002
Study Title: A SINGLE DOSE TOXICITY STUDY OF AAV9/SURF1 ADMINISTERED BY INTRATHECAL INJECTION IN RATS

Animal: 1008 (Male)

Sacrifice: DAY 91
Group: 1MD91 / VEHICLE (0 VG)
Fate: SCHEDULED SACRIFICE / Time on Test: 87 / Cause(s) of Death:

Gross or Microscopic Finding
Disposition
Tissue: Site / Finding, Severity

MICROSCOPIC EXAMINATION

Disposition: NORMAL

BRAIN, AMYGDALOID BODY
BRAIN, BASAL NUCLEI/STRIATUM
BRAIN, CEREBELLUM
BRAIN, CEREBRAL CORTEX
BRAIN, HIPPOCAMPUS
BRAIN, HYPOTHALAMUS
BRAIN, MEDULLA OBLONGATA
BRAIN, MENINGES
BRAIN, MIDBRAIN
BRAIN, OLFACTORY BULB
BRAIN, PONS
BRAIN, THALAMUS
BRAIN, VENTRICULAR SYSTEM
BRAIN, WHITE MATTER
GANGLION, DORSAL ROOT, CERVICAL
GANGLION, DORSAL ROOT, THORACIC
LIVER
LYMPH NODE, MANDIBULAR
LYMPH NODE, MESENTERIC
MUSCLE, BICEPS FEMORIS
MUSCLE, GASTROCNEMIUS
NERVE ROOT, SPINAL, CERVICAL
NERVE ROOT, SPINAL, LUMBAR
NERVE ROOT, SPINAL, THORACIC
NERVE, OPTIC
NERVE, TIBIAL
SPINAL CORD, CERVICAL
SPINAL CORD, LUMBAR
SPINAL CORD, THORACIC
SPLEEN
TESTIS

Disposition: ABNORMAL/FINDING

GANGLION, DORSAL ROOT, LUMBAR

Appendix 10

Individual Animal Report

Study ID: 02776-0018: CRL 2954-001 / U of Tex Southwestern Med UTSW.Gray-002
Study Title: A SINGLE DOSE TOXICITY STUDY OF AAV9/SURF1 ADMINISTERED BY INTRATHECAL INJECTION IN RATS

Animal: 1008 (Male)

Sacrifice: DAY 91
Group: 1MD91 / VEHICLE (0 VG)
Fate: SCHEDULED SACRIFICE / Time on Test: 87 / Cause(s) of Death:

Gross or Microscopic Finding
Disposition
Tissue: Site / Finding, Severity

GANGLION, DORSAL ROOT, LUMBAR, GLIAL CELL: HYPERTROPHY/HYPERPLASIA, MINIMAL
HEART
HEART, MYOCARDIUM: DEGENERATION/NECROSIS, MINIMAL, FOCAL
HEART, MYOCARDIUM: INFILTRATE, MONONUCLEAR CELLS, MINIMAL, FOCAL
KIDNEY
KIDNEY, TUBULAR: ACCUMULATION, HYALINE DROPLETS, MINIMAL
COMMENT(S): HYALINE DROPLETS
LUNG
LUNG, ALVEOLUS: INFILTRATE, MACROPHAGES, MINIMAL, FOCAL
NERVE, SCIATIC
NERVE, SCIATIC: DEGENERATION, NERVE FIBER, MINIMAL
PANCREAS
PANCREAS: INFILTRATE, MONONUCLEAR CELLS, MINIMAL, FOCAL
THYMUS
THYMUS: INCREASED CELLULARITY, MINIMAL
COMMENT(S): AFFECTING THYMIC EPITHELIAL CELLS

Disposition: UNABLE TO OBTAIN SPECIMEN
EYE

Disposition: NOT PRESENT AT TRIM
LYMPH NODE, ILIAC

Appendix 10

Individual Animal Report

Study ID: 02776-0018: CRL 2954-001 / U of Tex Southwestern Med UTSW.Gray-002
Study Title: A SINGLE DOSE TOXICITY STUDY OF AAV9/SURF1 ADMINISTERED BY INTRATHECAL INJECTION IN RATS

Animal: 1009 (Male)

Sacrifice: DAY 91
Group: 1MD91 / VEHICLE (0 VG)
Fate: SCHEDULED SACRIFICE / Time on Test: 91 / Cause(s) of Death:

Gross or Microscopic Finding
Disposition
Tissue: Site / Finding, Severity

MICROSCOPIC EXAMINATION

Disposition: NORMAL

BRAIN, AMYGDALOID BODY
BRAIN, BASAL NUCLEI/STRIATUM
BRAIN, CEREBELLUM
BRAIN, CEREBRAL CORTEX
BRAIN, HIPPOCAMPUS
BRAIN, HYPOTHALAMUS
BRAIN, MENINGES
BRAIN, MIDBRAIN
BRAIN, OLFACTORY BULB
BRAIN, PONS
BRAIN, THALAMUS
BRAIN, VENTRICULAR SYSTEM
BRAIN, WHITE MATTER
EYE
GANGLION, DORSAL ROOT, CERVICAL
GANGLION, DORSAL ROOT, LUMBAR
GANGLION, DORSAL ROOT, THORACIC
KIDNEY
LUNG
LYMPH NODE, ILIAC
LYMPH NODE, MANDIBULAR
LYMPH NODE, MESENTERIC
MUSCLE, BICEPS FEMORIS
MUSCLE, GASTROCNEMIUS
NERVE ROOT, SPINAL, CERVICAL
NERVE ROOT, SPINAL, LUMBAR
NERVE ROOT, SPINAL, THORACIC
NERVE, OPTIC
NERVE, SCIATIC
NERVE, TIBIAL
PANCREAS
SPINAL CORD, CERVICAL
SPINAL CORD, LUMBAR
SPLEEN

Appendix 10

Individual Animal Report

Study ID: 02776-0018: CRL 2954-001 / U of Tex Southwestern Med UTSW.Gray-002
Study Title: A SINGLE DOSE TOXICITY STUDY OF AAV9/SURF1 ADMINISTERED BY INTRATHECAL INJECTION IN RATS

Animal: 1009 (Male)

Sacrifice: DAY 91
Group: 1MD91 / VEHICLE (0 VG)
Fate: SCHEDULED SACRIFICE / Time on Test: 91 / Cause(s) of Death:

Gross or Microscopic Finding
Disposition
Tissue: Site / Finding, Severity

TESTIS
THYMUS

Disposition: **ABNORMAL/FINDING**

BRAIN, MEDULLA OBLONGATA
BRAIN, MEDULLA OBLONGATA, WHITE MATTER: DEGENERATION, NERVE FIBER, MINIMAL
COMMENT(S): PYRAMIDAL TRACT

HEART
HEART, MYOCARDIUM: DEGENERATION/NECROSIS, MINIMAL, FOCAL
HEART, MYOCARDIUM: INFILTRATE, MONONUCLEAR CELLS, MINIMAL, FOCAL

LIVER
LIVER: INFILTRATE, MIXED, MINIMAL
LIVER: NECROSIS, MINIMAL, FOCAL

SPINAL CORD, THORACIC
SPINAL CORD, THORACIC, WHITE MATTER: DEGENERATION, NERVE FIBER, MINIMAL
COMMENT(S): SEEN IN LONG SECTION AND EXACT LOCATION COULD NOT BE DETERMINED

Appendix 10

Individual Animal Report

Study ID: 02776-0018: CRL 2954-001 / U of Tex Southwestern Med UTSW.Gray-002
Study Title: A SINGLE DOSE TOXICITY STUDY OF AAV9/SURF1 ADMINISTERED BY INTRATHECAL INJECTION IN RATS

Animal: 1010 (Male)

Sacrifice: DAY 91
Group: 1MD91 / VEHICLE (0 VG)
Fate: SCHEDULED SACRIFICE / Time on Test: 91 / Cause(s) of Death:

Gross or Microscopic Finding
Disposition
Tissue: Site / Finding, Severity

MICROSCOPIC EXAMINATION

Disposition: NORMAL

BRAIN, AMYGDALOID BODY
BRAIN, BASAL NUCLEI/STRIATUM
BRAIN, CEREBELLUM
BRAIN, CEREBRAL CORTEX
BRAIN, HIPPOCAMPUS
BRAIN, HYPOTHALAMUS
BRAIN, MEDULLA OBLONGATA
BRAIN, MENINGES
BRAIN, MIDBRAIN
BRAIN, OLFACTORY BULB
BRAIN, PONS
BRAIN, THALAMUS
BRAIN, VENTRICULAR SYSTEM
BRAIN, WHITE MATTER
EYE
GANGLION, DORSAL ROOT, CERVICAL
GANGLION, DORSAL ROOT, LUMBAR
GANGLION, DORSAL ROOT, THORACIC
LUNG
LYMPH NODE, ILIAC
LYMPH NODE, MESENTERIC
MUSCLE, BICEPS FEMORIS
MUSCLE, GASTROCNEMIUS
NERVE ROOT, SPINAL, CERVICAL
NERVE ROOT, SPINAL, LUMBAR
NERVE ROOT, SPINAL, THORACIC
NERVE, OPTIC
NERVE, SCIATIC
NERVE, TIBIAL
SPINAL CORD, CERVICAL
SPINAL CORD, LUMBAR
SPINAL CORD, THORACIC
SPLEEN
TESTIS

Appendix 10

Individual Animal Report

Study ID: 02776-0018: CRL 2954-001 / U of Tex Southwestern Med UTSW.Gray-002
Study Title: A SINGLE DOSE TOXICITY STUDY OF AAV9/SURF1 ADMINISTERED BY INTRATHECAL INJECTION IN RATS

Animal: 1010 (Male)

Sacrifice: DAY 91
Group: 1MD91 / VEHICLE (0 VG)
Fate: SCHEDULED SACRIFICE / Time on Test: 91 / Cause(s) of Death:

Gross or Microscopic Finding
Disposition
Tissue: Site / Finding, Severity

THYMUS

Disposition: ABNORMAL/FINDING

HEART
HEART, MYOCARDIUM: DEGENERATION/NECROSIS, MINIMAL, FOCAL
HEART, MYOCARDIUM: INFILTRATE, MONONUCLEAR CELLS, MINIMAL, FOCAL
KIDNEY
KIDNEY, TUBULAR: DILATATION, MINIMAL, FOCAL
LIVER
LIVER: INFILTRATE, MIXED, MINIMAL
PANCREAS
PANCREAS: FIBROSIS, MINIMAL, FOCAL
COMMENT(S): AFFECTING ISLETS OF LANGERHANS

Disposition: UNABLE TO OBTAIN SPECIMEN

LYMPH NODE, MANDIBULAR

Appendix 10

Individual Animal Report

Study ID: 02776-0018: CRL 2954-001 / U of Tex Southwestern Med UTSW.Gray-002
Study Title: A SINGLE DOSE TOXICITY STUDY OF AAV9/SURF1 ADMINISTERED BY INTRATHECAL INJECTION IN RATS

Animal: 2006 (Male)

Sacrifice: DAY 91
Group: 2MD91 / AAV9/SURF1 LOW DOSE (0.28E12 VG)
Fate: SCHEDULED SACRIFICE / Time on Test: 87 / Cause(s) of Death:

Gross or Microscopic Finding
Disposition
Tissue: Site / Finding, Severity

MICROSCOPIC EXAMINATION

Disposition: NORMAL

BRAIN, AMYGDALOID BODY
BRAIN, BASAL NUCLEI/STRIATUM
BRAIN, CEREBELLUM
BRAIN, CEREBRAL CORTEX
BRAIN, HIPPOCAMPUS
BRAIN, HYPOTHALAMUS
BRAIN, MEDULLA OBLONGATA
BRAIN, MENINGES
BRAIN, MIDBRAIN
BRAIN, OLFACTORY BULB
BRAIN, PONS
BRAIN, THALAMUS
BRAIN, VENTRICULAR SYSTEM
BRAIN, WHITE MATTER
EYE
GANGLION, DORSAL ROOT, CERVICAL
GANGLION, DORSAL ROOT, LUMBAR
GANGLION, DORSAL ROOT, THORACIC
LUNG
LYMPH NODE, ILIAC
LYMPH NODE, MANDIBULAR
LYMPH NODE, MESENTERIC
MUSCLE, BICEPS FEMORIS
MUSCLE, GASTROCNEMIUS
NERVE ROOT, SPINAL, CERVICAL
NERVE ROOT, SPINAL, LUMBAR
NERVE ROOT, SPINAL, THORACIC
NERVE, OPTIC
NERVE, SCIATIC
NERVE, TIBIAL
PANCREAS
SPLEEN
TESTIS
THYMUS

Appendix 10

Individual Animal Report

Study ID: 02776-0018: CRL 2954-001 / U of Tex Southwestern Med UTSW.Gray-002
Study Title: A SINGLE DOSE TOXICITY STUDY OF AAV9/SURF1 ADMINISTERED BY INTRATHECAL INJECTION IN RATS

Animal: 2006 (Male)

Sacrifice: DAY 91
Group: 2MD91 / AAV9/SURF1 LOW DOSE (0.28E12 VG)
Fate: SCHEDULED SACRIFICE / Time on Test: 87 / Cause(s) of Death:

Gross or Microscopic Finding
Disposition
Tissue: Site / Finding, Severity

Disposition: **ABNORMAL/FINDING**

HEART
HEART, MYOCARDIUM: DEGENERATION/NECROSIS, MINIMAL, MULTIFOCAL
HEART, MYOCARDIUM: FIBROSIS, MINIMAL, MULTIFOCAL
HEART, MYOCARDIUM: INFILTRATE, MONONUCLEAR CELLS, MINIMAL, MULTIFOCAL
KIDNEY
KIDNEY, TUBULAR: DILATATION, MINIMAL, FOCAL
LIVER
LIVER: INFILTRATE, MIXED, MINIMAL
SPINAL CORD, CERVICAL
SPINAL CORD, CERVICAL, WHITE MATTER: DEGENERATION, NERVE FIBER, MINIMAL
COMMENT(S): SEEN IN LONG SECTION AND EXACT LOCATION COULD NOT BE DETERMINED
SPINAL CORD, LUMBAR
SPINAL CORD, LUMBAR, WHITE MATTER: DEGENERATION, NERVE FIBER, MINIMAL, DORSAL
SPINAL CORD, THORACIC
SPINAL CORD, THORACIC, GLIAL CELL: INCREASED CELLULARITY, MINIMAL, FOCAL, VENTRAL

Appendix 10

Individual Animal Report

Study ID: 02776-0018: CRL 2954-001 / U of Tex Southwestern Med UTSW.Gray-002
Study Title: A SINGLE DOSE TOXICITY STUDY OF AAV9/SURF1 ADMINISTERED BY INTRATHECAL INJECTION IN RATS

Animal: 2007 (Male)

Sacrifice: DAY 91
Group: 2MD91 / AAV9/SURF1 LOW DOSE (0.28E12 VG)
Fate: SCHEDULED SACRIFICE / Time on Test: 87 / Cause(s) of Death:

Gross or Microscopic Finding
Disposition
Tissue: Site / Finding, Severity

MICROSCOPIC EXAMINATION

Disposition: NORMAL

BRAIN, AMYGDALOID BODY
BRAIN, BASAL NUCLEI/STRIATUM
BRAIN, CEREBELLUM
BRAIN, CEREBRAL CORTEX
BRAIN, HIPPOCAMPUS
BRAIN, HYPOTHALAMUS
BRAIN, MEDULLA OBLONGATA
BRAIN, MENINGES
BRAIN, MIDBRAIN
BRAIN, OLFACTORY BULB
BRAIN, PONS
BRAIN, THALAMUS
BRAIN, VENTRICULAR SYSTEM
BRAIN, WHITE MATTER
GANGLION, DORSAL ROOT, CERVICAL
GANGLION, DORSAL ROOT, THORACIC
LUNG
LYMPH NODE, ILIAC
LYMPH NODE, MANDIBULAR
LYMPH NODE, MESENTERIC
MUSCLE, BICEPS FEMORIS
MUSCLE, GASTROCNEMIUS
NERVE ROOT, SPINAL, CERVICAL
NERVE ROOT, SPINAL, THORACIC
PANCREAS
SPINAL CORD, LUMBAR
SPLEEN
TESTIS
THYMUS

Disposition: ABNORMAL/FINDING

GANGLION, DORSAL ROOT, LUMBAR
GANGLION, DORSAL ROOT, LUMBAR: INFILTRATE, MONONUCLEAR CELLS, MILD, MULTIFOCAL
COMMENT(S): MAY HAVE MINIMAL DECREASED NEURON CELLULARITY BUT CANNOT DEFINITELY DETERMINE DUE TO SOME PROCESSING/SECTIONING ARTIFACTS

Appendix 10

Individual Animal Report

Study ID: 02776-0018: CRL 2954-001 / U of Tex Southwestern Med UTSW.Gray-002
Study Title: A SINGLE DOSE TOXICITY STUDY OF AAV9/SURF1 ADMINISTERED BY INTRATHECAL INJECTION IN RATS

Animal: 2007 (Male)

Sacrifice: DAY 91
Group: 2MD91 / AAV9/SURF1 LOW DOSE (0.28E12 VG)
Fate: SCHEDULED SACRIFICE / Time on Test: 87 / Cause(s) of Death:

Gross or Microscopic Finding
Disposition
Tissue: Site / Finding, Severity

GANGLION, DORSAL ROOT, LUMBAR, GLIAL CELL: HYPERTROPHY/HYPERPLASIA, MINIMAL
HEART
HEART, MYOCARDIUM: DEGENERATION/NECROSIS, MINIMAL, FOCAL
HEART, MYOCARDIUM: FIBROSIS, MINIMAL, FOCAL
HEART, MYOCARDIUM: INFILTRATE, MONONUCLEAR CELLS, MINIMAL, MULTIFOCAL
KIDNEY
KIDNEY: CHRONIC PROGRESSIVE NEPHROPATHY, MINIMAL
LIVER
LIVER: INFILTRATE, MIXED, MINIMAL
NERVE ROOT, SPINAL, LUMBAR
NERVE ROOT, SPINAL, LUMBAR, NERVE ROOT, DORSAL: DEGENERATION, NERVE FIBER, MILD
NERVE, SCIATIC
NERVE, SCIATIC: DEGENERATION, NERVE FIBER, MINIMAL
NERVE, TIBIAL
NERVE, TIBIAL: DEGENERATION, NERVE FIBER, MINIMAL
SPINAL CORD, CERVICAL
SPINAL CORD, CERVICAL, WHITE MATTER: DEGENERATION, NERVE FIBER, MINIMAL, VENTROLATERAL
SPINAL CORD, THORACIC
SPINAL CORD, THORACIC, MENINGES: INFILTRATE, MONONUCLEAR CELLS, MINIMAL, FOCAL
SPINAL CORD, THORACIC, NERVE ROOT, SPINAL: DEGENERATION, NERVE FIBER, MILD

Disposition: NOT PRESENT AT TRIM
EYE
NERVE, OPTIC

Appendix 10

Individual Animal Report

Study ID: 02776-0018: CRL 2954-001 / U of Tex Southwestern Med UTSW.Gray-002
Study Title: A SINGLE DOSE TOXICITY STUDY OF AAV9/SURF1 ADMINISTERED BY INTRATHECAL INJECTION IN RATS

Animal: 2008 (Male)

Sacrifice: DAY 91
Group: 2MD91 / AAV9/SURF1 LOW DOSE (0.28E12 VG)
Fate: SCHEDULED SACRIFICE / Time on Test: 87 / Cause(s) of Death:

Gross or Microscopic Finding
Disposition
Tissue: Site / Finding, Severity

MICROSCOPIC EXAMINATION

Disposition: NORMAL

BRAIN, AMYGDALOID BODY
BRAIN, BASAL NUCLEI/STRIATUM
BRAIN, CEREBELLUM
BRAIN, CEREBRAL CORTEX
BRAIN, HIPPOCAMPUS
BRAIN, HYPOTHALAMUS
BRAIN, MENINGES
BRAIN, OLFACTORY BULB
BRAIN, PONS
BRAIN, THALAMUS
BRAIN, VENTRICULAR SYSTEM
BRAIN, WHITE MATTER
EYE
GANGLION, DORSAL ROOT, LUMBAR
GANGLION, DORSAL ROOT, THORACIC
KIDNEY
LUNG
LYMPH NODE, ILIAC
LYMPH NODE, MANDIBULAR
LYMPH NODE, MESENTERIC
MUSCLE, BICEPS FEMORIS
MUSCLE, GASTROCNEMIUS
NERVE ROOT, SPINAL, CERVICAL
NERVE ROOT, SPINAL, THORACIC
NERVE, OPTIC
NERVE, TIBIAL
PANCREAS
SPINAL CORD, CERVICAL
SPINAL CORD, LUMBAR
SPINAL CORD, THORACIC
SPLEEN
TESTIS
THYMUS

Appendix 10

Individual Animal Report

Study ID: 02776-0018: CRL 2954-001 / U of Tex Southwestern Med UTSW.Gray-002
Study Title: A SINGLE DOSE TOXICITY STUDY OF AAV9/SURF1 ADMINISTERED BY INTRATHECAL INJECTION IN RATS

Animal: 2008 (Male)

Sacrifice: DAY 91
Group: 2MD91 / AAV9/SURF1 LOW DOSE (0.28E12 VG)
Fate: SCHEDULED SACRIFICE / Time on Test: 87 / Cause(s) of Death:

Gross or Microscopic Finding
Disposition
Tissue: Site / Finding, Severity

Disposition: ABNORMAL/FINDING

BRAIN, MEDULLA OBLONGATA
BRAIN, MEDULLA OBLONGATA, WHITE MATTER: DEGENERATION, NERVE FIBER, MINIMAL
COMMENT(S): PYRAMIDAL TRACT

BRAIN, MIDBRAIN
BRAIN, MIDBRAIN, PINEAL GLAND: VACUOLATION, MODERATE

GANGLION, DORSAL ROOT, CERVICAL
GANGLION, DORSAL ROOT, CERVICAL, GLIAL CELL: HYPERTROPHY/HYPERPLASIA, MINIMAL

HEART
HEART, MYOCARDIUM: DEGENERATION/NECROSIS, MINIMAL, MULTIFOCAL
HEART, MYOCARDIUM: INFILTRATE, MONONUCLEAR CELLS, MINIMAL, MULTIFOCAL

LIVER
LIVER: INFILTRATE, MIXED, MINIMAL

NERVE ROOT, SPINAL, LUMBAR
NERVE ROOT, SPINAL, LUMBAR, NERVE ROOT, DORSAL: DEGENERATION, NERVE FIBER, MINIMAL

NERVE, SCIATIC
NERVE, SCIATIC: DEGENERATION, NERVE FIBER, MINIMAL

Appendix 10

Individual Animal Report

Study ID: 02776-0018: CRL 2954-001 / U of Tex Southwestern Med UTSW.Gray-002
Study Title: A SINGLE DOSE TOXICITY STUDY OF AAV9/SURF1 ADMINISTERED BY INTRATHECAL INJECTION IN RATS

Animal: 2009 (Male)

Sacrifice: DAY 91
Group: 2MD91 / AAV9/SURF1 LOW DOSE (0.28E12 VG)
Fate: SCHEDULED SACRIFICE / Time on Test: 91 / Cause(s) of Death:

Gross or Microscopic Finding
Disposition
Tissue: Site / Finding, Severity

MICROSCOPIC EXAMINATION

Disposition: NORMAL

BRAIN, AMYGDALOID BODY
BRAIN, BASAL NUCLEI/STRIATUM
BRAIN, CEREBELLUM
BRAIN, CEREBRAL CORTEX
BRAIN, HIPPOCAMPUS
BRAIN, HYPOTHALAMUS
BRAIN, MENINGES
BRAIN, MIDBRAIN
BRAIN, OLFACTORY BULB
BRAIN, PONS
BRAIN, THALAMUS
BRAIN, VENTRICULAR SYSTEM
BRAIN, WHITE MATTER
EYE
LYMPH NODE, ILIAC
LYMPH NODE, MANDIBULAR
LYMPH NODE, MESENTERIC
MUSCLE, BICEPS FEMORIS
MUSCLE, GASTROCNEMIUS
NERVE ROOT, SPINAL, CERVICAL
NERVE ROOT, SPINAL, THORACIC
NERVE, OPTIC
NERVE, TIBIAL
PANCREAS
SPINAL CORD, CERVICAL
SPINAL CORD, LUMBAR
SPLEEN
TESTIS
THYMUS

Disposition: ABNORMAL/FINDING

BRAIN, MEDULLA OBLONGATA
BRAIN, MEDULLA OBLONGATA, WHITE MATTER: DEGENERATION, NERVE FIBER, MINIMAL
COMMENT(S): PYRAMIDAL TRACT
GANGLION, DORSAL ROOT, CERVICAL

Appendix 10

Individual Animal Report

Study ID: 02776-0018: CRL 2954-001 / U of Tex Southwestern Med UTSW.Gray-002
Study Title: A SINGLE DOSE TOXICITY STUDY OF AAV9/SURF1 ADMINISTERED BY INTRATHECAL INJECTION IN RATS

Animal: 2009 (Male)

Sacrifice: DAY 91
Group: 2MD91 / AAV9/SURF1 LOW DOSE (0.28E12 VG)
Fate: SCHEDULED SACRIFICE / Time on Test: 91 / Cause(s) of Death:

Gross or Microscopic Finding
Disposition
Tissue: Site / Finding, Severity

GANGLION, DORSAL ROOT, CERVICAL: INFILTRATE, MONONUCLEAR CELLS, MINIMAL
GANGLION, DORSAL ROOT, CERVICAL, GLIAL CELL: HYPERTROPHY/HYPERPLASIA, MINIMAL
GANGLION, DORSAL ROOT, LUMBAR
GANGLION, DORSAL ROOT, LUMBAR, GLIAL CELL: HYPERTROPHY/HYPERPLASIA, MINIMAL
GANGLION, DORSAL ROOT, THORACIC
GANGLION, DORSAL ROOT, THORACIC, GLIAL CELL: HYPERTROPHY/HYPERPLASIA, MINIMAL
HEART
HEART, MYOCARDIUM: DEGENERATION/NECROSIS, MINIMAL, MULTIFOCAL
HEART, MYOCARDIUM: FIBROSIS, MINIMAL, MULTIFOCAL
HEART, MYOCARDIUM: INFILTRATE, MONONUCLEAR CELLS, MINIMAL, MULTIFOCAL
KIDNEY
KIDNEY, TUBULAR: CYST, MINIMAL, FOCAL
LIVER
LIVER: INFILTRATE, MIXED, MINIMAL
LUNG
LUNG, ALVEOLUS: INFILTRATE, MACROPHAGES, MINIMAL
NERVE ROOT, SPINAL, LUMBAR
NERVE ROOT, SPINAL, LUMBAR, EPINEURIUM: INFILTRATE, MONONUCLEAR CELLS, MINIMAL, FOCAL
NERVE, SCIATIC
NERVE, SCIATIC: DEGENERATION, NERVE FIBER, MINIMAL
SPINAL CORD, THORACIC
SPINAL CORD, THORACIC, WHITE MATTER: DEGENERATION, NERVE FIBER, MINIMAL
COMMENT(S): SEEN IN LONG SECTION AND EXACT LOCATION COULD NOT BE DETERMINED

Appendix 10

Individual Animal Report

Study ID: 02776-0018: CRL 2954-001 / U of Tex Southwestern Med UTSW.Gray-002
Study Title: A SINGLE DOSE TOXICITY STUDY OF AAV9/SURF1 ADMINISTERED BY INTRATHECAL INJECTION IN RATS

Animal: 2010 (Male)

Sacrifice: DAY 91
Group: 2MD91 / AAV9/SURF1 LOW DOSE (0.28E12 VG)
Fate: SCHEDULED SACRIFICE / Time on Test: 91 / Cause(s) of Death:

Gross or Microscopic Finding
Disposition
Tissue: Site / Finding, Severity

MICROSCOPIC EXAMINATION

Disposition: **NORMAL**

BRAIN, AMYGDALOID BODY
BRAIN, BASAL NUCLEI/STRIATUM
BRAIN, CEREBELLUM
BRAIN, CEREBRAL CORTEX
BRAIN, HIPPOCAMPUS
BRAIN, HYPOTHALAMUS
BRAIN, MEDULLA OBLONGATA
BRAIN, MENINGES
BRAIN, MIDBRAIN
BRAIN, OLFACTORY BULB
BRAIN, PONS
BRAIN, THALAMUS
BRAIN, VENTRICULAR SYSTEM
BRAIN, WHITE MATTER
EYE
GANGLION, DORSAL ROOT, CERVICAL
GANGLION, DORSAL ROOT, THORACIC
LUNG
LYMPH NODE, ILIAC
LYMPH NODE, MANDIBULAR
LYMPH NODE, MESENTERIC
MUSCLE, BICEPS FEMORIS
MUSCLE, GASTROCNEMIUS
NERVE ROOT, SPINAL, CERVICAL
NERVE ROOT, SPINAL, LUMBAR
NERVE ROOT, SPINAL, THORACIC
NERVE, OPTIC
NERVE, SCIATIC
NERVE, TIBIAL
PANCREAS
SPINAL CORD, CERVICAL
SPINAL CORD, LUMBAR
SPLEEN
TESTIS

Appendix 10

Individual Animal Report

Study ID: 02776-0018: CRL 2954-001 / U of Tex Southwestern Med UTSW.Gray-002
Study Title: A SINGLE DOSE TOXICITY STUDY OF AAV9/SURF1 ADMINISTERED BY INTRATHECAL INJECTION IN RATS

Animal: 2010 (Male)

Sacrifice: DAY 91
Group: 2MD91 / AAV9/SURF1 LOW DOSE (0.28E12 VG)
Fate: SCHEDULED SACRIFICE / Time on Test: 91 / Cause(s) of Death:

Gross or Microscopic Finding
Disposition
Tissue: Site / Finding, Severity

THYMUS

Disposition: **ABNORMAL/FINDING**

GANGLION, DORSAL ROOT, LUMBAR
GANGLION, DORSAL ROOT, LUMBAR, GLIAL CELL: HYPERTROPHY/HYPERPLASIA, MINIMAL
HEART
HEART, MYOCARDIUM: DEGENERATION/NECROSIS, MINIMAL, MULTIFOCAL
HEART, MYOCARDIUM: FIBROSIS, MINIMAL, MULTIFOCAL
HEART, MYOCARDIUM: INFILTRATE, MONONUCLEAR CELLS, MILD, MULTIFOCAL
KIDNEY
KIDNEY: CHRONIC PROGRESSIVE NEPHROPATHY, MINIMAL
LIVER
LIVER: INFILTRATE, MIXED, MILD
LIVER: NECROSIS, MINIMAL, FOCAL
SPINAL CORD, THORACIC
SPINAL CORD, THORACIC, WHITE MATTER: DEGENERATION, NERVE FIBER, MINIMAL
COMMENT(S): SEEN IN LONG SECTION AND EXACT LOCATION COULD NOT BE DETERMINED

Appendix 10

Individual Animal Report

Study ID: 02776-0018: CRL 2954-001 / U of Tex Southwestern Med UTSW.Gray-002
Study Title: A SINGLE DOSE TOXICITY STUDY OF AAV9/SURF1 ADMINISTERED BY INTRATHECAL INJECTION IN RATS

Animal: 3006 (Male)

Sacrifice: DAY 91
Group: 3MD91 / AAV9/SURF1 MID DOSE (0.83E12 VG)
Fate: SCHEDULED SACRIFICE / Time on Test: 87 / Cause(s) of Death:

Gross or Microscopic Finding
Disposition
Tissue: Site / Finding, Severity

MICROSCOPIC EXAMINATION

Disposition: NORMAL

BRAIN, AMYGDALOID BODY
BRAIN, BASAL NUCLEI/STRIATUM
BRAIN, CEREBELLUM
BRAIN, CEREBRAL CORTEX
BRAIN, HIPPOCAMPUS
BRAIN, HYPOTHALAMUS
BRAIN, MEDULLA OBLONGATA
BRAIN, MENINGES
BRAIN, MIDBRAIN
BRAIN, OLFACTORY BULB
BRAIN, PONS
BRAIN, THALAMUS
BRAIN, VENTRICULAR SYSTEM
BRAIN, WHITE MATTER
EYE
GANGLION, DORSAL ROOT, CERVICAL
KIDNEY
LYMPH NODE, MANDIBULAR
LYMPH NODE, MESENTERIC
MUSCLE, BICEPS FEMORIS
MUSCLE, GASTROCNEMIUS
NERVE ROOT, SPINAL, CERVICAL
NERVE ROOT, SPINAL, LUMBAR
NERVE ROOT, SPINAL, THORACIC
NERVE, OPTIC
PANCREAS
SPINAL CORD, CERVICAL
SPLEEN
TESTIS
THYMUS

Disposition: ABNORMAL/FINDING

GANGLION, DORSAL ROOT, LUMBAR
GANGLION, DORSAL ROOT, LUMBAR: INFILTRATE, MONONUCLEAR CELLS, MINIMAL

Appendix 10

Individual Animal Report

Study ID: 02776-0018: CRL 2954-001 / U of Tex Southwestern Med UTSW.Gray-002
Study Title: A SINGLE DOSE TOXICITY STUDY OF AAV9/SURF1 ADMINISTERED BY INTRATHECAL INJECTION IN RATS

Animal: 3006 (Male)

Sacrifice: DAY 91
Group: 3MD91 / AAV9/SURF1 MID DOSE (0.83E12 VG)
Fate: SCHEDULED SACRIFICE / Time on Test: 87 / Cause(s) of Death:

Gross or Microscopic Finding
Disposition
Tissue: Site / Finding, Severity

GANGLION, DORSAL ROOT, THORACIC
GANGLION, DORSAL ROOT, THORACIC: INFILTRATE, MONONUCLEAR CELLS, MINIMAL, FOCAL
HEART
HEART, MYOCARDIUM: DEGENERATION/NECROSIS, MILD, MULTIFOCAL
HEART, MYOCARDIUM: FIBROSIS, MODERATE, MULTIFOCAL
HEART, MYOCARDIUM: INFILTRATE, MONONUCLEAR CELLS, MARKED, MULTIFOCAL
LIVER
LIVER: INFILTRATE, MIXED, MINIMAL
LUNG
LUNG, ALVEOLUS: INFILTRATE, MACROPHAGES, MINIMAL, FOCAL
LYMPH NODE, ILIAC
LYMPH NODE, ILIAC: INCREASED CELLULARITY, MINIMAL
COMMENT(S): INCREASED PLASMA CELLS WITHIN MEDULLARY CORDS
NERVE, SCIATIC
NERVE, SCIATIC: DEGENERATION, NERVE FIBER, MILD
NERVE, TIBIAL
NERVE, TIBIAL: DEGENERATION, NERVE FIBER, MILD
SPINAL CORD, LUMBAR
SPINAL CORD, LUMBAR, WHITE MATTER: DEGENERATION, NERVE FIBER, MINIMAL, DORSAL
COMMENT(S): DEGENERATION ALSO SEEN IN LONG SECTION BUT EXACT LOCATION COULD NOT BE DETERMINED IN THIS ORIENTATION
SPINAL CORD, THORACIC
SPINAL CORD, THORACIC, WHITE MATTER: DEGENERATION, NERVE FIBER, MINIMAL, DORSAL
COMMENT(S): DEGENERATION ALSO SEEN IN LONG SECTION BUT EXACT LOCATION COULD NOT BE DETERMINED IN THIS ORIENTATION

Appendix 10

Individual Animal Report

Study ID: 02776-0018: CRL 2954-001 / U of Tex Southwestern Med UTSW.Gray-002
Study Title: A SINGLE DOSE TOXICITY STUDY OF AAV9/SURF1 ADMINISTERED BY INTRATHECAL INJECTION IN RATS

Animal: 3007 (Male)

Sacrifice: DAY 91
Group: 3MD91 / AAV9/SURF1 MID DOSE (0.83E12 VG)
Fate: SCHEDULED SACRIFICE / Time on Test: 87 / Cause(s) of Death:

Gross or Microscopic Finding
Disposition
Tissue: Site / Finding, Severity

MICROSCOPIC EXAMINATION

Disposition: NORMAL

BRAIN, AMYGDALOID BODY
BRAIN, BASAL NUCLEI/STRIATUM
BRAIN, CEREBELLUM
BRAIN, CEREBRAL CORTEX
BRAIN, HIPPOCAMPUS
BRAIN, HYPOTHALAMUS
BRAIN, MEDULLA OBLONGATA
BRAIN, MENINGES
BRAIN, MIDBRAIN
BRAIN, OLFACTORY BULB
BRAIN, PONS
BRAIN, THALAMUS
BRAIN, VENTRICULAR SYSTEM
BRAIN, WHITE MATTER
EYE
LYMPH NODE, ILIAC
LYMPH NODE, MANDIBULAR
LYMPH NODE, MESENTERIC
MUSCLE, BICEPS FEMORIS
MUSCLE, GASTROCNEMIUS
NERVE ROOT, SPINAL, CERVICAL
NERVE ROOT, SPINAL, LUMBAR
NERVE ROOT, SPINAL, THORACIC
NERVE, OPTIC
NERVE, TIBIAL
PANCREAS
SPINAL CORD, LUMBAR
SPLEEN
TESTIS
THYMUS

Disposition: ABNORMAL/FINDING

GANGLION, DORSAL ROOT, CERVICAL
GANGLION, DORSAL ROOT, CERVICAL, GLIAL CELL: HYPERTROPHY/HYPERPLASIA, MINIMAL

Appendix 10

Individual Animal Report

Study ID: 02776-0018: CRL 2954-001 / U of Tex Southwestern Med UTSW.Gray-002
Study Title: A SINGLE DOSE TOXICITY STUDY OF AAV9/SURF1 ADMINISTERED BY INTRATHECAL INJECTION IN RATS

Animal: 3007 (Male)

Sacrifice: DAY 91
Group: 3MD91 / AAV9/SURF1 MID DOSE (0.83E12 VG)
Fate: SCHEDULED SACRIFICE / Time on Test: 87 / Cause(s) of Death:

Gross or Microscopic Finding
Disposition
Tissue: Site / Finding, Severity

GANGLION, DORSAL ROOT, LUMBAR
GANGLION, DORSAL ROOT, LUMBAR: INFILTRATE, MONONUCLEAR CELLS, MINIMAL, FOCAL
GANGLION, DORSAL ROOT, LUMBAR, GLIAL CELL: HYPERTROPHY/HYPERPLASIA, MINIMAL
GANGLION, DORSAL ROOT, THORACIC
GANGLION, DORSAL ROOT, THORACIC, GLIAL CELL: HYPERTROPHY/HYPERPLASIA, MINIMAL
HEART
HEART, MYOCARDIUM: DEGENERATION/NECROSIS, MINIMAL, MULTIFOCAL
HEART, MYOCARDIUM: INFILTRATE, MONONUCLEAR CELLS, MINIMAL, MULTIFOCAL
KIDNEY
KIDNEY: CHRONIC PROGRESSIVE NEPHROPATHY, MINIMAL
LIVER
LIVER: INFILTRATE, MIXED, MINIMAL
LIVER: NECROSIS, MINIMAL, FOCAL
LUNG
LUNG: INFLAMMATION, MINIMAL, SUBACUTE, LOCALLY EXTENSIVE
NERVE, SCIATIC
NERVE, SCIATIC: DEGENERATION, NERVE FIBER, MINIMAL
SPINAL CORD, CERVICAL
SPINAL CORD, CERVICAL, WHITE MATTER: DEGENERATION, NERVE FIBER, MINIMAL
COMMENT(S): SEEN IN LONG SECTION AND EXACT LOCATION COULD NOT BE DETERMINED
SPINAL CORD, THORACIC
SPINAL CORD, THORACIC, WHITE MATTER: DEGENERATION, NERVE FIBER, MINIMAL
COMMENT(S): SEEN IN LONG SECTION AND EXACT LOCATION COULD NOT BE DETERMINED

Appendix 10

Individual Animal Report

Study ID: 02776-0018: CRL 2954-001 / U of Tex Southwestern Med UTSW.Gray-002
Study Title: A SINGLE DOSE TOXICITY STUDY OF AAV9/SURF1 ADMINISTERED BY INTRATHECAL INJECTION IN RATS

Animal: 3009 (Male)

Sacrifice: DAY 91
Group: 3MD91 / AAV9/SURF1 MID DOSE (0.83E12 VG)
Fate: SCHEDULED SACRIFICE / Time on Test: 91 / Cause(s) of Death:

Gross or Microscopic Finding
Disposition
Tissue: Site / Finding, Severity

MICROSCOPIC EXAMINATION

Disposition: NORMAL

BRAIN, AMYGDALOID BODY
BRAIN, BASAL NUCLEI/STRIATUM
BRAIN, CEREBELLUM
BRAIN, CEREBRAL CORTEX
BRAIN, HIPPOCAMPUS
BRAIN, HYPOTHALAMUS
BRAIN, MENINGES
BRAIN, OLFACTORY BULB
BRAIN, PONS
BRAIN, THALAMUS
BRAIN, VENTRICULAR SYSTEM
BRAIN, WHITE MATTER
EYE
LUNG
LYMPH NODE, ILIAC
LYMPH NODE, MANDIBULAR
LYMPH NODE, MESENTERIC
MUSCLE, BICEPS FEMORIS
MUSCLE, GASTROCNEMIUS
NERVE ROOT, SPINAL, CERVICAL
NERVE ROOT, SPINAL, LUMBAR
NERVE ROOT, SPINAL, THORACIC
NERVE, OPTIC
SPLEEN
TESTIS
THYMUS

Disposition: ABNORMAL/FINDING

BRAIN, MEDULLA OBLONGATA
BRAIN, MEDULLA OBLONGATA, WHITE MATTER: DEGENERATION, NERVE FIBER, MINIMAL
COMMENT(S): PYRAMIDAL TRACT
BRAIN, MIDBRAIN
BRAIN, MIDBRAIN, PINEAL GLAND: INFILTRATE, MONONUCLEAR CELLS, MINIMAL
GANGLION, DORSAL ROOT, CERVICAL
GANGLION, DORSAL ROOT, CERVICAL, GLIAL CELL: HYPERTROPHY/HYPERPLASIA, MINIMAL

Appendix 10

Individual Animal Report

Study ID: 02776-0018: CRL 2954-001 / U of Tex Southwestern Med UTSW.Gray-002
Study Title: A SINGLE DOSE TOXICITY STUDY OF AAV9/SURF1 ADMINISTERED BY INTRATHECAL INJECTION IN RATS

Animal: 3009 (Male)

Sacrifice: DAY 91
Group: 3MD91 / AAV9/SURF1 MID DOSE (0.83E12 VG)
Fate: SCHEDULED SACRIFICE / Time on Test: 91 / Cause(s) of Death:

Gross or Microscopic Finding
Disposition
Tissue: Site / Finding, Severity

GANGLION, DORSAL ROOT, LUMBAR
GANGLION, DORSAL ROOT, LUMBAR, GLIAL CELL: HYPERTROPHY/HYPERPLASIA, MINIMAL
GANGLION, DORSAL ROOT, THORACIC
GANGLION, DORSAL ROOT, THORACIC: INFILTRATE, MONONUCLEAR CELLS, MINIMAL, FOCAL
GANGLION, DORSAL ROOT, THORACIC, GLIAL CELL: HYPERTROPHY/HYPERPLASIA, MINIMAL
HEART
HEART, MYOCARDIUM: DEGENERATION/NECROSIS, MINIMAL, MULTIFOCAL
HEART, MYOCARDIUM: INFILTRATE, MONONUCLEAR CELLS, MINIMAL, MULTIFOCAL
KIDNEY
KIDNEY, TUBULAR: DILATATION, MINIMAL, FOCAL
LIVER
LIVER: INFILTRATE, MIXED, MINIMAL
NERVE, SCIATIC
NERVE, SCIATIC: DEGENERATION, NERVE FIBER, MINIMAL
NERVE, TIBIAL
NERVE, TIBIAL: DEGENERATION, NERVE FIBER, MINIMAL
PANCREAS
PANCREAS: INFILTRATE, MONONUCLEAR CELLS, MINIMAL, MULTIFOCAL
SPINAL CORD, CERVICAL
SPINAL CORD, CERVICAL, WHITE MATTER: DEGENERATION, NERVE FIBER, MINIMAL
COMMENT(S): SEEN IN LONG SECTION AND EXACT LOCATION COULD NOT BE DETERMINED
SPINAL CORD, LUMBAR
SPINAL CORD, LUMBAR, WHITE MATTER: DEGENERATION, NERVE FIBER, MINIMAL, DORSAL
SPINAL CORD, THORACIC
SPINAL CORD, THORACIC, WHITE MATTER: DEGENERATION, NERVE FIBER, MINIMAL
COMMENT(S): SEEN IN LONG SECTION AND EXACT LOCATION COULD NOT BE DETERMINED

Appendix 10

Individual Animal Report

Study ID: 02776-0018: CRL 2954-001 / U of Tex Southwestern Med UTSW.Gray-002
Study Title: A SINGLE DOSE TOXICITY STUDY OF AAV9/SURF1 ADMINISTERED BY INTRATHECAL INJECTION IN RATS

Animal: 3010 (Male)

Sacrifice: DAY 91
Group: 3MD91 / AAV9/SURF1 MID DOSE (0.83E12 VG)
Fate: SCHEDULED SACRIFICE / Time on Test: 91 / Cause(s) of Death:

Gross or Microscopic Finding
Disposition
Tissue: Site / Finding, Severity

MICROSCOPIC EXAMINATION

Disposition: NORMAL

BRAIN, AMYGDALOID BODY
BRAIN, BASAL NUCLEI/STRIATUM
BRAIN, CEREBELLUM
BRAIN, CEREBRAL CORTEX
BRAIN, HIPPOCAMPUS
BRAIN, HYPOTHALAMUS
BRAIN, MEDULLA OBLONGATA
BRAIN, MENINGES
BRAIN, MIDBRAIN
BRAIN, OLFACTORY BULB
BRAIN, PONS
BRAIN, THALAMUS
BRAIN, VENTRICULAR SYSTEM
BRAIN, WHITE MATTER
KIDNEY
LIVER
LUNG
LYMPH NODE, ILIAC
LYMPH NODE, MANDIBULAR
LYMPH NODE, MESENTERIC
MUSCLE, BICEPS FEMORIS
MUSCLE, GASTROCNEMIUS
NERVE ROOT, SPINAL, CERVICAL
NERVE ROOT, SPINAL, THORACIC
NERVE, OPTIC
NERVE, TIBIAL
PANCREAS
SPINAL CORD, CERVICAL
SPINAL CORD, THORACIC
SPLEEN
TESTIS
THYMUS

Appendix 10

Individual Animal Report

Study ID: 02776-0018: CRL 2954-001 / U of Tex Southwestern Med UTSW.Gray-002
Study Title: A SINGLE DOSE TOXICITY STUDY OF AAV9/SURF1 ADMINISTERED BY INTRATHECAL INJECTION IN RATS

Animal: 3010 (Male)

Sacrifice: DAY 91
Group: 3MD91 / AAV9/SURF1 MID DOSE (0.83E12 VG)
Fate: SCHEDULED SACRIFICE / Time on Test: 91 / Cause(s) of Death:

Gross or Microscopic Finding
Disposition
Tissue: Site / Finding, Severity

Disposition: ABNORMAL/FINDING

EYE
EYE: RETINAL ROSETTE, MARKED
GANGLION, DORSAL ROOT, CERVICAL
GANGLION, DORSAL ROOT, CERVICAL, GLIAL CELL: HYPERTROPHY/HYPERPLASIA, MINIMAL
GANGLION, DORSAL ROOT, LUMBAR
GANGLION, DORSAL ROOT, LUMBAR: INFILTRATE, MONONUCLEAR CELLS, MINIMAL, FOCAL
GANGLION, DORSAL ROOT, LUMBAR, GLIAL CELL: HYPERTROPHY/HYPERPLASIA, MINIMAL
GANGLION, DORSAL ROOT, THORACIC
GANGLION, DORSAL ROOT, THORACIC, GLIAL CELL: HYPERTROPHY/HYPERPLASIA, MINIMAL
HEART
HEART, MYOCARDIUM: DEGENERATION/NECROSIS, MINIMAL, MULTIFOCAL
HEART, MYOCARDIUM: INFILTRATE, MONONUCLEAR CELLS, MINIMAL, MULTIFOCAL
NERVE ROOT, SPINAL, LUMBAR
NERVE ROOT, SPINAL, LUMBAR, NERVE ROOT, VENTRAL: DEGENERATION, NERVE FIBER, MINIMAL
NERVE, SCIATIC
NERVE, SCIATIC: DEGENERATION, NERVE FIBER, MINIMAL
SPINAL CORD, LUMBAR
SPINAL CORD, LUMBAR, NERVE ROOT, SPINAL: DEGENERATION, NERVE FIBER, MILD

Appendix 10

Individual Animal Report

Study ID: 02776-0018: CRL 2954-001 / U of Tex Southwestern Med UTSW.Gray-002
Study Title: A SINGLE DOSE TOXICITY STUDY OF AAV9/SURF1 ADMINISTERED BY INTRATHECAL INJECTION IN RATS

Animal: 4006 (Male)

Sacrifice: DAY 91
Group: 4MD91 / AAV9/SURF1 HIGH DOSE (2.49E12 VG)
Fate: SCHEDULED SACRIFICE / Time on Test: 87 / Cause(s) of Death:

Gross or Microscopic Finding
Disposition
Tissue: Site / Finding, Severity

MICROSCOPIC EXAMINATION

Disposition: NORMAL

BRAIN, AMYGDALOID BODY
BRAIN, BASAL NUCLEI/STRIATUM
BRAIN, CEREBELLUM
BRAIN, CEREBRAL CORTEX
BRAIN, HIPPOCAMPUS
BRAIN, HYPOTHALAMUS
BRAIN, MEDULLA OBLONGATA
BRAIN, MENINGES
BRAIN, MIDBRAIN
BRAIN, OLFACTORY BULB
BRAIN, PONS
BRAIN, THALAMUS
BRAIN, VENTRICULAR SYSTEM
BRAIN, WHITE MATTER
EYE
GANGLION, DORSAL ROOT, THORACIC
LYMPH NODE, ILIAC
LYMPH NODE, MANDIBULAR
LYMPH NODE, MESENTERIC
MUSCLE, BICEPS FEMORIS
MUSCLE, GASTROCNEMIUS
NERVE ROOT, SPINAL, CERVICAL
NERVE ROOT, SPINAL, THORACIC
NERVE, OPTIC
SPINAL CORD, LUMBAR
SPINAL CORD, THORACIC
SPLEEN
TESTIS

Disposition: ABNORMAL/FINDING

GANGLION, DORSAL ROOT, CERVICAL
GANGLION, DORSAL ROOT, CERVICAL, GLIAL CELL: HYPERTROPHY/HYPERPLASIA, MINIMAL
GANGLION, DORSAL ROOT, LUMBAR
GANGLION, DORSAL ROOT, LUMBAR, GLIAL CELL: HYPERTROPHY/HYPERPLASIA, MINIMAL

Appendix 10

Individual Animal Report

Study ID: 02776-0018: CRL 2954-001 / U of Tex Southwestern Med UTSW.Gray-002
Study Title: A SINGLE DOSE TOXICITY STUDY OF AAV9/SURF1 ADMINISTERED BY INTRATHECAL INJECTION IN RATS

Animal: 4006 (Male)

Sacrifice: DAY 91
Group: 4MD91 / AAV9/SURF1 HIGH DOSE (2.49E12 VG)
Fate: SCHEDULED SACRIFICE / Time on Test: 87 / Cause(s) of Death:

Gross or Microscopic Finding
Disposition
Tissue: Site / Finding, Severity

HEART
HEART, MYOCARDIUM: DEGENERATION/NECROSIS, MINIMAL, MULTIFOCAL
HEART, MYOCARDIUM: FIBROSIS, MILD, MULTIFOCAL
HEART, MYOCARDIUM: INFILTRATE, MONONUCLEAR CELLS, MILD, MULTIFOCAL
KIDNEY
KIDNEY: CHRONIC PROGRESSIVE NEPHROPATHY, MINIMAL
LIVER
LIVER: INFILTRATE, MIXED, MINIMAL
LUNG
LUNG, ALVEOLUS: INFILTRATE, MACROPHAGES, MINIMAL, FOCAL
NERVE ROOT, SPINAL, LUMBAR
NERVE ROOT, SPINAL, LUMBAR, NERVE ROOT, DORSAL: DEGENERATION, NERVE FIBER, MINIMAL
NERVE, SCIATIC
NERVE, SCIATIC: DEGENERATION, NERVE FIBER, MINIMAL
NERVE, TIBIAL
NERVE, TIBIAL: DEGENERATION, NERVE FIBER, MINIMAL
PANCREAS
PANCREAS: FIBROSIS, MINIMAL, FOCAL
COMMENT(S): AFFECTING ISLETS OF LANGERHANS
SPINAL CORD, CERVICAL
SPINAL CORD, CERVICAL, WHITE MATTER: DEGENERATION, NERVE FIBER, MINIMAL, DORSAL

Disposition: UNABLE TO OBTAIN SPECIMEN
THYMUS

Appendix 10

Individual Animal Report

Study ID: 02776-0018: CRL 2954-001 / U of Tex Southwestern Med UTSW.Gray-002
Study Title: A SINGLE DOSE TOXICITY STUDY OF AAV9/SURF1 ADMINISTERED BY INTRATHECAL INJECTION IN RATS

Animal: 4007 (Male)

Sacrifice: DAY 91
Group: 4MD91 / AAV9/SURF1 HIGH DOSE (2.49E12 VG)
Fate: SCHEDULED SACRIFICE / Time on Test: 87 / Cause(s) of Death:

Gross or Microscopic Finding
Disposition
Tissue: Site / Finding, Severity

MICROSCOPIC EXAMINATION

Disposition: NORMAL

BRAIN, AMYGDALOID BODY
BRAIN, BASAL NUCLEI/STRIATUM
BRAIN, CEREBELLUM
BRAIN, CEREBRAL CORTEX
BRAIN, HIPPOCAMPUS
BRAIN, HYPOTHALAMUS
BRAIN, MEDULLA OBLONGATA
BRAIN, MENINGES
BRAIN, MIDBRAIN
BRAIN, OLFACTORY BULB
BRAIN, PONS
BRAIN, THALAMUS
BRAIN, VENTRICULAR SYSTEM
BRAIN, WHITE MATTER
EYE
GANGLION, DORSAL ROOT, CERVICAL
KIDNEY
LUNG
LYMPH NODE, ILIAC
LYMPH NODE, MANDIBULAR
LYMPH NODE, MESENTERIC
MUSCLE, BICEPS FEMORIS
MUSCLE, GASTROCNEMIUS
NERVE ROOT, SPINAL, CERVICAL
NERVE ROOT, SPINAL, THORACIC
NERVE, OPTIC
PANCREAS
SPINAL CORD, LUMBAR
SPLEEN
TESTIS
THYMUS

Disposition: ABNORMAL/FINDING

GANGLION, DORSAL ROOT, LUMBAR

Appendix 10

Individual Animal Report

Study ID: 02776-0018: CRL 2954-001 / U of Tex Southwestern Med UTSW.Gray-002
Study Title: A SINGLE DOSE TOXICITY STUDY OF AAV9/SURF1 ADMINISTERED BY INTRATHECAL INJECTION IN RATS

Animal: 4007 (Male)

Sacrifice: DAY 91
Group: 4MD91 / AAV9/SURF1 HIGH DOSE (2.49E12 VG)
Fate: SCHEDULED SACRIFICE / Time on Test: 87 / Cause(s) of Death:

Gross or Microscopic Finding
Disposition
Tissue: Site / Finding, Severity

GANGLION, DORSAL ROOT, LUMBAR: INFILTRATE, MONONUCLEAR CELLS, MINIMAL, MULTIFOCAL
GANGLION, DORSAL ROOT, LUMBAR, GLIAL CELL: HYPERTROPHY/HYPERPLASIA, MINIMAL
GANGLION, DORSAL ROOT, THORACIC
GANGLION, DORSAL ROOT, THORACIC, GLIAL CELL: HYPERTROPHY/HYPERPLASIA, MINIMAL
HEART
HEART, MYOCARDIUM: DEGENERATION/NECROSIS, MINIMAL, MULTIFOCAL
HEART, MYOCARDIUM: FIBROSIS, MILD, MULTIFOCAL
HEART, MYOCARDIUM: INFILTRATE, MONONUCLEAR CELLS, MILD, MULTIFOCAL
LIVER
LIVER: INFILTRATE, MIXED, MINIMAL
NERVE ROOT, SPINAL, LUMBAR
NERVE ROOT, SPINAL, LUMBAR, EPINEURIUM: INFILTRATE, MONONUCLEAR CELLS, MINIMAL, FOCAL
NERVE ROOT, SPINAL, LUMBAR, NERVE ROOT, DORSAL: DEGENERATION, NERVE FIBER, MILD
NERVE, SCIATIC
NERVE, SCIATIC: DEGENERATION, NERVE FIBER, MILD
NERVE, TIBIAL
NERVE, TIBIAL: DEGENERATION, NERVE FIBER, MINIMAL
SPINAL CORD, CERVICAL
SPINAL CORD, CERVICAL, WHITE MATTER: DEGENERATION, NERVE FIBER, MINIMAL
COMMENT(S): SEEN IN LONG SECTION AND EXACT LOCATION COULD NOT BE DETERMINED
SPINAL CORD, THORACIC
SPINAL CORD, THORACIC, GLIAL CELL: INCREASED CELLULARITY, MINIMAL, FOCAL
COMMENT(S): UNILATERAL WITHIN DORSALMOST PORTION OF GRAY MATTER

Appendix 10

Individual Animal Report

Study ID: 02776-0018: CRL 2954-001 / U of Tex Southwestern Med UTSW.Gray-002
Study Title: A SINGLE DOSE TOXICITY STUDY OF AAV9/SURF1 ADMINISTERED BY INTRATHECAL INJECTION IN RATS

Animal: 4008 (Male)

Sacrifice: DAY 91
Group: 4MD91 / AAV9/SURF1 HIGH DOSE (2.49E12 VG)
Fate: SCHEDULED SACRIFICE / Time on Test: 87 / Cause(s) of Death:

Gross or Microscopic Finding
Disposition
Tissue: Site / Finding, Severity

MICROSCOPIC EXAMINATION

Disposition: NORMAL

BRAIN, AMYGDALOID BODY
BRAIN, BASAL NUCLEI/STRIATUM
BRAIN, CEREBELLUM
BRAIN, CEREBRAL CORTEX
BRAIN, HIPPOCAMPUS
BRAIN, HYPOTHALAMUS
BRAIN, MEDULLA OBLONGATA
BRAIN, MENINGES
BRAIN, MIDBRAIN
BRAIN, OLFACTORY BULB
BRAIN, PONS
BRAIN, THALAMUS
BRAIN, VENTRICULAR SYSTEM
BRAIN, WHITE MATTER
EYE
GANGLION, DORSAL ROOT, LUMBAR
GANGLION, DORSAL ROOT, THORACIC
LUNG
LYMPH NODE, ILIAC
LYMPH NODE, MANDIBULAR
LYMPH NODE, MESENTERIC
MUSCLE, BICEPS FEMORIS
MUSCLE, GASTROCNEMIUS
NERVE ROOT, SPINAL, CERVICAL
NERVE ROOT, SPINAL, LUMBAR
NERVE ROOT, SPINAL, THORACIC
NERVE, OPTIC
NERVE, SCIATIC
NERVE, TIBIAL
SPINAL CORD, CERVICAL
SPINAL CORD, LUMBAR
SPLEEN
TESTIS
THYMUS

Appendix 10

Individual Animal Report

Study ID: 02776-0018: CRL 2954-001 / U of Tex Southwestern Med UTSW.Gray-002
Study Title: A SINGLE DOSE TOXICITY STUDY OF AAV9/SURF1 ADMINISTERED BY INTRATHECAL INJECTION IN RATS

Animal: 4008 (Male)

Sacrifice: DAY 91
Group: 4MD91 / AAV9/SURF1 HIGH DOSE (2.49E12 VG)
Fate: SCHEDULED SACRIFICE / Time on Test: 87 / Cause(s) of Death:

Gross or Microscopic Finding
Disposition
Tissue: Site / Finding, Severity

Disposition: **ABNORMAL/FINDING**

GANGLION, DORSAL ROOT, CERVICAL
GANGLION, DORSAL ROOT, CERVICAL, GLIAL CELL: HYPERTROPHY/HYPERPLASIA, MINIMAL
HEART
HEART, MYOCARDIUM: DEGENERATION/NECROSIS, MINIMAL, MULTIFOCAL
HEART, MYOCARDIUM: FIBROSIS, MILD, MULTIFOCAL
HEART, MYOCARDIUM: INFILTRATE, MONONUCLEAR CELLS, MILD, MULTIFOCAL
KIDNEY
KIDNEY, TUBULAR: BASOPHILIA, MINIMAL
LIVER
LIVER: INFILTRATE, MIXED, MINIMAL
PANCREAS
PANCREAS: ATROPHY, MINIMAL, FOCAL
COMMENT(S): AFFECTING EXOCRINE/ACINAR CELLS
SPINAL CORD, THORACIC
SPINAL CORD, THORACIC, WHITE MATTER: DEGENERATION, NERVE FIBER, MINIMAL, VENTRAL

Appendix 10

Individual Animal Report

Study ID: 02776-0018: CRL 2954-001 / U of Tex Southwestern Med UTSW.Gray-002
Study Title: A SINGLE DOSE TOXICITY STUDY OF AAV9/SURF1 ADMINISTERED BY INTRATHECAL INJECTION IN RATS

Animal: 4009 (Male)

Sacrifice: DAY 91
Group: 4MD91 / AAV9/SURF1 HIGH DOSE (2.49E12 VG)
Fate: SCHEDULED SACRIFICE / Time on Test: 91 / Cause(s) of Death:

Gross or Microscopic Finding
Disposition
Tissue: Site / Finding, Severity

GROSS EXAMINATION

Disposition:

TGL #1: KIDNEY; GROSS LESION: DILATATION; RIGHT (SEE COMMENTS REPORT) // MICROSCOPIC CORRELATION:

MICROSCOPIC EXAMINATION

Disposition: NORMAL

BRAIN, AMYGDALOID BODY
BRAIN, BASAL NUCLEI/STRIATUM
BRAIN, CEREBELLUM
BRAIN, CEREBRAL CORTEX
BRAIN, HIPPOCAMPUS
BRAIN, HYPOTHALAMUS
BRAIN, MEDULLA OBLONGATA
BRAIN, MENINGES
BRAIN, MIDBRAIN
BRAIN, OLFACTORY BULB
BRAIN, PONS
BRAIN, THALAMUS
BRAIN, VENTRICULAR SYSTEM
BRAIN, WHITE MATTER
GANGLION, DORSAL ROOT, CERVICAL
GANGLION, DORSAL ROOT, LUMBAR
GANGLION, DORSAL ROOT, THORACIC
KIDNEY
LYMPH NODE, ILIAC
LYMPH NODE, MANDIBULAR
LYMPH NODE, MESENTERIC
MUSCLE, BICEPS FEMORIS
MUSCLE, GASTROCNEMIUS
NERVE ROOT, SPINAL, CERVICAL
NERVE ROOT, SPINAL, LUMBAR
NERVE ROOT, SPINAL, THORACIC
NERVE, OPTIC
NERVE, SCIATIC
NERVE, TIBIAL

Appendix 10

Individual Animal Report

Study ID: 02776-0018: CRL 2954-001 / U of Tex Southwestern Med UTSW.Gray-002
Study Title: A SINGLE DOSE TOXICITY STUDY OF AAV9/SURF1 ADMINISTERED BY INTRATHECAL INJECTION IN RATS

Animal: 4009 (Male)

Sacrifice: DAY 91
Group: 4MD91 / AAV9/SURF1 HIGH DOSE (2.49E12 VG)
Fate: SCHEDULED SACRIFICE / Time on Test: 91 / Cause(s) of Death:

Gross or Microscopic Finding
Disposition
Tissue: Site / Finding, Severity

SPINAL CORD, CERVICAL
SPINAL CORD, LUMBAR
SPLEEN
TESTIS
THYMUS

Disposition: **ABNORMAL/FINDING**

EYE
EYE: RETINAL ROSETTE, MILD
HEART
HEART, MYOCARDIUM: FIBROSIS, MINIMAL, MULTIFOCAL
LIVER
LIVER: INFILTRATE, MIXED, MINIMAL
LUNG
LUNG, ALVEOLUS: INFILTRATE, MACROPHAGES, MINIMAL, FOCAL
PANCREAS
PANCREAS: ATROPHY, MINIMAL, FOCAL
COMMENT(S): AFFECTING EXOCRINE/ACINAR CELLS
PANCREAS: FIBROSIS, MINIMAL, MULTIFOCAL
COMMENT(S): AFFECTING ISLETS OF LANGERHANS
SPINAL CORD, THORACIC
SPINAL CORD, THORACIC, WHITE MATTER: DEGENERATION, NERVE FIBER, MINIMAL
COMMENT(S): SEEN IN LONG SECTION AND EXACT LOCATION COULD NOT BE DETERMINED

Appendix 10

Individual Animal Report

Study ID: 02776-0018: CRL 2954-001 / U of Tex Southwestern Med UTSW.Gray-002
Study Title: A SINGLE DOSE TOXICITY STUDY OF AAV9/SURF1 ADMINISTERED BY INTRATHECAL INJECTION IN RATS

Animal: 4010 (Male)

Sacrifice: DAY 91
Group: 4MD91 / AAV9/SURF1 HIGH DOSE (2.49E12 VG)
Fate: SCHEDULED SACRIFICE / Time on Test: 91 / Cause(s) of Death:

Gross or Microscopic Finding
Disposition
Tissue: Site / Finding, Severity

MICROSCOPIC EXAMINATION

Disposition: NORMAL

BRAIN, AMYGDALOID BODY
BRAIN, BASAL NUCLEI/STRIATUM
BRAIN, CEREBELLUM
BRAIN, CEREBRAL CORTEX
BRAIN, HIPPOCAMPUS
BRAIN, HYPOTHALAMUS
BRAIN, MENINGES
BRAIN, MIDBRAIN
BRAIN, OLFACTORY BULB
BRAIN, PONS
BRAIN, THALAMUS
BRAIN, VENTRICULAR SYSTEM
BRAIN, WHITE MATTER
EYE
GANGLION, DORSAL ROOT, CERVICAL
KIDNEY
LYMPH NODE, ILIAC
LYMPH NODE, MANDIBULAR
LYMPH NODE, MESENTERIC
MUSCLE, BICEPS FEMORIS
MUSCLE, GASTROCNEMIUS
NERVE ROOT, SPINAL, CERVICAL
NERVE ROOT, SPINAL, THORACIC
NERVE, OPTIC
SPINAL CORD, CERVICAL
SPLEEN
TESTIS
THYMUS

Disposition: ABNORMAL/FINDING

BRAIN, MEDULLA OBLONGATA
BRAIN, MEDULLA OBLONGATA, WHITE MATTER: DEGENERATION, NERVE FIBER, MINIMAL
COMMENT(S): PYRAMIDAL TRACT
GANGLION, DORSAL ROOT, LUMBAR
GANGLION, DORSAL ROOT, LUMBAR: INFILTRATE, MONONUCLEAR CELLS, MINIMAL, MULTIFOCAL

Appendix 10

Individual Animal Report

Study ID: 02776-0018: CRL 2954-001 / U of Tex Southwestern Med UTSW.Gray-002
Study Title: A SINGLE DOSE TOXICITY STUDY OF AAV9/SURF1 ADMINISTERED BY INTRATHECAL INJECTION IN RATS

Animal: 4010 (Male)

Sacrifice: DAY 91
Group: 4MD91 / AAV9/SURF1 HIGH DOSE (2.49E12 VG)
Fate: SCHEDULED SACRIFICE / Time on Test: 91 / Cause(s) of Death:

Gross or Microscopic Finding
Disposition
Tissue: Site / Finding, Severity

GANGLION, DORSAL ROOT, LUMBAR, GLIAL CELL: HYPERTROPHY/HYPERPLASIA, MINIMAL
GANGLION, DORSAL ROOT, THORACIC
GANGLION, DORSAL ROOT, THORACIC, GLIAL CELL: HYPERTROPHY/HYPERPLASIA, MINIMAL
HEART
HEART, MYOCARDIUM: DEGENERATION/NECROSIS, MINIMAL, MULTIFOCAL
HEART, MYOCARDIUM: FIBROSIS, MINIMAL, MULTIFOCAL
HEART, MYOCARDIUM: INFILTRATE, MONONUCLEAR CELLS, MINIMAL, MULTIFOCAL
LIVER
LIVER: NECROSIS, MINIMAL, FOCAL
LUNG
LUNG: INFLAMMATION, MINIMAL, SUBACUTE, FOCAL
NERVE ROOT, SPINAL, LUMBAR
NERVE ROOT, SPINAL, LUMBAR, NERVE ROOT, VENTRAL: DEGENERATION, NERVE FIBER, MINIMAL
NERVE, SCIATIC
NERVE, SCIATIC: DEGENERATION, NERVE FIBER, MINIMAL
NERVE, TIBIAL
NERVE, TIBIAL: DEGENERATION, NERVE FIBER, MINIMAL
PANCREAS
PANCREAS: FIBROSIS, MILD, MULTIFOCAL
COMMENT(S): AFFECTING ISLETS OF LANGERHANS
SPINAL CORD, LUMBAR
SPINAL CORD, LUMBAR, NERVE ROOT, SPINAL: DEGENERATION, NERVE FIBER, MINIMAL
SPINAL CORD, THORACIC
SPINAL CORD, THORACIC, NERVE ROOT, SPINAL: DEGENERATION, NERVE FIBER, MINIMAL

Appendix 10

Individual Animal Report

Study ID: 02776-0018: CRL 2954-001 / U of Tex Southwestern Med UTSW.Gray-002
Study Title: A SINGLE DOSE TOXICITY STUDY OF AAV9/SURF1 ADMINISTERED BY INTRATHECAL INJECTION IN RATS

Animal: 1506 (Female)

Sacrifice: DAY 91
Group: 1FD91 / VEHICLE (0 VG)
Fate: SCHEDULED SACRIFICE / Time on Test: 90 / Cause(s) of Death:

Gross or Microscopic Finding
Disposition
Tissue: Site / Finding, Severity

MICROSCOPIC EXAMINATION

Disposition: NORMAL

BRAIN, AMYGDALOID BODY
BRAIN, BASAL NUCLEI/STRIATUM
BRAIN, CEREBELLUM
BRAIN, CEREBRAL CORTEX
BRAIN, HIPPOCAMPUS
BRAIN, HYPOTHALAMUS
BRAIN, MEDULLA OBLONGATA
BRAIN, MENINGES
BRAIN, MIDBRAIN
BRAIN, OLFACTORY BULB
BRAIN, PONS
BRAIN, THALAMUS
BRAIN, VENTRICULAR SYSTEM
BRAIN, WHITE MATTER
EYE
GANGLION, DORSAL ROOT, CERVICAL
GANGLION, DORSAL ROOT, LUMBAR
GANGLION, DORSAL ROOT, THORACIC
HEART
KIDNEY
LUNG
LYMPH NODE, ILIAC
LYMPH NODE, MANDIBULAR
LYMPH NODE, MESENTERIC
MUSCLE, BICEPS FEMORIS
MUSCLE, GASTROCNEMIUS
NERVE ROOT, SPINAL, CERVICAL
NERVE ROOT, SPINAL, THORACIC
NERVE, OPTIC
NERVE, SCIATIC
NERVE, TIBIAL
OVARY
PANCREAS
SPINAL CORD, CERVICAL

Appendix 10

Individual Animal Report

Study ID: 02776-0018: CRL 2954-001 / U of Tex Southwestern Med UTSW.Gray-002
Study Title: A SINGLE DOSE TOXICITY STUDY OF AAV9/SURF1 ADMINISTERED BY INTRATHECAL INJECTION IN RATS

Animal: 1506 (Female)

Sacrifice: DAY 91
Group: 1FD91 / VEHICLE (0 VG)
Fate: SCHEDULED SACRIFICE / Time on Test: 90 / Cause(s) of Death:

Gross or Microscopic Finding
Disposition
Tissue: Site / Finding, Severity

SPINAL CORD, LUMBAR
SPINAL CORD, THORACIC
SPLEEN

Disposition: ABNORMAL/FINDING

LIVER
LIVER: INFILTRATE, MIXED, MINIMAL
NERVE ROOT, SPINAL, LUMBAR
NERVE ROOT, SPINAL, LUMBAR, EPINEURIUM: INFILTRATE, MONONUCLEAR CELLS, MINIMAL, FOCAL
THYMUS
THYMUS: INCREASED CELLULARITY, MINIMAL, FOCAL
COMMENT(S): AFFECTING EPITHELIAL CELLS

Appendix 10

Individual Animal Report

Study ID: 02776-0018: CRL 2954-001 / U of Tex Southwestern Med UTSW.Gray-002
Study Title: A SINGLE DOSE TOXICITY STUDY OF AAV9/SURF1 ADMINISTERED BY INTRATHECAL INJECTION IN RATS

Animal: 1507 (Female)

Sacrifice: DAY 91
Group: 1FD91 / VEHICLE (0 VG)
Fate: SCHEDULED SACRIFICE / Time on Test: 90 / Cause(s) of Death:

Gross or Microscopic Finding
Disposition
Tissue: Site / Finding, Severity

MICROSCOPIC EXAMINATION

Disposition: NORMAL

BRAIN, AMYGDALOID BODY
BRAIN, BASAL NUCLEI/STRIATUM
BRAIN, CEREBELLUM
BRAIN, CEREBRAL CORTEX
BRAIN, HIPPOCAMPUS
BRAIN, HYPOTHALAMUS
BRAIN, MEDULLA OBLONGATA
BRAIN, MENINGES
BRAIN, MIDBRAIN
BRAIN, OLFACTORY BULB
BRAIN, PONS
BRAIN, THALAMUS
BRAIN, VENTRICULAR SYSTEM
BRAIN, WHITE MATTER
GANGLION, DORSAL ROOT, CERVICAL
GANGLION, DORSAL ROOT, LUMBAR
GANGLION, DORSAL ROOT, THORACIC
HEART
KIDNEY
LIVER
LUNG
LYMPH NODE, ILIAC
LYMPH NODE, MANDIBULAR
MUSCLE, BICEPS FEMORIS
MUSCLE, GASTROCNEMIUS
NERVE ROOT, SPINAL, CERVICAL
NERVE ROOT, SPINAL, LUMBAR
NERVE ROOT, SPINAL, THORACIC
NERVE, OPTIC
NERVE, SCIATIC
NERVE, TIBIAL
OVARY
PANCREAS
SPINAL CORD, CERVICAL

Appendix 10

Individual Animal Report

Study ID: 02776-0018: CRL 2954-001 / U of Tex Southwestern Med UTSW.Gray-002
Study Title: A SINGLE DOSE TOXICITY STUDY OF AAV9/SURF1 ADMINISTERED BY INTRATHECAL INJECTION IN RATS

Animal: 1507 (Female)

Sacrifice: DAY 91
Group: 1FD91 / VEHICLE (0 VG)
Fate: SCHEDULED SACRIFICE / Time on Test: 90 / Cause(s) of Death:

Gross or Microscopic Finding
Disposition
Tissue: Site / Finding, Severity

SPINAL CORD, LUMBAR
SPINAL CORD, THORACIC
SPLEEN

Disposition: **ABNORMAL/FINDING**

EYE
EYE: RETINAL ROSETTE, MINIMAL
LYMPH NODE, MESENTERIC
LYMPH NODE, MESENTERIC: PIGMENT, MINIMAL
COMMENT(S): GREENISH GOLDEN BROWN AND GLOBULAR
THYMUS
THYMUS: INCREASED CELLULARITY, MINIMAL, FOCAL

Appendix 10

Individual Animal Report

Study ID: 02776-0018: CRL 2954-001 / U of Tex Southwestern Med UTSW.Gray-002
Study Title: A SINGLE DOSE TOXICITY STUDY OF AAV9/SURF1 ADMINISTERED BY INTRATHECAL INJECTION IN RATS

Animal: 1508 (Female)

Sacrifice: DAY 91
Group: 1FD91 / VEHICLE (0 VG)
Fate: SCHEDULED SACRIFICE / Time on Test: 90 / Cause(s) of Death:

Gross or Microscopic Finding
Disposition
Tissue: Site / Finding, Severity

MICROSCOPIC EXAMINATION

Disposition: **NORMAL**

BRAIN, AMYGDALOID BODY
BRAIN, BASAL NUCLEI/STRIATUM
BRAIN, CEREBELLUM
BRAIN, CEREBRAL CORTEX
BRAIN, HIPPOCAMPUS
BRAIN, HYPOTHALAMUS
BRAIN, MEDULLA OBLONGATA
BRAIN, MENINGES
BRAIN, MIDBRAIN
BRAIN, OLFACTORY BULB
BRAIN, PONS
BRAIN, THALAMUS
BRAIN, VENTRICULAR SYSTEM
BRAIN, WHITE MATTER
EYE
GANGLION, DORSAL ROOT, CERVICAL
GANGLION, DORSAL ROOT, LUMBAR
GANGLION, DORSAL ROOT, THORACIC
HEART
KIDNEY
LIVER
LYMPH NODE, ILIAC
LYMPH NODE, MANDIBULAR
LYMPH NODE, MESENTERIC
MUSCLE, BICEPS FEMORIS
MUSCLE, GASTROCNEMIUS
NERVE ROOT, SPINAL, CERVICAL
NERVE ROOT, SPINAL, LUMBAR
NERVE ROOT, SPINAL, THORACIC
NERVE, OPTIC
NERVE, SCIATIC
NERVE, TIBIAL
OVARY
PANCREAS

Appendix 10

Individual Animal Report

Study ID: 02776-0018: CRL 2954-001 / U of Tex Southwestern Med UTSW.Gray-002
Study Title: A SINGLE DOSE TOXICITY STUDY OF AAV9/SURF1 ADMINISTERED BY INTRATHECAL INJECTION IN RATS

Animal: 1508 (Female)

Sacrifice: DAY 91
Group: 1FD91 / VEHICLE (0 VG)
Fate: SCHEDULED SACRIFICE / Time on Test: 90 / Cause(s) of Death:

Gross or Microscopic Finding
Disposition
Tissue: Site / Finding, Severity

SPINAL CORD, CERVICAL
SPINAL CORD, LUMBAR
SPINAL CORD, THORACIC
SPLEEN
THYMUS

Disposition: ABNORMAL/FINDING

LUNG
LUNG, ALVEOLUS: INFILTRATE, MACROPHAGES, MINIMAL, FOCAL

Appendix 10

Individual Animal Report

Study ID: 02776-0018: CRL 2954-001 / U of Tex Southwestern Med UTSW.Gray-002
Study Title: A SINGLE DOSE TOXICITY STUDY OF AAV9/SURF1 ADMINISTERED BY INTRATHECAL INJECTION IN RATS

Animal: 1509 (Female)

Sacrifice: DAY 91
Group: 1FD91 / VEHICLE (0 VG)
Fate: SCHEDULED SACRIFICE / Time on Test: 91 / Cause(s) of Death:

Gross or Microscopic Finding
Disposition
Tissue: Site / Finding, Severity

MICROSCOPIC EXAMINATION

Disposition: NORMAL

BRAIN, AMYGDALOID BODY
BRAIN, BASAL NUCLEI/STRIATUM
BRAIN, CEREBELLUM
BRAIN, CEREBRAL CORTEX
BRAIN, HIPPOCAMPUS
BRAIN, HYPOTHALAMUS
BRAIN, MEDULLA OBLONGATA
BRAIN, MENINGES
BRAIN, MIDBRAIN
BRAIN, OLFACTORY BULB
BRAIN, PONS
BRAIN, THALAMUS
BRAIN, VENTRICULAR SYSTEM
BRAIN, WHITE MATTER
EYE
GANGLION, DORSAL ROOT, CERVICAL
GANGLION, DORSAL ROOT, LUMBAR
GANGLION, DORSAL ROOT, THORACIC
KIDNEY
LIVER
LUNG
LYMPH NODE, ILIAC
LYMPH NODE, MANDIBULAR
LYMPH NODE, MESENTERIC
MUSCLE, BICEPS FEMORIS
MUSCLE, GASTROCNEMIUS
NERVE ROOT, SPINAL, CERVICAL
NERVE ROOT, SPINAL, LUMBAR
NERVE ROOT, SPINAL, THORACIC
NERVE, OPTIC
NERVE, SCIATIC
NERVE, TIBIAL
OVARY
PANCREAS

Appendix 10

Individual Animal Report

Study ID: 02776-0018: CRL 2954-001 / U of Tex Southwestern Med UTSW.Gray-002
Study Title: A SINGLE DOSE TOXICITY STUDY OF AAV9/SURF1 ADMINISTERED BY INTRATHECAL INJECTION IN RATS

Animal: 1509 (Female)

Sacrifice: DAY 91
Group: 1FD91 / VEHICLE (0 VG)
Fate: SCHEDULED SACRIFICE / Time on Test: 91 / Cause(s) of Death:

Gross or Microscopic Finding
Disposition
Tissue: Site / Finding, Severity

SPINAL CORD, CERVICAL
SPINAL CORD, LUMBAR
SPINAL CORD, THORACIC
SPLEEN
THYMUS

Disposition: ABNORMAL/FINDING

HEART
HEART, MYOCARDIUM: INFILTRATE, MONONUCLEAR CELLS, MINIMAL, FOCAL

Appendix 10

Individual Animal Report

Study ID: 02776-0018: CRL 2954-001 / U of Tex Southwestern Med UTSW.Gray-002
Study Title: A SINGLE DOSE TOXICITY STUDY OF AAV9/SURF1 ADMINISTERED BY INTRATHECAL INJECTION IN RATS

Animal: 1510 (Female)

Sacrifice: DAY 91
Group: 1FD91 / VEHICLE (0 VG)
Fate: SCHEDULED SACRIFICE / Time on Test: 91 / Cause(s) of Death:

Gross or Microscopic Finding
Disposition
Tissue: Site / Finding, Severity

MICROSCOPIC EXAMINATION

Disposition: **NORMAL**

BRAIN, AMYGDALOID BODY
BRAIN, BASAL NUCLEI/STRIATUM
BRAIN, CEREBELLUM
BRAIN, CEREBRAL CORTEX
BRAIN, HIPPOCAMPUS
BRAIN, HYPOTHALAMUS
BRAIN, MEDULLA OBLONGATA
BRAIN, MENINGES
BRAIN, MIDBRAIN
BRAIN, OLFACTORY BULB
BRAIN, PONS
BRAIN, THALAMUS
BRAIN, VENTRICULAR SYSTEM
BRAIN, WHITE MATTER
EYE
GANGLION, DORSAL ROOT, CERVICAL
GANGLION, DORSAL ROOT, LUMBAR
GANGLION, DORSAL ROOT, THORACIC
KIDNEY
LUNG
LYMPH NODE, ILIAC
LYMPH NODE, MANDIBULAR
LYMPH NODE, MESENTERIC
MUSCLE, BICEPS FEMORIS
MUSCLE, GASTROCNEMIUS
NERVE ROOT, SPINAL, CERVICAL
NERVE ROOT, SPINAL, LUMBAR
NERVE ROOT, SPINAL, THORACIC
NERVE, OPTIC
NERVE, SCIATIC
NERVE, TIBIAL
OVARY
PANCREAS
SPINAL CORD, CERVICAL

Appendix 10

Individual Animal Report

Study ID: 02776-0018: CRL 2954-001 / U of Tex Southwestern Med UTSW.Gray-002
Study Title: A SINGLE DOSE TOXICITY STUDY OF AAV9/SURF1 ADMINISTERED BY INTRATHECAL INJECTION IN RATS

Animal: 1510 (Female)

Sacrifice: DAY 91
Group: 1FD91 / VEHICLE (0 VG)
Fate: SCHEDULED SACRIFICE / Time on Test: 91 / Cause(s) of Death:

Gross or Microscopic Finding
Disposition
Tissue: Site / Finding, Severity

SPINAL CORD, THORACIC
SPLEEN
THYMUS

Disposition: **ABNORMAL/FINDING**

HEART
HEART, MYOCARDIUM: DEGENERATION/NECROSIS, MINIMAL, MULTIFOCAL
HEART, MYOCARDIUM: INFILTRATE, MONONUCLEAR CELLS, MINIMAL, MULTIFOCAL
LIVER
LIVER: INFILTRATE, MIXED, MINIMAL

Disposition: **NOT PRESENT AT TRIM**

SPINAL CORD, LUMBAR

Appendix 10

Individual Animal Report

Study ID: 02776-0018: CRL 2954-001 / U of Tex Southwestern Med UTSW.Gray-002
Study Title: A SINGLE DOSE TOXICITY STUDY OF AAV9/SURF1 ADMINISTERED BY INTRATHECAL INJECTION IN RATS

Animal: 2506 (Female)

Sacrifice: DAY 91
Group: 2FD91 / AAV9/SURF1 LOW DOSE (0.28E12 VG)
Fate: SCHEDULED SACRIFICE / Time on Test: 90 / Cause(s) of Death:

Gross or Microscopic Finding
Disposition
Tissue: Site / Finding, Severity

MICROSCOPIC EXAMINATION

Disposition: **NORMAL**

BRAIN, AMYGDALOID BODY
BRAIN, BASAL NUCLEI/STRIATUM
BRAIN, CEREBELLUM
BRAIN, CEREBRAL CORTEX
BRAIN, HIPPOCAMPUS
BRAIN, HYPOTHALAMUS
BRAIN, MEDULLA OBLONGATA
BRAIN, MENINGES
BRAIN, MIDBRAIN
BRAIN, OLFACTORY BULB
BRAIN, PONS
BRAIN, THALAMUS
BRAIN, VENTRICULAR SYSTEM
BRAIN, WHITE MATTER
EYE
GANGLION, DORSAL ROOT, THORACIC
KIDNEY
LIVER
LUNG
LYMPH NODE, ILIAC
LYMPH NODE, MANDIBULAR
LYMPH NODE, MESENTERIC
MUSCLE, BICEPS FEMORIS
MUSCLE, GASTROCNEMIUS
NERVE ROOT, SPINAL, CERVICAL
NERVE ROOT, SPINAL, LUMBAR
NERVE ROOT, SPINAL, THORACIC
NERVE, OPTIC
NERVE, SCIATIC
NERVE, TIBIAL
OVARY
SPINAL CORD, CERVICAL
SPLEEN
THYMUS

Appendix 10

Individual Animal Report

Study ID: 02776-0018: CRL 2954-001 / U of Tex Southwestern Med UTSW.Gray-002
Study Title: A SINGLE DOSE TOXICITY STUDY OF AAV9/SURF1 ADMINISTERED BY INTRATHECAL INJECTION IN RATS

Animal: 2506 (Female)

Sacrifice: DAY 91
Group: 2FD91 / AAV9/SURF1 LOW DOSE (0.28E12 VG)
Fate: SCHEDULED SACRIFICE / Time on Test: 90 / Cause(s) of Death:

Gross or Microscopic Finding
Disposition
Tissue: Site / Finding, Severity

Disposition: **ABNORMAL/FINDING**

GANGLION, DORSAL ROOT, CERVICAL
GANGLION, DORSAL ROOT, CERVICAL: INFILTRATE, MONONUCLEAR CELLS, MINIMAL
GANGLION, DORSAL ROOT, LUMBAR
GANGLION, DORSAL ROOT, LUMBAR, GLIAL CELL: HYPERTROPHY/HYPERPLASIA, MINIMAL
HEART
HEART, MYOCARDIUM: DEGENERATION/NECROSIS, MINIMAL, MULTIFOCAL
HEART, MYOCARDIUM: INFILTRATE, MONONUCLEAR CELLS, MILD, MULTIFOCAL
PANCREAS
PANCREAS: INFILTRATE, MONONUCLEAR CELLS, MINIMAL, FOCAL
SPINAL CORD, LUMBAR
SPINAL CORD, LUMBAR: PIGMENT, HEMOSIDERIN, MINIMAL, LOCALLY EXTENSIVE
COMMENT(S): ASSOCIATED WITH LOCALLY EXTENSIVE INCREASED GLIAL CELLULARITY
SPINAL CORD, LUMBAR, GLIAL CELL: INCREASED CELLULARITY, MILD, LOCALLY EXTENSIVE
COMMENT(S): LIKELY REPRESENTING NEEDLE TRACT
SPINAL CORD, THORACIC
SPINAL CORD, THORACIC, WHITE MATTER: DEGENERATION, NERVE FIBER, MINIMAL
COMMENT(S): SEEN IN LONG SECTION AND EXACT LOCATION COULD NOT BE DETERMINED

Appendix 10

Individual Animal Report

Study ID: 02776-0018: CRL 2954-001 / U of Tex Southwestern Med UTSW.Gray-002
Study Title: A SINGLE DOSE TOXICITY STUDY OF AAV9/SURF1 ADMINISTERED BY INTRATHECAL INJECTION IN RATS

Animal: 2507 (Female)

Sacrifice: DAY 91
Group: 2FD91 / AAV9/SURF1 LOW DOSE (0.28E12 VG)
Fate: SCHEDULED SACRIFICE / Time on Test: 90 / Cause(s) of Death:

Gross or Microscopic Finding
Disposition
Tissue: Site / Finding, Severity

MICROSCOPIC EXAMINATION

Disposition: NORMAL

BRAIN, AMYGDALOID BODY
BRAIN, BASAL NUCLEI/STRIATUM
BRAIN, CEREBELLUM
BRAIN, CEREBRAL CORTEX
BRAIN, HIPPOCAMPUS
BRAIN, HYPOTHALAMUS
BRAIN, MEDULLA OBLONGATA
BRAIN, MENINGES
BRAIN, MIDBRAIN
BRAIN, OLFACTORY BULB
BRAIN, PONS
BRAIN, THALAMUS
BRAIN, VENTRICULAR SYSTEM
BRAIN, WHITE MATTER
EYE
GANGLION, DORSAL ROOT, CERVICAL
GANGLION, DORSAL ROOT, THORACIC
HEART
LIVER
LYMPH NODE, ILIAC
LYMPH NODE, MANDIBULAR
LYMPH NODE, MESENTERIC
MUSCLE, BICEPS FEMORIS
MUSCLE, GASTROCNEMIUS
NERVE ROOT, SPINAL, CERVICAL
NERVE ROOT, SPINAL, LUMBAR
NERVE ROOT, SPINAL, THORACIC
NERVE, OPTIC
OVARY
PANCREAS
SPINAL CORD, CERVICAL
SPINAL CORD, LUMBAR
SPINAL CORD, THORACIC
SPLEEN

Appendix 10

Individual Animal Report

Study ID: 02776-0018: CRL 2954-001 / U of Tex Southwestern Med UTSW.Gray-002
Study Title: A SINGLE DOSE TOXICITY STUDY OF AAV9/SURF1 ADMINISTERED BY INTRATHECAL INJECTION IN RATS

Animal: 2507 (Female)

Sacrifice: DAY 91
Group: 2FD91 / AAV9/SURF1 LOW DOSE (0.28E12 VG)
Fate: SCHEDULED SACRIFICE / Time on Test: 90 / Cause(s) of Death:

Gross or Microscopic Finding
Disposition
Tissue: Site / Finding, Severity

Disposition: **ABNORMAL/FINDING**

GANGLION, DORSAL ROOT, LUMBAR
GANGLION, DORSAL ROOT, LUMBAR, GLIAL CELL: HYPERTROPHY/HYPERPLASIA, MINIMAL
KIDNEY
KIDNEY, TUBULAR: CYST, MINIMAL, FOCAL
LUNG
LUNG, ALVEOLUS: INFILTRATE, MACROPHAGES, MINIMAL, MULTIFOCAL
NERVE, SCIATIC
NERVE, SCIATIC: DEGENERATION, NERVE FIBER, MINIMAL
NERVE, TIBIAL
NERVE, TIBIAL: DEGENERATION, NERVE FIBER, MINIMAL
THYMUS
THYMUS: INCREASED CELLULARITY, MINIMAL, FOCAL
COMMENT(S): AFFECTING EPITHELIAL CELLS

Appendix 10

Individual Animal Report

Study ID: 02776-0018: CRL 2954-001 / U of Tex Southwestern Med UTSW.Gray-002
Study Title: A SINGLE DOSE TOXICITY STUDY OF AAV9/SURF1 ADMINISTERED BY INTRATHECAL INJECTION IN RATS

Animal: 2508 (Female)

Sacrifice: DAY 91
Group: 2FD91 / AAV9/SURF1 LOW DOSE (0.28E12 VG)
Fate: SCHEDULED SACRIFICE / Time on Test: 90 / Cause(s) of Death:

Gross or Microscopic Finding
Disposition
Tissue: Site / Finding, Severity

MICROSCOPIC EXAMINATION

Disposition: **NORMAL**

BRAIN, AMYGDALOID BODY
BRAIN, BASAL NUCLEI/STRIATUM
BRAIN, CEREBELLUM
BRAIN, CEREBRAL CORTEX
BRAIN, HIPPOCAMPUS
BRAIN, HYPOTHALAMUS
BRAIN, MEDULLA OBLONGATA
BRAIN, MENINGES
BRAIN, MIDBRAIN
BRAIN, OLFACTORY BULB
BRAIN, PONS
BRAIN, THALAMUS
BRAIN, VENTRICULAR SYSTEM
BRAIN, WHITE MATTER
EYE
GANGLION, DORSAL ROOT, CERVICAL
GANGLION, DORSAL ROOT, THORACIC
KIDNEY
LIVER
LUNG
LYMPH NODE, ILIAC
LYMPH NODE, MANDIBULAR
LYMPH NODE, MESENTERIC
MUSCLE, BICEPS FEMORIS
MUSCLE, GASTROCNEMIUS
NERVE ROOT, SPINAL, CERVICAL
NERVE ROOT, SPINAL, LUMBAR
NERVE ROOT, SPINAL, THORACIC
NERVE, OPTIC
NERVE, TIBIAL
OVARY
PANCREAS
SPINAL CORD, CERVICAL
SPINAL CORD, LUMBAR

Appendix 10

Individual Animal Report

Study ID: 02776-0018: CRL 2954-001 / U of Tex Southwestern Med UTSW.Gray-002
Study Title: A SINGLE DOSE TOXICITY STUDY OF AAV9/SURF1 ADMINISTERED BY INTRATHECAL INJECTION IN RATS

Animal: 2508 (Female)

Sacrifice: DAY 91
Group: 2FD91 / AAV9/SURF1 LOW DOSE (0.28E12 VG)
Fate: SCHEDULED SACRIFICE / Time on Test: 90 / Cause(s) of Death:

Gross or Microscopic Finding
Disposition
Tissue: Site / Finding, Severity

SPINAL CORD, THORACIC
SPLEEN

Disposition: **ABNORMAL/FINDING**

GANGLION, DORSAL ROOT, LUMBAR
GANGLION, DORSAL ROOT, LUMBAR, GLIAL CELL: HYPERTROPHY/HYPERPLASIA, MINIMAL
HEART
HEART, MYOCARDIUM: DEGENERATION/NECROSIS, MINIMAL, MULTIFOCAL
HEART, MYOCARDIUM: INFILTRATE, MONONUCLEAR CELLS, MINIMAL, MULTIFOCAL
NERVE, SCIATIC
NERVE, SCIATIC: DEGENERATION, NERVE FIBER, MINIMAL
THYMUS
THYMUS: INCREASED CELLULARITY, MINIMAL, MULTIFOCAL
COMMENT(S): AFFECTING EPITHELIAL CELLS

Appendix 10

Individual Animal Report

Study ID: 02776-0018: CRL 2954-001 / U of Tex Southwestern Med UTSW.Gray-002
Study Title: A SINGLE DOSE TOXICITY STUDY OF AAV9/SURF1 ADMINISTERED BY INTRATHECAL INJECTION IN RATS

Animal: 2509 (Female)

Sacrifice: DAY 91
Group: 2FD91 / AAV9/SURF1 LOW DOSE (0.28E12 VG)
Fate: SCHEDULED SACRIFICE / Time on Test: 91 / Cause(s) of Death:

Gross or Microscopic Finding
Disposition
Tissue: Site / Finding, Severity

MICROSCOPIC EXAMINATION

Disposition: **NORMAL**

BRAIN, AMYGDALOID BODY
BRAIN, BASAL NUCLEI/STRIATUM
BRAIN, CEREBELLUM
BRAIN, CEREBRAL CORTEX
BRAIN, HIPPOCAMPUS
BRAIN, HYPOTHALAMUS
BRAIN, MENINGES
BRAIN, MIDBRAIN
BRAIN, OLFACTORY BULB
BRAIN, PONS
BRAIN, THALAMUS
BRAIN, VENTRICULAR SYSTEM
BRAIN, WHITE MATTER
EYE
GANGLION, DORSAL ROOT, CERVICAL
GANGLION, DORSAL ROOT, LUMBAR
GANGLION, DORSAL ROOT, THORACIC
HEART
KIDNEY
LIVER
LUNG
LYMPH NODE, ILIAC
LYMPH NODE, MANDIBULAR
LYMPH NODE, MESENTERIC
MUSCLE, BICEPS FEMORIS
MUSCLE, GASTROCNEMIUS
NERVE ROOT, SPINAL, CERVICAL
NERVE ROOT, SPINAL, LUMBAR
NERVE ROOT, SPINAL, THORACIC
NERVE, SCIATIC
NERVE, TIBIAL
OVARY
PANCREAS
SPINAL CORD, CERVICAL

Appendix 10

Individual Animal Report

Study ID: 02776-0018: CRL 2954-001 / U of Tex Southwestern Med UTSW.Gray-002
Study Title: A SINGLE DOSE TOXICITY STUDY OF AAV9/SURF1 ADMINISTERED BY INTRATHECAL INJECTION IN RATS

Animal: 2509 (Female)

Sacrifice: DAY 91
Group: 2FD91 / AAV9/SURF1 LOW DOSE (0.28E12 VG)
Fate: SCHEDULED SACRIFICE / Time on Test: 91 / Cause(s) of Death:

Gross or Microscopic Finding
Disposition
Tissue: Site / Finding, Severity

SPINAL CORD, LUMBAR
SPINAL CORD, THORACIC
SPLEEN
THYMUS

Disposition: **ABNORMAL/FINDING**

BRAIN, MEDULLA OBLONGATA
BRAIN, MEDULLA OBLONGATA, WHITE MATTER: DEGENERATION, NERVE FIBER, MINIMAL
COMMENT(S): PYRAMIDAL TRACT

Disposition: **UNABLE TO OBTAIN SPECIMEN**

NERVE, OPTIC

Appendix 10

Individual Animal Report

Study ID: 02776-0018: CRL 2954-001 / U of Tex Southwestern Med UTSW.Gray-002
Study Title: A SINGLE DOSE TOXICITY STUDY OF AAV9/SURF1 ADMINISTERED BY INTRATHECAL INJECTION IN RATS

Animal: 2510 (Female)

Sacrifice: DAY 91
Group: 2FD91 / AAV9/SURF1 LOW DOSE (0.28E12 VG)
Fate: SCHEDULED SACRIFICE / Time on Test: 91 / Cause(s) of Death:

Gross or Microscopic Finding
Disposition
Tissue: Site / Finding, Severity

MICROSCOPIC EXAMINATION

Disposition: NORMAL

BRAIN, AMYGDALOID BODY
BRAIN, BASAL NUCLEI/STRIATUM
BRAIN, CEREBELLUM
BRAIN, CEREBRAL CORTEX
BRAIN, HIPPOCAMPUS
BRAIN, HYPOTHALAMUS
BRAIN, MEDULLA OBLONGATA
BRAIN, MENINGES
BRAIN, OLFACTORY BULB
BRAIN, PONS
BRAIN, THALAMUS
BRAIN, VENTRICULAR SYSTEM
BRAIN, WHITE MATTER
EYE
KIDNEY
LUNG
LYMPH NODE, ILIAC
LYMPH NODE, MANDIBULAR
LYMPH NODE, MESENTERIC
MUSCLE, BICEPS FEMORIS
MUSCLE, GASTROCNEMIUS
NERVE ROOT, SPINAL, CERVICAL
NERVE ROOT, SPINAL, THORACIC
NERVE, OPTIC
NERVE, SCIATIC
NERVE, TIBIAL
OVARY
PANCREAS
SPINAL CORD, CERVICAL
SPINAL CORD, LUMBAR
SPLEEN
THYMUS

Appendix 10

Individual Animal Report

Study ID: 02776-0018: CRL 2954-001 / U of Tex Southwestern Med UTSW.Gray-002
Study Title: A SINGLE DOSE TOXICITY STUDY OF AAV9/SURF1 ADMINISTERED BY INTRATHECAL INJECTION IN RATS

Animal: 2510 (Female)

Sacrifice: DAY 91
Group: 2FD91 / AAV9/SURF1 LOW DOSE (0.28E12 VG)
Fate: SCHEDULED SACRIFICE / Time on Test: 91 / Cause(s) of Death:

Gross or Microscopic Finding
Disposition
Tissue: Site / Finding, Severity

Disposition: ABNORMAL/FINDING

BRAIN, MIDBRAIN
BRAIN, MIDBRAIN, PINEAL GLAND: INFILTRATE, MONONUCLEAR CELLS, MINIMAL, FOCAL
GANGLION, DORSAL ROOT, CERVICAL
GANGLION, DORSAL ROOT, CERVICAL, GLIAL CELL: HYPERTROPHY/HYPERPLASIA, MINIMAL
GANGLION, DORSAL ROOT, LUMBAR
GANGLION, DORSAL ROOT, LUMBAR, GLIAL CELL: HYPERTROPHY/HYPERPLASIA, MINIMAL
GANGLION, DORSAL ROOT, THORACIC
GANGLION, DORSAL ROOT, THORACIC: INFILTRATE, MONONUCLEAR CELLS, MINIMAL
HEART
HEART, MYOCARDIUM: DEGENERATION/NECROSIS, MINIMAL, MULTIFOCAL
HEART, MYOCARDIUM: INFILTRATE, MONONUCLEAR CELLS, MINIMAL, MULTIFOCAL
LIVER
LIVER: INFILTRATE, MIXED, MINIMAL
NERVE ROOT, SPINAL, LUMBAR
NERVE ROOT, SPINAL, LUMBAR, NERVE ROOT, VENTRAL: DEGENERATION, NERVE FIBER, MINIMAL
SPINAL CORD, THORACIC
SPINAL CORD, THORACIC, WHITE MATTER: DEGENERATION, NERVE FIBER, MINIMAL
COMMENT(S): SEEN IN LONG SECTION AND EXACT LOCATION COULD NOT BE DETERMINED

Appendix 10

Individual Animal Report

Study ID: 02776-0018: CRL 2954-001 / U of Tex Southwestern Med UTSW.Gray-002
Study Title: A SINGLE DOSE TOXICITY STUDY OF AAV9/SURF1 ADMINISTERED BY INTRATHECAL INJECTION IN RATS

Animal: 3506 (Female)

Sacrifice: DAY 91
Group: 3FD91 / AAV9/SURF1 MID DOSE (0.83E12 VG)
Fate: SCHEDULED SACRIFICE / Time on Test: 91 / Cause(s) of Death:

Gross or Microscopic Finding
Disposition
Tissue: Site / Finding, Severity

MICROSCOPIC EXAMINATION

Disposition: NORMAL

BRAIN, AMYGDALOID BODY
BRAIN, BASAL NUCLEI/STRIATUM
BRAIN, CEREBELLUM
BRAIN, CEREBRAL CORTEX
BRAIN, HIPPOCAMPUS
BRAIN, HYPOTHALAMUS
BRAIN, MEDULLA OBLONGATA
BRAIN, OLFACTORY BULB
BRAIN, PONS
BRAIN, THALAMUS
BRAIN, VENTRICULAR SYSTEM
BRAIN, WHITE MATTER
EYE
GANGLION, DORSAL ROOT, CERVICAL
KIDNEY
LIVER
LUNG
LYMPH NODE, ILIAC
LYMPH NODE, MANDIBULAR
LYMPH NODE, MESENTERIC
MUSCLE, BICEPS FEMORIS
MUSCLE, GASTROCNEMIUS
NERVE ROOT, SPINAL, CERVICAL
NERVE ROOT, SPINAL, THORACIC
NERVE, OPTIC
OVARY
PANCREAS
SPINAL CORD, CERVICAL
SPLEEN
THYMUS

Disposition: ABNORMAL/FINDING

BRAIN, MENINGES
BRAIN, MENINGES: INFILTRATE, MONONUCLEAR CELLS, MINIMAL, FOCAL

Appendix 10

Individual Animal Report

Study ID: 02776-0018: CRL 2954-001 / U of Tex Southwestern Med UTSW.Gray-002
Study Title: A SINGLE DOSE TOXICITY STUDY OF AAV9/SURF1 ADMINISTERED BY INTRATHECAL INJECTION IN RATS

Animal: 3506 (Female)

Sacrifice: DAY 91
Group: 3FD91 / AAV9/SURF1 MID DOSE (0.83E12 VG)
Fate: SCHEDULED SACRIFICE / Time on Test: 91 / Cause(s) of Death:

Gross or Microscopic Finding
Disposition
Tissue: Site / Finding, Severity

BRAIN, MIDBRAIN
BRAIN, MIDBRAIN, PINEAL GLAND: INFILTRATE, MONONUCLEAR CELLS, MINIMAL, FOCAL
GANGLION, DORSAL ROOT, LUMBAR
GANGLION, DORSAL ROOT, LUMBAR: INFILTRATE, MONONUCLEAR CELLS, MINIMAL
GANGLION, DORSAL ROOT, LUMBAR, GLIAL CELL: HYPERTROPHY/HYPERPLASIA, MINIMAL
GANGLION, DORSAL ROOT, THORACIC
GANGLION, DORSAL ROOT, THORACIC: INFILTRATE, MONONUCLEAR CELLS, MINIMAL, FOCAL
HEART
HEART, MYOCARDIUM: DEGENERATION/NECROSIS, MINIMAL, MULTIFOCAL
HEART, MYOCARDIUM: FIBROSIS, MINIMAL, MULTIFOCAL
HEART, MYOCARDIUM: INFILTRATE, MONONUCLEAR CELLS, MILD, MULTIFOCAL
NERVE ROOT, SPINAL, LUMBAR
NERVE ROOT, SPINAL, LUMBAR, NERVE ROOT, VENTRAL: DEGENERATION, NERVE FIBER, MILD
NERVE, SCIATIC
NERVE, SCIATIC: DEGENERATION, NERVE FIBER, MINIMAL
NERVE, TIBIAL
NERVE, TIBIAL: DEGENERATION, NERVE FIBER, MINIMAL
SPINAL CORD, LUMBAR
SPINAL CORD, LUMBAR, NERVE ROOT, SPINAL: DEGENERATION, NERVE FIBER, MINIMAL
SPINAL CORD, THORACIC
SPINAL CORD, THORACIC, WHITE MATTER: DEGENERATION, NERVE FIBER, MINIMAL, DORSAL

Appendix 10

Individual Animal Report

Study ID: 02776-0018: CRL 2954-001 / U of Tex Southwestern Med UTSW.Gray-002
Study Title: A SINGLE DOSE TOXICITY STUDY OF AAV9/SURF1 ADMINISTERED BY INTRATHECAL INJECTION IN RATS

Animal: 3507 (Female)

Sacrifice: DAY 91
Group: 3FD91 / AAV9/SURF1 MID DOSE (0.83E12 VG)
Fate: SCHEDULED SACRIFICE / Time on Test: 91 / Cause(s) of Death:

Gross or Microscopic Finding
Disposition
Tissue: Site / Finding, Severity

MICROSCOPIC EXAMINATION

Disposition: NORMAL

BRAIN, AMYGDALOID BODY
BRAIN, BASAL NUCLEI/STRIATUM
BRAIN, CEREBELLUM
BRAIN, CEREBRAL CORTEX
BRAIN, HIPPOCAMPUS
BRAIN, HYPOTHALAMUS
BRAIN, MEDULLA OBLONGATA
BRAIN, MENINGES
BRAIN, MIDBRAIN
BRAIN, OLFACTORY BULB
BRAIN, PONS
BRAIN, THALAMUS
BRAIN, VENTRICULAR SYSTEM
BRAIN, WHITE MATTER
EYE
GANGLION, DORSAL ROOT, CERVICAL
GANGLION, DORSAL ROOT, THORACIC
KIDNEY
LIVER
LUNG
LYMPH NODE, ILIAC
LYMPH NODE, MANDIBULAR
LYMPH NODE, MESENTERIC
MUSCLE, BICEPS FEMORIS
MUSCLE, GASTROCNEMIUS
NERVE ROOT, SPINAL, CERVICAL
NERVE ROOT, SPINAL, THORACIC
NERVE, OPTIC
NERVE, SCIATIC
NERVE, TIBIAL
OVARY
PANCREAS
SPINAL CORD, CERVICAL
SPINAL CORD, THORACIC

Appendix 10

Individual Animal Report

Study ID: 02776-0018: CRL 2954-001 / U of Tex Southwestern Med UTSW.Gray-002
Study Title: A SINGLE DOSE TOXICITY STUDY OF AAV9/SURF1 ADMINISTERED BY INTRATHECAL INJECTION IN RATS

Animal: 3507 (Female)

Sacrifice: DAY 91
Group: 3FD91 / AAV9/SURF1 MID DOSE (0.83E12 VG)
Fate: SCHEDULED SACRIFICE / Time on Test: 91 / Cause(s) of Death:

Gross or Microscopic Finding
Disposition
Tissue: Site / Finding, Severity

SPLEEN
THYMUS

Disposition: ABNORMAL/FINDING

GANGLION, DORSAL ROOT, LUMBAR
GANGLION, DORSAL ROOT, LUMBAR: INFILTRATE, MONONUCLEAR CELLS, MINIMAL
GANGLION, DORSAL ROOT, LUMBAR, GLIAL CELL: HYPERTROPHY/HYPERPLASIA, MINIMAL
HEART
HEART, MYOCARDIUM: DEGENERATION/NECROSIS, MINIMAL, MULTIFOCAL
HEART, MYOCARDIUM: FIBROSIS, MILD, MULTIFOCAL
HEART, MYOCARDIUM: INFILTRATE, MONONUCLEAR CELLS, MILD, MULTIFOCAL
NERVE ROOT, SPINAL, LUMBAR
NERVE ROOT, SPINAL, LUMBAR, EPINEURIUM: INFILTRATE, MONONUCLEAR CELLS, MINIMAL, FOCAL
SPINAL CORD, LUMBAR
SPINAL CORD, LUMBAR, NERVE ROOT, SPINAL: DEGENERATION, NERVE FIBER, MINIMAL

Appendix 10

Individual Animal Report

Study ID: 02776-0018: CRL 2954-001 / U of Tex Southwestern Med UTSW.Gray-002
Study Title: A SINGLE DOSE TOXICITY STUDY OF AAV9/SURF1 ADMINISTERED BY INTRATHECAL INJECTION IN RATS

Animal: 3508 (Female)

Sacrifice: DAY 91
Group: 3FD91 / AAV9/SURF1 MID DOSE (0.83E12 VG)
Fate: SCHEDULED SACRIFICE / Time on Test: 91 / Cause(s) of Death:

Gross or Microscopic Finding
Disposition
Tissue: Site / Finding, Severity

MICROSCOPIC EXAMINATION

Disposition: NORMAL

BRAIN, AMYGDALOID BODY
BRAIN, BASAL NUCLEI/STRIATUM
BRAIN, CEREBELLUM
BRAIN, CEREBRAL CORTEX
BRAIN, HIPPOCAMPUS
BRAIN, HYPOTHALAMUS
BRAIN, MEDULLA OBLONGATA
BRAIN, MENINGES
BRAIN, MIDBRAIN
BRAIN, OLFACTORY BULB
BRAIN, PONS
BRAIN, THALAMUS
BRAIN, VENTRICULAR SYSTEM
BRAIN, WHITE MATTER
EYE
KIDNEY
LIVER
LUNG
LYMPH NODE, ILIAC
LYMPH NODE, MANDIBULAR
LYMPH NODE, MESENTERIC
MUSCLE, BICEPS FEMORIS
MUSCLE, GASTROCNEMIUS
NERVE ROOT, SPINAL, CERVICAL
NERVE ROOT, SPINAL, THORACIC
NERVE, OPTIC
OVARY
PANCREAS
SPINAL CORD, CERVICAL
SPINAL CORD, THORACIC
SPLEEN
THYMUS

Appendix 10

Individual Animal Report

Study ID: 02776-0018: CRL 2954-001 / U of Tex Southwestern Med UTSW.Gray-002
Study Title: A SINGLE DOSE TOXICITY STUDY OF AAV9/SURF1 ADMINISTERED BY INTRATHECAL INJECTION IN RATS

Animal: 3508 (Female)

Sacrifice: DAY 91
Group: 3FD91 / AAV9/SURF1 MID DOSE (0.83E12 VG)
Fate: SCHEDULED SACRIFICE / Time on Test: 91 / Cause(s) of Death:

Gross or Microscopic Finding
Disposition
Tissue: Site / Finding, Severity

Disposition: **ABNORMAL/FINDING**

GANGLION, DORSAL ROOT, CERVICAL
GANGLION, DORSAL ROOT, CERVICAL, GLIAL CELL: HYPERTROPHY/HYPERPLASIA, MINIMAL
GANGLION, DORSAL ROOT, LUMBAR
GANGLION, DORSAL ROOT, LUMBAR, GLIAL CELL: HYPERTROPHY/HYPERPLASIA, MINIMAL
GANGLION, DORSAL ROOT, THORACIC
GANGLION, DORSAL ROOT, THORACIC, GLIAL CELL: HYPERTROPHY/HYPERPLASIA, MINIMAL
HEART
HEART, MYOCARDIUM: DEGENERATION/NECROSIS, MINIMAL, MULTIFOCAL
HEART, MYOCARDIUM: FIBROSIS, MILD, MULTIFOCAL
HEART, MYOCARDIUM: INFILTRATE, MONONUCLEAR CELLS, MILD, MULTIFOCAL
NERVE ROOT, SPINAL, LUMBAR
NERVE ROOT, SPINAL, LUMBAR, NERVE ROOT, VENTRAL: DEGENERATION, NERVE FIBER, MILD
COMMENT(S): AFFECTING SIDE WITH NO DORSAL ROOT GANGLION PRESENT. PRESUMED TO BE VENTRAL NERVE ROOT DUE TO APPEARANCE OF NERVE FIBERS
NERVE, SCIATIC
NERVE, SCIATIC: DEGENERATION, NERVE FIBER, MINIMAL
NERVE, TIBIAL
NERVE, TIBIAL: DEGENERATION, NERVE FIBER, MINIMAL
SPINAL CORD, LUMBAR
SPINAL CORD, LUMBAR, NERVE ROOT, SPINAL: DEGENERATION, NERVE FIBER, MILD

Appendix 10

Individual Animal Report

Study ID: 02776-0018: CRL 2954-001 / U of Tex Southwestern Med UTSW.Gray-002
Study Title: A SINGLE DOSE TOXICITY STUDY OF AAV9/SURF1 ADMINISTERED BY INTRATHECAL INJECTION IN RATS

Animal: 3509 (Female)

Sacrifice: DAY 91
Group: 3FD91 / AAV9/SURF1 MID DOSE (0.83E12 VG)
Fate: SCHEDULED SACRIFICE / Time on Test: 91 / Cause(s) of Death:

Gross or Microscopic Finding
Disposition
Tissue: Site / Finding, Severity

MICROSCOPIC EXAMINATION

Disposition: NORMAL

BRAIN, AMYGDALOID BODY
BRAIN, BASAL NUCLEI/STRIATUM
BRAIN, CEREBELLUM
BRAIN, CEREBRAL CORTEX
BRAIN, HIPPOCAMPUS
BRAIN, HYPOTHALAMUS
BRAIN, MEDULLA OBLONGATA
BRAIN, MENINGES
BRAIN, MIDBRAIN
BRAIN, OLFACTORY BULB
BRAIN, PONS
BRAIN, THALAMUS
BRAIN, VENTRICULAR SYSTEM
BRAIN, WHITE MATTER
EYE
KIDNEY
LIVER
LUNG
LYMPH NODE, ILIAC
LYMPH NODE, MANDIBULAR
LYMPH NODE, MESENTERIC
MUSCLE, BICEPS FEMORIS
MUSCLE, GASTROCNEMIUS
NERVE ROOT, SPINAL, CERVICAL
NERVE ROOT, SPINAL, THORACIC
NERVE, OPTIC
OVARY
PANCREAS
SPINAL CORD, CERVICAL
SPLEEN

Disposition: ABNORMAL/FINDING

GANGLION, DORSAL ROOT, CERVICAL
GANGLION, DORSAL ROOT, CERVICAL, GLIAL CELL: HYPERTROPHY/HYPERPLASIA, MINIMAL

Appendix 10

Individual Animal Report

Study ID: 02776-0018: CRL 2954-001 / U of Tex Southwestern Med UTSW.Gray-002
Study Title: A SINGLE DOSE TOXICITY STUDY OF AAV9/SURF1 ADMINISTERED BY INTRATHECAL INJECTION IN RATS

Animal: 3509 (Female)

Sacrifice: DAY 91
Group: 3FD91 / AAV9/SURF1 MID DOSE (0.83E12 VG)
Fate: SCHEDULED SACRIFICE / Time on Test: 91 / Cause(s) of Death:

Gross or Microscopic Finding
Disposition
Tissue: Site / Finding, Severity

GANGLION, DORSAL ROOT, LUMBAR
GANGLION, DORSAL ROOT, LUMBAR: INFILTRATE, MONONUCLEAR CELLS, MINIMAL
GANGLION, DORSAL ROOT, LUMBAR, GLIAL CELL: HYPERTROPHY/HYPERPLASIA, MINIMAL
GANGLION, DORSAL ROOT, THORACIC
GANGLION, DORSAL ROOT, THORACIC, GLIAL CELL: HYPERTROPHY/HYPERPLASIA, MINIMAL
HEART
HEART, MYOCARDIUM: DEGENERATION/NECROSIS, MINIMAL, MULTIFOCAL
HEART, MYOCARDIUM: FIBROSIS, MINIMAL, MULTIFOCAL
HEART, MYOCARDIUM: INFILTRATE, MONONUCLEAR CELLS, MILD, MULTIFOCAL
NERVE ROOT, SPINAL, LUMBAR
NERVE ROOT, SPINAL, LUMBAR, EPINEURIUM: INFILTRATE, MONONUCLEAR CELLS, MINIMAL, FOCAL
NERVE, SCIATIC
NERVE, SCIATIC: DEGENERATION, NERVE FIBER, MILD
NERVE, SCIATIC, SCHWANN CELL: HYPERTROPHY/HYPERPLASIA, MINIMAL
NERVE, TIBIAL
NERVE, TIBIAL: DEGENERATION, NERVE FIBER, MILD
NERVE, TIBIAL, SCHWANN CELL: HYPERTROPHY/HYPERPLASIA, MILD
SPINAL CORD, LUMBAR
SPINAL CORD, LUMBAR, MENINGES: INFILTRATE, MONONUCLEAR CELLS, MINIMAL, MULTIFOCAL
SPINAL CORD, LUMBAR, MENINGES: MINERALIZATION, MINIMAL, FOCAL
SPINAL CORD, LUMBAR, NERVE ROOT, SPINAL: DEGENERATION, NERVE FIBER, MARKED
COMMENT(S): SPINAL NERVES VARIABLY AFFECTED AND THOSE MOST AFFECTED HAVE A CONCOMITANT INCREASE IN SCHWANN CELLS
SPINAL CORD, THORACIC
SPINAL CORD, THORACIC, WHITE MATTER: DEGENERATION, NERVE FIBER, MILD, DORSAL
THYMUS
THYMUS: INCREASED CELLULARITY, MINIMAL, FOCAL
COMMENT(S): AFFECTING EPITHELIAL CELLS

Appendix 10

Individual Animal Report

Study ID: 02776-0018: CRL 2954-001 / U of Tex Southwestern Med UTSW.Gray-002
Study Title: A SINGLE DOSE TOXICITY STUDY OF AAV9/SURF1 ADMINISTERED BY INTRATHECAL INJECTION IN RATS

Animal: 3510 (Female)

Sacrifice: DAY 91
Group: 3FD91 / AAV9/SURF1 MID DOSE (0.83E12 VG)
Fate: SCHEDULED SACRIFICE / Time on Test: 91 / Cause(s) of Death:

Gross or Microscopic Finding
Disposition
Tissue: Site / Finding, Severity

MICROSCOPIC EXAMINATION

Disposition: NORMAL

BRAIN, AMYGDALOID BODY
BRAIN, BASAL NUCLEI/STRIATUM
BRAIN, CEREBELLUM
BRAIN, CEREBRAL CORTEX
BRAIN, HIPPOCAMPUS
BRAIN, HYPOTHALAMUS
BRAIN, MENINGES
BRAIN, MIDBRAIN
BRAIN, OLFACTORY BULB
BRAIN, PONS
BRAIN, THALAMUS
BRAIN, VENTRICULAR SYSTEM
BRAIN, WHITE MATTER
EYE
GANGLION, DORSAL ROOT, LUMBAR
KIDNEY
LUNG
LYMPH NODE, ILIAC
LYMPH NODE, MANDIBULAR
LYMPH NODE, MESENTERIC
MUSCLE, BICEPS FEMORIS
MUSCLE, GASTROCNEMIUS
NERVE ROOT, SPINAL, CERVICAL
NERVE ROOT, SPINAL, LUMBAR
NERVE ROOT, SPINAL, THORACIC
NERVE, OPTIC
NERVE, SCIATIC
NERVE, TIBIAL
OVARY
PANCREAS
SPINAL CORD, CERVICAL
SPINAL CORD, LUMBAR
SPLEEN
THYMUS

Appendix 10

Individual Animal Report

Study ID: 02776-0018: CRL 2954-001 / U of Tex Southwestern Med UTSW.Gray-002
Study Title: A SINGLE DOSE TOXICITY STUDY OF AAV9/SURF1 ADMINISTERED BY INTRATHECAL INJECTION IN RATS

Animal: 3510 (Female)

Sacrifice: DAY 91
Group: 3FD91 / AAV9/SURF1 MID DOSE (0.83E12 VG)
Fate: SCHEDULED SACRIFICE / Time on Test: 91 / Cause(s) of Death:

Gross or Microscopic Finding
Disposition
Tissue: Site / Finding, Severity

Disposition: **ABNORMAL/FINDING**

BRAIN, MEDULLA OBLONGATA
BRAIN, MEDULLA OBLONGATA, WHITE MATTER: DEGENERATION, NERVE FIBER, MINIMAL
COMMENT(S): AFFECTING THE SPINAL TRACT OF THE TRIGEMINAL NERVE

GANGLION, DORSAL ROOT, CERVICAL
GANGLION, DORSAL ROOT, CERVICAL, GLIAL CELL: HYPERTROPHY/HYPERPLASIA, MINIMAL

GANGLION, DORSAL ROOT, THORACIC
GANGLION, DORSAL ROOT, THORACIC, GLIAL CELL: HYPERTROPHY/HYPERPLASIA, MINIMAL

HEART
HEART, MYOCARDIUM: INFILTRATE, MONONUCLEAR CELLS, MINIMAL, FOCAL

LIVER
LIVER: INFILTRATE, MIXED, MINIMAL
LIVER: NECROSIS, MINIMAL, MULTIFOCAL

SPINAL CORD, THORACIC
SPINAL CORD, THORACIC, WHITE MATTER: DEGENERATION, NERVE FIBER, MINIMAL
COMMENT(S): SEEN IN LONG SECTION AND EXACT LOCATION COULD NOT BE DETERMINED

Appendix 10

Individual Animal Report

Study ID: 02776-0018: CRL 2954-001 / U of Tex Southwestern Med UTSW.Gray-002
Study Title: A SINGLE DOSE TOXICITY STUDY OF AAV9/SURF1 ADMINISTERED BY INTRATHECAL INJECTION IN RATS

Animal: 4506 (Female)

Sacrifice: DAY 91
Group: 4FD91 / AAV9/SURF1 HIGH DOSE (2.49E12 VG)
Fate: SCHEDULED SACRIFICE / Time on Test: 91 / Cause(s) of Death:

Gross or Microscopic Finding
Disposition
Tissue: Site / Finding, Severity

MICROSCOPIC EXAMINATION

Disposition: NORMAL

BRAIN, AMYGDALOID BODY
BRAIN, BASAL NUCLEI/STRIATUM
BRAIN, CEREBELLUM
BRAIN, CEREBRAL CORTEX
BRAIN, HIPPOCAMPUS
BRAIN, HYPOTHALAMUS
BRAIN, MEDULLA OBLONGATA
BRAIN, MENINGES
BRAIN, MIDBRAIN
BRAIN, OLFACTORY BULB
BRAIN, PONS
BRAIN, THALAMUS
BRAIN, VENTRICULAR SYSTEM
BRAIN, WHITE MATTER
EYE
GANGLION, DORSAL ROOT, THORACIC
KIDNEY
LUNG
LYMPH NODE, ILIAC
LYMPH NODE, MANDIBULAR
LYMPH NODE, MESENTERIC
MUSCLE, BICEPS FEMORIS
MUSCLE, GASTROCNEMIUS
NERVE ROOT, SPINAL, CERVICAL
NERVE ROOT, SPINAL, THORACIC
NERVE, OPTIC
OVARY
PANCREAS
SPLEEN
THYMUS

Disposition: ABNORMAL/FINDING

GANGLION, DORSAL ROOT, CERVICAL
GANGLION, DORSAL ROOT, CERVICAL, GLIAL CELL: HYPERTROPHY/HYPERPLASIA, MINIMAL

Appendix 10

Individual Animal Report

Study ID: 02776-0018: CRL 2954-001 / U of Tex Southwestern Med UTSW.Gray-002
Study Title: A SINGLE DOSE TOXICITY STUDY OF AAV9/SURF1 ADMINISTERED BY INTRATHECAL INJECTION IN RATS

Animal: 4506 (Female)

Sacrifice: DAY 91
Group: 4FD91 / AAV9/SURF1 HIGH DOSE (2.49E12 VG)
Fate: SCHEDULED SACRIFICE / Time on Test: 91 / Cause(s) of Death:

Gross or Microscopic Finding
Disposition
Tissue: Site / Finding, Severity

GANGLION, DORSAL ROOT, LUMBAR
GANGLION, DORSAL ROOT, LUMBAR, GLIAL CELL: HYPERTROPHY/HYPERPLASIA, MINIMAL
HEART
HEART, MYOCARDIUM: DEGENERATION/NECROSIS, MINIMAL, MULTIFOCAL
HEART, MYOCARDIUM: INFILTRATE, MONONUCLEAR CELLS, MINIMAL, MULTIFOCAL
LIVER
LIVER: INFILTRATE, MIXED, MINIMAL
NERVE ROOT, SPINAL, LUMBAR
NERVE ROOT, SPINAL, LUMBAR, EPINEURIUM: INFILTRATE, MONONUCLEAR CELLS, MINIMAL, FOCAL
NERVE, SCIATIC
NERVE, SCIATIC: DEGENERATION, NERVE FIBER, MILD
NERVE, TIBIAL
NERVE, TIBIAL: DEGENERATION, NERVE FIBER, MILD
SPINAL CORD, CERVICAL
SPINAL CORD, CERVICAL, WHITE MATTER: DEGENERATION, NERVE FIBER, MINIMAL
COMMENT(S): SEEN IN LONG SECTION AND EXACT LOCATION COULD NOT BE DETERMINED
SPINAL CORD, LUMBAR
SPINAL CORD, LUMBAR, WHITE MATTER: DEGENERATION, NERVE FIBER, MINIMAL, DORSAL
SPINAL CORD, THORACIC
SPINAL CORD, THORACIC, WHITE MATTER: DEGENERATION, NERVE FIBER, MINIMAL, DORSAL

Appendix 10

Individual Animal Report

Study ID: 02776-0018: CRL 2954-001 / U of Tex Southwestern Med UTSW.Gray-002
Study Title: A SINGLE DOSE TOXICITY STUDY OF AAV9/SURF1 ADMINISTERED BY INTRATHECAL INJECTION IN RATS

Animal: 4507 (Female)

Sacrifice: DAY 91
Group: 4FD91 / AAV9/SURF1 HIGH DOSE (2.49E12 VG)
Fate: SCHEDULED SACRIFICE / Time on Test: 91 / Cause(s) of Death:

Gross or Microscopic Finding
Disposition
Tissue: Site / Finding, Severity

MICROSCOPIC EXAMINATION

Disposition: NORMAL

BRAIN, AMYGDALOID BODY
BRAIN, BASAL NUCLEI/STRIATUM
BRAIN, CEREBELLUM
BRAIN, CEREBRAL CORTEX
BRAIN, HIPPOCAMPUS
BRAIN, HYPOTHALAMUS
BRAIN, MENINGES
BRAIN, OLFACTORY BULB
BRAIN, PONS
BRAIN, THALAMUS
BRAIN, VENTRICULAR SYSTEM
BRAIN, WHITE MATTER
EYE
GANGLION, DORSAL ROOT, CERVICAL
KIDNEY
LIVER
LUNG
LYMPH NODE, ILIAC
LYMPH NODE, MANDIBULAR
LYMPH NODE, MESENTERIC
MUSCLE, BICEPS FEMORIS
MUSCLE, GASTROCNEMIUS
NERVE ROOT, SPINAL, CERVICAL
NERVE ROOT, SPINAL, THORACIC
NERVE, OPTIC
OVARY
PANCREAS
SPINAL CORD, CERVICAL
SPLEEN
THYMUS

Disposition: ABNORMAL/FINDING

BRAIN, MEDULLA OBLONGATA
BRAIN, MEDULLA OBLONGATA, WHITE MATTER: DEGENERATION, NERVE FIBER, MINIMAL
COMMENT(S): PYRAMIDAL TRACT

Appendix 10

Individual Animal Report

Study ID: 02776-0018: CRL 2954-001 / U of Tex Southwestern Med UTSW.Gray-002
Study Title: A SINGLE DOSE TOXICITY STUDY OF AAV9/SURF1 ADMINISTERED BY INTRATHECAL INJECTION IN RATS

Animal: 4507 (Female)

Sacrifice: DAY 91
Group: 4FD91 / AAV9/SURF1 HIGH DOSE (2.49E12 VG)
Fate: SCHEDULED SACRIFICE / Time on Test: 91 / Cause(s) of Death:

Gross or Microscopic Finding
Disposition
Tissue: Site / Finding, Severity

BRAIN, MIDBRAIN
BRAIN, MIDBRAIN, PINEAL GLAND: INFILTRATE, MONONUCLEAR CELLS, MINIMAL, FOCAL
GANGLION, DORSAL ROOT, LUMBAR
GANGLION, DORSAL ROOT, LUMBAR: INFILTRATE, MONONUCLEAR CELLS, MINIMAL
GANGLION, DORSAL ROOT, LUMBAR, GLIAL CELL: HYPERTROPHY/HYPERPLASIA, MINIMAL
GANGLION, DORSAL ROOT, THORACIC
GANGLION, DORSAL ROOT, THORACIC, GLIAL CELL: HYPERTROPHY/HYPERPLASIA, MINIMAL
HEART
HEART, MYOCARDIUM: DEGENERATION/NECROSIS, MINIMAL, MULTIFOCAL
HEART, MYOCARDIUM: INFILTRATE, MONONUCLEAR CELLS, MINIMAL, MULTIFOCAL
NERVE ROOT, SPINAL, LUMBAR
NERVE ROOT, SPINAL, LUMBAR, NERVE ROOT, DORSAL: DEGENERATION, NERVE FIBER, MINIMAL
NERVE, SCIATIC
NERVE, SCIATIC: DEGENERATION, NERVE FIBER, MILD
NERVE, TIBIAL
NERVE, TIBIAL: DEGENERATION, NERVE FIBER, MILD
SPINAL CORD, LUMBAR
SPINAL CORD, LUMBAR, NERVE ROOT, SPINAL: DEGENERATION, NERVE FIBER, MILD
SPINAL CORD, THORACIC
SPINAL CORD, THORACIC, WHITE MATTER: DEGENERATION, NERVE FIBER, MINIMAL, DORSAL

Appendix 10

Individual Animal Report

Study ID: 02776-0018: CRL 2954-001 / U of Tex Southwestern Med UTSW.Gray-002
Study Title: A SINGLE DOSE TOXICITY STUDY OF AAV9/SURF1 ADMINISTERED BY INTRATHECAL INJECTION IN RATS

Animal: 4508 (Female)

Sacrifice: DAY 91
Group: 4FD91 / AAV9/SURF1 HIGH DOSE (2.49E12 VG)
Fate: SCHEDULED SACRIFICE / Time on Test: 91 / Cause(s) of Death:

Gross or Microscopic Finding
Disposition
Tissue: Site / Finding, Severity

MICROSCOPIC EXAMINATION

Disposition: NORMAL

BRAIN, AMYGDALOID BODY
BRAIN, BASAL NUCLEI/STRIATUM
BRAIN, CEREBELLUM
BRAIN, CEREBRAL CORTEX
BRAIN, HIPPOCAMPUS
BRAIN, HYPOTHALAMUS
BRAIN, MEDULLA OBLONGATA
BRAIN, MENINGES
BRAIN, MIDBRAIN
BRAIN, OLFACTORY BULB
BRAIN, PONS
BRAIN, THALAMUS
BRAIN, VENTRICULAR SYSTEM
BRAIN, WHITE MATTER
EYE
GANGLION, DORSAL ROOT, CERVICAL
KIDNEY
LUNG
LYMPH NODE, ILIAC
LYMPH NODE, MANDIBULAR
LYMPH NODE, MESENTERIC
MUSCLE, BICEPS FEMORIS
MUSCLE, GASTROCNEMIUS
NERVE ROOT, SPINAL, CERVICAL
NERVE ROOT, SPINAL, THORACIC
NERVE, OPTIC
NERVE, SCIATIC
NERVE, TIBIAL
OVARY
PANCREAS
SPINAL CORD, CERVICAL
SPINAL CORD, LUMBAR
SPLEEN
THYMUS

Appendix 10

Individual Animal Report

Study ID: 02776-0018: CRL 2954-001 / U of Tex Southwestern Med UTSW.Gray-002
Study Title: A SINGLE DOSE TOXICITY STUDY OF AAV9/SURF1 ADMINISTERED BY INTRATHECAL INJECTION IN RATS

Animal: 4508 (Female)

Sacrifice: DAY 91
Group: 4FD91 / AAV9/SURF1 HIGH DOSE (2.49E12 VG)
Fate: SCHEDULED SACRIFICE / Time on Test: 91 / Cause(s) of Death:

Gross or Microscopic Finding
Disposition
Tissue: Site / Finding, Severity

Disposition: **ABNORMAL/FINDING**

GANGLION, DORSAL ROOT, LUMBAR
GANGLION, DORSAL ROOT, LUMBAR, GLIAL CELL: HYPERTROPHY/HYPERPLASIA, MINIMAL
GANGLION, DORSAL ROOT, THORACIC
GANGLION, DORSAL ROOT, THORACIC: INFILTRATE, MONONUCLEAR CELLS, MINIMAL, FOCAL
HEART
HEART, MYOCARDIUM: DEGENERATION/NECROSIS, MINIMAL, MULTIFOCAL
HEART, MYOCARDIUM: FIBROSIS, MINIMAL, FOCAL
HEART, MYOCARDIUM: INFILTRATE, MONONUCLEAR CELLS, MINIMAL, MULTIFOCAL
LIVER
LIVER: INFILTRATE, MIXED, MINIMAL
NERVE ROOT, SPINAL, LUMBAR
NERVE ROOT, SPINAL, LUMBAR, EPINEURIUM: INFILTRATE, MONONUCLEAR CELLS, MINIMAL, FOCAL
SPINAL CORD, THORACIC
SPINAL CORD, THORACIC, WHITE MATTER: DEGENERATION, NERVE FIBER, MINIMAL
COMMENT(S): SEEN IN LONG SECTION AND EXACT LOCATION COULD NOT BE DETERMINED

Appendix 10

Individual Animal Report

Study ID: 02776-0018: CRL 2954-001 / U of Tex Southwestern Med UTSW.Gray-002
Study Title: A SINGLE DOSE TOXICITY STUDY OF AAV9/SURF1 ADMINISTERED BY INTRATHECAL INJECTION IN RATS

Animal: 4509 (Female)

Sacrifice: DAY 91
Group: 4FD91 / AAV9/SURF1 HIGH DOSE (2.49E12 VG)
Fate: SCHEDULED SACRIFICE / Time on Test: 91 / Cause(s) of Death:

Gross or Microscopic Finding
Disposition
Tissue: Site / Finding, Severity

MICROSCOPIC EXAMINATION

Disposition: NORMAL

BRAIN, AMYGDALOID BODY
BRAIN, BASAL NUCLEI/STRIATUM
BRAIN, CEREBELLUM
BRAIN, CEREBRAL CORTEX
BRAIN, HIPPOCAMPUS
BRAIN, HYPOTHALAMUS
BRAIN, MENINGES
BRAIN, MIDBRAIN
BRAIN, OLFACTORY BULB
BRAIN, PONS
BRAIN, THALAMUS
BRAIN, VENTRICULAR SYSTEM
BRAIN, WHITE MATTER
EYE
KIDNEY
LUNG
LYMPH NODE, ILIAC
LYMPH NODE, MANDIBULAR
LYMPH NODE, MESENTERIC
MUSCLE, BICEPS FEMORIS
MUSCLE, GASTROCNEMIUS
NERVE ROOT, SPINAL, CERVICAL
NERVE ROOT, SPINAL, THORACIC
NERVE, OPTIC
OVARY
PANCREAS
SPINAL CORD, CERVICAL
SPINAL CORD, LUMBAR
SPLEEN
THYMUS

Disposition: ABNORMAL/FINDING

BRAIN, MEDULLA OBLONGATA

BRAIN, MEDULLA OBLONGATA, WHITE MATTER: DEGENERATION, NERVE FIBER, MINIMAL
COMMENT(S): PYRAMIDAL TRACT

Appendix 10

Individual Animal Report

Study ID: 02776-0018: CRL 2954-001 / U of Tex Southwestern Med UTSW.Gray-002
Study Title: A SINGLE DOSE TOXICITY STUDY OF AAV9/SURF1 ADMINISTERED BY INTRATHECAL INJECTION IN RATS

Animal: 4509 (Female)

Sacrifice: DAY 91
Group: 4FD91 / AAV9/SURF1 HIGH DOSE (2.49E12 VG)
Fate: SCHEDULED SACRIFICE / Time on Test: 91 / Cause(s) of Death:

Gross or Microscopic Finding
Disposition
Tissue: Site / Finding, Severity

GANGLION, DORSAL ROOT, CERVICAL
GANGLION, DORSAL ROOT, CERVICAL: INFILTRATE, MONONUCLEAR CELLS, MINIMAL
GANGLION, DORSAL ROOT, CERVICAL, GLIAL CELL: HYPERTROPHY/HYPERPLASIA, MINIMAL
GANGLION, DORSAL ROOT, LUMBAR
GANGLION, DORSAL ROOT, LUMBAR: INFILTRATE, MONONUCLEAR CELLS, MINIMAL, MULTIFOCAL
GANGLION, DORSAL ROOT, LUMBAR, GLIAL CELL: HYPERTROPHY/HYPERPLASIA, MINIMAL
GANGLION, DORSAL ROOT, THORACIC
GANGLION, DORSAL ROOT, THORACIC, GLIAL CELL: HYPERTROPHY/HYPERPLASIA, MINIMAL
HEART
HEART, MYOCARDIUM: DEGENERATION/NECROSIS, MINIMAL, MULTIFOCAL
HEART, MYOCARDIUM: INFILTRATE, MONONUCLEAR CELLS, MINIMAL, MULTIFOCAL
LIVER
LIVER: INFILTRATE, MIXED, MINIMAL
NERVE ROOT, SPINAL, LUMBAR
NERVE ROOT, SPINAL, LUMBAR, NERVE ROOT, DORSAL: DEGENERATION, NERVE FIBER, MINIMAL
NERVE, SCIATIC
NERVE, SCIATIC: DEGENERATION, NERVE FIBER, MINIMAL
NERVE, TIBIAL
NERVE, TIBIAL: DEGENERATION, NERVE FIBER, MINIMAL
SPINAL CORD, THORACIC
SPINAL CORD, THORACIC, WHITE MATTER: DEGENERATION, NERVE FIBER, MINIMAL, LATERAL

Appendix 10

Individual Animal Report

Study ID: 02776-0018: CRL 2954-001 / U of Tex Southwestern Med UTSW.Gray-002
Study Title: A SINGLE DOSE TOXICITY STUDY OF AAV9/SURF1 ADMINISTERED BY INTRATHECAL INJECTION IN RATS

Animal: 4510 (Female)

Sacrifice: DAY 91
Group: 4FD91 / AAV9/SURF1 HIGH DOSE (2.49E12 VG)
Fate: SCHEDULED SACRIFICE / Time on Test: 91 / Cause(s) of Death:

Gross or Microscopic Finding
Disposition
Tissue: Site / Finding, Severity

MICROSCOPIC EXAMINATION

Disposition: NORMAL

BRAIN, AMYGDALOID BODY
BRAIN, BASAL NUCLEI/STRIATUM
BRAIN, CEREBELLUM
BRAIN, CEREBRAL CORTEX
BRAIN, HIPPOCAMPUS
BRAIN, HYPOTHALAMUS
BRAIN, MEDULLA OBLONGATA
BRAIN, MENINGES
BRAIN, MIDBRAIN
BRAIN, OLFACTORY BULB
BRAIN, PONS
BRAIN, THALAMUS
BRAIN, VENTRICULAR SYSTEM
BRAIN, WHITE MATTER
EYE
GANGLION, DORSAL ROOT, THORACIC
KIDNEY
LYMPH NODE, ILIAC
LYMPH NODE, MANDIBULAR
LYMPH NODE, MESENTERIC
MUSCLE, BICEPS FEMORIS
MUSCLE, GASTROCNEMIUS
NERVE ROOT, SPINAL, CERVICAL
NERVE ROOT, SPINAL, LUMBAR
NERVE ROOT, SPINAL, THORACIC
NERVE, OPTIC
OVARY
PANCREAS
SPLEEN
THYMUS

Disposition: ABNORMAL/FINDING

GANGLION, DORSAL ROOT, CERVICAL
GANGLION, DORSAL ROOT, CERVICAL, GLIAL CELL: HYPERTROPHY/HYPERPLASIA, MINIMAL

Appendix 10

Individual Animal Report

Study ID: 02776-0018: CRL 2954-001 / U of Tex Southwestern Med UTSW.Gray-002
Study Title: A SINGLE DOSE TOXICITY STUDY OF AAV9/SURF1 ADMINISTERED BY INTRATHECAL INJECTION IN RATS

Animal: 4510 (Female)

Sacrifice: DAY 91
Group: 4FD91 / AAV9/SURF1 HIGH DOSE (2.49E12 VG)
Fate: SCHEDULED SACRIFICE / Time on Test: 91 / Cause(s) of Death:

Gross or Microscopic Finding
Disposition
Tissue: Site / Finding, Severity

GANGLION, DORSAL ROOT, LUMBAR
GANGLION, DORSAL ROOT, LUMBAR: INFILTRATE, MONONUCLEAR CELLS, MINIMAL, MULTIFOCAL
HEART
HEART, MYOCARDIUM: DEGENERATION/NECROSIS, MINIMAL, MULTIFOCAL
HEART, MYOCARDIUM: FIBROSIS, MINIMAL
HEART, MYOCARDIUM: INFILTRATE, MONONUCLEAR CELLS, MINIMAL, MULTIFOCAL
LIVER
LIVER: INFILTRATE, MIXED, MINIMAL
LIVER: NECROSIS, MINIMAL, MULTIFOCAL
LUNG
LUNG, ALVEOLUS: INFILTRATE, MACROPHAGES, MINIMAL, FOCAL
NERVE, SCIATIC
NERVE, SCIATIC: DEGENERATION, NERVE FIBER, MINIMAL
NERVE, TIBIAL
NERVE, TIBIAL: DEGENERATION, NERVE FIBER, MINIMAL
SPINAL CORD, CERVICAL
SPINAL CORD, CERVICAL, WHITE MATTER: DEGENERATION, NERVE FIBER, MINIMAL
COMMENT(S): SEEN IN LONG SECTION AND EXACT LOCATION COULD NOT BE DETERMINED
SPINAL CORD, LUMBAR
SPINAL CORD, LUMBAR, NERVE ROOT, SPINAL: DEGENERATION, NERVE FIBER, MINIMAL
SPINAL CORD, THORACIC
SPINAL CORD, THORACIC, WHITE MATTER: DEGENERATION, NERVE FIBER, MINIMAL, DORSAL

Appendix 10

Individual Animal Report

Study ID: 02776-0018: CRL 2954-001 / U of Tex Southwestern Med UTSW.Gray-002
Study Title: A SINGLE DOSE TOXICITY STUDY OF AAV9/SURF1 ADMINISTERED BY INTRATHECAL INJECTION IN RATS

Animal: 3008 (Male)

Sacrifice: US
Group: ED / EARLY DEATH (0.83E12 VG)
Fate: UNSCHEDULED SACRIFICE / Time on Test: 16 / Cause(s) of Death:

Gross or Microscopic Finding
Disposition
Tissue: Site / Finding, Severity

MICROSCOPIC EXAMINATION

Disposition: NORMAL

BRAIN, AMYGDALOID BODY
BRAIN, BASAL NUCLEI/STRIATUM
BRAIN, CEREBELLUM
BRAIN, CEREBRAL CORTEX
BRAIN, HIPPOCAMPUS
BRAIN, HYPOTHALAMUS
BRAIN, MEDULLA OBLONGATA
BRAIN, MENINGES
BRAIN, MIDBRAIN
BRAIN, OLFACTORY BULB
BRAIN, PONS
BRAIN, THALAMUS
BRAIN, WHITE MATTER
EYE
GANGLION, DORSAL ROOT, CERVICAL
LUNG
LYMPH NODE, ILIAC
LYMPH NODE, MANDIBULAR
LYMPH NODE, MESENTERIC
MUSCLE, BICEPS FEMORIS
MUSCLE, GASTROCNEMIUS
NERVE ROOT, SPINAL, CERVICAL
NERVE, OPTIC
PANCREAS
SPLEEN
TESTIS
THYMUS

Disposition: ABNORMAL/FINDING

BRAIN, VENTRICULAR SYSTEM
BRAIN, VENTRICULAR SYSTEM: INFILTRATE, MONONUCLEAR CELLS, MINIMAL, FOCAL
COMMENT(S): PERIVENTRICULAR ADJACENT TO SUBFORNICAL ORGAN
GANGLION, DORSAL ROOT, LUMBAR
GANGLION, DORSAL ROOT, LUMBAR, GLIAL CELL: HYPERTROPHY/HYPERPLASIA, MINIMAL
GANGLION, DORSAL ROOT, THORACIC

Appendix 10

Individual Animal Report

Study ID: 02776-0018: CRL 2954-001 / U of Tex Southwestern Med UTSW.Gray-002
Study Title: A SINGLE DOSE TOXICITY STUDY OF AAV9/SURF1 ADMINISTERED BY INTRATHECAL INJECTION IN RATS

Animal: 3008 (Male)

Sacrifice: US
Group: ED / EARLY DEATH (0.83E12 VG)
Fate: UNSCHEDULED SACRIFICE / Time on Test: 16 / Cause(s) of Death:

Gross or Microscopic Finding
Disposition
Tissue: Site / Finding, Severity

GANGLION, DORSAL ROOT, THORACIC: INFILTRATE, MONONUCLEAR CELLS, MINIMAL, FOCAL
GANGLION, DORSAL ROOT, THORACIC, GLIAL CELL: HYPERTROPHY/HYPERPLASIA, MINIMAL
HEART
HEART, MYOCARDIUM: DEGENERATION/NECROSIS, MINIMAL, FOCAL
HEART, MYOCARDIUM: INFILTRATE, MONONUCLEAR CELLS, MINIMAL, MULTIFOCAL
KIDNEY
KIDNEY: CHRONIC PROGRESSIVE NEPHROPATHY, MINIMAL
LIVER
LIVER: INFILTRATE, MIXED, MINIMAL
NERVE ROOT, SPINAL, LUMBAR
NERVE ROOT, SPINAL, LUMBAR, NERVE ROOT, VENTRAL: DEGENERATION, NERVE FIBER, SEVERE
NERVE ROOT, SPINAL, LUMBAR, NERVE ROOT, VENTRAL: INFILTRATE, MONONUCLEAR CELLS, MINIMAL
NERVE ROOT, SPINAL, LUMBAR, SCHWANN CELL: HYPERTROPHY/HYPERPLASIA, MODERATE
NERVE ROOT, SPINAL, THORACIC
NERVE ROOT, SPINAL, THORACIC, EPINEURIUM: INFILTRATE, MONONUCLEAR CELLS, MINIMAL, FOCAL
NERVE ROOT, SPINAL, THORACIC, NERVE ROOT, VENTRAL: DEGENERATION, NERVE FIBER, MILD
NERVE ROOT, SPINAL, THORACIC, SCHWANN CELL: HYPERTROPHY/HYPERPLASIA, MINIMAL
NERVE, SCIATIC
NERVE, SCIATIC: DEGENERATION, NERVE FIBER, MILD
NERVE, SCIATIC, SCHWANN CELL: HYPERTROPHY/HYPERPLASIA, MINIMAL
NERVE, TIBIAL
NERVE, TIBIAL: DEGENERATION, NERVE FIBER, MILD
SPINAL CORD, CERVICAL
SPINAL CORD, CERVICAL, GLIAL CELL: INCREASED CELLULARITY, MINIMAL
COMMENT(S): FOCAL REGIONS IN BOTH WHITE MATTER AND GRAY MATTER
SPINAL CORD, CERVICAL, GRAY MATTER: INFILTRATE, MONONUCLEAR CELLS, MINIMAL, PERIVASCULAR
SPINAL CORD, LUMBAR
SPINAL CORD, LUMBAR, GLIAL CELL: INCREASED CELLULARITY, MODERATE
COMMENT(S): PREDOMINANTLY WITHIN GRAY MATTER
SPINAL CORD, LUMBAR, GRAY MATTER: DEGENERATION/NECROSIS, MINIMAL
COMMENT(S): AFFECTING CELLS PREDOMINANTLY WITHIN LATERAL OR VENTRAL HORNS BILATERALLY
SPINAL CORD, LUMBAR, NERVE ROOT, SPINAL: DEGENERATION, NERVE FIBER, MINIMAL
SPINAL CORD, LUMBAR, WHITE MATTER: DEGENERATION, NERVE FIBER, MINIMAL
SPINAL CORD, THORACIC
SPINAL CORD, THORACIC, GLIAL CELL: INCREASED CELLULARITY, MARKED
COMMENT(S): WITHIN BOTH GRAY AND WHITE MATTER BUT PREDOMINANTLY WITHIN GRAY MATTER

Appendix 10

Individual Animal Report

Study ID: 02776-0018: CRL 2954-001 / U of Tex Southwestern Med UTSW.Gray-002
Study Title: A SINGLE DOSE TOXICITY STUDY OF AAV9/SURF1 ADMINISTERED BY INTRATHECAL INJECTION IN RATS

Animal: 3008 (Male)

Sacrifice: US
Group: ED / EARLY DEATH (0.83E12 VG)
Fate: UNSCHEDULED SACRIFICE / Time on Test: 16 / Cause(s) of Death:

Gross or Microscopic Finding
Disposition
Tissue: Site / Finding, Severity

SPINAL CORD, THORACIC, GRAY MATTER: DEGENERATION/NECROSIS, SEVERE
COMMENT(S): AFFECTING CELLS PREDOMINANTLY WITHIN LATERAL OR VENTRAL HORNS
SPINAL CORD, THORACIC, GRAY MATTER: INFILTRATE, MONONUCLEAR CELLS, MILD, MULTIFOCAL, PERIVASCULAR
SPINAL CORD, THORACIC, WHITE MATTER: DEGENERATION, NERVE FIBER, MARKED

Appendix 10

DATA SECTION II: MICROSCOPIC DATA REPORT

The Microscopic Data Report lists all individual microscopic findings, organized by dose group, in a cross-tab format.

- In this report, the microscopic observations are organized by Tissue/Site (optional)/Diagnosis.
- Regardless of modifiers, all findings in the same tissue and site with the same diagnosis are merged together in this report.
    - Example: *Inflammation* (diagnosis), *bilateral* (modifier) and *Inflammation*, *unilateral* would be merged into the single finding *Inflammation* in this report.
- A grade for each diagnosis is provided. Grades are defined in the Diagnosis Explanation Report (Data Section VII) and/or Appendix 3.

Appendix 10

Microscopic Data Report

Study ID: 02776-0018: CRL 2954-001 / U of Tex Southwestern Med UTSW.Gray-002
Study Title: A SINGLE DOSE TOXICITY STUDY OF AAV9/SURF1 ADMINISTERED BY INTRATHECAL INJECTION IN RATS

DAY 08 SACRIFICE<br>GROUP 1MD08<br>VEHICLE (0 VG)	ANIMAL	1011	1012	1013	1014	1015
**Tissue**	**Sex**	M	M	M	M	M
Site	**Fate**	SS	SS	SS	SS	SS
Diagnosis	**Time on Test**	08	08	08	08	08
**NERVE ROOT, SPINAL, CERVICAL**		N	N	N	N	N
**GANGLION, DORSAL ROOT, CERVICAL**		N	N	N	A	N
GLIAL CELL						
HYPERTROPHY/HYPERPLASIA		-	-	-	1	-
**NERVE ROOT, SPINAL, THORACIC**		N	N	A	N	N
NERVE ROOT, VENTRAL						
DEGENERATION, NERVE FIBER		-	-	1	-	-
**GANGLION, DORSAL ROOT, THORACIC**		N	N	N	A	N
GLIAL CELL						
HYPERTROPHY/HYPERPLASIA		-	-	-	1	-
**NERVE ROOT, SPINAL, LUMBAR**		N	N	A	N	N
NERVE ROOT, VENTRAL						
DEGENERATION, NERVE FIBER		-	-	1	-	-
**GANGLION, DORSAL ROOT, LUMBAR**		N	N	N	A	N
GLIAL CELL						
HYPERTROPHY/HYPERPLASIA		-	-	-	1	-
**BRAIN, MENINGES**		N	N	N	N	N
**BRAIN, CEREBRAL CORTEX**		N	N	N	N	N
**BRAIN, OLFACTORY BULB**		N	N	N	N	N
**BRAIN, WHITE MATTER**		N	N	N	N	N
**BRAIN, VENTRICULAR SYSTEM**		N	N	N	N	N
**BRAIN, BASAL NUCLEI/STRIATUM**		N	N	N	N	N
**BRAIN, AMYGDALOID BODY**		N	N	N	N	N
**BRAIN, HIPPOCAMPUS**		N	N	N	N	N
**BRAIN, HYPOTHALAMUS**		N	N	N	N	N
**BRAIN, THALAMUS**		N	N	N	N	N
**BRAIN, MIDBRAIN**		N	N	N	N	N
**BRAIN, CEREBELLUM**		N	N	N	N	N
**BRAIN, PONS**		N	N	N	N	N
**BRAIN, MEDULLA OBLONGATA**		N	N	N	N	N
**SPINAL CORD, CERVICAL**		N	N	N	N	N
**SPINAL CORD, THORACIC**		N	N	N	N	N
**SPINAL CORD, LUMBAR**		A	N	N	N	N
NERVE ROOT, SPINAL						
DEGENERATION, NERVE FIBER		1	-	-	-	-
**NERVE, SCIATIC**		A	N	A	N	A
DEGENERATION, NERVE FIBER		2	-	1	-	1
**NERVE, TIBIAL**		N	N	N	N	N
**EYE**		N	N	N	N	N
**NERVE, OPTIC**		N	N	N	N	N
**LYMPH NODE, MANDIBULAR**		N	N	N	N	N
**THYMUS**		N	N	N	N	N
**PANCREAS**		N	N	N	N	N
**LUNG**		N	N	N	N	N

Appendix 10

Microscopic Data Report

Study ID: 02776-0018: CRL 2954-001 / U of Tex Southwestern Med UTSW.Gray-002
Study Title: A SINGLE DOSE TOXICITY STUDY OF AAV9/SURF1 ADMINISTERED BY INTRATHECAL INJECTION IN RATS

DAY 08 SACRIFICE GROUP 1MD08 VEHICLE (0 VG)	ANIMAL	1011	1012	1013	1014	1015
**Tissue**	Sex	M	M	M	M	M
Site	Fate	SS	SS	SS	SS	SS
Diagnosis	Time on Test	08	08	08	08	08
**MUSCLE, BICEPS FEMORIS**		N	N	N	N	N
**MUSCLE, GASTROCNEMIUS**		N	N	N	N	N
**HEART**		N	N	N	N	N
**SPLEEN**		N	N	N	N	N
**LIVER**		A	A	A	A	N
EXTRAMEDULLARY HEMATOPOIESIS		1	-	1	1	-
INFILTRATE, MIXED		-	1	-	1	-
**KIDNEY**		A	N	A	A	A
CHRONIC PROGRESSIVE NEPHROPATHY		1	-	-	1	1
TUBULAR						
BASOPHILIA		-	-	1	-	-
CYST		-	-	1	-	-
**LYMPH NODE, MESENTERIC**		N	N	N	N	N
**TESTIS**		N	N	N	N	N
**LYMPH NODE, ILIAC**		N	N	N	N	N

Appendix 10

Microscopic Data Report

Study ID: 02776-0018: CRL 2954-001 / U of Tex Southwestern Med UTSW.Gray-002
Study Title: A SINGLE DOSE TOXICITY STUDY OF AAV9/SURF1 ADMINISTERED BY INTRATHECAL INJECTION IN RATS

DAY 08 SACRIFICE<br>GROUP 2MD08<br>AAV9/SURF1 LOW DOSE (0.28E12 VG)	ANIMAL	2011	2012	2013	2014	2015
**Tissue**	Sex	M	M	M	M	M
Site	Fate	SS	SS	SS	SS	SS
Diagnosis	Time on Test	08	08	08	08	08
**NERVE ROOT, SPINAL, CERVICAL**		N	N	N	N	N
**GANGLION, DORSAL ROOT, CERVICAL**		A	A	A	N	N
GLIAL CELL						
HYPERTROPHY/HYPERPLASIA		1	1	1	-	-
**NERVE ROOT, SPINAL, THORACIC**		A	N	N	N	N
NERVE ROOT, VENTRAL						
DEGENERATION, NERVE FIBER		1	-	-	-	-
**GANGLION, DORSAL ROOT, THORACIC**		A	N	N	N	N
GLIAL CELL						
HYPERTROPHY/HYPERPLASIA		1	-	-	-	-
**NERVE ROOT, SPINAL, LUMBAR**		N	N	N	A	N
EPINEURIUM						
INFILTRATE, MONONUCLEAR CELLS		-	-	-	1	-
**GANGLION, DORSAL ROOT, LUMBAR**		A	A	A	A	A
INFILTRATE, MONONUCLEAR CELLS		-	-	-	1	-
GLIAL CELL						
HYPERTROPHY/HYPERPLASIA		1	1	1	-	1
**BRAIN, MENINGES**		N	N	N	N	N
**BRAIN, CEREBRAL CORTEX**		N	N	N	N	N
**BRAIN, OLFACTORY BULB**		N	N	N	N	N
**BRAIN, WHITE MATTER**		N	N	N	N	N
**BRAIN, VENTRICULAR SYSTEM**		N	N	N	N	N
**BRAIN, BASAL NUCLEI/STRIATUM**		N	N	N	N	N
**BRAIN, AMYGDALOID BODY**		N	N	N	N	N
**BRAIN, HIPPOCAMPUS**		N	N	N	N	N
**BRAIN, HYPOTHALAMUS**		N	N	N	N	N
**BRAIN, THALAMUS**		N	N	N	N	N
**BRAIN, MIDBRAIN**		N	N	N	N	N
**BRAIN, CEREBELLUM**		N	N	N	N	N
**BRAIN, PONS**		N	N	N	N	N
**BRAIN, MEDULLA OBLONGATA**		N	N	N	N	N
**SPINAL CORD, CERVICAL**		N	N	N	N	N
**SPINAL CORD, THORACIC**		N	N	N	N	N
**SPINAL CORD, LUMBAR**		N	N	N	N	A
WHITE MATTER						
DEGENERATION, NERVE FIBER		-	-	-	-	1
**NERVE, SCIATIC**		N	A	A	A	N
DEGENERATION, NERVE FIBER		-	1	1	1	-
**NERVE, TIBIAL**		N	N	A	N	N
DEGENERATION, NERVE FIBER		-	-	1	-	-
**EYE**		N	N	N	N	N
**NERVE, OPTIC**		N	N	N	N	N
**LYMPH NODE, MANDIBULAR**		N	N	N	N	N
**THYMUS**		N	N	N	N	N

Appendix 10

Microscopic Data Report

Study ID: 02776-0018: CRL 2954-001 / U of Tex Southwestern Med UTSW.Gray-002
Study Title: A SINGLE DOSE TOXICITY STUDY OF AAV9/SURF1 ADMINISTERED BY INTRATHECAL INJECTION IN RATS

DAY 08 SACRIFICE<br>GROUP 2MD08<br>AAV9/SURF1 LOW DOSE (0.28E12 VG)	ANIMAL	2011	2012	2013	2014	2015
**Tissue**	Sex	M	M	M	M	M
Site	Fate	SS	SS	SS	SS	SS
Diagnosis	Time on Test	08	08	08	08	08
**PANCREAS**		N	N	N	N	N
**LUNG**		N	N	N	N	N
**MUSCLE, BICEPS FEMORIS**		N	N	N	N	N
**MUSCLE, GASTROCNEMIUS**		N	N	N	N	N
**HEART**		A	A	A	A	A
MYOCARDIUM						
DEGENERATION/NECROSIS		1	1	1	1	1
INFILTRATE, MONONUCLEAR CELLS		1	1	1	1	1
**SPLEEN**		N	N	N	N	N
**LIVER**		A	A	A	A	A
EXTRAMEDULLARY HEMATOPOIESIS		1	1	1	1	1
HYPERTROPHY/HYPERPLASIA		1	-	-	-	-
INFILTRATE, MIXED		1	1	1	1	1
**KIDNEY**		N	N	N	A	A
CHRONIC PROGRESSIVE NEPHROPATHY		-	-	-	1	-
TUBULAR						
CYST		-	-	-	-	1
**LYMPH NODE, MESENTERIC**		N	N	N	N	N
**TESTIS**		N	N	N	N	N
**LYMPH NODE, ILIAC**		N	N	N	N	N

Appendix 10

Microscopic Data Report

Study ID: 02776-0018: CRL 2954-001 / U of Tex Southwestern Med UTSW.Gray-002
Study Title: A SINGLE DOSE TOXICITY STUDY OF AAV9/SURF1 ADMINISTERED BY INTRATHECAL INJECTION IN RATS

DAY 08 SACRIFICE<br>GROUP 3MD08<br>AAV9/SURF1 MID DOSE (0.83E12 VG)	ANIMAL	3011	3012	3013	3014	3015	4013
**Tissue**	Sex	M	M	M	M	M	M
Site	Fate	SS	SS	SS	SS	SS	SS
Diagnosis	Time on Test	08	08	08	08	08	08
**NERVE ROOT, SPINAL, CERVICAL**		N	N	N	N	N	N
**GANGLION, DORSAL ROOT, CERVICAL**		N	A	N	N	N	N
INFILTRATE, MONONUCLEAR CELLS		-	1	-	-	-	-
**NERVE ROOT, SPINAL, THORACIC**		N	N	N	N	N	N
**GANGLION, DORSAL ROOT, THORACIC**		N	N	N	N	N	N
**NERVE ROOT, SPINAL, LUMBAR**		A	N	A	N	N	A
EPINEURIUM							
INFILTRATE, MONONUCLEAR CELLS		1	-	1	-	-	1
**GANGLION, DORSAL ROOT, LUMBAR**		A	N	A	A	N	A
INFILTRATE, MONONUCLEAR CELLS		1	-	-	-	-	1
GLIAL CELL							
HYPERTROPHY/HYPERPLASIA		1	-	1	1	-	-
**BRAIN, MENINGES**		N	N	N	N	N	N
**BRAIN, CEREBRAL CORTEX**		N	N	N	N	N	N
**BRAIN, OLFACTORY BULB**		N	N	U	N	N	N
**BRAIN, WHITE MATTER**		N	N	N	N	N	N
**BRAIN, VENTRICULAR SYSTEM**		N	N	N	N	N	N
**BRAIN, BASAL NUCLEI/STRIATUM**		N	N	N	N	N	N
**BRAIN, AMYGDALOID BODY**		N	N	N	N	N	N
**BRAIN, HIPPOCAMPUS**		N	N	N	N	N	N
**BRAIN, HYPOTHALAMUS**		N	N	N	N	N	N
**BRAIN, THALAMUS**		N	N	N	N	N	N
**BRAIN, MIDBRAIN**		N	N	N	N	N	N
**BRAIN, CEREBELLUM**		N	N	N	N	N	N
**BRAIN, PONS**		N	N	N	N	N	N
**BRAIN, MEDULLA OBLONGATA**		N	N	N	N	N	N
**SPINAL CORD, CERVICAL**		N	N	N	N	N	N
**SPINAL CORD, THORACIC**		N	N	N	A	N	N
WHITE MATTER							
DEGENERATION, NERVE FIBER		-	-	-	1	-	-
**SPINAL CORD, LUMBAR**		N	N	N	N	N	A
NERVE ROOT, SPINAL							
DEGENERATION, NERVE FIBER		-	-	-	-	-	1
**NERVE, SCIATIC**		A	A	A	A	N	A
DEGENERATION, NERVE FIBER		1	1	1	1	-	1
**NERVE, TIBIAL**		N	N	N	U	N	N
**EYE**		N	N	N	N	N	N
**NERVE, OPTIC**		N	N	N	N	N	N
**LYMPH NODE, MANDIBULAR**		N	N	N	N	N	N
**THYMUS**		N	N	N	N	N	N
**PANCREAS**		N	N	N	N	N	N
**LUNG**		N	N	N	N	A	N
INFLAMMATION		-	-	-	-	1	-
**MUSCLE, BICEPS FEMORIS**		N	N	N	N	N	N

Appendix 10

Microscopic Data Report

Study ID: 02776-0018: CRL 2954-001 / U of Tex Southwestern Med UTSW.Gray-002
Study Title: A SINGLE DOSE TOXICITY STUDY OF AAV9/SURF1 ADMINISTERED BY INTRATHECAL INJECTION IN RATS

DAY 08 SACRIFICE<br>GROUP 3MD08<br>AAV9/SURF1 MID DOSE (0.83E12 VG)	ANIMAL	3011	3012	3013	3014	3015	4013
**Tissue**	Sex	M	M	M	M	M	M
Site	Fate	SS	SS	SS	SS	SS	SS
Diagnosis	Time on Test	08	08	08	08	08	08
**MUSCLE, GASTROCNEMIUS**		N	N	N	N	N	N
**HEART**		N	A	A	A	A	N
MYOCARDIUM							
DEGENERATION/NECROSIS		-	1	-	1	1	-
INFILTRATE, MONONUCLEAR CELLS		-	1	1	1	1	-
**SPLEEN**		N	N	N	N	N	A
EXTRAMEDULLARY HEMATOPOIESIS		-	-	-	-	-	1
**LIVER**		A	A	A	A	A	A
EXTRAMEDULLARY HEMATOPOIESIS		-	1	1	-	-	1
HYPERTROPHY/HYPERPLASIA		-	-	1	-	-	-
INFILTRATE, MIXED		1	1	1	1	1	1
HEPATOCYTE							
NECROSIS, SINGLE CELL		-	-	1	-	-	-
**KIDNEY**		A	A	N	A	A	A
CHRONIC PROGRESSIVE NEPHROPATHY		1	-	-	-	-	1
INFILTRATE, MONONUCLEAR CELLS		-	-	-	-	1	-
TUBULAR							
BASOPHILIA		-	1	-	1	-	-
CYST		-	1	-	-	-	-
**LYMPH NODE, MESENTERIC**		N	N	N	N	N	N
**TESTIS**		N	N	N	N	N	N
**LYMPH NODE, ILIAC**		N	N	N	N	N	N

Appendix 10

Microscopic Data Report

Study ID: 02776-0018: CRL 2954-001 / U of Tex Southwestern Med UTSW.Gray-002
Study Title: A SINGLE DOSE TOXICITY STUDY OF AAV9/SURF1 ADMINISTERED BY INTRATHECAL INJECTION IN RATS

DAY 08 SACRIFICE<br>GROUP 4MD08<br>AAV9/SURF1 HIGH DOSE (2.49E12 VG)	ANIMAL	4011	4012	4021	4022	4113
**Tissue**	Sex	M	M	M	M	M
Site	Fate	SS	SS	SS	SS	SS
Diagnosis	Time on Test	08	08	08	08	08
**NERVE ROOT, SPINAL, CERVICAL**		N	A	N	N	N
NERVE ROOT, VENTRAL						
DEGENERATION, NERVE FIBER		-	1	-	-	-
**GANGLION, DORSAL ROOT, CERVICAL**		N	N	N	N	N
**NERVE ROOT, SPINAL, THORACIC**		N	N	N	N	N
**GANGLION, DORSAL ROOT, THORACIC**		N	N	N	N	N
**NERVE ROOT, SPINAL, LUMBAR**		N	N	N	N	N
**GANGLION, DORSAL ROOT, LUMBAR**		N	N	N	N	N
**BRAIN, MENINGES**		N	N	N	N	N
**BRAIN, CEREBRAL CORTEX**		N	N	N	N	N
**BRAIN, OLFACTORY BULB**		N	N	N	N	N
**BRAIN, WHITE MATTER**		N	N	N	N	N
**BRAIN, VENTRICULAR SYSTEM**		N	N	N	N	N
**BRAIN, BASAL NUCLEI/STRIATUM**		N	N	N	N	N
**BRAIN, AMYGDALOID BODY**		N	N	N	N	N
**BRAIN, HIPPOCAMPUS**		N	N	N	N	N
**BRAIN, HYPOTHALAMUS**		N	N	N	N	N
**BRAIN, THALAMUS**		N	N	N	N	N
**BRAIN, MIDBRAIN**		N	N	N	N	N
**BRAIN, CEREBELLUM**		N	N	N	N	N
**BRAIN, PONS**		N	N	N	N	N
**BRAIN, MEDULLA OBLONGATA**		N	N	N	N	N
**SPINAL CORD, CERVICAL**		N	N	N	N	N
**SPINAL CORD, THORACIC**		A	N	N	N	N
MENINGES						
INFILTRATE, MONONUCLEAR CELLS		1	-	-	-	-
**SPINAL CORD, LUMBAR**		N	N	N	N	A
NERVE ROOT, SPINAL						
DEGENERATION, NERVE FIBER		-	-	-	-	1
**NERVE, SCIATIC**		N	N	N	A	N
DEGENERATION, NERVE FIBER		-	-	-	1	-
**NERVE, TIBIAL**		N	N	N	N	N
**EYE**		N	N	N	N	N
**NERVE, OPTIC**		N	N	N	N	N
**LYMPH NODE, MANDIBULAR**		N	N	N	N	N
**THYMUS**		N	U	N	N	N
**PANCREAS**		A	N	N	N	N
APOPTOSIS/SINGLE CELL NECROSIS		1	-	-	-	-
MITOTIC FIGURES, INCREASED		1	-	-	-	-
**LUNG**		N	N	N	N	N
**MUSCLE, BICEPS FEMORIS**		A	N	N	N	N
DEGENERATION/NECROSIS		1	-	-	-	-
**MUSCLE, GASTROCNEMIUS**		N	N	N	N	N
**HEART**		N	N	N	N	A

Appendix 10

Microscopic Data Report

Study ID: 02776-0018: CRL 2954-001 / U of Tex Southwestern Med UTSW.Gray-002
Study Title: A SINGLE DOSE TOXICITY STUDY OF AAV9/SURF1 ADMINISTERED BY INTRATHECAL INJECTION IN RATS

DAY 08 SACRIFICE<br>GROUP 4MD08<br>AAV9/SURF1 HIGH DOSE (2.49E12 VG)	ANIMAL	4011	4012	4021	4022	4113
**Tissue**	**Sex**	M	M	M	M	M
Site	**Fate**	SS	SS	SS	SS	SS
Diagnosis	**Time on Test**	08	08	08	08	08
MYOCARDIUM						
INFILTRATE, MONONUCLEAR CELLS		-	-	-	-	1
**SPLEEN**		N	N	N	N	N
**LIVER**		A	A	A	A	A
EXTRAMEDULLARY HEMATOPOIESIS		1	1	-	-	1
HYPERTROPHY/HYPERPLASIA		1	-	-	-	-
INFILTRATE, MIXED		1	1	1	1	1
MITOTIC FIGURES, INCREASED		1	-	-	-	-
HEPATOCYTE						
NECROSIS, SINGLE CELL		2	1	-	-	-
**KIDNEY**		A	A	A	A	N
CHRONIC PROGRESSIVE NEPHROPATHY		-	1	-	1	-
TUBULAR						
BASOPHILIA		1	-	1	-	-
**LYMPH NODE, MESENTERIC**		N	N	N	N	N
**TESTIS**		N	N	N	N	N
**LYMPH NODE, ILIAC**		N	N	N	N	N

Appendix 10

Microscopic Data Report

Study ID: 02776-0018: CRL 2954-001 / U of Tex Southwestern Med UTSW.Gray-002
Study Title: A SINGLE DOSE TOXICITY STUDY OF AAV9/SURF1 ADMINISTERED BY INTRATHECAL INJECTION IN RATS

DAY 08 SACRIFICE<br>GROUP 1FD08<br>VEHICLE (0 VG)	ANIMAL	1511	1512	1513	1514	1515
**Tissue**	**Sex**	F	F	F	F	F
Site	**Fate**	SS	SS	SS	SS	SS
Diagnosis	**Time on Test**	08	08	08	08	08
**NERVE ROOT, SPINAL, CERVICAL**		N	N	N	N	N
**GANGLION, DORSAL ROOT, CERVICAL**		N	N	N	N	A
GLIAL CELL						
HYPERTROPHY/HYPERPLASIA		-	-	-	-	1
**NERVE ROOT, SPINAL, THORACIC**		N	N	N	N	N
**GANGLION, DORSAL ROOT, THORACIC**		N	N	N	N	N
**NERVE ROOT, SPINAL, LUMBAR**		N	N	N	N	N
**GANGLION, DORSAL ROOT, LUMBAR**		N	A	N	N	N
GLIAL CELL						
HYPERTROPHY/HYPERPLASIA		-	1	-	-	-
**BRAIN, MENINGES**		N	N	N	N	N
**BRAIN, CEREBRAL CORTEX**		N	N	N	N	N
**BRAIN, OLFACTORY BULB**		N	N	N	N	N
**BRAIN, WHITE MATTER**		N	N	N	N	N
**BRAIN, VENTRICULAR SYSTEM**		N	N	N	N	N
**BRAIN, BASAL NUCLEI/STRIATUM**		N	N	N	N	N
**BRAIN, AMYGDALOID BODY**		N	N	N	N	N
**BRAIN, HIPPOCAMPUS**		N	N	N	N	N
**BRAIN, HYPOTHALAMUS**		N	N	N	N	N
**BRAIN, THALAMUS**		N	N	N	N	N
**BRAIN, MIDBRAIN**		N	N	N	N	N
**BRAIN, CEREBELLUM**		N	N	N	N	N
**BRAIN, PONS**		N	N	N	N	N
**BRAIN, MEDULLA OBLONGATA**		N	N	N	N	N
**SPINAL CORD, CERVICAL**		N	N	N	N	N
**SPINAL CORD, THORACIC**		N	N	N	N	N
**SPINAL CORD, LUMBAR**		N	N	N	N	A
WHITE MATTER						
DEGENERATION, NERVE FIBER		-	-	-	-	1
**NERVE, SCIATIC**		N	N	N	A	A
DEGENERATION, NERVE FIBER		-	-	-	1	1
**NERVE, TIBIAL**		N	N	N	N	A
DEGENERATION, NERVE FIBER		-	-	-	-	1
**EYE**		A	N	N	N	A
RETINAL ROSETTE		1	-	-	-	1
**NERVE, OPTIC**		N	N	N	N	N
**LYMPH NODE, MANDIBULAR**		N	N	N	N	N
**THYMUS**		N	N	N	N	N
**PANCREAS**		N	N	N	N	N
**LUNG**		N	N	N	A	N
ALVEOLUS						
INFILTRATE, MACROPHAGES		-	-	-	1	-
**MUSCLE, BICEPS FEMORIS**		N	N	N	N	N
**MUSCLE, GASTROCNEMIUS**		N	N	N	N	N

Appendix 10

Microscopic Data Report

Study ID: 02776-0018: CRL 2954-001 / U of Tex Southwestern Med UTSW.Gray-002
Study Title: A SINGLE DOSE TOXICITY STUDY OF AAV9/SURF1 ADMINISTERED BY INTRATHECAL INJECTION IN RATS

DAY 08 SACRIFICE<br>GROUP 1FD08<br>VEHICLE (0 VG)	ANIMAL	1511	1512	1513	1514	1515
**Tissue**	Sex	F	F	F	F	F
Site	Fate	SS	SS	SS	SS	SS
Diagnosis	Time on Test	08	08	08	08	08
**HEART**		A	A	N	N	N
MYOCARDIUM						
DEGENERATION/NECROSIS		1	1	-	-	-
INFILTRATE, MONONUCLEAR CELLS		1	1	-	-	-
**SPLEEN**		N	N	N	N	N
**LIVER**		A	A	A	N	N
EXTRAMEDULLARY HEMATOPOIESIS		1	1	1	-	-
INFILTRATE, MIXED		1	-	1	-	-
**KIDNEY**		N	A	A	N	N
CHRONIC PROGRESSIVE NEPHROPATHY		-	1	-	-	-
TUBULAR						
BASOPHILIA		-	-	1	-	-
**LYMPH NODE, MESENTERIC**		N	N	N	N	N
**OVARY**		N	N	N	N	N
**LYMPH NODE, ILIAC**		N	N	N	N	N

Appendix 10

Microscopic Data Report

Study ID: 02776-0018: CRL 2954-001 / U of Tex Southwestern Med UTSW.Gray-002
Study Title: A SINGLE DOSE TOXICITY STUDY OF AAV9/SURF1 ADMINISTERED BY INTRATHECAL INJECTION IN RATS

DAY 08 SACRIFICE<br>GROUP 2FD08<br>AAV9/SURF1 LOW DOSE (0.28E12 VG)	ANIMAL	2511	2512	2513	2514	2515
**Tissue**	Sex	F	F	F	F	F
Site	Fate	SS	SS	SS	SS	SS
Diagnosis	Time on Test	08	08	08	08	08
**NERVE ROOT, SPINAL, CERVICAL**		N	N	N	N	N
**GANGLION, DORSAL ROOT, CERVICAL**		N	N	N	N	N
**NERVE ROOT, SPINAL, THORACIC**		N	N	N	N	N
**GANGLION, DORSAL ROOT, THORACIC**		N	N	N	N	N
**NERVE ROOT, SPINAL, LUMBAR**		A	A	A	A	N
EPINEURIUM						
INFILTRATE, MONONUCLEAR CELLS		1	1	-	-	-
NERVE ROOT, VENTRAL						
DEGENERATION, NERVE FIBER		-	-	2	2	-
**GANGLION, DORSAL ROOT, LUMBAR**		A	N	N	N	N
GLIAL CELL						
HYPERTROPHY/HYPERPLASIA		1	-	-	-	-
**BRAIN, MENINGES**		N	N	N	N	N
**BRAIN, CEREBRAL CORTEX**		N	N	N	N	N
**BRAIN, OLFACTORY BULB**		N	N	N	N	N
**BRAIN, WHITE MATTER**		N	N	N	N	N
**BRAIN, VENTRICULAR SYSTEM**		N	N	N	N	N
**BRAIN, BASAL NUCLEI/STRIATUM**		N	N	N	N	N
**BRAIN, AMYGDALOID BODY**		N	N	N	N	N
**BRAIN, HIPPOCAMPUS**		N	N	N	N	N
**BRAIN, HYPOTHALAMUS**		N	N	N	N	N
**BRAIN, THALAMUS**		N	N	N	N	N
**BRAIN, MIDBRAIN**		N	N	N	N	N
**BRAIN, CEREBELLUM**		N	N	N	N	N
**BRAIN, PONS**		N	N	N	N	N
**BRAIN, MEDULLA OBLONGATA**		A	N	N	N	N
WHITE MATTER						
DEGENERATION, NERVE FIBER		1	-	-	-	-
**SPINAL CORD, CERVICAL**		N	N	N	N	N
**SPINAL CORD, THORACIC**		N	N	N	N	N
**SPINAL CORD, LUMBAR**		N	N	N	N	N
**NERVE, SCIATIC**		N	N	N	A	A
DEGENERATION, NERVE FIBER		-	-	-	1	1
**NERVE, TIBIAL**		N	N	N	A	A
DEGENERATION, NERVE FIBER		-	-	-	1	1
**EYE**		A	N	N	N	N
RETINAL ROSETTE		1	-	-	-	-
**NERVE, OPTIC**		N	N	N	N	N
**LYMPH NODE, MANDIBULAR**		N	N	N	N	N
**THYMUS**		N	N	N	N	N
**PANCREAS**		N	N	N	N	N
**LUNG**		N	N	N	A	A
INFLAMMATION		-	-	-	1	-
ALVEOLUS						

Appendix 10

Microscopic Data Report

Study ID: 02776-0018: CRL 2954-001 / U of Tex Southwestern Med UTSW.Gray-002
Study Title: A SINGLE DOSE TOXICITY STUDY OF AAV9/SURF1 ADMINISTERED BY INTRATHECAL INJECTION IN RATS

DAY 08 SACRIFICE<br>GROUP 2FD08<br>AAV9/SURF1 LOW DOSE (0.28E12 VG)	ANIMAL	2511	2512	2513	2514	2515
**Tissue**	**Sex**	F	F	F	F	F
Site	**Fate**	SS	SS	SS	SS	SS
Diagnosis	**Time on Test**	08	08	08	08	08
INFILTRATE, MACROPHAGES		-	-	-	-	1
**MUSCLE, BICEPS FEMORIS**		N	N	N	N	N
**MUSCLE, GASTROCNEMIUS**		N	N	N	N	N
**HEART**		N	N	N	N	N
**SPLEEN**		N	N	N	N	N
**LIVER**		A	N	A	A	A
INFILTRATE, MIXED		1	-	1	1	1
**KIDNEY**		A	N	N	N	N
TUBULAR						
CYST		1	-	-	-	-
**LYMPH NODE, MESENTERIC**		N	N	N	N	N
**OVARY**		N	N	N	N	N
**LYMPH NODE, ILIAC**		N	N	N	N	N

Appendix 10

Microscopic Data Report

Study ID: 02776-0018: CRL 2954-001 / U of Tex Southwestern Med UTSW.Gray-002
Study Title: A SINGLE DOSE TOXICITY STUDY OF AAV9/SURF1 ADMINISTERED BY INTRATHECAL INJECTION IN RATS

DAY 08 SACRIFICE<br>GROUP 3FD08<br>AAV9/SURF1 MID DOSE (0.83E12 VG)	ANIMAL	3511	3512	3513	3514	3515
**Tissue**	Sex	F	F	F	F	F
Site	Fate	SS	SS	SS	SS	SS
Diagnosis	Time on Test	08	08	08	08	08
**NERVE ROOT, SPINAL, CERVICAL**		N	N	N	N	N
**GANGLION, DORSAL ROOT, CERVICAL**		N	N	N	N	N
**NERVE ROOT, SPINAL, THORACIC**		N	N	N	N	N
**GANGLION, DORSAL ROOT, THORACIC**		N	N	N	N	N
**NERVE ROOT, SPINAL, LUMBAR**		A	N	A	N	A
EPINEURIUM						
INFILTRATE, MONONUCLEAR CELLS		1	-	1	-	1
**GANGLION, DORSAL ROOT, LUMBAR**		N	N	A	N	N
INFILTRATE, MONONUCLEAR CELLS		-	-	1	-	-
**BRAIN, MENINGES**		N	N	N	N	N
**BRAIN, CEREBRAL CORTEX**		N	N	N	N	N
**BRAIN, OLFACTORY BULB**		N	N	N	N	N
**BRAIN, WHITE MATTER**		N	N	N	N	N
**BRAIN, VENTRICULAR SYSTEM**		N	N	N	N	N
**BRAIN, BASAL NUCLEI/STRIATUM**		N	N	N	N	N
**BRAIN, AMYGDALOID BODY**		N	N	N	N	N
**BRAIN, HIPPOCAMPUS**		N	N	N	N	N
**BRAIN, HYPOTHALAMUS**		N	N	N	N	N
**BRAIN, THALAMUS**		N	N	N	N	N
**BRAIN, MIDBRAIN**		N	N	N	N	N
**BRAIN, CEREBELLUM**		N	N	N	N	N
**BRAIN, PONS**		N	N	N	N	N
**BRAIN, MEDULLA OBLONGATA**		N	N	N	N	N
**SPINAL CORD, CERVICAL**		N	N	N	N	N
**SPINAL CORD, THORACIC**		N	A	N	N	A
GLIAL CELL						
INCREASED CELLULARITY		-	2	-	-	-
GRAY MATTER						
DEGENERATION/NECROSIS		-	2	-	-	-
INFILTRATE, MONONUCLEAR CELLS		-	1	-	-	-
WHITE MATTER						
DEGENERATION, NERVE FIBER		-	1	-	-	1
**SPINAL CORD, LUMBAR**		N	N	N	A	N
NERVE ROOT, SPINAL						
DEGENERATION, NERVE FIBER		-	-	-	1	-
**NERVE, SCIATIC**		N	N	N	N	A
DEGENERATION, NERVE FIBER		-	-	-	-	1
**NERVE, TIBIAL**		N	N	N	A	A
DEGENERATION, NERVE FIBER		-	-	-	1	1
**EYE**		N	N	N	N	N
**NERVE, OPTIC**		N	N	N	N	N
**LYMPH NODE, MANDIBULAR**		N	N	N	N	N
**THYMUS**		N	N	N	N	N
**PANCREAS**		N	N	N	N	N

Appendix 10

Microscopic Data Report

Study ID: 02776-0018: CRL 2954-001 / U of Tex Southwestern Med UTSW.Gray-002
Study Title: A SINGLE DOSE TOXICITY STUDY OF AAV9/SURF1 ADMINISTERED BY INTRATHECAL INJECTION IN RATS

DAY 08 SACRIFICE<br>GROUP 3FD08<br>AAV9/SURF1 MID DOSE (0.83E12 VG)	ANIMAL	3511	3512	3513	3514	3515
**Tissue**	**Sex**	F	F	F	F	F
Site	**Fate**	SS	SS	SS	SS	SS
Diagnosis	**Time on Test**	08	08	08	08	08
**LUNG**		N	N	N	N	N
**MUSCLE, BICEPS FEMORIS**		N	N	N	N	N
**MUSCLE, GASTROCNEMIUS**		N	N	N	N	N
**HEART**		A	N	N	N	N
MYOCARDIUM						
DEGENERATION/NECROSIS		1	-	-	-	-
INFILTRATE, MONONUCLEAR CELLS		1	-	-	-	-
**SPLEEN**		N	N	N	N	N
**LIVER**		A	A	A	A	A
EXTRAMEDULLARY HEMATOPOIESIS		-	1	-	-	1
INFILTRATE, MIXED		1	1	1	1	1
**KIDNEY**		A	N	N	A	N
CHRONIC PROGRESSIVE NEPHROPATHY		1	-	-	-	-
TUBULAR						
BASOPHILIA		-	-	-	1	-
**LYMPH NODE, MESENTERIC**		N	N	N	N	N
**OVARY**		N	N	N	N	N
**LYMPH NODE, ILIAC**		N	N	N	N	N

Appendix 10

Microscopic Data Report

Study ID: 02776-0018: CRL 2954-001 / U of Tex Southwestern Med UTSW.Gray-002
Study Title: A SINGLE DOSE TOXICITY STUDY OF AAV9/SURF1 ADMINISTERED BY INTRATHECAL INJECTION IN RATS

DAY 08 SACRIFICE<br>GROUP 4FD08<br>AAV9/SURF1 HIGH DOSE (2.49E12 VG)	ANIMAL	4511	4512	4513	4514	4515
**Tissue**	Sex	F	F	F	F	F
Site	Fate	SS	SS	SS	SS	SS
Diagnosis	Time on Test	08	08	08	08	08
**NERVE ROOT, SPINAL, CERVICAL**		N	N	N	N	N
**GANGLION, DORSAL ROOT, CERVICAL**		A	N	N	N	A
GLIAL CELL						
HYPERTROPHY/HYPERPLASIA		1	-	-	-	1
**NERVE ROOT, SPINAL, THORACIC**		N	N	N	N	N
**GANGLION, DORSAL ROOT, THORACIC**		N	N	N	N	A
GLIAL CELL						
HYPERTROPHY/HYPERPLASIA		-	-	-	-	1
**NERVE ROOT, SPINAL, LUMBAR**		A	N	N	A	A
EPINEURIUM						
INFILTRATE, MONONUCLEAR CELLS		1	-	-	1	1
**GANGLION, DORSAL ROOT, LUMBAR**		A	N	N	A	A
GLIAL CELL						
HYPERTROPHY/HYPERPLASIA		1	-	-	1	1
**BRAIN, MENINGES**		N	N	N	N	N
**BRAIN, CEREBRAL CORTEX**		N	N	N	N	N
**BRAIN, OLFACTORY BULB**		N	N	N	N	N
**BRAIN, WHITE MATTER**		N	N	N	N	N
**BRAIN, VENTRICULAR SYSTEM**		N	N	N	N	N
**BRAIN, BASAL NUCLEI/STRIATUM**		N	N	N	N	N
**BRAIN, AMYGDALOID BODY**		N	N	N	N	N
**BRAIN, HIPPOCAMPUS**		N	N	N	N	N
**BRAIN, HYPOTHALAMUS**		N	N	N	N	N
**BRAIN, THALAMUS**		N	N	N	N	N
**BRAIN, MIDBRAIN**		N	N	N	N	N
**BRAIN, CEREBELLUM**		N	N	N	N	N
**BRAIN, PONS**		N	N	N	N	N
**BRAIN, MEDULLA OBLONGATA**		N	N	N	U	N
**SPINAL CORD, CERVICAL**		N	N	N	N	N
**SPINAL CORD, THORACIC**		N	N	N	N	N
**SPINAL CORD, LUMBAR**		N	N	N	N	N
**NERVE, SCIATIC**		N	N	N	N	N
**NERVE, TIBIAL**		N	N	N	N	N
**EYE**		N	N	N	N	N
**NERVE, OPTIC**		N	N	N	N	N
**LYMPH NODE, MANDIBULAR**		N	N	N	N	N
**THYMUS**		N	N	N	N	N
**PANCREAS**		N	N	N	N	N
**LUNG**		N	N	N	N	N
**MUSCLE, BICEPS FEMORIS**		N	N	N	N	N
**MUSCLE, GASTROCNEMIUS**		N	N	N	N	N
**HEART**		N	A	A	N	A
MYOCARDIUM						
DEGENERATION/NECROSIS		-	1	-	-	1

Appendix 10

Microscopic Data Report

Study ID: 02776-0018: CRL 2954-001 / U of Tex Southwestern Med UTSW.Gray-002
Study Title: A SINGLE DOSE TOXICITY STUDY OF AAV9/SURF1 ADMINISTERED BY INTRATHECAL INJECTION IN RATS

DAY 08 SACRIFICE<br>GROUP 4FD08<br>AAV9/SURF1 HIGH DOSE (2.49E12 VG)	ANIMAL	4511	4512	4513	4514	4515
**Tissue**	**Sex**	F	F	F	F	F
Site	**Fate**	SS	SS	SS	SS	SS
Diagnosis	**Time on Test**	08	08	08	08	08
INFILTRATE, MONONUCLEAR CELLS		-	1	1	-	1
**SPLEEN**		N	N	N	N	N
**LIVER**		A	A	A	A	A
HYPERTROPHY/HYPERPLASIA		-	-	1	-	-
INFILTRATE, MIXED		1	1	1	1	1
HEPATOCYTE						
VACUOLATION		-	-	1	-	-
**KIDNEY**		A	N	N	N	N
CHRONIC PROGRESSIVE NEPHROPATHY		1	-	-	-	-
**LYMPH NODE, MESENTERIC**		N	N	N	N	N
**OVARY**		N	N	N	N	N
**LYMPH NODE, ILIAC**		U	N	N	N	N

Appendix 10

Microscopic Data Report

Study ID: 02776-0018: CRL 2954-001 / U of Tex Southwestern Med UTSW.Gray-002
Study Title: A SINGLE DOSE TOXICITY STUDY OF AAV9/SURF1 ADMINISTERED BY INTRATHECAL INJECTION IN RATS

DAY 29 SACRIFICE<br>GROUP 1MD29<br>VEHICLE (0 VG)	ANIMAL	1021	1022	1023	1024	1025
**Tissue**	Sex	M	M	M	M	M
Site	Fate	SS	SS	SS	SS	SS
Diagnosis	Time on Test	29	29	29	29	29
**NERVE ROOT, SPINAL, CERVICAL**		N	N	N	N	N
**GANGLION, DORSAL ROOT, CERVICAL**		A	N	N	A	A
GLIAL CELL						
HYPERTROPHY/HYPERPLASIA		1	-	-	1	1
**NERVE ROOT, SPINAL, THORACIC**		N	N	N	N	N
**GANGLION, DORSAL ROOT, THORACIC**		N	N	N	N	N
**NERVE ROOT, SPINAL, LUMBAR**		A	N	A	A	N
EPINEURIUM						
INFILTRATE, MONONUCLEAR CELLS		1	-	-	1	-
NERVE ROOT, DORSAL						
DEGENERATION, NERVE FIBER		-	-	1	-	-
**GANGLION, DORSAL ROOT, LUMBAR**		A	N	N	A	A
GLIAL CELL						
HYPERTROPHY/HYPERPLASIA		1	-	-	1	1
**BRAIN, MENINGES**		N	N	N	N	N
**BRAIN, CEREBRAL CORTEX**		N	N	N	N	N
**BRAIN, OLFACTORY BULB**		N	N	N	N	N
**BRAIN, WHITE MATTER**		N	N	N	N	N
**BRAIN, VENTRICULAR SYSTEM**		N	N	N	N	N
**BRAIN, BASAL NUCLEI/STRIATUM**		N	N	N	N	N
**BRAIN, AMYGDALOID BODY**		N	N	N	N	N
**BRAIN, HIPPOCAMPUS**		N	N	N	N	N
**BRAIN, HYPOTHALAMUS**		N	N	N	N	N
**BRAIN, THALAMUS**		N	N	N	N	N
**BRAIN, MIDBRAIN**		N	N	N	N	N
**BRAIN, CEREBELLUM**		N	N	N	N	N
**BRAIN, PONS**		N	N	N	N	N
**BRAIN, MEDULLA OBLONGATA**		N	N	A	N	N
WHITE MATTER						
DEGENERATION, NERVE FIBER		-	-	1	-	-
**SPINAL CORD, CERVICAL**		N	N	N	A	N
WHITE MATTER						
DEGENERATION, NERVE FIBER		-	-	-	1	-
**SPINAL CORD, THORACIC**		N	N	N	N	N
**SPINAL CORD, LUMBAR**		N	N	N	N	A
WHITE MATTER						
DEGENERATION, NERVE FIBER		-	-	-	-	1
**NERVE, SCIATIC**		N	N	N	N	N
**NERVE, TIBIAL**		N	N	N	N	N
**EYE**		N	N	N	N	N
**NERVE, OPTIC**		N	N	N	N	N
**LYMPH NODE, MANDIBULAR**		N	N	N	N	N
**THYMUS**		N	N	N	N	N
**PANCREAS**		N	N	N	N	N

Appendix 10

Microscopic Data Report

Study ID: 02776-0018: CRL 2954-001 / U of Tex Southwestern Med UTSW.Gray-002
Study Title: A SINGLE DOSE TOXICITY STUDY OF AAV9/SURF1 ADMINISTERED BY INTRATHECAL INJECTION IN RATS

DAY 29 SACRIFICE<br>GROUP 1MD29<br>VEHICLE (0 VG)	ANIMAL	1021	1022	1023	1024	1025
**Tissue**	**Sex**	M	M	M	M	M
Site	**Fate**	SS	SS	SS	SS	SS
Diagnosis	**Time on Test**	29	29	29	29	29
**LUNG**		N	N	N	N	N
**MUSCLE, BICEPS FEMORIS**		N	N	N	N	N
**MUSCLE, GASTROCNEMIUS**		N	N	N	N	N
**HEART**		N	N	A	N	N
MYOCARDIUM						
DEGENERATION/NECROSIS		-	-	1	-	-
INFILTRATE, MONONUCLEAR CELLS		-	-	1	-	-
**SPLEEN**		N	N	N	N	N
**LIVER**		A	A	A	A	N
INFILTRATE, MIXED		1	1	1	1	-
**KIDNEY**		A	A	A	N	A
CHRONIC PROGRESSIVE NEPHROPATHY		1	1	1	-	1
TUBULAR						
CYST		-	-	-	-	1
**LYMPH NODE, MESENTERIC**		N	N	N	N	N
**TESTIS**		N	N	N	A	N
TUBULAR						
DEGENERATION		-	-	-	1	-
**LYMPH NODE, ILIAC**		N	U	N	N	N

Appendix 10

Microscopic Data Report

Study ID: 02776-0018: CRL 2954-001 / U of Tex Southwestern Med UTSW.Gray-002
Study Title: A SINGLE DOSE TOXICITY STUDY OF AAV9/SURF1 ADMINISTERED BY INTRATHECAL INJECTION IN RATS

DAY 29 SACRIFICE<br>GROUP 2MD29<br>AAV9/SURF1 LOW DOSE (0.28E12 VG)	ANIMAL	2021	2022	2023	2024	2025
**Tissue**	Sex	M	M	M	M	M
Site	Fate	SS	SS	SS	SS	SS
Diagnosis	Time on Test	29	29	29	29	29
**NERVE ROOT, SPINAL, CERVICAL**		N	N	N	N	N
**GANGLION, DORSAL ROOT, CERVICAL**		N	N	N	N	N
**NERVE ROOT, SPINAL, THORACIC**		N	N	N	N	N
**GANGLION, DORSAL ROOT, THORACIC**		N	N	N	N	N
**NERVE ROOT, SPINAL, LUMBAR**		A	A	A	N	A
EPINEURIUM						
INFILTRATE, MONONUCLEAR CELLS		1	1	1	-	1
NERVE ROOT, DORSAL						
DEGENERATION, NERVE FIBER		1	-	1	-	-
**GANGLION, DORSAL ROOT, LUMBAR**		A	A	A	N	A
INFILTRATE, MONONUCLEAR CELLS		-	1	-	-	1
GLIAL CELL						
HYPERTROPHY/HYPERPLASIA		1	-	1	-	-
NEURON						
DEGENERATION/NECROSIS		-	-	-	-	1
**BRAIN, MENINGES**		N	N	A	A	N
INFILTRATE, MONONUCLEAR CELLS		-	-	1	1	-
**BRAIN, CEREBRAL CORTEX**		N	N	N	N	N
**BRAIN, OLFACTORY BULB**		N	N	N	N	N
**BRAIN, WHITE MATTER**		N	N	N	N	N
**BRAIN, VENTRICULAR SYSTEM**		N	N	N	N	N
**BRAIN, BASAL NUCLEI/STRIATUM**		N	N	N	N	N
**BRAIN, AMYGDALOID BODY**		N	N	N	N	N
**BRAIN, HIPPOCAMPUS**		N	N	N	N	N
**BRAIN, HYPOTHALAMUS**		N	N	N	N	N
**BRAIN, THALAMUS**		N	N	N	N	N
**BRAIN, MIDBRAIN**		N	N	A	N	N
PINEAL GLAND						
INFILTRATE, MONONUCLEAR CELLS		-	-	1	-	-
**BRAIN, CEREBELLUM**		N	N	N	N	N
**BRAIN, PONS**		N	N	N	N	N
**BRAIN, MEDULLA OBLONGATA**		N	N	N	N	N
**SPINAL CORD, CERVICAL**		N	N	N	A	A
GLIAL CELL						
INCREASED CELLULARITY		-	-	-	1	-
WHITE MATTER						
DEGENERATION, NERVE FIBER		-	-	-	-	1
**SPINAL CORD, THORACIC**		N	N	A	N	N
WHITE MATTER						
DEGENERATION, NERVE FIBER		-	-	1	-	-
**SPINAL CORD, LUMBAR**		N	N	N	N	A
NERVE ROOT, SPINAL						
DEGENERATION, NERVE FIBER		-	-	-	-	1

Appendix 10

Microscopic Data Report

Study ID: 02776-0018: CRL 2954-001 / U of Tex Southwestern Med UTSW.Gray-002
Study Title: A SINGLE DOSE TOXICITY STUDY OF AAV9/SURF1 ADMINISTERED BY INTRATHECAL INJECTION IN RATS

DAY 29 SACRIFICE<br>GROUP 2MD29<br>AAV9/SURF1 LOW DOSE (0.28E12 VG)	ANIMAL	2021	2022	2023	2024	2025
**Tissue**	Sex	M	M	M	M	M
Site	Fate	SS	SS	SS	SS	SS
Diagnosis	Time on Test	29	29	29	29	29
**NERVE, SCIATIC**		A	A	A	N	A
DEGENERATION, NERVE FIBER		1	1	1	-	1
**NERVE, TIBIAL**		N	N	A	N	A
DEGENERATION, NERVE FIBER		-	-	1	-	2
**EYE**		N	N	N	N	N
**NERVE, OPTIC**		N	N	N	N	N
**LYMPH NODE, MANDIBULAR**		N	N	N	N	N
**THYMUS**		N	N	N	N	N
**PANCREAS**		N	N	N	A	N
FIBROSIS		-	-	-	1	-
**LUNG**		N	N	N	N	A
PERIVASCULAR						
INFILTRATE, EOSINOPHILS		-	-	-	-	1
**MUSCLE, BICEPS FEMORIS**		N	N	N	N	N
**MUSCLE, GASTROCNEMIUS**		N	N	N	N	N
**HEART**		A	A	A	A	A
MYOCARDIUM						
DEGENERATION/NECROSIS		1	1	2	4	1
FIBROSIS		-	-	1	2	-
INFILTRATE, MONONUCLEAR CELLS		1	1	2	4	1
**SPLEEN**		N	N	N	N	N
**LIVER**		A	A	A	A	A
INFILTRATE, MIXED		1	1	1	1	1
HEPATOCYTE						
VACUOLATION		-	-	-	2	-
PERIVASCULAR						
INFILTRATE, MONONUCLEAR CELLS		-	1	-	-	-
**KIDNEY**		N	A	N	A	A
CHRONIC PROGRESSIVE NEPHROPATHY		-	-	-	1	1
TUBULAR						
BASOPHILIA		-	1	-	-	-
**LYMPH NODE, MESENTERIC**		N	N	N	N	N
**TESTIS**		N	N	N	N	N
**LYMPH NODE, ILIAC**		N	N	N	N	N

Appendix 10

Microscopic Data Report

Study ID: 02776-0018: CRL 2954-001 / U of Tex Southwestern Med UTSW.Gray-002
Study Title: A SINGLE DOSE TOXICITY STUDY OF AAV9/SURF1 ADMINISTERED BY INTRATHECAL INJECTION IN RATS

DAY 29 SACRIFICE<br>GROUP 3MD29<br>AAV9/SURF1 MID DOSE (0.83E12 VG)	ANIMAL	3021	3022	3023	3024	3025
**Tissue**	**Sex**	M	M	M	M	M
Site	**Fate**	SS	SS	SS	SS	SS
Diagnosis	**Time on Test**	29	29	29	29	29
**NERVE ROOT, SPINAL, CERVICAL**		N	N	N	N	N
**GANGLION, DORSAL ROOT, CERVICAL**		A	A	A	A	N
INFILTRATE, MONONUCLEAR CELLS		1	-	-	-	-
GLIAL CELL						
HYPERTROPHY/HYPERPLASIA		1	1	1	1	-
**NERVE ROOT, SPINAL, THORACIC**		N	N	U	N	N
**GANGLION, DORSAL ROOT, THORACIC**		N	N	U	A	N
GLIAL CELL						
HYPERTROPHY/HYPERPLASIA		-	-	-	1	-
**NERVE ROOT, SPINAL, LUMBAR**		A	A	A	A	N
EPINEURIUM						
INFILTRATE, MONONUCLEAR CELLS		1	1	-	1	-
NERVE ROOT, DORSAL						
DEGENERATION, NERVE FIBER		-	1	1	-	-
NERVE ROOT, VENTRAL						
DEGENERATION, NERVE FIBER		-	-	1	1	-
**GANGLION, DORSAL ROOT, LUMBAR**		A	A	A	A	A
INFILTRATE, MONONUCLEAR CELLS		1	1	-	1	1
GLIAL CELL						
HYPERTROPHY/HYPERPLASIA		1	1	1	1	1
**BRAIN, MENINGES**		N	N	N	N	N
**BRAIN, CEREBRAL CORTEX**		N	N	N	N	N
**BRAIN, OLFACTORY BULB**		N	N	N	N	N
**BRAIN, WHITE MATTER**		N	N	N	N	N
**BRAIN, VENTRICULAR SYSTEM**		N	N	N	N	N
**BRAIN, BASAL NUCLEI/STRIATUM**		N	N	N	N	N
**BRAIN, AMYGDALOID BODY**		N	N	N	N	N
**BRAIN, HIPPOCAMPUS**		N	N	N	N	N
**BRAIN, HYPOTHALAMUS**		N	N	N	N	N
**BRAIN, THALAMUS**		N	N	N	N	N
**BRAIN, MIDBRAIN**		N	N	A	N	N
PINEAL GLAND						
INFILTRATE, MONONUCLEAR CELLS		-	-	1	-	-
**BRAIN, CEREBELLUM**		N	N	N	N	N
**BRAIN, PONS**		N	N	N	N	N
**BRAIN, MEDULLA OBLONGATA**		N	N	N	N	N
**SPINAL CORD, CERVICAL**		N	N	N	N	N
**SPINAL CORD, THORACIC**		A	A	A	N	N
GRAY MATTER						
INFLAMMATION		-	-	1	-	-
MENINGES						
INFILTRATE, MONONUCLEAR CELLS		-	-	1	-	-
WHITE MATTER						

Appendix 10

Microscopic Data Report

Study ID: 02776-0018: CRL 2954-001 / U of Tex Southwestern Med UTSW.Gray-002
Study Title: A SINGLE DOSE TOXICITY STUDY OF AAV9/SURF1 ADMINISTERED BY INTRATHECAL INJECTION IN RATS

DAY 29 SACRIFICE<br>GROUP 3MD29<br>AAV9/SURF1 MID DOSE (0.83E12 VG)	ANIMAL	3021	3022	3023	3024	3025
**Tissue**	**Sex**	M	M	M	M	M
Site	**Fate**	SS	SS	SS	SS	SS
Diagnosis	**Time on Test**	29	29	29	29	29
DEGENERATION, NERVE FIBER		1	1	2	-	-
**SPINAL CORD, LUMBAR**		A	A	A	N	N
NERVE ROOT, SPINAL						
DEGENERATION, NERVE FIBER		-	-	2	-	-
WHITE MATTER						
DEGENERATION, NERVE FIBER		1	2	1	-	-
**NERVE, SCIATIC**		A	A	A	A	N
DEGENERATION, NERVE FIBER		3	2	1	1	-
**NERVE, TIBIAL**		A	A	N	N	N
DEGENERATION, NERVE FIBER		3	3	-	-	-
SCHWANN CELL						
HYPERTROPHY/HYPERPLASIA		1	1	-	-	-
**EYE**		N	N	N	N	N
**NERVE, OPTIC**		N	N	N	N	N
**LYMPH NODE, MANDIBULAR**		N	N	N	N	N
**THYMUS**		N	N	N	N	N
**PANCREAS**		N	N	N	A	N
ATROPHY		-	-	-	1	-
**LUNG**		A	N	N	N	A
INFLAMMATION		1	-	-	-	-
ALVEOLUS						
INFILTRATE, MACROPHAGES		-	-	-	-	1
**MUSCLE, BICEPS FEMORIS**		N	N	N	N	N
**MUSCLE, GASTROCNEMIUS**		N	N	N	N	N
**HEART**		A	A	A	A	A
MYOCARDIUM						
DEGENERATION/NECROSIS		3	1	4	2	2
FIBROSIS		-	-	1	1	-
INFILTRATE, MONONUCLEAR CELLS		3	1	4	2	2
**SPLEEN**		N	N	N	N	N
**LIVER**		A	A	A	A	A
INFILTRATE, MIXED		1	2	1	1	1
**KIDNEY**		N	A	N	A	A
CHRONIC PROGRESSIVE NEPHROPATHY		-	1	-	1	-
TUBULAR						
CYST		-	-	-	-	1
**LYMPH NODE, MESENTERIC**		N	N	N	N	N
**TESTIS**		N	N	N	N	N
**LYMPH NODE, ILIAC**		N	N	N	N	N

Appendix 10

Microscopic Data Report

Study ID: 02776-0018: CRL 2954-001 / U of Tex Southwestern Med UTSW.Gray-002
Study Title: A SINGLE DOSE TOXICITY STUDY OF AAV9/SURF1 ADMINISTERED BY INTRATHECAL INJECTION IN RATS

DAY 29 SACRIFICE<br>GROUP 4MD29<br>AAV9/SURF1 HIGH DOSE (2.49E12 VG)	ANIMAL	4023	4024	4025	4026	4027
**Tissue**	Sex	M	M	M	M	M
Site	Fate	SS	SS	SS	SS	SS
Diagnosis	Time on Test	29	29	29	29	29
**NERVE ROOT, SPINAL, CERVICAL**		N	N	N	N	N
**GANGLION, DORSAL ROOT, CERVICAL**		N	A	A	N	A
GLIAL CELL						
HYPERTROPHY/HYPERPLASIA		-	1	1	-	1
**NERVE ROOT, SPINAL, THORACIC**		N	A	N	N	N
NERVE ROOT, DORSAL						
DEGENERATION, NERVE FIBER		-	1	-	-	-
**GANGLION, DORSAL ROOT, THORACIC**		A	A	A	N	N
GLIAL CELL						
HYPERTROPHY/HYPERPLASIA		1	1	1	-	-
**NERVE ROOT, SPINAL, LUMBAR**		A	A	N	A	A
EPINEURIUM						
INFILTRATE, MONONUCLEAR CELLS		-	1	-	1	1
NERVE ROOT, DORSAL						
DEGENERATION, NERVE FIBER		1	2	-	1	-
**GANGLION, DORSAL ROOT, LUMBAR**		A	A	A	A	A
INFILTRATE, MONONUCLEAR CELLS		1	2	-	-	-
GLIAL CELL						
HYPERTROPHY/HYPERPLASIA		1	2	1	1	1
NEURON						
DEGENERATION/NECROSIS		-	1	-	-	-
**BRAIN, MENINGES**		N	N	N	N	N
**BRAIN, CEREBRAL CORTEX**		N	N	N	N	N
**BRAIN, OLFACTORY BULB**		N	N	N	N	N
**BRAIN, WHITE MATTER**		N	N	N	N	N
**BRAIN, VENTRICULAR SYSTEM**		N	N	N	N	N
**BRAIN, BASAL NUCLEI/STRIATUM**		N	N	N	N	N
**BRAIN, AMYGDALOID BODY**		N	N	N	N	N
**BRAIN, HIPPOCAMPUS**		N	N	N	N	N
**BRAIN, HYPOTHALAMUS**		N	N	N	N	N
**BRAIN, THALAMUS**		N	N	N	N	N
**BRAIN, MIDBRAIN**		N	N	N	N	N
**BRAIN, CEREBELLUM**		N	N	N	N	N
**BRAIN, PONS**		N	N	N	N	N
**BRAIN, MEDULLA OBLONGATA**		N	N	N	N	N
**SPINAL CORD, CERVICAL**		N	N	N	N	N
**SPINAL CORD, THORACIC**		N	A	N	A	N
WHITE MATTER						
DEGENERATION, NERVE FIBER		-	1	-	1	-
**SPINAL CORD, LUMBAR**		A	A	A	A	N
NERVE ROOT, SPINAL						
DEGENERATION, NERVE FIBER		-	-	1	1	-
WHITE MATTER						

Microscopic Data Report

Study ID: 02776-0018: CRL 2954-001 / U of Tex Southwestern Med UTSW.Gray-002
Study Title: A SINGLE DOSE TOXICITY STUDY OF AAV9/SURF1 ADMINISTERED BY INTRATHECAL INJECTION IN RATS

DAY 29 SACRIFICE<br>GROUP 4MD29<br>AAV9/SURF1 HIGH DOSE (2.49E12 VG)	ANIMAL	4023	4024	4025	4026	4027
**Tissue**	**Sex**	M	M	M	M	M
Site	**Fate**	SS	SS	SS	SS	SS
Diagnosis	**Time on Test**	29	29	29	29	29
DEGENERATION, NERVE FIBER		1	1	-	-	-
**NERVE, SCIATIC**		A	A	N	N	N
DEGENERATION, NERVE FIBER		3	2	-	-	-
SCHWANN CELL						
HYPERTROPHY/HYPERPLASIA		2	-	-	-	-
**NERVE, TIBIAL**		A	A	N	N	N
DEGENERATION, NERVE FIBER		3	2	-	-	-
SCHWANN CELL						
HYPERTROPHY/HYPERPLASIA		2	1	-	-	-
**EYE**		N	N	N	N	N
**NERVE, OPTIC**		N	N	N	N	N
**LYMPH NODE, MANDIBULAR**		N	N	N	N	N
**THYMUS**		N	N	N	N	N
**PANCREAS**		N	N	A	N	N
APOPTOSIS/SINGLE CELL NECROSIS		-	-	1	-	-
**LUNG**		N	N	A	N	N
INFLAMMATION		-	-	1	-	-
**MUSCLE, BICEPS FEMORIS**		N	N	N	N	N
**MUSCLE, GASTROCNEMIUS**		N	N	N	N	N
**HEART**		A	A	A	A	A
MYOCARDIUM						
DEGENERATION/NECROSIS		1	1	1	2	1
INFILTRATE, MONONUCLEAR CELLS		1	1	1	2	1
**SPLEEN**		N	N	N	N	N
**LIVER**		A	A	A	A	A
HYPERTROPHY/HYPERPLASIA		-	-	2	-	1
INFILTRATE, MIXED		2	1	2	1	2
HEPATOCYTE						
NECROSIS, SINGLE CELL		1	-	2	-	1
**KIDNEY**		A	A	N	A	A
CHRONIC PROGRESSIVE NEPHROPATHY		-	1	-	-	1
TUBULAR						
BASOPHILIA		1	-	-	-	-
CYST		-	-	-	1	-
**LYMPH NODE, MESENTERIC**		N	N	N	N	N
**TESTIS**		N	N	N	N	N
**LYMPH NODE, ILIAC**		N	N	N	N	N

Appendix 10

Microscopic Data Report

Study ID: 02776-0018: CRL 2954-001 / U of Tex Southwestern Med UTSW.Gray-002
Study Title: A SINGLE DOSE TOXICITY STUDY OF AAV9/SURF1 ADMINISTERED BY INTRATHECAL INJECTION IN RATS

DAY 29 SACRIFICE<br>GROUP 1FD29<br>VEHICLE (0 VG)	ANIMAL	1521	1522	1523	1524	1525
**Tissue**	Sex	F	F	F	F	F
Site	Fate	SS	SS	SS	SS	SS
Diagnosis	Time on Test	29	29	29	29	29
**NERVE ROOT, SPINAL, CERVICAL**		A	N	N	N	N
EPINEURIUM						
INFILTRATE, MONONUCLEAR CELLS		1	-	-	-	-
**GANGLION, DORSAL ROOT, CERVICAL**		N	N	N	N	A
GLIAL CELL						
HYPERTROPHY/HYPERPLASIA		-	-	-	-	1
**NERVE ROOT, SPINAL, THORACIC**		N	N	N	N	A
NERVE ROOT, VENTRAL						
DEGENERATION, NERVE FIBER		-	-	-	-	1
**GANGLION, DORSAL ROOT, THORACIC**		N	N	N	A	N
GLIAL CELL						
HYPERTROPHY/HYPERPLASIA		-	-	-	1	-
**NERVE ROOT, SPINAL, LUMBAR**		N	A	A	N	N
EPINEURIUM						
INFILTRATE, MONONUCLEAR CELLS		-	1	1	-	-
**GANGLION, DORSAL ROOT, LUMBAR**		N	A	N	N	N
INFILTRATE, MONONUCLEAR CELLS		-	1	-	-	-
**BRAIN, MENINGES**		N	N	N	N	N
**BRAIN, CEREBRAL CORTEX**		N	N	N	N	N
**BRAIN, OLFACTORY BULB**		N	N	N	N	N
**BRAIN, WHITE MATTER**		N	N	N	N	N
**BRAIN, VENTRICULAR SYSTEM**		N	N	N	N	N
**BRAIN, BASAL NUCLEI/STRIATUM**		N	N	N	N	N
**BRAIN, AMYGDALOID BODY**		N	N	N	N	N
**BRAIN, HIPPOCAMPUS**		N	N	N	N	N
**BRAIN, HYPOTHALAMUS**		N	N	N	N	N
**BRAIN, THALAMUS**		N	N	N	N	N
**BRAIN, MIDBRAIN**		N	N	N	N	N
**BRAIN, CEREBELLUM**		N	N	N	N	N
**BRAIN, PONS**		N	N	N	N	N
**BRAIN, MEDULLA OBLONGATA**		N	N	N	N	N
**SPINAL CORD, CERVICAL**		N	N	N	N	N
**SPINAL CORD, THORACIC**		N	N	N	N	N
**SPINAL CORD, LUMBAR**		N	N	N	N	N
**NERVE, SCIATIC**		N	N	N	N	N
**NERVE, TIBIAL**		N	N	N	N	N
**EYE**		N	N	N	N	N
**NERVE, OPTIC**		N	N	N	N	N
**LYMPH NODE, MANDIBULAR**		N	N	N	N	N
**THYMUS**		N	N	N	N	N
**PANCREAS**		N	N	N	N	N
**LUNG**		N	N	A	A	N
INFLAMMATION		-	-	1	-	-
PERIVASCULAR						

Appendix 10

Microscopic Data Report

Study ID: 02776-0018: CRL 2954-001 / U of Tex Southwestern Med UTSW.Gray-002
Study Title: A SINGLE DOSE TOXICITY STUDY OF AAV9/SURF1 ADMINISTERED BY INTRATHECAL INJECTION IN RATS

DAY 29 SACRIFICE<br>GROUP 1FD29<br>VEHICLE (0 VG)	ANIMAL	1521	1522	1523	1524	1525
**Tissue**	**Sex**	F	F	F	F	F
Site	**Fate**	SS	SS	SS	SS	SS
Diagnosis	**Time on Test**	29	29	29	29	29
INFILTRATE, EOSINOPHILS		-	-	-	1	-
**MUSCLE, BICEPS FEMORIS**		N	N	N	N	N
**MUSCLE, GASTROCNEMIUS**		N	N	N	N	N
**HEART**		N	N	N	N	N
**SPLEEN**		N	N	N	N	N
**LIVER**		A	N	N	A	A
INFILTRATE, MIXED		1	-	-	1	1
HEPATOCYTE						
VACUOLATION		1	-	-	-	-
**KIDNEY**		N	A	N	N	A
CHRONIC PROGRESSIVE NEPHROPATHY		-	-	-	-	1
TUBULAR						
BASOPHILIA		-	1	-	-	-
**LYMPH NODE, MESENTERIC**		N	N	N	N	N
**OVARY**		N	N	N	N	N
**LYMPH NODE, ILIAC**		N	N	N	N	N

Appendix 10

Microscopic Data Report

Study ID: 02776-0018: CRL 2954-001 / U of Tex Southwestern Med UTSW.Gray-002
Study Title: A SINGLE DOSE TOXICITY STUDY OF AAV9/SURF1 ADMINISTERED BY INTRATHECAL INJECTION IN RATS

DAY 29 SACRIFICE<br>GROUP 2FD29<br>AAV9/SURF1 LOW DOSE (0.28E12 VG)	ANIMAL	2521	2522	2523	2524	2525
**Tissue**	Sex	F	F	F	F	F
Site	Fate	SS	SS	SS	SS	SS
Diagnosis	Time on Test	29	29	29	29	29
**NERVE ROOT, SPINAL, CERVICAL**		N	N	N	N	N
**GANGLION, DORSAL ROOT, CERVICAL**		N	N	N	N	N
**NERVE ROOT, SPINAL, THORACIC**		N	N	N	N	N
**GANGLION, DORSAL ROOT, THORACIC**		N	N	N	N	N
**NERVE ROOT, SPINAL, LUMBAR**		N	N	A	A	N
NERVE ROOT, VENTRAL						
DEGENERATION, NERVE FIBER		-	-	1	1	-
**GANGLION, DORSAL ROOT, LUMBAR**		N	N	N	N	N
**BRAIN, MENINGES**		N	N	N	N	N
**BRAIN, CEREBRAL CORTEX**		N	N	N	N	N
**BRAIN, OLFACTORY BULB**		N	N	N	N	N
**BRAIN, WHITE MATTER**		N	N	N	N	N
**BRAIN, VENTRICULAR SYSTEM**		N	N	N	N	N
**BRAIN, BASAL NUCLEI/STRIATUM**		N	N	N	N	N
**BRAIN, AMYGDALOID BODY**		N	N	N	N	N
**BRAIN, HIPPOCAMPUS**		N	N	N	N	N
**BRAIN, HYPOTHALAMUS**		N	N	N	N	N
**BRAIN, THALAMUS**		N	N	N	N	N
**BRAIN, MIDBRAIN**		N	N	N	N	N
**BRAIN, CEREBELLUM**		N	N	N	N	N
**BRAIN, PONS**		N	N	N	N	N
**BRAIN, MEDULLA OBLONGATA**		N	N	N	N	N
**SPINAL CORD, CERVICAL**		N	N	N	N	N
**SPINAL CORD, THORACIC**		A	N	N	N	A
WHITE MATTER						
DEGENERATION, NERVE FIBER		1	-	-	-	1
**SPINAL CORD, LUMBAR**		A	A	A	N	N
WHITE MATTER						
DEGENERATION, NERVE FIBER		1	1	1	-	-
**NERVE, SCIATIC**		N	N	A	N	A
DEGENERATION, NERVE FIBER		-	-	2	-	1
**NERVE, TIBIAL**		N	N	A	N	N
DEGENERATION, NERVE FIBER		-	-	2	-	-
**EYE**		N	N	N	N	N
**NERVE, OPTIC**		N	N	N	N	N
**LYMPH NODE, MANDIBULAR**		N	N	N	N	N
**THYMUS**		N	N	N	N	N
**PANCREAS**		N	N	N	N	N
**LUNG**		N	N	N	N	A
INFLAMMATION		-	-	-	-	2
**MUSCLE, BICEPS FEMORIS**		N	N	N	N	N
**MUSCLE, GASTROCNEMIUS**		N	N	N	N	N
**HEART**		A	A	A	A	A
MYOCARDIUM						

Appendix 10

Microscopic Data Report

Study ID: 02776-0018: CRL 2954-001 / U of Tex Southwestern Med UTSW.Gray-002
Study Title: A SINGLE DOSE TOXICITY STUDY OF AAV9/SURF1 ADMINISTERED BY INTRATHECAL INJECTION IN RATS

DAY 29 SACRIFICE<br>GROUP 2FD29<br>AAV9/SURF1 LOW DOSE (0.28E12 VG)	ANIMAL	2521	2522	2523	2524	2525
**Tissue**	**Sex**	F	F	F	F	F
Site	**Fate**	SS	SS	SS	SS	SS
Diagnosis	**Time on Test**	29	29	29	29	29
DEGENERATION/NECROSIS		2	1	1	2	4
FIBROSIS		1	-	-	-	3
INFILTRATE, MONONUCLEAR CELLS		2	1	1	2	4
**SPLEEN**		N	N	N	N	N
**LIVER**		A	A	N	A	A
INFILTRATE, MIXED		1	2	-	1	1
**KIDNEY**		N	N	N	N	N
**LYMPH NODE, MESENTERIC**		N	N	N	N	N
**OVARY**		N	N	N	N	N
**LYMPH NODE, ILIAC**		N	N	N	N	N

Appendix 10

Microscopic Data Report

Study ID: 02776-0018: CRL 2954-001 / U of Tex Southwestern Med UTSW.Gray-002
Study Title: A SINGLE DOSE TOXICITY STUDY OF AAV9/SURF1 ADMINISTERED BY INTRATHECAL INJECTION IN RATS

DAY 29 SACRIFICE<br>GROUP 3FD29<br>AAV9/SURF1 MID DOSE (0.83E12 VG)	ANIMAL	3521	3522	3523	3524	3525
**Tissue**	Sex	F	F	F	F	F
Site	Fate	SS	SS	SS	SS	SS
Diagnosis	Time on Test	29	29	29	29	29
**NERVE ROOT, SPINAL, CERVICAL**		N	N	N	N	N
**GANGLION, DORSAL ROOT, CERVICAL**		A	N	N	N	A
GLIAL CELL						
HYPERTROPHY/HYPERPLASIA		1	-	-	-	1
**NERVE ROOT, SPINAL, THORACIC**		N	N	N	N	N
**GANGLION, DORSAL ROOT, THORACIC**		N	N	A	N	N
GLIAL CELL						
HYPERTROPHY/HYPERPLASIA		-	-	1	-	-
**NERVE ROOT, SPINAL, LUMBAR**		A	N	A	A	A
EPINEURIUM						
INFILTRATE, MONONUCLEAR CELLS		-	-	-	-	1
NERVE ROOT, DORSAL						
DEGENERATION, NERVE FIBER		-	-	3	1	1
NERVE ROOT, VENTRAL						
DEGENERATION, NERVE FIBER		1	-	-	-	3
**GANGLION, DORSAL ROOT, LUMBAR**		A	A	A	A	A
INFILTRATE, MONONUCLEAR CELLS		-	1	1	1	1
GLIAL CELL						
HYPERTROPHY/HYPERPLASIA		1	1	1	1	-
**BRAIN, MENINGES**		N	N	N	N	A
INFILTRATE, MONONUCLEAR CELLS		-	-	-	-	1
**BRAIN, CEREBRAL CORTEX**		N	N	N	N	N
**BRAIN, OLFACTORY BULB**		N	N	N	N	N
**BRAIN, WHITE MATTER**		N	N	N	N	N
**BRAIN, VENTRICULAR SYSTEM**		N	N	N	N	N
**BRAIN, BASAL NUCLEI/STRIATUM**		N	N	N	N	N
**BRAIN, AMYGDALOID BODY**		N	N	N	N	N
**BRAIN, HIPPOCAMPUS**		N	N	N	N	N
**BRAIN, HYPOTHALAMUS**		N	N	N	N	N
**BRAIN, THALAMUS**		N	N	N	N	N
**BRAIN, MIDBRAIN**		N	N	N	N	N
**BRAIN, CEREBELLUM**		N	N	N	N	A
WHITE MATTER						
DEGENERATION, NERVE FIBER		-	-	-	-	2
**BRAIN, PONS**		A	N	N	N	A
DEGENERATION, NERVE FIBER		1	-	-	-	1
**BRAIN, MEDULLA OBLONGATA**		N	N	N	N	A
WHITE MATTER						
DEGENERATION, NERVE FIBER		-	-	-	-	2
**SPINAL CORD, CERVICAL**		A	N	N	N	A
INFILTRATE, MONONUCLEAR CELLS		1	-	-	-	-
GLIAL CELL						
INCREASED CELLULARITY		-	-	-	-	1

Appendix 10

Microscopic Data Report

Study ID: 02776-0018: CRL 2954-001 / U of Tex Southwestern Med UTSW.Gray-002
Study Title: A SINGLE DOSE TOXICITY STUDY OF AAV9/SURF1 ADMINISTERED BY INTRATHECAL INJECTION IN RATS

DAY 29 SACRIFICE<br>GROUP 3FD29<br>AAV9/SURF1 MID DOSE (0.83E12 VG)	ANIMAL	3521	3522	3523	3524	3525
**Tissue**	Sex	F	F	F	F	F
Site	Fate	SS	SS	SS	SS	SS
Diagnosis	Time on Test	29	29	29	29	29
WHITE MATTER						
DEGENERATION, NERVE FIBER		1	-	-	-	1
**SPINAL CORD, THORACIC**		A	A	A	A	A
GLIAL CELL						
INCREASED CELLULARITY		-	-	-	-	2
GRAY MATTER						
DEGENERATION/NECROSIS		-	-	-	-	5
INFILTRATE, MONONUCLEAR CELLS		-	-	-	-	1
WHITE MATTER						
DEGENERATION, NERVE FIBER		1	1	1	1	3
**SPINAL CORD, LUMBAR**		A	A	N	N	A
INFILTRATE, MONONUCLEAR CELLS		1	-	-	-	-
GLIAL CELL						
INCREASED CELLULARITY		-	-	-	-	2
GRAY MATTER						
DEGENERATION/NECROSIS		-	-	-	-	3
INFILTRATE, MONONUCLEAR CELLS		-	-	-	-	1
WHITE MATTER						
DEGENERATION, NERVE FIBER		1	1	-	-	3
**NERVE, SCIATIC**		A	A	A	A	A
DEGENERATION, NERVE FIBER		3	2	1	1	1
INFILTRATE, MIXED		-	1	-	-	-
**NERVE, TIBIAL**		A	A	A	A	N
DEGENERATION, NERVE FIBER		3	2	1	1	-
**EYE**		N	N	N	N	N
**NERVE, OPTIC**		N	N	N	U	N
**LYMPH NODE, MANDIBULAR**		N	N	N	N	N
**THYMUS**		N	N	N	N	U
**PANCREAS**		N	N	N	N	N
**LUNG**		N	N	N	N	N
**MUSCLE, BICEPS FEMORIS**		N	N	N	N	N
**MUSCLE, GASTROCNEMIUS**		N	N	N	N	N
**HEART**		A	A	A	N	A
MYOCARDIUM						
DEGENERATION/NECROSIS		3	2	1	-	2
FIBROSIS		-	1	-	-	-
INFILTRATE, MONONUCLEAR CELLS		3	2	1	-	2
**SPLEEN**		N	N	N	N	N
**LIVER**		A	A	A	A	A
HYPERTROPHY/HYPERPLASIA		-	-	1	-	-
INFILTRATE, MIXED		1	2	1	1	1
HEPATOCYTE						
NECROSIS, SINGLE CELL		-	-	1	-	-

Appendix 10

Microscopic Data Report

Study ID: 02776-0018: CRL 2954-001 / U of Tex Southwestern Med UTSW.Gray-002
Study Title: A SINGLE DOSE TOXICITY STUDY OF AAV9/SURF1 ADMINISTERED BY INTRATHECAL INJECTION IN RATS

DAY 29 SACRIFICE<br>GROUP 3FD29<br>AAV9/SURF1 MID DOSE (0.83E12 VG)	ANIMAL	3521	3522	3523	3524	3525
**Tissue**	Sex	F	F	F	F	F
Site	Fate	SS	SS	SS	SS	SS
Diagnosis	Time on Test	29	29	29	29	29
**KIDNEY**		N	N	N	N	N
**LYMPH NODE, MESENTERIC**		N	N	N	N	N
**OVARY**		N	N	N	N	N
**LYMPH NODE, ILIAC**		N	N	N	N	N

Appendix 10

Microscopic Data Report

Study ID: 02776-0018: CRL 2954-001 / U of Tex Southwestern Med UTSW.Gray-002
Study Title: A SINGLE DOSE TOXICITY STUDY OF AAV9/SURF1 ADMINISTERED BY INTRATHECAL INJECTION IN RATS

DAY 29 SACRIFICE<br>GROUP 4FD29<br>AAV9/SURF1 HIGH DOSE (2.49E12 VG)	ANIMAL	4521	4522	4523	4524	4525
**Tissue**	**Sex**	F	F	F	F	F
Site	**Fate**	SS	SS	SS	SS	SS
Diagnosis	**Time on Test**	29	29	29	29	29
**NERVE ROOT, SPINAL, CERVICAL**		N	N	N	N	N
**GANGLION, DORSAL ROOT, CERVICAL**		A	A	A	A	A
GLIAL CELL						
HYPERTROPHY/HYPERPLASIA		1	1	1	1	1
**NERVE ROOT, SPINAL, THORACIC**		N	N	N	N	N
**GANGLION, DORSAL ROOT, THORACIC**		A	N	N	N	N
GLIAL CELL						
HYPERTROPHY/HYPERPLASIA		1	-	-	-	-
**NERVE ROOT, SPINAL, LUMBAR**		A	A	A	A	N
EPINEURIUM						
INFILTRATE, MONONUCLEAR CELLS		1	-	1	-	-
NERVE ROOT, DORSAL						
DEGENERATION, NERVE FIBER		-	2	-	2	-
**GANGLION, DORSAL ROOT, LUMBAR**		A	A	A	A	A
INFILTRATE, MONONUCLEAR CELLS		-	-	-	1	-
GLIAL CELL						
HYPERTROPHY/HYPERPLASIA		1	1	1	1	1
**BRAIN, MENINGES**		N	N	A	N	N
INFILTRATE, MONONUCLEAR CELLS		-	-	1	-	-
**BRAIN, CEREBRAL CORTEX**		N	N	N	N	N
**BRAIN, OLFACTORY BULB**		N	N	N	N	N
**BRAIN, WHITE MATTER**		N	N	N	N	N
**BRAIN, VENTRICULAR SYSTEM**		N	N	N	N	N
**BRAIN, BASAL NUCLEI/STRIATUM**		N	N	N	N	N
**BRAIN, AMYGDALOID BODY**		N	N	N	N	N
**BRAIN, HIPPOCAMPUS**		N	N	N	N	N
**BRAIN, HYPOTHALAMUS**		N	N	N	N	N
**BRAIN, THALAMUS**		N	N	N	N	N
**BRAIN, MIDBRAIN**		N	N	N	A	N
PINEAL GLAND						
INFILTRATE, MONONUCLEAR CELLS		-	-	-	2	-
**BRAIN, CEREBELLUM**		N	N	N	N	N
**BRAIN, PONS**		N	N	N	N	N
**BRAIN, MEDULLA OBLONGATA**		N	N	N	N	N
**SPINAL CORD, CERVICAL**		N	N	N	A	N
MENINGES						
INFILTRATE, MONONUCLEAR CELLS		-	-	-	1	-
WHITE MATTER						
DEGENERATION, NERVE FIBER		-	-	-	1	-
**SPINAL CORD, THORACIC**		N	A	A	A	N
WHITE MATTER						
DEGENERATION, NERVE FIBER		-	1	1	1	-
**SPINAL CORD, LUMBAR**		A	N	A	A	N

Appendix 10

Microscopic Data Report

Study ID: 02776-0018: CRL 2954-001 / U of Tex Southwestern Med UTSW.Gray-002
Study Title: A SINGLE DOSE TOXICITY STUDY OF AAV9/SURF1 ADMINISTERED BY INTRATHECAL INJECTION IN RATS

DAY 29 SACRIFICE<br>GROUP 4FD29<br>AAV9/SURF1 HIGH DOSE (2.49E12 VG)	ANIMAL	4521	4522	4523	4524	4525
**Tissue**	**Sex**	F	F	F	F	F
Site	**Fate**	SS	SS	SS	SS	SS
Diagnosis	**Time on Test**	29	29	29	29	29
WHITE MATTER						
DEGENERATION, NERVE FIBER		1	-	1	1	-
**NERVE, SCIATIC**		A	A	A	A	N
DEGENERATION, NERVE FIBER		2	1	2	3	-
**NERVE, TIBIAL**		A	A	A	A	N
DEGENERATION, NERVE FIBER		2	1	2	3	-
**EYE**		N	N	N	N	N
**NERVE, OPTIC**		N	N	N	N	N
**LYMPH NODE, MANDIBULAR**		N	N	N	N	N
**THYMUS**		N	N	N	N	N
**PANCREAS**		N	N	A	N	N
APOPTOSIS/SINGLE CELL NECROSIS		-	-	1	-	-
**LUNG**		N	N	A	N	N
INFLAMMATION		-	-	1	-	-
**MUSCLE, BICEPS FEMORIS**		N	N	N	N	N
**MUSCLE, GASTROCNEMIUS**		N	N	N	N	N
**HEART**		A	A	A	A	N
MYOCARDIUM						
DEGENERATION/NECROSIS		2	1	4	1	-
FIBROSIS		-	-	1	-	-
INFILTRATE, MONONUCLEAR CELLS		2	1	4	1	-
**SPLEEN**		N	N	N	N	N
**LIVER**		A	A	A	A	A
INFILTRATE, MIXED		1	1	1	1	2
**KIDNEY**		A	N	N	A	N
CHRONIC PROGRESSIVE NEPHROPATHY		1	-	-	1	-
**LYMPH NODE, MESENTERIC**		N	N	N	N	N
**OVARY**		N	N	N	N	N
**LYMPH NODE, ILIAC**		N	N	N	N	N

Appendix 10

Microscopic Data Report

Study ID: 02776-0018: CRL 2954-001 / U of Tex Southwestern Med UTSW.Gray-002
Study Title: A SINGLE DOSE TOXICITY STUDY OF AAV9/SURF1 ADMINISTERED BY INTRATHECAL INJECTION IN RATS

DAY 91 SACRIFICE<br>GROUP 1MD91<br>VEHICLE (0 VG)	ANIMAL	1006	1007	1008	1009	1010
**Tissue**	Sex	M	M	M	M	M
Site	Fate	SS	SS	SS	SS	SS
Diagnosis	Time on Test	87	87	87	91	91
**NERVE ROOT, SPINAL, CERVICAL**		N	N	N	N	N
**GANGLION, DORSAL ROOT, CERVICAL**		N	N	N	N	N
**NERVE ROOT, SPINAL, THORACIC**		N	N	N	N	N
**GANGLION, DORSAL ROOT, THORACIC**		N	A	N	N	N
INFILTRATE, MONONUCLEAR CELLS		-	1	-	-	-
**NERVE ROOT, SPINAL, LUMBAR**		N	N	N	N	N
**GANGLION, DORSAL ROOT, LUMBAR**		A	A	A	N	N
GLIAL CELL						
HYPERTROPHY/HYPERPLASIA		1	1	1	-	-
**BRAIN, MENINGES**		N	N	N	N	N
**BRAIN, CEREBRAL CORTEX**		N	N	N	N	N
**BRAIN, OLFACTORY BULB**		N	N	N	N	N
**BRAIN, WHITE MATTER**		N	N	N	N	N
**BRAIN, VENTRICULAR SYSTEM**		N	N	N	N	N
**BRAIN, BASAL NUCLEI/STRIATUM**		N	N	N	N	N
**BRAIN, AMYGDALOID BODY**		N	N	N	N	N
**BRAIN, HIPPOCAMPUS**		N	N	N	N	N
**BRAIN, HYPOTHALAMUS**		N	N	N	N	N
**BRAIN, THALAMUS**		N	N	N	N	N
**BRAIN, MIDBRAIN**		N	N	N	N	N
**BRAIN, CEREBELLUM**		N	N	N	N	N
**BRAIN, PONS**		N	N	N	N	N
**BRAIN, MEDULLA OBLONGATA**		N	N	N	A	N
WHITE MATTER						
DEGENERATION, NERVE FIBER		-	-	-	1	-
**SPINAL CORD, CERVICAL**		N	N	N	N	N
**SPINAL CORD, THORACIC**		N	A	N	A	N
WHITE MATTER						
DEGENERATION, NERVE FIBER		-	1	-	1	-
**SPINAL CORD, LUMBAR**		A	A	N	N	N
NERVE ROOT, SPINAL						
DEGENERATION, NERVE FIBER		-	1	-	-	-
WHITE MATTER						
DEGENERATION, NERVE FIBER		1	-	-	-	-
**NERVE, SCIATIC**		N	N	A	N	N
DEGENERATION, NERVE FIBER		-	-	1	-	-
**NERVE, TIBIAL**		N	N	N	N	N
**EYE**		T	U	U	N	N
**NERVE, OPTIC**		T	N	N	N	N
**LYMPH NODE, MANDIBULAR**		N	N	N	N	U
**THYMUS**		N	N	A	N	N
INCREASED CELLULARITY		-	-	1	-	-
**PANCREAS**		N	N	A	N	A
FIBROSIS		-	-	-	-	1

Appendix 10

Microscopic Data Report

Study ID: 02776-0018: CRL 2954-001 / U of Tex Southwestern Med UTSW.Gray-002
Study Title: A SINGLE DOSE TOXICITY STUDY OF AAV9/SURF1 ADMINISTERED BY INTRATHECAL INJECTION IN RATS

DAY 91 SACRIFICE GROUP 1MD91 VEHICLE (0 VG)	ANIMAL	1006	1007	1008	1009	1010
**Tissue**	**Sex**	M	M	M	M	M
Site	**Fate**	SS	SS	SS	SS	SS
Diagnosis	**Time on Test**	87	87	87	91	91
INFILTRATE, MONONUCLEAR CELLS		-	-	1	-	-
**LUNG**		N	N	A	N	N
ALVEOLUS						
INFILTRATE, MACROPHAGES		-	-	1	-	-
**MUSCLE, BICEPS FEMORIS**		N	N	N	N	N
**MUSCLE, GASTROCNEMIUS**		N	N	N	N	N
**HEART**		N	A	A	A	A
MYOCARDIUM						
DEGENERATION/NECROSIS		-	1	1	1	1
FIBROSIS		-	1	-	-	-
INFILTRATE, MONONUCLEAR CELLS		-	1	1	1	1
**SPLEEN**		N	N	N	N	N
**LIVER**		N	A	N	A	A
INFILTRATE, MIXED		-	1	-	1	1
NECROSIS		-	1	-	1	-
HEPATOCYTE						
VACUOLATION		-	1	-	-	-
**KIDNEY**		N	A	A	N	A
CHRONIC PROGRESSIVE NEPHROPATHY		-	1	-	-	-
TUBULAR						
ACCUMULATION, HYALINE DROPLETS		-	-	1	-	-
DILATATION		-	-	-	-	1
**LYMPH NODE, MESENTERIC**		N	N	N	N	N
**TESTIS**		N	N	N	N	N
**LYMPH NODE, ILIAC**		N	N	T	N	N

Appendix 10

Microscopic Data Report

Study ID: 02776-0018: CRL 2954-001 / U of Tex Southwestern Med UTSW.Gray-002
Study Title: A SINGLE DOSE TOXICITY STUDY OF AAV9/SURF1 ADMINISTERED BY INTRATHECAL INJECTION IN RATS

DAY 91 SACRIFICE<br>GROUP 2MD91<br>AAV9/SURF1 LOW DOSE (0.28E12 VG)	ANIMAL	2006	2007	2008	2009	2010
**Tissue**	**Sex**	M	M	M	M	M
Site	**Fate**	SS	SS	SS	SS	SS
Diagnosis	**Time on Test**	87	87	87	91	91
**NERVE ROOT, SPINAL, CERVICAL**		N	N	N	N	N
**GANGLION, DORSAL ROOT, CERVICAL**		N	N	A	A	N
INFILTRATE, MONONUCLEAR CELLS		-	-	-	1	-
GLIAL CELL						
HYPERTROPHY/HYPERPLASIA		-	-	1	1	-
**NERVE ROOT, SPINAL, THORACIC**		N	N	N	N	N
**GANGLION, DORSAL ROOT, THORACIC**		N	N	N	A	N
GLIAL CELL						
HYPERTROPHY/HYPERPLASIA		-	-	-	1	-
**NERVE ROOT, SPINAL, LUMBAR**		N	A	A	A	N
EPINEURIUM						
INFILTRATE, MONONUCLEAR CELLS		-	-	-	1	-
NERVE ROOT, DORSAL						
DEGENERATION, NERVE FIBER		-	2	1	-	-
**GANGLION, DORSAL ROOT, LUMBAR**		N	A	N	A	A
INFILTRATE, MONONUCLEAR CELLS		-	2	-	-	-
GLIAL CELL						
HYPERTROPHY/HYPERPLASIA		-	1	-	1	1
**BRAIN, MENINGES**		N	N	N	N	N
**BRAIN, CEREBRAL CORTEX**		N	N	N	N	N
**BRAIN, OLFACTORY BULB**		N	N	N	N	N
**BRAIN, WHITE MATTER**		N	N	N	N	N
**BRAIN, VENTRICULAR SYSTEM**		N	N	N	N	N
**BRAIN, BASAL NUCLEI/STRIATUM**		N	N	N	N	N
**BRAIN, AMYGDALOID BODY**		N	N	N	N	N
**BRAIN, HIPPOCAMPUS**		N	N	N	N	N
**BRAIN, HYPOTHALAMUS**		N	N	N	N	N
**BRAIN, THALAMUS**		N	N	N	N	N
**BRAIN, MIDBRAIN**		N	N	A	N	N
PINEAL GLAND						
VACUOLATION		-	-	3	-	-
**BRAIN, CEREBELLUM**		N	N	N	N	N
**BRAIN, PONS**		N	N	N	N	N
**BRAIN, MEDULLA OBLONGATA**		N	N	A	A	N
WHITE MATTER						
DEGENERATION, NERVE FIBER		-	-	1	1	-
**SPINAL CORD, CERVICAL**		A	A	N	N	N
WHITE MATTER						
DEGENERATION, NERVE FIBER		1	1	-	-	-
**SPINAL CORD, THORACIC**		A	A	N	A	A
GLIAL CELL						
INCREASED CELLULARITY		1	-	-	-	-
MENINGES						

Appendix 10

Microscopic Data Report

Study ID: 02776-0018: CRL 2954-001 / U of Tex Southwestern Med UTSW.Gray-002
Study Title: A SINGLE DOSE TOXICITY STUDY OF AAV9/SURF1 ADMINISTERED BY INTRATHECAL INJECTION IN RATS

DAY 91 SACRIFICE<br>GROUP 2MD91<br>AAV9/SURF1 LOW DOSE (0.28E12 VG)	ANIMAL	2006	2007	2008	2009	2010
**Tissue**	Sex	M	M	M	M	M
Site	Fate	SS	SS	SS	SS	SS
Diagnosis	Time on Test	87	87	87	91	91
INFILTRATE, MONONUCLEAR CELLS		-	1	-	-	-
NERVE ROOT, SPINAL						
DEGENERATION, NERVE FIBER		-	2	-	-	-
WHITE MATTER						
DEGENERATION, NERVE FIBER		-	-	-	1	1
**SPINAL CORD, LUMBAR**		A	N	N	N	N
WHITE MATTER						
DEGENERATION, NERVE FIBER		1	-	-	-	-
**NERVE, SCIATIC**		N	A	A	A	N
DEGENERATION, NERVE FIBER		-	1	1	1	-
**NERVE, TIBIAL**		N	A	N	N	N
DEGENERATION, NERVE FIBER		-	1	-	-	-
**EYE**		N	T	N	N	N
**NERVE, OPTIC**		N	T	N	N	N
**LYMPH NODE, MANDIBULAR**		N	N	N	N	N
**THYMUS**		N	N	N	N	N
**PANCREAS**		N	N	N	N	N
**LUNG**		N	N	N	A	N
ALVEOLUS						
INFILTRATE, MACROPHAGES		-	-	-	1	-
**MUSCLE, BICEPS FEMORIS**		N	N	N	N	N
**MUSCLE, GASTROCNEMIUS**		N	N	N	N	N
**HEART**		A	A	A	A	A
MYOCARDIUM						
DEGENERATION/NECROSIS		1	1	1	1	1
FIBROSIS		1	1	-	1	1
INFILTRATE, MONONUCLEAR CELLS		1	1	1	1	2
**SPLEEN**		N	N	N	N	N
**LIVER**		A	A	A	A	A
INFILTRATE, MIXED		1	1	1	1	2
NECROSIS		-	-	-	-	1
**KIDNEY**		A	A	N	A	A
CHRONIC PROGRESSIVE NEPHROPATHY		-	1	-	-	1
TUBULAR						
CYST		-	-	-	1	-
DILATATION		1	-	-	-	-
**LYMPH NODE, MESENTERIC**		N	N	N	N	N
**TESTIS**		N	N	N	N	N
**LYMPH NODE, ILIAC**		N	N	N	N	N

Appendix 10

Microscopic Data Report

Study ID: 02776-0018: CRL 2954-001 / U of Tex Southwestern Med UTSW.Gray-002
Study Title: A SINGLE DOSE TOXICITY STUDY OF AAV9/SURF1 ADMINISTERED BY INTRATHECAL INJECTION IN RATS

DAY 91 SACRIFICE<br>GROUP 3MD91<br>AAV9/SURF1 MID DOSE (0.83E12 VG)	ANIMAL	3006	3007	3009	3010
**Tissue**	Sex	M	M	M	M
Site	Fate	SS	SS	SS	SS
Diagnosis	Time on Test	87	87	91	91
**NERVE ROOT, SPINAL, CERVICAL**		N	N	N	N
**GANGLION, DORSAL ROOT, CERVICAL**		N	A	A	A
GLIAL CELL					
HYPERTROPHY/HYPERPLASIA		-	1	1	1
**NERVE ROOT, SPINAL, THORACIC**		N	N	N	N
**GANGLION, DORSAL ROOT, THORACIC**		A	A	A	A
INFILTRATE, MONONUCLEAR CELLS		1	-	1	-
GLIAL CELL					
HYPERTROPHY/HYPERPLASIA		-	1	1	1
**NERVE ROOT, SPINAL, LUMBAR**		N	N	N	A
NERVE ROOT, VENTRAL					
DEGENERATION, NERVE FIBER		-	-	-	1
**GANGLION, DORSAL ROOT, LUMBAR**		A	A	A	A
INFILTRATE, MONONUCLEAR CELLS		1	1	-	1
GLIAL CELL					
HYPERTROPHY/HYPERPLASIA		-	1	1	1
**BRAIN, MENINGES**		N	N	N	N
**BRAIN, CEREBRAL CORTEX**		N	N	N	N
**BRAIN, OLFACTORY BULB**		N	N	N	N
**BRAIN, WHITE MATTER**		N	N	N	N
**BRAIN, VENTRICULAR SYSTEM**		N	N	N	N
**BRAIN, BASAL NUCLEI/STRIATUM**		N	N	N	N
**BRAIN, AMYGDALOID BODY**		N	N	N	N
**BRAIN, HIPPOCAMPUS**		N	N	N	N
**BRAIN, HYPOTHALAMUS**		N	N	N	N
**BRAIN, THALAMUS**		N	N	N	N
**BRAIN, MIDBRAIN**		N	N	A	N
PINEAL GLAND					
INFILTRATE, MONONUCLEAR CELLS		-	-	1	-
**BRAIN, CEREBELLUM**		N	N	N	N
**BRAIN, PONS**		N	N	N	N
**BRAIN, MEDULLA OBLONGATA**		N	N	A	N
WHITE MATTER					
DEGENERATION, NERVE FIBER		-	-	1	-
**SPINAL CORD, CERVICAL**		N	A	A	N
WHITE MATTER					
DEGENERATION, NERVE FIBER		-	1	1	-
**SPINAL CORD, THORACIC**		A	A	A	N
WHITE MATTER					
DEGENERATION, NERVE FIBER		1	1	1	-
**SPINAL CORD, LUMBAR**		A	N	A	A
NERVE ROOT, SPINAL					
DEGENERATION, NERVE FIBER		-	-	-	2

Appendix 10

Microscopic Data Report

Study ID: 02776-0018: CRL 2954-001 / U of Tex Southwestern Med UTSW.Gray-002
Study Title: A SINGLE DOSE TOXICITY STUDY OF AAV9/SURF1 ADMINISTERED BY INTRATHECAL INJECTION IN RATS

DAY 91 SACRIFICE<br>GROUP 3MD91<br>AAV9/SURF1 MID DOSE (0.83E12 VG)	ANIMAL	3006	3007	3009	3010
**Tissue**	**Sex**	M	M	M	M
Site	**Fate**	SS	SS	SS	SS
Diagnosis	**Time on Test**	87	87	91	91
WHITE MATTER					
DEGENERATION, NERVE FIBER		1	-	1	-
**NERVE, SCIATIC**		A	A	A	A
DEGENERATION, NERVE FIBER		2	1	1	1
**NERVE, TIBIAL**		A	N	A	N
DEGENERATION, NERVE FIBER		2	-	1	-
**EYE**		N	N	N	A
RETINAL ROSETTE		-	-	-	4
**NERVE, OPTIC**		N	N	N	N
**LYMPH NODE, MANDIBULAR**		N	N	N	N
**THYMUS**		N	N	N	N
**PANCREAS**		N	N	A	N
INFILTRATE, MONONUCLEAR CELLS		-	-	1	-
**LUNG**		A	A	N	N
INFLAMMATION		-	1	-	-
ALVEOLUS					
INFILTRATE, MACROPHAGES		1	-	-	-
**MUSCLE, BICEPS FEMORIS**		N	N	N	N
**MUSCLE, GASTROCNEMIUS**		N	N	N	N
**HEART**		A	A	A	A
MYOCARDIUM					
DEGENERATION/NECROSIS		2	1	1	1
FIBROSIS		3	-	-	-
INFILTRATE, MONONUCLEAR CELLS		4	1	1	1
**SPLEEN**		N	N	N	N
**LIVER**		A	A	A	N
INFILTRATE, MIXED		1	1	1	-
NECROSIS		-	1	-	-
**KIDNEY**		N	A	A	N
CHRONIC PROGRESSIVE NEPHROPATHY		-	1	-	-
TUBULAR					
DILATATION		-	-	1	-
**LYMPH NODE, MESENTERIC**		N	N	N	N
**TESTIS**		N	N	N	N
**LYMPH NODE, ILIAC**		A	N	N	N
INCREASED CELLULARITY		1	-	-	-

Appendix 10

Microscopic Data Report

Study ID: 02776-0018: CRL 2954-001 / U of Tex Southwestern Med UTSW.Gray-002
Study Title: A SINGLE DOSE TOXICITY STUDY OF AAV9/SURF1 ADMINISTERED BY INTRATHECAL INJECTION IN RATS

DAY 91 SACRIFICE<br>GROUP 4MD91<br>AAV9/SURF1 HIGH DOSE (2.49E12 VG)	ANIMAL	4006	4007	4008	4009	4010
**Tissue**	Sex	M	M	M	M	M
Site	Fate	SS	SS	SS	SS	SS
Diagnosis	Time on Test	87	87	87	91	91
**NERVE ROOT, SPINAL, CERVICAL**		N	N	N	N	N
**GANGLION, DORSAL ROOT, CERVICAL**		A	N	A	N	N
GLIAL CELL						
HYPERTROPHY/HYPERPLASIA		1	-	1	-	-
**NERVE ROOT, SPINAL, THORACIC**		N	N	N	N	N
**GANGLION, DORSAL ROOT, THORACIC**		N	A	N	N	A
GLIAL CELL						
HYPERTROPHY/HYPERPLASIA		-	1	-	-	1
**NERVE ROOT, SPINAL, LUMBAR**		A	A	N	N	A
EPINEURIUM						
INFILTRATE, MONONUCLEAR CELLS		-	1	-	-	-
NERVE ROOT, DORSAL						
DEGENERATION, NERVE FIBER		1	2	-	-	-
NERVE ROOT, VENTRAL						
DEGENERATION, NERVE FIBER		-	-	-	-	1
**GANGLION, DORSAL ROOT, LUMBAR**		A	A	N	N	A
INFILTRATE, MONONUCLEAR CELLS		-	1	-	-	1
GLIAL CELL						
HYPERTROPHY/HYPERPLASIA		1	1	-	-	1
**BRAIN, MENINGES**		N	N	N	N	N
**BRAIN, CEREBRAL CORTEX**		N	N	N	N	N
**BRAIN, OLFACTORY BULB**		N	N	N	N	N
**BRAIN, WHITE MATTER**		N	N	N	N	N
**BRAIN, VENTRICULAR SYSTEM**		N	N	N	N	N
**BRAIN, BASAL NUCLEI/STRIATUM**		N	N	N	N	N
**BRAIN, AMYGDALOID BODY**		N	N	N	N	N
**BRAIN, HIPPOCAMPUS**		N	N	N	N	N
**BRAIN, HYPOTHALAMUS**		N	N	N	N	N
**BRAIN, THALAMUS**		N	N	N	N	N
**BRAIN, MIDBRAIN**		N	N	N	N	N
**BRAIN, CEREBELLUM**		N	N	N	N	N
**BRAIN, PONS**		N	N	N	N	N
**BRAIN, MEDULLA OBLONGATA**		N	N	N	N	A
WHITE MATTER						
DEGENERATION, NERVE FIBER		-	-	-	-	1
**SPINAL CORD, CERVICAL**		A	A	N	N	N
WHITE MATTER						
DEGENERATION, NERVE FIBER		1	1	-	-	-
**SPINAL CORD, THORACIC**		N	A	A	A	A
GLIAL CELL						
INCREASED CELLULARITY		-	1	-	-	-
NERVE ROOT, SPINAL						
DEGENERATION, NERVE FIBER		-	-	-	-	1

Appendix 10

Microscopic Data Report

Study ID: 02776-0018: CRL 2954-001 / U of Tex Southwestern Med UTSW.Gray-002
Study Title: A SINGLE DOSE TOXICITY STUDY OF AAV9/SURF1 ADMINISTERED BY INTRATHECAL INJECTION IN RATS

DAY 91 SACRIFICE<br>GROUP 4MD91<br>AAV9/SURF1 HIGH DOSE (2.49E12 VG)	ANIMAL	4006	4007	4008	4009	4010
**Tissue**	Sex	M	M	M	M	M
Site	Fate	SS	SS	SS	SS	SS
Diagnosis	Time on Test	87	87	87	91	91
WHITE MATTER						
DEGENERATION, NERVE FIBER		-	-	1	1	-
**SPINAL CORD, LUMBAR**		N	N	N	N	A
NERVE ROOT, SPINAL						
DEGENERATION, NERVE FIBER		-	-	-	-	1
**NERVE, SCIATIC**		A	A	N	N	A
DEGENERATION, NERVE FIBER		1	2	-	-	1
**NERVE, TIBIAL**		A	A	N	N	A
DEGENERATION, NERVE FIBER		1	1	-	-	1
**EYE**		N	N	N	A	N
RETINAL ROSETTE		-	-	-	2	-
**NERVE, OPTIC**		N	N	N	N	N
**LYMPH NODE, MANDIBULAR**		N	N	N	N	N
**THYMUS**		U	N	N	N	N
**PANCREAS**		A	N	A	A	A
ATROPHY		-	-	1	1	-
FIBROSIS		1	-	-	1	2
**LUNG**		A	N	N	A	A
INFLAMMATION		-	-	-	-	1
ALVEOLUS						
INFILTRATE, MACROPHAGES		1	-	-	1	-
**MUSCLE, BICEPS FEMORIS**		N	N	N	N	N
**MUSCLE, GASTROCNEMIUS**		N	N	N	N	N
**HEART**		A	A	A	A	A
MYOCARDIUM						
DEGENERATION/NECROSIS		1	1	1	-	1
FIBROSIS		2	2	2	1	1
INFILTRATE, MONONUCLEAR CELLS		2	2	2	-	1
**SPLEEN**		N	N	N	N	N
**LIVER**		A	A	A	A	A
INFILTRATE, MIXED		1	1	1	1	-
NECROSIS		-	-	-	-	1
**KIDNEY**		A	N	A	N	N
CHRONIC PROGRESSIVE NEPHROPATHY		1	-	-	-	-
TUBULAR						
BASOPHILIA		-	-	1	-	-
**LYMPH NODE, MESENTERIC**		N	N	N	N	N
**TESTIS**		N	N	N	N	N
**LYMPH NODE, ILIAC**		N	N	N	N	N

Appendix 10

Microscopic Data Report

Study ID: 02776-0018: CRL 2954-001 / U of Tex Southwestern Med UTSW.Gray-002
Study Title: A SINGLE DOSE TOXICITY STUDY OF AAV9/SURF1 ADMINISTERED BY INTRATHECAL INJECTION IN RATS

DAY 91 SACRIFICE<br>GROUP 1FD91<br>VEHICLE (0 VG)	ANIMAL	1506	1507	1508	1509	1510
**Tissue**	Sex	F	F	F	F	F
Site	Fate	SS	SS	SS	SS	SS
Diagnosis	Time on Test	90	90	90	91	91
**NERVE ROOT, SPINAL, CERVICAL**		N	N	N	N	N
**GANGLION, DORSAL ROOT, CERVICAL**		N	N	N	N	N
**NERVE ROOT, SPINAL, THORACIC**		N	N	N	N	N
**GANGLION, DORSAL ROOT, THORACIC**		N	N	N	N	N
**NERVE ROOT, SPINAL, LUMBAR**		A	N	N	N	N
EPINEURIUM						
INFILTRATE, MONONUCLEAR CELLS		1	-	-	-	-
**GANGLION, DORSAL ROOT, LUMBAR**		N	N	N	N	N
**BRAIN, MENINGES**		N	N	N	N	N
**BRAIN, CEREBRAL CORTEX**		N	N	N	N	N
**BRAIN, OLFACTORY BULB**		N	N	N	N	N
**BRAIN, WHITE MATTER**		N	N	N	N	N
**BRAIN, VENTRICULAR SYSTEM**		N	N	N	N	N
**BRAIN, BASAL NUCLEI/STRIATUM**		N	N	N	N	N
**BRAIN, AMYGDALOID BODY**		N	N	N	N	N
**BRAIN, HIPPOCAMPUS**		N	N	N	N	N
**BRAIN, HYPOTHALAMUS**		N	N	N	N	N
**BRAIN, THALAMUS**		N	N	N	N	N
**BRAIN, MIDBRAIN**		N	N	N	N	N
**BRAIN, CEREBELLUM**		N	N	N	N	N
**BRAIN, PONS**		N	N	N	N	N
**BRAIN, MEDULLA OBLONGATA**		N	N	N	N	N
**SPINAL CORD, CERVICAL**		N	N	N	N	N
**SPINAL CORD, THORACIC**		N	N	N	N	N
**SPINAL CORD, LUMBAR**		N	N	N	N	T
**NERVE, SCIATIC**		N	N	N	N	N
**NERVE, TIBIAL**		N	N	N	N	N
**EYE**		N	A	N	N	N
RETINAL ROSETTE		-	1	-	-	-
**NERVE, OPTIC**		N	N	N	N	N
**LYMPH NODE, MANDIBULAR**		N	N	N	N	N
**THYMUS**		A	A	N	N	N
INCREASED CELLULARITY		1	1	-	-	-
**PANCREAS**		N	N	N	N	N
**LUNG**		N	N	A	N	N
ALVEOLUS						
INFILTRATE, MACROPHAGES		-	-	1	-	-
**MUSCLE, BICEPS FEMORIS**		N	N	N	N	N
**MUSCLE, GASTROCNEMIUS**		N	N	N	N	N
**HEART**		N	N	N	A	A
MYOCARDIUM						
DEGENERATION/NECROSIS		-	-	-	-	1
INFILTRATE, MONONUCLEAR CELLS		-	-	-	1	1
**SPLEEN**		N	N	N	N	N

Appendix 10

Microscopic Data Report

Study ID: 02776-0018: CRL 2954-001 / U of Tex Southwestern Med UTSW.Gray-002
Study Title: A SINGLE DOSE TOXICITY STUDY OF AAV9/SURF1 ADMINISTERED BY INTRATHECAL INJECTION IN RATS

DAY 91 SACRIFICE<br>GROUP 1FD91<br>VEHICLE (0 VG)	ANIMAL	1506	1507	1508	1509	1510
**Tissue**	**Sex**	F	F	F	F	F
Site	**Fate**	SS	SS	SS	SS	SS
Diagnosis	**Time on Test**	90	90	90	91	91
**LIVER**		A	N	N	N	A
INFILTRATE, MIXED		1	-	-	-	1
**KIDNEY**		N	N	N	N	N
**LYMPH NODE, MESENTERIC**		N	A	N	N	N
PIGMENT		-	1	-	-	-
**OVARY**		N	N	N	N	N
**LYMPH NODE, ILIAC**		N	N	N	N	N

Appendix 10

Microscopic Data Report

Study ID: 02776-0018: CRL 2954-001 / U of Tex Southwestern Med UTSW.Gray-002
Study Title: A SINGLE DOSE TOXICITY STUDY OF AAV9/SURF1 ADMINISTERED BY INTRATHECAL INJECTION IN RATS

DAY 91 SACRIFICE<br>GROUP 2FD91<br>AAV9/SURF1 LOW DOSE (0.28E12 VG)	ANIMAL	2506	2507	2508	2509	2510
**Tissue**	**Sex**	F	F	F	F	F
Site	**Fate**	SS	SS	SS	SS	SS
Diagnosis	**Time on Test**	90	90	90	91	91
**NERVE ROOT, SPINAL, CERVICAL**		N	N	N	N	N
**GANGLION, DORSAL ROOT, CERVICAL**		A	N	N	N	A
INFILTRATE, MONONUCLEAR CELLS		1	-	-	-	-
GLIAL CELL						
HYPERTROPHY/HYPERPLASIA		-	-	-	-	1
**NERVE ROOT, SPINAL, THORACIC**		N	N	N	N	N
**GANGLION, DORSAL ROOT, THORACIC**		N	N	N	N	A
INFILTRATE, MONONUCLEAR CELLS		-	-	-	-	1
**NERVE ROOT, SPINAL, LUMBAR**		N	N	N	N	A
NERVE ROOT, VENTRAL						
DEGENERATION, NERVE FIBER		-	-	-	-	1
**GANGLION, DORSAL ROOT, LUMBAR**		A	A	A	N	A
GLIAL CELL						
HYPERTROPHY/HYPERPLASIA		1	1	1	-	1
**BRAIN, MENINGES**		N	N	N	N	N
**BRAIN, CEREBRAL CORTEX**		N	N	N	N	N
**BRAIN, OLFACTORY BULB**		N	N	N	N	N
**BRAIN, WHITE MATTER**		N	N	N	N	N
**BRAIN, VENTRICULAR SYSTEM**		N	N	N	N	N
**BRAIN, BASAL NUCLEI/STRIATUM**		N	N	N	N	N
**BRAIN, AMYGDALOID BODY**		N	N	N	N	N
**BRAIN, HIPPOCAMPUS**		N	N	N	N	N
**BRAIN, HYPOTHALAMUS**		N	N	N	N	N
**BRAIN, THALAMUS**		N	N	N	N	N
**BRAIN, MIDBRAIN**		N	N	N	N	A
PINEAL GLAND						
INFILTRATE, MONONUCLEAR CELLS		-	-	-	-	1
**BRAIN, CEREBELLUM**		N	N	N	N	N
**BRAIN, PONS**		N	N	N	N	N
**BRAIN, MEDULLA OBLONGATA**		N	N	N	A	N
WHITE MATTER						
DEGENERATION, NERVE FIBER		-	-	-	1	-
**SPINAL CORD, CERVICAL**		N	N	N	N	N
**SPINAL CORD, THORACIC**		A	N	N	N	A
WHITE MATTER						
DEGENERATION, NERVE FIBER		1	-	-	-	1
**SPINAL CORD, LUMBAR**		A	N	N	N	N
PIGMENT, HEMOSIDERIN		1	-	-	-	-
GLIAL CELL						
INCREASED CELLULARITY		2	-	-	-	-
**NERVE, SCIATIC**		N	A	A	N	N
DEGENERATION, NERVE FIBER		-	1	1	-	-
**NERVE, TIBIAL**		N	A	N	N	N

Appendix 10

Microscopic Data Report

Study ID: 02776-0018: CRL 2954-001 / U of Tex Southwestern Med UTSW.Gray-002
Study Title: A SINGLE DOSE TOXICITY STUDY OF AAV9/SURF1 ADMINISTERED BY INTRATHECAL INJECTION IN RATS

DAY 91 SACRIFICE<br>GROUP 2FD91<br>AAV9/SURF1 LOW DOSE (0.28E12 VG)	ANIMAL	2506	2507	2508	2509	2510
**Tissue**	**Sex**	F	F	F	F	F
Site	**Fate**	SS	SS	SS	SS	SS
Diagnosis	**Time on Test**	90	90	90	91	91
DEGENERATION, NERVE FIBER		-	1	-	-	-
**EYE**		N	N	N	N	N
**NERVE, OPTIC**		N	N	N	U	N
**LYMPH NODE, MANDIBULAR**		N	N	N	N	N
**THYMUS**		N	A	A	N	N
INCREASED CELLULARITY		-	1	1	-	-
**PANCREAS**		A	N	N	N	N
INFILTRATE, MONONUCLEAR CELLS		1	-	-	-	-
**LUNG**		N	A	N	N	N
ALVEOLUS						
INFILTRATE, MACROPHAGES		-	1	-	-	-
**MUSCLE, BICEPS FEMORIS**		N	N	N	N	N
**MUSCLE, GASTROCNEMIUS**		N	N	N	N	N
**HEART**		A	N	A	N	A
MYOCARDIUM						
DEGENERATION/NECROSIS		1	-	1	-	1
INFILTRATE, MONONUCLEAR CELLS		2	-	1	-	1
**SPLEEN**		N	N	N	N	N
**LIVER**		N	N	N	N	A
INFILTRATE, MIXED		-	-	-	-	1
**KIDNEY**		N	A	N	N	N
TUBULAR						
CYST		-	1	-	-	-
**LYMPH NODE, MESENTERIC**		N	N	N	N	N
**OVARY**		N	N	N	N	N
**LYMPH NODE, ILIAC**		N	N	N	N	N

Appendix 10

Microscopic Data Report

Study ID: 02776-0018: CRL 2954-001 / U of Tex Southwestern Med UTSW.Gray-002
Study Title: A SINGLE DOSE TOXICITY STUDY OF AAV9/SURF1 ADMINISTERED BY INTRATHECAL INJECTION IN RATS

DAY 91 SACRIFICE<br>GROUP 3FD91<br>AAV9/SURF1 MID DOSE (0.83E12 VG)	ANIMAL	3506	3507	3508	3509	3510
**Tissue**	Sex	F	F	F	F	F
Site	Fate	SS	SS	SS	SS	SS
Diagnosis	Time on Test	91	91	91	91	91
**NERVE ROOT, SPINAL, CERVICAL**		N	N	N	N	N
**GANGLION, DORSAL ROOT, CERVICAL**		N	N	A	A	A
GLIAL CELL						
HYPERTROPHY/HYPERPLASIA		-	-	1	1	1
**NERVE ROOT, SPINAL, THORACIC**		N	N	N	N	N
**GANGLION, DORSAL ROOT, THORACIC**		A	N	A	A	A
INFILTRATE, MONONUCLEAR CELLS		1	-	-	-	-
GLIAL CELL						
HYPERTROPHY/HYPERPLASIA		-	-	1	1	1
**NERVE ROOT, SPINAL, LUMBAR**		A	A	A	A	N
EPINEURIUM						
INFILTRATE, MONONUCLEAR CELLS		-	1	-	1	-
NERVE ROOT, VENTRAL						
DEGENERATION, NERVE FIBER		2	-	2	-	-
**GANGLION, DORSAL ROOT, LUMBAR**		A	A	A	A	N
INFILTRATE, MONONUCLEAR CELLS		1	1	-	1	-
GLIAL CELL						
HYPERTROPHY/HYPERPLASIA		1	1	1	1	-
**BRAIN, MENINGES**		A	N	N	N	N
INFILTRATE, MONONUCLEAR CELLS		1	-	-	-	-
**BRAIN, CEREBRAL CORTEX**		N	N	N	N	N
**BRAIN, OLFACTORY BULB**		N	N	N	N	N
**BRAIN, WHITE MATTER**		N	N	N	N	N
**BRAIN, VENTRICULAR SYSTEM**		N	N	N	N	N
**BRAIN, BASAL NUCLEI/STRIATUM**		N	N	N	N	N
**BRAIN, AMYGDALOID BODY**		N	N	N	N	N
**BRAIN, HIPPOCAMPUS**		N	N	N	N	N
**BRAIN, HYPOTHALAMUS**		N	N	N	N	N
**BRAIN, THALAMUS**		N	N	N	N	N
**BRAIN, MIDBRAIN**		A	N	N	N	N
PINEAL GLAND						
INFILTRATE, MONONUCLEAR CELLS		1	-	-	-	-
**BRAIN, CEREBELLUM**		N	N	N	N	N
**BRAIN, PONS**		N	N	N	N	N
**BRAIN, MEDULLA OBLONGATA**		N	N	N	N	A
WHITE MATTER						
DEGENERATION, NERVE FIBER		-	-	-	-	1
**SPINAL CORD, CERVICAL**		N	N	N	N	N
**SPINAL CORD, THORACIC**		A	N	N	A	A
WHITE MATTER						
DEGENERATION, NERVE FIBER		1	-	-	2	1
**SPINAL CORD, LUMBAR**		A	A	A	A	N
MENINGES						

Appendix 10

Microscopic Data Report

Study ID: 02776-0018: CRL 2954-001 / U of Tex Southwestern Med UTSW.Gray-002
Study Title: A SINGLE DOSE TOXICITY STUDY OF AAV9/SURF1 ADMINISTERED BY INTRATHECAL INJECTION IN RATS

DAY 91 SACRIFICE<br>GROUP 3FD91<br>AAV9/SURF1 MID DOSE (0.83E12 VG)	ANIMAL	3506	3507	3508	3509	3510
**Tissue**	**Sex**	**F**	**F**	**F**	**F**	**F**
Site	**Fate**	**SS**	**SS**	**SS**	**SS**	**SS**
Diagnosis	**Time on Test**	**91**	**91**	**91**	**91**	**91**
INFILTRATE, MONONUCLEAR CELLS		-	-	-	1	-
MINERALIZATION		-	-	-	1	-
NERVE ROOT, SPINAL						
DEGENERATION, NERVE FIBER		1	1	2	4	-
**NERVE, SCIATIC**		**A**	**N**	**A**	**A**	**N**
DEGENERATION, NERVE FIBER		1	-	1	2	-
SCHWANN CELL						
HYPERTROPHY/HYPERPLASIA		-	-	-	1	-
**NERVE, TIBIAL**		**A**	**N**	**A**	**A**	**N**
DEGENERATION, NERVE FIBER		1	-	1	2	-
SCHWANN CELL						
HYPERTROPHY/HYPERPLASIA		-	-	-	2	-
**EYE**		**N**	**N**	**N**	**N**	**N**
**NERVE, OPTIC**		**N**	**N**	**N**	**N**	**N**
**LYMPH NODE, MANDIBULAR**		**N**	**N**	**N**	**N**	**N**
**THYMUS**		**N**	**N**	**N**	**A**	**N**
INCREASED CELLULARITY		-	-	-	1	-
**PANCREAS**		**N**	**N**	**N**	**N**	**N**
**LUNG**		**N**	**N**	**N**	**N**	**N**
**MUSCLE, BICEPS FEMORIS**		**N**	**N**	**N**	**N**	**N**
**MUSCLE, GASTROCNEMIUS**		**N**	**N**	**N**	**N**	**N**
**HEART**		**A**	**A**	**A**	**A**	**A**
MYOCARDIUM						
DEGENERATION/NECROSIS		1	1	1	1	-
FIBROSIS		1	2	2	1	-
INFILTRATE, MONONUCLEAR CELLS		2	2	2	2	1
**SPLEEN**		**N**	**N**	**N**	**N**	**N**
**LIVER**		**N**	**N**	**N**	**N**	**A**
INFILTRATE, MIXED		-	-	-	-	1
NECROSIS		-	-	-	-	1
**KIDNEY**		**N**	**N**	**N**	**N**	**N**
**LYMPH NODE, MESENTERIC**		**N**	**N**	**N**	**N**	**N**
**OVARY**		**N**	**N**	**N**	**N**	**N**
**LYMPH NODE, ILIAC**		**N**	**N**	**N**	**N**	**N**

Appendix 10

Microscopic Data Report

Study ID: 02776-0018: CRL 2954-001 / U of Tex Southwestern Med UTSW.Gray-002
Study Title: A SINGLE DOSE TOXICITY STUDY OF AAV9/SURF1 ADMINISTERED BY INTRATHECAL INJECTION IN RATS

DAY 91 SACRIFICE<br>GROUP 4FD91<br>AAV9/SURF1 HIGH DOSE (2.49E12 VG)	ANIMAL	4506	4507	4508	4509	4510
**Tissue**	**Sex**	F	F	F	F	F
Site	**Fate**	SS	SS	SS	SS	SS
Diagnosis	**Time on Test**	91	91	91	91	91
**NERVE ROOT, SPINAL, CERVICAL**		N	N	N	N	N
**GANGLION, DORSAL ROOT, CERVICAL**		A	N	N	A	A
INFILTRATE, MONONUCLEAR CELLS		-	-	-	1	-
GLIAL CELL						
HYPERTROPHY/HYPERPLASIA		1	-	-	1	1
**NERVE ROOT, SPINAL, THORACIC**		N	N	N	N	N
**GANGLION, DORSAL ROOT, THORACIC**		N	A	A	A	N
INFILTRATE, MONONUCLEAR CELLS		-	-	1	-	-
GLIAL CELL						
HYPERTROPHY/HYPERPLASIA		-	1	-	1	-
**NERVE ROOT, SPINAL, LUMBAR**		A	A	A	A	N
EPINEURIUM						
INFILTRATE, MONONUCLEAR CELLS		1	-	1	-	-
NERVE ROOT, DORSAL						
DEGENERATION, NERVE FIBER		-	1	-	1	-
**GANGLION, DORSAL ROOT, LUMBAR**		A	A	A	A	A
INFILTRATE, MONONUCLEAR CELLS		-	1	-	1	1
GLIAL CELL						
HYPERTROPHY/HYPERPLASIA		1	1	1	1	-
**BRAIN, MENINGES**		N	N	N	N	N
**BRAIN, CEREBRAL CORTEX**		N	N	N	N	N
**BRAIN, OLFACTORY BULB**		N	N	N	N	N
**BRAIN, WHITE MATTER**		N	N	N	N	N
**BRAIN, VENTRICULAR SYSTEM**		N	N	N	N	N
**BRAIN, BASAL NUCLEI/STRIATUM**		N	N	N	N	N
**BRAIN, AMYGDALOID BODY**		N	N	N	N	N
**BRAIN, HIPPOCAMPUS**		N	N	N	N	N
**BRAIN, HYPOTHALAMUS**		N	N	N	N	N
**BRAIN, THALAMUS**		N	N	N	N	N
**BRAIN, MIDBRAIN**		N	A	N	N	N
PINEAL GLAND						
INFILTRATE, MONONUCLEAR CELLS		-	1	-	-	-
**BRAIN, CEREBELLUM**		N	N	N	N	N
**BRAIN, PONS**		N	N	N	N	N
**BRAIN, MEDULLA OBLONGATA**		N	A	N	A	N
WHITE MATTER						
DEGENERATION, NERVE FIBER		-	1	-	1	-
**SPINAL CORD, CERVICAL**		A	N	N	N	A
WHITE MATTER						
DEGENERATION, NERVE FIBER		1	-	-	-	1
**SPINAL CORD, THORACIC**		A	A	A	A	A
WHITE MATTER						
DEGENERATION, NERVE FIBER		1	1	1	1	1

Microscopic Data Report

Study ID: 02776-0018: CRL 2954-001 / U of Tex Southwestern Med UTSW.Gray-002
Study Title: A SINGLE DOSE TOXICITY STUDY OF AAV9/SURF1 ADMINISTERED BY INTRATHECAL INJECTION IN RATS

DAY 91 SACRIFICE<br>GROUP 4FD91<br>AAV9/SURF1 HIGH DOSE (2.49E12 VG)	ANIMAL	4506	4507	4508	4509	4510
**Tissue**	Sex	F	F	F	F	F
Site	Fate	SS	SS	SS	SS	SS
Diagnosis	Time on Test	91	91	91	91	91
**SPINAL CORD, LUMBAR**		A	A	N	N	A
NERVE ROOT, SPINAL						
DEGENERATION, NERVE FIBER		-	2	-	-	1
WHITE MATTER						
DEGENERATION, NERVE FIBER		1	-	-	-	-
**NERVE, SCIATIC**		A	A	N	A	A
DEGENERATION, NERVE FIBER		2	2	-	1	1
**NERVE, TIBIAL**		A	A	N	A	A
DEGENERATION, NERVE FIBER		2	2	-	1	1
**EYE**		N	N	N	N	N
**NERVE, OPTIC**		N	N	N	N	N
**LYMPH NODE, MANDIBULAR**		N	N	N	N	N
**THYMUS**		N	N	N	N	N
**PANCREAS**		N	N	N	N	N
**LUNG**		N	N	N	N	A
ALVEOLUS						
INFILTRATE, MACROPHAGES		-	-	-	-	1
**MUSCLE, BICEPS FEMORIS**		N	N	N	N	N
**MUSCLE, GASTROCNEMIUS**		N	N	N	N	N
**HEART**		A	A	A	A	A
MYOCARDIUM						
DEGENERATION/NECROSIS		1	1	1	1	1
FIBROSIS		-	-	1	-	1
INFILTRATE, MONONUCLEAR CELLS		1	1	1	1	1
**SPLEEN**		N	N	N	N	N
**LIVER**		A	N	A	A	A
INFILTRATE, MIXED		1	-	1	1	1
NECROSIS		-	-	-	-	1
**KIDNEY**		N	N	N	N	N
**LYMPH NODE, MESENTERIC**		N	N	N	N	N
**OVARY**		N	N	N	N	N
**LYMPH NODE, ILIAC**		N	N	N	N	N

Appendix 10

Microscopic Data Report

Study ID: 02776-0018: CRL 2954-001 / U of Tex Southwestern Med UTSW.Gray-002
Study Title: A SINGLE DOSE TOXICITY STUDY OF AAV9/SURF1 ADMINISTERED BY INTRATHECAL INJECTION IN RATS

US SACRIFICE<br>GROUP ED<br>EARLY DEATH (0.83E12 VG)	ANIMAL	3008
**Tissue**	Sex	M
Site	Fate	US
Diagnosis	Time on Test	16
**NERVE ROOT, SPINAL, CERVICAL**		N
**GANGLION, DORSAL ROOT, CERVICAL**		N
**NERVE ROOT, SPINAL, THORACIC**		A
EPINEURIUM		
INFILTRATE, MONONUCLEAR CELLS		1
NERVE ROOT, VENTRAL		
DEGENERATION, NERVE FIBER		2
SCHWANN CELL		
HYPERTROPHY/HYPERPLASIA		1
**GANGLION, DORSAL ROOT, THORACIC**		A
INFILTRATE, MONONUCLEAR CELLS		1
GLIAL CELL		
HYPERTROPHY/HYPERPLASIA		1
**NERVE ROOT, SPINAL, LUMBAR**		A
NERVE ROOT, VENTRAL		
DEGENERATION, NERVE FIBER		5
INFILTRATE, MONONUCLEAR CELLS		1
SCHWANN CELL		
HYPERTROPHY/HYPERPLASIA		3
**GANGLION, DORSAL ROOT, LUMBAR**		A
GLIAL CELL		
HYPERTROPHY/HYPERPLASIA		1
**BRAIN, MENINGES**		N
**BRAIN, CEREBRAL CORTEX**		N
**BRAIN, OLFACTORY BULB**		N
**BRAIN, WHITE MATTER**		N
**BRAIN, VENTRICULAR SYSTEM**		A
INFILTRATE, MONONUCLEAR CELLS		1
**BRAIN, BASAL NUCLEI/STRIATUM**		N
**BRAIN, AMYGDALOID BODY**		N
**BRAIN, HIPPOCAMPUS**		N
**BRAIN, HYPOTHALAMUS**		N
**BRAIN, THALAMUS**		N
**BRAIN, MIDBRAIN**		N
**BRAIN, CEREBELLUM**		N
**BRAIN, PONS**		N
**BRAIN, MEDULLA OBLONGATA**		N
**SPINAL CORD, CERVICAL**		A
GLIAL CELL		
INCREASED CELLULARITY		1
GRAY MATTER		
INFILTRATE, MONONUCLEAR CELLS		1
**SPINAL CORD, THORACIC**		A

Appendix 10

Microscopic Data Report

Study ID: 02776-0018: CRL 2954-001 / U of Tex Southwestern Med UTSW.Gray-002
Study Title: A SINGLE DOSE TOXICITY STUDY OF AAV9/SURF1 ADMINISTERED BY INTRATHECAL INJECTION IN RATS

US SACRIFICE<br>GROUP ED<br>EARLY DEATH (0.83E12 VG)	ANIMAL	3008
**Tissue**	**Sex**	**M**
Site	**Fate**	**US**
Diagnosis	**Time on Test**	**16**
GLIAL CELL		
INCREASED CELLULARITY		4
GRAY MATTER		
DEGENERATION/NECROSIS		5
INFILTRATE, MONONUCLEAR CELLS		2
WHITE MATTER		
DEGENERATION, NERVE FIBER		4
**SPINAL CORD, LUMBAR**		**A**
GLIAL CELL		
INCREASED CELLULARITY		3
GRAY MATTER		
DEGENERATION/NECROSIS		1
NERVE ROOT, SPINAL		
DEGENERATION, NERVE FIBER		1
WHITE MATTER		
DEGENERATION, NERVE FIBER		1
**NERVE, SCIATIC**		**A**
DEGENERATION, NERVE FIBER		2
SCHWANN CELL		
HYPERTROPHY/HYPERPLASIA		1
**NERVE, TIBIAL**		**A**
DEGENERATION, NERVE FIBER		2
**EYE**		**N**
**NERVE, OPTIC**		**N**
**LYMPH NODE, MANDIBULAR**		**N**
**THYMUS**		**N**
**PANCREAS**		**N**
**LUNG**		**N**
**MUSCLE, BICEPS FEMORIS**		**N**
**MUSCLE, GASTROCNEMIUS**		**N**
**HEART**		**A**
MYOCARDIUM		
DEGENERATION/NECROSIS		1
INFILTRATE, MONONUCLEAR CELLS		1
**SPLEEN**		**N**
**LIVER**		**A**
INFILTRATE, MIXED		1
**KIDNEY**		**A**
CHRONIC PROGRESSIVE NEPHROPATHY		1
**LYMPH NODE, MESENTERIC**		**N**
**TESTIS**		**N**
**LYMPH NODE, ILIAC**		**N**

DATA SECTION III: SUMMARY INCIDENCE AND AVERAGE SEVERITY REPORT

The Summary Incidence and Average Severity Report lists the incidence of microscopic changes and the group average severity for those changes.

- Only tissues for which microscopic findings were recorded are included in this report.
- Regardless of other modifiers (modifiers are not included in the diagnosis descriptions in this report), findings with the same Diagnosis at the same Tissue/Site are grouped together.
- The average severity is calculated according to the following formula: sum of severity grades for a specific diagnosis in a specific group divided by the number of times that specific tissue was examined in that group. Example: A group in which Tissue A was examined 5 times, with Diagnosis X diagnosed three times with severity grades of 0, 0, 2, 3, 4 (0=diagnosis was not observed) would have an average severity of: 9/5=1.80.

Appendix 10

Summary Incidence and Average Severity Report

Study ID: 02776-0018: CRL 2954-001 / U of Tex Southwestern Med UTSW.Gray-002
Study Title: A SINGLE DOSE TOXICITY STUDY OF AAV9/SURF1 ADMINISTERED BY INTRATHECAL INJECTION IN RATS

This is a summary report and only displays those tissues which have at least one finding present for this study.

Tissue / *Site* / Diagnosis	Sacrifice: DAY 08		DAY 08		DAY 08		DAY 08		DAY 08		DAY 08	
Group:	1MD08		2MD08		3MD08		4MD08		1FD08		2FD08	
Dose:	0 VG		0.28E12 VG		0.83E12 VG		2.49E12 VG		0 VG		0.28E12 VG	
	INC	SEV	INC	SEV	INC	SEV	INC	SEV	INC	SEV	INC	SEV
**NERVE ROOT, SPINAL, CERVICAL**	5		5		6		5		5		5	
EPINEURIUM												
INFILTRATE, MONONUCLEAR CELLS	–	–	–	–	–	–	–	–	–	–	–	–
NERVE ROOT, VENTRAL												
DEGENERATION, NERVE FIBER	–	–	–	–	–	–	1	0.20	–	–	–	–
**GANGLION, DORSAL ROOT, CERVICAL**	5		5		6		5		5		5	
INFILTRATE, MONONUCLEAR CELLS	–	–	–	–	1	0.17	–	–	–	–	–	–
GLIAL CELL												
HYPERTROPHY/HYPERPLASIA	1	0.20	3	0.60	–	–	–	–	1	0.20	–	–
**NERVE ROOT, SPINAL, THORACIC**	5		5		6		5		5		5	
EPINEURIUM												
INFILTRATE, MONONUCLEAR CELLS	–	–	–	–	–	–	–	–	–	–	–	–
NERVE ROOT, DORSAL												
DEGENERATION, NERVE FIBER	–	–	–	–	–	–	–	–	–	–	–	–
NERVE ROOT, VENTRAL												
DEGENERATION, NERVE FIBER	1	0.20	1	0.20	–	–	–	–	–	–	–	–
SCHWANN CELL												
HYPERTROPHY/HYPERPLASIA	–	–	–	–	–	–	–	–	–	–	–	–
**GANGLION, DORSAL ROOT, THORACIC**	5		5		6		5		5		5	
INFILTRATE, MONONUCLEAR CELLS	–	–	–	–	–	–	–	–	–	–	–	–
GLIAL CELL												
HYPERTROPHY/HYPERPLASIA	1	0.20	1	0.20	–	–	–	–	–	–	–	–
**NERVE ROOT, SPINAL, LUMBAR**	5		5		6		5		5		5	
EPINEURIUM												
INFILTRATE, MONONUCLEAR CELLS	–	–	1	0.20	3	0.50	–	–	–	–	2	0.40

Appendix 10

Summary Incidence and Average Severity Report

Study ID: 02776-0018: CRL 2954-001 / U of Tex Southwestern Med UTSW.Gray-002
Study Title: A SINGLE DOSE TOXICITY STUDY OF AAV9/SURF1 ADMINISTERED BY INTRATHECAL INJECTION IN RATS

This is a summary report and only displays those tissues which have at least one finding present for this study.

Tissue / *Site* / Diagnosis	INC	SEV	INC	SEV	INC	SEV	INC	SEV	INC	SEV	INC	SEV
**Sacrifice:**	DAY 08		DAY 08		DAY 08		DAY 08		DAY 08		DAY 08	
**Group:**	1MD08		2MD08		3MD08		4MD08		1FD08		2FD08	
**Dose:**	0 VG		0.28E12 VG		0.83E12 VG		2.49E12 VG		0 VG		0.28E12 VG	
NERVE ROOT, DORSAL												
DEGENERATION, NERVE FIBER	–	–	–	–	–	–	–	–	–	–	–	–
NERVE ROOT, VENTRAL												
DEGENERATION, NERVE FIBER	1	0.20	–	–	–	–	–	–	–	–	2	0.80
INFILTRATE, MONONUCLEAR CELLS	–	–	–	–	–	–	–	–	–	–	–	–
SCHWANN CELL												
HYPERTROPHY/HYPERPLASIA	–	–	–	–	–	–	–	–	–	–	–	–
**GANGLION, DORSAL ROOT, LUMBAR**	5		5		6		5		5		5	
INFILTRATE, MONONUCLEAR CELLS	–	–	**1**	**0.20**	2	0.33	–	–	–	–	–	–
GLIAL CELL												
HYPERTROPHY/HYPERPLASIA	1	0.20	4	0.80	3	0.50	–	–	1	0.20	1	0.20
NEURON												
DEGENERATION/NECROSIS	–	–	–	–	–	–	–	–	–	–	–	–
**BRAIN, MENINGES**	5		5		6		5		5		5	
INFILTRATE, MONONUCLEAR CELLS	–	–	–	–	–	–	–	–	–	–	–	–
**BRAIN, VENTRICULAR SYSTEM**	5		5		6		5		5		5	
INFILTRATE, MONONUCLEAR CELLS	–	–	–	–	–	–	–	–	–	–	–	–
**BRAIN, MIDBRAIN**	5		5		6		5		5		5	
PINEAL GLAND												
INFILTRATE, MONONUCLEAR CELLS	–	–	–	–	–	–	–	–	–	–	–	–
VACUOLATION	–	–	–	–	–	–	–	–	–	–	–	–
**BRAIN, CEREBELLUM**	5		5		6		5		5		5	
WHITE MATTER												
DEGENERATION, NERVE FIBER	–	–	–	–	–	–	–	–	–	–	–	–

Appendix 10

Summary Incidence and Average Severity Report

Study ID: 02776-0018: CRL 2954-001 / U of Tex Southwestern Med UTSW.Gray-002
Study Title: A SINGLE DOSE TOXICITY STUDY OF AAV9/SURF1 ADMINISTERED BY INTRATHECAL INJECTION IN RATS

This is a summary report and only displays those tissues which have at least one finding present for this study.

Tissue / *Site* / Diagnosis	Sacrifice: DAY 08		DAY 08		DAY 08		DAY 08		DAY 08		DAY 08	
Group:	1MD08		2MD08		3MD08		4MD08		1FD08		2FD08	
Dose:	0 VG		0.28E12 VG		0.83E12 VG		2.49E12 VG		0 VG		0.28E12 VG	
	INC	SEV	INC	SEV	INC	SEV	INC	SEV	INC	SEV	INC	SEV
**BRAIN, PONS**	5		5		6		5		5		5	
DEGENERATION, NERVE FIBER	–	–	–	–	–	–	–	–	–	–	–	–
**BRAIN, MEDULLA OBLONGATA**	5		5		6		5		5		5	
WHITE MATTER												
DEGENERATION, NERVE FIBER	–	–	–	–	–	–	–	–	–	–	1	0.20
**SPINAL CORD, CERVICAL**	5		5		6		5		5		5	
INFILTRATE, MONONUCLEAR CELLS	–	–	–	–	–	–	–	–	–	–	–	–
GLIAL CELL												
INCREASED CELLULARITY	–	–	–	–	–	–	–	–	–	–	–	–
GRAY MATTER												
INFILTRATE, MONONUCLEAR CELLS	–	–	–	–	–	–	–	–	–	–	–	–
MENINGES												
INFILTRATE, MONONUCLEAR CELLS	–	–	–	–	–	–	–	–	–	–	–	–
WHITE MATTER												
DEGENERATION, NERVE FIBER	–	–	–	–	–	–	–	–	–	–	–	–
**SPINAL CORD, THORACIC**	5		5		6		5		5		5	
GLIAL CELL												
INCREASED CELLULARITY	–	–	–	–	–	–	–	–	–	–	–	–
GRAY MATTER												
DEGENERATION/NECROSIS	–	–	–	–	–	–	–	–	–	–	–	–
INFILTRATE, MONONUCLEAR CELLS	–	–	–	–	–	–	–	–	–	–	–	–
INFLAMMATION	–	–	–	–	–	–	–	–	–	–	–	–
MENINGES												
INFILTRATE, MONONUCLEAR CELLS	–	–	–	–	–	–	**1**	**0.20**	–	–	–	–
NERVE ROOT, SPINAL												

Appendix 10

Summary Incidence and Average Severity Report

Study ID: 02776-0018: CRL 2954-001 / U of Tex Southwestern Med UTSW.Gray-002
Study Title: A SINGLE DOSE TOXICITY STUDY OF AAV9/SURF1 ADMINISTERED BY INTRATHECAL INJECTION IN RATS

This is a summary report and only displays those tissues which have at least one finding present for this study.

Tissue / *Site* / Diagnosis	Sacrifice: DAY 08		DAY 08		DAY 08		DAY 08		DAY 08		DAY 08	
Group:	1MD08		2MD08		3MD08		4MD08		1FD08		2FD08	
Dose:	0 VG		0.28E12 VG		0.83E12 VG		2.49E12 VG		0 VG		0.28E12 VG	
	INC	SEV	INC	SEV	INC	SEV	INC	SEV	INC	SEV	INC	SEV
DEGENERATION, NERVE FIBER	–	–	–	–	–	–	–	–	–	–	–	–
WHITE MATTER												
DEGENERATION, NERVE FIBER	–	–	–	–	**1**	**0.17**	–	–	–	–	–	–
**SPINAL CORD, LUMBAR**	5		5		6		5		5		5	
INFILTRATE, MONONUCLEAR CELLS	–	–	–	–	–	–	–	–	–	–	–	–
PIGMENT, HEMOSIDERIN	–	–	–	–	–	–	–	–	–	–	–	–
GLIAL CELL												
INCREASED CELLULARITY	–	–	–	–	–	–	–	–	–	–	–	–
GRAY MATTER												
DEGENERATION/NECROSIS	–	–	–	–	–	–	–	–	–	–	–	–
INFILTRATE, MONONUCLEAR CELLS	–	–	–	–	–	–	–	–	–	–	–	–
MENINGES												
INFILTRATE, MONONUCLEAR CELLS	–	–	–	–	–	–	–	–	–	–	–	–
MINERALIZATION	–	–	–	–	–	–	–	–	–	–	–	–
NERVE ROOT, SPINAL												
DEGENERATION, NERVE FIBER	1	0.20	–	–	**1**	**0.17**	**1**	**0.20**	–	–	–	–
WHITE MATTER												
DEGENERATION, NERVE FIBER	–	–	**1**	**0.20**	–	–	–	–	1	0.20	–	–
**NERVE, SCIATIC**	5		5		6		5		5		5	
DEGENERATION, NERVE FIBER	3	0.80	**3**	**0.60**	**5**	**0.83**	**1**	**0.20**	2	0.40	**2**	**0.40**
INFILTRATE, MIXED	–	–	–	–	–	–	–	–	–	–	–	–
SCHWANN CELL												
HYPERTROPHY/HYPERPLASIA	–	–	–	–	–	–	–	–	–	–	–	–
**NERVE, TIBIAL**	5		5		5		5		5		5	

Appendix 10

Summary Incidence and Average Severity Report

Study ID: 02776-0018: CRL 2954-001 / U of Tex Southwestern Med UTSW.Gray-002
Study Title: A SINGLE DOSE TOXICITY STUDY OF AAV9/SURF1 ADMINISTERED BY INTRATHECAL INJECTION IN RATS

This is a summary report and only displays those tissues which have at least one finding present for this study.

Tissue / *Site* / Diagnosis	DAY 08		DAY 08		DAY 08		DAY 08		DAY 08		DAY 08	
Group:	1MD08		2MD08		3MD08		4MD08		1FD08		2FD08	
Dose:	0 VG		0.28E12 VG		0.83E12 VG		2.49E12 VG		0 VG		0.28E12 VG	
	INC	SEV	INC	SEV	INC	SEV	INC	SEV	INC	SEV	INC	SEV
DEGENERATION, NERVE FIBER	–	–	**1**	**0.20**	–	–	–	–	1	0.20	**2**	**0.40**
SCHWANN CELL												
HYPERTROPHY/HYPERPLASIA	–	–	–	–	–	–	–	–	–	–	–	–
**EYE**	5		5		6		5		5		5	
RETINAL ROSETTE	–	–	–	–	–	–	–	–	2	0.40	1	0.20
**THYMUS**	5		5		6		4		5		5	
INCREASED CELLULARITY	–	–	–	–	–	–	–	–	–	–	–	–
**PANCREAS**	5		5		6		5		5		5	
APOPTOSIS/SINGLE CELL NECROSIS	–	–	–	–	–	–	1	0.20	–	–	–	–
ATROPHY	–	–	–	–	–	–	–	–	–	–	–	–
FIBROSIS	–	–	–	–	–	–	–	–	–	–	–	–
INFILTRATE, MONONUCLEAR CELLS	–	–	–	–	–	–	–	–	–	–	–	–
MITOTIC FIGURES, INCREASED	–	–	–	–	–	–	1	0.20	–	–	–	–
**LUNG**	5		5		6		5		5		5	
INFLAMMATION	–	–	–	–	1	0.17	–	–	–	–	1	0.20
ALVEOLUS												
INFILTRATE, MACROPHAGES	–	–	–	–	–	–	–	–	1	0.20	1	0.20
PERIVASCULAR												
INFILTRATE, EOSINOPHILS	–	–	–	–	–	–	–	–	–	–	–	–
**MUSCLE, BICEPS FEMORIS**	5		5		6		5		5		5	
DEGENERATION/NECROSIS	–	–	–	–	–	–	1	0.20	–	–	–	–
**HEART**	5		5		6		5		5		5	

Appendix 10

Summary Incidence and Average Severity Report

Study ID: 02776-0018: CRL 2954-001 / U of Tex Southwestern Med UTSW.Gray-002
Study Title: A SINGLE DOSE TOXICITY STUDY OF AAV9/SURF1 ADMINISTERED BY INTRATHECAL INJECTION IN RATS

This is a summary report and only displays those tissues which have at least one finding present for this study.

Tissue / *Site* / Diagnosis	Sacrifice: DAY 08		DAY 08		DAY 08		DAY 08		DAY 08		DAY 08	
Group:	1MD08		2MD08		3MD08		4MD08		1FD08		2FD08	
Dose:	0 VG		0.28E12 VG		0.83E12 VG		2.49E12 VG		0 VG		0.28E12 VG	
	INC	SEV	INC	SEV	INC	SEV	INC	SEV	INC	SEV	INC	SEV
MYOCARDIUM												
DEGENERATION/NECROSIS	–	–	5	1.00	3	0.50	–	–	2	0.40	–	–
FIBROSIS	–	–	–	–	–	–	–	–	–	–	–	–
INFILTRATE, MONONUCLEAR CELLS	–	–	5	1.00	4	0.67	1	0.20	2	0.40	–	–
**SPLEEN**	5		5		6		5		5		5	
EXTRAMEDULLARY HEMATOPOIESIS	–	–	–	–	1	0.17	–	–	–	–	–	–
**LIVER**	5		5		6		5		5		5	
EXTRAMEDULLARY HEMATOPOIESIS	3	0.60	5	1.00	3	0.50	3	0.60	3	0.60	–	–
HYPERTROPHY/HYPERPLASIA	–	–	**1**	**0.20**	**1**	**0.17**	**1**	**0.20**	–	–	–	–
INFILTRATE, MIXED	2	0.40	**5**	**1.00**	**6**	**1.00**	**5**	**1.00**	2	0.40	**4**	**0.80**
MITOTIC FIGURES, INCREASED	–	–	–	–	–	–	**1**	**0.20**	–	–	–	–
NECROSIS	–	–	–	–	–	–	–	–	–	–	–	–
HEPATOCYTE												
NECROSIS, SINGLE CELL	–	–	–	–	**1**	**0.17**	**2**	**0.60**	–	–	–	–
VACUOLATION	–	–	–	–	–	–	–	–	–	–	–	–
PERIVASCULAR												
INFILTRATE, MONONUCLEAR CELLS	–	–	–	–	–	–	–	–	–	–	–	–
**KIDNEY**	5		5		6		5		5		5	
CHRONIC PROGRESSIVE NEPHROPATHY	3	0.60	1	0.20	2	0.33	2	0.40	1	0.20	–	–
INFILTRATE, MONONUCLEAR CELLS	–	–	–	–	1	0.17	–	–	–	–	–	–
TUBULAR												
ACCUMULATION, HYALINE DROPLETS	–	–	–	–	–	–	–	–	–	–	–	–
BASOPHILIA	1	0.20	–	–	2	0.33	2	0.40	1	0.20	–	–
CYST	1	0.20	1	0.20	1	0.17	–	–	–	–	1	0.20

Appendix 10

Summary Incidence and Average Severity Report

Study ID: 02776-0018: CRL 2954-001 / U of Tex Southwestern Med UTSW.Gray-002
Study Title: A SINGLE DOSE TOXICITY STUDY OF AAV9/SURF1 ADMINISTERED BY INTRATHECAL INJECTION IN RATS

This is a summary report and only displays those tissues which have at least one finding present for this study.

Tissue / *Site* / Diagnosis	Sacrifice: DAY 08		DAY 08		DAY 08		DAY 08		DAY 08		DAY 08	
Group:	1MD08		2MD08		3MD08		4MD08		1FD08		2FD08	
Dose:	0 VG		0.28E12 VG		0.83E12 VG		2.49E12 VG		0 VG		0.28E12 VG	
	INC	SEV	INC	SEV	INC	SEV	INC	SEV	INC	SEV	INC	SEV
DILATATION	-	-	-	-	-	-	-	-	-	-	-	-
**LYMPH NODE, MESENTERIC**	5		5		6		5		5		5	
PIGMENT	-	-	-	-	-	-	-	-	-	-	-	-
**TESTIS**	5		5		6		5		-		-	
TUBULAR												
DEGENERATION	-	-	-	-	-	-	-	-	-	-	-	-
**LYMPH NODE, ILIAC**	5		5		6		5		5		5	
INCREASED CELLULARITY	-	-	-	-	-	-	-	-	-	-	-	-

Appendix 10

Summary Incidence and Average Severity Report

Study ID: 02776-0018: CRL 2954-001 / U of Tex Southwestern Med UTSW.Gray-002
Study Title: A SINGLE DOSE TOXICITY STUDY OF AAV9/SURF1 ADMINISTERED BY INTRATHECAL INJECTION IN RATS

This is a summary report and only displays those tissues which have at least one finding present for this study.

Tissue / *Site* / Diagnosis	DAY 08		DAY 08		DAY 29		DAY 29		DAY 29		DAY 29	
Group:	3FD08		4FD08		1MD29		2MD29		3MD29		4MD29	
Dose:	0.83E12 VG		2.49E12 VG		0 VG		0.28E12 VG		0.83E12 VG		2.49E12 VG	
	INC	SEV	INC	SEV	INC	SEV	INC	SEV	INC	SEV	INC	SEV
**NERVE ROOT, SPINAL, CERVICAL**	5		5		5		5		5		5	
EPINEURIUM												
INFILTRATE, MONONUCLEAR CELLS	–	–	–	–	–	–	–	–	–	–	–	–
NERVE ROOT, VENTRAL												
DEGENERATION, NERVE FIBER	–	–	–	–	–	–	–	–	–	–	–	–
**GANGLION, DORSAL ROOT, CERVICAL**	5		5		5		5		5		5	
INFILTRATE, MONONUCLEAR CELLS	–	–	–	–	–	–	–	–	1	0.20	–	–
GLIAL CELL												
HYPERTROPHY/HYPERPLASIA	–	–	2	0.40	3	0.60	–	–	4	0.80	3	0.60
**NERVE ROOT, SPINAL, THORACIC**	5		5		5		5		4		5	
EPINEURIUM												
INFILTRATE, MONONUCLEAR CELLS	–	–	–	–	–	–	–	–	–	–	–	–
NERVE ROOT, DORSAL												
DEGENERATION, NERVE FIBER	–	–	–	–	–	–	–	–	–	–	1	0.20
NERVE ROOT, VENTRAL												
DEGENERATION, NERVE FIBER	–	–	–	–	–	–	–	–	–	–	–	–
SCHWANN CELL												
HYPERTROPHY/HYPERPLASIA	–	–	–	–	–	–	–	–	–	–	–	–
**GANGLION, DORSAL ROOT, THORACIC**	5		5		5		5		4		5	
INFILTRATE, MONONUCLEAR CELLS	–	–	–	–	–	–	–	–	–	–	–	–
GLIAL CELL												
HYPERTROPHY/HYPERPLASIA	–	–	1	0.20	–	–	–	–	1	0.25	3	0.60
**NERVE ROOT, SPINAL, LUMBAR**	5		5		5		5		5		5	
EPINEURIUM												
INFILTRATE, MONONUCLEAR CELLS	3	0.60	3	0.60	2	0.40	4	0.80	3	0.60	3	0.60

Appendix 10

Summary Incidence and Average Severity Report

Study ID: 02776-0018: CRL 2954-001 / U of Tex Southwestern Med UTSW.Gray-002
Study Title: A SINGLE DOSE TOXICITY STUDY OF AAV9/SURF1 ADMINISTERED BY INTRATHECAL INJECTION IN RATS

This is a summary report and only displays those tissues which have at least one finding present for this study.

Sacrifice:	DAY 08		DAY 08		DAY 29		DAY 29		DAY 29		DAY 29	
Group:	3FD08		4FD08		1MD29		2MD29		3MD29		4MD29	
Dose:	0.83E12 VG		2.49E12 VG		0 VG		0.28E12 VG		0.83E12 VG		2.49E12 VG	
**Tissue** / *Site* / Diagnosis	INC	SEV	INC	SEV	INC	SEV	INC	SEV	INC	SEV	INC	SEV
NERVE ROOT, DORSAL												
DEGENERATION, NERVE FIBER	–	–	–	–	1	0.20	2	0.40	2	0.40	3	0.80
NERVE ROOT, VENTRAL												
DEGENERATION, NERVE FIBER	–	–	–	–	–	–	–	–	2	0.40	–	–
INFILTRATE, MONONUCLEAR CELLS	–	–	–	–	–	–	–	–	–	–	–	–
SCHWANN CELL												
HYPERTROPHY/HYPERPLASIA	–	–	–	–	–	–	–	–	–	–	–	–
**GANGLION, DORSAL ROOT, LUMBAR**	5		5		5		5		5		5	
INFILTRATE, MONONUCLEAR CELLS	1	0.20	–	–	–	–	**2**	**0.40**	**4**	**0.80**	**2**	**0.60**
GLIAL CELL												
HYPERTROPHY/HYPERPLASIA	–	–	3	0.60	3	0.60	2	0.40	5	1.00	5	1.20
NEURON												
DEGENERATION/NECROSIS	–	–	–	–	–	–	**1**	**0.20**	–	–	**1**	**0.20**
**BRAIN, MENINGES**	5		5		5		5		5		5	
INFILTRATE, MONONUCLEAR CELLS	–	–	–	–	–	–	**2**	**0.40**	–	–	–	–
**BRAIN, VENTRICULAR SYSTEM**	5		5		5		5		5		5	
INFILTRATE, MONONUCLEAR CELLS	–	–	–	–	–	–	–	–	–	–	–	–
**BRAIN, MIDBRAIN**	5		5		5		5		5		5	
PINEAL GLAND												
INFILTRATE, MONONUCLEAR CELLS	–	–	–	–	–	–	1	0.20	1	0.20	–	–
VACUOLATION	–	–	–	–	–	–	–	–	–	–	–	–
**BRAIN, CEREBELLUM**	5		5		5		5		5		5	
WHITE MATTER												
DEGENERATION, NERVE FIBER	–	–	–	–	–	–	–	–	–	–	–	–

Appendix 10

Summary Incidence and Average Severity Report

Study ID: 02776-0018: CRL 2954-001 / U of Tex Southwestern Med UTSW.Gray-002
Study Title: A SINGLE DOSE TOXICITY STUDY OF AAV9/SURF1 ADMINISTERED BY INTRATHECAL INJECTION IN RATS

This is a summary report and only displays those tissues which have at least one finding present for this study.

Tissue / Site / Diagnosis	Sacrifice: DAY 08		DAY 08		DAY 29		DAY 29		DAY 29		DAY 29	
Group:	3FD08		4FD08		1MD29		2MD29		3MD29		4MD29	
Dose:	0.83E12 VG		2.49E12 VG		0 VG		0.28E12 VG		0.83E12 VG		2.49E12 VG	
	INC	SEV	INC	SEV	INC	SEV	INC	SEV	INC	SEV	INC	SEV
**BRAIN, PONS**	5		5		5		5		5		5	
DEGENERATION, NERVE FIBER	–	–	–	–	–	–	–	–	–	–	–	–
**BRAIN, MEDULLA OBLONGATA**	5		4		5		5		5		5	
WHITE MATTER												
DEGENERATION, NERVE FIBER	–	–	–	–	1	0.20	–	–	–	–	–	–
**SPINAL CORD, CERVICAL**	5		5		5		5		5		5	
INFILTRATE, MONONUCLEAR CELLS	–	–	–	–	–	–	–	–	–	–	–	–
GLIAL CELL												
INCREASED CELLULARITY	–	–	–	–	–	–	**1**	**0.20**	–	–	–	–
GRAY MATTER												
INFILTRATE, MONONUCLEAR CELLS	–	–	–	–	–	–	–	–	–	–	–	–
MENINGES												
INFILTRATE, MONONUCLEAR CELLS	–	–	–	–	–	–	–	–	–	–	–	–
WHITE MATTER												
DEGENERATION, NERVE FIBER	–	–	–	–	1	0.20	**1**	**0.20**	–	–	–	–
**SPINAL CORD, THORACIC**	5		5		5		5		5		5	
GLIAL CELL												
INCREASED CELLULARITY	**1**	**0.40**	–	–	–	–	–	–	–	–	–	–
GRAY MATTER												
DEGENERATION/NECROSIS	**1**	**0.40**	–	–	–	–	–	–	–	–	–	–
INFILTRATE, MONONUCLEAR CELLS	**1**	**0.20**	–	–	–	–	–	–	–	–	–	–
INFLAMMATION	–	–	–	–	–	–	–	–	1	0.20	–	–
MENINGES												
INFILTRATE, MONONUCLEAR CELLS	–	–	–	–	–	–	–	–	1	0.20	–	–
NERVE ROOT, SPINAL												

Appendix 10

Summary Incidence and Average Severity Report

Study ID: 02776-0018: CRL 2954-001 / U of Tex Southwestern Med UTSW.Gray-002
Study Title: A SINGLE DOSE TOXICITY STUDY OF AAV9/SURF1 ADMINISTERED BY INTRATHECAL INJECTION IN RATS

This is a summary report and only displays those tissues which have at least one finding present for this study.

Tissue / *Site* / Diagnosis	DAY 08 / 3FD08 / 0.83E12 VG INC	SEV	DAY 08 / 4FD08 / 2.49E12 VG INC	SEV	DAY 29 / 1MD29 / 0 VG INC	SEV	DAY 29 / 2MD29 / 0.28E12 VG INC	SEV	DAY 29 / 3MD29 / 0.83E12 VG INC	SEV	DAY 29 / 4MD29 / 2.49E12 VG INC	SEV
DEGENERATION, NERVE FIBER	–	–	–	–	–	–	–	–	–	–	–	–
WHITE MATTER												
DEGENERATION, NERVE FIBER	2	0.40	–	–	–	–	1	0.20	3	0.80	2	0.40
**SPINAL CORD, LUMBAR**	5		5		5		5		5		5	
INFILTRATE, MONONUCLEAR CELLS	–	–	–	–	–	–	–	–	–	–	–	–
PIGMENT, HEMOSIDERIN	–	–	–	–	–	–	–	–	–	–	–	–
GLIAL CELL												
INCREASED CELLULARITY	–	–	–	–	–	–	–	–	–	–	–	–
GRAY MATTER												
DEGENERATION/NECROSIS	–	–	–	–	–	–	–	–	–	–	–	–
INFILTRATE, MONONUCLEAR CELLS	–	–	–	–	–	–	–	–	–	–	–	–
MENINGES												
INFILTRATE, MONONUCLEAR CELLS	–	–	–	–	–	–	–	–	–	–	–	–
MINERALIZATION	–	–	–	–	–	–	–	–	–	–	–	–
NERVE ROOT, SPINAL												
DEGENERATION, NERVE FIBER	1	0.20	–	–	–	–	1	0.20	1	0.40	2	0.40
WHITE MATTER												
DEGENERATION, NERVE FIBER	–	–	–	–	1	0.20	–	–	3	0.80	2	0.40
**NERVE, SCIATIC**	5		5		5		5		5		5	
DEGENERATION, NERVE FIBER	1	0.20	–	–	–	–	4	0.80	4	1.40	2	1.00
INFILTRATE, MIXED	–	–	–	–	–	–	–	–	–	–	–	–
SCHWANN CELL												
HYPERTROPHY/HYPERPLASIA	–	–	–	–	–	–	–	–	–	–	1	0.40
**NERVE, TIBIAL**	5		5		5		5		5		5	

Summary Incidence and Average Severity Report

Study ID: 02776-0018: CRL 2954-001 / U of Tex Southwestern Med UTSW.Gray-002
Study Title: A SINGLE DOSE TOXICITY STUDY OF AAV9/SURF1 ADMINISTERED BY INTRATHECAL INJECTION IN RATS

This is a summary report and only displays those tissues which have at least one finding present for this study.

Tissue / Site / Diagnosis	Sacrifice: DAY 08		DAY 08		DAY 29		DAY 29		DAY 29		DAY 29	
	Group: 3FD08		4FD08		1MD29		2MD29		3MD29		4MD29	
	Dose: 0.83E12 VG		2.49E12 VG		0 VG		0.28E12 VG		0.83E12 VG		2.49E12 VG	
	INC	SEV	INC	SEV	INC	SEV	INC	SEV	INC	SEV	INC	SEV
DEGENERATION, NERVE FIBER	**2**	**0.40**	–	–	–	–	**2**	**0.60**	**2**	**1.20**	**2**	**1.00**
SCHWANN CELL												
HYPERTROPHY/HYPERPLASIA	–	–	–	–	–	–	–	–	**2**	**0.40**	**2**	**0.60**
**EYE**	5		5		5		5		5		5	
RETINAL ROSETTE	–	–	–	–	–	–	–	–	–	–	–	–
**THYMUS**	5		5		5		5		5		5	
INCREASED CELLULARITY	–	–	–	–	–	–	–	–	–	–	–	–
**PANCREAS**	5		5		5		5		5		5	
APOPTOSIS/SINGLE CELL NECROSIS	–	–	–	–	–	–	–	–	–	–	1	0.20
ATROPHY	–	–	–	–	–	–	–	–	1	0.20	–	–
FIBROSIS	–	–	–	–	–	–	1	0.20	–	–	–	–
INFILTRATE, MONONUCLEAR CELLS	–	–	–	–	–	–	–	–	–	–	–	–
MITOTIC FIGURES, INCREASED	–	–	–	–	–	–	–	–	–	–	–	–
**LUNG**	5		5		5		5		5		5	
INFLAMMATION	–	–	–	–	–	–	–	–	1	0.20	1	0.20
ALVEOLUS												
INFILTRATE, MACROPHAGES	–	–	–	–	–	–	–	–	1	0.20	–	–
PERIVASCULAR												
INFILTRATE, EOSINOPHILS	–	–	–	–	–	–	1	0.20	–	–	–	–
**MUSCLE, BICEPS FEMORIS**	5		5		5		5		5		5	
DEGENERATION/NECROSIS	–	–	–	–	–	–	–	–	–	–	–	–
**HEART**	5		5		5		5		5		5	

Appendix 10

Summary Incidence and Average Severity Report

Study ID: 02776-0018: CRL 2954-001 / U of Tex Southwestern Med UTSW.Gray-002
Study Title: A SINGLE DOSE TOXICITY STUDY OF AAV9/SURF1 ADMINISTERED BY INTRATHECAL INJECTION IN RATS

This is a summary report and only displays those tissues which have at least one finding present for this study.

Tissue / *Site* / Diagnosis	DAY 08		DAY 08		DAY 29		DAY 29		DAY 29		DAY 29	
Group:	3FD08		4FD08		1MD29		2MD29		3MD29		4MD29	
Dose:	0.83E12 VG		2.49E12 VG		0 VG		0.28E12 VG		0.83E12 VG		2.49E12 VG	
	INC	SEV	INC	SEV	INC	SEV	INC	SEV	INC	SEV	INC	SEV
MYOCARDIUM												
DEGENERATION/NECROSIS	1	0.20	2	0.40	1	0.20	**5**	**1.80**	**5**	**2.40**	**5**	**1.20**
FIBROSIS	–	–	–	–	–	–	**2**	**0.60**	**2**	**0.40**	–	–
INFILTRATE, MONONUCLEAR CELLS	1	0.20	3	0.60	1	0.20	**5**	**1.80**	**5**	**2.40**	**5**	**1.20**
**SPLEEN**	5		5		5		5		5		5	
EXTRAMEDULLARY HEMATOPOIESIS	–	–	–	–	–	–	–	–	–	–	–	–
**LIVER**	5		5		5		5		5		5	
EXTRAMEDULLARY HEMATOPOIESIS	2	0.40	–	–	–	–	–	–	–	–	–	–
HYPERTROPHY/HYPERPLASIA	–	–	1	0.20	–	–	–	–	–	–	**2**	**0.60**
INFILTRATE, MIXED	**5**	**1.00**	**5**	**1.00**	4	0.80	**5**	**1.00**	**5**	**1.20**	**5**	**1.60**
MITOTIC FIGURES, INCREASED	–	–	–	–	–	–	–	–	–	–	–	–
NECROSIS	–	–	–	–	–	–	–	–	–	–	–	–
HEPATOCYTE												
NECROSIS, SINGLE CELL	–	–	–	–	–	–	–	–	–	–	**3**	**0.80**
VACUOLATION	–	–	1	0.20	–	–	1	0.40	–	–	–	–
PERIVASCULAR												
INFILTRATE, MONONUCLEAR CELLS	–	–	–	–	–	–	1	0.20	–	–	–	–
**KIDNEY**	5		5		5		5		5		5	
CHRONIC PROGRESSIVE NEPHROPATHY	1	0.20	1	0.20	4	0.80	2	0.40	2	0.40	2	0.40
INFILTRATE, MONONUCLEAR CELLS	–	–	–	–	–	–	–	–	–	–	–	–
TUBULAR												
ACCUMULATION, HYALINE DROPLETS	–	–	–	–	–	–	–	–	–	–	–	–
BASOPHILIA	1	0.20	–	–	–	–	1	0.20	–	–	1	0.20
CYST	–	–	–	–	1	0.20	–	–	1	0.20	1	0.20

Appendix 10

Summary Incidence and Average Severity Report

Study ID: 02776-0018: CRL 2954-001 / U of Tex Southwestern Med UTSW.Gray-002
Study Title: A SINGLE DOSE TOXICITY STUDY OF AAV9/SURF1 ADMINISTERED BY INTRATHECAL INJECTION IN RATS

This is a summary report and only displays those tissues which have at least one finding present for this study.

Tissue / *Site* / Diagnosis	DAY 08		DAY 08		DAY 29		DAY 29		DAY 29		DAY 29	
Group:	3FD08		4FD08		1MD29		2MD29		3MD29		4MD29	
Dose:	0.83E12 VG		2.49E12 VG		0 VG		0.28E12 VG		0.83E12 VG		2.49E12 VG	
	INC	SEV	INC	SEV	INC	SEV	INC	SEV	INC	SEV	INC	SEV
DILATATION	–	–	–	–	–	–	–	–	–	–	–	–
**LYMPH NODE, MESENTERIC**	5		5		5		5		5		5	
PIGMENT	–	–	–	–	–	–	–	–	–	–	–	–
**TESTIS**	-		-		5		5		5		5	
TUBULAR												
DEGENERATION	–	–	–	–	1	0.20	–	–	–	–	–	–
**LYMPH NODE, ILIAC**	5		4		4		5		5		5	
INCREASED CELLULARITY	–	–	–	–	–	–	–	–	–	–	–	–

Appendix 10

Summary Incidence and Average Severity Report

Study ID: 02776-0018: CRL 2954-001 / U of Tex Southwestern Med UTSW.Gray-002
Study Title: A SINGLE DOSE TOXICITY STUDY OF AAV9/SURF1 ADMINISTERED BY INTRATHECAL INJECTION IN RATS

This is a summary report and only displays those tissues which have at least one finding present for this study.

Tissue / *Site* / Diagnosis	Sacrifice: DAY 29		DAY 29		DAY 29		DAY 29		DAY 91		DAY 91	
Group:	1FD29		2FD29		3FD29		4FD29		1MD91		2MD91	
Dose:	0 VG		0.28E12 VG		0.83E12 VG		2.49E12 VG		0 VG		0.28E12 VG	
	INC	SEV	INC	SEV	INC	SEV	INC	SEV	INC	SEV	INC	SEV
**NERVE ROOT, SPINAL, CERVICAL**	5		5		5		5		5		5	
EPINEURIUM												
INFILTRATE, MONONUCLEAR CELLS	1	0.20	–	–	–	–	–	–	–	–	–	–
NERVE ROOT, VENTRAL												
DEGENERATION, NERVE FIBER	–	–	–	–	–	–	–	–	–	–	–	–
**GANGLION, DORSAL ROOT, CERVICAL**	5		5		5		5		5		5	
INFILTRATE, MONONUCLEAR CELLS	–	–	–	–	–	–	–	–	–	–	1	0.20
GLIAL CELL												
HYPERTROPHY/HYPERPLASIA	1	0.20	–	–	2	0.40	5	1.00	–	–	2	0.40
**NERVE ROOT, SPINAL, THORACIC**	5		5		5		5		5		5	
EPINEURIUM												
INFILTRATE, MONONUCLEAR CELLS	–	–	–	–	–	–	–	–	–	–	–	–
NERVE ROOT, DORSAL												
DEGENERATION, NERVE FIBER	–	–	–	–	–	–	–	–	–	–	–	–
NERVE ROOT, VENTRAL												
DEGENERATION, NERVE FIBER	1	0.20	–	–	–	–	–	–	–	–	–	–
SCHWANN CELL												
HYPERTROPHY/HYPERPLASIA	–	–	–	–	–	–	–	–	–	–	–	–
**GANGLION, DORSAL ROOT, THORACIC**	5		5		5		5		5		5	
INFILTRATE, MONONUCLEAR CELLS	–	–	–	–	–	–	–	–	1	0.20	–	–
GLIAL CELL												
HYPERTROPHY/HYPERPLASIA	1	0.20	–	–	1	0.20	1	0.20	–	–	1	0.20
**NERVE ROOT, SPINAL, LUMBAR**	5		5		5		5		5		5	
EPINEURIUM												
INFILTRATE, MONONUCLEAR CELLS	2	0.40	–	–	1	0.20	2	0.40	–	–	1	0.20

Appendix 10

Summary Incidence and Average Severity Report

Study ID: 02776-0018: CRL 2954-001 / U of Tex Southwestern Med UTSW.Gray-002
Study Title: A SINGLE DOSE TOXICITY STUDY OF AAV9/SURF1 ADMINISTERED BY INTRATHECAL INJECTION IN RATS

This is a summary report and only displays those tissues which have at least one finding present for this study.

Tissue / *Site* / Diagnosis	Sacrifice: DAY 29		DAY 29		DAY 29		DAY 29		DAY 91		DAY 91	
Group:	1FD29		2FD29		3FD29		4FD29		1MD91		2MD91	
Dose:	0 VG		0.28E12 VG		0.83E12 VG		2.49E12 VG		0 VG		0.28E12 VG	
	INC	SEV	INC	SEV	INC	SEV	INC	SEV	INC	SEV	INC	SEV
NERVE ROOT, DORSAL												
DEGENERATION, NERVE FIBER	–	–	–	–	3	1.00	2	0.80	–	–	2	0.60
NERVE ROOT, VENTRAL												
DEGENERATION, NERVE FIBER	–	–	2	0.40	2	0.80	–	–	–	–	–	–
INFILTRATE, MONONUCLEAR CELLS	–	–	–	–	–	–	–	–	–	–	–	–
SCHWANN CELL												
HYPERTROPHY/HYPERPLASIA	–	–	–	–	–	–	–	–	–	–	–	–
**GANGLION, DORSAL ROOT, LUMBAR**	5		5		5		5		5		5	
INFILTRATE, MONONUCLEAR CELLS	1	0.20	–	–	**4**	**0.80**	**1**	**0.20**	–	–	**1**	**0.40**
GLIAL CELL												
HYPERTROPHY/HYPERPLASIA	–	–	–	–	**4**	**0.80**	5	1.00	3	0.60	3	0.60
NEURON												
DEGENERATION/NECROSIS	–	–	–	–	–	–	–	–	–	–	–	–
**BRAIN, MENINGES**	5		5		5		5		5		5	
INFILTRATE, MONONUCLEAR CELLS	–	–	–	–	**1**	**0.20**	**1**	**0.20**	–	–	–	–
**BRAIN, VENTRICULAR SYSTEM**	5		5		5		5		5		5	
INFILTRATE, MONONUCLEAR CELLS	–	–	–	–	–	–	–	–	–	–	–	–
**BRAIN, MIDBRAIN**	5		5		5		5		5		5	
PINEAL GLAND												
INFILTRATE, MONONUCLEAR CELLS	–	–	–	–	–	–	1	0.40	–	–	–	–
VACUOLATION	–	–	–	–	–	–	–	–	–	–	1	0.60
**BRAIN, CEREBELLUM**	5		5		5		5		5		5	
WHITE MATTER												
DEGENERATION, NERVE FIBER	–	–	–	–	1	0.40	–	–	–	–	–	–

Appendix 10

Summary Incidence and Average Severity Report

Study ID: 02776-0018: CRL 2954-001 / U of Tex Southwestern Med UTSW.Gray-002
Study Title: A SINGLE DOSE TOXICITY STUDY OF AAV9/SURF1 ADMINISTERED BY INTRATHECAL INJECTION IN RATS

This is a summary report and only displays those tissues which have at least one finding present for this study.

Tissue / *Site* / Diagnosis	Sacrifice: DAY 29		DAY 29		DAY 29		DAY 29		DAY 91		DAY 91	
	Group: 1FD29		2FD29		3FD29		4FD29		1MD91		2MD91	
	Dose: 0 VG		0.28E12 VG		0.83E12 VG		2.49E12 VG		0 VG		0.28E12 VG	
	INC	SEV	INC	SEV	INC	SEV	INC	SEV	INC	SEV	INC	SEV
**BRAIN, PONS**	5		5		5		5		5		5	
DEGENERATION, NERVE FIBER	–	–	–	–	2	0.40	–	–	–	–	–	–
**BRAIN, MEDULLA OBLONGATA**	5		5		5		5		5		5	
WHITE MATTER												
DEGENERATION, NERVE FIBER	–	–	–	–	1	0.40	–	–	1	0.20	2	0.40
**SPINAL CORD, CERVICAL**	5		5		5		5		5		5	
INFILTRATE, MONONUCLEAR CELLS	–	–	–	–	**1**	**0.20**	–	–	–	–	–	–
GLIAL CELL												
INCREASED CELLULARITY	–	–	–	–	**1**	**0.20**	–	–	–	–	–	–
GRAY MATTER												
INFILTRATE, MONONUCLEAR CELLS	–	–	–	–	–	–	–	–	–	–	–	–
MENINGES												
INFILTRATE, MONONUCLEAR CELLS	–	–	–	–	–	–	**1**	**0.20**	–	–	–	–
WHITE MATTER												
DEGENERATION, NERVE FIBER	–	–	–	–	**2**	**0.40**	**1**	**0.20**	–	–	**2**	**0.40**
**SPINAL CORD, THORACIC**	5		5		5		5		5		5	
GLIAL CELL												
INCREASED CELLULARITY	–	–	–	–	**1**	**0.40**	–	–	–	–	**1**	**0.20**
GRAY MATTER												
DEGENERATION/NECROSIS	–	–	–	–	**1**	**1.00**	–	–	–	–	–	–
INFILTRATE, MONONUCLEAR CELLS	–	–	–	–	**1**	**0.20**	–	–	–	–	–	–
INFLAMMATION	–	–	–	–	–	–	–	–	–	–	–	–
MENINGES												
INFILTRATE, MONONUCLEAR CELLS	–	–	–	–	–	–	–	–	–	–	**1**	**0.20**
NERVE ROOT, SPINAL												

Appendix 10

Summary Incidence and Average Severity Report

Study ID: 02776-0018: CRL 2954-001 / U of Tex Southwestern Med UTSW.Gray-002
Study Title: A SINGLE DOSE TOXICITY STUDY OF AAV9/SURF1 ADMINISTERED BY INTRATHECAL INJECTION IN RATS

This is a summary report and only displays those tissues which have at least one finding present for this study.

Tissue / *Site* / Diagnosis	Sacrifice: DAY 29		DAY 29		DAY 29		DAY 29		DAY 91		DAY 91	
Group:	1FD29		2FD29		3FD29		4FD29		1MD91		2MD91	
Dose:	0 VG		0.28E12 VG		0.83E12 VG		2.49E12 VG		0 VG		0.28E12 VG	
	INC	SEV	INC	SEV	INC	SEV	INC	SEV	INC	SEV	INC	SEV
DEGENERATION, NERVE FIBER	–	–	–	–	–	–	–	–	–	–	**1**	**0.40**
WHITE MATTER												
DEGENERATION, NERVE FIBER	–	–	**2**	**0.40**	**5**	**1.40**	**3**	**0.60**	2	0.40	**2**	**0.40**
**SPINAL CORD, LUMBAR**	5		5		5		5		5		5	
INFILTRATE, MONONUCLEAR CELLS	–	–	–	–	**1**	**0.20**	–	–	–	–	–	–
PIGMENT, HEMOSIDERIN	–	–	–	–	–	–	–	–	–	–	–	–
GLIAL CELL												
INCREASED CELLULARITY	–	–	–	–	**1**	**0.40**	–	–	–	–	–	–
GRAY MATTER												
DEGENERATION/NECROSIS	–	–	–	–	**1**	**0.60**	–	–	–	–	–	–
INFILTRATE, MONONUCLEAR CELLS	–	–	–	–	**1**	**0.20**	–	–	–	–	–	–
MENINGES												
INFILTRATE, MONONUCLEAR CELLS	–	–	–	–	–	–	–	–	–	–	–	–
MINERALIZATION	–	–	–	–	–	–	–	–	–	–	–	–
NERVE ROOT, SPINAL												
DEGENERATION, NERVE FIBER	–	–	–	–	–	–	–	–	1	0.20	–	–
WHITE MATTER												
DEGENERATION, NERVE FIBER	–	–	**3**	**0.60**	**3**	**1.00**	**3**	**0.60**	1	0.20	**1**	**0.20**
**NERVE, SCIATIC**	5		5		5		5		5		5	
DEGENERATION, NERVE FIBER	–	–	**2**	**0.60**	**5**	**1.60**	**4**	**1.60**	1	0.20	**3**	**0.60**
INFILTRATE, MIXED	–	–	–	–	**1**	**0.20**	–	–	–	–	–	–
SCHWANN CELL												
HYPERTROPHY/HYPERPLASIA	–	–	–	–	–	–	–	–	–	–	–	–
**NERVE, TIBIAL**	5		5		5		5		5		5	

Appendix 10

Summary Incidence and Average Severity Report

Study ID: 02776-0018: CRL 2954-001 / U of Tex Southwestern Med UTSW.Gray-002
Study Title: A SINGLE DOSE TOXICITY STUDY OF AAV9/SURF1 ADMINISTERED BY INTRATHECAL INJECTION IN RATS

This is a summary report and only displays those tissues which have at least one finding present for this study.

Sacrifice:	DAY 29		DAY 29		DAY 29		DAY 29		DAY 91		DAY 91	
Group:	1FD29		2FD29		3FD29		4FD29		1MD91		2MD91	
Tissue / *Site* / Diagnosis — Dose:	0 VG		0.28E12 VG		0.83E12 VG		2.49E12 VG		0 VG		0.28E12 VG	
	INC	SEV	INC	SEV	INC	SEV	INC	SEV	INC	SEV	INC	SEV
DEGENERATION, NERVE FIBER	–	–	**1**	**0.40**	**4**	**1.40**	**4**	**1.60**	–	–	**1**	**0.20**
SCHWANN CELL												
HYPERTROPHY/HYPERPLASIA	–	–	–	–	–	–	–	–	–	–	–	–
**EYE**	5		5		5		5		2		4	
RETINAL ROSETTE	–	–	–	–	–	–	–	–	–	–	–	–
**THYMUS**	5		5		4		5		5		5	
INCREASED CELLULARITY	–	–	–	–	–	–	–	–	1	0.20	–	–
**PANCREAS**	5		5		5		5		5		5	
APOPTOSIS/SINGLE CELL NECROSIS	–	–	–	–	–	–	1	0.20	–	–	–	–
ATROPHY	–	–	–	–	–	–	–	–	–	–	–	–
FIBROSIS	–	–	–	–	–	–	–	–	1	0.20	–	–
INFILTRATE, MONONUCLEAR CELLS	–	–	–	–	–	–	–	–	1	0.20	–	–
MITOTIC FIGURES, INCREASED	–	–	–	–	–	–	–	–	–	–	–	–
**LUNG**	5		5		5		5		5		5	
INFLAMMATION	1	0.20	1	0.40	–	–	1	0.20	–	–	–	–
ALVEOLUS												
INFILTRATE, MACROPHAGES	–	–	–	–	–	–	–	–	1	0.20	1	0.20
PERIVASCULAR												
INFILTRATE, EOSINOPHILS	1	0.20	–	–	–	–	–	–	–	–	–	–
**MUSCLE, BICEPS FEMORIS**	5		5		5		5		5		5	
DEGENERATION/NECROSIS	–	–	–	–	–	–	–	–	–	–	–	–
**HEART**	5		5		5		5		5		5	

Appendix 10

Summary Incidence and Average Severity Report

Study ID: 02776-0018: CRL 2954-001 / U of Tex Southwestern Med UTSW.Gray-002
Study Title: A SINGLE DOSE TOXICITY STUDY OF AAV9/SURF1 ADMINISTERED BY INTRATHECAL INJECTION IN RATS

This is a summary report and only displays those tissues which have at least one finding present for this study.

Tissue / *Site* / Diagnosis	DAY 29		DAY 29		DAY 29		DAY 29		DAY 91		DAY 91	
Group:	1FD29		2FD29		3FD29		4FD29		1MD91		2MD91	
Dose:	0 VG		0.28E12 VG		0.83E12 VG		2.49E12 VG		0 VG		0.28E12 VG	
	INC	SEV	INC	SEV	INC	SEV	INC	SEV	INC	SEV	INC	SEV
MYOCARDIUM												
DEGENERATION/NECROSIS	–	–	**5**	**2.00**	**4**	**1.60**	**4**	**1.60**	4	0.80	**5**	**1.00**
FIBROSIS	–	–	**2**	**0.80**	**1**	**0.20**	**1**	**0.20**	1	0.20	**4**	**0.80**
INFILTRATE, MONONUCLEAR CELLS	–	–	**5**	**2.00**	**4**	**1.60**	**4**	**1.60**	4	0.80	**5**	**1.20**
**SPLEEN**	5		5		5		5		5		5	
EXTRAMEDULLARY HEMATOPOIESIS	–	–	–	–	–	–	–	–	–	–	–	–
**LIVER**	5		5		5		5		5		5	
EXTRAMEDULLARY HEMATOPOIESIS	–	–	–	–	–	–	–	–	–	–	–	–
HYPERTROPHY/HYPERPLASIA	–	–	–	–	**1**	**0.20**	–	–	–	–	–	–
INFILTRATE, MIXED	3	0.60	**4**	**1.00**	**5**	**1.20**	**5**	**1.20**	3	0.60	**5**	**1.20**
MITOTIC FIGURES, INCREASED	–	–	–	–	–	–	–	–	–	–	–	–
NECROSIS	–	–	–	–	–	–	–	–	2	0.40	1	0.20
HEPATOCYTE												
NECROSIS, SINGLE CELL	–	–	–	–	**1**	**0.20**	–	–	–	–	–	–
VACUOLATION	1	0.20	–	–	–	–	–	–	1	0.20	–	–
PERIVASCULAR												
INFILTRATE, MONONUCLEAR CELLS	–	–	–	–	–	–	–	–	–	–	–	–
**KIDNEY**	5		5		5		5		5		5	
CHRONIC PROGRESSIVE NEPHROPATHY	1	0.20	–	–	–	–	2	0.40	1	0.20	2	0.40
INFILTRATE, MONONUCLEAR CELLS	–	–	–	–	–	–	–	–	–	–	–	–
TUBULAR												
ACCUMULATION, HYALINE DROPLETS	–	–	–	–	–	–	–	–	1	0.20	–	–
BASOPHILIA	1	0.20	–	–	–	–	–	–	–	–	–	–
CYST	–	–	–	–	–	–	–	–	–	–	1	0.20

Appendix 10

Summary Incidence and Average Severity Report

Study ID: 02776-0018: CRL 2954-001 / U of Tex Southwestern Med UTSW.Gray-002
Study Title: A SINGLE DOSE TOXICITY STUDY OF AAV9/SURF1 ADMINISTERED BY INTRATHECAL INJECTION IN RATS

This is a summary report and only displays those tissues which have at least one finding present for this study.

Tissue / *Site* / Diagnosis	Sacrifice: DAY 29		DAY 29		DAY 29		DAY 29		DAY 91		DAY 91	
Group:	1FD29		2FD29		3FD29		4FD29		1MD91		2MD91	
Dose:	0 VG		0.28E12 VG		0.83E12 VG		2.49E12 VG		0 VG		0.28E12 VG	
	INC	SEV	INC	SEV	INC	SEV	INC	SEV	INC	SEV	INC	SEV
DILATATION	–	–	–	–	–	–	–	–	1	0.20	1	0.20
**LYMPH NODE, MESENTERIC**	5		5		5		5		5		5	
PIGMENT	–	–	–	–	–	–	–	–	–	–	–	–
**TESTIS**	-		-		-		-		5		5	
TUBULAR												
DEGENERATION	–	–	–	–	–	–	–	–	–	–	–	–
**LYMPH NODE, ILIAC**	5		5		5		5		4		5	
INCREASED CELLULARITY	–	–	–	–	–	–	–	–	–	–	–	–

Appendix 10

Summary Incidence and Average Severity Report

Study ID: 02776-0018: CRL 2954-001 / U of Tex Southwestern Med UTSW.Gray-002
Study Title: A SINGLE DOSE TOXICITY STUDY OF AAV9/SURF1 ADMINISTERED BY INTRATHECAL INJECTION IN RATS

This is a summary report and only displays those tissues which have at least one finding present for this study.

**Sacrifice:**	**DAY 91**		**DAY 91**		**DAY 91**		**DAY 91**		**DAY 91**		**DAY 91**	
**Group:**	**3MD91**		**4MD91**		**1FD91**		**2FD91**		**3FD91**		**4FD91**	
**Dose:**	**0.83E12 VG**		**2.49E12 VG**		**0 VG**		**0.28E12 VG**		**0.83E12 VG**		**2.49E12 VG**	
**Tissue** / *Site* / Diagnosis	**INC**	**SEV**	**INC**	**SEV**	**INC**	**SEV**	**INC**	**SEV**	**INC**	**SEV**	**INC**	**SEV**
**NERVE ROOT, SPINAL, CERVICAL**	4		5		5		5		5		5	
EPINEURIUM												
INFILTRATE, MONONUCLEAR CELLS	–	–	–	–	–	–	–	–	–	–	–	–
NERVE ROOT, VENTRAL												
DEGENERATION, NERVE FIBER	–	–	–	–	–	–	–	–	–	–	–	–
**GANGLION, DORSAL ROOT, CERVICAL**	4		5		5		5		5		5	
INFILTRATE, MONONUCLEAR CELLS	–	–	–	–	–	–	1	0.20	–	–	1	0.20
GLIAL CELL												
HYPERTROPHY/HYPERPLASIA	3	0.75	2	0.40	–	–	1	0.20	3	0.60	3	0.60
**NERVE ROOT, SPINAL, THORACIC**	4		5		5		5		5		5	
EPINEURIUM												
INFILTRATE, MONONUCLEAR CELLS	–	–	–	–	–	–	–	–	–	–	–	–
NERVE ROOT, DORSAL												
DEGENERATION, NERVE FIBER	–	–	–	–	–	–	–	–	–	–	–	–
NERVE ROOT, VENTRAL												
DEGENERATION, NERVE FIBER	–	–	–	–	–	–	–	–	–	–	–	–
SCHWANN CELL												
HYPERTROPHY/HYPERPLASIA	–	–	–	–	–	–	–	–	–	–	–	–
**GANGLION, DORSAL ROOT, THORACIC**	4		5		5		5		5		5	
INFILTRATE, MONONUCLEAR CELLS	2	0.50	–	–	–	–	1	0.20	1	0.20	1	0.20
GLIAL CELL												
HYPERTROPHY/HYPERPLASIA	3	0.75	2	0.40	–	–	–	–	3	0.60	2	0.40
**NERVE ROOT, SPINAL, LUMBAR**	4		5		5		5		5		5	
EPINEURIUM												
INFILTRATE, MONONUCLEAR CELLS	–	–	1	0.20	1	0.20	–	–	2	0.40	2	0.40

Appendix 10

Summary Incidence and Average Severity Report

Study ID: 02776-0018: CRL 2954-001 / U of Tex Southwestern Med UTSW.Gray-002
Study Title: A SINGLE DOSE TOXICITY STUDY OF AAV9/SURF1 ADMINISTERED BY INTRATHECAL INJECTION IN RATS

This is a summary report and only displays those tissues which have at least one finding present for this study.

Tissue / *Site* / Diagnosis	Sacrifice: DAY 91		DAY 91		DAY 91		DAY 91		DAY 91		DAY 91	
	Group: 3MD91		4MD91		1FD91		2FD91		3FD91		4FD91	
	Dose: 0.83E12 VG		2.49E12 VG		0 VG		0.28E12 VG		0.83E12 VG		2.49E12 VG	
	INC	SEV	INC	SEV	INC	SEV	INC	SEV	INC	SEV	INC	SEV
NERVE ROOT, DORSAL												
DEGENERATION, NERVE FIBER	–	–	2	0.60	–	–	–	–	–	–	2	0.40
NERVE ROOT, VENTRAL												
DEGENERATION, NERVE FIBER	1	0.25	1	0.20	–	–	1	0.20	2	0.80	–	–
INFILTRATE, MONONUCLEAR CELLS	–	–	–	–	–	–	–	–	–	–	–	–
SCHWANN CELL												
HYPERTROPHY/HYPERPLASIA	–	–	–	–	–	–	–	–	–	–	–	–
**GANGLION, DORSAL ROOT, LUMBAR**	4		5		5		5		5		5	
INFILTRATE, MONONUCLEAR CELLS	**3**	**0.75**	**2**	**0.40**	–	–	–	–	3	0.60	**3**	**0.60**
GLIAL CELL												
HYPERTROPHY/HYPERPLASIA	3	0.75	3	0.60	–	–	4	0.80	4	0.80	4	0.80
NEURON												
DEGENERATION/NECROSIS	–	–	–	–	–	–	–	–	–	–	–	–
**BRAIN, MENINGES**	4		5		5		5		5		5	
INFILTRATE, MONONUCLEAR CELLS	–	–	–	–	–	–	–	–	**1**	**0.20**	–	–
**BRAIN, VENTRICULAR SYSTEM**	4		5		5		5		5		5	
INFILTRATE, MONONUCLEAR CELLS	–	–	–	–	–	–	–	–	–	–	–	–
**BRAIN, MIDBRAIN**	4		5		5		5		5		5	
PINEAL GLAND												
INFILTRATE, MONONUCLEAR CELLS	1	0.25	–	–	–	–	1	0.20	1	0.20	1	0.20
VACUOLATION	–	–	–	–	–	–	–	–	–	–	–	–
**BRAIN, CEREBELLUM**	4		5		5		5		5		5	
WHITE MATTER												
DEGENERATION, NERVE FIBER	–	–	–	–	–	–	–	–	–	–	–	–

Summary Incidence and Average Severity Report

Study ID: 02776-0018: CRL 2954-001 / U of Tex Southwestern Med UTSW.Gray-002
Study Title: A SINGLE DOSE TOXICITY STUDY OF AAV9/SURF1 ADMINISTERED BY INTRATHECAL INJECTION IN RATS

This is a summary report and only displays those tissues which have at least one finding present for this study.

Tissue / *Site* / Diagnosis	Sacrifice: DAY 91		DAY 91		DAY 91		DAY 91		DAY 91		DAY 91	
Group:	3MD91		4MD91		1FD91		2FD91		3FD91		4FD91	
Dose:	0.83E12 VG		2.49E12 VG		0 VG		0.28E12 VG		0.83E12 VG		2.49E12 VG	
	INC	SEV	INC	SEV	INC	SEV	INC	SEV	INC	SEV	INC	SEV
**BRAIN, PONS**	4		5		5		5		5		5	
DEGENERATION, NERVE FIBER	–	–	–	–	–	–	–	–	–	–	–	–
**BRAIN, MEDULLA OBLONGATA**	4		5		5		5		5		5	
WHITE MATTER												
DEGENERATION, NERVE FIBER	1	0.25	1	0.20	–	–	1	0.20	1	0.20	2	0.40
**SPINAL CORD, CERVICAL**	4		5		5		5		5		5	
INFILTRATE, MONONUCLEAR CELLS	–	–	–	–	–	–	–	–	–	–	–	–
GLIAL CELL												
INCREASED CELLULARITY	–	–	–	–	–	–	–	–	–	–	–	–
GRAY MATTER												
INFILTRATE, MONONUCLEAR CELLS	–	–	–	–	–	–	–	–	–	–	–	–
MENINGES												
INFILTRATE, MONONUCLEAR CELLS	–	–	–	–	–	–	–	–	–	–	–	–
WHITE MATTER												
DEGENERATION, NERVE FIBER	**2**	**0.50**	**2**	**0.40**	–	–	–	–	–	–	**2**	**0.40**
**SPINAL CORD, THORACIC**	4		5		5		5		5		5	
GLIAL CELL												
INCREASED CELLULARITY	–	–	**1**	**0.20**	–	–	–	–	–	–	–	–
GRAY MATTER												
DEGENERATION/NECROSIS	–	–	–	–	–	–	–	–	–	–	–	–
INFILTRATE, MONONUCLEAR CELLS	–	–	–	–	–	–	–	–	–	–	–	–
INFLAMMATION	–	–	–	–	–	–	–	–	–	–	–	–
MENINGES												
INFILTRATE, MONONUCLEAR CELLS	–	–	–	–	–	–	–	–	–	–	–	–
NERVE ROOT, SPINAL												

Appendix 10

Summary Incidence and Average Severity Report

Study ID: 02776-0018: CRL 2954-001 / U of Tex Southwestern Med UTSW.Gray-002
Study Title: A SINGLE DOSE TOXICITY STUDY OF AAV9/SURF1 ADMINISTERED BY INTRATHECAL INJECTION IN RATS

This is a summary report and only displays those tissues which have at least one finding present for this study.

Tissue / *Site* / Diagnosis	Sacrifice: DAY 91		DAY 91		DAY 91		DAY 91		DAY 91		DAY 91	
Group:	3MD91		4MD91		1FD91		2FD91		3FD91		4FD91	
Dose:	0.83E12 VG		2.49E12 VG		0 VG		0.28E12 VG		0.83E12 VG		2.49E12 VG	
	INC	SEV	INC	SEV	INC	SEV	INC	SEV	INC	SEV	INC	SEV
DEGENERATION, NERVE FIBER	–	–	1	0.20	–	–	–	–	–	–	–	–
WHITE MATTER												
DEGENERATION, NERVE FIBER	3	0.75	2	0.40	–	–	2	0.40	3	0.80	5	1.00
**SPINAL CORD, LUMBAR**	4		5		4		5		5		5	
INFILTRATE, MONONUCLEAR CELLS	–	–	–	–	–	–	–	–	–	–	–	–
PIGMENT, HEMOSIDERIN	–	–	–	–	–	–	1	0.20	–	–	–	–
GLIAL CELL												
INCREASED CELLULARITY	–	–	–	–	–	–	1	0.40	–	–	–	–
GRAY MATTER												
DEGENERATION/NECROSIS	–	–	–	–	–	–	–	–	–	–	–	–
INFILTRATE, MONONUCLEAR CELLS	–	–	–	–	–	–	–	–	–	–	–	–
MENINGES												
INFILTRATE, MONONUCLEAR CELLS	–	–	–	–	–	–	–	–	1	0.20	–	–
MINERALIZATION	–	–	–	–	–	–	–	–	1	0.20	–	–
NERVE ROOT, SPINAL												
DEGENERATION, NERVE FIBER	1	0.50	1	0.20	–	–	–	–	4	1.60	2	0.60
WHITE MATTER												
DEGENERATION, NERVE FIBER	2	0.50	–	–	–	–	–	–	–	–	1	0.20
**NERVE, SCIATIC**	4		5		5		5		5		5	
DEGENERATION, NERVE FIBER	4	1.25	3	0.80	–	–	2	0.40	3	0.80	4	1.20
INFILTRATE, MIXED	–	–	–	–	–	–	–	–	–	–	–	–
SCHWANN CELL												
HYPERTROPHY/HYPERPLASIA	–	–	–	–	–	–	–	–	1	0.20	–	–
**NERVE, TIBIAL**	4		5		5		5		5		5	

Appendix 10

Summary Incidence and Average Severity Report

Study ID: 02776-0018: CRL 2954-001 / U of Tex Southwestern Med UTSW.Gray-002
Study Title: A SINGLE DOSE TOXICITY STUDY OF AAV9/SURF1 ADMINISTERED BY INTRATHECAL INJECTION IN RATS

This is a summary report and only displays those tissues which have at least one finding present for this study.

Tissue / *Site* / Diagnosis	Sacrifice: DAY 91		DAY 91		DAY 91		DAY 91		DAY 91		DAY 91	
Group:	3MD91		4MD91		1FD91		2FD91		3FD91		4FD91	
Dose:	0.83E12 VG		2.49E12 VG		0 VG		0.28E12 VG		0.83E12 VG		2.49E12 VG	
	INC	SEV	INC	SEV	INC	SEV	INC	SEV	INC	SEV	INC	SEV
DEGENERATION, NERVE FIBER	**2**	**0.75**	**3**	**0.60**	–	–	**1**	**0.20**	**3**	**0.80**	**4**	**1.20**
SCHWANN CELL												
HYPERTROPHY/HYPERPLASIA	–	–	–	–	–	–	–	–	**1**	**0.40**	–	–
**EYE**	4		5		5		5		5		5	
RETINAL ROSETTE	1	1.00	1	0.40	1	0.20	–	–	–	–	–	–
**THYMUS**	4		4		5		5		5		5	
INCREASED CELLULARITY	–	–	–	–	2	0.40	2	0.40	1	0.20	–	–
**PANCREAS**	4		5		5		5		5		5	
APOPTOSIS/SINGLE CELL NECROSIS	–	–	–	–	–	–	–	–	–	–	–	–
ATROPHY	–	–	2	0.40	–	–	–	–	–	–	–	–
FIBROSIS	–	–	3	0.80	–	–	–	–	–	–	–	–
INFILTRATE, MONONUCLEAR CELLS	1	0.25	–	–	–	–	1	0.20	–	–	–	–
MITOTIC FIGURES, INCREASED	–	–	–	–	–	–	–	–	–	–	–	–
**LUNG**	4		5		5		5		5		5	
INFLAMMATION	1	0.25	1	0.20	–	–	–	–	–	–	–	–
ALVEOLUS												
INFILTRATE, MACROPHAGES	1	0.25	2	0.40	1	0.20	1	0.20	–	–	1	0.20
PERIVASCULAR												
INFILTRATE, EOSINOPHILS	–	–	–	–	–	–	–	–	–	–	–	–
**MUSCLE, BICEPS FEMORIS**	4		5		5		5		5		5	
DEGENERATION/NECROSIS	–	–	–	–	–	–	–	–	–	–	–	–
**HEART**	4		5		5		5		5		5	

Summary Incidence and Average Severity Report

Study ID: 02776-0018: CRL 2954-001 / U of Tex Southwestern Med UTSW.Gray-002
Study Title: A SINGLE DOSE TOXICITY STUDY OF AAV9/SURF1 ADMINISTERED BY INTRATHECAL INJECTION IN RATS

This is a summary report and only displays those tissues which have at least one finding present for this study.

Tissue / *Site* / Diagnosis	Sacrifice: DAY 91		DAY 91		DAY 91		DAY 91		DAY 91		DAY 91	
Group:	3MD91		4MD91		1FD91		2FD91		3FD91		4FD91	
Dose:	0.83E12 VG		2.49E12 VG		0 VG		0.28E12 VG		0.83E12 VG		2.49E12 VG	
	INC	SEV	INC	SEV	INC	SEV	INC	SEV	INC	SEV	INC	SEV
MYOCARDIUM												
DEGENERATION/NECROSIS	**4**	**1.25**	**4**	**0.80**	1	0.20	**3**	**0.60**	**4**	**0.80**	**5**	**1.00**
FIBROSIS	**1**	**0.75**	**5**	**1.60**	–	–	–	–	**4**	**1.20**	2	0.40
INFILTRATE, MONONUCLEAR CELLS	**4**	**1.75**	**4**	**1.40**	2	0.40	**3**	**0.80**	**5**	**1.80**	**5**	**1.00**
**SPLEEN**	4		5		5		5		5		5	
EXTRAMEDULLARY HEMATOPOIESIS	–	–	–	–	–	–	–	–	–	–	–	–
**LIVER**	4		5		5		5		5		5	
EXTRAMEDULLARY HEMATOPOIESIS	–	–	–	–	–	–	–	–	–	–	–	–
HYPERTROPHY/HYPERPLASIA	–	–	–	–	–	–	–	–	–	–	–	–
INFILTRATE, MIXED	**3**	**0.75**	**4**	**0.80**	2	0.40	**1**	**0.20**	**1**	**0.20**	**4**	**0.80**
MITOTIC FIGURES, INCREASED	–	–	–	–	–	–	–	–	–	–	–	–
NECROSIS	1	0.25	1	0.20	–	–	–	–	1	0.20	1	0.20
HEPATOCYTE												
NECROSIS, SINGLE CELL	–	–	–	–	–	–	–	–	–	–	–	–
VACUOLATION	–	–	–	–	–	–	–	–	–	–	–	–
PERIVASCULAR												
INFILTRATE, MONONUCLEAR CELLS	–	–	–	–	–	–	–	–	–	–	–	–
**KIDNEY**	4		5		5		5		5		5	
CHRONIC PROGRESSIVE NEPHROPATHY	1	0.25	1	0.20	–	–	–	–	–	–	–	–
INFILTRATE, MONONUCLEAR CELLS	–	–	–	–	–	–	–	–	–	–	–	–
TUBULAR												
ACCUMULATION, HYALINE DROPLETS	–	–	–	–	–	–	–	–	–	–	–	–
BASOPHILIA	–	–	1	0.20	–	–	–	–	–	–	–	–
CYST	–	–	–	–	–	–	1	0.20	–	–	–	–

Appendix 10

Summary Incidence and Average Severity Report

Study ID: 02776-0018: CRL 2954-001 / U of Tex Southwestern Med UTSW.Gray-002
Study Title: A SINGLE DOSE TOXICITY STUDY OF AAV9/SURF1 ADMINISTERED BY INTRATHECAL INJECTION IN RATS

This is a summary report and only displays those tissues which have at least one finding present for this study.

Sacrifice:	DAY 91		DAY 91		DAY 91		DAY 91		DAY 91		DAY 91	
Group:	3MD91		4MD91		1FD91		2FD91		3FD91		4FD91	
Dose:	0.83E12 VG		2.49E12 VG		0 VG		0.28E12 VG		0.83E12 VG		2.49E12 VG	
**Tissue** / *Site* / Diagnosis	INC	SEV	INC	SEV	INC	SEV	INC	SEV	INC	SEV	INC	SEV
DILATATION	1	0.25	-	-	-	-	-	-	-	-	-	-
**LYMPH NODE, MESENTERIC**	4		5		5		5		5		5	
PIGMENT	-	-	-	-	1	0.20	-	-	-	-	-	-
**TESTIS**	4		5		-		-		-		-	
TUBULAR												
DEGENERATION	-	-	-	-	-	-	-	-	-	-	-	-
**LYMPH NODE, ILIAC**	4		5		5		5		5		5	
INCREASED CELLULARITY	1	0.25	-	-	-	-	-	-	-	-	-	-

Appendix 10

Summary Incidence and Average Severity Report

Study ID: 02776-0018: CRL 2954-001 / U of Tex Southwestern Med UTSW.Gray-002
Study Title: A SINGLE DOSE TOXICITY STUDY OF AAV9/SURF1 ADMINISTERED BY INTRATHECAL INJECTION IN RATS

This is a summary report and only displays those tissues which have at least one finding present for this study.

Tissue / *Site* / Diagnosis	Sacrifice: US<br>Group: ED<br>Dose: 0.83E12 VG<br>INC	SEV
**NERVE ROOT, SPINAL, CERVICAL**	1	
EPINEURIUM		
INFILTRATE, MONONUCLEAR CELLS	-	-
NERVE ROOT, VENTRAL		
DEGENERATION, NERVE FIBER	-	-
**GANGLION, DORSAL ROOT, CERVICAL**	1	
INFILTRATE, MONONUCLEAR CELLS	-	-
GLIAL CELL		
HYPERTROPHY/HYPERPLASIA	-	-
**NERVE ROOT, SPINAL, THORACIC**	1	
EPINEURIUM		
INFILTRATE, MONONUCLEAR CELLS	1	1.00
NERVE ROOT, DORSAL		
DEGENERATION, NERVE FIBER	-	-
NERVE ROOT, VENTRAL		
DEGENERATION, NERVE FIBER	1	2.00
SCHWANN CELL		
HYPERTROPHY/HYPERPLASIA	1	1.00
**GANGLION, DORSAL ROOT, THORACIC**	1	
INFILTRATE, MONONUCLEAR CELLS	1	1.00
GLIAL CELL		
HYPERTROPHY/HYPERPLASIA	1	1.00
**NERVE ROOT, SPINAL, LUMBAR**	1	
EPINEURIUM		
INFILTRATE, MONONUCLEAR CELLS	-	-

Appendix 10

Summary Incidence and Average Severity Report

Study ID: 02776-0018: CRL 2954-001 / U of Tex Southwestern Med UTSW.Gray-002
Study Title: A SINGLE DOSE TOXICITY STUDY OF AAV9/SURF1 ADMINISTERED BY INTRATHECAL INJECTION IN RATS

This is a summary report and only displays those tissues which have at least one finding present for this study.

Tissue / *Site* / Diagnosis	Sacrifice: US	
	Group: ED	
	Dose: 0.83E12 VG	
	INC	SEV
NERVE ROOT, DORSAL		
DEGENERATION, NERVE FIBER	-	-
NERVE ROOT, VENTRAL		
DEGENERATION, NERVE FIBER	1	5.00
INFILTRATE, MONONUCLEAR CELLS	1	1.00
SCHWANN CELL		
HYPERTROPHY/HYPERPLASIA	1	3.00
**GANGLION, DORSAL ROOT, LUMBAR**	1	
INFILTRATE, MONONUCLEAR CELLS	-	-
GLIAL CELL		
HYPERTROPHY/HYPERPLASIA	1	1.00
NEURON		
DEGENERATION/NECROSIS	-	-
**BRAIN, MENINGES**	1	
INFILTRATE, MONONUCLEAR CELLS	-	-
**BRAIN, VENTRICULAR SYSTEM**	1	
INFILTRATE, MONONUCLEAR CELLS	1	1.00
**BRAIN, MIDBRAIN**	1	
PINEAL GLAND		
INFILTRATE, MONONUCLEAR CELLS	-	-
VACUOLATION	-	-
**BRAIN, CEREBELLUM**	1	
WHITE MATTER		
DEGENERATION, NERVE FIBER	-	-

Appendix 10

Summary Incidence and Average Severity Report

Study ID: 02776-0018: CRL 2954-001 / U of Tex Southwestern Med UTSW.Gray-002
Study Title: A SINGLE DOSE TOXICITY STUDY OF AAV9/SURF1 ADMINISTERED BY INTRATHECAL INJECTION IN RATS

This is a summary report and only displays those tissues which have at least one finding present for this study.

Tissue / *Site* / Diagnosis	Sacrifice: US	
	Group: ED	
	Dose: 0.83E12 VG	
	INC	SEV
**BRAIN, PONS**	1	
DEGENERATION, NERVE FIBER	–	–
**BRAIN, MEDULLA OBLONGATA**	1	
WHITE MATTER		
DEGENERATION, NERVE FIBER	–	–
**SPINAL CORD, CERVICAL**	1	
INFILTRATE, MONONUCLEAR CELLS	–	–
GLIAL CELL		
INCREASED CELLULARITY	1	1.00
GRAY MATTER		
INFILTRATE, MONONUCLEAR CELLS	1	1.00
MENINGES		
INFILTRATE, MONONUCLEAR CELLS	–	–
WHITE MATTER		
DEGENERATION, NERVE FIBER	–	–
**SPINAL CORD, THORACIC**	1	
GLIAL CELL		
INCREASED CELLULARITY	1	4.00
GRAY MATTER		
DEGENERATION/NECROSIS	1	5.00
INFILTRATE, MONONUCLEAR CELLS	1	2.00
INFLAMMATION	–	–
MENINGES		
INFILTRATE, MONONUCLEAR CELLS	–	–
NERVE ROOT, SPINAL		

Appendix 10

Summary Incidence and Average Severity Report

Study ID: 02776-0018: CRL 2954-001 / U of Tex Southwestern Med UTSW.Gray-002
Study Title: A SINGLE DOSE TOXICITY STUDY OF AAV9/SURF1 ADMINISTERED BY INTRATHECAL INJECTION IN RATS

This is a summary report and only displays those tissues which have at least one finding present for this study.

Tissue / *Site* / Diagnosis	Sacrifice: US	
	Group: ED	
	Dose: 0.83E12 VG	
	INC	SEV
DEGENERATION, NERVE FIBER	–	–
WHITE MATTER		
DEGENERATION, NERVE FIBER	**1**	**4.00**
**SPINAL CORD, LUMBAR**	1	
INFILTRATE, MONONUCLEAR CELLS	–	–
PIGMENT, HEMOSIDERIN	–	–
GLIAL CELL		
INCREASED CELLULARITY	**1**	**3.00**
GRAY MATTER		
DEGENERATION/NECROSIS	**1**	**1.00**
INFILTRATE, MONONUCLEAR CELLS	–	–
MENINGES		
INFILTRATE, MONONUCLEAR CELLS	–	–
MINERALIZATION	–	–
NERVE ROOT, SPINAL		
DEGENERATION, NERVE FIBER	**1**	**1.00**
WHITE MATTER		
DEGENERATION, NERVE FIBER	**1**	**1.00**
**NERVE, SCIATIC**	1	
DEGENERATION, NERVE FIBER	1	2.00
INFILTRATE, MIXED	–	–
SCHWANN CELL		
HYPERTROPHY/HYPERPLASIA	1	1.00
**NERVE, TIBIAL**	1	

Appendix 10

Summary Incidence and Average Severity Report

Study ID: 02776-0018: CRL 2954-001 / U of Tex Southwestern Med UTSW.Gray-002
Study Title: A SINGLE DOSE TOXICITY STUDY OF AAV9/SURF1 ADMINISTERED BY INTRATHECAL INJECTION IN RATS

This is a summary report and only displays those tissues which have at least one finding present for this study.

Tissue / *Site* / Diagnosis	Sacrifice: US<br>Group: ED<br>Dose: 0.83E12 VG<br>INC	SEV
DEGENERATION, NERVE FIBER	1	2.00
SCHWANN CELL		
HYPERTROPHY/HYPERPLASIA	–	–
**EYE**	1	
RETINAL ROSETTE	–	–
**THYMUS**	1	
INCREASED CELLULARITY	–	–
**PANCREAS**	1	
APOPTOSIS/SINGLE CELL NECROSIS	–	–
ATROPHY	–	–
FIBROSIS	–	–
INFILTRATE, MONONUCLEAR CELLS	–	–
MITOTIC FIGURES, INCREASED	–	–
**LUNG**	1	
INFLAMMATION	–	–
ALVEOLUS		
INFILTRATE, MACROPHAGES	–	–
PERIVASCULAR		
INFILTRATE, EOSINOPHILS	–	–
**MUSCLE, BICEPS FEMORIS**	1	
DEGENERATION/NECROSIS	–	–
**HEART**	1	

Appendix 10

Summary Incidence and Average Severity Report

Study ID: 02776-0018: CRL 2954-001 / U of Tex Southwestern Med UTSW.Gray-002
Study Title: A SINGLE DOSE TOXICITY STUDY OF AAV9/SURF1 ADMINISTERED BY INTRATHECAL INJECTION IN RATS

This is a summary report and only displays those tissues which have at least one finding present for this study.

Tissue / *Site* / Diagnosis	Sacrifice: US	
	Group: ED	
	Dose: 0.83E12 VG	
	INC	SEV
MYOCARDIUM		
DEGENERATION/NECROSIS	1	1.00
FIBROSIS	-	-
INFILTRATE, MONONUCLEAR CELLS	1	1.00
**SPLEEN**	1	
EXTRAMEDULLARY HEMATOPOIESIS	-	-
**LIVER**	1	
EXTRAMEDULLARY HEMATOPOIESIS	-	-
HYPERTROPHY/HYPERPLASIA	-	-
INFILTRATE, MIXED	**1**	**1.00**
MITOTIC FIGURES, INCREASED	-	-
NECROSIS	-	-
HEPATOCYTE		
NECROSIS, SINGLE CELL	-	-
VACUOLATION	-	-
PERIVASCULAR		
INFILTRATE, MONONUCLEAR CELLS	-	-
**KIDNEY**	1	
CHRONIC PROGRESSIVE NEPHROPATHY	1	1.00
INFILTRATE, MONONUCLEAR CELLS	-	-
TUBULAR		
ACCUMULATION, HYALINE DROPLETS	-	-
BASOPHILIA	-	-
CYST	-	-

Appendix 10

Summary Incidence and Average Severity Report

Study ID: 02776-0018: CRL 2954-001 / U of Tex Southwestern Med UTSW.Gray-002
Study Title: A SINGLE DOSE TOXICITY STUDY OF AAV9/SURF1 ADMINISTERED BY INTRATHECAL INJECTION IN RATS

This is a summary report and only displays those tissues which have at least one finding present for this study.

Tissue / *Site* / Diagnosis	Sacrifice: US<br>Group: ED<br>Dose: 0.83E12 VG INC	SEV
DILATATION	–	–
**LYMPH NODE, MESENTERIC**	1	
PIGMENT	–	–
**TESTIS**	1	
TUBULAR		
DEGENERATION	–	–
**LYMPH NODE, ILIAC**	1	
INCREASED CELLULARITY	–	–

DATA SECTION IV: GROSS LESION AND MICROSCOPIC CORRELATE REPORT

The Gross Lesion and Microscopic Correlate Report lists all gross lesions and their microscopic correlates.

- The gross lesion description includes the tissue and the text provided by the testing facility or recorded at the time of trimming.
- The microscopic correlate does not contain any comment(s) specific to that finding. Comments specific to individual microscopic findings are included in Data Section I and the Comments Report.

Appendix 10

Gross Lesion and Microscopic Correlate Report

Study ID: 02776-0018: CRL 2954-001 / U of Tex Southwestern Med UTSW.Gray-002
Study Title: A SINGLE DOSE TOXICITY STUDY OF AAV9/SURF1 ADMINISTERED BY INTRATHECAL INJECTION IN RATS

Group	Animal	TGL	Tissue	Site	Dx
3FD29	3523	1	LYMPH NODE, AXILLARY		

Gross Lesion: ENLARGEMENT; GENERALIZED, BILATERAL

Microscopic Correlate: *NO MICROSCOPIC CORRELATE*

Group	Animal	TGL	Tissue	Site	Dx
3FD29	3523	2	LYMPH NODE, INGUINAL		

Gross Lesion: ENLARGEMENT, GENERALIZED, BILATERAL

Microscopic Correlate: *NO MICROSCOPIC CORRELATE*

Group	Animal	TGL	Tissue	Site	Dx
4MD29	4024	1	LUNG		

Gross Lesion: DILATATION

Microscopic Correlate: *NO MICROSCOPIC CORRELATE*

Group	Animal	TGL	Tissue	Site	Dx
4MD91	4009	1	KIDNEY		

Gross Lesion: DILATATION; RIGHT (SEE COMMENTS REPORT)

Microscopic Correlate:

Appendix 10

DATA SECTION V: COMMENTS REPORT

This report lists all comments pertinent to the study, individual animals, and/or individual findings.

- Study (General) comments and animal comments not specifically related to a microscopic finding are only in this report (they are not included in Data Section I).

Appendix 10

Comments Report

Study ID: 02776-0018: CRL 2954-001 / U of Tex Southwestern Med UTSW.Gray-002
Study Title: A SINGLE DOSE TOXICITY STUDY OF AAV9/SURF1 ADMINISTERED BY INTRATHECAL INJECTION IN RATS

Group	Animal ID	Comment
		**GENERAL**
GENERAL	-	ALL HEART SECTIONS INCOMPLETE
GENERAL	-	HYPERTROPHY/HYPERPLASIA OF GLIAL CELLS IN THE DORSAL ROOT GANGLIA IS IN REFERENCE TO SATELLITE GLIAL CELLS
		**DAY 08**
1FD08	1515	CROSS SECTION NOT PRESENT ON ORIGINAL OR REWORK SLIDE<br>*RELATED FINDING:* *NERVE, TIBIAL: DEGENERATION, NERVE FIBER, MINIMAL*
1MD08	1015	TRANSVERSE SECTION NOT PRESENT FOR TIBIAL NERVE
2FD08	2511	PYRAMIDAL TRACT<br>*RELATED FINDING:* *BRAIN, MEDULLA OBLONGATA, WHITE MATTER: DEGENERATION, NERVE FIBER, MINIMAL*
2MD08	2011	AFFECTING KUPFFER CELLS<br>*RELATED FINDING:* *LIVER: HYPERTROPHY/HYPERPLASIA, MINIMAL*
2MD08	2015	SEEN IN LONG SECTION AND EXACT LOCATION COULD NOT BE DETERMINED<br>*RELATED FINDING:* *SPINAL CORD, LUMBAR, WHITE MATTER: DEGENERATION, NERVE FIBER, MINIMAL*
3FD08	3512	ADJACENT TO CENTRAL CANAL<br>*RELATED FINDING:* *SPINAL CORD, THORACIC, GRAY MATTER: INFILTRATE, MONONUCLEAR CELLS, MINIMAL, MULTIFOCAL, PERIVASCULAR*
3FD08	3512	PREDOMINANTLY AFFECTING GRAY MATTER REGIONS JUST ADJACENT TO THE CENTRAL CANAL<br>*RELATED FINDING:* *SPINAL CORD, THORACIC, GLIAL CELL: INCREASED CELLULARITY, MILD*
3FD08	3512	AFFECTED CELLS ARE ADJACENT TO THE CENTRAL CANAL<br>*RELATED FINDING:* *SPINAL CORD, THORACIC, GRAY MATTER: DEGENERATION/NECROSIS, MILD*
3FD08	3512	MINIMAL CHANGES SEEN IN BOTH LATERAL AND VENTRAL WHITE MATTER TRACTS<br>*RELATED FINDING:* *SPINAL CORD, THORACIC, WHITE MATTER: DEGENERATION, NERVE FIBER, MINIMAL*
3FD08	3515	SEEN IN LONG SECTION AND EXACT LOCATION COULD NOT BE DETERMINED<br>*RELATED FINDING:* *SPINAL CORD, THORACIC, WHITE MATTER: DEGENERATION, NERVE FIBER, MINIMAL*
3MD08	3013	AFFECTING KUPFFER CELLS<br>*RELATED FINDING:* *LIVER: HYPERTROPHY/HYPERPLASIA, MINIMAL*
3MD08	3013	OLFACTORY BULBS NOT PRESENT ON ORIGINAL OR REWORK SLIDE
3MD08	3014	CHANGE DEFINITIVE TO DORSAL TRACT SEEN IN CROSS SECTION AND CHANGE SEEN IN LONG SECTION WAS OF UNDETERMINED LOCATION (VENTRAL OR DORSAL)<br>*RELATED FINDING:* *SPINAL CORD, THORACIC, WHITE MATTER: DEGENERATION, NERVE FIBER, MINIMAL*
3MD08	3015	ASSOCIATED WITH BRIGHT EOSINOPHILIC ACELLULAR ELONGATE CRYSTALLINE MATERIAL.<br>*RELATED FINDING:* *LUNG: INFLAMMATION, MINIMAL, SUBACUTE, FOCAL*

Appendix 10

Comments Report

Study ID: 02776-0018: CRL 2954-001 / U of Tex Southwestern Med UTSW.Gray-002
Study Title: A SINGLE DOSE TOXICITY STUDY OF AAV9/SURF1 ADMINISTERED BY INTRATHECAL INJECTION IN RATS

Group	Animal ID	Comment
3MD08	3015	ONLY TRANSVERSE SECTION OF CERVICAL SPINAL CORD PRESENT
4FD08	4513	AFFECTING HEPATOCYTES IN PERIPORTAL REGIONS<br>*RELATED FINDING:* *LIVER, HEPATOCYTE: VACUOLATION, MINIMAL*
4FD08	4513	AFFECTING KUPFFER CELLS<br>*RELATED FINDING:* *LIVER: HYPERTROPHY/HYPERPLASIA, MINIMAL*
4MD08	4011	AFFECTING KUPFFER CELLS<br>*RELATED FINDING:* *LIVER: HYPERTROPHY/HYPERPLASIA, MINIMAL*
4MD08	4012	THYMUS NOT PRESENT ON SLIDE DESPITE REPRESENTATIVE SECTION OF WET TISSUE SUBMITTED BY TRIMMER.
		**DAY 29**
1MD29	1022	ILIAC LYMPH NODE NOT PRESENT ON SLIDE AND NOT AVAILABLE IN WET TISSUE FOR RETRIM ATTEMPT
1MD29	1023	PYRAMIDAL TRACT<br>*RELATED FINDING:* *BRAIN, MEDULLA OBLONGATA, WHITE MATTER: DEGENERATION, NERVE FIBER, MINIMAL*
1MD29	1025	SEEN IN LONG SECTION AND EXACT LOCATION COULD NOT BE DETERMINED<br>*RELATED FINDING:* *SPINAL CORD, LUMBAR, WHITE MATTER: DEGENERATION, NERVE FIBER, MINIMAL*
2FD29	2521	SEEN IN LONG SECTION AND EXACT LOCATION COULD NOT BE DETERMINED<br>*RELATED FINDING:* *SPINAL CORD, THORACIC, WHITE MATTER: DEGENERATION, NERVE FIBER, MINIMAL*
2FD29	2521	SEEN IN LONG SECTION AND EXACT LOCATION COULD NOT BE DETERMINED<br>*RELATED FINDING:* *SPINAL CORD, LUMBAR, WHITE MATTER: DEGENERATION, NERVE FIBER, MINIMAL*
2MD29	2023	SEEN IN LONG SECTION AND EXACT LOCATION COULD NOT BE DETERMINED<br>*RELATED FINDING:* *SPINAL CORD, THORACIC, WHITE MATTER: DEGENERATION, NERVE FIBER, MINIMAL*
2MD29	2024	SOME HEPATOCYTES ALSO HAVE BRIGHT EOSINOPHILIC INTRACYTOPLASMIC ‘INCLUSIONS’<br>*RELATED FINDING:* *LIVER, HEPATOCYTE: VACUOLATION, MILD, MULTIFOCAL*
2MD29	2024	AFFECTING ISLETS OF LANGERHANS; SOME AFFECTED ISLETS ALSO HAVE CHRONIC ACTIVE INFLAMMATION, HEMORRHAGE AND HEMOSIDERIN PIGMENT<br>*RELATED FINDING:* *PANCREAS: FIBROSIS, MINIMAL, MULTIFOCAL*
2MD29	2024	IN DORSAL WHITE MATTER AT JUNCTION WITH SPINAL NERVE<br>*RELATED FINDING:* *SPINAL CORD, CERVICAL, GLIAL CELL: INCREASED CELLULARITY, MINIMAL, FOCAL*
3FD29	3521	SEEN IN LONG SECTION AND EXACT LOCATION COULD NOT BE DETERMINED<br>*RELATED FINDING:* *SPINAL CORD, THORACIC, WHITE MATTER: DEGENERATION, NERVE FIBER, MINIMAL*
3FD29	3521	SEEN IN LONG SECTION AND EXACT LOCATION COULD NOT BE DETERMINED<br>*RELATED FINDING:* *SPINAL CORD, CERVICAL, WHITE MATTER: DEGENERATION, NERVE FIBER, MINIMAL*

Appendix 10

Comments Report

Study ID: 02776-0018: CRL 2954-001 / U of Tex Southwestern Med UTSW.Gray-002
Study Title: A SINGLE DOSE TOXICITY STUDY OF AAV9/SURF1 ADMINISTERED BY INTRATHECAL INJECTION IN RATS

Group	Animal ID	Comment
3FD29	3523	SEEN IN LONG SECTION AND EXACT LOCATION COULD NOT BE DETERMINED<br>*RELATED FINDING:* *SPINAL CORD, THORACIC, WHITE MATTER: DEGENERATION, NERVE FIBER, MINIMAL*
3FD29	3523	AFFECTING KUPFFER CELLS<br>*RELATED FINDING:* *LIVER: HYPERTROPHY/HYPERPLASIA, MINIMAL*
3FD29	3523	FORMING SMALL DISCRETE CLUSTERS CONSISTENT TO WHAT HAS BEEN DESCRIBED AS NAGEOTTE NODULES<br>*RELATED FINDING:* *GANGLION, DORSAL ROOT, LUMBAR, GLIAL CELL: HYPERTROPHY/HYPERPLASIA, MINIMAL, MULTIFOCAL*
3FD29	3524	OPTIC NERVES NOT PRESENT ON ORIGINAL OR REWORK SLIDES
3FD29	3525	A SINGLE DIGESTION CHAMBER IS PRESENT<br>*RELATED FINDING:* *BRAIN, PONS: DEGENERATION, NERVE FIBER, MINIMAL*
3FD29	3525	INFERIOR CEREBELLAR PEDUNCLE<br>*RELATED FINDING:* *BRAIN, MEDULLA OBLONGATA, WHITE MATTER: DEGENERATION, NERVE FIBER, MILD*
3FD29	3525	PREDOMINANTLY AFFECTING THE LATERAL AND VENTRAL HORNS BILATERALLY<br>*RELATED FINDING:* *SPINAL CORD, THORACIC, GRAY MATTER: DEGENERATION/NECROSIS, SEVERE*
3FD29	3525	PREDOMINANTLY WITHIN GRAY MATTER<br>*RELATED FINDING:* *SPINAL CORD, THORACIC, GLIAL CELL: INCREASED CELLULARITY, MILD*
3FD29	3525	WITHIN GRAY MATTER<br>*RELATED FINDING:* *SPINAL CORD, CERVICAL, GLIAL CELL: INCREASED CELLULARITY, MINIMAL, FOCAL*
3FD29	3525	AFFECTING CELLS WITHIN LATERAL AND VENTRAL HORNS BILATERALLY<br>*RELATED FINDING:* *SPINAL CORD, LUMBAR, GRAY MATTER: DEGENERATION/NECROSIS, MODERATE*
3FD29	3525	PREDOMINANTLY WITHIN GRAY MATTER<br>*RELATED FINDING:* *SPINAL CORD, LUMBAR, GLIAL CELL: INCREASED CELLULARITY, MILD*
3FD29	3525	THYMUS NOT PRESENT ON ORIGINAL OR REWORK SLIDE
3FD29	3525	MODERATE DEGENERATION WITHIN VENTRAL WHITE MATTER TRACTS AND MILD CHANGES WITHIN LATERAL WHITE MATTER TRACTS<br>*RELATED FINDING:* *SPINAL CORD, LUMBAR, WHITE MATTER: DEGENERATION, NERVE FIBER, MODERATE, VENTRAL*
3FD29	3525	MODERATE DEGENERATION BOTH WITH VENTRAL AND LATERAL WHITE MATTER TRACTS<br>*RELATED FINDING:* *SPINAL CORD, THORACIC, WHITE MATTER: DEGENERATION, NERVE FIBER, MODERATE, VENTRAL*
3MD29	3022	MILD DEGENERATION PRESENT WITHIN THE DORSAL WHITE MATTER TRACTS AND MINIMAL CHANGES PRESENT WITHIN THE LATERAL WHITE MATTER TRACTS<br>*RELATED FINDING:* *SPINAL CORD, LUMBAR, WHITE MATTER: DEGENERATION, NERVE FIBER, MILD, DORSAL*

Appendix 10

Comments Report

Study ID: 02776-0018: CRL 2954-001 / U of Tex Southwestern Med UTSW.Gray-002
Study Title: A SINGLE DOSE TOXICITY STUDY OF AAV9/SURF1 ADMINISTERED BY INTRATHECAL INJECTION IN RATS

Group	Animal ID	Comment
3MD29	3023	TRACT-LIKE LESION ALONG GRAY MATTER/LATERAL WHITE MATTER TRACT JUNCTION<br>*RELATED FINDING:* *SPINAL CORD, THORACIC, GRAY MATTER: INFLAMMATION, MINIMAL, LOCALLY EXTENSIVE*
3MD29	3023	THORACIC DORSAL ROOT GANGLION AND ASSOCIATED NERVE ROOTS NOT PRESENT ON ORIGINAL OR REWORK SLIDE
4FD29	4524	SEEN IN LONG SECTION AND EXACT LOCATION COULD NOT BE DETERMINED<br>*RELATED FINDING:* *SPINAL CORD, CERVICAL, WHITE MATTER: DEGENERATION, NERVE FIBER, MINIMAL*
4FD29	4524	ONE SIDE MINIMALLY AFFECTED<br>*RELATED FINDING:* *NERVE ROOT, SPINAL, LUMBAR, NERVE ROOT, DORSAL: DEGENERATION, NERVE FIBER, MILD*
4MD29	4024	SEEN IN LONG SECTION AND EXACT LOCATION COULD NOT BE DETERMINED<br>*RELATED FINDING:* *SPINAL CORD, LUMBAR, WHITE MATTER: DEGENERATION, NERVE FIBER, MINIMAL*
4MD29	4025	AFFECTING KUPFFER CELLS<br>*RELATED FINDING:* *LIVER: HYPERTROPHY/HYPERPLASIA, MILD*
4MD29	4026	SEEN IN LONG SECTION AND EXACT LOCATION COULD NOT BE DETERMINED<br>*RELATED FINDING:* *SPINAL CORD, THORACIC, WHITE MATTER: DEGENERATION, NERVE FIBER, MINIMAL*
4MD29	4027	AFFECTING KUPFFER CELLS<br>*RELATED FINDING:* *LIVER: HYPERTROPHY/HYPERPLASIA, MINIMAL*
		**DAY 91**
1FD91	1506	AFFECTING EPITHELIAL CELLS<br>*RELATED FINDING:* *THYMUS: INCREASED CELLULARITY, MINIMAL, FOCAL*
1FD91	1507	GREENISH GOLDEN BROWN AND GLOBULAR<br>*RELATED FINDING:* *LYMPH NODE, MESENTERIC: PIGMENT, MINIMAL*
1MD91	1007	SEEN IN LONG SECTION AND EXACT LOCATION COULD NOT BE DETERMINED<br>*RELATED FINDING:* *SPINAL CORD, THORACIC, WHITE MATTER: DEGENERATION, NERVE FIBER, MINIMAL*
1MD91	1007	CONSISTENT WITH TENSION LIPIDOSIS<br>*RELATED FINDING:* *LIVER, HEPATOCYTE: VACUOLATION, MINIMAL, LOCALLY EXTENSIVE*
1MD91	1007	TWO SETS OF EYES RECEIVED IN WET TISSUE FOR ANIMAL 1007 AND NONE FOR ANIMAL 1008. NEITHER SET OF EYES HAD MICROSCOPIC FINDINGS AND NEITHER COULD BE ASCRIBED TO ONE ANIMAL OR THE OTHER.
1MD91	1008	HEART APEX NOT PRESENT ON SLIDE AND ONLY ONE LIVER SECTION PRESENT ON SLIDE
1MD91	1008	AFFECTING THYMIC EPITHELIAL CELLS<br>*RELATED FINDING:* *THYMUS: INCREASED CELLULARITY, MINIMAL*
1MD91	1008	HYALINE DROPLETS<br>*RELATED FINDING:* *KIDNEY, TUBULAR: ACCUMULATION, HYALINE DROPLETS, MINIMAL*

Appendix 10

Comments Report

Study ID: 02776-0018: CRL 2954-001 / U of Tex Southwestern Med UTSW.Gray-002
Study Title: A SINGLE DOSE TOXICITY STUDY OF AAV9/SURF1 ADMINISTERED BY INTRATHECAL INJECTION IN RATS

Group	Animal ID	Comment
1MD91	1008	EYES NOT PRESENT ON SLIDE AND NOT PRESENT IN WET TISSUES FOR REWORK EFFORT
1MD91	1008	TWO SETS OF EYES RECEIVED IN WET TISSUE FOR ANIMAL 1007 AND NONE FOR ANIMAL 1008. NEITHER SET OF EYES HAD MICROSCOPIC FINDINGS AND NEITHER COULD BE ASCRIBED TO ONE ANIMAL OR THE OTHER.
1MD91	1009	PYRAMIDAL TRACT<br>*RELATED FINDING:* *BRAIN, MEDULLA OBLONGATA, WHITE MATTER: DEGENERATION, NERVE FIBER, MINIMAL*
1MD91	1009	SEEN IN LONG SECTION AND EXACT LOCATION COULD NOT BE DETERMINED<br>*RELATED FINDING:* *SPINAL CORD, THORACIC, WHITE MATTER: DEGENERATION, NERVE FIBER, MINIMAL*
1MD91	1010	AFFECTING ISLETS OF LANGERHANS<br>*RELATED FINDING:* *PANCREAS: FIBROSIS, MINIMAL, FOCAL*
1MD91	1010	MANDIBULAR LYMPH NODE NOT PRESENT ON ORIGINAL OR REWORK SLIDE
2FD91	2506	SEEN IN LONG SECTION AND EXACT LOCATION COULD NOT BE DETERMINED<br>*RELATED FINDING:* *SPINAL CORD, THORACIC, WHITE MATTER: DEGENERATION, NERVE FIBER, MINIMAL*
2FD91	2506	LIKELY REPRESENTING NEEDLE TRACT<br>*RELATED FINDING:* *SPINAL CORD, LUMBAR, GLIAL CELL: INCREASED CELLULARITY, MILD, LOCALLY EXTENSIVE*
2FD91	2506	ASSOCIATED WITH LOCALLY EXTENSIVE INCREASED GLIAL CELLULARITY<br>*RELATED FINDING:* *SPINAL CORD, LUMBAR: PIGMENT, HEMOSIDERIN, MINIMAL, LOCALLY EXTENSIVE*
2FD91	2507	AFFECTING EPITHELIAL CELLS<br>*RELATED FINDING:* *THYMUS: INCREASED CELLULARITY, MINIMAL, FOCAL*
2FD91	2508	LUMBAR SPINAL CORD SECTION COMPOSED PRIMARILY OF SPINAL NERVES
2FD91	2508	AFFECTING EPITHELIAL CELLS<br>*RELATED FINDING:* *THYMUS: INCREASED CELLULARITY, MINIMAL, MULTIFOCAL*
2FD91	2509	PYRAMIDAL TRACT<br>*RELATED FINDING:* *BRAIN, MEDULLA OBLONGATA, WHITE MATTER: DEGENERATION, NERVE FIBER, MINIMAL*
2FD91	2510	SEEN IN LONG SECTION AND EXACT LOCATION COULD NOT BE DETERMINED<br>*RELATED FINDING:* *SPINAL CORD, THORACIC, WHITE MATTER: DEGENERATION, NERVE FIBER, MINIMAL*
2MD91	2006	SEEN IN LONG SECTION AND EXACT LOCATION COULD NOT BE DETERMINED<br>*RELATED FINDING:* *SPINAL CORD, CERVICAL, WHITE MATTER: DEGENERATION, NERVE FIBER, MINIMAL*
2MD91	2007	MAY HAVE MINIMAL DECREASED NEURON CELLULARITY BUT CANNOT DEFINITELY DETERMINE DUE TO SOME PROCESSING/SECTIONING ARTIFACTS<br>*RELATED FINDING:* *GANGLION, DORSAL ROOT, LUMBAR: INFILTRATE, MONONUCLEAR CELLS, MILD, MULTIFOCAL*

Comments Report

Study ID: 02776-0018: CRL 2954-001 / U of Tex Southwestern Med UTSW.Gray-002
Study Title: A SINGLE DOSE TOXICITY STUDY OF AAV9/SURF1 ADMINISTERED BY INTRATHECAL INJECTION IN RATS

Group	Animal ID	Comment
2MD91	2008	PYRAMIDAL TRACT<br>*RELATED FINDING:* *BRAIN, MEDULLA OBLONGATA, WHITE MATTER: DEGENERATION, NERVE FIBER, MINIMAL*
2MD91	2009	PYRAMIDAL TRACT<br>*RELATED FINDING:* *BRAIN, MEDULLA OBLONGATA, WHITE MATTER: DEGENERATION, NERVE FIBER, MINIMAL*
2MD91	2009	SEEN IN LONG SECTION AND EXACT LOCATION COULD NOT BE DETERMINED<br>*RELATED FINDING:* *SPINAL CORD, THORACIC, WHITE MATTER: DEGENERATION, NERVE FIBER, MINIMAL*
2MD91	2010	SEEN IN LONG SECTION AND EXACT LOCATION COULD NOT BE DETERMINED<br>*RELATED FINDING:* *SPINAL CORD, THORACIC, WHITE MATTER: DEGENERATION, NERVE FIBER, MINIMAL*
3FD91	3508	ONE LUMBAR GANGLION AVAILABLE FOR EVALUATION
3FD91	3508	AFFECTING SIDE WITH NO DORSAL ROOT GANGLION PRESENT. PRESUMED TO BE VENTRAL NERVE ROOT DUE TO APPEARANCE OF NERVE FIBERS<br>*RELATED FINDING:* *NERVE ROOT, SPINAL, LUMBAR, NERVE ROOT, VENTRAL: DEGENERATION, NERVE FIBER, MILD*
3FD91	3509	SPINAL NERVES VARIABLY AFFECTED AND THOSE MOST AFFECTED HAVE A CONCOMITANT INCREASE IN SCHWANN CELLS<br>*RELATED FINDING:* *SPINAL CORD, LUMBAR, NERVE ROOT, SPINAL: DEGENERATION, NERVE FIBER, MARKED*
3FD91	3509	AFFECTING EPITHELIAL CELLS<br>*RELATED FINDING:* *THYMUS: INCREASED CELLULARITY, MINIMAL, FOCAL*
3FD91	3510	AFFECTING THE SPINAL TRACT OF THE TRIGEMINAL NERVE<br>*RELATED FINDING:* *BRAIN, MEDULLA OBLONGATA, WHITE MATTER: DEGENERATION, NERVE FIBER, MINIMAL*
3FD91	3510	SEEN IN LONG SECTION AND EXACT LOCATION COULD NOT BE DETERMINED<br>*RELATED FINDING:* *SPINAL CORD, THORACIC, WHITE MATTER: DEGENERATION, NERVE FIBER, MINIMAL*
3MD91	3006	INCREASED PLASMA CELLS WITHIN MEDULLARY CORDS<br>*RELATED FINDING:* *LYMPH NODE, ILIAC: INCREASED CELLULARITY, MINIMAL*
3MD91	3006	DEGENERATION ALSO SEEN IN LONG SECTION BUT EXACT LOCATION COULD NOT BE DETERMINED IN THIS ORIENTATION<br>*RELATED FINDING:* *SPINAL CORD, THORACIC, WHITE MATTER: DEGENERATION, NERVE FIBER, MINIMAL, DORSAL*
3MD91	3006	DEGENERATION ALSO SEEN IN LONG SECTION BUT EXACT LOCATION COULD NOT BE DETERMINED IN THIS ORIENTATION<br>*RELATED FINDING:* *SPINAL CORD, LUMBAR, WHITE MATTER: DEGENERATION, NERVE FIBER, MINIMAL, DORSAL*
3MD91	3007	SEEN IN LONG SECTION AND EXACT LOCATION COULD NOT BE DETERMINED<br>*RELATED FINDING:* *SPINAL CORD, CERVICAL, WHITE MATTER: DEGENERATION, NERVE FIBER, MINIMAL*

Appendix 10

Comments Report

Study ID: 02776-0018: CRL 2954-001 / U of Tex Southwestern Med UTSW.Gray-002
Study Title: A SINGLE DOSE TOXICITY STUDY OF AAV9/SURF1 ADMINISTERED BY INTRATHECAL INJECTION IN RATS

Group	Animal ID	Comment
3MD91	3007	SEEN IN LONG SECTION AND EXACT LOCATION COULD NOT BE DETERMINED<br>*RELATED FINDING:* *SPINAL CORD, THORACIC, WHITE MATTER: DEGENERATION, NERVE FIBER, MINIMAL*
3MD91	3009	PYRAMIDAL TRACT<br>*RELATED FINDING:* *BRAIN, MEDULLA OBLONGATA, WHITE MATTER: DEGENERATION, NERVE FIBER, MINIMAL*
3MD91	3009	SEEN IN LONG SECTION AND EXACT LOCATION COULD NOT BE DETERMINED<br>*RELATED FINDING:* *SPINAL CORD, THORACIC, WHITE MATTER: DEGENERATION, NERVE FIBER, MINIMAL*
3MD91	3009	SEEN IN LONG SECTION AND EXACT LOCATION COULD NOT BE DETERMINED<br>*RELATED FINDING:* *SPINAL CORD, CERVICAL, WHITE MATTER: DEGENERATION, NERVE FIBER, MINIMAL*
4FD91	4506	SEEN IN LONG SECTION AND EXACT LOCATION COULD NOT BE DETERMINED<br>*RELATED FINDING:* *SPINAL CORD, CERVICAL, WHITE MATTER: DEGENERATION, NERVE FIBER, MINIMAL*
4FD91	4507	PYRAMIDAL TRACT<br>*RELATED FINDING:* *BRAIN, MEDULLA OBLONGATA, WHITE MATTER: DEGENERATION, NERVE FIBER, MINIMAL*
4FD91	4508	SEEN IN LONG SECTION AND EXACT LOCATION COULD NOT BE DETERMINED<br>*RELATED FINDING:* *SPINAL CORD, THORACIC, WHITE MATTER: DEGENERATION, NERVE FIBER, MINIMAL*
4FD91	4508	NO CORNEA PRESENT IN SECTIONS OF EYE
4FD91	4509	PYRAMIDAL TRACT<br>*RELATED FINDING:* *BRAIN, MEDULLA OBLONGATA, WHITE MATTER: DEGENERATION, NERVE FIBER, MINIMAL*
4FD91	4510	SEEN IN LONG SECTION AND EXACT LOCATION COULD NOT BE DETERMINED<br>*RELATED FINDING:* *SPINAL CORD, CERVICAL, WHITE MATTER: DEGENERATION, NERVE FIBER, MINIMAL*
4MD91	4006	AFFECTING ISLETS OF LANGERHANS<br>*RELATED FINDING:* *PANCREAS: FIBROSIS, MINIMAL, FOCAL*
4MD91	4006	THYMUS NOT PRESENT ON ORIGINAL OR REWORK SLIDE
4MD91	4007	UNILATERAL WITHIN DORSALMOST PORTION OF GRAY MATTER<br>*RELATED FINDING:* *SPINAL CORD, THORACIC, GLIAL CELL: INCREASED CELLULARITY, MINIMAL, FOCAL*
4MD91	4007	SEEN IN LONG SECTION AND EXACT LOCATION COULD NOT BE DETERMINED<br>*RELATED FINDING:* *SPINAL CORD, CERVICAL, WHITE MATTER: DEGENERATION, NERVE FIBER, MINIMAL*
4MD91	4008	AFFECTING EXOCRINE/ACINAR CELLS<br>*RELATED FINDING:* *PANCREAS: ATROPHY, MINIMAL, FOCAL*
4MD91	4009	AFFECTING EXOCRINE/ACINAR CELLS<br>*RELATED FINDING:* *PANCREAS: ATROPHY, MINIMAL, FOCAL*

Appendix 10

Comments Report

Study ID: 02776-0018: CRL 2954-001 / U of Tex Southwestern Med UTSW.Gray-002
Study Title: A SINGLE DOSE TOXICITY STUDY OF AAV9/SURF1 ADMINISTERED BY INTRATHECAL INJECTION IN RATS

Group	Animal ID	Comment
4MD91	4009	AFFECTING ISLETS OF LANGERHANS<br>*RELATED FINDING:* *PANCREAS: FIBROSIS, MINIMAL, MULTIFOCAL*
4MD91	4009	SEEN IN LONG SECTION AND EXACT LOCATION COULD NOT BE DETERMINED<br>*RELATED FINDING:* *SPINAL CORD, THORACIC, WHITE MATTER: DEGENERATION, NERVE FIBER, MINIMAL*
4MD91	4009	RIGHT KIDNEY NOT SUBMITTED FOR HISTOPATHOLOGIC EVALUATION; COULD NOT CORRELATE MACROSCOPIC OBSERVATION OF DILATATION
4MD91	4010	AFFECTING ISLETS OF LANGERHANS<br>*RELATED FINDING:* *PANCREAS: FIBROSIS, MILD, MULTIFOCAL*
4MD91	4010	PYRAMIDAL TRACT<br>*RELATED FINDING:* *BRAIN, MEDULLA OBLONGATA, WHITE MATTER: DEGENERATION, NERVE FIBER, MINIMAL*
		**US**
ED	3008	PERIVENTRICULAR ADJACENT TO SUBFORNICAL ORGAN<br>*RELATED FINDING:* *BRAIN, VENTRICULAR SYSTEM: INFILTRATE, MONONUCLEAR CELLS, MINIMAL, FOCAL*
ED	3008	FOCAL REGIONS IN BOTH WHITE MATTER AND GRAY MATTER<br>*RELATED FINDING:* *SPINAL CORD, CERVICAL, GLIAL CELL: INCREASED CELLULARITY, MINIMAL*
ED	3008	WITHIN BOTH GRAY AND WHITE MATTER BUT PREDOMINANTLY WITHIN GRAY MATTER<br>*RELATED FINDING:* *SPINAL CORD, THORACIC, GLIAL CELL: INCREASED CELLULARITY, MARKED*
ED	3008	AFFECTING CELLS PREDOMINANTLY WITHIN LATERAL OR VENTRAL HORNS BILATERALLY<br>*RELATED FINDING:* *SPINAL CORD, LUMBAR, GRAY MATTER: DEGENERATION/NECROSIS, MINIMAL*
ED	3008	PREDOMINANTLY WITHIN GRAY MATTER<br>*RELATED FINDING:* *SPINAL CORD, LUMBAR, GLIAL CELL: INCREASED CELLULARITY, MODERATE*
ED	3008	AFFECTING CELLS PREDOMINANTLY WITHIN LATERAL OR VENTRAL HORNS<br>*RELATED FINDING:* *SPINAL CORD, THORACIC, GRAY MATTER: DEGENERATION/NECROSIS, SEVERE*

Appendix 10

DATA SECTION VI: FIGURES

Appendix 10

Representative Images from Study: **2954-001**

Figure 1

Group: 3 **Animal:** 3008

Tissue: THORACIC.SPINAL.CORD.TRANSVERSE

Slide: 7 **Stain:** HE **Mag:** 4X

Notes: DAY 16 EARLY DEATH GROUP 3 ANIMAL 3008. SEVERE DEGENERATION/NECROSIS (LOSS) OF LATERAL AND VENTRAL GRAY MATTER WITH MARKED INCREASES IN GLIAL CELLS AND MILD MULTIFOCAL PERIVASCULAR MONONCULEAR CELL INFILTRATES (ARROWS). CHANGES ARE BILATERAL AROUND CENTRAL CANAL (ASTERISK). THERE IS ASSOCIATED NERVE FIBER DEGENERATION OF THE LATERAL (L) AND VENTRAL (V) WHITE MATTER.

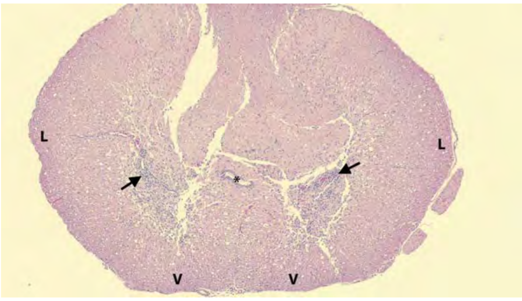

Figure 2

Group: 3 **Animal:** 3008

Tissue: THORACIC.SPINAL.CORD.LONG

Slide: 7 **Stain:** HE **Mag:** 4X

Notes: DAY 16 EARLY DEATH GROUP 3 ANIMAL 3008. SEVERE DEGENERATION/NECROSIS (LOSS) OF LATERAL AND VENTRAL GRAY MATTER (OUTLINED WITH ARROWHEADS) WITH MARKED INCREASES IN GLIAL CELLS. CHANGES ARE DIFFUSE THROUGH GRAY MATTER. THERE IS ASSOCIATED NERVE FIBER DEGENERATION OF WHITE MATTER.

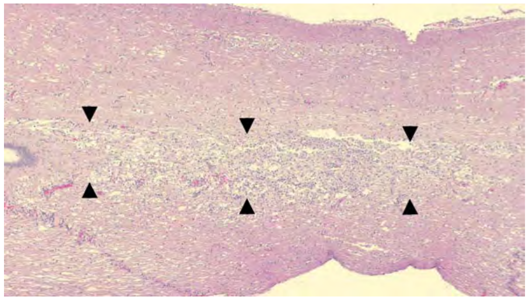

Representative Images from Study: **2954-001**

Figure 3

Group: 3 **Animal:** 3008

Tissue: LUMBAR.SPINAL.CORD.TRANSVERSE

Slide: 7 **Stain:** HE **Mag:** 4X

Notes: DAY 16 EARLY DEATH GROUP 3 ANIMAL 3008. MINIMAL DEGENERATION/NECROSIS (VACUOLAR CHANGE/LOSS) OF GRAY MATTER ADJACENT TO CENTRAL CANAL (ASTERISK) WITH MODERATE INCREASES IN GLIAL CELLS (WHITE ARROW). THERE IS ASSOCIATED NERVE FIBER DEGENERATION OF THE LATERAL (L) AND VENTRAL (V) WHITE MATTER.

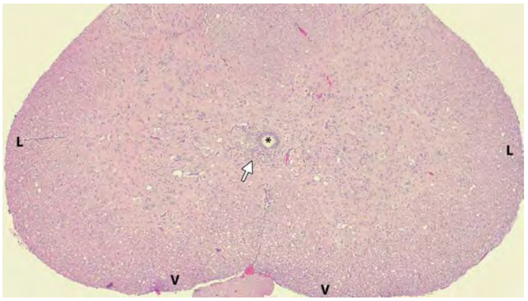

Figure 4

Group: 3 **Animal:** 3008

Tissue: LUMBAR.DORSAL.ROOT.GANGLION

Slide: 3 **Stain:** HE **Mag:** 10X

Notes: DAY 16 EARLY DEATH GROUP 3 ANIMAL 3008. SEVERE NERVE FIBER DEGENERATION OF VENTRAL NERVE ROOT ADJACENT TO THE LUMBAR DORSAL ROOT GANGLION (DRG). THERE IS FRAGMENTATION OF AXONS (ARROWS) AND DILATION OF MYELIN SHEATHS WITH MODERATE SCHWANN CELLS HYPERTROPHY/HYPERPLASIA AND MINIMAL MONONUCLEAR CELL INFILTRATES (ARROWHEADS).

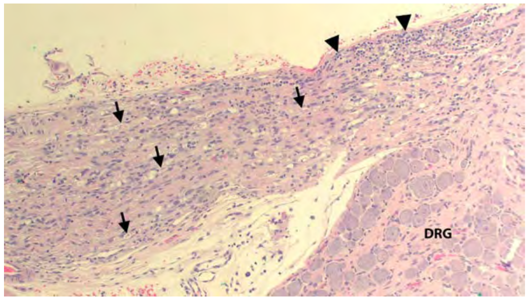

Representative Images from Study: **2954-001**

Figure 5

Group: 3 **Animal:** 3512

Tissue: THORACIC.SPINAL.CORD.TRANSVERSE

Slide: 7 **Stain:** HE **Mag:** 4X

Notes: DAY 8 GROUP 3 ANIMAL 3512. MILD DEGENERATION/NECROSIS OF GRAY MATTER ADJACENT TO CENTRAL CANAL WITH MILD INCREASES IN GLIAL CELLS (WHITE ARROW) AND MINIMAL PERIVASCULAR MONONUCLEAR CELL INFILTRATES (BLACK ARROW).

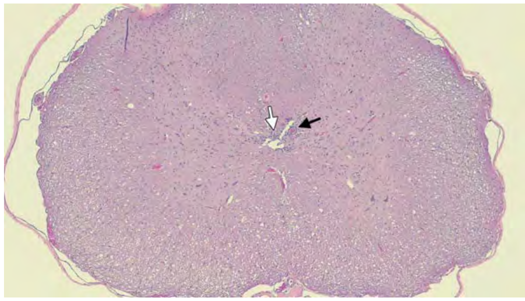

Figure 6

Group: 3 **Animal:** 3512

Tissue: THORACIC.SPINAL.CORD.LONG

Slide: 7 **Stain:** HE **Mag:** 4X

Notes: DAY 8 GROUP 3 ANIMAL 3512. MILD DEGENERATION/NECROSIS OF GRAY MATTER WITH MILD INCREASES IN GLIAL CELLS AND MINIMAL PERIVASCULAR MONONUCLEAR CELL INFILTRATES (BLACK ARROWS). THERE IS ARTIFACTUAL TEARING OF THE SECTION (ASTERISKS) AT THE CENTER OF THE GRAY MATTER.

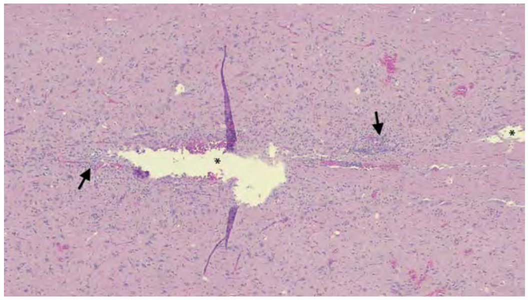

Representative Images from Study: **2954-001**

Figure 7

Group: 3 **Animal:** 3525

Tissue: THORACIC.SPINAL.CORD.TRANSVERSE

Slide: 7 **Stain:** HE **Mag:** 4X

Notes: DAY 29 GROUP 3 ANIMAL 3525. SEVERE DEGENERATION/NECROSIS (LOSS) OF LATERAL AND VENTRAL GRAY MATTER WITH MILD INCREASES IN GLIAL CELLS IN THE REMNANT LATERAL AND VENTRAL HORNS. CHANGES ARE BILATERAL AROUND CENTRAL CANAL (ASTERISK). THERE IS ASSOCIATED MODERATE NERVE FIBER DEGENERATION OF THE LATERAL (L) AND VENTRAL (V) WHITE MATTER.

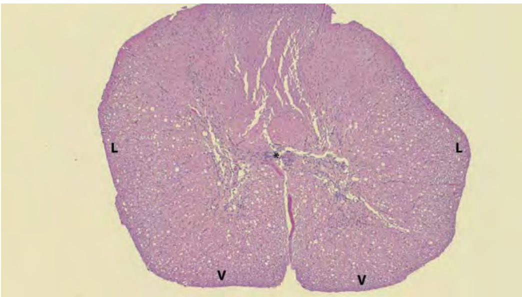

Figure 8

Group: 3 **Animal:** 3525

Tissue: THORACIC.SPINAL.CORD.LONG

Slide: 7 **Stain:** HE **Mag:** 4X

Notes: DAY 29 GROUP 3 ANIMAL 3525. SEVERE DEGENERATION/NECROSIS (LOSS) OF LATERAL AND VENTRAL GRAY MATTER (OUTLINED WITH ARROWHEADS) WITH MILD INCREASES IN GLIAL CELLS IN REMNANT GRAY MATTER AND MINIMAL PERIVASCULAR MONONUCLEAR CELL INFITLTRATES. CHANGES ARE DIFFUSE THROUGH GRAY MATTER. THERE IS ASSOCIATED NERVE FIBER DEGENERATION OF WHITE MATTER.

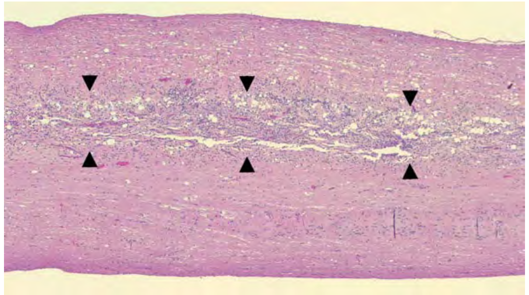

Representative Images from Study: **2954-001**

Figure 9

Group: 3 **Animal:** 3525

Tissue: LUMBAR.SPINAL.CORD.TRANSVERSE

Slide: 7 **Stain:** HE **Mag:** 4X

Notes: DAY 29 GROUP 3 ANIMAL 3525. MODERATE DEGENERATION/NECROSIS (VACUOLAR CHANGE/LOSS) OF LATERAL AND VENTRAL GRAY MATTER WITH MILD INCREASES IN GLIAL CELLS IN THE REMNANT LATERAL AND VENTRAL HORNS. CHANGES ARE BILATERAL AROUND CENTRAL CANAL (ASTERISK). THERE IS ASSOCIATED NERVE FIBER DEGENERATION OF THE LATERAL (L) AND VENTRAL (V) WHITE MATTER.

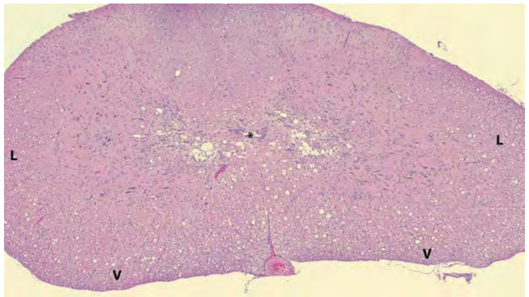

Figure 10

Group: 3 **Animal:** 3525

Tissue: LUMBAR.SPINAL.CORD.LONG

Slide: 7 **Stain:** HE **Mag:** 4X

Notes: DAY 29 GROUP 3 ANIMAL 3525. MODERATE DEGENERATION/NECROSIS OF GRAY MATTER WITH MILD INCREASES IN GLIAL CELLS IN REMNANT GRAY MATTER AND MINIMAL PERIVASCULAR MONONUCLEAR CELL INFITLTRATES (ARROWS). CHANGES ARE DIFFUSE THROUGH GRAY MATTER. THERE IS ASSOCIATED NERVE FIBER DEGENERATION OF WHITE MATTER.

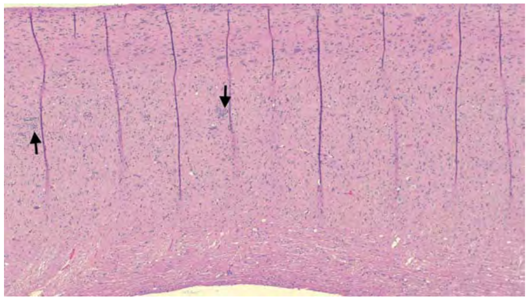

Representative Images from Study: **2954-001**

Figure 11

Group: 4 **Animal:** 4024

Tissue: LUMBAR.DORSAL.ROOT.GANGLION

Slide: 3 **Stain:** HE **Mag:** 10X

Notes: DAY 29 GROUP 4 ANIMAL 4024. MILD MONONUCLEAR CELL INFILTRATION (ARROW) OF THE LUMBAR DORSAL ROOT GANGLION AND MILD NERVE FIBER DEGENERATION (ARROWHEADS) OF THE DORSAL NERVE ROOT.

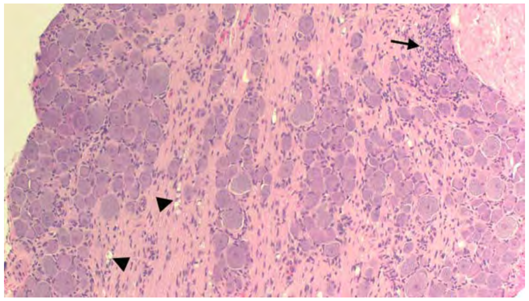

Figure 12

Group: 3 **Animal:** 3523

Tissue: LUMBAR.DORSAL.ROOT.GANGLION

Slide: 3 **Stain:** HE **Mag:** 20X

Notes: DAY 29 GROUP 3 ANIMAL 3523. MULTIFOCAL GLIAL CELL HYPERTROPHY/HYPERPLASIA (ARROWS) OF THE SATELLITE GLIAL CELLS WITHIN THE LUMBAR DORSAL ROOT GANGLION FORMING SMALL DISCRETE NODULES, CONSISTENT TO WHAT HAS BEEN DESCRIBED FOR NODULES OF NAGEOTTE.

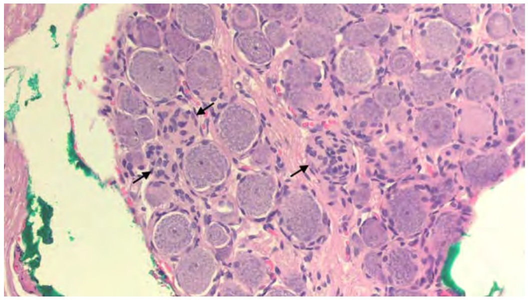

Representative Images from Study: **2954-001**

Figure 13

Group: 2 **Animal:** 2024

Tissue: HEART

Slide: 17 **Stain:** HE **Mag:** 2X

Notes: DAY 29 GROUP 2 ANIMAL 2024. MARKED DEGENERATION/NECROSIS AND MONONUCLEAR CELL INFILTRATES AND MILD FIBROSIS OF THE MYOCARDIUM MULTIFOCALLY AFFECTING THE HEART. THE LEFT VENTRICULAR FREE (LVF) WALL AND INTERVENTRICULAR SEPTUM (IVS) ARE DEPICTED, BUT ANY PORTION OF THE HEART COULD BE AFFECTED, INCLUDING ATRIA.

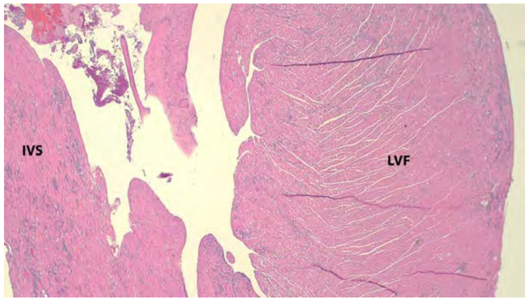

Figure 14

Group: 2 **Animal:** 2024

Tissue: HEART

Slide: 17 **Stain:** HE **Mag:** 10X

Notes: DAY 29 GROUP 2 ANIMAL 2024. HIGHER MAGNIFICATION OF THE MARKED DEGENERATION/NECROSIS AND MONONUCLEAR CELL INFILTRATES (BLACK ARROWHEADS) AND MILD FIBROSIS (WHITE ARROWHEADS) OF THE MYOCARDIUM MULTIFOCALLY AFFECTING THE HEART.

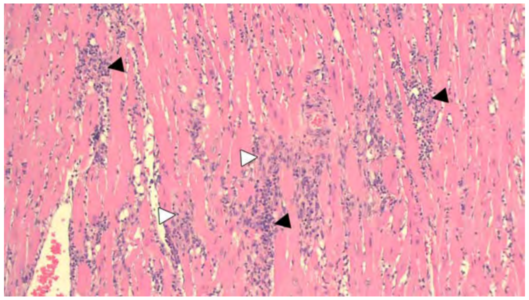

Appendix 10

Representative Images from Study: **2954-001**

Figure 15

Group: 4 **Animal:** 4025

Tissue: LIVER

Slide: 18 **Stain:** HE **Mag:** 20X

Notes: DAY 29 GROUP 4 ANIMAL 4025. MILD SINGLE CELL NECROSIS OF HEPATOCYTES (ARROWHEADS) WITH ASSOCIATED MIXED CELLULAR INFILTRATES (ARROW).

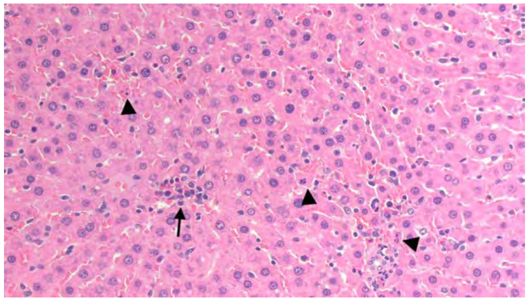

Figure 16

Group: 2 **Animal:** 2506

Tissue: LUMBAR.SPINAL.CORD

Slide: 7 **Stain:** HE **Mag:** 4X

Notes: DAY 91 GROUP 2 ANIMAL 2506. LOCALLY EXTENSIVE MILD INCREASE IN GLIAL CELLS PERPENDICULARLY ORIENTED TO THE WHITE MATTER NERVE FIBERS (DELINEATED BY ARROWHEADS) WITHIN THE LONGITUDINAL SECTION OF LUMBAR SPINAL CORD STRONGLY SUGGESTIVE OF A NEEDLE/INJECTION TRACT. THERE WAS ALSO MINIMAL GOLDEN-BROWN GRANULAR INTRACYTOPLASMIC PIGMENT CONSISTENT WITH HEMOSIDERIN PRESENT IN SMALL NUMBERS OF CELLS WITHIN THIS TRACT.

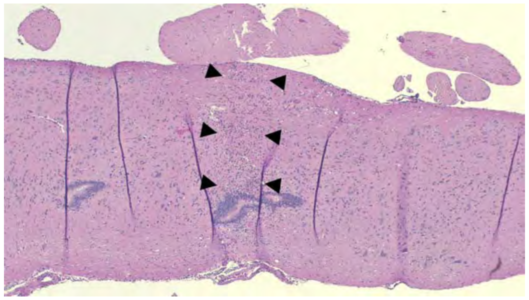

DATA SECTION VII: DIAGNOSIS EXPLANATION REPORT

Appendix 10

Diagnosis Explanation Report

ACCUMULATION, HYALINE DROPLETS

PRESENCE OF INTRACYTOPLASMIC EOSINOPHILIC MATERIAL REPRESENTING INTRA-LYSOSOMAL ACCUMULATION OF PROTEIN THAT TYPICALLY TAKES THE FORM OF SPHERICAL "DROPLETS" BUT OCCASIONALLY, PARTICULARLY IN MORE SEVERELY AFFECTED CELLS, CAN BECOME ENLARGED AND ANGULAR OR FORM CLUMPS. MOST FREQUENTLY SEEN IN PROXIMAL CONVOLUTED TUBULES OF THE KIDNEY AND CAN BE SEEN AS A NORMAL SPONTANEOUS MICROSCOPIC FINDING IN YOUNG ADULT MALE RATS.

**Severity Grade 1 (Minimal)**	DROPLETS BARELY VISIBLE BY ROUTINE LIGHT MICROSCOPY AND ARE PRESENT IN UP TO 25% OF RENAL CORTICAL TUBULES WITH AT MOST MINIMAL ASSOCIATED DEGENERATIVE CHANGES OF THE KIDNEY. DROPLETS ARE PREDOMINANTLY SPHERICAL AND NORMAL DROPLET CONGESTED CELLS ARE PRESENT.
**Severity Grade 2 (Mild)**	DROPLETS EASILY VISIBLE BY ROUTINE LIGHT MICROSCOPY AND ARE PRESENT IN APPROXIMATELY 25-30% OF RENAL CORTICAL TUBULES WITH AT MOST MINIMAL ASSOCIATED DEGENERATIVE CHANGES OF THE KIDNEY. DROPLETS ARE PREDOMINANTLY SPHERICAL AND NORMAL DROPLET CONGESTED CELLS ARE PRESENT.
**Severity Grade 3 (Moderate)**	DROPLETS VISIBLE BY ROUTINE LIGHT MICROSCOPY AND ARE PRESENT IN APPROXIMATELY 30-50% OF RENAL CORTICAL TUBULES TYPICALLY WITH MINIMAL OR GREATER ASSOCIATED DEGENERATIVE CHANGES OF THE KIDNEY. DROPLETS ARE PREDOMINANTLY SPHERICAL BUT ANGULAR FORMS CAN BE SEEN. DROPLET CONGESTED CELLS ARE PRESENT AT DECREASED NUMBERS OR TOTALLY ABSENT.
**Severity Grade 4 (Marked)**	DROPLETS VISIBLE BY ROUTINE LIGHT MICROSCOPY AND ARE PRESENT IN APPROXIMATELY 50-75% OF RENAL CORTICAL TUBULES WITH ASSOCIATED DEGENERATIVE CHANGES OF THE KIDNEY. DROPLETS ARE PREDOMINANTLY ANGULAR FORMS OR FORMING CLUMPS. DROPLET CONGESTED CELLS ARE ABSENT.
**Severity Grade 5 (Severe)**	DROPLETS VISIBLE BY ROUTINE LIGHT MICROSCOPY AND ARE PRESENT IN GREATER THAN 75% OF RENAL CORTICAL TUBULES WITH ASSOCIATED DEGENERATIVE CHANGES OF THE KIDNEY. DROPLETS ARE PREDOMINANTLY ANGULAR FORMS OR FORMING CLUMPS. DROPLET CONGESTED CELLS ARE ABSENT.

APOPTOSIS/SINGLE CELL NECROSIS

RANDOMLY, SCATTERED INDIVIDUAL DEAD CELLS OF A SPECIFIC CELL TYPE WITHIN THE TISSUE PARENCHYMA OR MUCOSA. TYPICALLY CHARACTERIZED BY EOSINOPHILIC NECROTIC DEBRIS AND/OR VARIABLY SIZED AGGREGATES OF CONDENSED CHROMATIN (APOPTOTIC BODIES).

**Severity Grade 1 (Minimal)**	AFFECTS FEW SPORADIC CELLS OR A VERY SMALL PERCENTAGE (<2%) OF A TISSUE REGION/SITE; TYPICALLY NOT BIOLOGICALLY RELEVANT IN NON-NERVOUS SYSTEM ORGANS.
**Severity Grade 2 (Mild)**	AFFECTS MORE THAN A FEW SPORADIC CELLS (UP TO 5%), MORE READILY APPARENT AS COMPARED TO A MINIMAL GRADE; UNLIKELY TO PRODUCE ANY STRUCTURAL OR FUNCTIONAL IMPAIRMENT.
**Severity Grade 3 (Moderate)**	AFFECTS UP TO 15% OF A PARTICULAR CELL TYPE; READILY APPARENT; FUNCTIONAL IMPAIRMENT POSSIBLE, BUT STILL CONSIDERED UNLIKELY.
**Severity Grade 4 (Marked)**	AFFECTS UP TO 40% OF A PARTICULAR CELL TYPE; PROMINENT AND CONSPICUOUS CHANGE; MAY PRODUCE FUNCTIONAL IMPAIRMENT.
**Severity Grade 5 (Severe)**	AFFECTS >40% OF A PARTICULAR CELL TYPE; PROMINENT, CONSPICUOUS, AND EFFACES TISSUE STRUCTURE; LIKELY TO PRODUCE FUNCTIONAL IMPAIRMENT.

Diagnosis Explanation Report

ATROPHY

SHRINKAGE OF THE SIZE OF THE CELL BY LOSS OF CELL SUBSTANCE. ATROPHY IS NOT A DEGENERATIVE CHANGE. ATROPHY IS TYPICALLY REVERSIBLE.

**Severity Grade 1 (Minimal)**	THE DEGREE OF ATROPHY IS PERCEPTIBLE, BUT ONLY AFTER CAREFUL COMPARISON TO CONCURRENT CONTROLS.
**Severity Grade 2 (Mild)**	THE DEGREE OF ATROPHY IS RECOGNIZABLE EVEN WITHOUT COMPARISON TO CONTROLS, BUT THE OVERALL DECREASE IN SIZE OF THE AFFECTED CELLS OR TISSUE IS STILL SLIGHT.
**Severity Grade 3 (Moderate)**	THE DEGREE OF ATROPHY IMPARTS AN EASILY RECOGNIZED CHANGE IN THE SIZE OF THE AFFECTED CELLS OR TISSUE.
**Severity Grade 4 (Marked)**	THE DEGREE OF ATROPHY IMPARTS A PRONOUNCED CHANGE IN THE SIZE OF THE AFFECTED CELLS OR TISSUE.
**Severity Grade 5 (Severe)**	THE DEGREE OF ATROPHY IMPARTS A VERY PRONOUNCED CHANGE IN THE SIZE OF THE AFFECTED CELLS OR TISSUE.

BASOPHILIA

THIS REFERS TO THE PRESENCE OF CELLS THAT ARE MORE BASOPHILIC THAN SURROUNDING TISSUE. BASOPHILIA MAY BE A COMPONENT OF PANCREATIC ACINAR HYPERPLASIA WHICH OCCURS AS A SPONTANEOUS BACKGROUND FINDING IN RATS.

**Severity Grade 1 (Minimal)**	LESS THAN APPROXIMATELY 1% OF THE TISSUE IS AFFECTED.
**Severity Grade 2 (Mild)**	APPROXIMATELY 2-5% OF THE TISSUE IS AFFECTED.
**Severity Grade 3 (Moderate)**	APPROXIMATELY 6-15% OF THE TISSUE IS AFFECTED.
**Severity Grade 4 (Marked)**	APPROXIMATELY 16-40% OF THE TISSUE IS AFFECTED.
**Severity Grade 5 (Severe)**	OVER APPROXIMATELY 40% OF THE TISSUE IS AFFECTED.

Appendix 10

Diagnosis Explanation Report

CHRONIC PROGRESSIVE NEPHROPATHY

THIS IS A SPONTANEOUS CHANGE THAT OCCURS IN AGING RATS. IT IS COMPRISED OF A SPECTRUM OF LESIONS WHICH MAY INCLUDE THE FOLLOWING: BASOPHILIC TUBULES WITHIN THE CORTEX, HYALINE CASTS, INTERSTITIAL INFLAMMATORY CELL INFILTRATION, INTERSTITIAL FIBROSIS, AND GLOMERULOSCLEROSIS AND/OR GLOMERULAR ATROPHY. IT OCCURS MORE COMMONLY IN MALES.

**Severity Grade 1 (Minimal)**	LESS THAN APPROXIMATELY 1% OF THE TISSUE IS AFFECTED.
**Severity Grade 2 (Mild)**	APPROXIMATELY 2-5% OF THE TISSUE IS AFFECTED.
**Severity Grade 3 (Moderate)**	APPROXIMATELY 6-15% OF THE TISSUE IS AFFECTED.
**Severity Grade 4 (Marked)**	APPROXIMATELY 16-40% OF THE TISSUE IS AFFECTED.
**Severity Grade 5 (Severe)**	OVER APPROXIMATELY 40% OF THE TISSUE IS AFFECTED.

CYST

AN EPITHELIAL-LINED, TYPICALLY FLUID-FILLED STRUCTURE. A CYST MAY DEVELOP FROM LOCAL EPITHELIAL TISSUE, MAY ARISE FROM AN EMBRYONIC REMNANT OF TISSUE, OR MAY HAVE ANOTHER CAUSE.

**Severity Grade 1 (Minimal)**	SMALL; NOT READILY DISCERNIBLE; MAY REPRESENT A SLIGHT DILATION OF A LOCAL STRUCTURE.
**Severity Grade 2 (Mild)**	READILY APPARENT; MAY REPRESENT A MORE PRONOUNCED (THAN MINIMAL) DILATION OF A LOCAL STRUCTURE.
**Severity Grade 3 (Moderate)**	VERY APPARENT; MAY ALTER THE ARCHITECTURE OF THE TISSUE; MAY REPRESENT A MORE PRONOUNCED (THAN MILD) DILATION OF A LOCAL STRUCTURE.
**Severity Grade 4 (Marked)**	VERY APPARENT; MAY DRAMATICALLY ALTER THE ARCHITECTURE OF THE TISSUE; MAY REPRESENT A MORE PRONOUNCED (THAN MODERATE) DILATION OF A LOCAL STRUCTURE.
**Severity Grade 5 (Severe)**	VERY APPARENT; EFFACES THE ARCHITECTURE OF THE TISSUE; MAY REPRESENT A VERY PRONOUNCED DILATION OF A LOCAL STRUCTURE.

Diagnosis Explanation Report

DEGENERATION

CHANGE IN A GIVEN CELL TYPE OR TISSUE CHANGING THAT CELL TYPE/TISSUE TO A (GENERALLY) LESS FUNCTIONAL FORM; IN NERVES OR TRACTS OF NERVES (EITHER A PERIPHERAL NERVE OR A NERVE/NERVE TRACT IN THE BRAIN OR SPINAL CORD), THE TERM IMPLIES DEGENERATION OF MYELIN AND/OR AXON(S) SINCE A DISTINCTION IS NOT TYPICALLY POSSIBLE IN PARAFFIN-EMBEDDED, H&E-STAINED SECTIONS.

**Severity Grade 1 (Minimal)**	ONE OR A FEW FOCI OF DEGENERATION.
**Severity Grade 2 (Mild)**	UP TO 5% (ESTIMATED) OF THE CELLS/STRUCTURES IN A GIVEN TISSUE/REGION (NERVE, SPINAL CORD TRACT, BRAIN REGION, ETC.) DEGENERATED.
**Severity Grade 3 (Moderate)**	5 TO 15% (ESTIMATED) OF THE CELLS/STRUCTURES IN A GIVEN TISSUE/REGION (NERVE, SPINAL CORD TRACT, BRAIN REGION, ETC.) DEGENERATED.
**Severity Grade 4 (Marked)**	15 TO 40% (ESTIMATED) OF THE CELLS/STRUCTURES IN A GIVEN TISSUE/REGION (NERVE, SPINAL CORD TRACT, BRAIN REGION, ETC.) DEGENERATED.
**Severity Grade 5 (Severe)**	GREATER THAN 40% (ESTIMATED) OF THE CELLS/STRUCTURES IN A GIVEN TISSUE/REGION (NERVE, SPINAL CORD TRACT, BRAIN REGION, ETC.) DEGENERATED.

DEGENERATION, NERVE FIBER

NERVE FIBER DEGENERATION REFERS TO DEGENERATION OCCURRING IN EITHER AXONS AND/OR MYELIN.

**Severity Grade 1 (Minimal)**	VERY FEW NERVE FIBERS ARE AFFECTED (LESS THAN APPROXIMATELY 1%).
**Severity Grade 2 (Mild)**	APPROXIMATELY 2-5% OF NERVE FIBERS ARE AFFECTED.
**Severity Grade 3 (Moderate)**	APPROXIMATELY 6-15% OF NERVE FIBERS ARE AFFECTED.
**Severity Grade 4 (Marked)**	APPROXIMATELY 16-40% OF NERVE FIBERS ARE AFFECTED.
**Severity Grade 5 (Severe)**	APPROXIMATELY OVER 40% OF NERVE FIBERS ARE AFFECTED.

DEGENERATION/NECROSIS

A CONTINUUM OF CHANGES IN CELLULAR MORPHOLOGY INDICATIVE OF CELLULAR DAMAGE (CYTOPLASMIC SWELLING, VACUOLATION, EOSINOPHILIA, FRAGMENTATION) THAT IN SOME CELLS RESULTS IN CELL DEATH.

**Severity Grade 1 (Minimal)**	AFFECTING FEW SCATTERED CELLS OR SMALL FOCUS OF CELLS.
**Severity Grade 2 (Mild)**	MORE THAN A FEW SCATTERED OR SINGLE FOCUS OF CELLS AFFECTED BUT AFFECTING LESS THAN 10% OF TISSUE SECTION.
**Severity Grade 3 (Moderate)**	AFFECTING 10-40% OF TISSUE.
**Severity Grade 4 (Marked)**	AFFECTING 41-70% OF TISSUE.
**Severity Grade 5 (Severe)**	AFFECTING GREATER THAN 70% OF TISSUE.

Diagnosis Explanation Report

DILATATION

SYNONYMOUS WITH DILATION (DILATATION IS THE SEND/CDISC CONTROLLED TERM). ABNORMAL ENLARGEMENT OF AN ORGAN (EXAMPLE: URINARY BLADDER) OR PART OF AN ORGAN (EXAMPLE: RENAL TUBULE). TYPICALLY, THIS TERM IS USED TO NOTE THE DILATION OF A HOLLOW ORGAN OR A STRUCTURE WITH A LUMEN.

**Severity Grade 1 (Minimal)**	VERY SLIGHT ENLARGEMENT/DILATION.
**Severity Grade 2 (Mild)**	ENLARGEMENT/DILATION MORE APPARENT THAN MINIMAL BUT NOT CONSIDERED PRONOUNCED.
**Severity Grade 3 (Moderate)**	PRONOUNCED ENLARGEMENT/DILATION; MAY DISTORT SURROUNDING TISSUE/TISSUES.
**Severity Grade 4 (Marked)**	VERY PRONOUNCED ENLARGEMENT/DILATION; DISTORTS SURROUNDING TISSUE/TISSUES.
**Severity Grade 5 (Severe)**	EXTREME ENLARGEMENT/DILATION THAT NOTABLY DISTORTS SURROUNDING TISSUE/TISSUES.

EXTRAMEDULLARY HEMATOPOIESIS

PRESENCE OF HEMATOPOIETIC PRECURSOR CELLS IN A TISSUE OUTSIDE THE BONE MARROW.

**Severity Grade 1 (Minimal)**	FEW, SCATTERED CLUSTERS OF PRECURSOR CELLS (LESS THAN 1% OF THE TISSUE AFFECTED).
**Severity Grade 2 (Mild)**	APPROXIMATELY 2-5% OF THE TISSUE CONTAINS PRECURSOR CELLS.
**Severity Grade 3 (Moderate)**	APPROXIMATELY 6-15% OF THE TISSUE CONTAINS PRECURSOR CELLS.
**Severity Grade 4 (Marked)**	APPROXIMATELY 16-40% OF THE TISSUE CONTAINS PRECURSOR CELLS.
**Severity Grade 5 (Severe)**	LARGE PORTIONS OF THE TISSUE CONTAIN PRECURSOR CELLS (0VER 40% OF THE TISSUE AFFECTED).

FIBROSIS

INCREASE OF CONNECTIVE TISSUE, USUALLY IN RESPONSE TO PRIOR OR CONCURRENT TISSUE DAMAGE/INFLAMMATION; MAY VARY IN THE EXTENT OF CELLULARITY (FIBROBLASTS) AND COLLAGEN; MAY BE OBSERVED IN CONTROLS.

**Severity Grade 1 (Minimal)**	FOCAL CHANGE THAT BARELY EXCEEDS THE NORMAL CONDITION.
**Severity Grade 2 (Mild)**	FOCAL TO MORE EXTENSIVE CHANGE THAT IS MORE READILY APPARENT THAN A MINIMAL CHANGE.
**Severity Grade 3 (Moderate)**	FOCALLY EXTENSIVE TO MORE DIFFUSE CHANGE THAT AFFECTS A PROMINENT PORTION OF A TISSUE/REGION.
**Severity Grade 4 (Marked)**	FOCALLY EXTENSIVE TO MORE DIFFUSE CHANGE THAT NOTABLY AFFECTS A TISSUE/REGION, CAUSING IMPAIRMENT OF THE STRUCTURE OF THE REGION/TISSUE.
**Severity Grade 5 (Severe)**	VERY EXTENSIVE TO DIFFUSE CHANGE THAT DRAMATICALLY AFFECTS A TISSUE/REGION, CAUSING IMPAIRMENT OF THE STRUCTURE OF THE REGION/TISSUE.

Diagnosis Explanation Report

HYPERTROPHY/HYPERPLASIA

INCREASE IN SIZE AND NUMBER OF A PARTICULAR CELL TYPE. WHEN USED TO DESCRIBE SATELLITE GLIAL CELLS, HYPERTROPHY/HYPERPLASIA TYPICALLY INDICATES THE FORMATION OF A NAGEOTTE NODULE, USUALLY FORMED IN REACTION TO LOSS OF A NEURON.

**Severity Grade 1 (Minimal)**	ONE OR A FEW FOCI; BARELY OUTSIDE OF NORMAL.
**Severity Grade 2 (Mild)**	ONE OR A FEW FOCI, BUT MORE READILY APPARENT THAN A MINIMAL CHANGE.
**Severity Grade 3 (Moderate)**	CHANGE THAT AFFECTS UP TO 20% OF A PARTICULAR TISSUE OR CELL TYPE.
**Severity Grade 4 (Marked)**	CHANGE THAT AFFECTS 20 TO 50% OF A PARTICULAR TISSUE OR CELL TYPE.
**Severity Grade 5 (Severe)**	PRONOUNCED CHANGE THAT AFFECTS GREATER THAN 50% OF A PARTICULAR TISSUE OR CELL TYPE.

INCREASED CELLULARITY

USED AS A DESCRIPTIVE DIAGNOSIS TO INDICATE THE INCREASE OF A CELL TYPE. EXAMPLE: "BONE MARROW, INCREASED CELLULARITY" MAY BE USED TO DENOTE AN INCREASE IN THE QUANTITY OF HEMATOPOIETIC CELLS IN BONE MARROW WHEN IT IS FELT THAT A DIAGNOSIS OF HYPERPLASIA IS NOT APPROPRIATE.

**Severity Grade 1 (Minimal)**	INCREASE IS BARELY ABOVE NORMAL LIMITS.
**Severity Grade 2 (Mild)**	INCREASE IS MORE READILY APPARENT THAN A MINIMAL CHANGE, BUT IS STILL JUST ABOVE NORMAL LIMITS.
**Severity Grade 3 (Moderate)**	THE INCREASE IS READILY APPARENT, AND EASILY DIFFERENTIATED FROM NORMAL LIMITS.
**Severity Grade 4 (Marked)**	INCREASE IS PRONOUNCED.
**Severity Grade 5 (Severe)**	INCREASE IS VERY PRONOUNCED.

Diagnosis Explanation Report

INFILTRATE, EOSINOPHILS

RECOGNIZABLE LEUKOCYTE INFILTRATE OF EOSINOPHILS, TYPICALLY WITHOUT ASSOCIATED INDICATORS OF TISSUE DAMAGE; EOSINOPHILS TYPICALLY RESPOND TO FOREIGN MATERIALS (PARASITES; DEVICES) OR OTHER PERSISTENT ANTIGENIC STIMULI; MAY BE PROMINENT IN ALLERGIC REACTIONS.

**Severity Grade 1 (Minimal)**	INFILTRATE BARELY EXCEEDS NORMAL LIMITS; TYPICALLY FOCAL AND/OR SPORADIC, AND OF NO BIOLOGIC RELEVANCE.
**Severity Grade 2 (Mild)**	INFILTRATE MORE READILY APPARENT THAN A MINIMAL INFILTRATE BUT STILL UNLIKELY TO PRODUCE ANY STRUCTURAL OR FUNCTIONAL IMPAIRMENT; MAY BE PRESENT IN ONE OR MORE FOCI.
**Severity Grade 3 (Moderate)**	INFILTRATE READILY APPARENT; POSSIBLE, BUT STILL CONSIDERED UNLIKELY, TO PRODUCE ANY FUNCTIONAL IMPAIRMENT; MAY BE FOCAL, MULTIFOCAL, OR DIFFUSE.
**Severity Grade 4 (Marked)**	INFILTRATE THAT IS PROMINENT, CONSPICUOUS, BUT NOT CONSIDERED TO BE THE MAXIMUM POSSIBLE EFFECT. MAY PRODUCE STRUCTURAL/FUNCTIONAL IMPAIRMENT.
**Severity Grade 5 (Severe)**	INFILTRATE THAT IS PROMINENT, CONSPICUOUS, AND EFFACES TISSUE STRUCTURE; LIKELY TO PRODUCE STRUCTURAL/FUNCTIONAL IMPAIRMENT.

INFILTRATE, MACROPHAGES

RECOGNIZABLE LEUKOCYTE INFILTRATE OF MONONUCLEAR CELLS MORPHOLOGICALLY CONSISTENT WITH MACROPHAGES, TYPICALLY WITHOUT ASSOCIATED INDICATORS OF TISSUE DAMAGE.

**Severity Grade 1 (Minimal)**	INFILTRATE BARELY EXCEEDS NORMAL LIMITS; TYPICALLY FOCAL AND/OR SPORADIC, AND OF NO BIOLOGIC RELEVANCE; AFFECTS UP TO 1% (TYPICALLY) OF A TISSUE OR REGION.
**Severity Grade 2 (Mild)**	INFILTRATE MORE READILY APPARENT THAN A MINIMAL INFILTRATE BUT STILL UNLIKELY TO PRODUCE ANY STRUCTURAL OR FUNCTIONAL IMPAIRMENT; MAY BE PRESENT IN ONE OR MORE FOCI; TYPICALLY AFFECTS 1 TO 5% OF A TISSUE OR REGION.
**Severity Grade 3 (Moderate)**	INFILTRATE READILY APPARENT; POSSIBLE, BUT STILL CONSIDERED UNLIKELY, TO PRODUCE ANY FUNCTIONAL IMPAIRMENT; MAY BE FOCAL, MULTIFOCAL, OR DIFFUSE; TYPICALLY AFFECTS 5 TO 15% OF A TISSUE OR REGION.
**Severity Grade 4 (Marked)**	INFILTRATE THAT IS PROMINENT, CONSPICUOUS, BUT NOT CONSIDERED TO BE THE MAXIMUM POSSIBLE EFFECT. MAY PRODUCE STRUCTURAL/FUNCTIONAL IMPAIRMENT; TYPICALLY AFFECTS 15 TO 40% OF A TISSUE OR REGION.
**Severity Grade 5 (Severe)**	INFILTRATE THAT IS PROMINENT, CONSPICUOUS, AND EFFACES TISSUE STRUCTURE; LIKELY TO PRODUCE STRUCTURAL/FUNCTIONAL IMPAIRMENT; TYPICALLY AFFECTS >40% OF A TISSUE OR REGION.

Diagnosis Explanation Report

INFILTRATE, MIXED

RECOGNIZABLE LEUKOCYTE INFILTRATE COMPOSED OF A MIX OF MONONUCLEAR AND POLYMORPHONUCLEAR (NEUTROPHILS AND/OR EOSINOPHILS) CELLS.

**Severity Grade 1 (Minimal)**	INFILTRATE BARELY EXCEEDS NORMAL LIMITS; TYPICALLY FOCAL AND/OR SPORADIC, AND OF NO BIOLOGIC RELEVANCE.
**Severity Grade 2 (Mild)**	INFILTRATE MORE READILY APPARENT THAN A MINIMAL INFILTRATE BUT STILL UNLIKELY TO PRODUCE ANY STRUCTURAL OR FUNCTIONAL IMPAIRMENT; MAY BE PRESENT IN ONE OR MORE FOCI.
**Severity Grade 3 (Moderate)**	INFILTRATE READILY APPARENT; POSSIBLE, BUT STILL CONSIDERED UNLIKELY, TO PRODUCE ANY FUNCTIONAL IMPAIRMENT; MAY BE FOCAL, MULTIFOCAL, OR DIFFUSE.
**Severity Grade 4 (Marked)**	INFILTRATE THAT IS PROMINENT, CONSPICUOUS, BUT NOT CONSIDERED TO BE THE MAXIMUM POSSIBLE EFFECT. MAY PRODUCE STRUCTURAL/FUNCTIONAL IMPAIRMENT.
**Severity Grade 5 (Severe)**	INFILTRATE THAT IS PROMINENT, CONSPICUOUS, AND EFFACES TISSUE STRUCTURE; LIKELY TO PRODUCE STRUCTURAL/FUNCTIONAL IMPAIRMENT.

INFILTRATE, MONONUCLEAR CELLS

RECOGNIZABLE LEUKOCYTE INFILTRATE OF LYMPHOCYTES (POSSIBLY INCLUDING PLASMA CELLS) AND/OR MACROPHAGES TYPICALLY WITHOUT ASSOCIATED INDICATORS OF TISSUE DAMAGE.

**Severity Grade 1 (Minimal)**	INFILTRATE BARELY EXCEEDS NORMAL LIMITS; TYPICALLY FOCAL AND/OR SPORADIC, AND OF NO BIOLOGIC RELEVANCE; VERY COMMON CHANGE IN CONTROL ANIMALS.
**Severity Grade 2 (Mild)**	INFILTRATE MORE READILY APPARENT THAN A MINIMAL INFILTRATE BUT STILL UNLIKELY TO PRODUCE ANY STRUCTURAL OR FUNCTIONAL IMPAIRMENT; MAY BE PRESENT IN ONE OR MORE FOCI.
**Severity Grade 3 (Moderate)**	INFILTRATE READILY APPARENT; POSSIBLE, BUT STILL CONSIDERED UNLIKELY, TO PRODUCE ANY FUNCTIONAL IMPAIRMENT; MAY BE FOCAL, MULTIFOCAL, OR DIFFUSE.
**Severity Grade 4 (Marked)**	INFILTRATE THAT IS PROMINENT, CONSPICUOUS, BUT NOT CONSIDERED TO BE THE MAXIMUM POSSIBLE EFFECT. MAY PRODUCE STRUCTURAL/FUNCTIONAL IMPAIRMENT.
**Severity Grade 5 (Severe)**	INFILTRATE THAT IS PROMINENT, CONSPICUOUS, AND EFFACES TISSUE STRUCTURE; LIKELY TO PRODUCE STRUCTURAL/FUNCTIONAL IMPAIRMENT.

Appendix 10

Diagnosis Explanation Report

INFLAMMATION

RECOGNIZABLE LEUKOCYTE INFILTRATE IN ASSOCIATION WITH OTHER INDICATORS OF TISSUE DAMAGE; THESE INDICATORS MAY INCLUDE EDEMA, FIBROSIS, GLIOSIS, HEMORRHAGE, NECROSIS, VASCULAR CONGESTION, ADHESIONS, ETC.; MAY BE DIAGNOSED IN ASSOCIATION WITH VARIOUS INFILTRATES.

**Severity Grade 1 (Minimal)**	CHANGES BARELY EXCEED NORMAL LIMITS; TYPICALLY FOCAL AND/OR SPORADIC, AND TYPICALLY OF NO BIOLOGIC RELEVANCE; AFFECTS A SMALL PERCENTAGE (LESS THAN 1%) OF A TISSUE OR A REGION.
**Severity Grade 2 (Mild)**	CHANGES ARE MORE READILY APPARENT THAN MINIMAL BUT STILL UNLIKELY TO PRODUCE ANY STRUCTURAL OR FUNCTIONAL IMPAIRMENT; MAY BE PRESENT IN ONE OR MORE FOCI; AFFECTS LESS THAN 5% OF A TISSUE OR REGION.
**Severity Grade 3 (Moderate)**	CHANGES ARE READILY APPARENT; POSSIBLE, BUT STILL CONSIDERED UNLIKELY, TO PRODUCE ANY FUNCTIONAL IMPAIRMENT; MAY BE FOCAL, MULTIFOCAL, OR DIFFUSE; AFFECTS 5 TO 10% (ESTIMATED) OF A TISSUE OR REGION.
**Severity Grade 4 (Marked)**	INFILTRATE THAT IS PROMINENT AND CONSPICUOUS, BUT NOT CONSIDERED TO BE THE MAXIMUM POSSIBLE EFFECT. MAY PRODUCE STRUCTURAL/FUNCTIONAL IMPAIRMENT; TYPICALLY AFFECTS UP TO 40% OF A TISSUE OR REGION.
**Severity Grade 5 (Severe)**	INFILTRATE THAT IS PROMINENT, CONSPICUOUS, AND EFFACES TISSUE STRUCTURE; LIKELY TO PRODUCE STRUCTURAL/FUNCTIONAL IMPAIRMENT; TYPICALLY AFFECTS MORE THAN 40% OF A TISSUE OR REGION.

MINERALIZATION

VARIABLY-SIZED (TYPICALLY FOCAL/MULTIFOCAL) AREAS OF CALCIFICATION; OFTEN PERIVASCULAR, ESPECIALLY IN THE BRAIN/SPINAL CORD. MAY INDICATE PRIOR NECROSIS (DYSTROPHIC CALCIFICATION) OF TISSUE, BUT MORE TYPICALLY IS AN INCIDENTAL FINDING. COMMONLY OBSERVED IN CONTROL ANIMALS; TYPICALLY OF NO BIOLOGIC RELEVANCE AT MINIMAL TO MILD SEVERITY.

**Severity Grade 1 (Minimal)**	ONE OR A FEW FOCI; OF NO BIOLOGIC RELEVANCE; OFTEN OBSERVED IN CONTROLS AT THIS LEVEL OF SEVERITY.
**Severity Grade 2 (Mild)**	MORE THAN A FEW FOCI; TYPICALLY OF NO BIOLOGIC RELEVANCE UNLESS THERE IS AN INDICATION OF PRIOR TISSUE NECROSIS; OCCASIONALLY OBSERVED IN CONTROLS AT THIS SEVERITY LEVEL.
**Severity Grade 3 (Moderate)**	MORE THAN A FEW FOCI AND/OR AFFECTS A FOCALLY EXTENSIVE AREA.
**Severity Grade 4 (Marked)**	PRONOUNCED CHANGE AFFECTING A FOCALLY EXTENSIVE TO MORE DIFFUSE REGION; LIKELY INDICATES A PATHOLOGIC PROCESS AND/OR PRIOR TISSUE NECROSIS.
**Severity Grade 5 (Severe)**	PRONOUNCED CHANGE THAT EFFACES A PROMINENT PORTION OF THE TISSUE; LIKELY INDICATES A PATHOLOGIC PROCESS AND/OR PRIOR TISSUE NECROSIS.

Diagnosis Explanation Report

MITOTIC FIGURES, INCREASED

INCREASED NUMBERS OF MITOTIC FIGURES WITHIN CELLS THAN IS SEEN IN CONCURRENT CONTROL TISSUE CELLS; TYPICALLY OBSERVED AS A REPARATIVE OR REGENERATIVE PROCESS.

**Severity Grade 1 (Minimal)**	MITOTIC FIGURES INFREQUENTLY SEEN THROUGHOUT TISSUE SECTION BUT AT INCREASED NUMBER COMPARED TO CONCURRENT CONTROLS.
**Severity Grade 2 (Mild)**	AVERAGE OF 2-3 MITOTIC FIGURES PER FIELD USING 20X OBJECTIVE.
**Severity Grade 3 (Moderate)**	AVERAGE OF 4-6 MITOTIC FIGURES PER FIELD USING 20X OBJECTIVE.
**Severity Grade 4 (Marked)**	AVERAGE OF 7-10 MITOTIC FIGURES PER FIELD USING 20X OBJECTIVE.
**Severity Grade 5 (Severe)**	GREATER THAN 10 MITOTIC FIGURES PER FIELD USING 20X OBJECTIVE.

NECROSIS

THE DEATH OF MOST OF ALL OF THE CELLS IN A TISSUE OR TISSUE REGION. NECROSIS MAY AFFECT INDIVIDUAL CELLS OR MAY AFFECT BROADER REGIONS OF TISSUE. WHEN AFFECTING A BROADER REGION OF TISSUE, THE NATURAL ARCHITECTURE OF THE TISSUE MAY NO LONGER BE EVIDENT. AREAS OF NECROSIS MAY FILL WITH NECROTIC DEBRIS, FLUID, OR MAY SCAR WITH CONNECTIVE TISSUE OR, IN THE CENTRAL NERVOUS SYSTEM, GLIAL CELLS/ASTROCYTES.

**Severity Grade 1 (Minimal)**	AFFECTS A FEW CELLS OR A VERY SMALL PERCENTAGE (<1%) OF A TISSUE OR TISSUE REGION/SITE.
**Severity Grade 2 (Mild)**	AFFECTS MORE THAN A FEW CELLS OR INFREQUENT CELLS BUT IN A LARGER PORTION OF THE TISSUE, OR A LARGER REGION OF A TISSUE (UP TO 5%) AS COMPARED TO A MINIMAL GRADE.
**Severity Grade 3 (Moderate)**	AFFECTS UP TO 15% OF A PARTICULAR CELL TYPE OR UP TO 15% OF A TISSUE OR TISSUE REGION.
**Severity Grade 4 (Marked)**	AFFECTS UP TO 40% OF A PARTICULAR CELL TYPE OR UP TO 40% OF A TISSUE OR TISSUE REGION.
**Severity Grade 5 (Severe)**	AFFECTS >40% OF A PARTICULAR CELL TYPE OR >40% OF A TISSUE OR TISSUE REGION.

Diagnosis Explanation Report

NECROSIS, SINGLE CELL

THIS DIAGNOSIS INDICATES THE OCCASIONAL OBSERVATION OF A SINGLE NECROTIC CELL. THIS CHANGE OFTEN ACCOMPANIES FOCI OF INFILTRATES IN THE LIVER WHERE THE INFILTRATES ARE COMPOSED OF A FEW MACROPHAGES RESPONDING TO THE NECROSIS OF A SINGLE HEPATOCYTE. THE CHANGE MAY BE USED TO RECORD THE RARE/SCATTERED OBSERVATION OF SINGLE NECROTIC CELLS IN ANY TISSUE.

**Severity Grade 1 (Minimal)**	ONE OR A FEW SCATTERED, NECROTIC CELLS.
**Severity Grade 2 (Mild)**	MORE THAN A FEW SCATTERED, NECROTIC CELLS.
**Severity Grade 3 (Moderate)**	MANY BUT STILL SCATTERED INDIVIDUAL NECROTIC CELLS.
**Severity Grade 4 (Marked)**	MANY SINGLE NECROTIC CELLS THROUGHOUT THE ENTIRE TISSUE SECTION.
**Severity Grade 5 (Severe)**	MANY SINGLE NECROTIC CELLS THROUGHOUT THE ENTIRE TISSUE SECTION SUCH THAT THE SINGLE CELLS ARE APPROXIMATING CLUSTERS OF FOCAL NECROSIS.

PIGMENT

THIS DIAGNOSIS INDICATES THE PRESENCE OF A COLORED MATERIAL. THE MOST COMMON PIGMENTS IN THE BRAIN ARE HEMOSIDERIN (BLOOD BREAKDOWN PIGMENT; THIS IS USUALLY DIAGNOSED SEPARATELY) AND LIPOFUSCIN /CEROID (YELLOW-GOLD). HOWEVER, A VARIETY OF SUBSTANCES MAY BE PRESENT AND DIAGNOSED AS PIGMENT.

**Severity Grade 1 (Minimal)**	ONE OR A FEW FOCI PRESENT.
**Severity Grade 2 (Mild)**	MORE THAN A FEW FOCI PRESENT.
**Severity Grade 3 (Moderate)**	FOCAL/MULTIFOCAL ACCUMULATION.
**Severity Grade 4 (Marked)**	FILLS A FOCALLY EXTENSIVE OR MORE DIFFUSE REGION.
**Severity Grade 5 (Severe)**	FILLS A DIFFUSE REGION.

Diagnosis Explanation Report

PIGMENT, HEMOSIDERIN

PRESENCE OF HEMOSIDERIN (BLOOD BREAKDOWN PIGMENT); INDICATES SOME PRIOR HEMORRHAGE/EXTRAVASATION OF RED BLOOD CELLS; THE PRESENCE OF HEMOSIDERIN ALONE IS NOT TYPICALLY BIOLOGICALLY RELEVANT. SIGNIFICANT ACCUMULATIONS (MARKED AND SEVERE) MAY INDICATE SIGNIFICANT, PRIOR HEMORRHAGE.

**Severity Grade 1 (Minimal)**	ONE OR A FEW FOCI PRESENT.
**Severity Grade 2 (Mild)**	MORE THAN A FEW FOCI PRESENT.
**Severity Grade 3 (Moderate)**	FOCAL/MULTIFOCAL ACCUMULATION.
**Severity Grade 4 (Marked)**	FILLS A FOCALLY EXTENSIVE OR MORE DIFFUSE REGION.
**Severity Grade 5 (Severe)**	FILLS A DIFFUSE REGION.

RETINAL ROSETTE

FOCAL TO MULTIFOCAL "ROSETTE-LIKE" AND TUBULAR STRUCTURES EXPANDING THE INNER AND OUTER NUCLEAR LAYERS OF THE RETINA DUE TO FAULTY DEVELOPMENT.

**Severity Grade 1 (Minimal)**	FOCAL LESION AFFECTING LESS THAN 10% OF RETINA.
**Severity Grade 2 (Mild)**	FOCAL OR MULTIFOCAL LESION AFFECTING 10-25% OF RETINA.
**Severity Grade 3 (Moderate)**	FOCAL OR MULTIFOCAL LESION AFFECTING 26-50% OF RETINA.
**Severity Grade 4 (Marked)**	FOCAL OR MULTIFOCAL LESION AFFECTING 51-75% OF RETINA.
**Severity Grade 5 (Severe)**	FOCAL OR MULTIFOCAL LESION AFFECTING GREATER THAN 75% OF RETINA.

Appendix 10

Diagnosis Explanation Report

VACUOLATION

PRESENCE OF VACUOLES/SPACES IN A TISSUE; COMMON ARTIFACTUAL CHANGE, BUT TYPICALLY DIAGNOSED ONLY WHEN SUSPECTED TO BE AN ACTUAL (IN LIFE/ANTEMORTEM) CHANGE. MAY BE INTRACYTOPLASMIC (OFTEN IN ASTROCYTES OR NEURONS), INTRANUCLEAR, OR WITHIN MYELIN SHEATHS; UNCOMMON CHANGE IN CONTROLS.

**Severity Grade 1 (Minimal)**	ONE OR A FEW FOCI (OR AFFECTED CELLS) PRESENT.
**Severity Grade 2 (Mild)**	MORE THAN A FEW FOCI (OR AFFECTED CELLS) PRESENT.
**Severity Grade 3 (Moderate)**	FOCALLY EXTENSIVE TO MORE DIFFUSE AREA/GROUPS OF CELLS AFFECTED.
**Severity Grade 4 (Marked)**	PROMINENT PORTION OF THE TISSUE AND/OR CELL TYPE AFFECTED.
**Severity Grade 5 (Severe)**	GREATER THAN 40% OF THE REGION/CELLS AFFECTED.

Appendix 10

APPENDIX 1: QUALITY ASSURANCE STATEMENT

Appendix 10

QUALITY ASSURANCE STATEMENT

Study Title: A Single Dose Toxicity Study of AAV9/SURF1 Administered by Intrathecal Injection in Rats

Study Number: 2954-001

StageBio Document Identification (ID): SBDOC004328

The following have been inspected by the StageBio Quality Assurance Unit (QAU) as required by the Good Laboratory Practice (GLP) regulations disseminated by the U.S. Department of Health and Human Services, Food and Drug Administration, United States Code of Federal Regulations, Title 21, Part 58: Good Laboratory Practice for Nonclinical Laboratory Studies and as accepted by Regulatory Authorities throughout the European Union (OECD Principles of Good Laboratory Practice), Japan (MHLW), and other countries that are signatories to the OECD Mutual Acceptance of Data Agreement, as well as the study protocol and amendments, and applicable StageBio SOPs.

Area Inspected	Date(s) of Inspection	Date Inspection Reported to Principal Investigator and Test Site Management	Date Inspection Reported to Study Director, Test Facility Management, and Lead QAU (as applicable)
Draft Pathology Report	25, 27 MAY, 02-03 JUN 2022	07 JUN 2022	07 JUN 2022
Final Pathology Report	31 OCT 2022	02 NOV 2022	02 NOV 2022

The report is an accurate reflection of the raw data.

Appendix 10

Signature Page

SBDOC004328: QAS 2954-001 Final Pathology Report

StageBio Project ID: 02776-0018

Testing Facility: Charles River Laboratories, Inc. / 2954-001

Sponsor: University of Texas Southwestern Medical Center / UTSW.Gray-002

Title: A SINGLE DOSE TOXICITY STUDY OF AAV9/SURF1 ADMINISTERED BY INTRATHECAL INJECTION IN RATS

Reason for Signing	Signature
I am the author of this Quality Assurance Statement	476B283B-B4F8-44D6-9D7B-FFE31438E3A0<br>Beth Taylor<br>11/2/2022 11:17:01 AM<br>Quality Assurance

APPENDIX 2: SUMMARY OF GLP, PROTOCOL, AND SOP DEVIATIONS

<u>SOP Deviation:</u>

1. Per SOP 214v03 Section 3.9 "each employee documents review and comprehension of the [Study Instruction Sheet] SIS … via BioDox … prior to performing work on a study." It was found that three StageBio employees inadvertently did not sign off on any version of the SIS despite having already performed work on the study, deviating from SOP 214v03. There is no resolution as the employees in question have already performed work on the study; however, the only study work they performed was inventory of slides to or from the Study Pathologist, documented on Form 208-1 Internal Transfer of Materials Record. The Study Pathologist received all slides for evaluation and slides are accounted for. This deviation did not impact the pathology data or the Study Pathologist's evaluation.
2. SOP 212v2 Section 4.1. states "It is the responsibility of all employees to ensure all data is recorded accurately and correctly in accordance with Good Laboratory Practice regulations and this SOP". This deviation documents instances on an Exit Inventory Sheet dated 06OCT2021 where slides for Animals 3009 and 2013 were not recorded and slide totals for Animals 3014, 3015, 1515, and 2523 were recorded incorrectly; an Inventory Sheet dated 26OCT2021 where slide totals for Animals 3014, 3015, and 2523 were recorded incorrectly; an Internal Transfer of Material Records prepared on 26OCT2021 where slide totals for Animals 3014, 3015, and 2523 were recorded incorrectly; and an Internal Transfer of Material Records prepared on 13DEC2021 where slides totals for Animals 3014, 3015, 2523, and 4521 were incorrectly recorded. There is no impact on the quality and integrity of the histology and/or pathology data as slides were verified to be produced and were evaluated by the Study Pathologist.

There were no additional GLP, protocol or SOP deviations during the phases of this study performed by StageBio.

APPENDIX 3: ABBREVIATIONS AND TERMS POSSIBLY USED IN THE DATA SECTIONS

Appendix 10

Abbreviations and Terms Possibly Used in the Data Sections	
**Abbreviation/Term**	**Explanation**
**TISSUE DISPOSITION ABBREVIATIONS**	
N	Normal; The tissue was examined and had no microscopic findings which indicated abnormality.
N1	Normal unilateral/not examined unilateral. The tissue was examined unilaterally and had no microscopic findings which indicated abnormality.
A	Abnormal; The tissue examined had at least one microscopic finding which indicated abnormality.
T	Not examined; The tissue was unavailable for evaluation because it was not present at trimming or at necropsy.
R	Recut/retrim/reembed pending; A pathology request has been submitted for the tissue but has not yet been evaluated.
U	Not examined; The tissue was unavailable for evaluation because it was not present on the slide.
X	The tissue was unavailable for evaluation because it was autolytic to a degree that prevented adequate microscopic examination.
E	Examined; The tissue was examined but other dispositions are not applicable. This is typically used to indicate stains evaluated for a particular tissue.
**TISSUE SEVERITY ABBREVIATIONS**<br>These are the general definitions for the various grades assigned to microscopic findings. For the specific grading schemes used, see the Diagnosis Explanations Report.	
P<br>Present	A non-gradable histologic change where no further information would be added by the assignment of a numerical grade. Examples include neoplasms, presence of a device, presence of a device track, etc.
1<br>Minimal	Severity grade minimal<br>A microscopic change that barely exceeds normal limits or what would be considered the normal condition. Typically, these are microscopic changes that are sporadic, focal, and of no biologic significance to the function or structure of the tissue. Microscopic changes graded as "1" are not readily apparent in the tissue. Generally, a minimal change affects less than 1% of the total tissue that might be possibly affected.
2<br>Mild	Severity grade mild<br>A microscopic change that is more readily apparent in the tissue than a minimal (Grade 1) change but is still unlikely to produce any structural or functional impairment. Typically, a grade of mild indicates the microscopic change was present in one or a few foci. Generally, a microscopic change graded as mild affects less than 5% of the total tissue that might be possibly affected.
3<br>Moderate	Severity grade moderate<br>A microscopic change that is readily observed in the section, but still of limited severity and unlikely to have any biologically significant effect on overall structure or function of the tissue. Moderate changes may be focal, multifocal, or even diffuse.
4<br>Marked	Severity grade marked<br>A microscopic change that is a prominent, conspicuous, and easily identified feature of the tissue, but is still not present at what would be considered to be the maximum possible effect. A marked change would reasonably be expected to have some effect on the structure and/or function of a tissue, although the correlating functional change may or may not be apparent.
5<br>Severe	Severity grade severe<br>A microscopic change that is prominent, conspicuous, and easily identified feature of the tissue, is present at a maximum severity (affects the majority of the tissue and/or is very pronounced in one or more foci), and/or is present at a severity that would be expected to have a prominent effect on the structure and/or function of a tissue, although the correlating functional change may or may not be apparent.

Abbreviations and Terms Possibly Used in the Data Sections	
**Abbreviation/Term**	**Explanation**
MISCELLANEOUS ABBREVIATIONS	
INC	Incidence. Indicates the number of animals in a particular dose group with the given diagnosis.
SEV	Average Severity. Calculated as the sum of the severity scores for a specific diagnosis at a specific site/tissue, divided by the number of animals with that particular site/tissue examined.
-	A dash is used to indicate that specific cell/field contains no other data. The dash is used in lieu of a cell/field remaining empty.
TGL	Tissue Gross Lesion. Tissue gross lesions will be numbered sequentially, beginning with 1, to include all lesions present on the necropsy sheet as well as those found at trim. Gross lesions on the necropsy sheet and those found at trim will be recorded.
M	Male
F	Female
SS	Scheduled Sacrifice
MS	Moribund Sacrifice
FD	Found Dead
US	Unscheduled Sacrifice
ED	Early Death
HE	Hematoxylin and Eosin
Long	Longitudinal
SGC	Satellite Glial Cells
D08	Day 8
D29	Day 29
D91	Day 91
SEND	Standard for Exchange of Non-Clinical Data
CDISC	Clinical Data Interchange Standards Consortium

APPENDIX 4: GLOSSARY OF ANATOMIC TERMS

Appendix 10

Anatomic Terms	Definitions
**Brain Regions**	
Brain, Amygdaloid Body/Amygdalae (Note: Amygdaloid Body is the term used in the SEND glossary)	Amygdalae: The amygdalae are bilateral nuclei groups in the ventral/medial aspects of the region of the temporal lobe. The amygdala is part of the limbic system and contributes to processing and memory of emotional reactions, playing an important role in anxiety and depression. It is composed of at least twelve nuclei that perform different functions. The amygdala sends inputs to the hypothalamus, thalamic reticular nucleus, trigeminal nerve nuclei, facial nerve nucleus, ventral tegmental area, locus coeruleus, hippocampus, and laterodorsal tegmental nucleus. It receives inputs from the olfactory bulb and olfactory cortex to process the sense of smell.
Brain, Basal Nuclei/Striatum/Caudate Nucleus/Putamen/Globus Pallidus	The striatum region is a subcortical (i.e. inside, rather than on the outside) part of the telencephalon. Anatomically, the dorsal striatum is the caudate nucleus and the putamen. The dorsal striatum forms a continuous and large mass, topographically separated by the internal capsule (in some species; this separation is not readily apparent in rodents) into the caudate nucleus medially and the putamen. The globus pallidus is part of the basal nuclei system. Mammalian basal nuclei are associated with a variety of functions: motor control, cognition, emotions, and learning.<br><br>Globus pallidus: The globus pallidus is composed of both the dorsal and ventral pallidus. The dorsal pallidus is divided into the internal and external pallidus by the medial medullary lamina. The internal pallidus contains GABAergic neurons that send their axons to the dorsal thalamus, the centromedian complex, substantia nigra and the pedunculopontine complex. Fibers from the external pallidus are the main regulators of the basal ganglia system. The ventral pallidum is involved in the regulation of motivation, behavior, and emotions. The globus pallidus receives input from the temporal lobes and the hippocampus via the ventral striatum and transmits this information to the medial dorsal and ventral anterior nuclei of the thalamus.
Brain, Basal Nuclei of Meynert/Diagonal Bands/Medial Septal Nuclei	Basal nuclei (of Meynert): Groups of cholinergic neurons within the substantia innominata. Other cholinergic neuron groups include the diagonal bands and the medial septal nuclei.

Appendix 10

Anatomic Terms	Definitions
Brain, Cerebellum	The cerebellum is a region of the brain that plays an important role in the integration of sensory perception, coordination and motor control. There are three layers to the cerebellar cortex; from outer to inner layer, these are the molecular, Purkinje, and granular layers. The cerebellum is located in the inferior posterior portion of the head (the hindbrain), directly dorsal to the pons, and inferior (caudal) to the occipital lobe. Because of its large number of tiny granule cells, the cerebellum contains more than 50% of all neurons in the brain, but it only takes up 10% of total brain volume. The cerebellar cortex receives most of its inputs from extracerebellar sources such as the vestibular nerve, spinal cord, reticular formation, olivary nucleus, and cerebral cortex (via the pontine nuclei). Its primary output (via the Purkinje neurons) is to the deep cerebellar nuclei. The cerebellum plays an important role in motor control such as coordination and precision, but it is also involved in cognitive functions of attention and language.
Brain, Cerebellum, Deep Cerebellar Nuclei	Deep cerebellar nuclei: The deep cerebellar nuclei are composed of the three nuclei, the fastigial, interposed, and dentate nuclei. The fastigial nucleus, which is the most medially located cerebellar nucleus, contains afferent axons from the cerebellar vermis and sends efferent axons to the vestibular nuclei and reticular formation. The interposed nucleus is located between the dentate and fastigial nuclei and has two distinct subdivisions, the globose and emboliform nuclei. Both subdivisions receive inputs from the cerebellar cortex of the medial hemisphere and project (via the cerebellar peduncles) to the red nucleus and ventrolateral nucleus of the thalamus. The dentate nucleus receives inputs from the cerebellar cortex and sends (via the cerebellar peduncles) outputs to the red nucleus, inferior olivary nucleus, and thalamus.
Brain, Cerebellum, Granular Layer	Innermost layer of the cerebellar cortex; This layer contains numerous, densely packed, small neurons.
Brain, Cerebellum, Molecular Layer	The outermost layer of the cerebellar cortex.
Brain, Cerebral Cortex	The dorsal telencephalon. The cerebral cortex directs the conscious functions of the body. These functions originate within the primary motor cortex and other frontal lobe motor areas where actions are planned.
Brain, Cingulate Gyrus/Cortex	The cingulate cortex is dorsal to the corpus callosum and is part of the limbic system, which is involved with formation and processing of emotions, learning, and memory. The rostral (anterior) and the caudal (posterior) portions of the cingulate cortex are related to the amygdala and hippocampus, respectively. This area is classically affected by a variety of chemicals that have N-methyl-D-aspartate (NMDA) receptor antagonist activity. The retrosplenial portion of the cingulate gyrus indicates that portion near the splenium (posterior portion) of the corpus callosum.

Appendix 10

Anatomic Terms	Definitions
Brain, Entorhinal Cortex	The entorhinal cortex covers the anterior part of the parahippocampal gyrus and receives afferent inputs from the amygdala, olfactory bulb, limbic areas, and neocortex. The entorhinal cortex sends most of its efferent outputs to the hippocampus and plays an important role in spatial memories, including memory formation and consolidation of memories during sleep.
Brain, Frontal Cortex	One of the four major lobes of the brain (with parietal, temporal, and occipital); Best defined in the larger species (monkeys; dogs).
Brain, Cerebral Cortex, Visual Cortex	The visual cortex is composed of six functionally distinct regions within the occipital lobe. The primary visual cortex receives information directly from the lateral geniculate nucleus and processes information about position and movement of objects and pattern recognition. The secondary visual cortex is responsible for transmitting messages with respect to motion, shape, and position via intracortical pathways.
Brain, Gray Matter, General	Those portions of the brain or spinal cord containing neuronal cell bodies.
Brain, Gray Matter, Inferior Colliculus	Prominent midbrain nucleus (group of neurons) involved in the auditory pathway.
Brain, Superior Colliculus	Rostral (superior) colliculus: The rostral colliculus receives afferent axons from the retina and visual cortex and sends efferent axons to the pulvinar nucleus and other thalamic nuclei in order to play an important role in directing visual attention and controlling eye movements.
Brain, Hippocampus	The hippocampus is a specialized cortical area integrated in the temporal lobe and belongs to the limbic system, playing important roles in long-term memory and spatial navigation. It is composed of different neuronal types that are organized into layers. Anatomically (in coronal sections), the hippocampus is composed of the dentate gyrus, hippocampus proper, and subiculum. The dentate gyrus receives afferent input from the entorhinal cortex and sends axons to the hippocampal pyramidal cells. The hippocampus proper (or Ammon's horn) receives afferent inputs from the dentate gyrus and septal nuclei and sends efferent inputs to the subiculum and septal nuclei. The subiculum is a transitional area between the hippocampus proper and entorhinal cortex and represents the main output of the hippocampus.
Brain, Medulla Oblongata	The medulla oblongata is the lower portion of the brainstem. It deals with autonomic functions, such as breathing and blood pressure. Numerous nuclei are in the medulla oblongata, including the nuclei for cranial nerves 5 (trigeminal nerve) to 12 including the cochlear nucleus (cranial 8) and the brainstem nuclei associated with the vagus nerve.

Anatomic Terms	Definitions
Brain, Meninges	The meninges (singular meninx) are the system of membranes which envelope the central nervous system. The meninges consist of three layers: the dura mater, the arachnoid mater, and the pia mater. The primary function of the meninges is to protect the central nervous system. Typically, in brain sections, only the pia and arachnoid layers are examined, as the dura is usually removed with the skull. In spinal cord sections, ideally all three layers are evaluated.
Brain, Midbrain	The mesencephalon (or midbrain) contains various nuclei (including the substantia nigra). The mesencephalon is between the pons (metencephalon) caudally and the diencephalon (thalamus) rostrally.
Brain, Nucleus Accumbens	Part of the basal nuclei group; located in the ventral striatum (below the rostral caudate putamen region).
Brain, Nucleus Ambiguus	Cluster of motor neurons in the reticular formation of the medulla oblongata; innervate the soft palate/pharyngeal region.
Brain, Nucleus, Cochlear	Neuron group in the medulla oblongata, which received axons from cranial nerve 8 (auditory nerve).
Brain, Olfactory Bulb	That portion of the cerebral cortex that develops in association with the olfactory system and, as used in the data tables, includes the olfactory bulbs, olfactory tract, and the piriform cortex. This area is specifically listed because of the prominence of this system in dogs and rodents. In primates, this system is less developed/prominent.
Brain, Pons	A region/group of structures (including pontine nuclei and the transverse fibers of the pons) located in the rostral portion of the brain stem. The pons region is cranial to the medulla oblongata, caudal to the midbrain, and ventral to the cerebellum. The pons relays sensory information between the cerebellum and cerebrum. As used in the data tables, the term "Pons" refers to the pons region.

Anatomic Terms	Definitions
Brain, Thalamus/Hypothalamus	The thalamus, which is often referred to as ''the gateway to the cortex,'' contains nineteen different association or relay nuclei that pass information to the cortex from the spinal cord, brainstem, cerebellum, and basal ganglia (as well as from other regions of the cerebral cortex). The thalamus, therefore, functions to relay sensation, special sense stimuli (excluding olfaction), and motor signals to the cerebral cortex and is also involved in the regulation of consciousness, sleep, and alertness. The anterior thalamic nucleus is a key link between the limbic system and the cortex. The lateral geniculate nuclei receive input from the retina and relay information to the primary visual cortex. The medial geniculate nuclei receive input from the inferior colliculus and send outputs to the primary auditory cortex. The ventral posterior nuclei (both lateral and medial) play key roles in bodily perception, relaying touch and proprioceptive information from the dorsal columns (spinal cord) and trigeminal nuclei to the primary somatosensory cortex. Thalamic nuclei have strong reciprocal connections with the cerebral cortex, forming thalamo-cortico-thalamic circuits that are believed to be involved with consciousness. In addition, the thalamus has connections with the cerebellum for controlling motor functions.<br><br>Hypothalamus: Multiple small hypothalamic nuclei (medial preoptic, paraventricular, anterior hypothalamic, suprachiasmatic, lateral preoptic, lateral, dorsomedial hypothalamic, ventromedial, arcuate, lateral tuberal, posterior, and mammillary nuclei) link the nervous system to the endocrine system via the pituitary gland (hypophysis).
Brain, Ventricular System	The ventricular system is a set of cavities, lined by ependymal cells, in the brain; these cavities are continuous with the central canal of the spinal cord. The two lateral ventricles, located within the cerebrum, are relatively large and C-shaped, roughly wrapping around the dorsal aspects of the basal nuclei. Each lateral ventricle extends into the frontal, temporal and occipital lobes. The lateral ventricles communicate via the interventricular foramina with the third ventricle. The third ventricle is within the diencephalon; it communicates via the cerebral aqueduct (located within the midbrain) with the fourth ventricle (found within the hindbrain). The three foramina to the subarachnoid space are connected to the fourth ventricle, permitting cerebrospinal fluid produced in the ventricles to surround the brainstem, cerebellum, and cerebral cortex. The fourth ventricle is continuous with the central canal of the spinal cord.
Brain, Ventricular System, Choroid Plexus	Tufts of blood vessels, covered by choroid plexus epithelium (modified ependymal cells), present in the lateral and fourth ventricles (and dorsal aspect of the third ventricle/interventricular foramen). The cerebrospinal fluid is produced by the choroid plexus.

Anatomic Terms	Definitions
Brain, White Matter, Anterior Commissure	This white matter structure consists of bundles of nerve fibers that cross the midline of the brain, connecting the two cerebral hemispheres. Specifically, it connects the left and right temporal lobes and amygdalae (RA), contains decussating fibers projecting from the olfactory tracts to the opposite side of the olfactory cortex, and forms part of the neospinothalamic tract for pain perception.
Brain, White Matter, Fornix	A large white matter tract that connects the hippocampus to the thalamus.
Brain, White Matter, Corona Radiata/External Capsule	The white matter in the cerebrum that lies just beneath the cerebral cortical tissue.
White Matter, Brain, Trapezoid Body	Transverse fibers (axons) in the pons region. This is a common site of slight, nerve fiber degeneration in the rat.
Brain, White Matter, Corpus Callosum	The large white matter tract that connects the two cerebral cortical hemispheres
**Spinal Cord Regions**	
Spinal Cord, Cervical	The spinal cord containing the segments from which the spinal nerves C1 (cervical nerve one) to C8 (cervical nerve eight) are produced. These segments are within the seven cervical vertebrae.
Spinal Cord, Thoracic	The spinal cord containing the segments from which the spinal nerves T1 (thoracic nerve one) to T12-18 (thoracic nerve 12 to 18; the number of thoracic vertebrae vary in the various laboratory animals) are produced. These segments generally correspond to the thoracic vertebrae with the same number, except the caudal segments may be slightly cranial to the analogous vertebrae.
Spinal Cord, Lumbar	The spinal cord containing the segments from which the lumbar spinal nerves originate. Lumbar vertebrae vary in the various laboratory species. Typically, because the actual spinal cord stops growing prior to the complete growth of the vertebrae, the lumbar spinal cord segments are always a variable distance cranial to the analogous (numerical) lumbar vertebra. The structural effect of this distance is that the dorsal and ventral spinal nerve roots typically traverse a variable distance caudally in the spinal canal prior to exiting caudal to the analogous vertebra. Sacral and coccygeal (tail) segments of the spinal cord are sometimes included with the lumbar cord segments.
Spinal Cord, Sacral	The spinal cord containing the segments from which the sacral spinal nerves originate.
Spinal Cord, Cauda Equina	The termination of the spinal cord. This region is composed (predominantly) of spinal nerve roots coursing caudally in the spinal canal on their way to exit the spinal column. The name derives from the appearance of this collection of spinal nerve roots, which appears similar to a horses tail.

Anatomic Terms	Definitions
Spinal Cord Tracts<br>Dorsal/Lateral/Ventral	A tract is a biologic structure (as opposed to a "track" which is the remnant of or evidence of the presence of an introduced material or structure such as a catheter) that is a collection of nerve fibers generally with a common purpose and/or direction. The dorsal tracts carry (predominantly) nerve fibers that are ascending (heading towards the brain) the spinal cord. Ventral tracts contain (predominantly) descending tracts (from the brain or other spinal cord segments). The lateral tracts contain a combination of both.

Anatomic Terms	Definitions
**Nerves/Ganglia**	
Sciatic/Sural/Peroneal/Tibial Nerves	These are the nerves formed by one or more branches of one or more spinal nerves. The usual peripheral nerves examined are one or more of the following: sciatic, tibial, sural, peroneal (all hind limb) or ulnar, radial (fore limb).
Spinal Nerve Roots/<br>Dorsal Root Ganglion<br>(Cervical, Thoracic, Lumbar, Treatment Site)	Dorsal and ventral spinal nerve roots are formed by numerous rootlets emerging from the dorsal (posterior in primates) or ventral (anterior in primates) region of the spinal cord. The roots curve around the cord and merge at the level of the dorsal root ganglion, which is within the intervertebral foramen (the "hole" formed by the contiguous caudal notch of the cranial vertebrae and the cranial notch of the caudal vertebrae). Typically, the spinal nerve roots and dorsal root ganglion are lumped together in one diagnostic term/region because they are always present together on the same tissue section.
Trigeminal Ganglion/ Trigeminal Nerve	Predominantly sensory ganglion in cranial nerve 5 (the trigeminal nerve). The trigeminal ganglion is similar to a dorsal root ganglion in that it contains predominantly sensory/unipolar neurons.
**Miscellaneous and Anatomic Terms**	
Astrocytes	Astrocytes perform a variety of functions in the central nervous system, including maintenance of ion balance, nutrition of neurons, and biochemical support of other cells. They may play a minor role in maintaining the blood-brain-barrier. The repair functions of astrocytes make them a useful target to identify (often with an immunohistochemical procedure to detect glial fibrillary acidic protein (GFAP)) as a marker for general damage/injury/changes to the brain/spinal cord. The astrocyte is derived from primordial cells forming the primordial neural tube.
Caudal	Towards the tail
Cerebrospinal fluid	Fluid produced by the choroid plexus; Fills the ventricular system and surrounds the brain and spinal cord in the intrathecal/subarachnoid space.
Contralateral	Opposite side (For example: Contralateral to catheter placement.)
Cranial	Towards the head
Dorsal	Towards the back

Anatomic Terms	Definitions
Endoneurium	The connective tissue surrounding each individual nerve fiber. An intact endoneurial tube may allow for the regrowth of an axon to its original target tissue/site.
Epineurium	The outermost layer of connective tissue surrounding a peripheral nerve (including spinal nerve roots/nerves).
Intrathecal space	The subarachnoid space.
Ipsilateral	Same side (For example: Ipsilateral to catheter placement.)
Laminar	Infers a linear pattern parallel to the surface of the brain or other reference surface/structure
Microglial Cells	These cells are (largely) derived from blood monocytes and are essentially central nervous system macrophages. They perform many functions and are highly reactive. Microglial reactions are often the most sensitive means of noting a disturbance/alteration/change in the central nervous system.
Oligodendrocytes	The myelinating cell of the central nervous system. The oligodendrocyte is derived from primordial cells forming the neural tube. They insulate and support axons/dendrites in the central nervous system.
Perineurium	The connective tissue surrounding bundles of nerve fibers.
Perivascular	Around blood vessels
Periventricular	The tissue immediately adjacent to the ventricles (lateral, third, fourth)
Rostral	Towards the nose
Satellite Glial Cell	Satellite Glial cells form a sheath around the pseudo-unipolar neurons in dorsal root ganglia, much the same manner that Schwann cells envelope axons, however, they do not form myelin. These cells are neural crest derived.
Glial Cells	Play a role in the function of the central nervous system under health and disease states and maintain bidirectional communication with neurons. Types of Glial cells include Oligodendrocytes, Microglia, and/or Astrocytes.
Schwann cells	The myelinating cell of the peripheral nervous system. These cells are derived from neural crest cells.
Ventral	Towards the underside/abdomen

Several of the definitions, or parts of definitions, are from the following references:

Haberberger RV, Barry C, Dominguez N, Matusica D. Human Dorsal Root Ganglia. *Frontiers in Cellular Neuroscience*. 2019;13:271.

Hanani M. Satellite Glial Cells in Sympathetic and Parasympathetic Ganglia: in Search of Function. *Brain Research Reviews*. 2010;64(2):304-327.

Hanini M, Spray DC. Emerging Importance of Satellite Glia in Nervous System Function and Dysfunction. *Nature Reviews Neuroscience*. 2020;21(9):485-498.

Pardo ID, Garman RH, Weber K, Bobrowski WF, Hardisty JF, Morton D. Technical Guide for Nervous System Sampling of the Cynomolgus Monkey for General Toxicity Studies. *Toxicologic Pathology*. 2012;40(4):624-636.

SIGNATURE(S) FOR DOCUMENT: 2954-001 Surgery Models Final Report Main Report

**QA Approval:**	I approve the Quality Assurance Statement for this report.		
Name:	**Weideman, Jennifer**		
	Weideman, Jennifer	22-Aug-2023 15:24:09 (UTC+00:00)	
Electronically Signed in	M-Files	Timestamp	

**Study Director Approval:**	I approve this Report.		
Name:	**Davis, Sarah (Mattawan)**		
	Davis, Sarah (Mattawan)	22-Aug-2023 15:24:59 (UTC+00:00)	
Electronically Signed in	M-Files	Timestamp	
